# What distinguishes the strength and the effect of a Lewis base: insights with a strong chromogenic silicon Lewis acid†

**DOI:** 10.1039/d5sc03200h

**Published:** 2025-07-23

**Authors:** Lennart Stoess, Lutz Greb

**Affiliations:** a Anorganisch-Chemisches Institut, Ruprechts-Karls-Universität Heidelberg Im Neuenheimer Feld 275 69120 Heidelberg Germany greb@uni-heidelberg.de

## Abstract

Quantifying Lewis basicity (LB) is essential for understanding chemical reactivity. Yet, the relationship between the intrinsic thermodynamic strength of a Lewis base and the effect it induces at a bound Lewis acid remains poorly defined, especially across structurally diverse bases. Here, we introduce a chromogenic silicon-based Lewis acid to disentangle this relationship *via* optical spectroscopy and computational analysis. Extending our previously developed concept of global and effective Lewis acidity (gLA and eLA), we propose analogous descriptors for Lewis basicity: global Lewis basicity (gLB) and effective Lewis basicity (eLB). Our findings reveal that gLB and eLB are distinct and independently varying properties. However, unlike for Lewis acidity—where the offset of gLA and eLA is governed by deformation energy—the divergence of gLB and eLB is dominated by solvation of the Lewis base. Specifically, solvation energy significantly affects adduct formation thermodynamics (gLB) but has minimal influence on the induced optical response (eLB). Furthermore, the chromogenic probe enables identification of π-type Lewis basicity contributions. The distinction developed in this work refines the conceptual framework of Lewis pair interactions and highlights the importance of solvation and electronic structure when applying LB descriptors in different chemical contexts.

## Abstract 2

A chromogenic Si(iv)-Lewis acid allows rationalizing the difference between the thermodynamic strength and the effect of Lewis bases.

## Graphical abstract

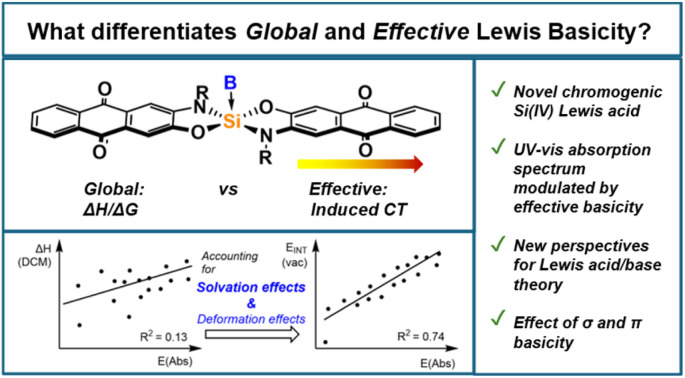

## Introduction

**Fig. 1:**
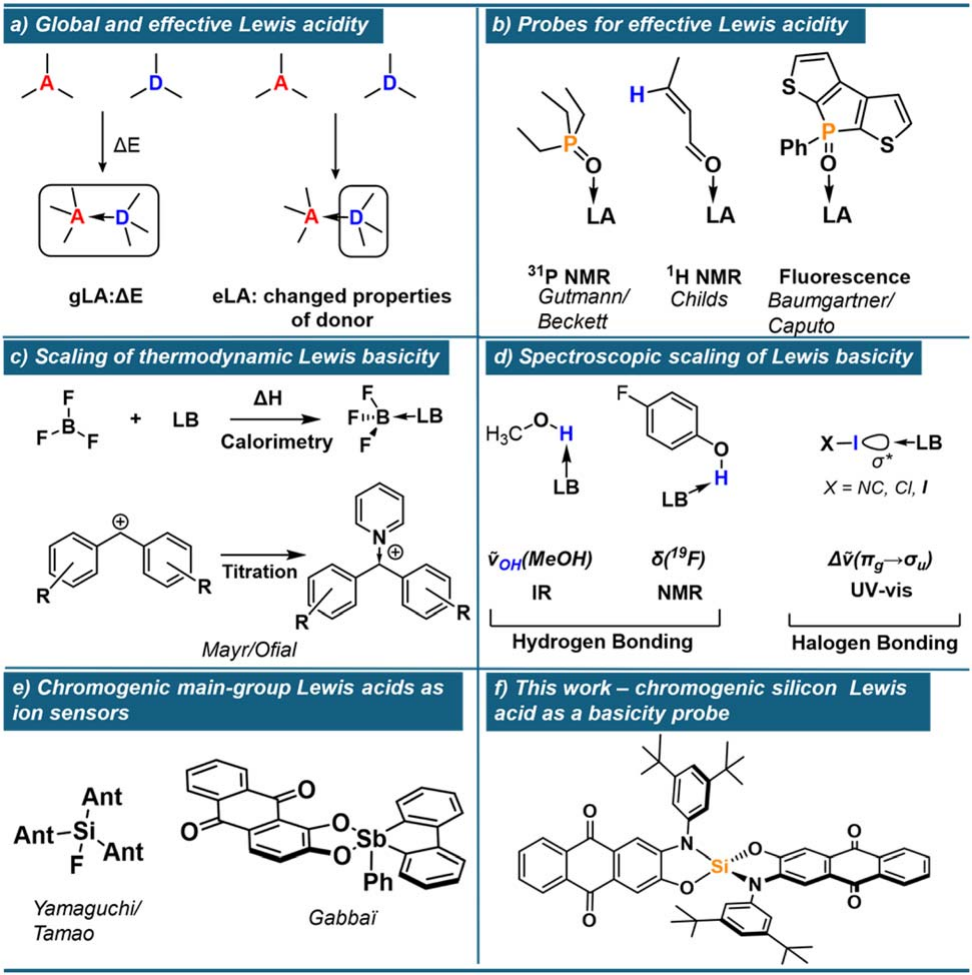
(a) Definitions of global and effective Lewis acidity. (b) Molecular probes for effective Lewis acidity. (c) Methods for the thermodynamics scaling of Lewis basicity. (d) Methods for the spectroscopic scaling of Lewis basicity. (e) Chromogenic Lewis acids for anion sensing. (f) Chromogenic silicon Lewis acid presented in this work.

Scaling Lewis acidity (LA) and Lewis basicity (LB) of molecules and materials is crucial for predicting and rationalising chemical behaviour across diverse fields of chemistry,^1–3^ ranging from solid–liquid interfaces^4^ to synthetic processes.^5^ While numerous methods exist to computationally or experimentally rank Lewis acids and bases, a fundamental question arises regarding the nature of the resulting scales: do they reflect the strength of the interaction (thermodynamics), or merely its consequences (effects)? To emphasise this segregation, we recently introduced the terms global and effective Lewis acidity (gLA and eLA, Fig. 1a), where the former describes the thermodynamic tendency to bind Lewis bases (*e.g.* the fluoride ion affinity, FIA), while the latter deals with the effect of a Lewis acid on a bound substrate.^6^ Prominent eLA scales include the Gutmann–Beckett (GB)^7,8^ and the Childs^9,10^ method, which utilise NMR spectroscopy to measure the induced chemical shift of a phosphine oxide or an α,β-unsaturated carbonyl, respectively (Fig. 1b). More recently, effective methods relying on fluorescence^11^ or IR spectroscopy^12^ have been developed. Importantly, if different classes of Lewis acids are concerned, gLA and eLA do not necessarily correlate. By analysis of >130 Lewis pair interactions, we derived that the main source of difference between gLA and eLA (in its GB variant) stems from the deformation energy of the Lewis acid.^13^ In the present work, we inspect whether the global/effective perspective should be extended to Lewis basicity. Thermodynamic Lewis basicity scales are well-established, using calorimetric data for reference Lewis acids such as SbCl_5_, BF_3_, or hydrogen bond donors.^14^ More recently, the groups of Mayr and Ofial contributed with titration and isothermal calorimetry data with carbocations or boranes (Fig. 1c).^15–21^ Probes for the effect of Lewis basicity have also been developed. Substantial collections of Lewis base induced variations in methanol *ν*(OH)-bond vibrational frequencies (IR),^22^^19^F NMR chemical shifts of 4-fluorophenol^23^ and UV-vis absorption bands of diiodine in their Lewis base adducts have been listed (Fig. 1d).^14^ However, a fundamental limitation lies in the inconsistent relationships among existing scaling methods, which often prove more restricted than suggested by their advocates, with meaningful correlations typically emerging only among structurally similar donors.^14,24,25^ As a result, it remains unclear how—if at all—the induced effects reflect the strength of Lewis bases. Furthermore, although established spectroscopic probes based on hydrogen- or halogen-bonding interactions are *a priori* analogous to conventional Lewis pair formation, they tend to leave steric and electronic characteristics of common p-block Lewis acids out of consideration. This mismatch hinders the development of predictive interpretation in this active realm of chemical research. Hence, we reasoned to implement a suitable probe Lewis acid. While chromogenic main-group Lewis acids have been implemented for sensing purposes (Fig. 1e),^26–33^ their use to investigate variational effects of different Lewis bases have not been reported. Here, we present a chromogenic silicon Lewis acid 1 which sensibly responds to the Lewis base under scrutiny. It allows us to provide a first idea of global Lewis basicity (gLB) and effective Lewis basicity (eLB), and to analyse their correlation. Solvation free energy of the Lewis bases and the presence/absence of π-electrons are identified as the most critical variables that set apart both regimes.

## Results and discussion

### Synthesis

**Fig. 2:**
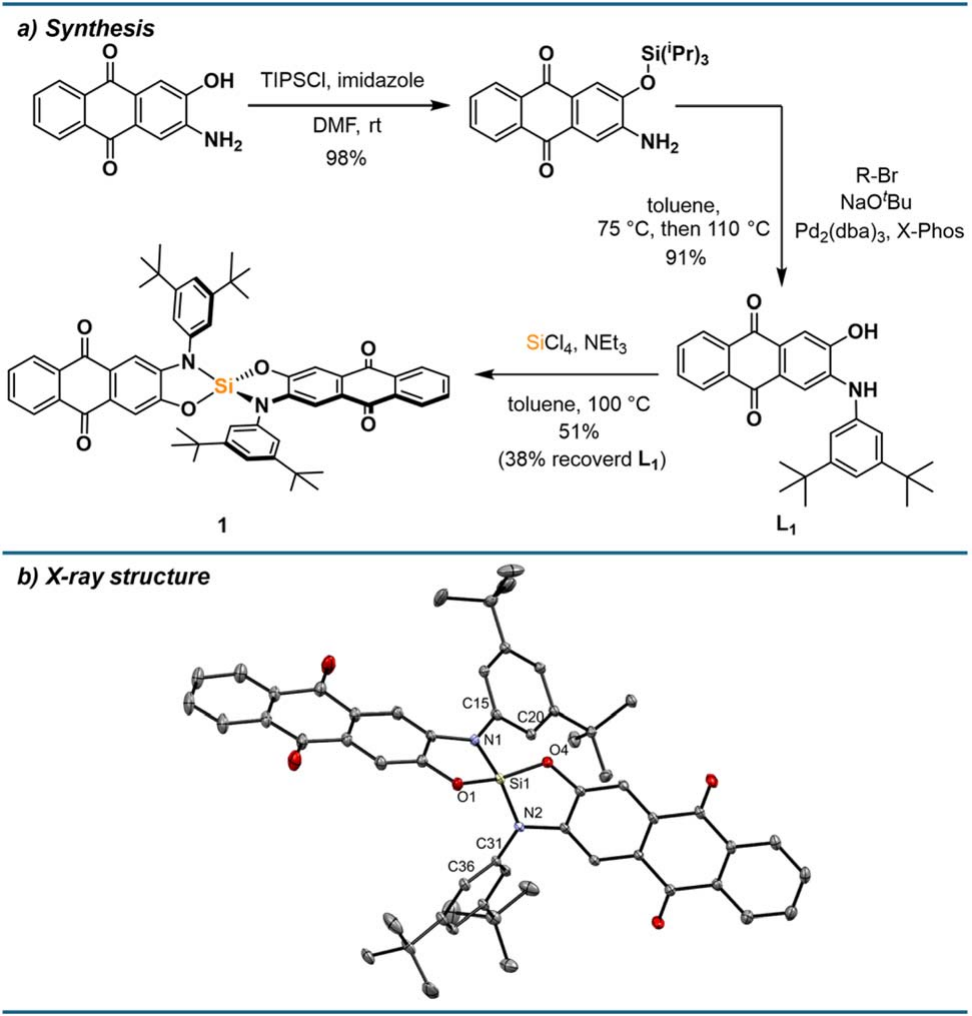
(a) Synthesis of aminophenol ligand L_1_ and bis(amidophenolato)silane 1. (b) Molecular structure of 1 determined by SCXRD analysis. Hydrogen atoms are omitted for clarity. Thermal displacement ellipsoids are displayed at the 50% probability level. Selected bond lengths [pm]: Si1–O1: 165.20(13), Si1–O4: 165.05(13), Si1–N1: 171.95(15), Si1–N2: 171.47(14). Selected bond angles [°]: O1–Si1–N1: 94.94(7), O1–Si1–O4: 119.78(7), N1–Si1–N2: 121.20(7). Selected dihedral angles [°]: Si1–N1–C15–C20: 32.7(2), Si1–N2–C31–C36: 64.0(2).

Building on our previously described bis(alizarinato)silane,^34^ which exhibited very limited solubility in organic solvents and could only be isolated as a Lewis base adduct, certain improvements were required for the design of a suitable chromogenic probe: the target Lewis acid should be (i) monomeric and donor-free, (ii) highly soluble in non-donor solvents, and (iii) possess a well-defined binding mode for Lewis bases. These features were met by using an amidophenolate ligand at silicon, which sterically prevents the oligomerisation generally associated with bis(catecholato)silanes,^35^ as well as disfavouring hexacoordinated bis-adducts that would complicate stoichiometries of the binding event.^36^ Starting from the commercially available 2-amino-3-hydroxy-anthraquinone, aminophenol L_1_ was accessible by silyl-protection of the hydroxy group, palladium catalysed amination with 1-bromo-3,5-di-*tert*-butylbenzene and *in situ* silyl deprotection (Fig. 2a). The ligand was isolated in excellent yield on a multigram scale. Complexation of silicon was achieved by reaction with silicon tetrachloride and triethylamine at 100 °C, followed by filtering off the precipitated hydrochloride salt and precipitation of the product from a cold dichloromethane solution. Although the isolated yield is only moderate (51%), the ligand L_1_ can be easily recovered by hydrolysis of the filtrates in 38% yield. The identity of 1 was confirmed by NMR spectroscopy, showing resonances of a single symmetric species in the ^1^H NMR spectrum, as well as a signal at −39.0 ppm in the ^29^Si, ^1^H HMBC spectrum, corresponding to a tetracoordinated silicon species.^35–37^1 is a bright yellow solid with excellent solubility in dichloromethane, toluene, and benzene. Yellow crystals suitable for SCXRD were obtained by vapor diffusion of *n*-pentane into a saturated DCM solution at room temperature. The solid-state structure shows the expected tetrahedrally coordinated silicon centre with orthogonal anthraquinone systems (Fig. 2b). The nitrogen aryl substituents are twisted with respect to the anthraquinone plane, with dihedral angles of 33° and 64°. Intermolecular interactions between the basic carbonyl functions and the Lewis acidic centre can be ruled out by the solid-state structure, and in solution, by absent concentration effects on UV-vis spectral features (ESI Section 2.2†).

### Assessment of Lewis acidity and optical properties

**Fig. 3:**
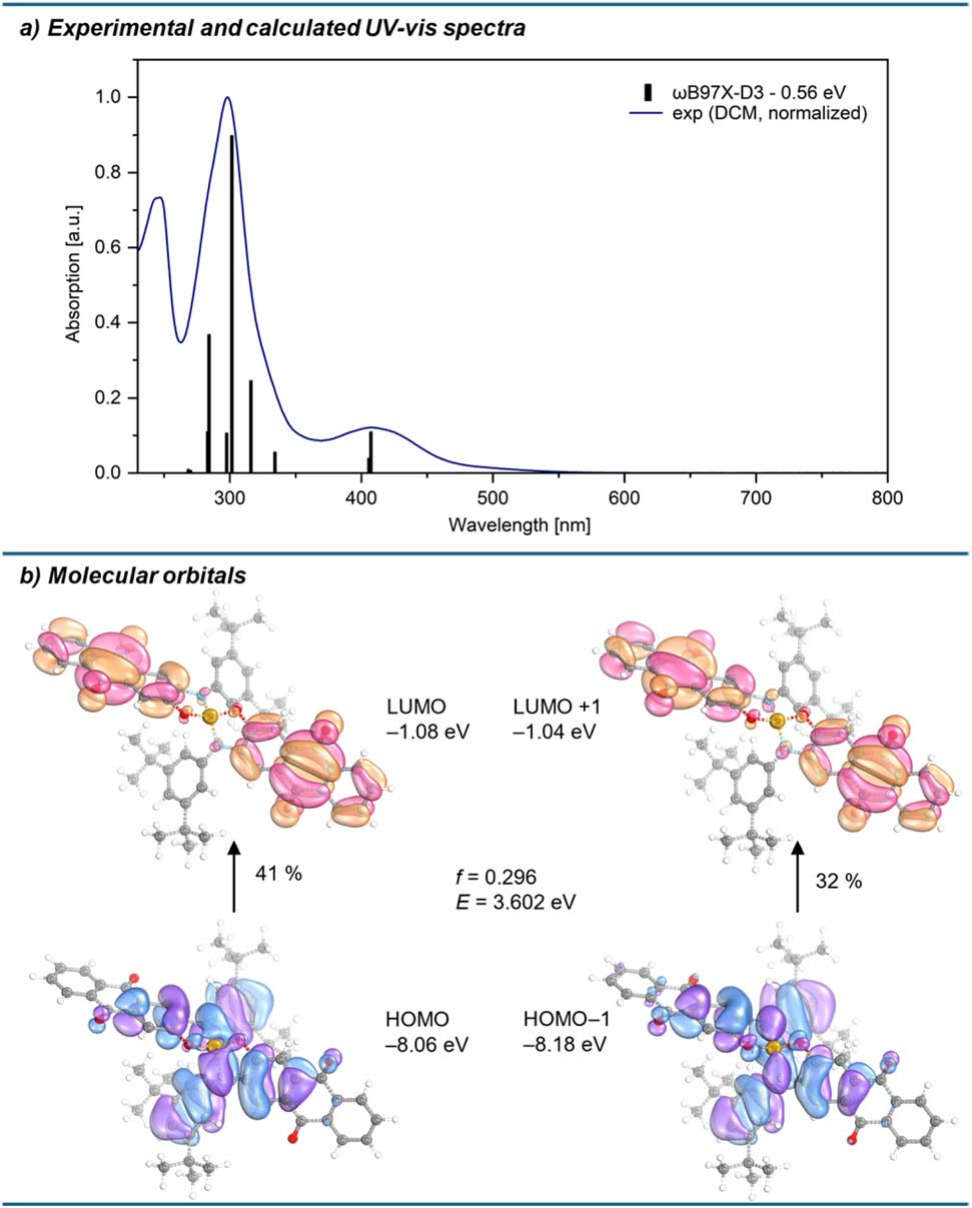
(a) Experimental UV-vis absorption spectrum of 1 (1.04 × 10^−4^ M in DCM, normalised, blue line) and calculated (ωB97X-D3/def2-TZVPP/SMD(DCM)//r^2^SCAN-3c, redshifted by 0.56 eV) transitions (black bars). (b) Frontier molecular orbitals involved in the low-energy CT excitation (*f* = oscillator strength; *E* = transition energy; transition energy given without 0.56 eV redshift).

**Fig. 4:**
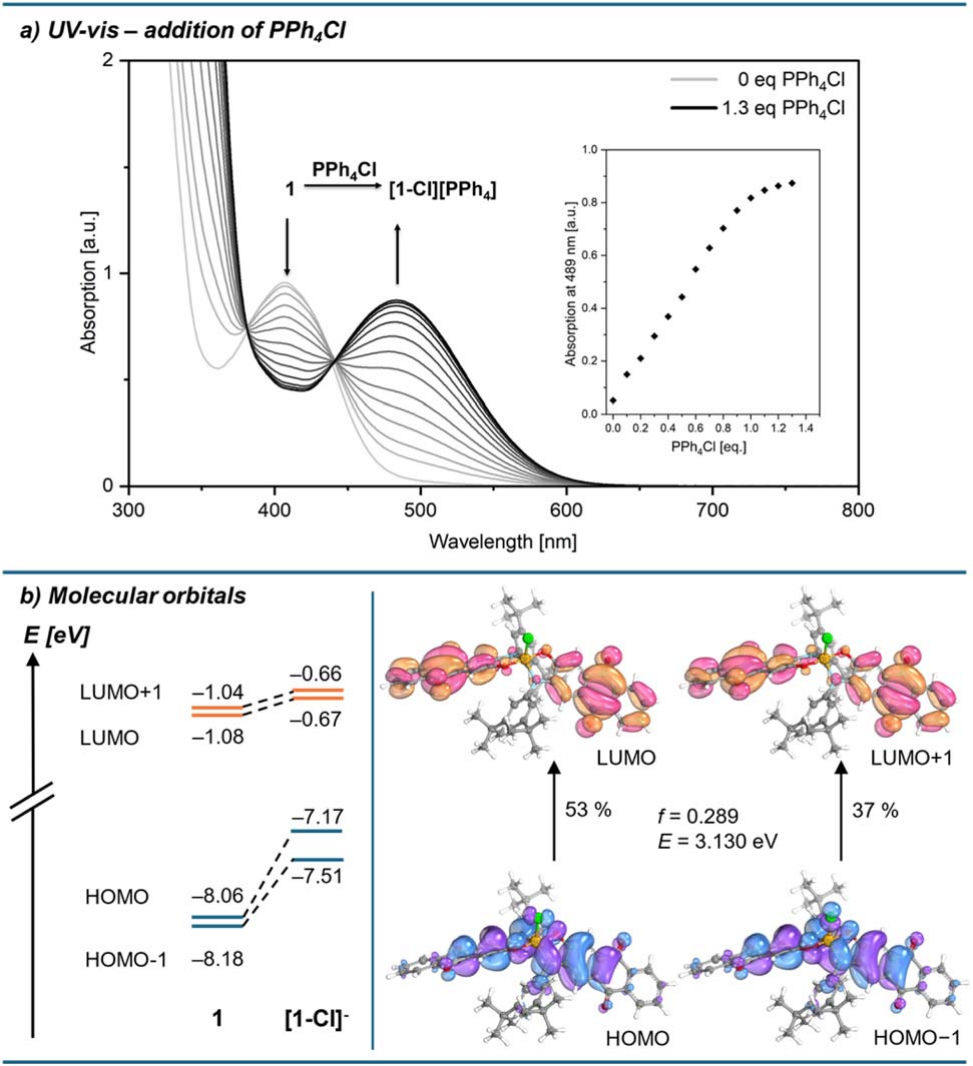
(a) Change in the experimental UV-vis absorption spectrum of 1 (5.84 × 10^−4^ M in DCM) upon stepwise addition of PPh_4_Cl (*K*_c_ ≈ 3.6 × 10^5^ mol^−1^ l). (b) Energy change in the frontier molecular orbitals upon chloride coordination in 1 and orbital transitions contributing to the low-energy CT excitation in the chloride adduct (*f* = oscillator strength; *E* = transition energy; transition energy given without 0.56 eV redshift; ωB97X-D3/def2-TZVPP/SMD(DCM)//r^2^SCAN-3c).

The global Lewis acidity of 1 was assessed by computation of the vacuum fluoride and hydride ion affinities at the DSD-BLYP(D3BJ)/def2-QZVPP//r^2^SCAN-3c level of theory, and solvent (DCM) corrected affinities with the COSMO-RS model. The ion affinities suggest Lewis acidity below the hard and soft superacid threshold, *e.g.*, lower values than the perfluorinated amidophenolatosilane Si(am^F^ph^F^)_2_ or the Lewis superacid Si(cat^Cl^)_2_, but still in the range of B(C_6_F_5_)_3_ in terms of FIA (Table 1). Effective Lewis acidity was gauged by the Gutmann–Beckett method, giving a ^31^P NMR shift of silicon bound triethyl phosphine oxide of 81.6 ppm, thus following a similar trend (Table 1). Optical properties were examined by UV-vis spectroscopy in DCM. 1 is characterised by a band with charge-transfer (CT) character at 407 nm, which is significantly blue-shifted compared to the absorption band of free aminophenol L_1_ (479 nm, measured in THF due to solubility issues in DCM, ESI Section 2.21†). TD-DFT calculations with the long-range corrected hybrid functional ωB97X-D3 ^40^ gave transitions in good agreement with the experimental absorption spectrum after applying a redshift of 0.56 eV (Fig. 3a). For 1, the relevant low-energy excitations with non-zero oscillator strength are comprised of HOMO → LUMO and HOMO−1 → LUMO+1 transitions as the largest contributors, with HOMO and HOMO−1 located on the electron-rich amidophenolate system and the nitrogen aryl substituents, while LUMO and LUMO+1 are located at the ligand backbone (Fig. 3b). The hypsochromically shifted absorption of 1 compared to the free L_1_ can be rationalised by the stabilisation of HOMO and HOMO−1 upon binding to silicon, whereas LUMO and LUMO+1 remain less affected. This HOMO and HOMO−1 stabilisation is caused by negative hyperconjugation of the oxygen and nitrogen lone pair type orbitals into the Si–O and Si–N σ* orbitals (see ESI† Section 3.4 for NBO analysis). Spiroconjugation across the tetrahedral silicon centre is less effective due to incorrect orbital symmetry in HOMO and HOMO−1.^41^ Of the occupied orbitals involved in the transition, only HOMO−4 has the suitable symmetry to enable spiroconjugation in its strict definition (ESI Section 3.5†). Importantly, both HOMO and HOMO−1 share significant localisation around the silicon centre, offering the chance to respond to electronic changes occurring from Lewis base coordination. Upon addition of a PPh_4_Cl solution, the absorption band at 407 nm vanished, and a new band at 489 nm appeared, along with a visible colour change from yellow to red, attributed to the formation of the corresponding chloridosilicate [1-Cl][PPh_4_] (Fig. 4a). UV-vis titration provided a binding constant of *K*_c_ ≈ 3.6 × 10^5^ mol^−1^ l (ESI Section 2.22†). TD-DFT calculations for [1-Cl]^–^ revealed low energy excitations consisting of HOMO → LUMO and HOMO−1 → LUMO+1 transitions, but with key differences in the frontier molecular orbitals compared to free 1. In [1-Cl]^–^, the LP(O/N) → σ*(SiO/SiN) negative hyperconjugation that is stabilising the HOMO in 1, is diminished by the geometry change from tetrahedral to trigonal bipyramidal (for NBO analysis, see ESI Section 3.4†). Additionally, antibonding interactions between the Si–Cl σ-bond orbital and the amidophenolate centred π-orbitals in HOMO−1, as well as between the chlorido p-orbital and the ligand π-orbitals in the HOMO, lead to a destabilisation of HOMO and HOMO−1 (Fig. 4b).

**Table 1:** Comparison between 1, Si(cat^Cl^)_2_, Si(am^F^ph^F^)_2_ and BCF of computed ion affinities (DSD-BLYP(D3BJ)/def2-QZVPP//r^2^SCAN-3c) and ^31^P NMR shifts of triethylphosphine oxide adducts. Solvent corrections (DCM) were calculated with the COSMO-RS solvent model and are given in parentheses

Compound	FIA vacuum (DCM)/kJ mol^−1^	HIA vacuum (DCM)/kJ mol^−1^	*δ* (^31^P NMR, Et_3_PO)/ppm
1	439 (220)	404 (317)	81.6
Si(am^F^ph^F^)_2_	497 (260)	460 (357)	83.3 (ref. 36)
Si(cat^Cl^)_2_	488 (270)	451 (368)	87.2 (ref. 38)
B(C_6_F_5_)_3_	438 (215)	476 (384)	77.0 (ref. 39)
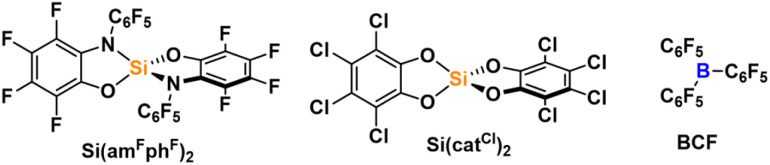			

### Formation of various donor adducts

**Fig. 5:**
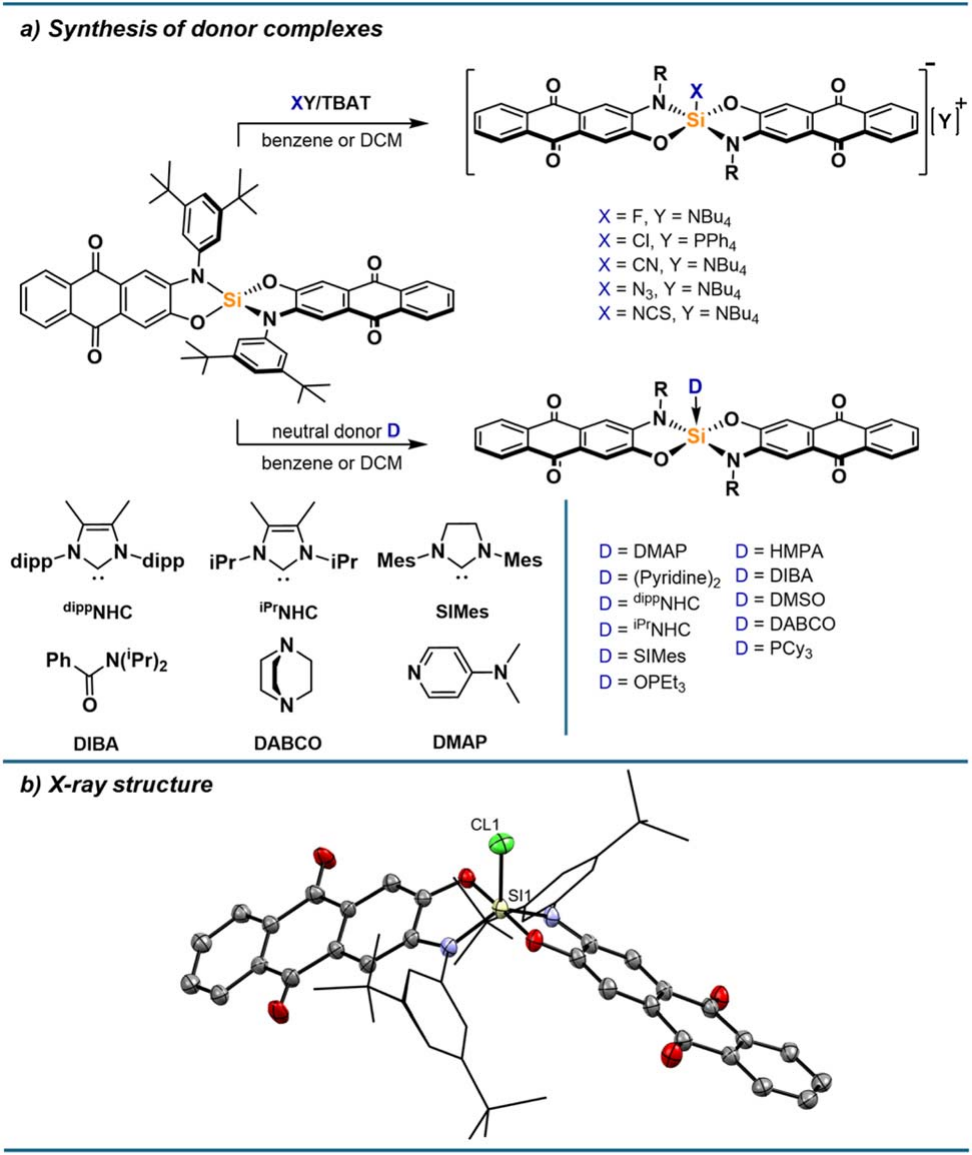
(a) Synthesis of pentacoordinate silicon complexes with neutral and anionic donors. (b) Molecular structure of [1-Cl][PPh_4_] determined by SCXRD analysis. Hydrogen atoms and the PPh_4_ cation are omitted for clarity. Thermal displacement ellipsoids are displayed at the 50% probability level. Selected bond lengths [pm]: Si1–Cl1: 211.64(10), Si1–O1: 174.78(18), Si1–N1: 178.1(2). Selected bond angles [°]: N1–Si1–N1: 128.16(10), O1–Si1–O4: 177.74(9), N1–Si1–Cl1: 116.45(8), O1–Si1–Cl1: 91.53(7).

The pronounced optical responses encouraged the screening of a structurally diverse set of donors. Thus, we turned our attention to the synthesis of a range of anionic and neutral donor complexes. Adding one equivalent of strong donors cleanly furnished the respective mono-adducts, which were all isolated and characterised. For anionic donors, salts with weakly coordinating cations were used to limit interactions between cations and the anionic complexes, especially *via* the Lewis basic carbonyl groups, which are cation binding sites for alizarin derivatives.^42^ While the fluoride and chloride adduct are readily accessible from tetrabutylammonium difluorotriphenylsilicate (TBAT) and tetraphenylphosphonium chloride, respectively, the bromide adduct significantly dissociates in solution (ESI Section 2.15†), and no binding of iodide is observed. Thiocyanate, cyanide and azide bind strongly to 1, allowing the isolation of their silicon complexes as respective tetrabutylammonium salts. For neutral donors, stable complexes with phosphine oxides, DMAP and N-heterocyclic carbenes are formed. Adducts with diisopropylbenzamide (DIBA), DMSO, pyridine, DABCO, and tricyclohexylphosphine (PCy_3_) could be isolated in the solid state but dissociate to some extent in solutions at low concentrations. When isolating the pyridine adduct by precipitation, the bis-adduct is obtained, likely in *trans* conformation based on calculated thermodynamics of different conformers (ESI Section 3.3†). However, the bis-adduct only forms in the solid-state, while the mono-adduct is the sole species present in solution based on NMR and UV-vis spectroscopy (ESI Section 1.21†). Weaker donors such as THF or acetonitrile were found to not form Lewis adducts to a significant degree, even at high donor concentrations in DCM. The donor complexes show ^29^Si NMR shifts typical for pentacoordinate silicon species, between −95 and −120 ppm. For the NCS adduct, a triplet with a ^29^Si–^14^N coupling constant of 27 Hz can be observed, as well as a shift of 88 ppm in the ^14^N NMR spectrum. In the ^1^H NMR spectra, the signals for protons closest to the silicon centre are broadened, which can be explained by the hindered rotation of the nitrogen substituents resulting in dynamics on the NMR timescale. As seen in the solid-state structure of [1-Cl][PPh_4_] (Fig. 5b) and of 1-PCy_3_ (ESI Section 4†), the pentacoordinate complexes adopt a trigonal bipyramidal geometry with the oxygen donors occupying the axial positions, in line with the higher electronegativity of oxygen and the steric bulk of the nitrogen substituents favouring the equatorial positions. SCXRD analysis of other donor complexes confirmed their structures, as well as the binding *via* nitrogen for the NCS adduct, while poor quality did not allow a detailed discussion of structural parameters (ESI Section 4†). The conformation of [1-Cl][PPh_4_] and 1-PCy_3_ in solid-state was also found as the minimum energy structure for all complexes optimised by DFT calculations at the r^2^SCAN-3c level of theory (ESI Section 3/6†). Corresponding Lewis pair formation enthalpies were obtained at the DSD-BLYP(D3BJ)/def2-TZVPP/SMD(DCM)//r^2^SCAN-3c level of theory. The calculated affinities are well reflected in the experimental findings, giving positive Δ*G* values for THF (+14.9 kJ mol^−1^), acetonitrile (+29.0 kJ mol^−1^), and iodide (+14.5 kJ mol^−1^), but negative values for the remaining Lewis bases. The association of a second Lewis base is less favoured than the first association in all cases (ESI Section 3.3† for further details).

**Fig. 6:**
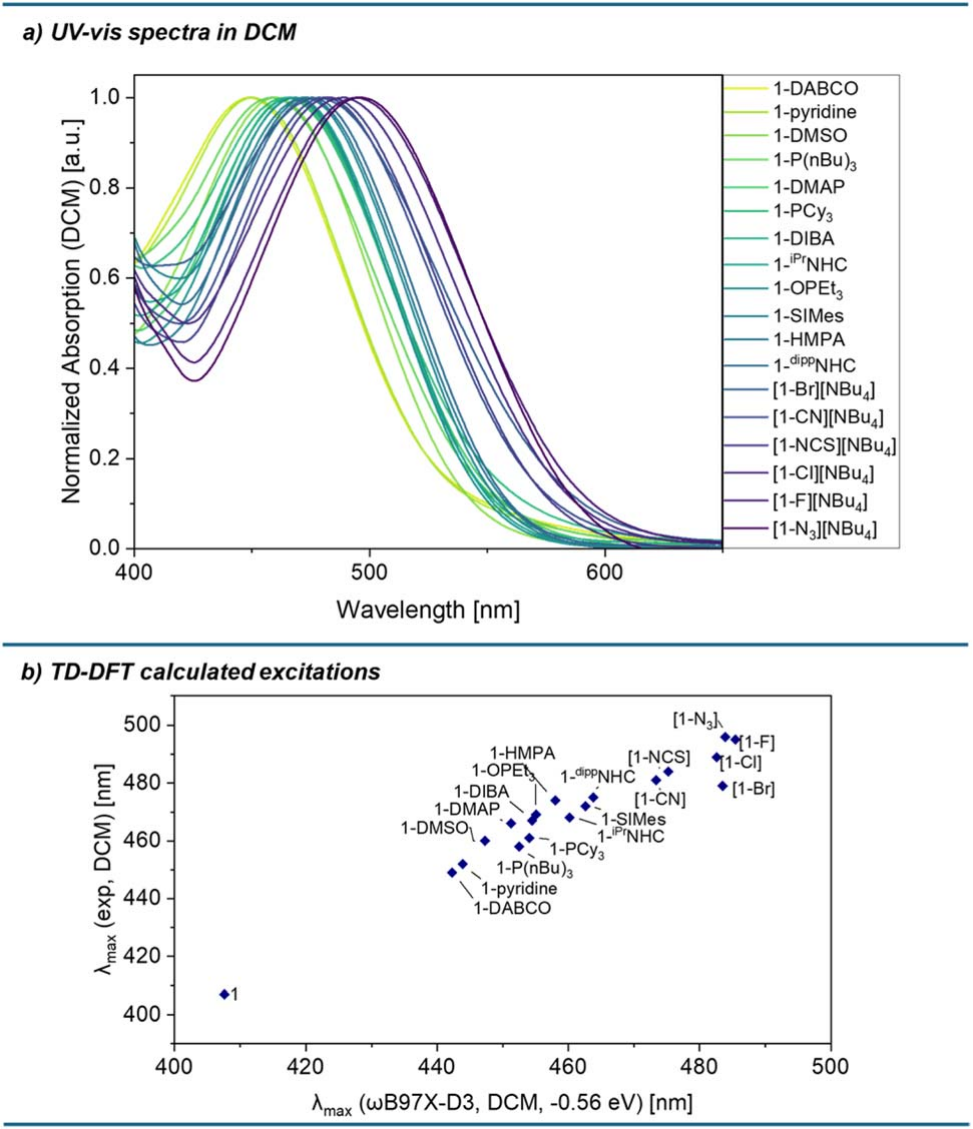
(a) Normalised CT absorption bands of donor adducts in DCM. (b) Calculated (ωB97X-D3/def2-TZVPP/SMD(DCM)//r^2^SCAN-3c) *vs.* experimentally observed absorption maxima for 1 and donor complexes 1-X/[1-X]^–^. Calculated absorption wavelengths were redshifted by 0.56 eV.

UV-vis absorption spectra of all compounds were measured in DCM (Fig. 6a). In the case of some donors (DIBA, DMSO, pyridine, PCy_3_, DABCO), excess of base was required to obtain spectra of the fully associated species (ESI Section 2†). In addition to the isolated complexes, UV-vis spectra of [1-Br][NBu_4_] and 1-P(*^n^*Bu)_3_ could be obtained with a large excess of the donor. Similarly to the chloride adduct, the CT absorption bands of all pentacoordinate silicon species are significantly red-shifted compared to donor-free 1, owing to the disruption of the negative hyperconjugation and destabilisation of the occupied frontier molecular orbitals. The relevant excitations were investigated by TD-DFT calculations on all species, which show reasonably good agreement with the experimental data (Fig. 6b).

### Comparing global and effective Lewis basicity

**Fig. 7:**
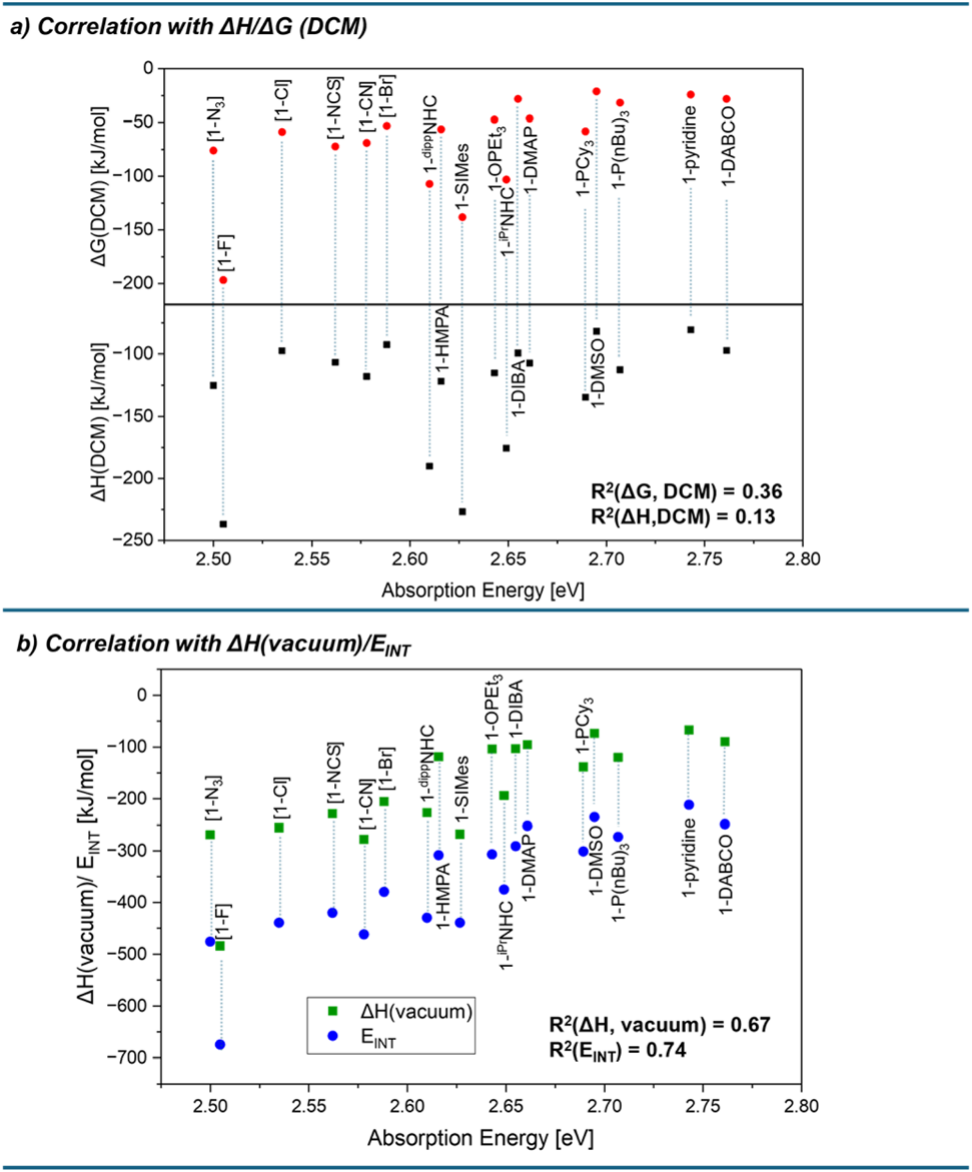
(a) Correlation between the absorption energy of donor adducts and the computed binding free energies (red circles) and enthalpies (black squares) in solution (DSD-BLYP(D3BJ)/def2-TZVPP/SMD(DCM)//r^2^SCAN-3c). (b) Correlation of the absorption energy of donor adducts with the gas phase enthalpies (green squares) and the interaction energy *E*_INT_ (*E* − *E*_DEF_) (blue circles), respectively (DSD-BLYP(D3BJ)/def2-TZVPP//r^2^SCAN-3c).

**Fig. 8:**
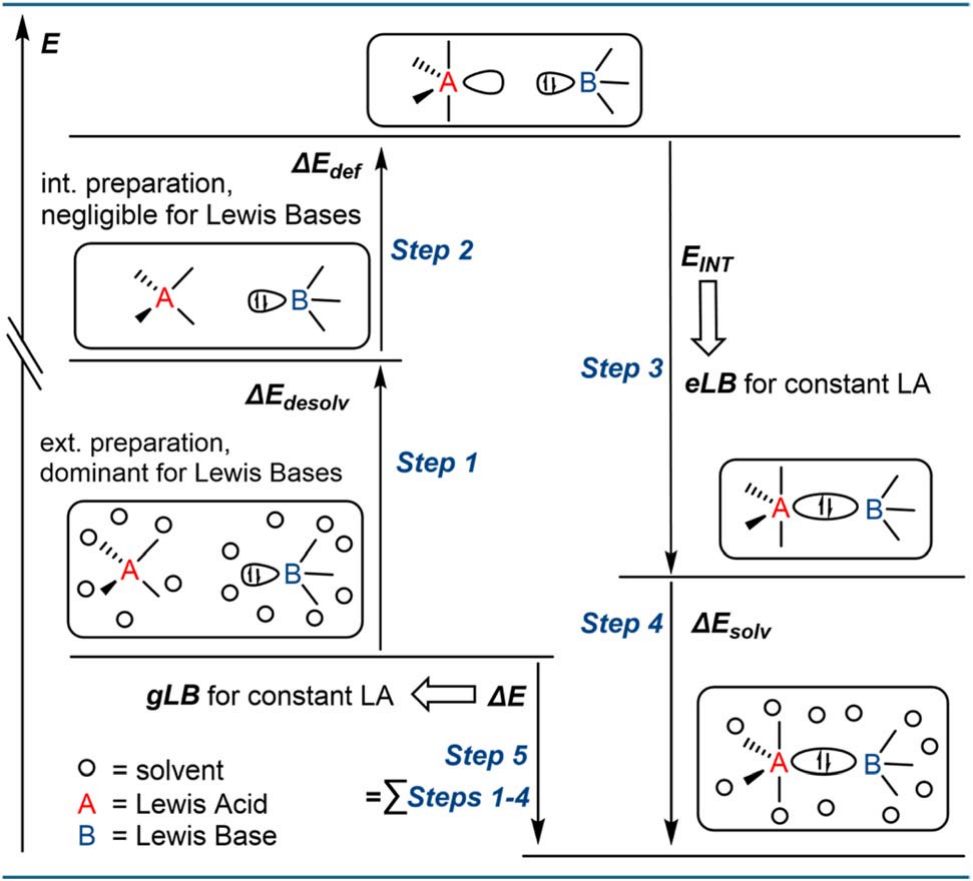
Proposed schematic Born–Haber cycle of Lewis acid–base interactions in solution. The binding enthalpy is differentiated from the interaction energy by desolvation and deformation of both acid and base components, as well as solvation of the formed acid–base adduct. For Lewis bases, the effect of desolvation is dominant, while deformation requires less preparation energy, in contrast to Lewis acids.

With a series of Lewis base-dependent spectroscopic parameters (*λ*_max_) in hand, we were interested in their correlation with the thermodynamics of Lewis pair formation. Any spectroscopic change at a Lewis acid by a Lewis base corresponds to effective Lewis basicity (eLB), while the Lewis pair formation (free) enthalpy with a fixed Lewis acid corresponds to global Lewis basicity (gLB). Comparing the absorption energies of the donor adducts (*E*_abs_, based on *λ*_max_ of the CT bands) with the computed solvation-corrected Δ*G*(DCM) values for adduct formation, only a weak correlation was found (red dots in Fig. 7a, *R*^2^ = 0.36). Plotting Δ*H*(DCM) *vs. E*_abs_ revealed an even poorer correlation (black squares in Fig. 7a, *R*^2^ = 0.13). Similar results were obtained with the COSMO-RS solvation model or by explicitly including cations for anionic Lewis bases (ESI Section 3.7†). Hence, a first important observation is that the spectroscopic responses are not reliable predictors for global Lewis basicity in solution. In other words, there is a clear distinction between global Lewis basicity and effective Lewis basicity, analogous to the difference between global and effective Lewis acidity.^13^ However, the correlation improved significantly when solvation energies were not considered. Thus, plotting *E*_abs_ against the vacuum enthalpies of Lewis base–acid pairing, the correlation improved to *R*^2^ = 0.67 (green squares in Fig. 7b), compared to the solvation-corrected data (*R*^2^ = 0.13). Notably, previous studies for neutral Lewis acids found solvation effects to be minor when comparing eLA and gLA.^13^ To rationalize this statistical improvement, we propose the following: The thermodynamics of adduct formation (gLB, Fig. 8, step 5) include substantial contributions from solvent reorganization (Fig. 8, steps 1/4). Since the Lewis acid is constant across the dataset, its desolvation energy (Δ*E*_Desolv_ of LA in step 1) is constant, and thus does not contribute to observed variance. Moreover, due to the relatively large size of 1 compared to the bases, the accessible surface areas of the Lewis adducts are also mostly invariant, and the influence of Δ*E*_Solv_ (step 4) for overall variance is minimal. This interpretation is supported by their numerical values: The mean deviation of Δ*E*_Solv_ across all Lewis adducts is 22 kJ mol^−1^, while that of Δ*E*_Desolv_ for all Lewis bases is 90 kJ mol^−1^ (ESI Table S3.37†). Hence, the observed variance between vacuum and solvation-corrected binding energies lies in Δ*E*_Desolv_ of the Lewis bases (step 1). In contrast, solvation energies have no direct influence for the spectroscopic responses (*E*_abs_). This is understandable from the fact that *E*_abs_ is a property of the final adduct only and does not report the energetic contributions along the adduct formation pathway. Specific dipole–dipole interactions of the Lewis pairs with the solvent were also evaluated but proved to be of minor relevance (ESI, Section 3.8†). Accordingly, the observed difference between gLB and eLB is a logical consequence of their respective energetic dependencies, explaining why eLB correlates better with gLB, if given as vacuum enthalpies.

We next considered the influence of deformation energy (Δ*E*_DEF_), which was previously identified as the key factor distinguishing gLA from eLA.^13^ To assess its impact on LB, we examined the interaction energy (*E*_INT_) between the deformed Lewis acid 1 and the deformed Lewis bases (step 3 in Fig. 8). *E*_INT_ is the binding energy corrected by the deformation energies Δ*E*_DEF_ of both the acid and the base required to adopt the adduct geometry (step 2, Fig. 8). Plotting vacuum *E*_INT_ against *E*_abs_ revealed a further improvement in correlation (*R*^2^ = 0.67 → *R*^2^ = 0.74, blue circles in Fig. 7b). This enhancement supports the view that deformation energy is also a relevant factor in explaining deviations between global and effective Lewis basicity. However, the structural changes experienced by the Lewis bases upon adduct formation are relatively minor. By consequence, Δ*E*_DEF_ is governed by contributions from the Lewis acid (see ESI, Section 3.3†). It means that eLB is distinguished from gLB by the deformation it induces in the Lewis acid. Nevertheless, based on the absolute values, Δ*E*_DEF_ plays a minor role compared to Δ*E*_Desolv_ of the Lewis base. Conceptually, Δ*E*_Desolv_ may be regarded as an “external” deformation energy arising from solvent reorganization, accompanying the “internal” deformation Δ*E*_DEF_ associated with the binding process. In summary, eLB is best described as a reflection of the intrinsic vacuum interaction energy (*E*_INT_), with deviations from gLB arising from both desolvation (external) and deformation (internal) effects.

### Experimental implications of the eLB *vs.* gLB distinction and transferability to other cases

An experimental manifestation of eLB is the extent to which binding of a donor to the Lewis acidic silicon centre is deactivating 1 from coordinating a second equivalent of the same base. This relation can be quantified by the difference in reaction enthalpy between the first and the second binding events. Notably, this value correlates well with eLB (as measured by *E*_abs_, *R*^2^ = 0.88), but only weakly with gLB (enthalpy of first binding event, *R*^2^ = 0.49, ESI Section 3.7†). In line with this, only for the base with weak eLB, pyridine, a bis-adduct was observed experimentally. It should be noted that steric effects may play a more significant role in the second coordination step, potentially contributing to deviations.

To evaluate the broader relevance of our findings beyond probe 1, we revisited prior studies that used a different effective probe. The ^19^F NMR chemical shift of *p*-fluorophenol has been shown to correlate with the thermodynamics of hydrogen bonding to Lewis bases in “near gas phase” solvent such as CCl_4_.^23^ In contrast, significant deviations in thermodynamic values were observed in more polar, hydrogen bond-donating solvents like dichloromethane, implying a breakdown in correlation with ^19^F NMR chemical shifts.^43^ Reinterpreting these results in light of our current findings provides an explanation: the induced ^19^F NMR chemical shifts of *p*-fluorophenol upon base binding are eLB parameters, whereas hydrogen bond formation enthalpies are gLB. The divergence between these two descriptors stems from desolvation: it is minimal in non-polar solvents like CCl_4_, but substantial in polar or H-bond-donating solvents. Supporting this interpretation, computational evaluation of hydrogen bond formation enthalpies shows a strong correlation between gas-phase and CCl_4_ values (*R*^2^ = 0.96), but a weaker correlation between gas-phase and DCM data (*R*^2^ = 0.61; see ESI Section 3.3† for details). This reinforces the utility of distinguishing between eLB and gLB not only for Lewis acid–base interactions but also in the context of hydrogen bonding. Additional comparisons of our *E*_abs_ scale with (heavily solvation dependant) p*K*_a_ values of the Lewis bases have also performed, further confirming the validity of our theory (see ESI, Section 3.9†).

### Evaluation of π-Lewis basicity

**Fig. 9:**
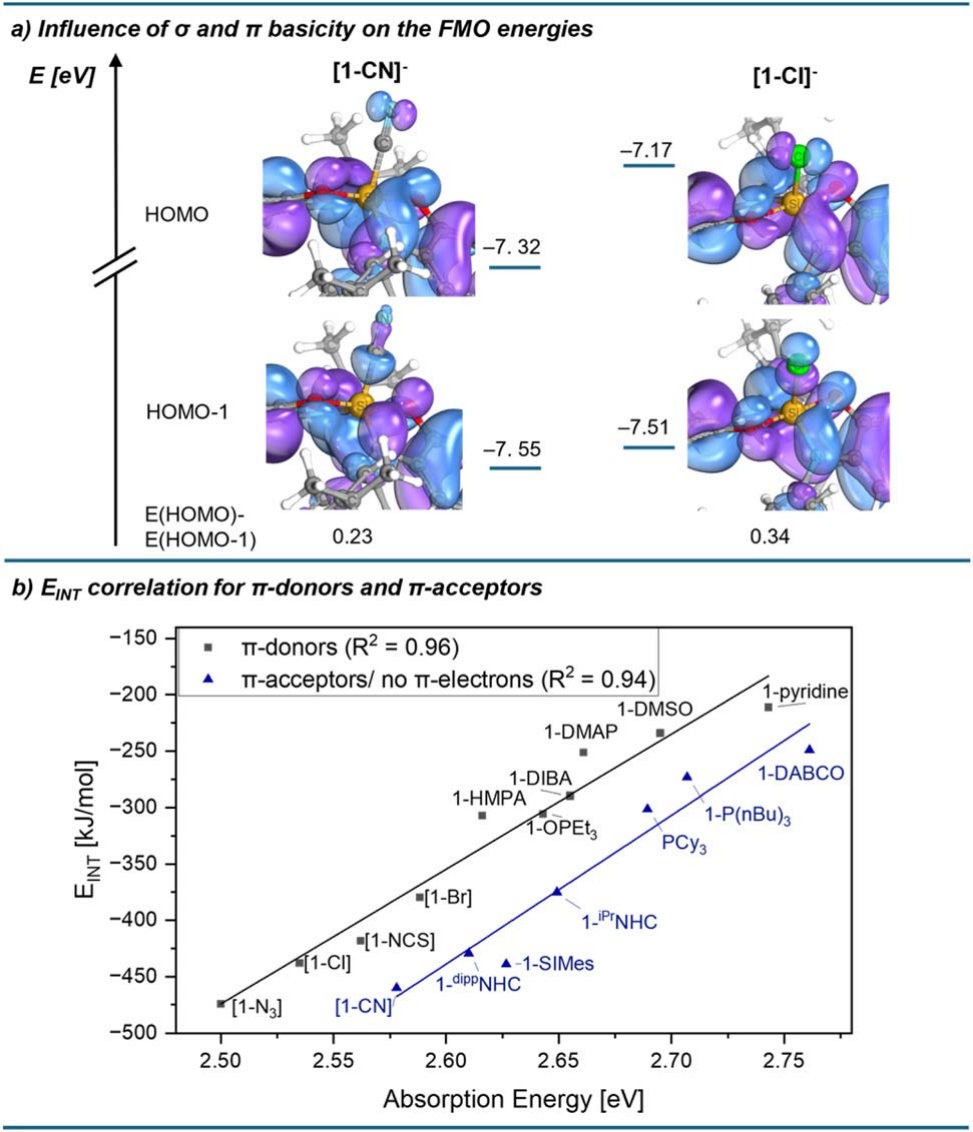
(a) Influence of π-basicity on the energy of occupied molecular orbitals ([1-CN]^–^*vs.*[1-Cl]^–^). (b) Correlation of the absorption energy of donor adducts (excluding outliers) with the interaction energy *E*_INT_ (*E* − *E*_DEF_, DSD-BLYP(D3BJ)/def2-TZVPP//r^2^SCAN-3c).

The remaining divergencies in the correlation plot between *E*_INT_ and *E*_abs_ (Fig. 7b) motivated for further interpretations concerning the electronic nature of the Lewis bases. Fluoride was found as a major outlier, for which we suggest the following: Due to the high specific affinity of silicon to fluoride, this anion represents the only Lewis base that can compete with the Si–O bonds of the chromogenic ligand. Indeed, the computed Si–O bond lengths are the longest for the fluoride adduct out of all species (ESI Table S3.12†), which in turn leads to the breakdown of the correlation for this base. Excluding this outlier, the bases can be grouped into donors with energetically available π-electrons (halides, thiocyanate, azide, and oxygen donors) on the one hand, and Lewis bases without occupied π-orbitals on the other. This second group contains π-acceptors (N-heterocyclic carbenes, phosphines, and cyanide),^44–47^ as well as DABCO, which has no π-electrons at the donor site. Occupied π-orbitals at the donor (π-basicity) contribute to a red shift in the absorption spectrum by destabilising the HOMO (Fig. 4b). In the case of Lewis bases without π-electrons (or π-acceptors), this destabilising effect should be absent, resulting in a less pronounced red shift. Indeed, the computed HOMO − HOMO−1 energy gaps for complexes with π-acceptors are smaller than for all other Lewis base adducts (Fig. 9a for chloride *vs.* cyanide, and ESI Section 3.5†). Grouping donors accordingly, a nearly ideal correlation (*R*^2^ = 0.96) is observed when comparing *E*_INT_ with *E*_abs_ for the set of π-donor Lewis bases (Fig. 9b). Similarly, an almost perfect correlation was found within the set of non-π-donor bases (*R*^2^ = 0.94). Since both σ- and π-effects are combined into a single experimental observable (*λ*_max_), deconvolutions would be required for a parametrisation of both contributions. A comparison between the unsaturated ^dipp^NHC and the saturated SIMes may offer insights into this interplay of σ-donation and π-acceptance. Saturated NHCs have been shown to be both stronger σ-donors and π-acceptors than their unsaturated analogues.^45,48,49^ In line with this, SIMes binds more strongly to 1, as reflected in both Δ*H* and *E*_INT_ values. However, 1-SIMes exhibits a higher-energy absorption than 1-^dipp^NHC, along with a smaller HOMO − HOMO−1 gap (ESI Section 3.5†), which is the result of stronger π-acceptance. While these observations are based on a limited dataset and should not be overinterpreted, compound 1 is in principle sensible to distinguish σ-basicity from π-basicity. These distinctions are reminiscent of the angular overlap model in transition metal chemistry,^50–58^ and recently gaining interest for halogen bond donors and its offsprings.^59–65^

## Conclusions

In this study, we investigated whether the established distinction between global and effective interactions for Lewis acidity also applies to Lewis basicity, leading to the definitions of global Lewis basicity (gLB) and effective Lewis basicity (eLB). Using the chromogenic Lewis acid 1 as a novel eLB probe, we examined a series of structurally and electronically diverse Lewis bases. Our findings confirm that gLB and eLB are fundamentally distinct descriptors of Lewis basicity. Importantly, the factors underlying this distinction differ from those known for Lewis acidity. While the divergence between global (gLA) and effective Lewis acidity (eLA) is primarily governed by the deformation energy (*E*_DEF_) of the Lewis acid, this factor is relatively minor for Lewis bases. Instead, the desolvation energy of the Lewis base emerges as the dominant factor separating the thermodynamics of adduct formation (gLB) from the spectroscopic response (eLB). Within this novel theory of Lewis pair interaction, desolvation can be conceptualized as “external deformation” that influences the global thermodynamics but not the spectroscopic response of the adduct, adding a new layer to the growing interest in solvation-dependent behaviour in Lewis acid–base chemistry.^66–70^ In addition, our study highlights the significance of distinguishing between σ- and π-basicity, also in p-block Lewis pair systems. Addressing such aspects could open new avenues with practical implications, such as tailoring solvent environments in Lewis base catalysis or designing p-block Lewis components that leverage π-interactions to enhance binding strength or enable selective reactivity *via* additional interaction pathways.

## Author contributions

L. G. and L. S. devised the project and designed the experiments. L. S. performed the experimental work, quantum chemical calculations and data analysis. L. G. and L. S. wrote the manuscript.

## Conflicts of interest

There are no conflicts to declare.

## Supplementary Material

SC-OLF-D5SC03200H-s001

SC-OLF-D5SC03200H-s002

## Data Availability

Computational and experimental details, Cartesian coordinates of the computed structures and characterisation data of the described compounds are available in the ESI.† Crystallographic data have been deposited at the Cambridge Crystallographic Data Centre (CCDC: 2403101, 2403102 and 2466565†).
