# Supplementary material for: What distinguishes the strength and the effect of a Lewis base: insights with a strong chromogenic silicon Lewis acid
Source: Chem Sci. 2025 Jul 23;16(34):15387–95. doi: 10.1039/d5sc03200h (PMC12308751; doi:10.1039/d5sc03200h)
Supplement: SC-016-D5SC03200H-s001 [file SC-016-D5SC03200H-s001.pdf]

Supplementary Information (SI) for Chemical Science.
This journal is © The Royal Society of Chemistry 2025

Supporting Information

What Distinguishes the Strength and the Effect of a Lewis Base: Insights with a Strong Chromogenic Silicon Lewis Acid

Lennart Stoess,[a] and Lutz Greb*[a]

a Lennart Stoess, Prof. Dr. Lutz Greb
Anorganisch-Chemisches Institut,
Ruprechts-Karls Universität Heidelberg
Im Neuenheimer Feld 270, 69120 Heidelberg (Germany)
E-mail: greb@uni-heidelberg.de

Table of Contents

1 Synthesis and Characterisation Data 1
1.1 General Considerations 1
1.2 2-Amino-3-(triisopropylsilyloxy)anthraquinone 2
1.3 2-((3,5-Di-*tert*-butylphenyl)amino)-3-hydroxyanthraquinone (L_1) 4
1.4 1 6
1.5 [1-F][NBu_4] 9
1.6 [1-Cl][PPh_4] 12
1.7 [1-CN][NBu_4] 15
1.8 [1-NCS][NBu_4] 17
1.9 [1-N_3][NBu_4] 20
1.10 1-(pyridine)$_2$ 23
1.11 1-DMAP 25
1.12 1-^{dipp}NHC 28
1.13 1-DMSO 30
1.14 1-$OPEt_3$ 32
1.15 1-DIBA 35
1.16 1-HMPA 37
1.17 1-DABCO 40
1.18 1-^{iPr}NHC 42
1.19 1-PCy_3 44
1.20 1-SIMes 47
1.21 Coordination State of 1-pyridine in Solution 49
2 UV-vis Spectroscopy 50
2.1 CT Absorption Bands of all Compounds 50
2.2 1 51
2.3 [1-CN][NBu_4] 52
2.4 [1-Cl][PPh_4] 53
2.5 [1-NCS][NBu_4] 54
2.6 [1-N_3][NBu_4] 55
2.7 [1-F][NBu_4] 56
2.8 1-HMPA 57
2.9 1-(pyridine)$_2$ 58
2.10 1-DMAP 59
2.11 1-$OPEt_3$ 60
2.12 1-DMSO 61
2.13 1-DIBA 62

2.14 1-^{dipp}NHC 63
2.15 [1-Br][NBu_4] 64
2.16 1-P(*n*Bu)$_3$ 65
2.17 1-DABCO 66
2.18 1-^{iPr}NHC 67
2.19 1-PCy_3 68
2.20 1-SIMes 69
2.21 2-((3,5-di-*tert*-butylphenyl)amino)-3-hydroxyanthraquinone (L_1) 70
2.22 UV-vis titration of 1 with PPh_4Cl 71
3 Computational Section 72
3.1 General Information 72
3.2 Fluoride and Hydride Ion Affinities 73
3.3 Thermodynamics 74
3.4 NBO Analysis of Negative Hyperconjugation in 1 and [1-Cl] 84
3.5 TD-DFT/TDA Calculated Excitations 86
3.6 Correlation between TD-DFT/TDA excitations and experimental UV-vis spectra 106
3.7 Correlations between UV-vis Data and Calculated Thermodynamics 107
3.8 Impact of Dipole Moments on Solvation Effects in Adducts 1-X 110
3.9 Comparison of Absorption Energy and pK_A Values 111
4 X-Ray Crystallography 112
5 IR Spectra of Isolated Compounds 117
6 Coordinates 125
7 References 160

1 Synthesis and Characterisation Data

1.1 General Considerations

All manipulations were carried out under air- and moisture-free conditions using standard Schlenk techniques under argon or nitrogen atmosphere. Sensitive compounds were stored in a nitrogen-filled *Sylatech* glovebox at –40 °C. All solvents were dried by standard procedures, stored over 3 Å or 4 Å molecular sieves and freshly degassed in three freeze-pump-thaw cycles prior to use. Commercially available starting materials were used as received. Identity and purity of newly synthesised compounds were confirmed by multinuclear NMR spectroscopy, as well as IR spectroscopy, mass spectrometry, and SCXRD if possible. ^{1}H, ^{13}C, ^{19}F, ^{31}P and ^{29}Si NMR spectra were collected either by the NMR department of the Institute of Inorganic Chemistry of the University of Heidelberg on either a *Bruker Avance II 400* or *Bruker Avance III 600* spectrometer, or in house on a *Bruker Ascend 400* spectrometer at 298K. ^{13}C and ^{29}Si spectra were recorded with broadband ^{1}H decoupling. NMR shifts are given as δ in ppm and referenced against solvent residual signals for ^{1}H and ^{13}C spectra. Coupling constants J are given in Hz. NMR spectra were analysed and plotted using *MestReNova 14.3*. Electronspray ionisation mass spectra were recorded on a *Bruker ApexQe hybrid 9.4 T FT-ICR* spectrometer and LIFDI mass spectra were recorded on a *JEOL AccuTOF GCx* spectrometer by the mass spectrometry department of the Institute of Organic Chemistry at the University of Heidelberg. ATR-IR spectra were measured on an *Agilent Cary 630* spectrometer inside a nitrogen-filled glovebox. UV-vis spectra were measured on a JASCO V-570 UV/VIS/NIR spectrophotometer in a 2 mm quartz cuvette equipped with a J-Young valve.

1.2 2-Amino-3-(triisopropylsilyloxy)anthraquinone

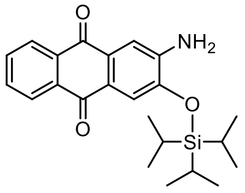

2-Amino-3-hydroxyanthraquinone (3.00 g, 12.5 mmol, 1.00 eq), imidazole (1.28 g, 18.8 mmol, 1.50 eq) and triisopropylsilyl chloride (3.20 ml, 15.1 mmol, 1.20 eq) were stirred in DMF (20 ml) at room temperature for 1 h. Water (100 ml) was added, and the resulting suspension filtered. The solid was washed with water (4x50 ml), dried *in vacuo*, washed with *n*-pentane (3x20 ml) and dried again *in vacuo*. The product was isolated as a red powder (4.53 g, 12.3 mmol, 98%).

^{1}H NMR (600 MHz, $CDCl_3$) δ 8.26 – 8.18 (m, 2H), 7.74 – 7.68 (m, 2H), 7.61 (s, 1H), 7.50 (s, 1H), 4.54 (s, 2H), 1.43 (sept, *J* = 7.5 Hz, 3H), 1.16 (d, *J* = 7.5 Hz, 18H).

^{13}C NMR (151 MHz, $CDCl_3$) δ 183.2, 182.2, 147.5, 144.2, 134.2, 133.8, 133.7, 133.4, 129.4, 126.9, 126.9, 125.7, 115.2, 111.6, 18.1, 12.9.

^{29}Si NMR (119 MHz, $CDCl_3$) δ 20.3.

HRMS (EI): $[C_{23}H_{29}NO_3Si]^+$, calcd.: 395.1911, found: 395.1895.

IR (ATR) [cm^{-1}] $\tilde{v}$ = 3481 (m, $\tilde{v}_{NH2}$), 3356 (m, $\tilde{v}_{NH2}$), 2947 (m, $\tilde{v}_{CH}$), 2867 (m, $\tilde{v}_{CH}$), 1665 (m, $\tilde{v}_{C=O}$), 1646 (m, $\tilde{v}_{C=O}$).

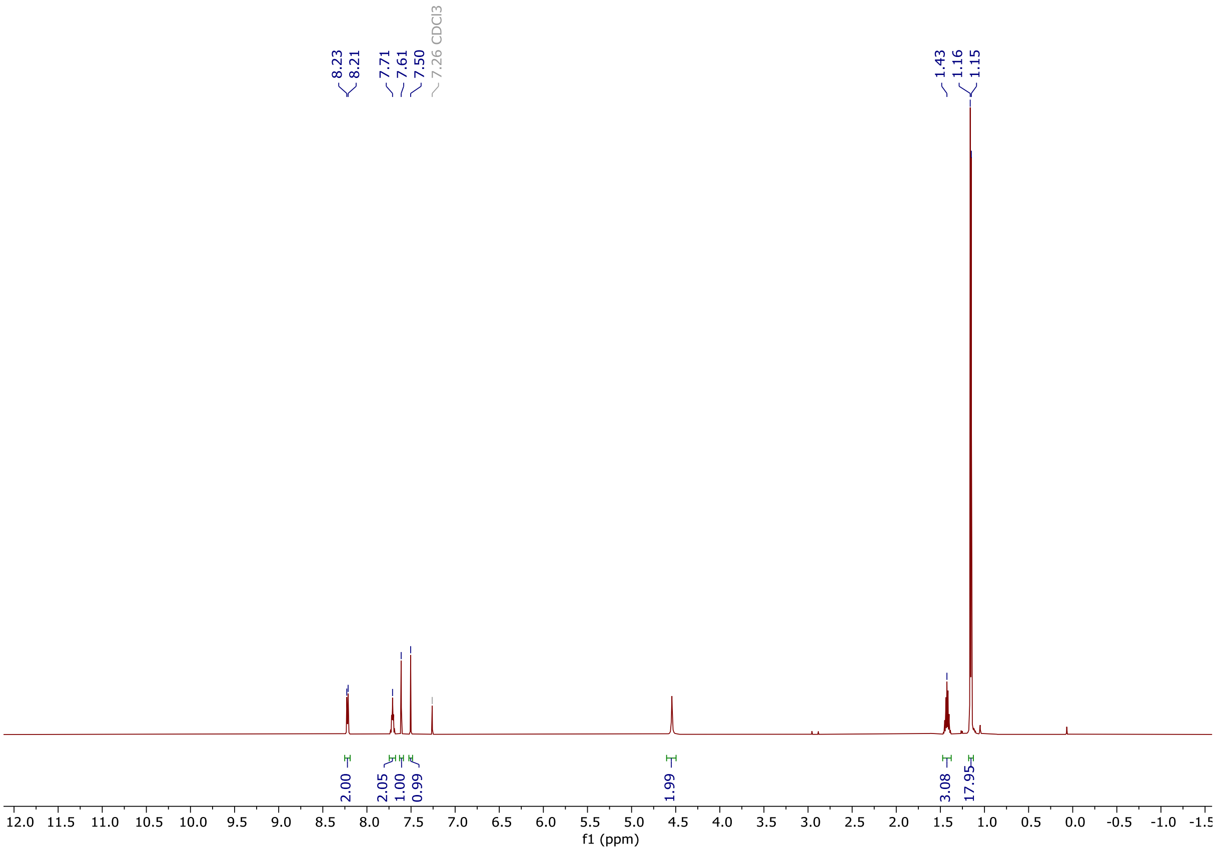

Figure S1.1. ^{1}H NMR spectrum (600 MHz, $CDCl_3$) of 2-amino-3-(triisopropylsilyloxy)anthraquinone.

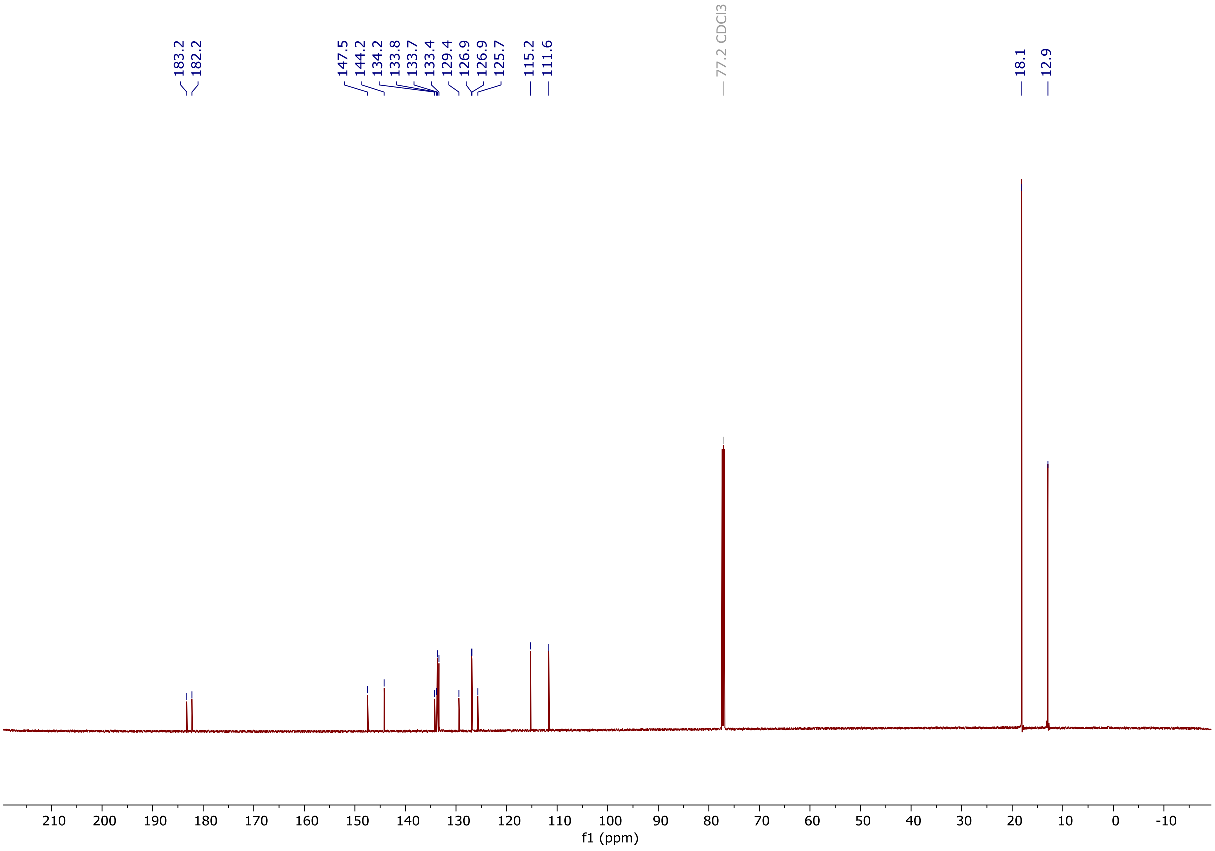

Figure S1.2. ^{13}C NMR spectrum (151 MHz, $CDCl_3$) of 2-amino-3-(triisopropylsilyloxy)anthraquinone.

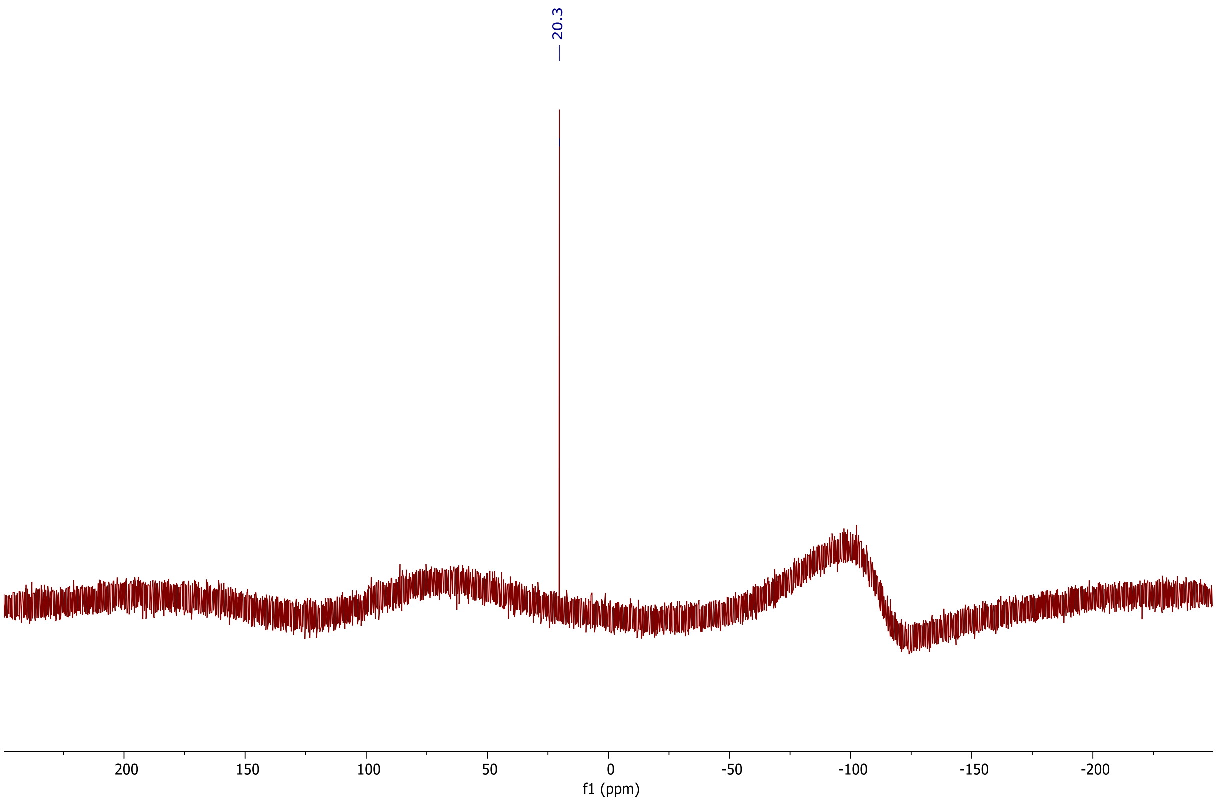

Figure S1.3. ^{29}Si NMR spectrum (119 MHz, $CDCl_3$) of 2-amino-3-(triisopropylsilyloxy)anthraquinone.

1.3 2-((3,5-Di-*tert*-butylphenyl)amino)-3-hydroxyanthraquinone (L_1)

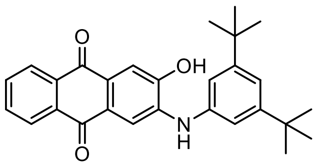

2-Amino-3-(triisopropylsilyloxy)anthraquinone (5.78 g, 14.6 mmol, 1.00 eq), 1-bromo-3,5-di-*tert*-butylbenzene (4.13 g, 15.3 mmol, 1.05 eq), NaOtBu (3.51 g, 36.5 mmol, 2.50 eq), $Pd_2(dba)_3$ (268 mg, 0.29 mmol, 0.02 eq) and XPhos (348 mg, 0.35 mmol, 0.05 eq) were suspended in toluene (100 ml) and heated to 75 °C for 12 h. Full conversion of the starting material was ensured by TLC monitoring. The mixture was then heated to 110 °C for 16h to complete the silyl ether deprotection. The mixture was added to a separatory funnel containing ethyl acetate (200 ml). The organic phase was washed with 2M NaOH (3x200 ml), then with sat. NH_4Cl (200 ml), resulting in the precipitation of a red solid. THF was added until the solid dissolved (about 300 ml) and the phases were separated. The organic phase was washed with sat. NaCl (200 ml), dried over $MgSO_4$ and filtered. The solvent was removed *in vacuo*, the resulting solid suspended in *n*-hexane and filtered. The solid was washed with acetone (2x10 ml) and DCM (5x30 ml), then dried *in vacuo* overnight. The product was isolated as a red solid (5.67 g, 13.3 mmol, 91%).

^{1}H NMR (600 MHz, DMSO-d_6) δ 11.28 (s, 1H), 8.12 (s, 1H), 8.11 – 8.07 (m, 2H), 7.85 (s, 1H), 7.83 – 7.77 (m, 2H), 7.55 (s, 1H), 7.21 (s, 2H), 7.11 (s, 1H), 1.30 (s, 18H).

^{13}C NMR (151 MHz, DMSO-d_6) δ 182.1, 181.0, 151.2, 150.3, 139.7, 139.3, 133.9, 133.6, 133.6, 133.1, 127.1, 126.4, 126.2, 124.9, 116.6, 115.9, 110.9, 108.0, 34.7, 31.3.

HRMS (EI): $[C_{28}H_{29}NO_3]^+$, calcd.: 427.2142, found: 427.2113.

UV-vis (THF): λ_{max} (ε) = 479 nm (7200 $M^{-1}cm^{-1}$).

IR (ATR) [cm^{-1}] $\tilde{\nu}$ = 3413 (w, $\tilde{\nu}_{NH/OH}$), 3329 (m, $\tilde{\nu}_{NH/OH}$), 2954 (m, $\tilde{\nu}_{CH}$), 2863 (w, $\tilde{\nu}_{CH}$), 1658 (s, $\tilde{\nu}_{C=O}$), 1647 (m, $\tilde{\nu}_{C=O}$).

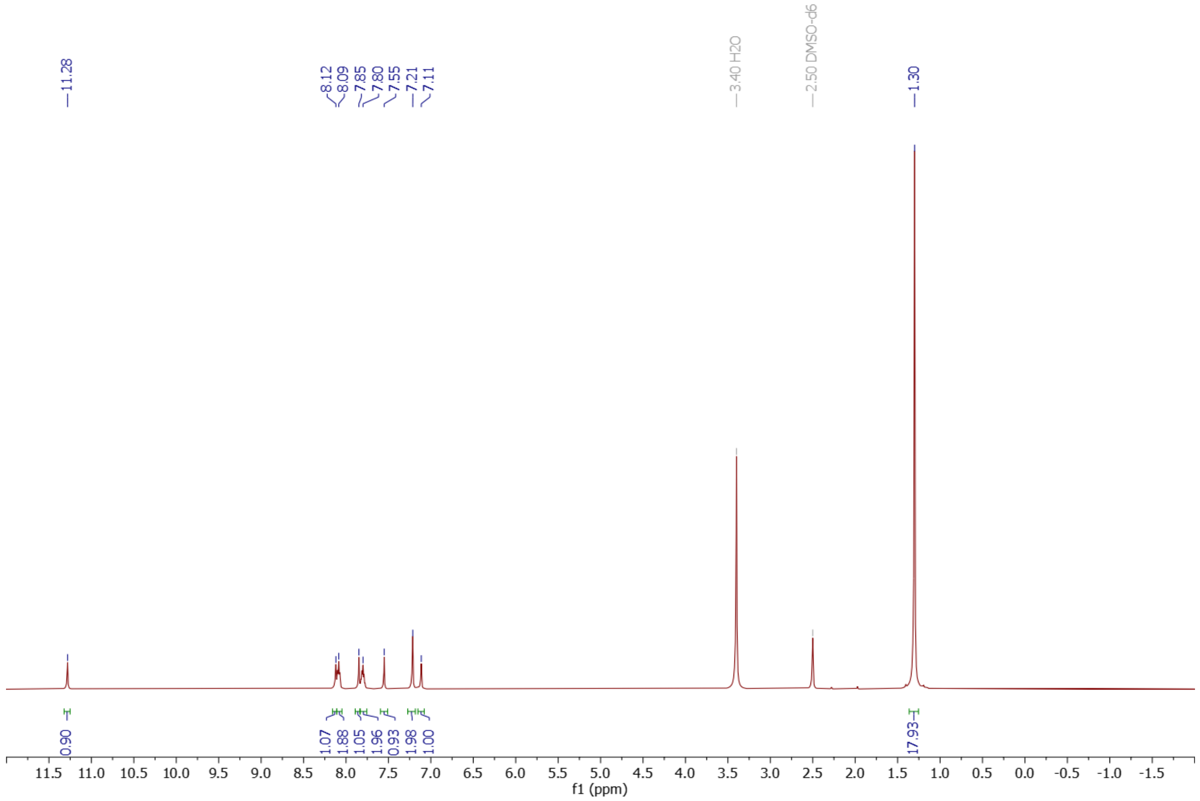

Figure S1.4. ^{1}H NMR spectrum (600 MHz, DMSO-d_6) of 2-((3,5-di-*tert*-butylphenyl)amino)-3-hydroxyanthraquinone.

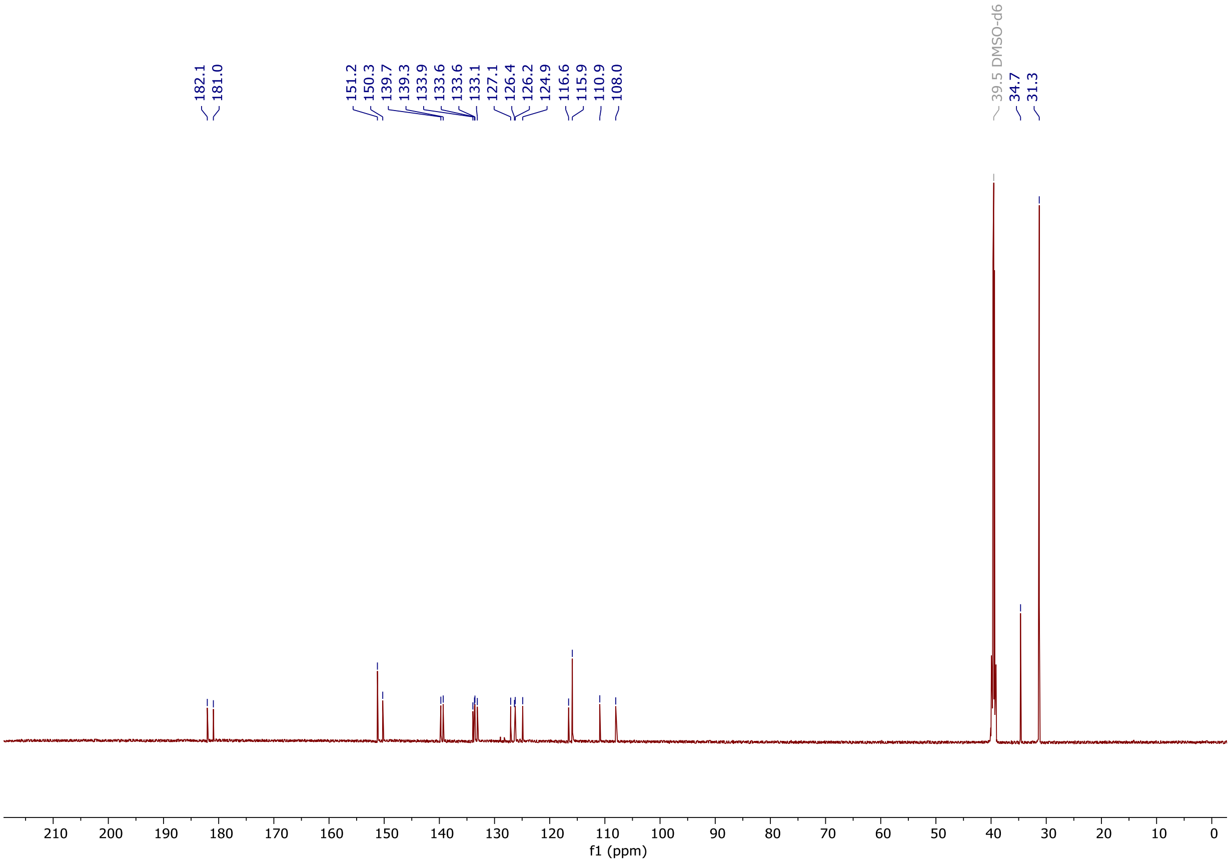

Figure S1.5. ^{13}C NMR spectrum (151 MHz, DMSO-d_6) of 2-((3,5-di-*tert*-butylphenyl)amino)-3-hydroxyanthraquinone.

1.4 1

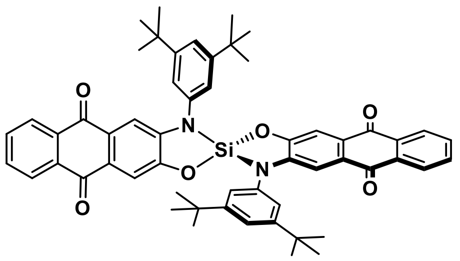

2-((3,5-Di-*tert*-butylphenyl)amino)-3-hydroxyanthraquinone (5.22 g, 12.2 mmol, 2.00 eq), triethylamine (3.40 ml, 42.4 mmol, 4.00 eq) and silicon tetrachloride (0.77 ml, 6.7 mmol, 1.10 eq) were heated in toluene (100 ml) to 100 °C for 16 h. After cooling to room temperature, the mixture was filtered, and the filtrate concentrated *in vacuo*. The resulting dark yellow solid was suspended in *n*-hexane, filtered, and washed with *n*-hexane (3x10 ml). The solid was redissolved in DCM (approx. 100 ml) and the volume reduced to one third, accompanied by cooling. The product precipitated as a yellow microcrystalline solid, which was filtered off, washed with DCM (3x5 ml), and dried *in vacuo* overnight (2.74 g, 3.12 mmol, 51%). Single crystals suitable for SCXRD were grown by gaseous diffusion of *n*-pentane into a saturated DCM solution at room temperature.

The combined filtrates from the previous steps were stirred under ambient air for 2 days, followed by removal of the solvent and washing with DCM to recover the aminophenol ligand (1.98 g, 4.63 mmol, 38%), giving a yield of 89% based on recovered starting material.

^{1}H NMR (600 MHz, $CDCl_3$) δ 8.31 – 8.27 (m, 2H), 8.25 – 8.21 (m, 2H), 8.00 (s, 2H), 7.85 (s, 2H), 7.79 – 7.71 (m, 4H), 7.31 (s, 2H), 7.04 (s, 4H), 1.22 (s, 36H).

^{13}C NMR (151 MHz, $CDCl_3$) δ 182.7, 182.2, 153.4, 149.3, 143.5, 136.1, 134.0, 133.8, 133.8, 133.6, 130.4, 128.4, 127.2, 127.2, 121.2, 118.4, 112.0, 109.0, 35.1, 31.5.

^{29}Si NMR (^{1}H-^{29}Si-HMBC) (119 MHz, $CDCl_3$) δ –39.0.

MS (LIFDI+): $[C_{56}H_{54}N_2O_6Si]^+$, calcd.: 878.38, found: 878.45.

EA Anal. Calcd. For $C_{56}H_{54}N_2O_6Si$: C, 76.51; H, 6.19; N, 3.19; found C, 75.85; H, 6.08; N, 3.62.

UV-vis (DCM): λ_{max} (ε) = 407 nm (8200 $M^{-1}cm^{-1}$).

IR (ATR) [cm^{-1}] $\tilde{\nu}$ = 3070 (w, $\tilde{\nu}_{CH}$), 2963 (m, $\tilde{\nu}_{CH}$), 2869 (w, $\tilde{\nu}_{CH}$), 1675 (s, $\tilde{\nu}_{C=O}$).

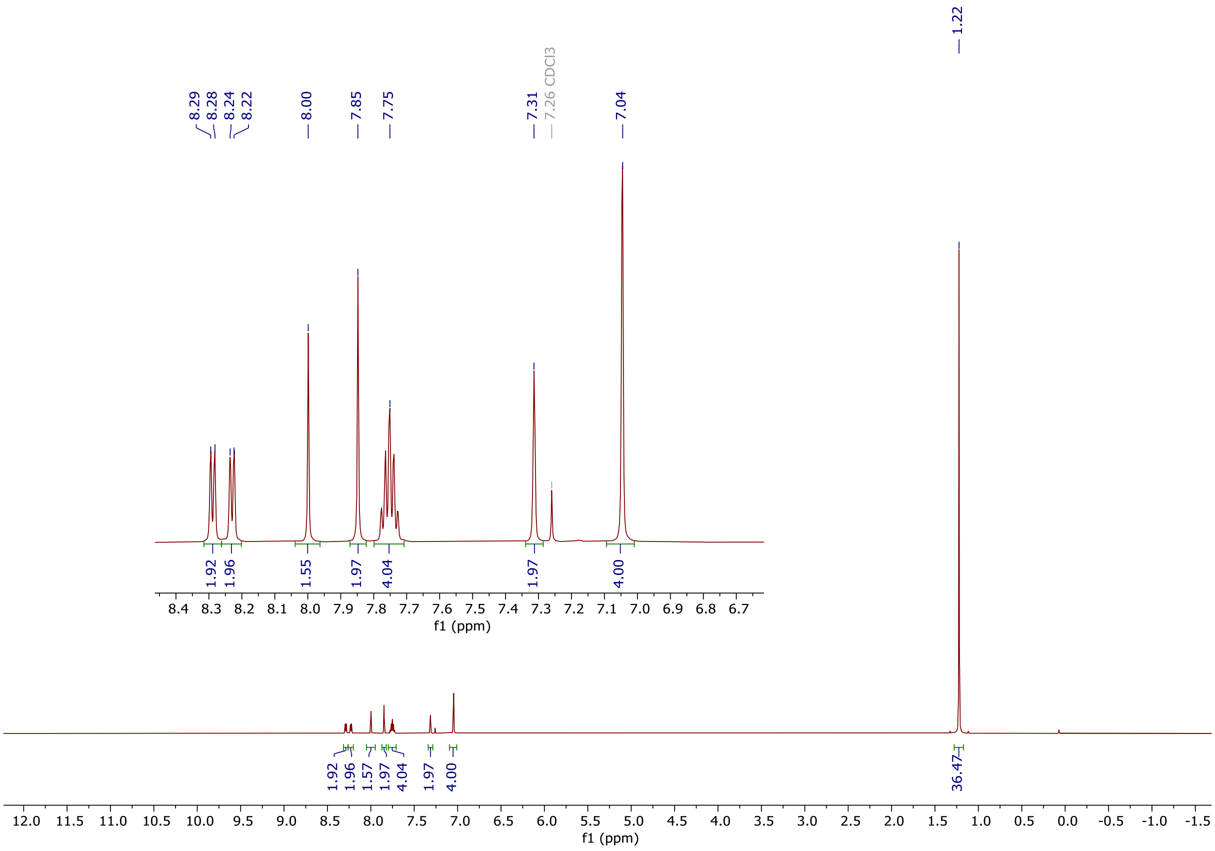

Figure S1.6. ^{1}H NMR spectrum (600 MHz, $CDCl_3$) of **1**.

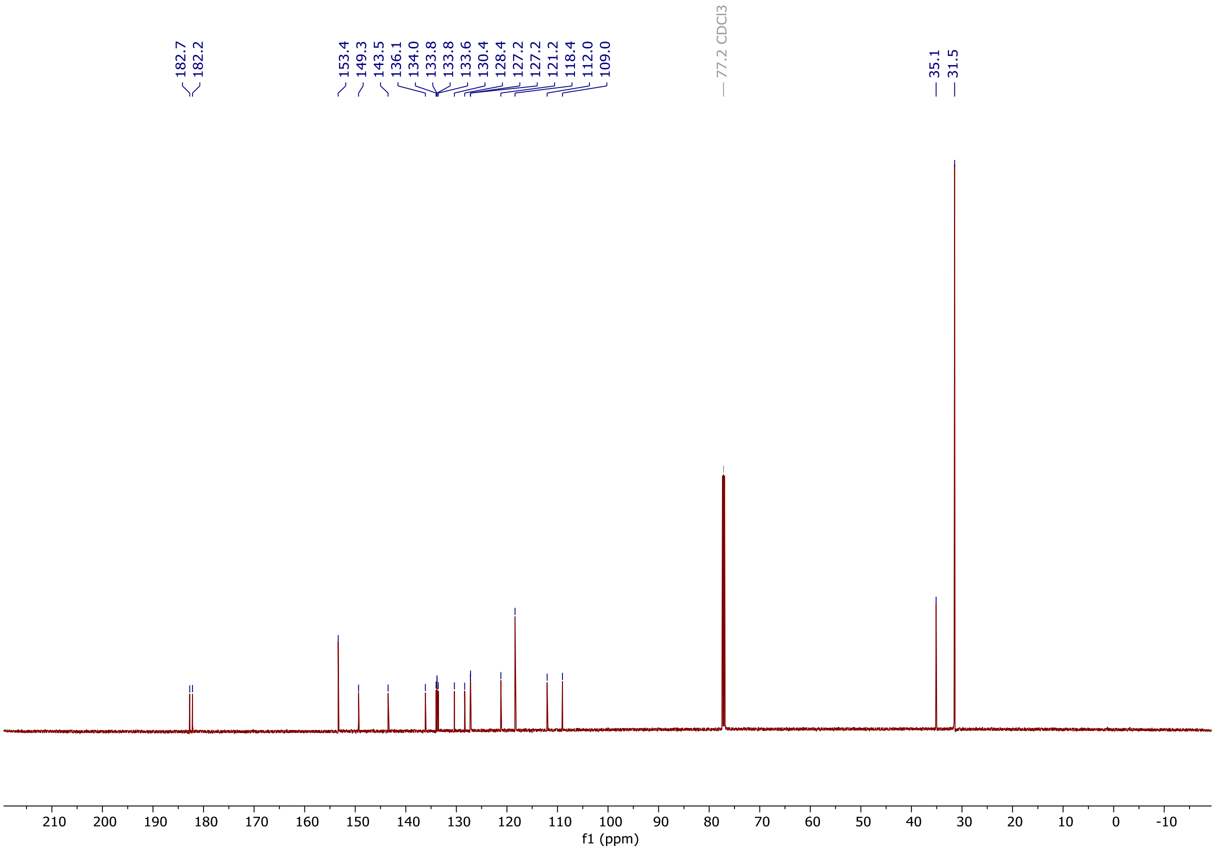

Figure S1.7. ^{13}C NMR spectrum (151 MHz, $CDCl_3$) of **1**.

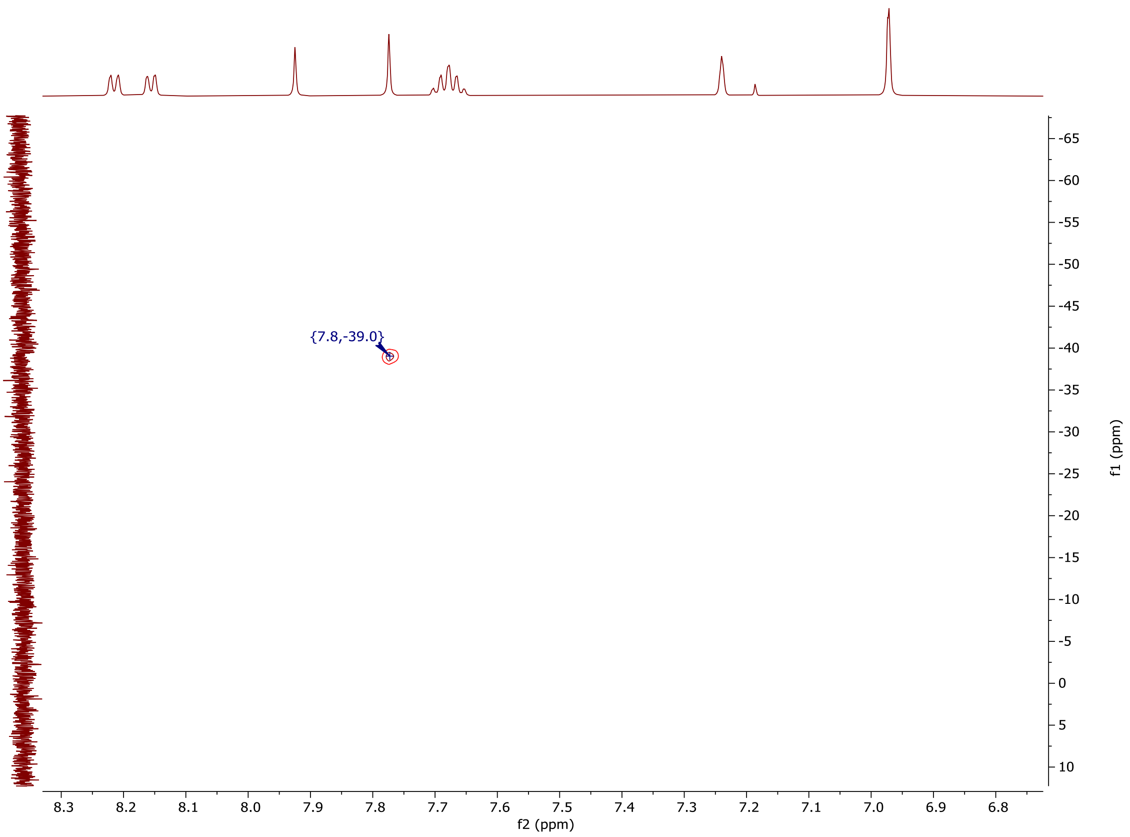

Figure S1.8. ^{1}H-^{29}Si HMBC spectrum (119 MHz, $CDCl_3$) of **1**.

1.5 [1-F][NBu4]

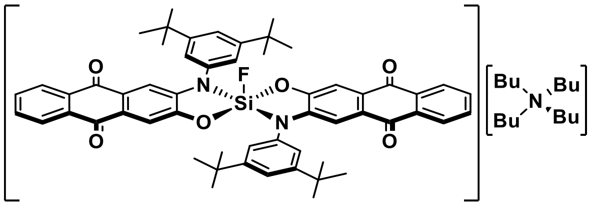

1 (20.0 mg, 22.8 µmol, 1.00 eq) was dissolved in a 1:2 benzene/*n*-hexane mixture (3 ml). TBAT (11.1 mg, 20.5 µmol, 0.90 eq) was added and the mixture stirred for 2 h. The red solution was passed through a syringe filter and the solvent was removed *in vacuo*. The residual sticky solid was triturated with *n*-hexane and filtered. The red solid was washed with *n*-hexane (3 x 2 ml), with a 3:1 *n*-hexane/benzene mixture (3 x 2 ml), and dried *in vacuo* (16.2 mg, 14.2 µmol, 62%).

^{1}H NMR (400 MHz, CDCl3) δ 8.17 – 8.10 (m, 4H), 7.67 – 7.57 (m, 4H), 7.20 (s, 2H), 7.08 (s, 2H), 6.93 (s, 4H), 3.03 – 2.94 (m, 8H), 1.45 (p, *J* = 7.7 Hz, 8H), 1.25 (m, 44H, *overlapping tBu/NBu$_4$*), 0.86 (t, *J* = 7.4 Hz, 12H).

Note: One signal with integral 2 is assumed to be underlying the solvent peak and the peak at 7.20 ppm.

^{13}C NMR (101 MHz, $CDCl_3$) δ 183.6, 182.9, 154.4, 151.0, 148.6, 143.2, 134.4, 134.3, 132.9, 132.8, 127.2, 126.6, 126.4, 126.3, 122.7, 119.4, 107.1, 106.5, 58.8, 34.9, 31.7, 31.6, 23.9, 19.7, 13.7.

^{19}F NMR (377 MHz, $CDCl_3$) δ -110.0 (s, 1F, ^{29}Si-satellites: *J* = 190 Hz).

^{29}Si NMR (80 MHz, $CDCl_3$) δ -107.9 (d, *J* = 190 Hz).

HRMS (ESI-): $[C_{56}H_{54}FN_2O_6Si]^-$, calcd.: 897.3735, found: 897.3948.

HRMS (ESI+): $[C_{16}H_{36}N]^+$, calcd.: 242.2842, found: 242.2830.

UV-vis (DCM): λ_{max} (ε) = 495 nm (6800 $M^{-1}cm^{-1}$).

IR (ATR) [cm^{-1}] $\tilde{\nu}$ = 3067 (w, $\tilde{\nu}_{CH}$), 2964 (m, $\tilde{\nu}_{CH}$), 2877 (w, $\tilde{\nu}_{CH}$), 1661 (m, $\tilde{\nu}_{C=O}$).

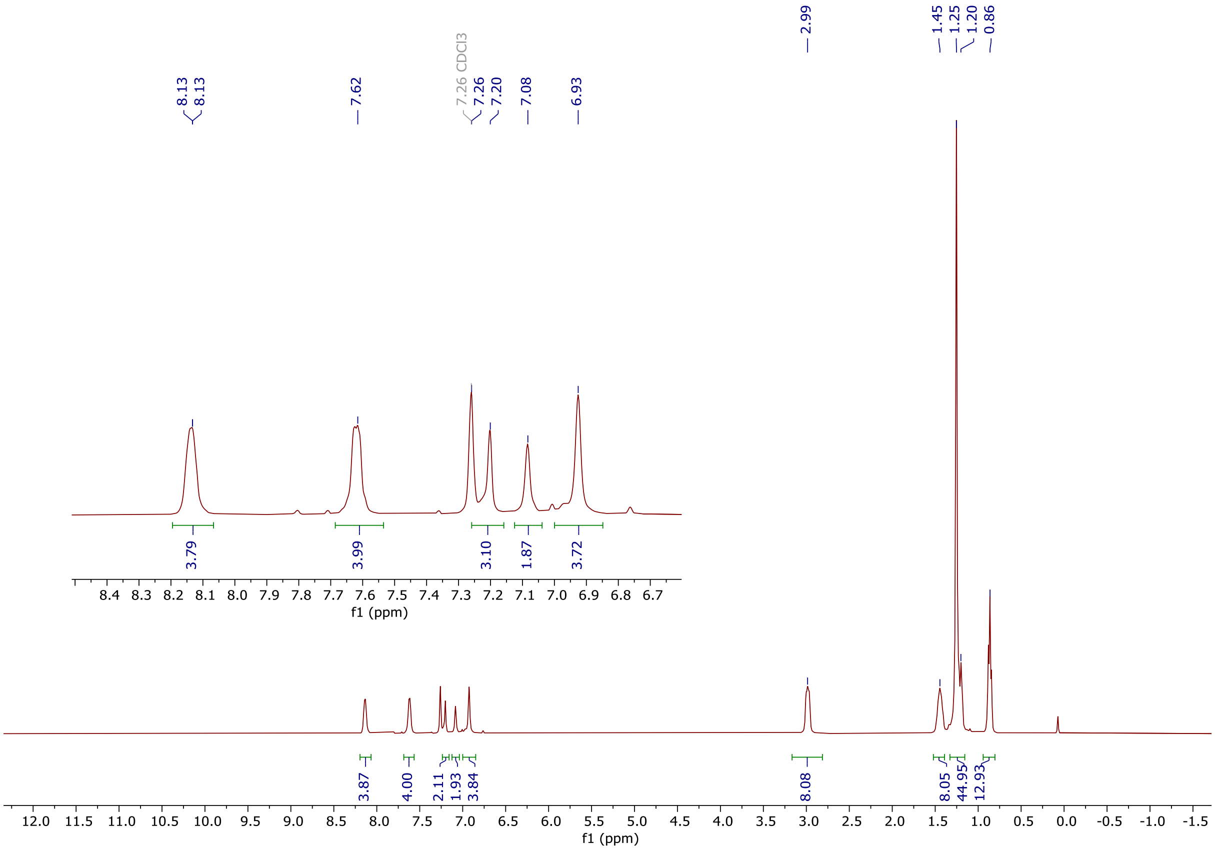

Figure S1.9. ^{1}H NMR spectrum (400 MHz, $CDCl_3$) of **[1-F][NBu$_4$]**.

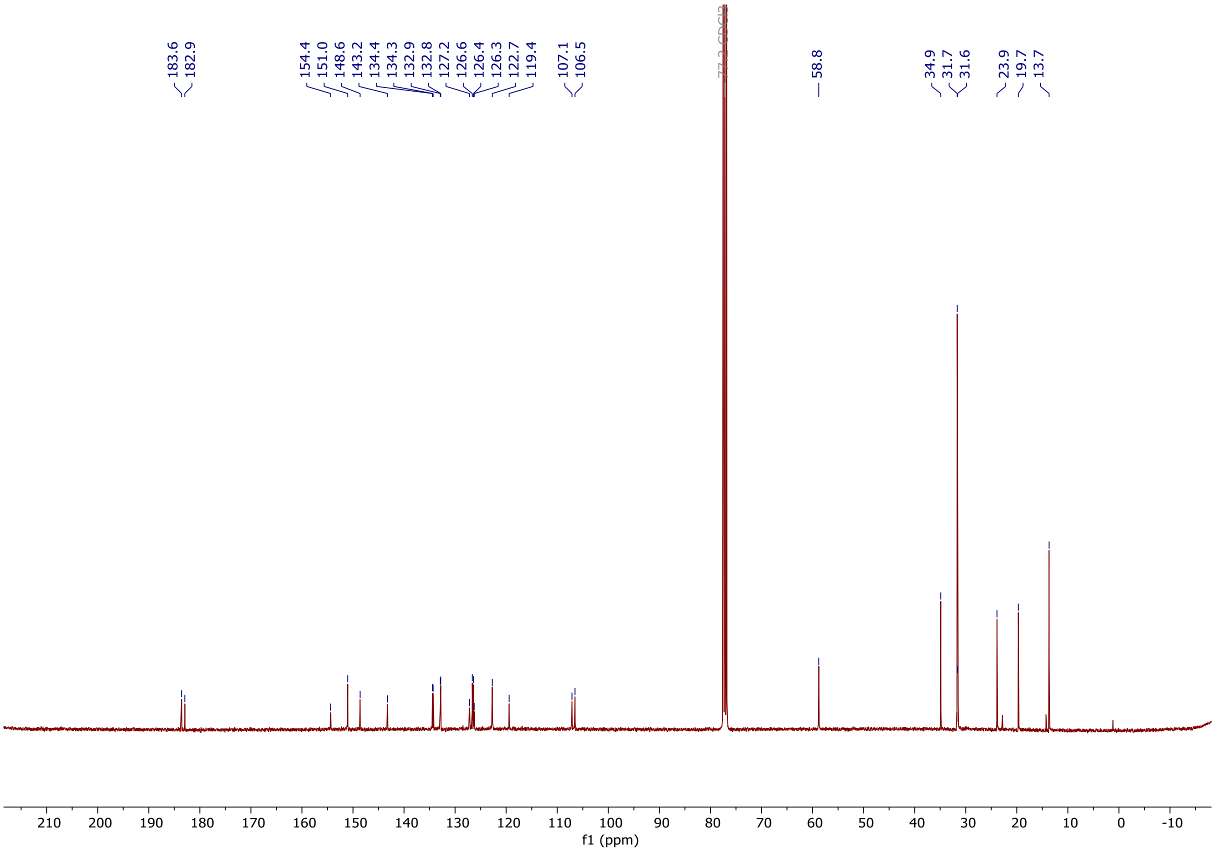

Figure S1.10. ^{13}C NMR spectrum (101 MHz, $CDCl_3$) of **[1-F][NBu$_4$]**.

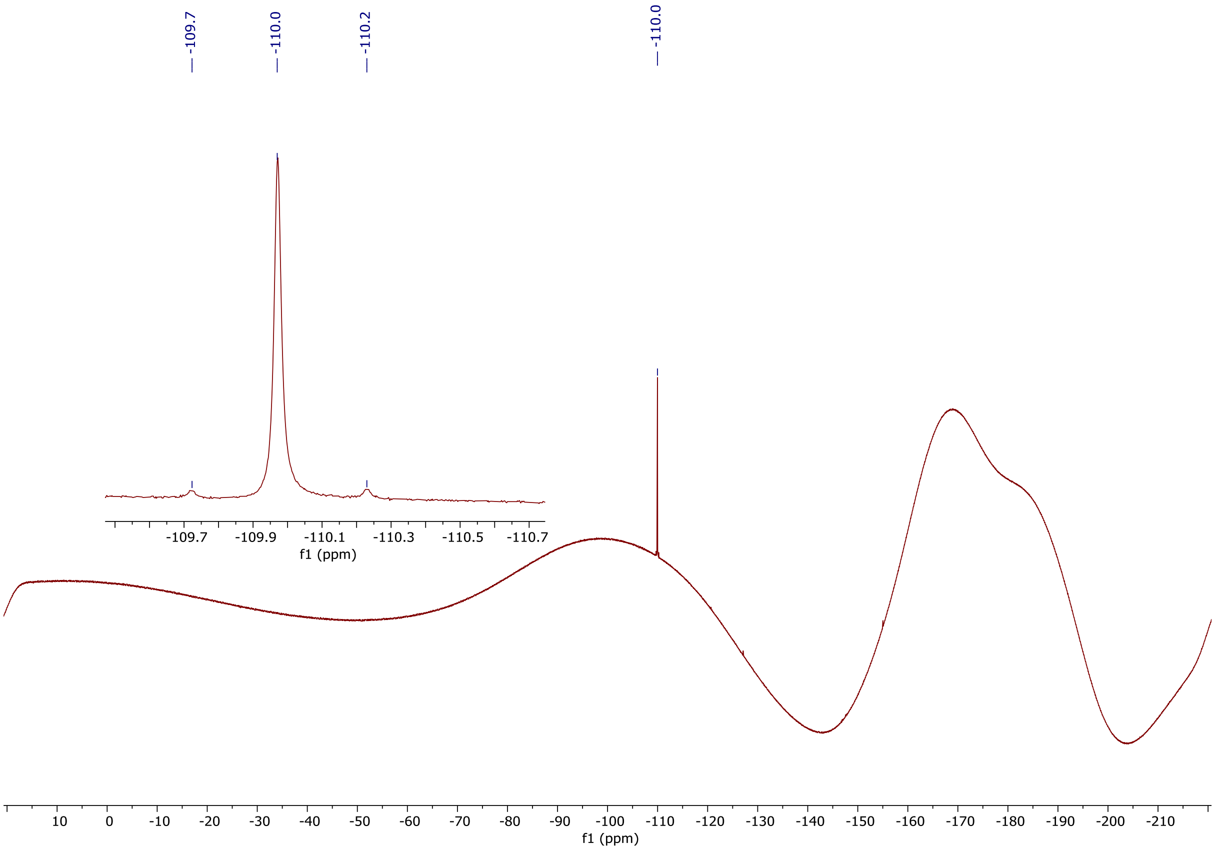

Figure S1.11. ^{19}F NMR spectrum (377 MHz, $CDCl_3$) of **[1-F][NBu$_4$]**.

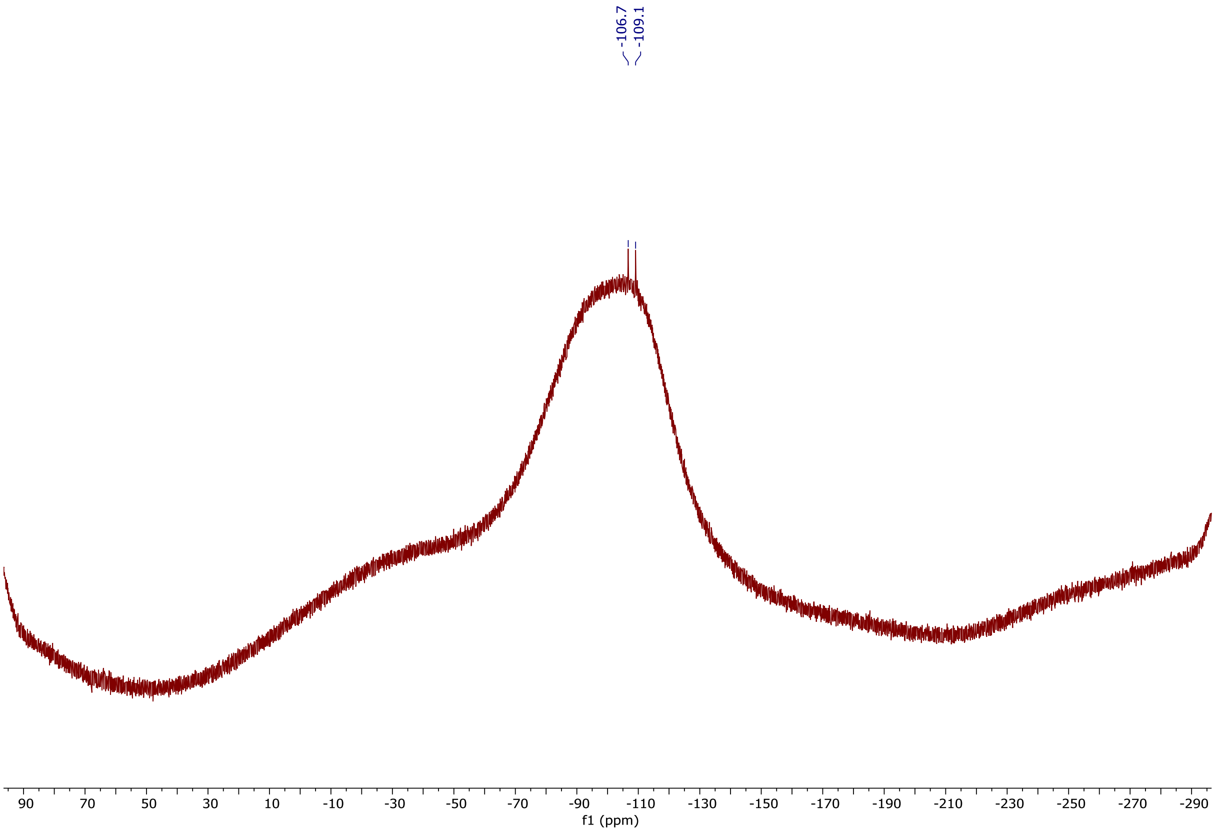

Figure S1.12. ^{29}Si NMR spectrum (80 MHz, $CDCl_3$) of **[1-F][NBu$_4$]**.

1.6 [1-Cl][PPh4]

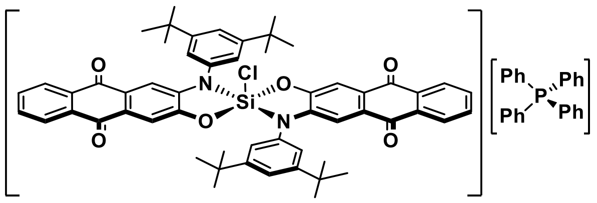

1 (40.0 mg, 45.5 µmol, 1.00 eq) and PPh_4Cl (17.2 mg, 45.5 µmol, 1.00 eq) were dissolved in DCM (2 ml), resulting in a red solution. The solution was layered with *n*-pentane (4 ml) and stored at –40 °C for 72 h, resulting in the formation of bright red crystals, which were filtered of, washed once with DCM/*n*-pentane (1:2, 2 ml) and dried *in vacuo* (56.4 mg, 45.0 µmol, 99%). Single crystals suitable for SCXRD were grown by gaseous diffusion of *n*-pentane into a solution of [1-Cl][PPh_4] in DCM at –40 °C.

^{1}**H NMR** (600 MHz, CD_2Cl_2) δ 8.12 – 8.06 (m, 4H), 7.81 – 7.76 (m, 4H), 7.67 – 7.58 (m, 12H, *overlapping PPh_4/anthraquinone*), 7.55 – 7.49 (m, 8H), 7.39 (s, 2H), 7.12, 7.10 (br s, 4H and s, 2H, overlapping), 7.04 (s, 2H), 1.33 (s, 36H).

^{13}**C NMR** (151 MHz, CD_2Cl_2) δ 183.3, 182.1, 154.1, 151.4, 148.1, 143.7, 136.0 (d, *J* = 3.1 Hz), 134.7 (d, *J* = 10.3 Hz), 134.4, 133.1, 132.9, 130.9 (d, *J* = 12.9 Hz), 127.4, 126.6, 126.5, 126.4, 123.4, 119.5, 117.8 (d, *J* = 89.6 Hz), 107.4, 106.2, 35.2, 31.7.

Note: There is one fewer signal in the ^{13}C due to overlapping signals, with a shoulder peak underlying the doublet at 134.7 ppm.

^{29}**Si NMR** (119 MHz, CD_2Cl_2) δ -98.5.

^{31}**P NMR** (243 MHz, CD_2Cl_2) δ 23.1.

HRMS (ESI-): **[1-Cl]** is not stable under ESI conditions. Ionisation of the ligand occurs. $[C_{28}H_{28}NO_3]^-$, calcd.: 426.2075, found: 426.2002.

HRMS (ESI+): $[C_{24}H_{20}P]^+$, calcd.: 339.1303, found: 339.1292.

UV-vis (DCM): λ_{max} (ε) = 489 nm (7600 $M^{-1}cm^{-1}$).

IR (ATR) [cm^{-1}] $\tilde{\nu}$ = 3065 (w, $\tilde{\nu}_{CH}$), 2953 (m, $\tilde{\nu}_{CH}$), 2902 (w, $\tilde{\nu}_{CH}$), 2864 (w, $\tilde{\nu}_{CH}$), 1659 (m, $\tilde{\nu}_{C=O}$).

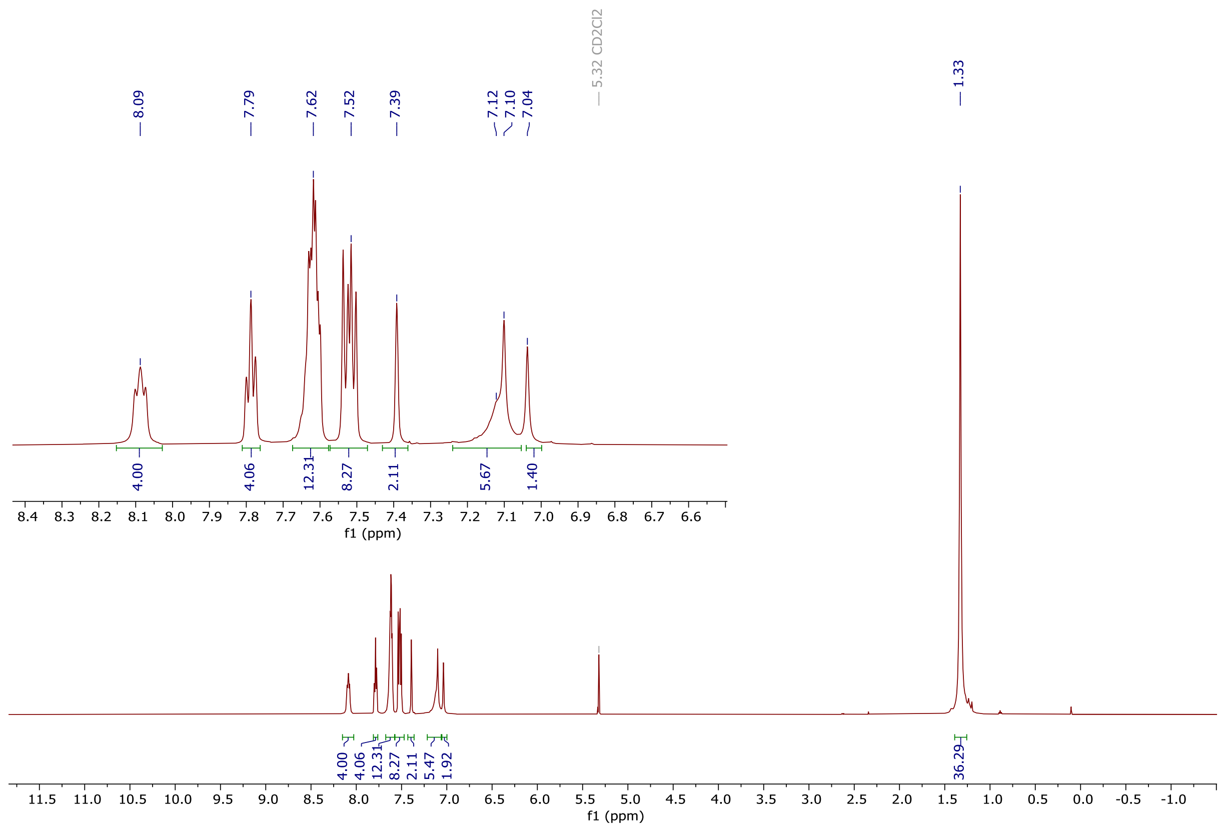

Figure S1.13. ^{1}H NMR spectrum (600 MHz, CD_2Cl_2) of **[1-Cl][PPh$_4$]**.

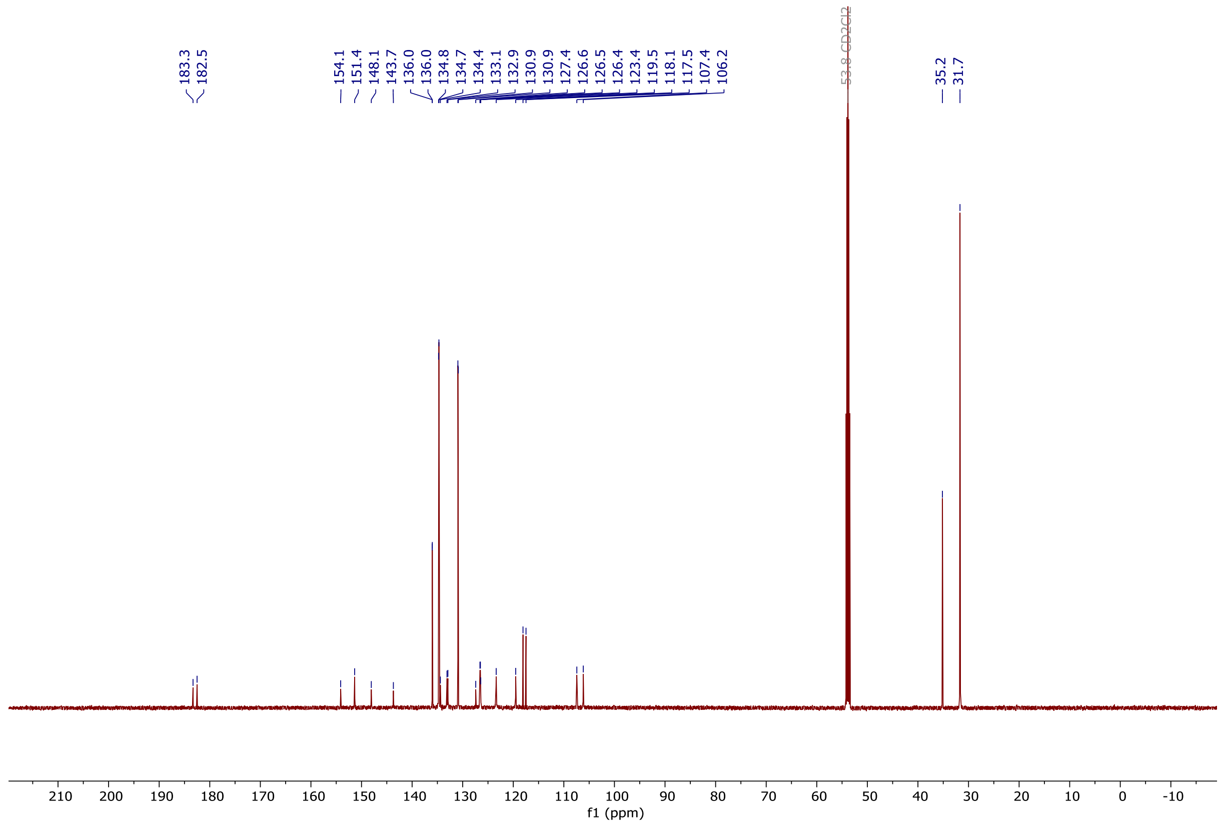

Figure S1.14. ^{13}C NMR spectrum (151 MHz, CD_2Cl_2) of **[1-Cl][PPh$_4$]**.

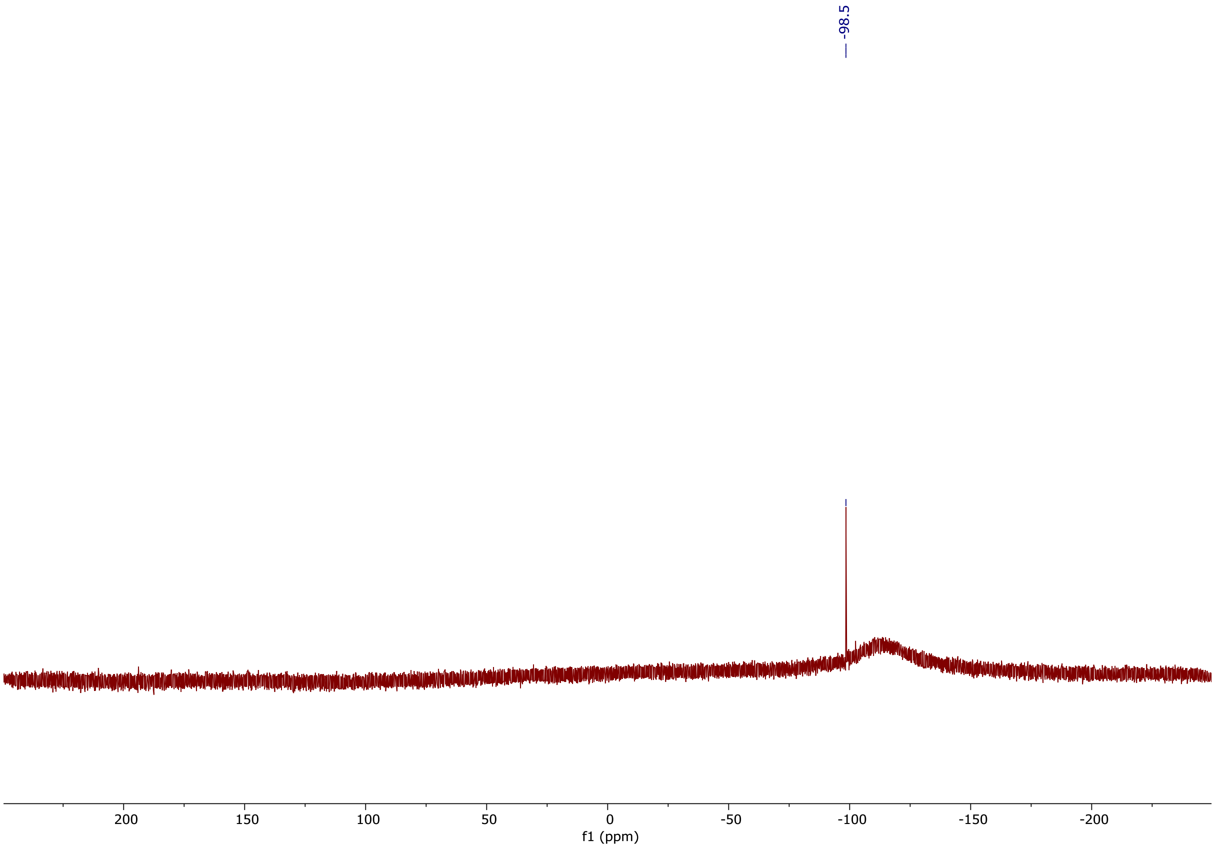

Figure S1.15. ^{29}Si NMR spectrum (119 MHz, CD_2Cl_2) of **[1-Cl][PPh$_4$]**.

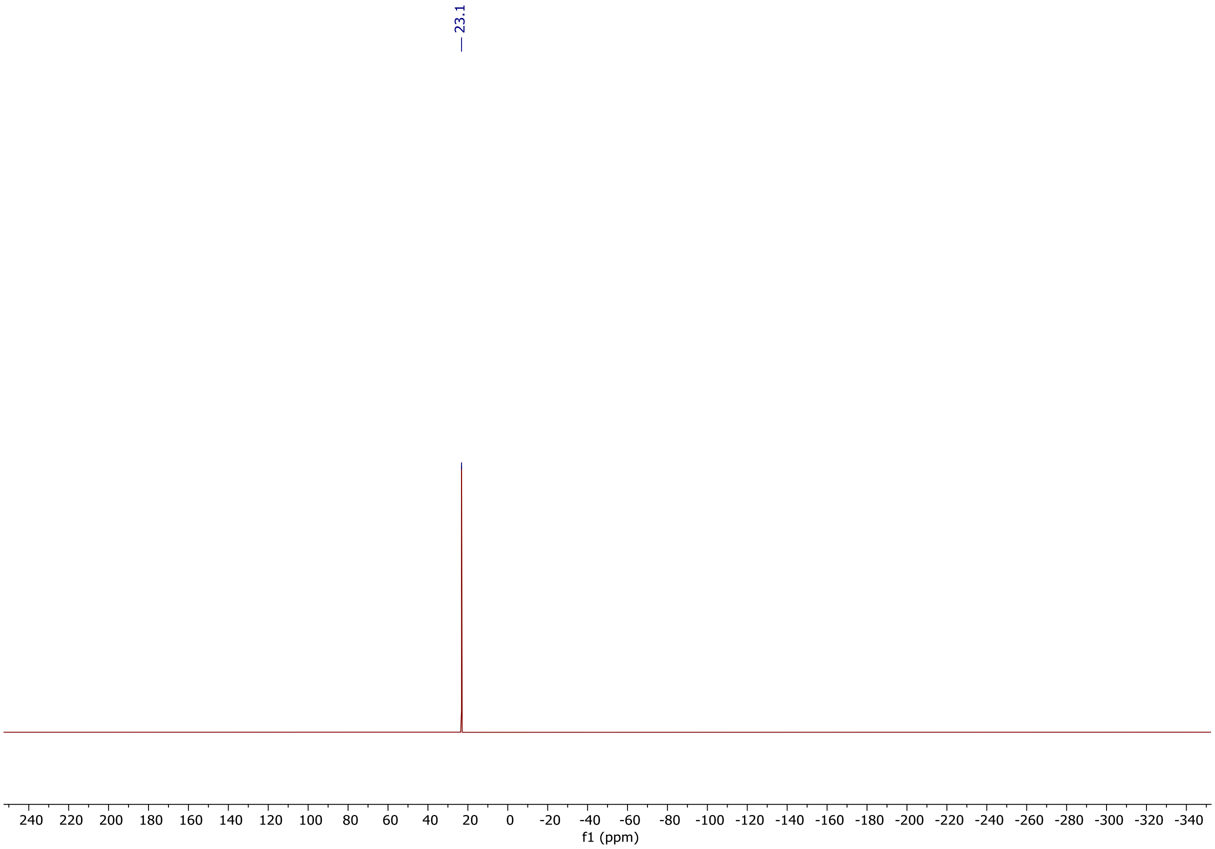

Figure S1.16. ^{31}P NMR spectrum (243 MHz, CD_2Cl_2) of **[1-Cl][PPh$_4$]**.

1.7 [1-CN][NBu4]

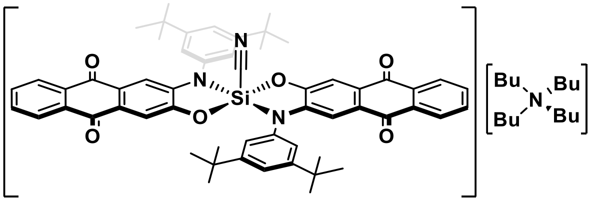

1 (40.0 mg, 45.5 µmol, 1.00 eq) and NBu_4CN (17.2 mg, 45.5 µmol, 1.00 eq) were dissolved in DCM (2 ml), resulting in an orange solution. The solution was layered with *n*-pentane (4 ml) and stored at –40 °C for 72 h, resulting in the separation of an oily layer at the bottom of the vessel. The supernatant solution was decanted off, the residue dissolved in benzene (1 ml) and the solvent removed by freeze-drying overnight, resulting in an orange solid (29.8 mg, 26.0 µmol, 57%).

^{1}H NMR (600 MHz, CD_2Cl_2) δ 8.17 – 8.08 (m, 4H), 7.70 – 7.63 (m, 4H), 7.37 (s, 2H), 7.13 (s, 2H), 7.11 (s, 2H), 7.00 (br s, 4H), 1.56 – 1.47 (m, 8H), 1.36 – 1.22 (m, 44H, *overlapping NBu_4/tBu*), 0.92 (t, *J* = 7.3 Hz, 12H).

^{13}C NMR (151 MHz, CD_2Cl_2) δ 183.2, 182.7, 155.4, 151.6, 148.0, 142.6, 134.6, 134.4, 133.3, 133.2, 129.2 (*C*≡N), 127.6, 126.9, 126.7, 126.6, 123.1, 120.0, 107.7, 106.8, 59.2, 35.1, 31.6, 24.1, 20.0, 13.7.

^{29}Si NMR (119 MHz, CD_2Cl_2) δ -113.7.

HRMS (ESI-): $[C_{57}H_{54}N_3O_6Si]^-$, calcd.: 904.3787, found: 904.3792.

HRMS (ESI+): $[C_{16}H_{36}N]^+$, calcd.: 242.2842, found: 242.2830.

UV-vis (DCM): λ_{max} (ε) = 481 nm (8200 $M^{-1}cm^{-1}$).

IR (ATR) [cm^{-1}] $\tilde{\nu}$ = 3065 (w, $\tilde{\nu}_{CH}$), 2961 (m, $\tilde{\nu}_{CH}$), 2873 (w, $\tilde{\nu}_{CH}$), 1661 (m, $\tilde{\nu}_{C=O}$).

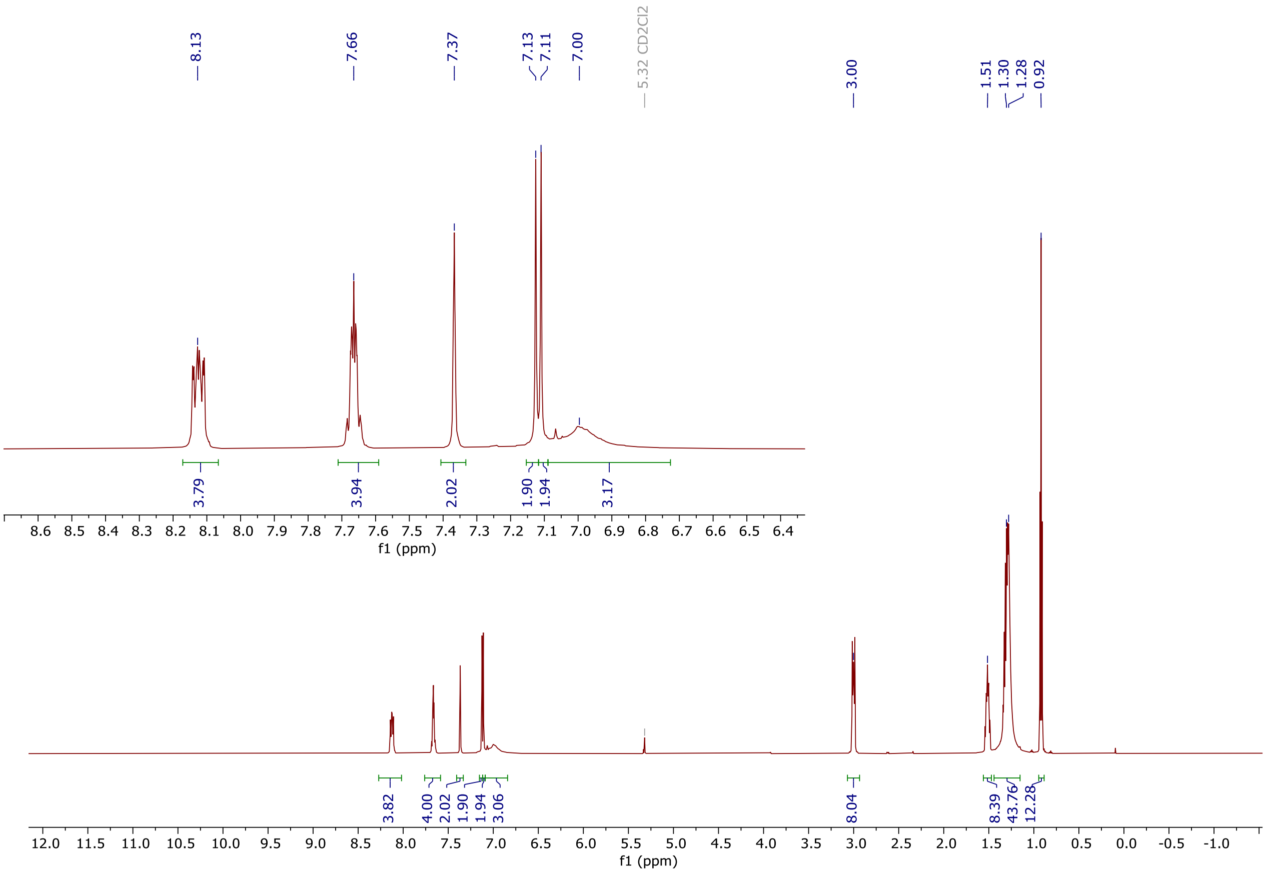

Figure S1.17. ^{1}H NMR spectrum (600 MHz, CD_2Cl_2) of **[1-CN][NBu4]**.

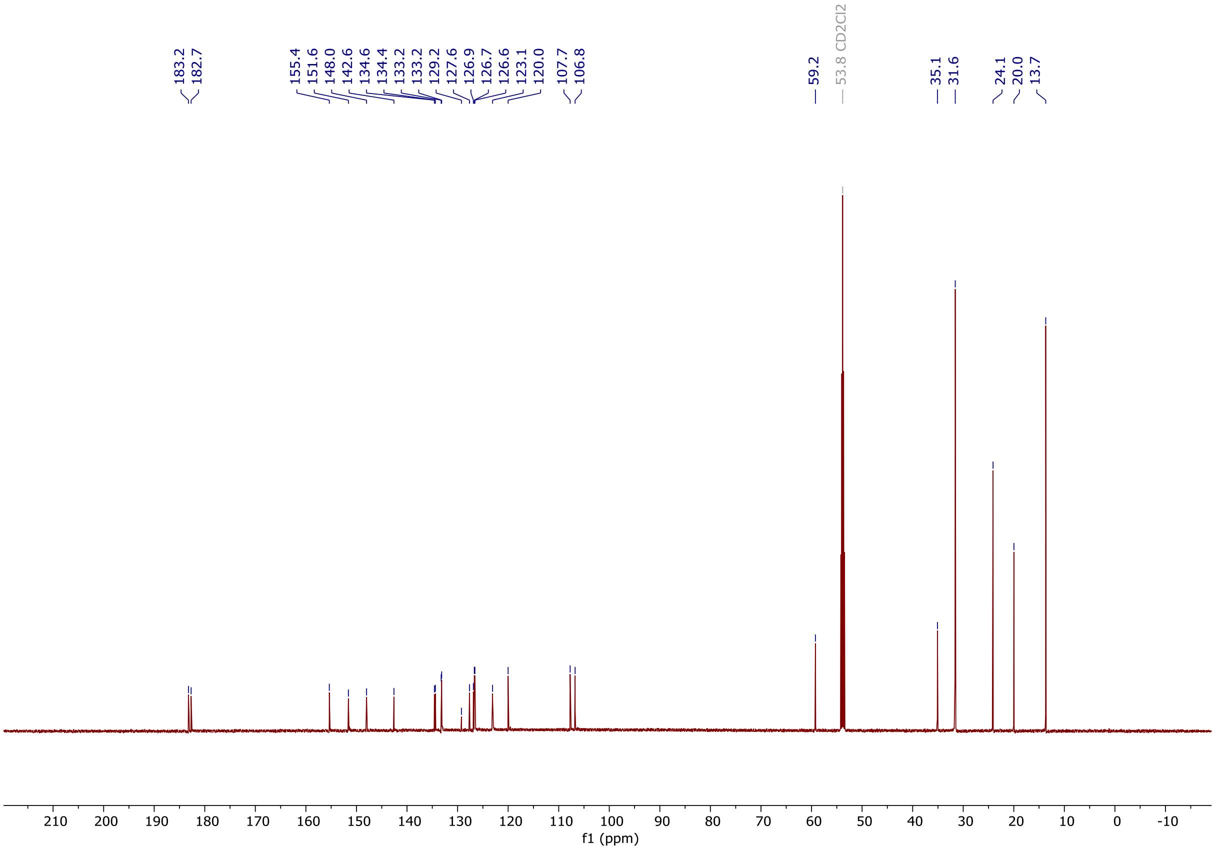

Figure S1.18. ^{13}C NMR spectrum (151 MHz, CD_2Cl_2) of **[1-CN][NBu₄]**.

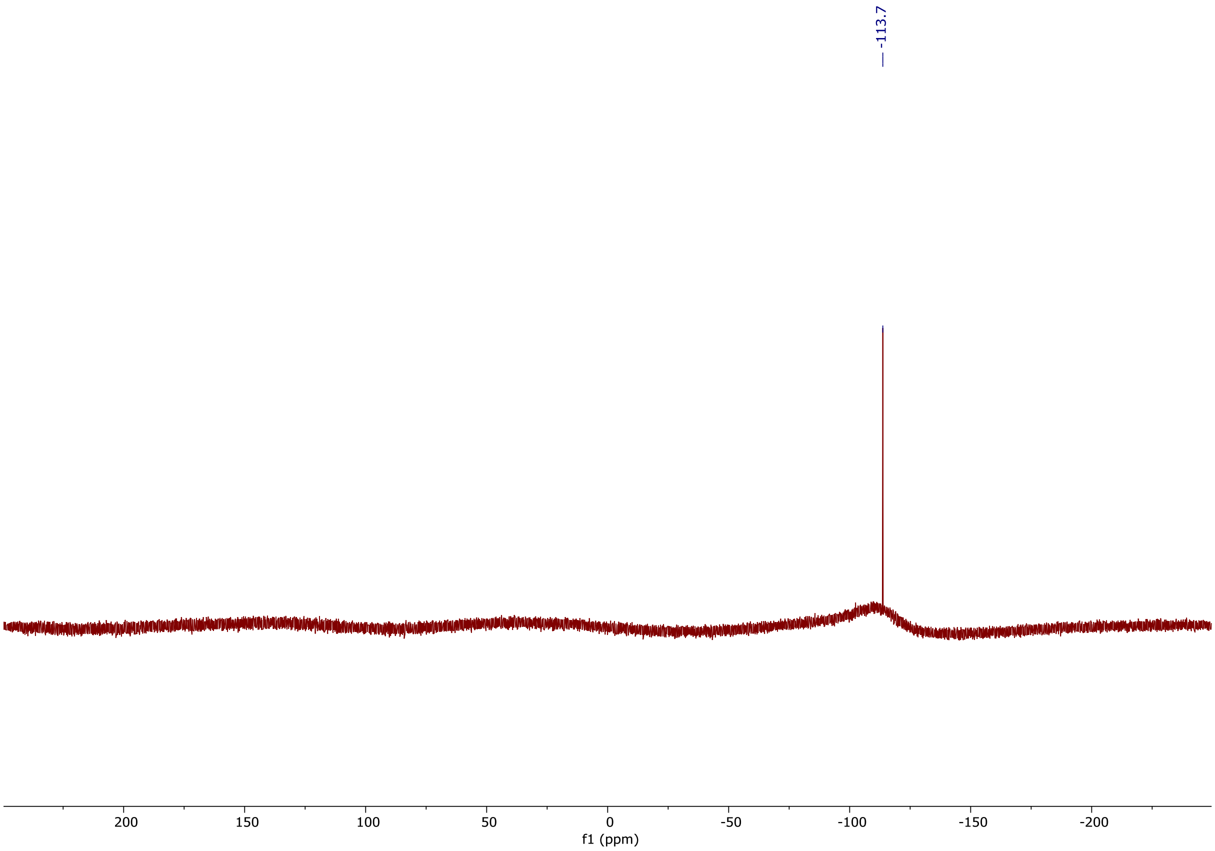

Figure S1.19. ^{29}Si NMR spectrum (119 MHz, CD_2Cl_2) of **[1-CN][NBu$_4$]**.

1.8 [1-NCS][NBu_4]

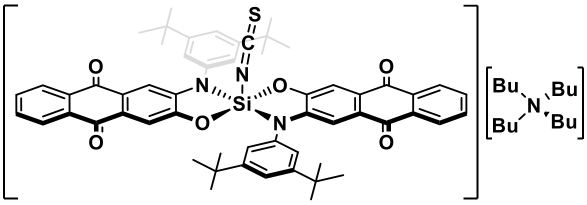

1 (40.0 mg, 45.5 µmol, 1.00 eq) and NBu_4SCN (13.0 mg, 43.2 µmol, 0.95 eq) were dissolved in benzene (2 ml), resulting in a red solution. Scratching the glass surface of the vessel or adding a seed crystal from a previous run induced crystallisation, resulting in the formation of dark orange crystals, which were filtered off, washed with benzene (3x1 ml), and dried *in vacuo* overnight (48.8 mg, 41.4 µmol, 91%). The product contains some residual benzene which could not be removed even with prolonged drying *in vacuo*.

^{1}H NMR (600 MHz, $CDCl_3$) δ 8.17 – 8.10 (m, 4H), 7.67 – 7.59 (m, 4H), 7.27 (t, *J* = 1.8 Hz, 2H), 7.23 (s, 2H), 7.09 (s, 2H), 6.95 (br s, 4H), 2.99 – 2.93 (m, 8H), 1.49 – 1.40 (m, 8H), 1.30 – 1.16 (m, 44H, *overlapping NBu_4/tBu*), 0.86 (t, *J* = 7.3 Hz, 12H).

^{13}C NMR (151 MHz, $CDCl_3$) δ 183.4, 182.9, 154.3, 151.1, 148.1, 142.4, 134.3, 134.2, 133.0, 133.0, 127.4, 126.7, 126.4, 126.2, 122.7, 119.6, 107.3, 106.9, 59.0, 34.9, 31.6, 23.9, 19.7, 13.7.

^{29}Si NMR (119 MHz, CD_2Cl_2) δ -116.0 (t, *J* = 27.0 Hz).

^{14}N NMR (29 MHz, CD_2Cl_2) δ 88, 22.

HRMS (ESI-): **[1-NCS]** is not stable under ESI conditions. Instead, ionisation of the ligand occurs. $[C_{28}H_{28}NO_3]^-$, calcd.: 426.2075, found: 426.2002.

HRMS (ESI+): $[C_{16}H_{36}N]^+$, calcd.: 242.2842, found: 242.2830.

UV-vis (DCM): λ_{max} (ε) = 484 nm (6700 $M^{-1}cm^{-1}$).

IR (ATR) [cm^{-1}] $\tilde{\nu}$ = 3063 (w, $\tilde{\nu}_{CH}$), 2960 (m, $\tilde{\nu}_{CH}$), 2869 (w, $\tilde{\nu}_{CH}$), 2083 (s, $\tilde{\nu}_{N=C=S}$), 1662 (m, $\tilde{\nu}_{C=O}$).

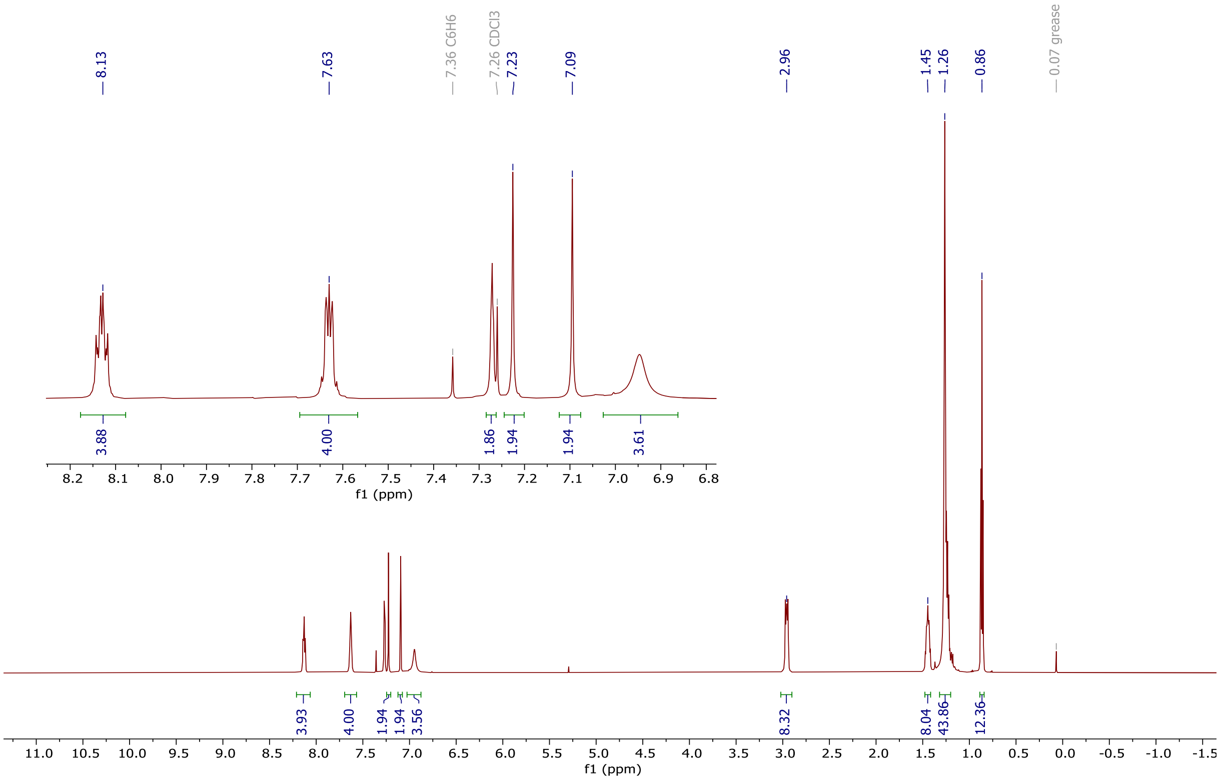

Figure S1.20. ^{1}H NMR spectrum (600 MHz, $CDCl_3$) of **[1-NCS][NBu₄]**.

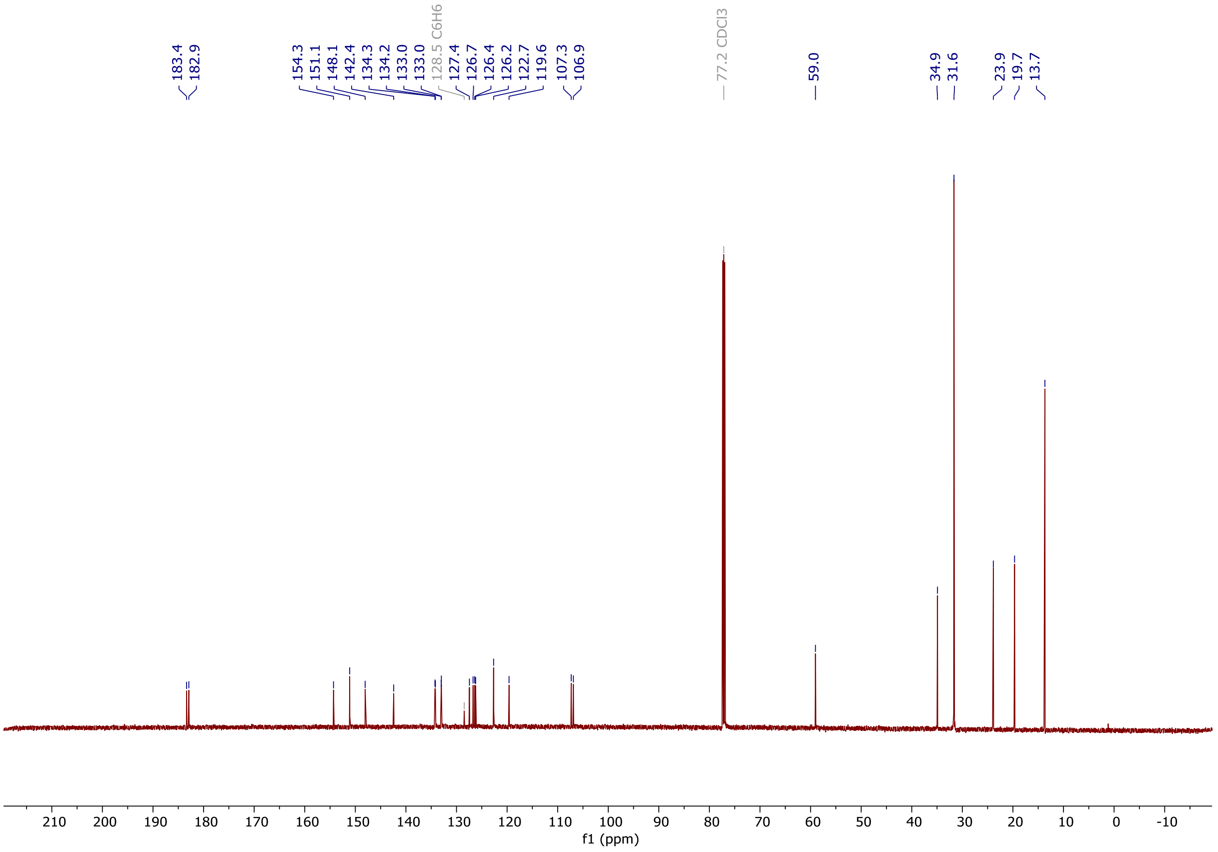

Figure S1.21. ^{13}C NMR spectrum (151 MHz, $CDCl_3$) of **[1-NCS][NBu$_4$]**.

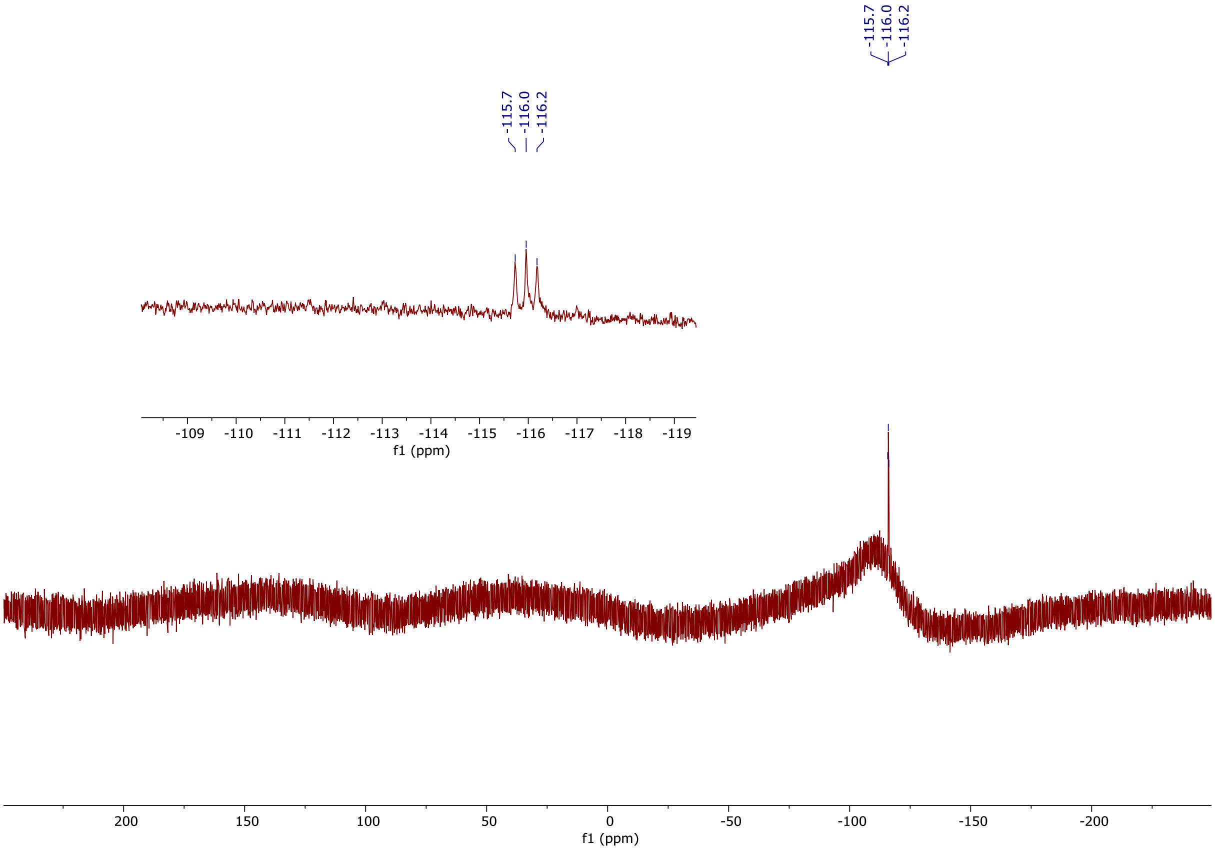

Figure S1.22. ^{29}Si NMR spectrum (119 MHz, CD_2Cl_2) of **[1-NCS][NBu$_4$]**.

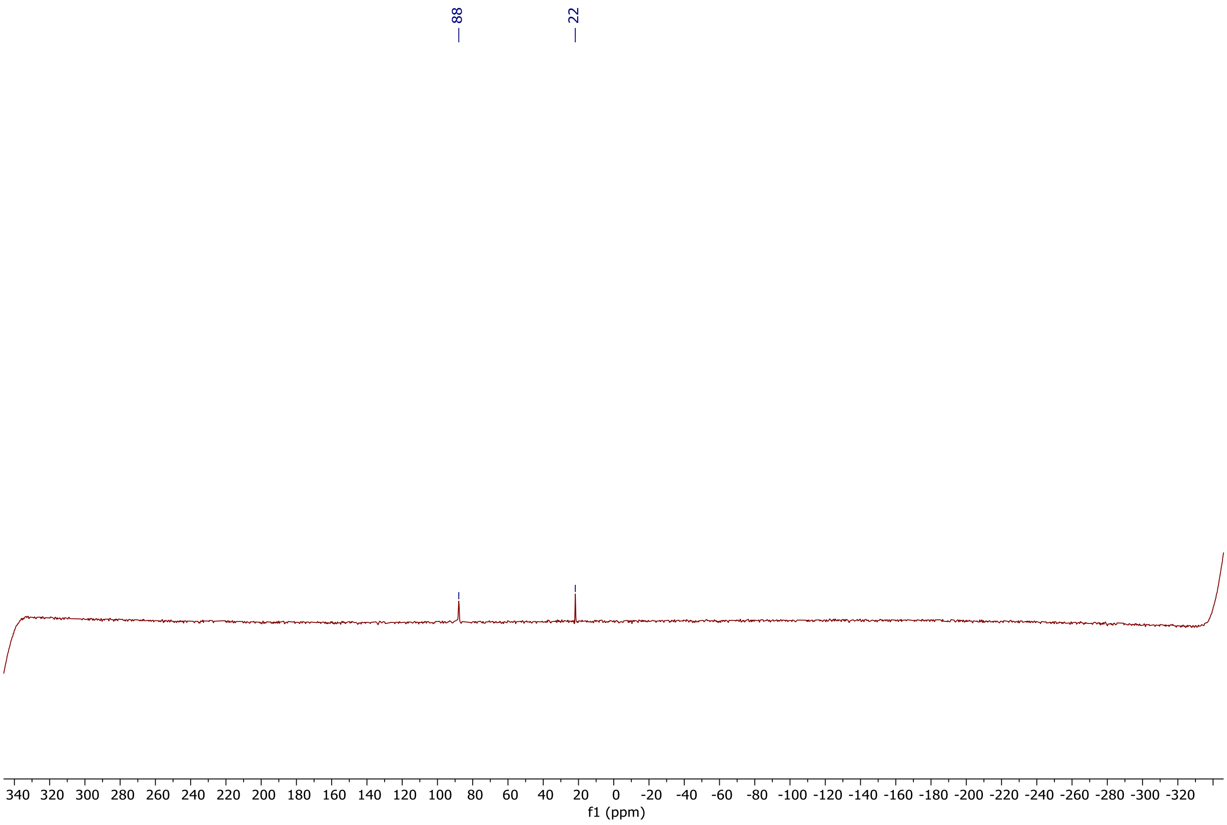

Figure S1.23. ^{14}N NMR spectrum (29 MHz, CD_2Cl_2) of **[1-NCS][NBu$_4$]**.

1.9 [1-N_3][NBu_4]

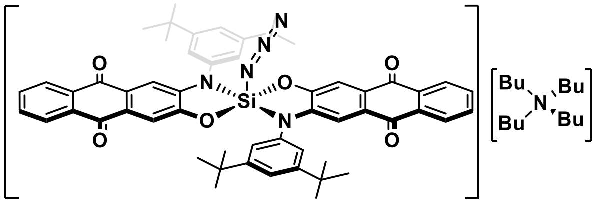

1 (40.0 mg, 45.5 µmol, 1.00 eq) and NBu_4N_3 (12.9 mg, 45.5 µmol, 1.00 eq) were dissolved in benzene (2 ml), resulting in a red solution. The solution was layered with *n*-pentane (4 ml) and stored at room temperature overnight, resulting in the separation of an oily layer at the bottom of the vessel. The supernatant solution was decanted off, the residue redissolved in benzene (2 ml) and the solvent removed by lyophilisation overnight. The product was isolated as a red powder (38.2 mg, 33.0 µmol, 73%). The product still contains benzene, even after drying *in vacuo* overnight.

^{1}H NMR (600 MHz, $CDCl_3$) δ 8.15 – 8.09 (m, 4H), 7.65 – 7.58 (m, 4H), 7.25 (t, *J* = 1.8 Hz, 2H), 7.19 (s, 2H), 7.05 (s, 2H), 6.95 (br s, 4H), 2.97 – 2.92 (m, 8H), 1.48 – 1.39 (m, 8H), 1.29 – 1.18 (m, 44H, overlapping NBu_4/tBu), 0.88 (t, *J* = 7.3 Hz, 12H).

^{13}C NMR (151 MHz, $CDCl_3$) δ 183.5, 182.9, 155.0, 151.0, 148.6, 142.9, 134.4, 134.2, 132.9, 132.9, 127.3, 126.7, 126.3, 126.1, 122.7, 119.4, 107.1, 106.6, 58.8, 34.9, 31.6, 23.8, 19.7, 13.6.

^{29}Si NMR (80 MHz, $CDCl_3$) δ -107.6.

HRMS (ESI-): [$C_{56}H_{54}N_5O_6Si$]$^-$, calcd.: 920.3843, found: 920.3850.

HRMS (ESI+): [$C_{16}H_{36}N$]$^+$, calcd.: 242.2842, found: 242.2830.

UV-vis (DCM): λ_{max} (ε) = 496 nm (7300 $M^{-1}cm^{-1}$).

IR (ATR) [cm^{-1}] $\tilde{\nu}$ = 3066 (w, $\tilde{\nu}_{CH}$), 2961 (m, $\tilde{\nu}_{CH}$), 2874 (w, $\tilde{\nu}_{CH}$), 2121 (m, $\tilde{\nu}_{N=N=N}$), 1660 (m, $\tilde{\nu}_{C=O}$).

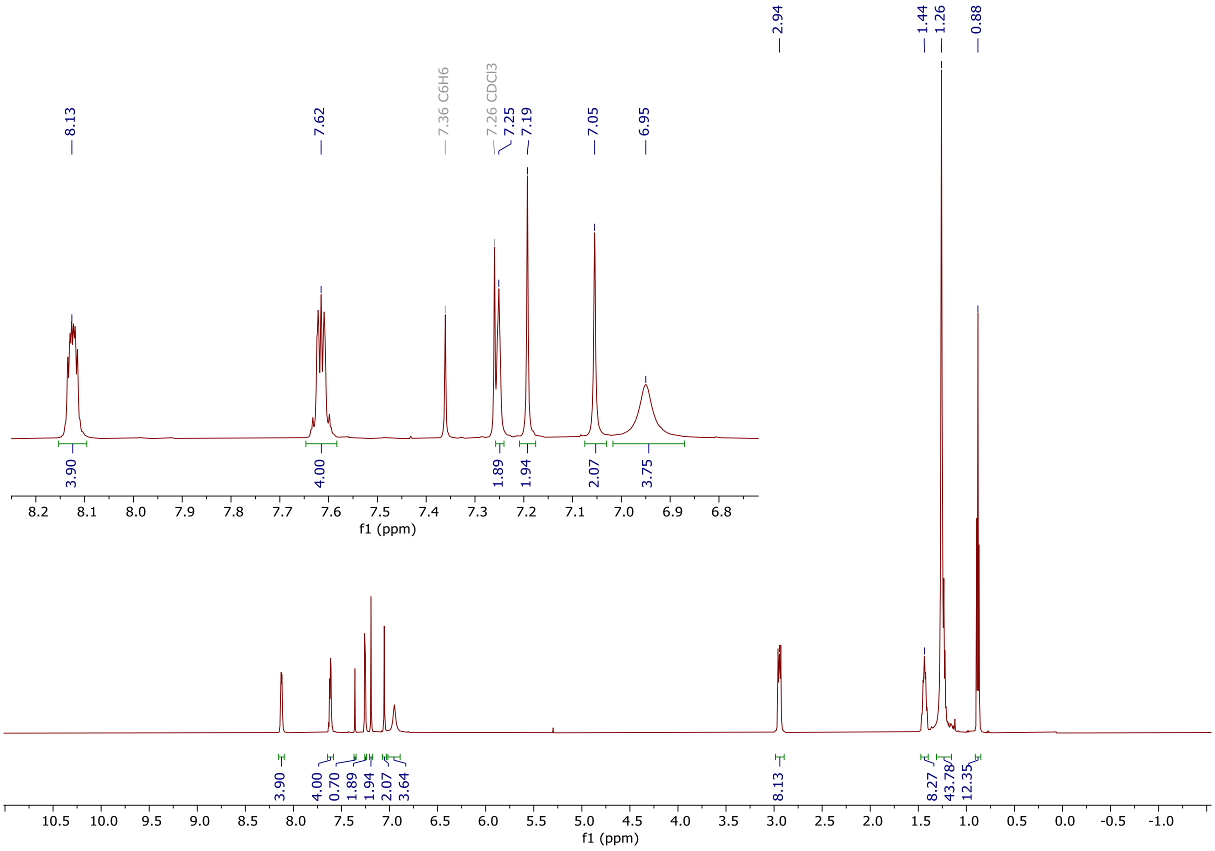

Figure S1.24. ^{1}H NMR spectrum (600 MHz, $CDCl_3$) of **[1-N$_3$][NBu$_4$]**.

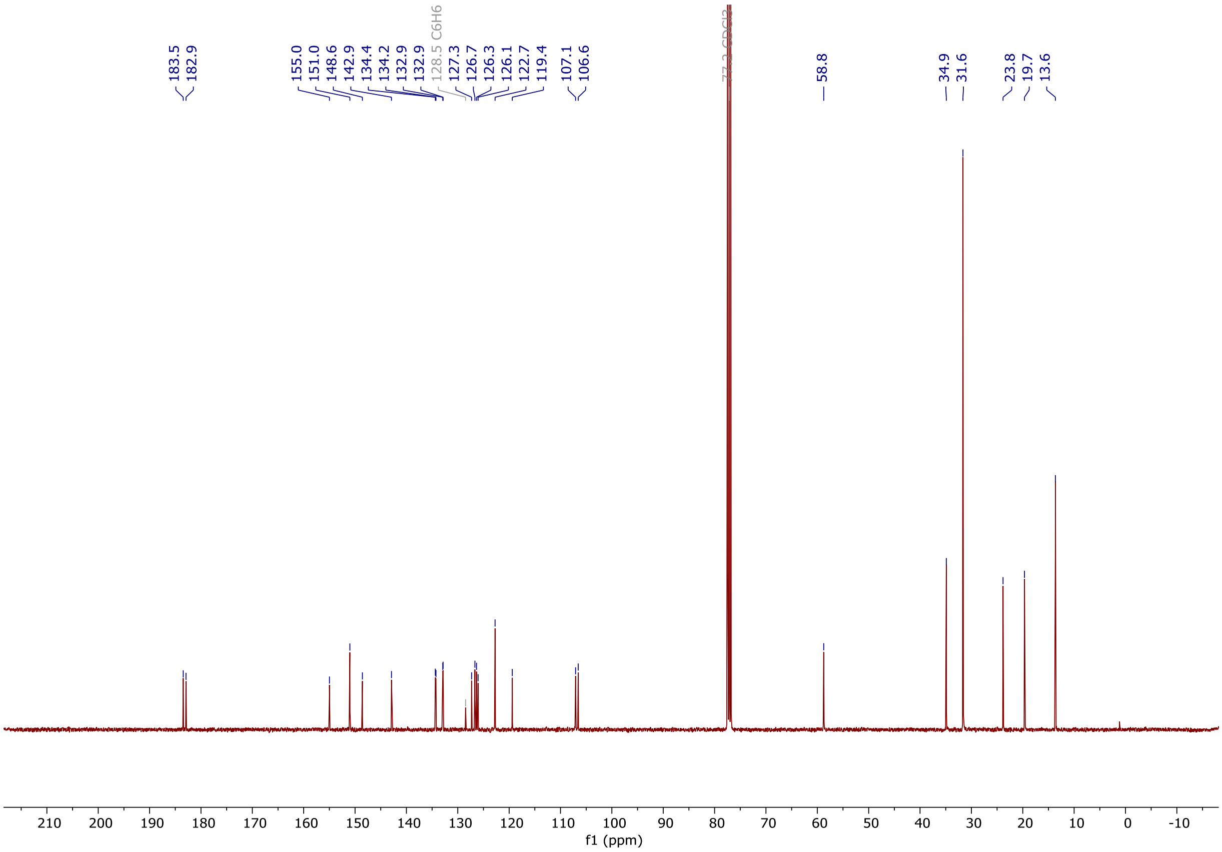

Figure S1.25. ^{13}C NMR spectrum (151 MHz, $CDCl_3$) of **[1-N$_3$][NBu$_4$]**.

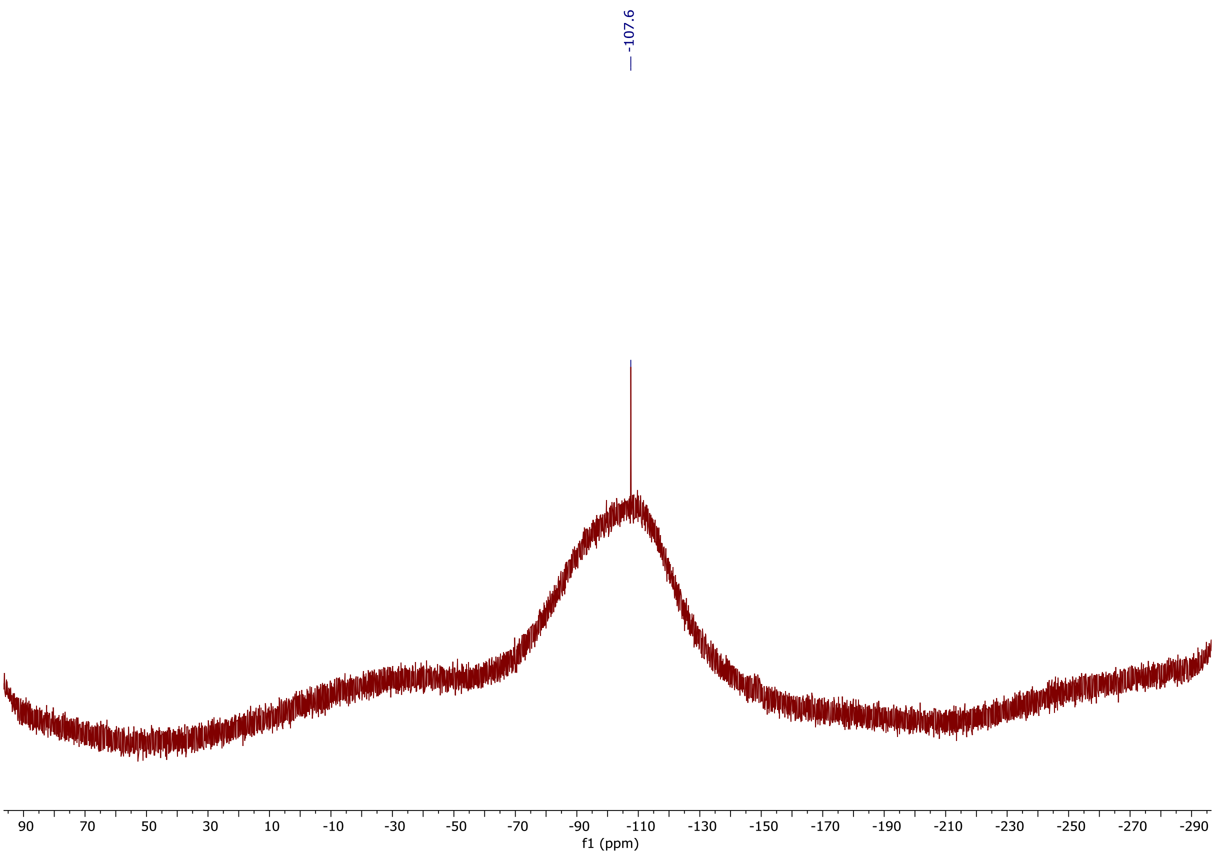

Figure S1.26. ^{29}Si NMR spectrum (80 MHz, $CDCl_3$) of **[1-N_3][NBu_4]**.

1.10 1-(pyridine)$_2$

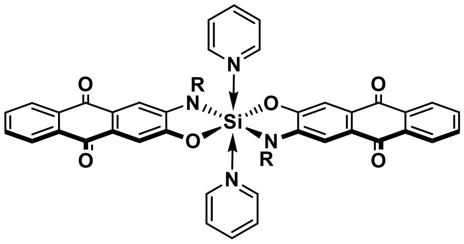

1 (40.0 mg, 45.5 µmol, 1.00 eq) and pyridine (8.1 µl, 0.10 mmol, 2.2 eq) were dissolved in DCM (1 ml), resulting in an orange solution. The solution was layered with *n*-pentane (4 ml) and stored at –40 °C overnight, resulting in the precipitation of a dark red solid. The solid was filtered off, washed with a 2:1 *n*-pentane/DCM mixture (2x1 ml), and dried *in vacuo* (38.3 mg, 36.9 µmol, 81%).

^{1}H NMR (600 MHz, $CDCl_3$) δ 8.75 – 8.71 (m, 4H), 8.22 – 8.13 (m, 4H), 7.93 (t, *J* = 7.6 Hz, 2H), 7.70 – 7.62 (m, 4H), 7.52 (t, *J* = 6.7 Hz, 4H), 7.40 (s, 2H), 7.38 (s, 2H), 7.35 (s, 2H), 7.00 (s, 4H), 1.33 (s, 36H).

^{13}C NMR (101 MHz, $CDCl_3$) δ 183.3, 182.7, 152.3, 152.0, 147.9, 147.0, 141.8, 140.2, 134.1, 133.9, 133.3, 133.2, 128.6, 127.0, 126.9, 126.7, 125.3, 122.4, 120.2, 108.8, 107.8, 35.1, 31.7.

^{29}Si NMR (80 MHz, $CDCl_3$) δ -104.9.

MS (LIFDI+): *Due to the lability of binding pyridine, only the parent complex **1** was detected*. $[C_{56}H_{54}N_2O_6Si]^+$, calcd.: 878.38, found: 878.34.

UV-vis (mono-adduct **1-pyridine**, DCM): λ_{max} (ε) = 452 nm (7300 $M^{-1}cm^{-1}$).

IR (ATR) [cm^{-1}] $\tilde{\nu}$ = 3063 (w, $\tilde{\nu}_{CH}$), 2956 (m, $\tilde{\nu}_{CH}$), 2863 (w, $\tilde{\nu}_{CH}$), 1662 (m, $\tilde{\nu}_{C=O}$), 1646 (w, $\tilde{\nu}_{C=O}$).

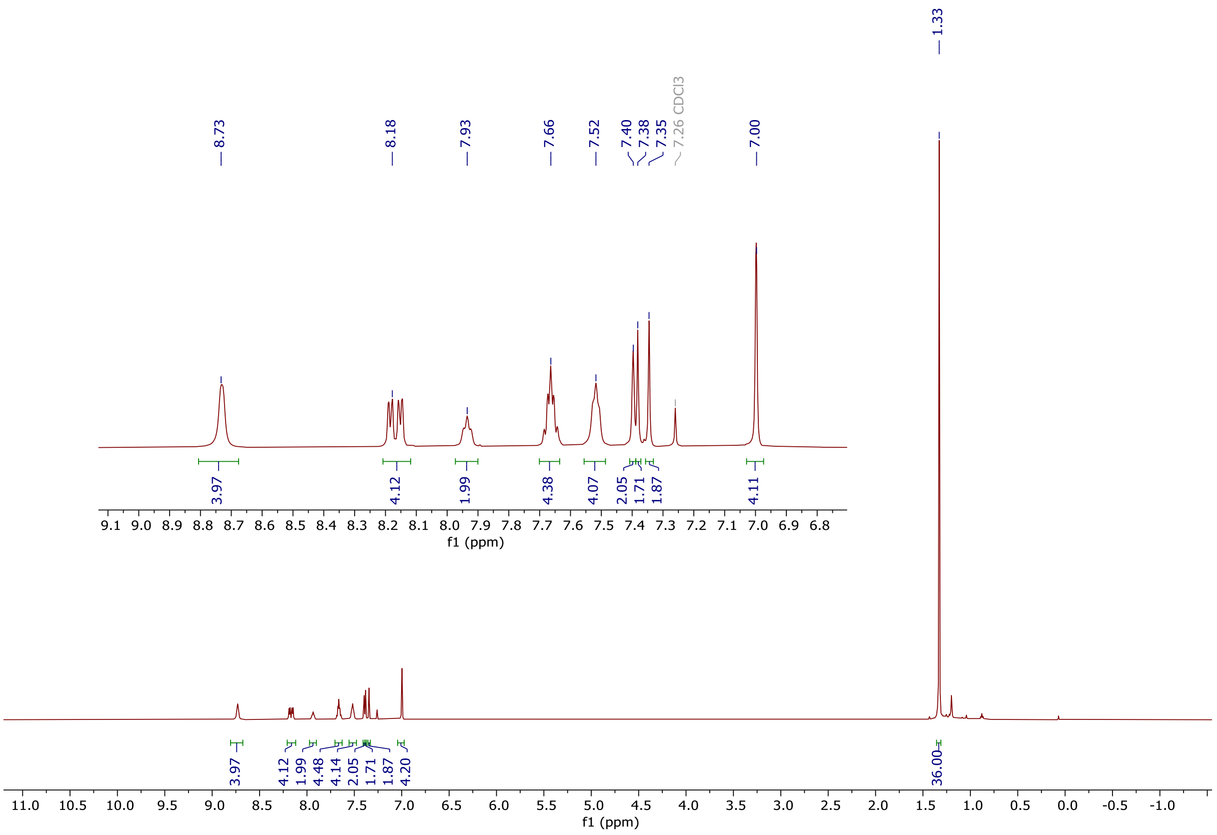

Figure S1.27. ^{1}H NMR spectrum (600 MHz, $CDCl_3$) of **1-(pyridine)$_2$**.

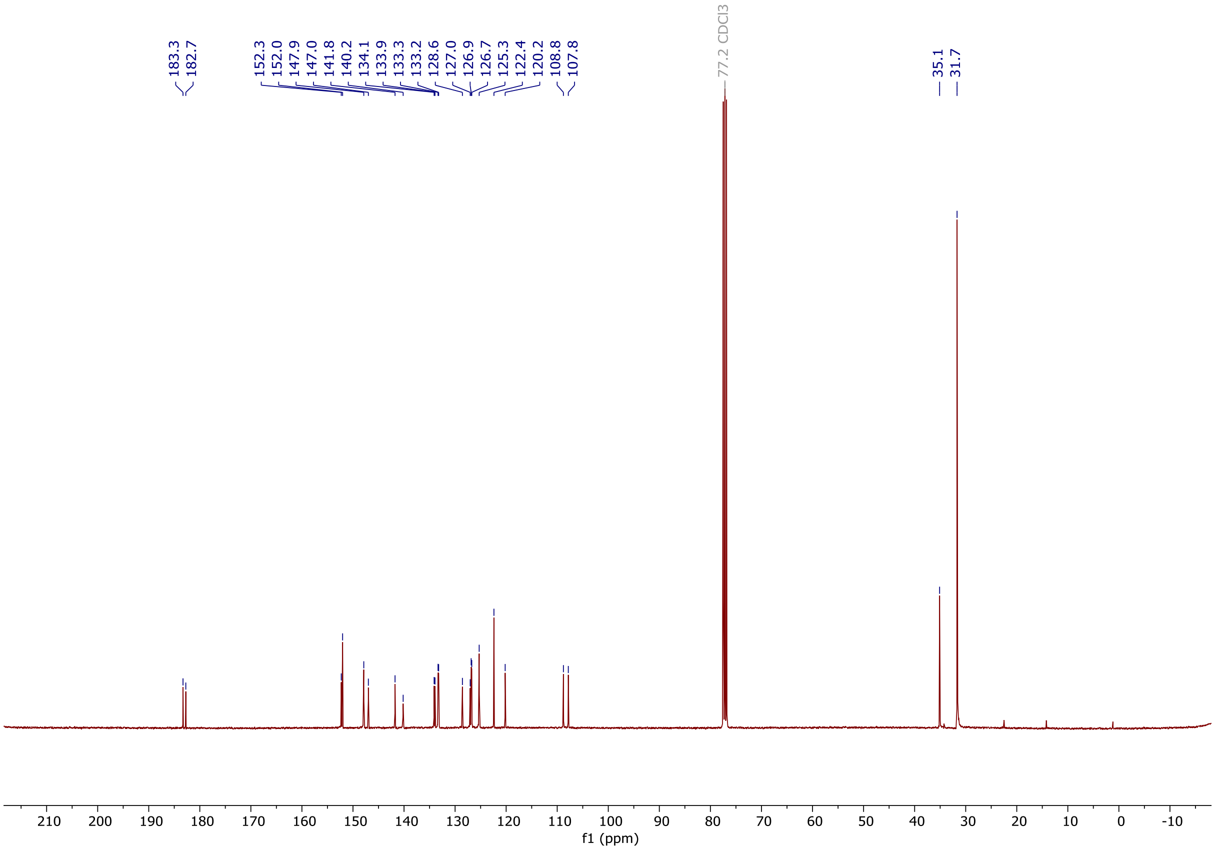

Figure S1.28. ^{13}C NMR spectrum (101 MHz, $CDCl_3$) of **1-(pyridine)$_2$**.

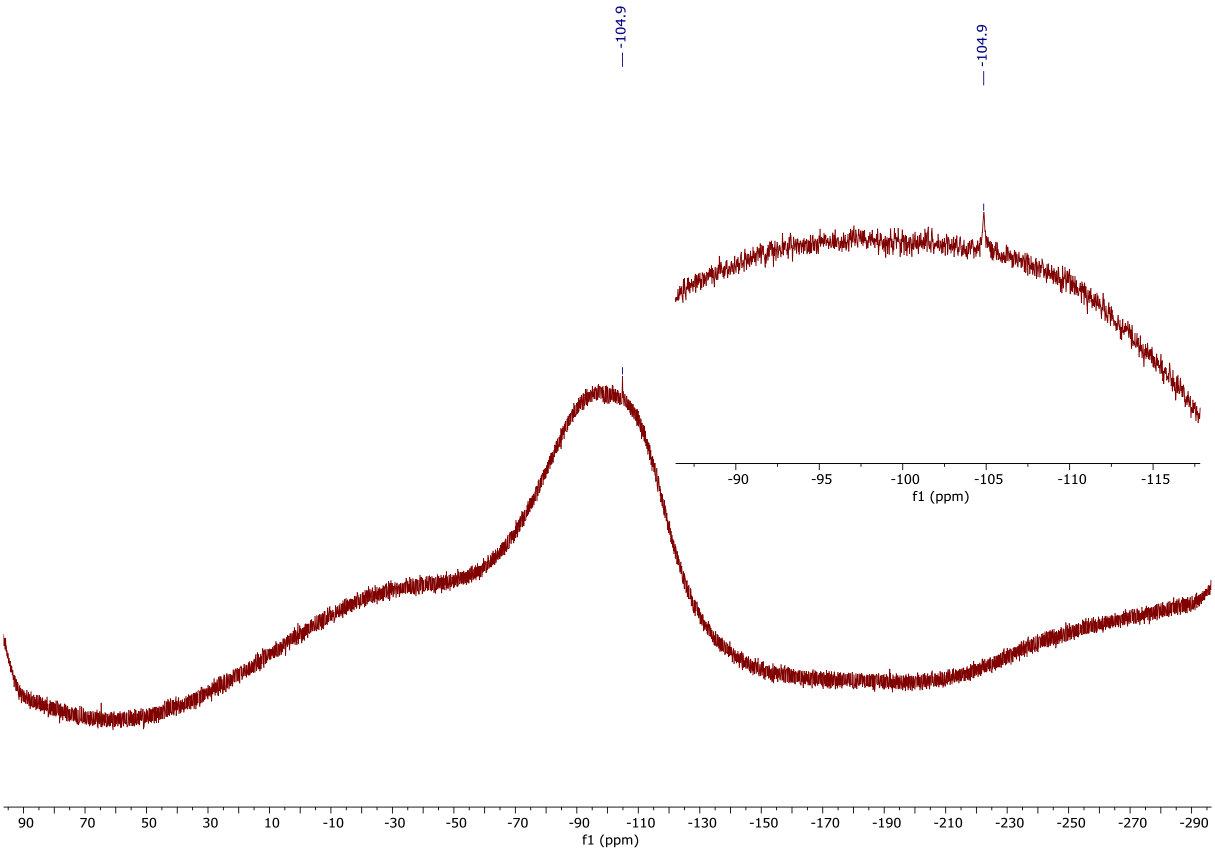

Figure S1.29. ^{29}Si NMR spectrum (80 MHz, $CDCl_3$) of **1-(pyridine)$_2$**.

1.11 1-DMAP

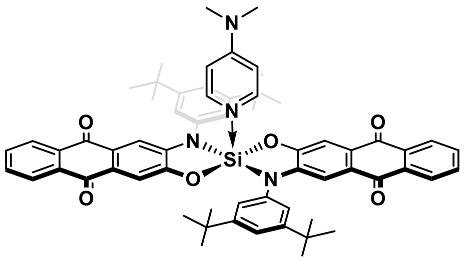

1 (40.0 mg, 45.5 µmol, 1.00 eq) was dissolved in benzene (2 ml). DMAP (5.4 mg, 44 µmol, 0.97 eq) was added, resulting in the immediate precipitation of an orange solid. After stirring for 15 minutes, the suspension was filtered and the solid washed with benzene (2x1 ml). The product was isolated as an orange powder after drying *in vacuo* overnight (36.4 mg, 36.4 µmol, 80 %).

^{1}H NMR (600 MHz, $CDCl_3$) δ 8.29 (d, *J* = 7.2 Hz, 2H), 8.22 – 8.16 (m, 2H), 8.16 – 8.13 (m, 2H), 7.70 – 7.61 (m, 4H), 7.39 – 7.37 (m, 2H), 7.31 (s, 2H), 7.25 (s, 2H), 6.99 (br s, 4H), 6.55 (d, *J* = 7.2 Hz, 2H), 3.14 (s, 6H), 1.34 (s, 36H).

^{13}C NMR (101 MHz, $CDCl_3$) δ 183.4, 182.9, 156.2, 152.9, 151.7, 147.8, 145.9, 142.5, 134.2, 134.1, 133.2, 133.0, 128.2, 126.8, 126.6, 122.8, 119.8, 108.2, 107.3, 106.6, 39.8, 35.1, 31.7.

Note: One less signal than expected is observed in the ^{13}C NMR spectrum. This is likely due to overlapping signals, with a shoulder peak next to the signal at 126.6 ppm.

^{29}Si NMR (119 MHz, CD_2Cl_2) δ -107.0.

MS (LIFDI+): *Due to the lability of binding DMAP, only the parent complex **1** was detected*. $[C_{56}H_{54}N_2O_6Si]^+$, calcd.: 878.38, found: 878.36.

UV-vis (DCM): λ_{max} (ε) = 466 nm (8100 $M^{-1}cm^{-1}$).

IR (ATR) [cm^{-1}] $\tilde{v}$ = 3073 (w, $\tilde{v}_{CH}$), 2963 (m, $\tilde{v}_{CH}$), 2869 (w, $\tilde{v}_{CH}$), 1660 (m, $\tilde{v}_{C=O}$).

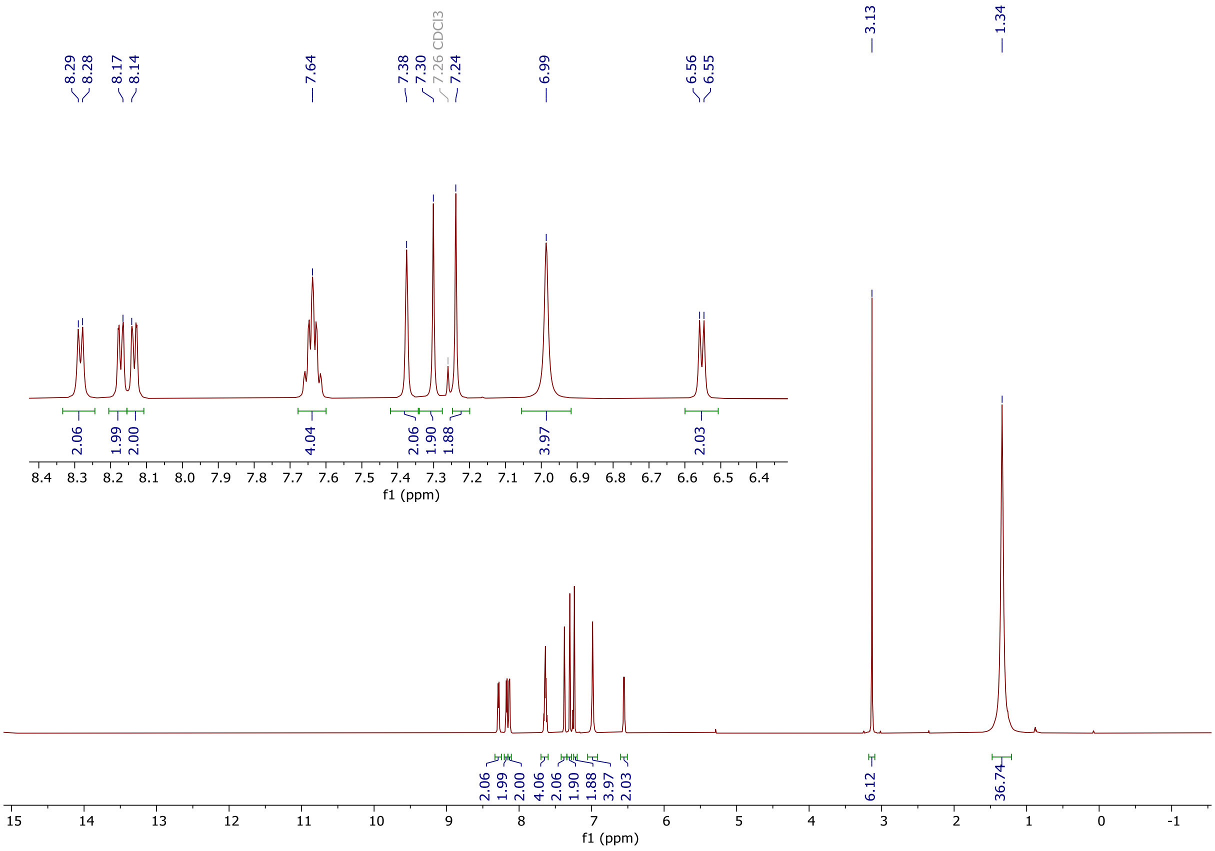

Figure S1.30. ^{1}H NMR spectrum (600 MHz, $CDCl_3$) of **1-DMAP**.

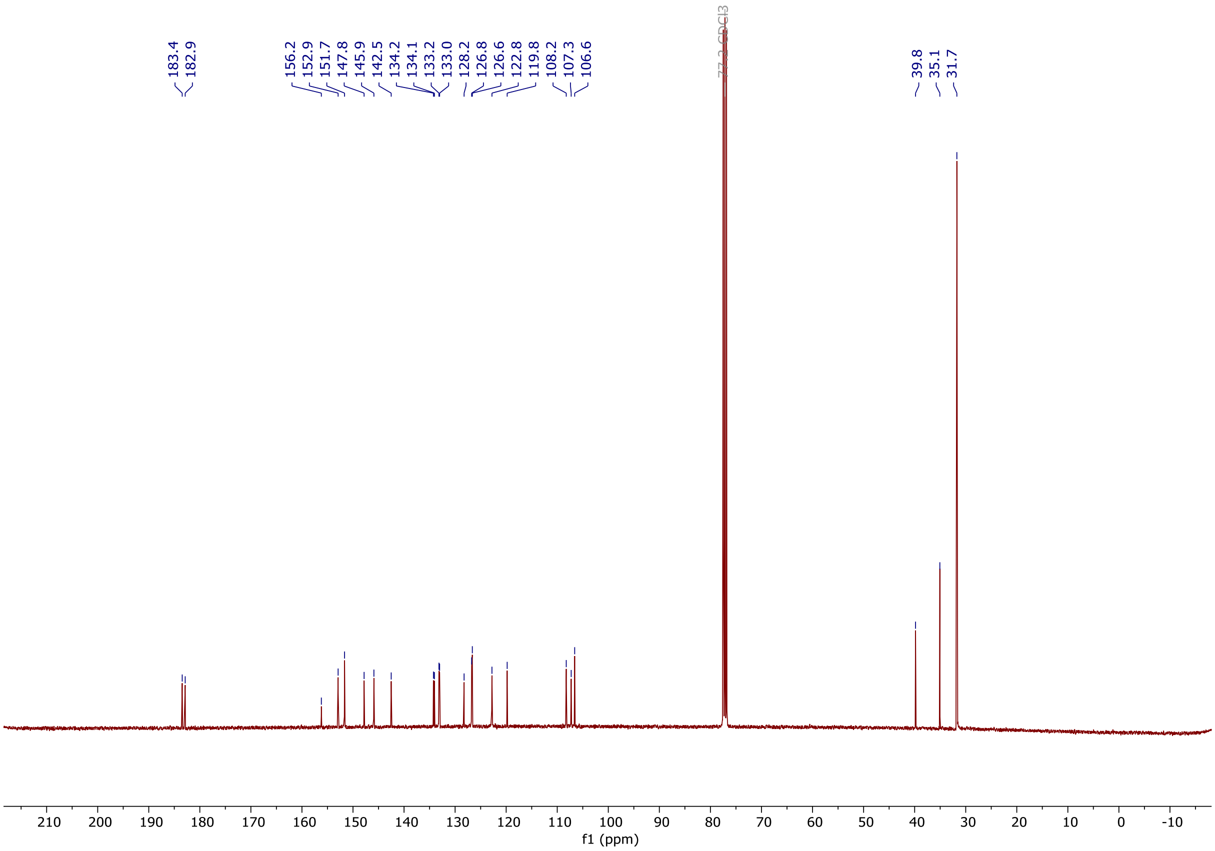

Figure S1.31. ^{13}C NMR spectrum (101 MHz, $CDCl_3$) of **1-DMAP**.

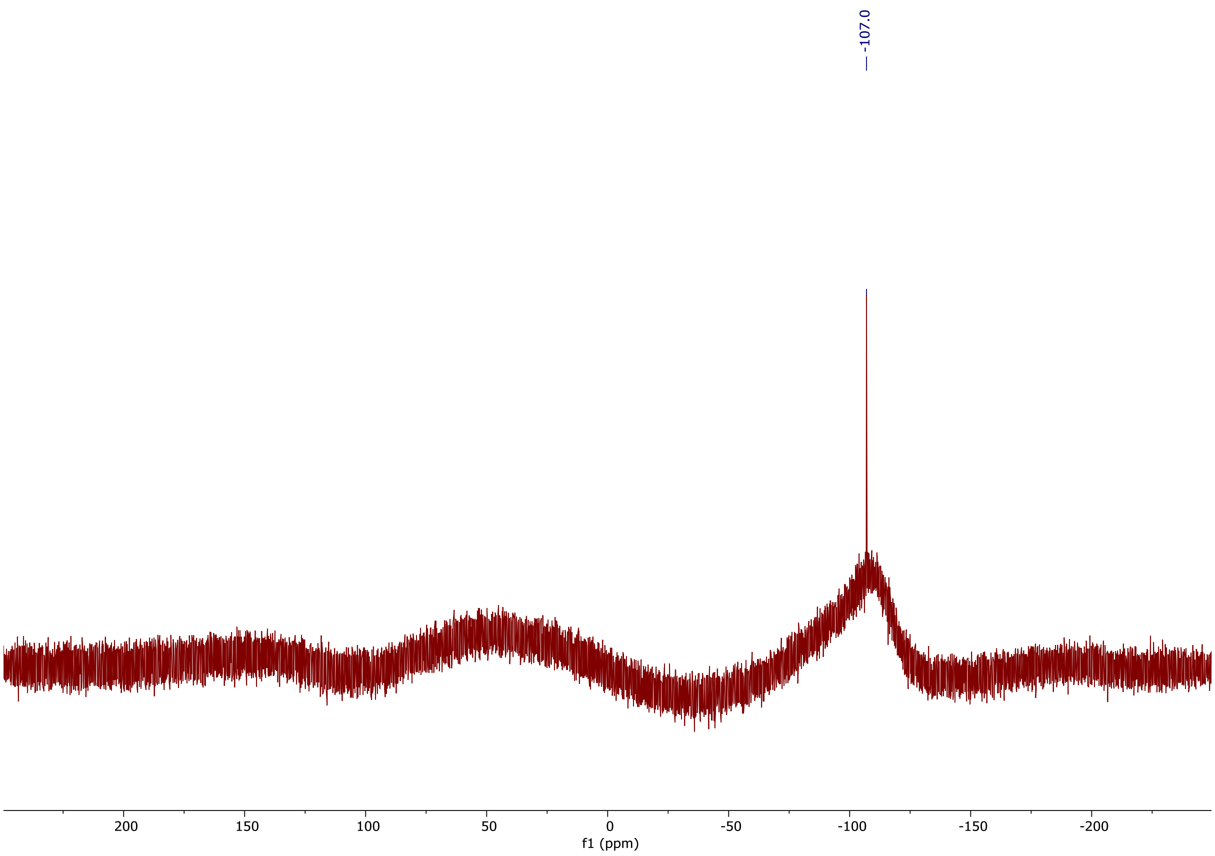

Figure S1.32. ^{29}Si NMR spectrum (119 MHz, CD_2Cl_2) of **1-DMAP**.

1.12 1-^{dipp}NHC

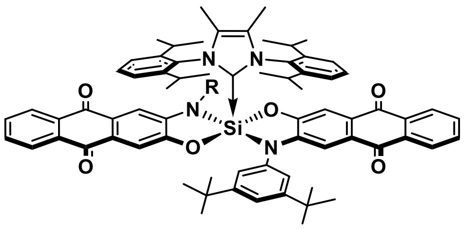

1 (40.0 mg, 45.5 µmol, 1.00 eq) was dissolved in DCM (2 ml). 4,5-Me_2-1,3-$(dipp)_2$-NHC (19.0 mg, 45.5 µmol, 1.00 eq) was added, resulting in a dark orange solution. Pentane (4 ml) was added, giving a clear solution, which was stored at –40 °C for 2 h. The precipitated solid was filtered off and washed with a 10:1 n-pentane/DCM mixture (3 x 1 ml). The product was isolated as an orange powder after drying in vacuo (43.2 mg, 33.3 µmol, 73 %).

^{1}H NMR (400 MHz, $CDCl_3$) δ 8.11 (m, 4H), 7.69 – 7.58 (m, 4H), 7.42 (d, *J* = 7.5 Hz, 2H), 7.20 (br, 2H), 7.13 (s, 2H), 6.82 (overlapping: br, 4H and s, 2H), 6.21 (overlapping: t, *J* = 1.9 Hz, 2H, and br, 2H), 3.06 (sept, *J* = 6.8 Hz, 2H), 2.39 (br sept, 2H), 1.78 (s, 6H), 1.37 (s, 18H), 1.20 – 1.04 (m, 18H), 0.93 (d, *J* = 5.5 Hz, 6H), 0.69 (s, 18H).

^{13}C NMR (101 MHz, $CDCl_3$) δ 183.5 (overlapping C=O and carbene-C), 182.4, 151.9, 150.7, 148.3, 142.3, 134.2, 134.1, 133.0, 132.9, 130.2, 127.5, 126.6, 126.5, 125.4, 122.8, 121.9, 121.1, 120.6, 107.7, 107.3, 35.0 (tBu-C), 34.3 (tBu-C), 31.9 (tBu-CH_3), 30.8 (tBu-CH_3), 29.1 (iPr-CH), 28.9 ((iPr-CH), 24.4 (iPr-CH_3), 23.9 (iPr-CH_3), 11.1 (carbene-CH_3).

Note: Binding of the NHC induces asymmetry (two sets of tBu/iPr groups), which, together with line broadening in the ^{13}C NMR spectrum, did not allow to account for all carbon atoms. Characteristic carbon shifts were assigned by HSQC and HMBC experiments and are denoted above.

^{29}Si NMR (119 MHz, $CDCl_3$) δ *No signal observed due to limited signal intensity and solubility.*

MS (LIFDI+): $[C_{85}H_{94}N_4O_6Si]^+$, calcd.: 1294.69, found: 1294.65.

UV-vis (DCM): λ_{max} (ε) = 475 nm (5700 $M^{-1}cm^{-1}$).

IR (ATR) [cm^{-1}] $\tilde{\nu}$ = 3072 (w, $\tilde{\nu}_{CH}$), 2964 (m, $\tilde{\nu}_{CH}$), 2870 (w, $\tilde{\nu}_{CH}$), 1664 (m, $\tilde{\nu}_{C=O}$), 1652 (w, $\tilde{\nu}_{C=O}$).

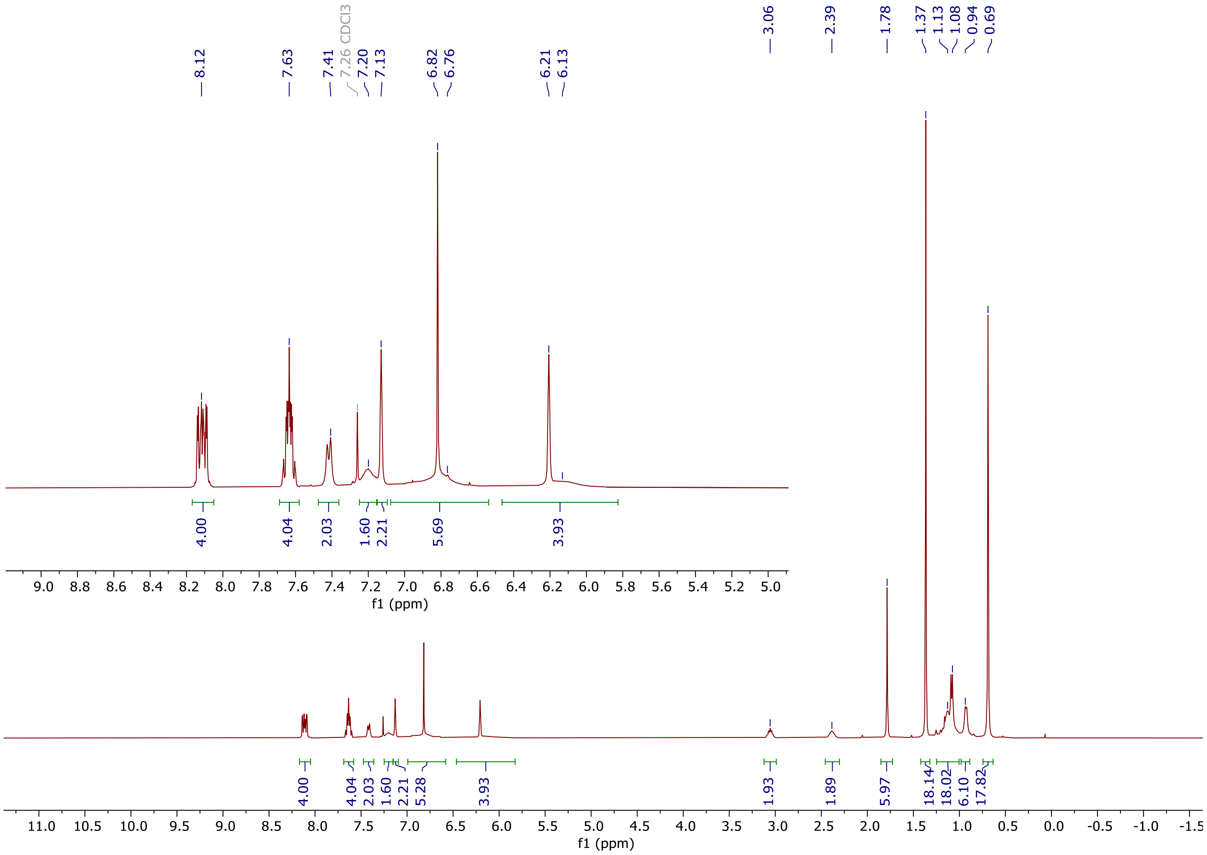

Figure S1.33. ^{1}H NMR spectrum (400 MHz, $CDCl_3$) of **1-^{dipp}NHC**.

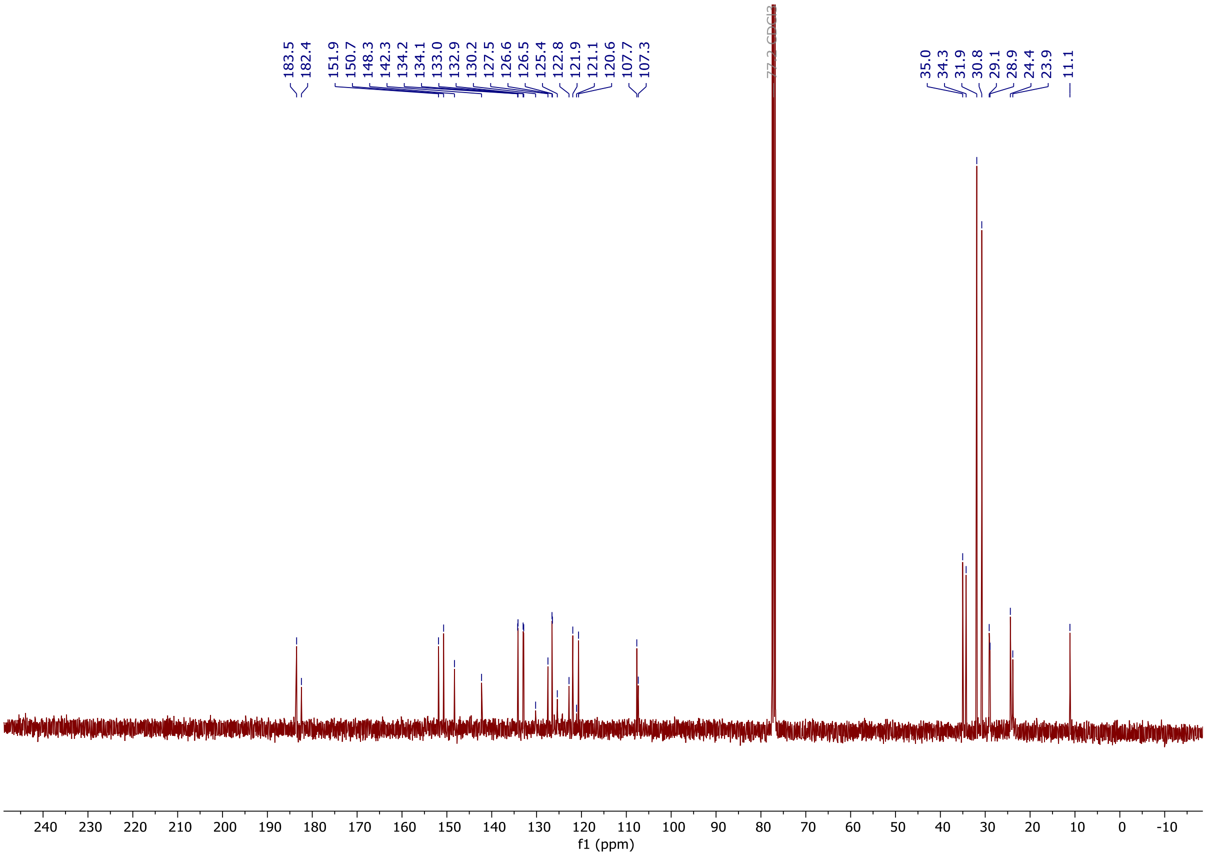

Figure S1.34. ^{13}C NMR spectrum (101 MHz, $CDCl_3$) of **1-^{dipp}NHC**.

1.13 1-DMSO

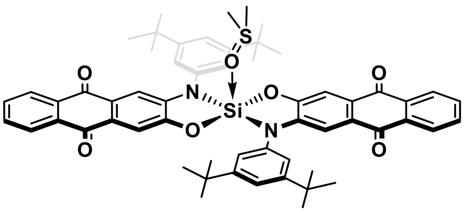

1 (40.0 mg, 45.5 µmol, 1.00 eq) was dissolved in DCM (1 ml). DMSO (4.9 µl, 68 µmol, 1.5 eq) was added, resulting in an orange solution. The solution was layered with *n*-hexane (4 ml) and stored at –40 °C overnight, resulting in the precipitation of an orange microcrystalline solid. The solid was filtered off, washed with a 4:1 *n*-hexane/DCM mixture (2x1 ml) and dried *in vacuo* (33.1 mg, 34.6 µmol, 76 %).

^{1}H NMR (400 MHz, $CDCl_3$) δ 8.26 – 8.14 (m, 4H), 7.75 – 7.63 (m, 4H), 7.46 (s, 2H), 7.44 (s, 2H), 7.31 (t, *J* = 2.1 Hz, 2H), 6.93 (br s, 4H), 2.96 (s, 6H), 1.23 (s, 36H).

^{13}C NMR (101 MHz, $CDCl_3$) δ 183.2, 182.6, 152.2, 151.8, 146.1, 140.1, 134.1, 133.9, 133.5, 133.4, 128.9, 127.3, 126.9, 126.9, 121.3, 120.5, 109.3, 107.9, 37.8* (br, DMSO), 35.0, 31.5.

**This signal was identified as the DMSO shift by a ^{1}H-^{13}C HSQC experiment.*

^{29}Si NMR (79 MHz, $CDCl_3$) *No signal observed due to limited solubility and signal intensity*

MS (LIFDI+): *Due to the lability of binding DMSO, only the parent complex **1** was detected*. $[C_{56}H_{54}N_2O_6Si]^+$, calcd.: 878.38, found: 878.38.

UV-vis (DCM): λ_{max} (ε) = 460 nm (8500 $M^{-1}cm^{-1}$).

IR (ATR) [cm^{-1}] $\tilde{\nu}$ = 3076 (w, $\tilde{\nu}_{CH}$), 3013 (w, $\tilde{\nu}_{CH}$), 2954 (w, $\tilde{\nu}_{CH}$), 2928 (w, $\tilde{\nu}_{CH}$), 2904 (w, $\tilde{\nu}_{CH}$), 2865 (w, $\tilde{\nu}_{CH}$), 1661 (m, $\tilde{\nu}_{C=O}$).

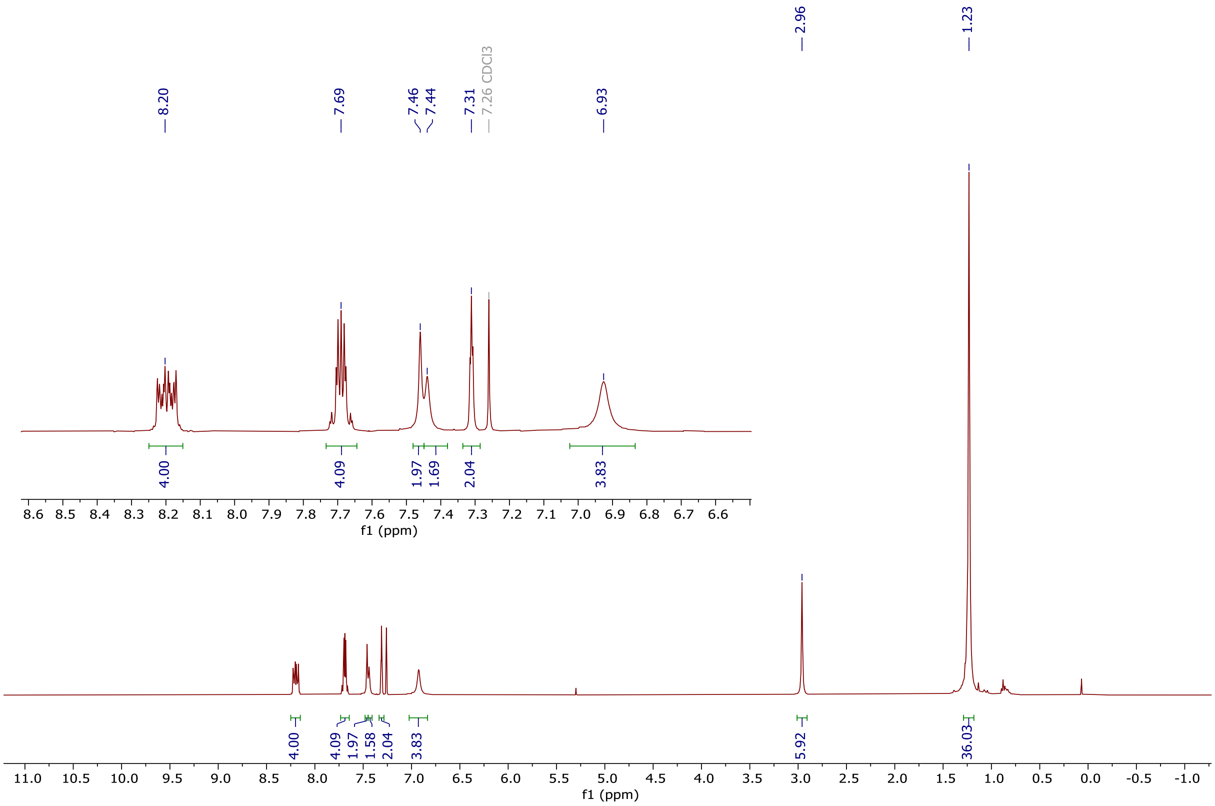

Figure S1.35. ^{1}H NMR spectrum (400 MHz, $CDCl_3$) of **1-DMSO**.

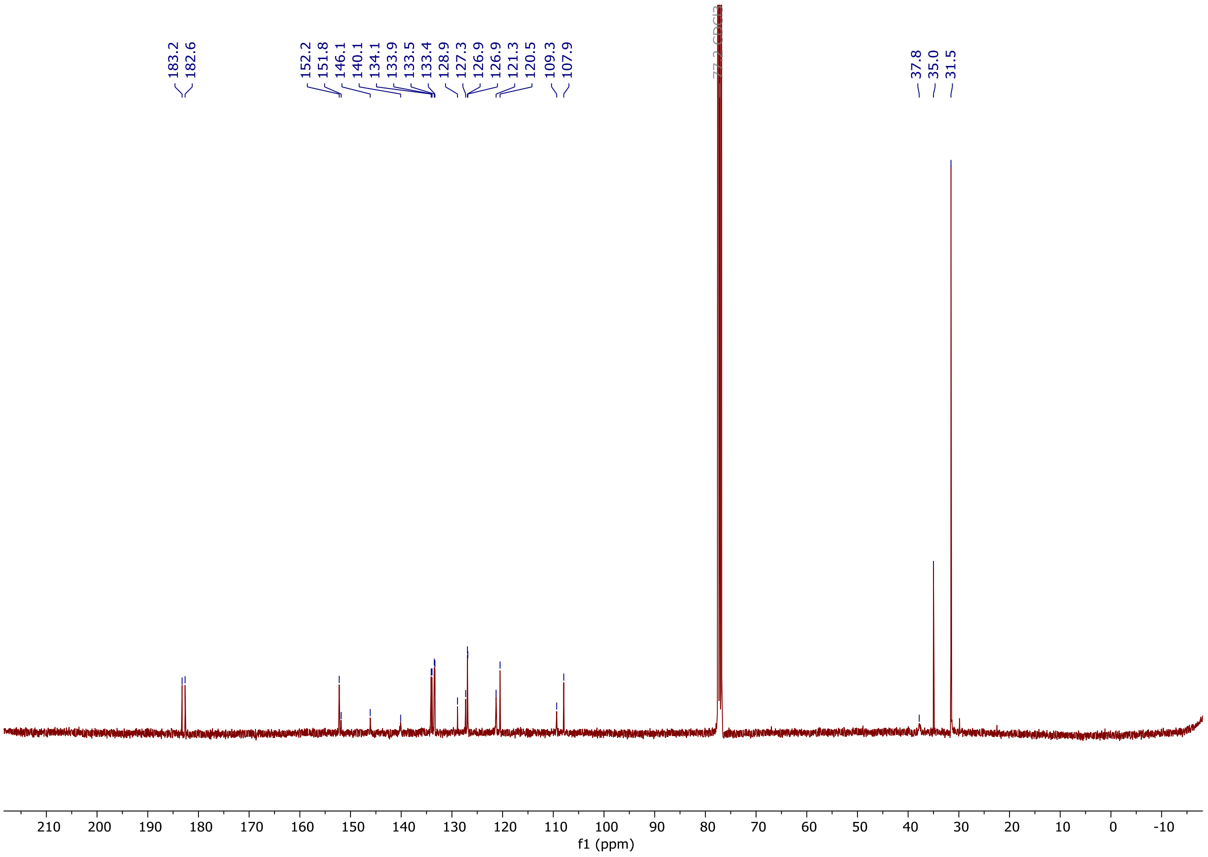

Figure S1.36. ^{13}C NMR spectrum (101 MHz, $CDCl_3$) of **1-DMSO**.

1.14 1-OPEt$_3$

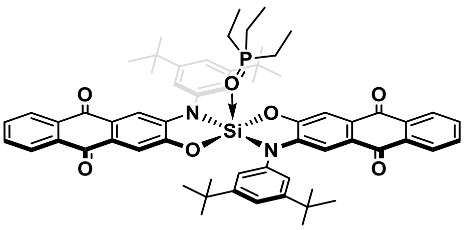

1 (40.0 mg, 45.5 µmol, 1.00 eq) was dissolved in DCM (1 ml). Et_3PO (5.1 mg, 46 µmol, 1.0 eq) was added, resulting in an orange solution. The solution was layered with *n*-hexane (4 ml) and stored at –40 °C overnight, resulting in the precipitation of an orange solid. The solid was filtered off, washed with a 2:1 *n*-hexane/DCM mixture (2x1 ml) and dried *in vacuo* (33.1 mg, 34.6 µmol, 76 %).

^{1}H NMR (400 MHz, $CDCl_3$) δ 8.22 – 8.12 (m, 4H), 7.71 – 7.60 (m, 4H), 7.30 (t, *J* = 1.8 Hz, 2H), 7.28 (s, 2H), 7.19 (s, 2H), 2.18 – 1.95 (m, 6H), 1.19 (overlapping: dt, *J* = 18.6, 7.7 Hz, 12H; br s, 36H; combined 48H).

^{13}C NMR (101 MHz, $CDCl_3$) δ 183.5, 182.9, 153.1, 151.5, 147.2, 142.0, 134.2, 134.1, 133.2, 133.1, 127.9, 126.9, 126.8, 126.6, 122.4, 120.1, 107.8, 107.2, 34.9, 31.6, 17.4 (d, *J* = 64.6 Hz), 5.4 (d, *J* = 4.7 Hz).

^{31}P NMR (162 MHz, $CDCl_3$) δ 80.8.

^{31}P NMR (162 MHz, CD_2Cl_2) δ 81.6.

Note: The ^{31}P NMR shift in DCM-d_2 is given for the evaluation of Lewis acidity by the Gutmann-Beckett method.

^{29}Si NMR (80 MHz, $CDCl_3$) δ -108.1.

MS (LIFDI+): *Due to the lability of binding $OPEt_3$, only the parent complex **1** was detected*. $[C_{56}H_{54}N_2O_6Si]^+$, calcd.: 878.38, found: 878.39.

UV-vis (DCM): λ_{max} (ε) = 469 nm (9300 $M^{-1}cm^{-1}$).

IR (ATR) [cm^{-1}] $\tilde{\nu}$ = 3066 (w, $\tilde{\nu}_{CH}$), 2959 (m, $\tilde{\nu}_{CH}$), 2906 (w, $\tilde{\nu}_{CH}$), 2867 (w, $\tilde{\nu}_{CH}$), 1663 (m, $\tilde{\nu}_{C=O}$).

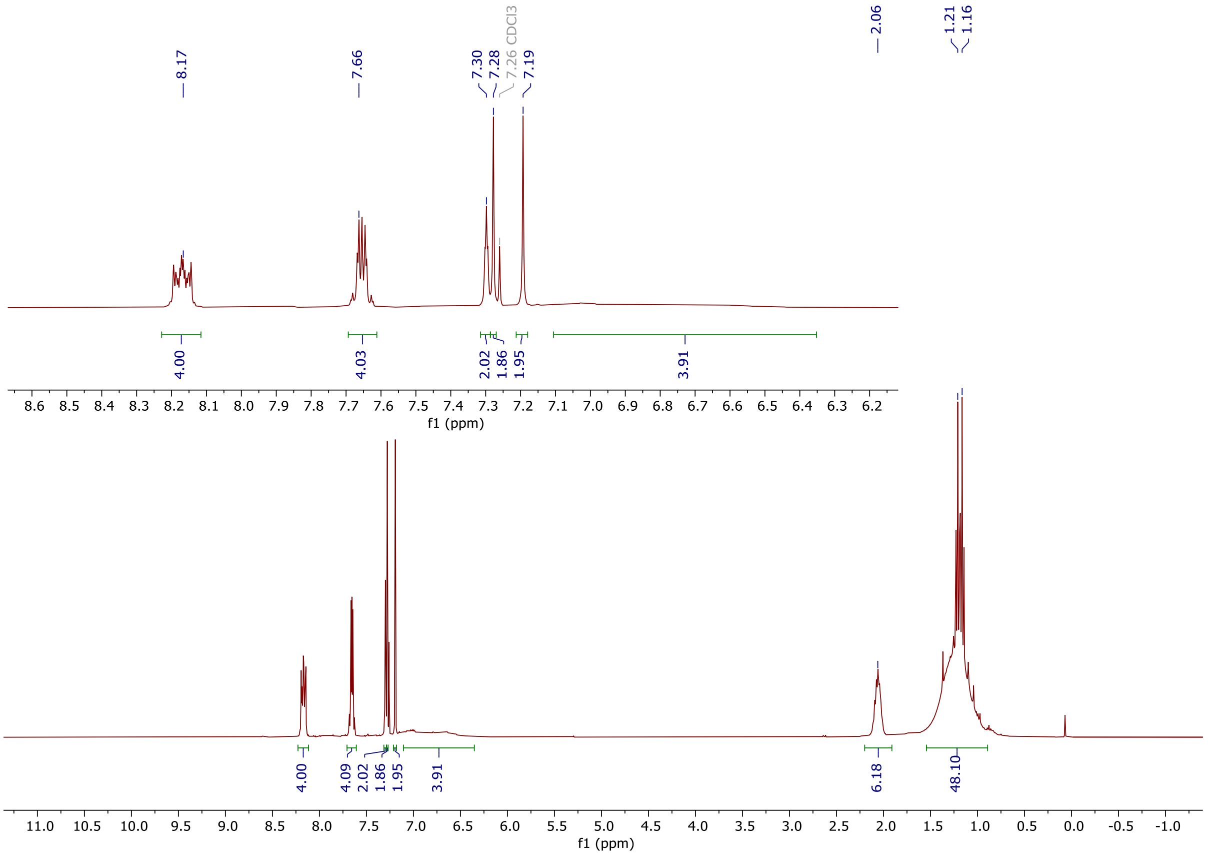

Figure S1.37. ^{1}H NMR spectrum (400 MHz, $CDCl_3$) of **1-OPEt₃**.

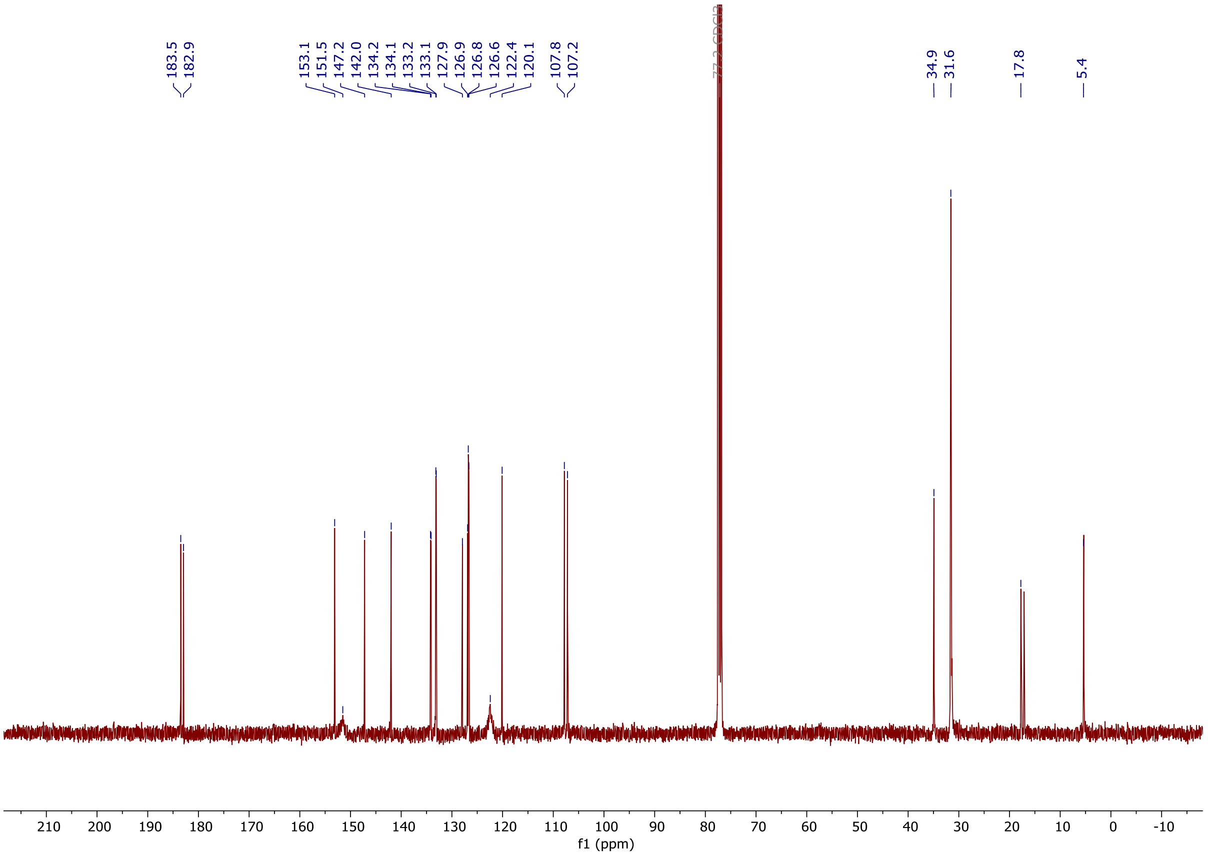

Figure S1.38. ^{13}C NMR spectrum (101 MHz, $CDCl_3$) of **1-OPEt₃.**

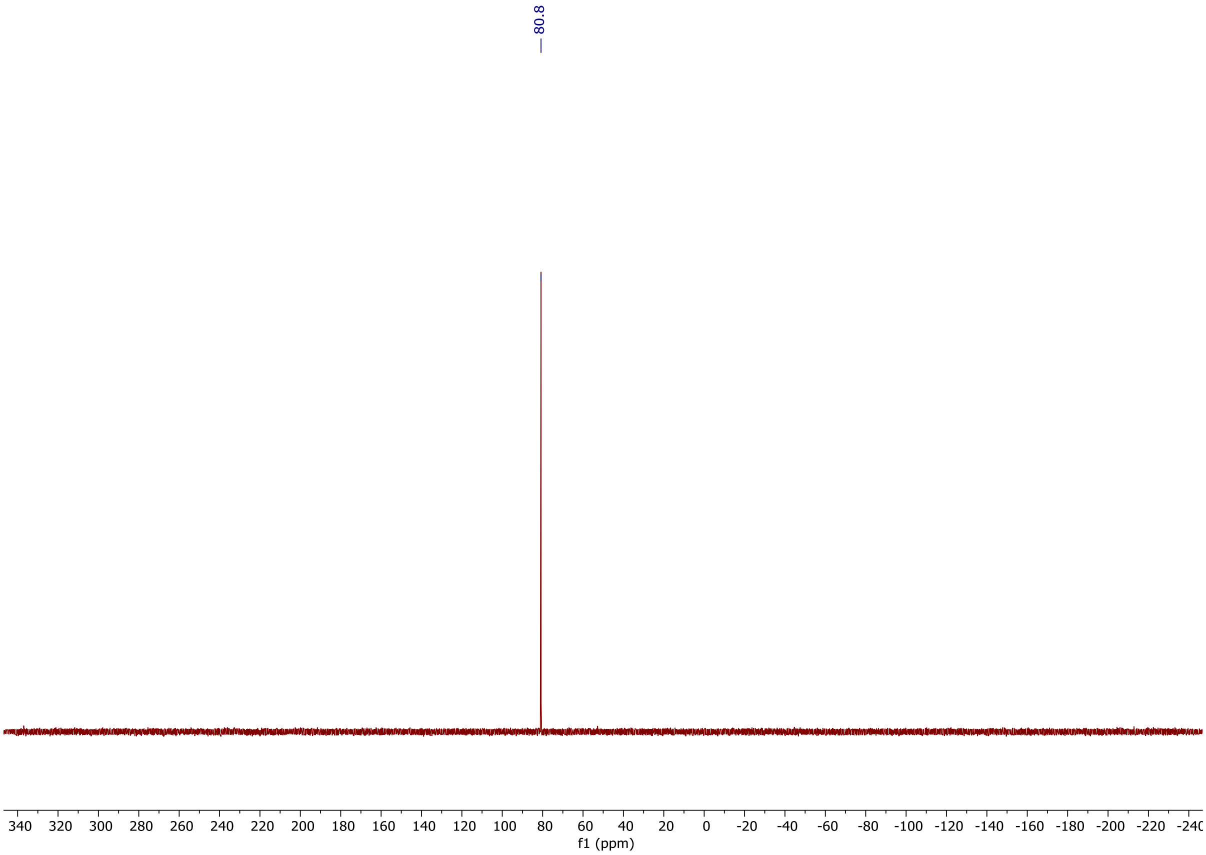

Figure S1.39. ^{31}P NMR spectrum (162 MHz, $CDCl_3$) of **1-OPEt$_3$.**

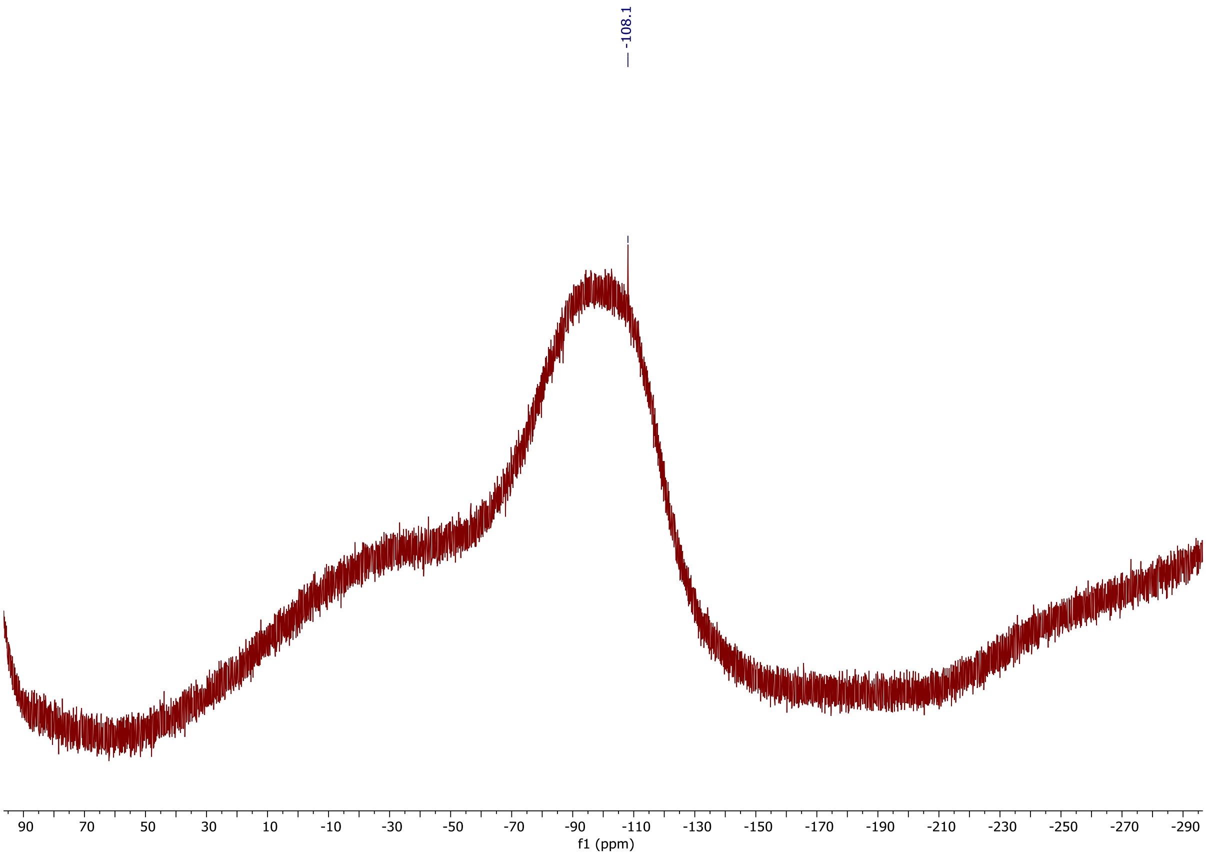

Figure S1.40. ^{29}Si NMR spectrum (80 MHz, $CDCl_3$) of **1-OPEt$_3$.**

1.15 1-DIBA

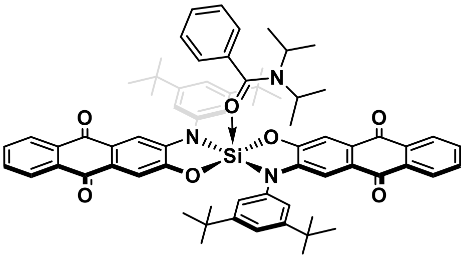

1 (40.0 mg, 45.5 µmol, 1.00 eq) was dissolved in DCM (1 ml). DIBA (9.3 mg, 46 µmol, 1.0 eq) was added, resulting in an orange solution. *n*-hexane (4 ml) was added and the mixture stored at –40 °C for 2h, resulting in the precipitation of an orange microcrystalline solid. The solid was filtered off, washed with a 10:1 *n*-hexane/DCM mixture (4x1 ml) and dried *in vacuo* (34.9 mg, 34.6 µmol, 71 %).

^{1}H NMR (600 MHz, $CDCl_3$) δ 8.25 – 8.15 (m, 4H), 7.72 – 7.65 (m, 4H), 7.36 (br, 7H), 7.25 (m, 4H), 6.80 (br s, 4H), 4.03 (br s, 1H, *iPr-H*), 3.74 (br s, 1H, *iPr-H*), 1.59 (br s, 6H, *iPr-CH$_3$*), 1.33 – 1.10 (m, 42H, *overlapping tBu/iPr-CH$_3$*).

^{13}C NMR (101 MHz, $CDCl_3$) δ 183.3, 182.8, 171.8 (*amide C=O*), 152.5, 151.9, 145.7, 140.2, 134.2, 134.0, 133.4, 133.2, 130.6, 128.7, 128.3, 127.1, 126.9, 126.8, 126.6, 121.1, 120.2, 109.3, 107.6, 53.6, 49.0, 34.9, 31.5, 20.7.

Note: Signals at 53.6 ppm and 49.0 ppm were assigned by a ^{1}H-^{13}C HSQC experiment.

^{29}Si NMR (80 MHz, $CDCl_3$) *No signal observed due to limited solubility and signal intensity*

MS (LIFDI+): *Due to the lability of binding DIBA, only the parent complex **1** was detected*. $[C_{56}H_{54}N_2O_6Si]^+$, calcd.: 878.38, found: 878.39. *The mass of DIBA was also detected.* $[C_{13}H_{19}NO]^+$, calcd.: 205.15, found: 205.15.

UV-vis (DCM): λ_{max} (ε) = 467 nm (7500 $M^{-1}cm^{-1}$).

IR (ATR) [cm^{-1}] $\tilde{\nu}$ = 3067 (w, $\tilde{\nu}_{CH}$), 2953 (m, $\tilde{\nu}_{CH}$), 2866 (w, $\tilde{\nu}_{CH}$), 1661 (m, $\tilde{\nu}_{C=O}$).

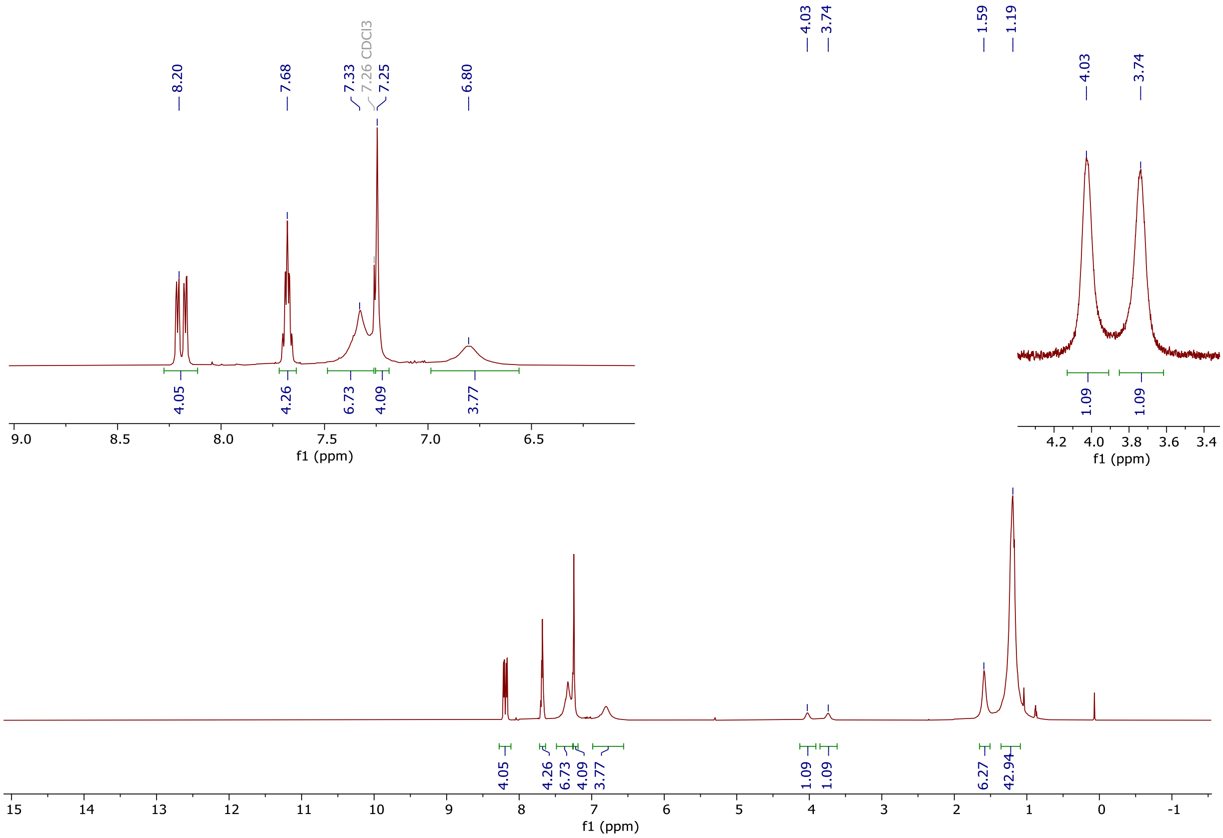

Figure S1.41. ^{1}H NMR spectrum (600 MHz, $CDCl_3$) of **1-DIBA.**

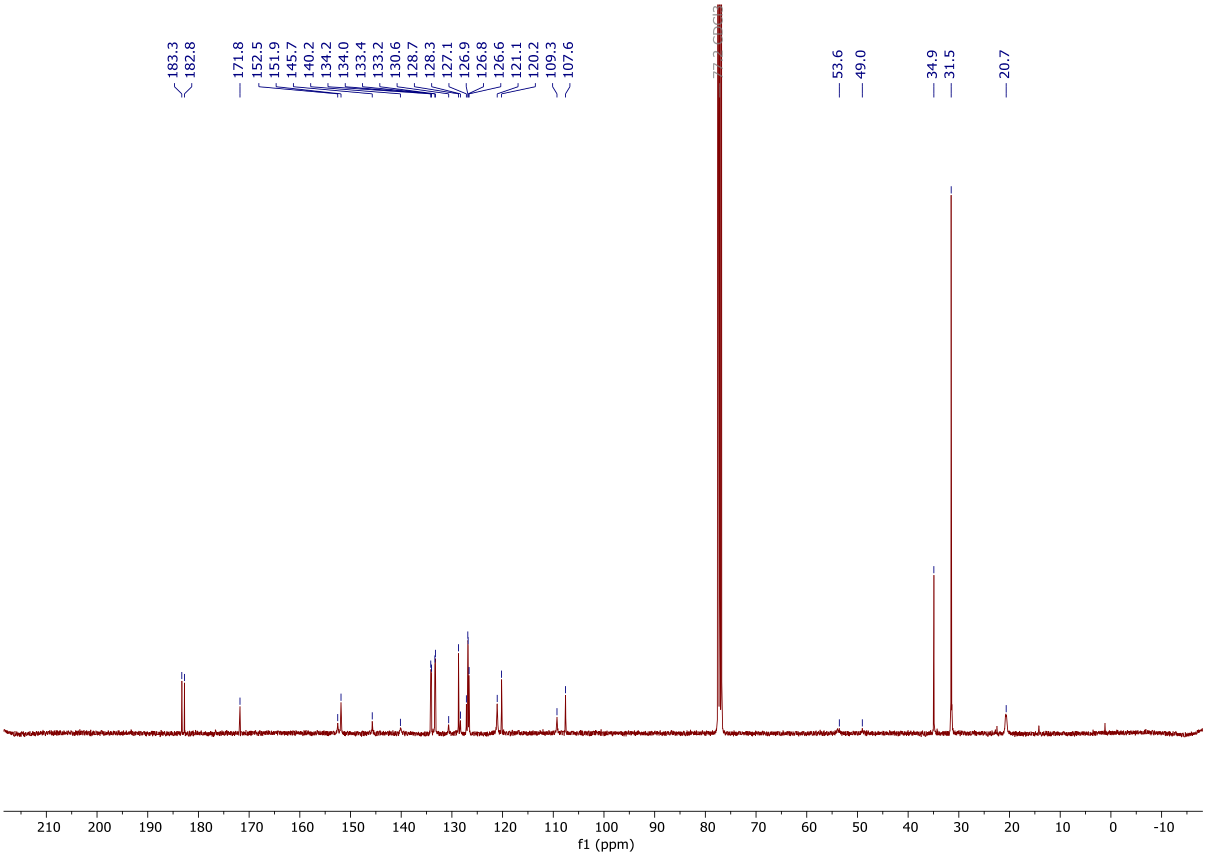

Figure S1.42. ^{13}C NMR spectrum (101 MHz, $CDCl_3$) of **1-DIBA.**

1.16 1-HMPA

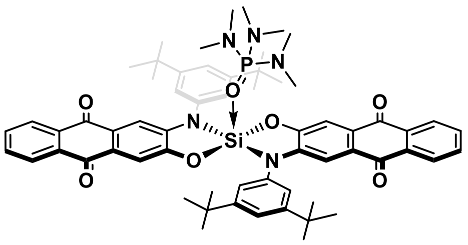

1 (40.0 mg, 45.5 µmol, 1.00 eq) was dissolved in DCM (1 ml). HMPA (9.8 mg, 55 µmol, 1.2 eq) was added, resulting in an orange solution. The solution was layered with *n*-pentane (4 ml) and stored at –40 °C for 1 h, resulting in the precipitation of an orange solid. The solid was filtered off, washed with a 2:1 *n*-pentane/DCM mixture (2x1 ml) and dried *in vacuo* (33.1 mg, 31.2 µmol, 69 %).

^{1}H NMR (400 MHz, $CDCl_3$) δ 8.22 – 8.09 (m, 4H), 7.69 – 7.59 (m, 4H), 7.29 (t, *J* = 1.8 Hz, 2H), 7.27 (s, 2H), 7.16 (s, 2H), 6.97 (br, 4H), 2.63 (d, *J* = 10.3 Hz, 18H), 1.26 (br s, 36H).

^{13}C NMR (101 MHz, $CDCl_3$) δ 183.5, 183.1, 153.8, 151.2, 147.5, 142.4, 134.3, 134.2, 133.1, 133.0, 127.5, 126.8, 126.7, 126.6, 122.7, 119.7, 107.6, 106.9, 36.9 (d, *J* = 5.1 Hz), 34.9, 31.6.

^{31}P NMR (162 MHz, $CDCl_3$) δ 22.1.

^{29}Si NMR (79 MHz, $CDCl_3$) δ -111.7.

MS (LIFDI+): *Due to the instability of **1-HMPA** under mass spectrometry conditions, only the parent complex **1** was detected.* $[C_{56}H_{54}N_2O_6Si]^+$, calcd.: 878.38, found: 878.34.

UV-vis (DCM): λ_{max} (ε) = 474 nm (8200 $M^{-1}cm^{-1}$).

IR (ATR) [cm^{-1}] $\tilde{\nu}$ = 3066 (w, $\tilde{\nu}_{CH}$), 2956 (m, $\tilde{\nu}_{CH}$), 2905 (w, $\tilde{\nu}_{CH}$), 2865 (w, $\tilde{\nu}_{CH}$), 2824 (w, $\tilde{\nu}_{CH}$), 1663 (m, $\tilde{\nu}_{C=O}$).

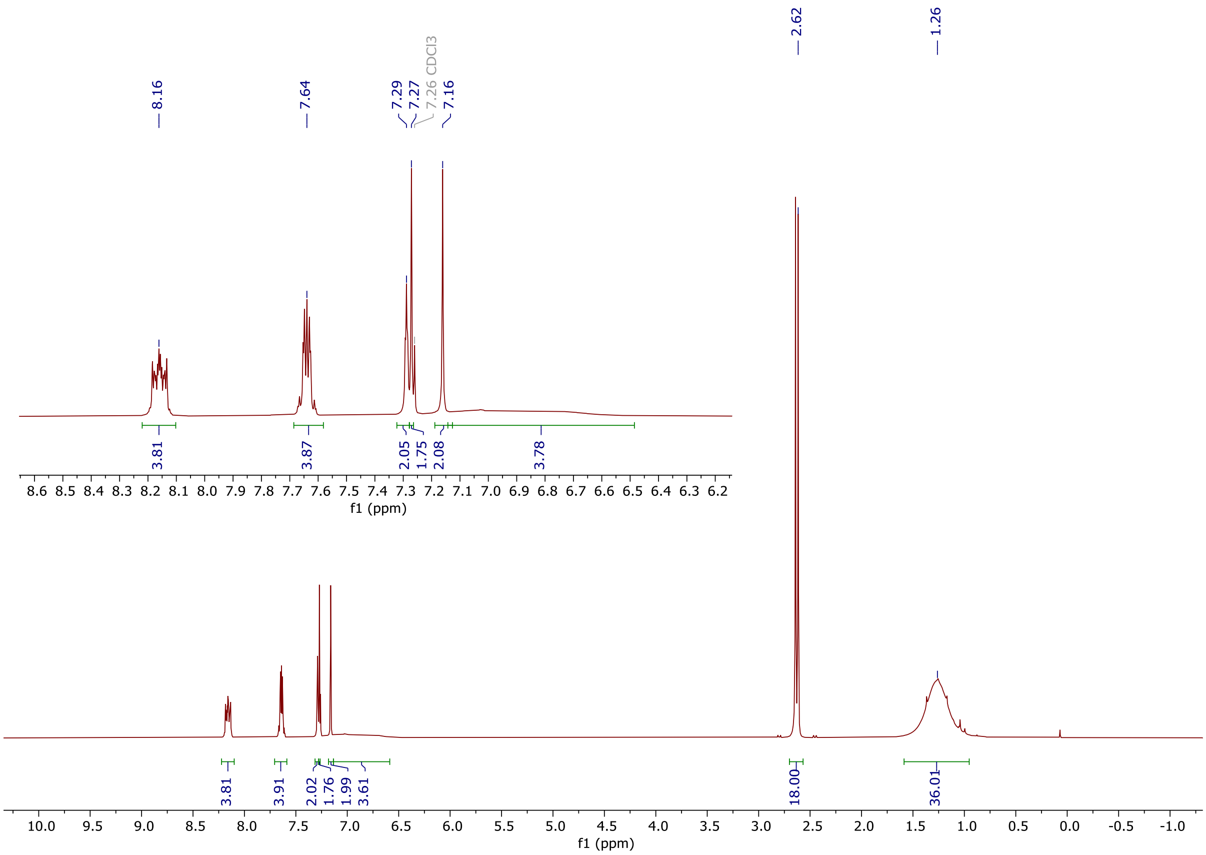

Figure S1.43. ^{1}H NMR spectrum (400 MHz, $CDCl_3$) of **1-HMPA**.

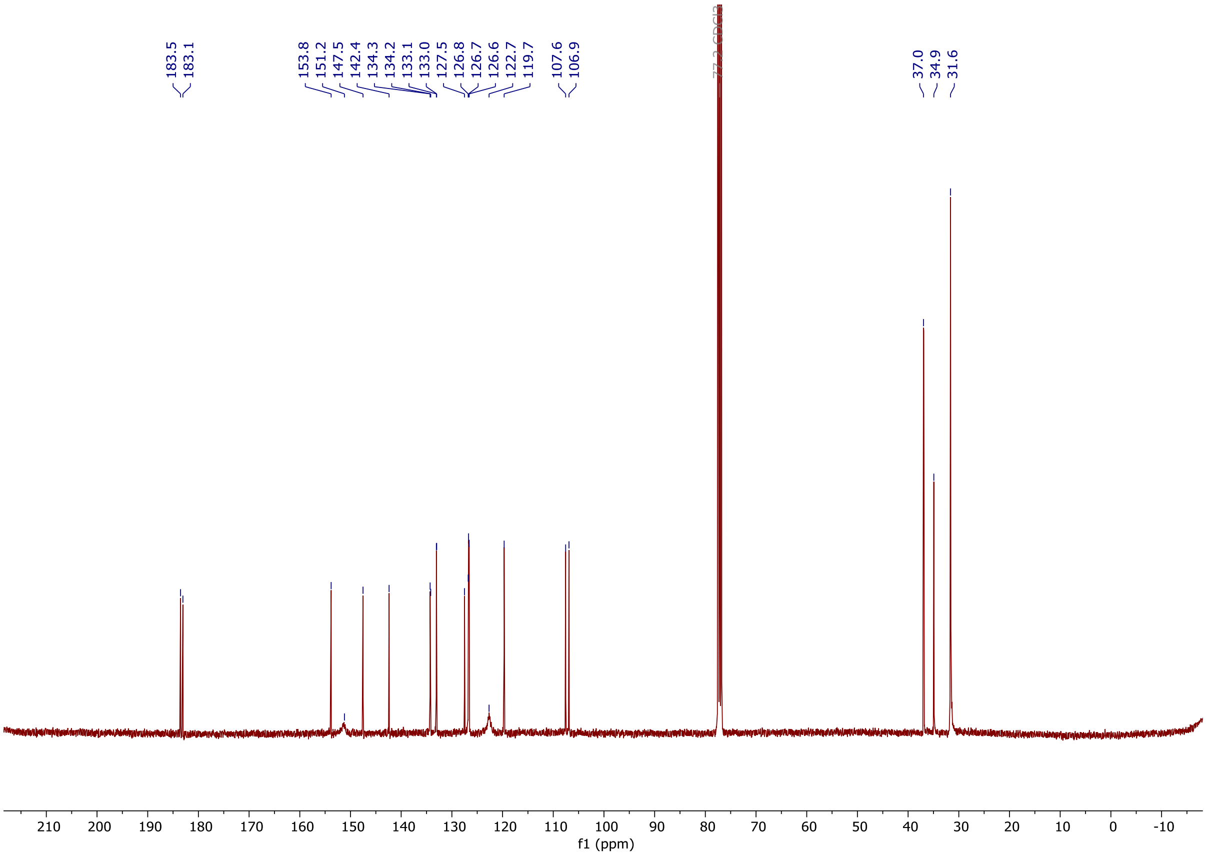

Figure S1.44. ^{13}C NMR spectrum (101 MHz, $CDCl_3$) of **1-HMPA**.

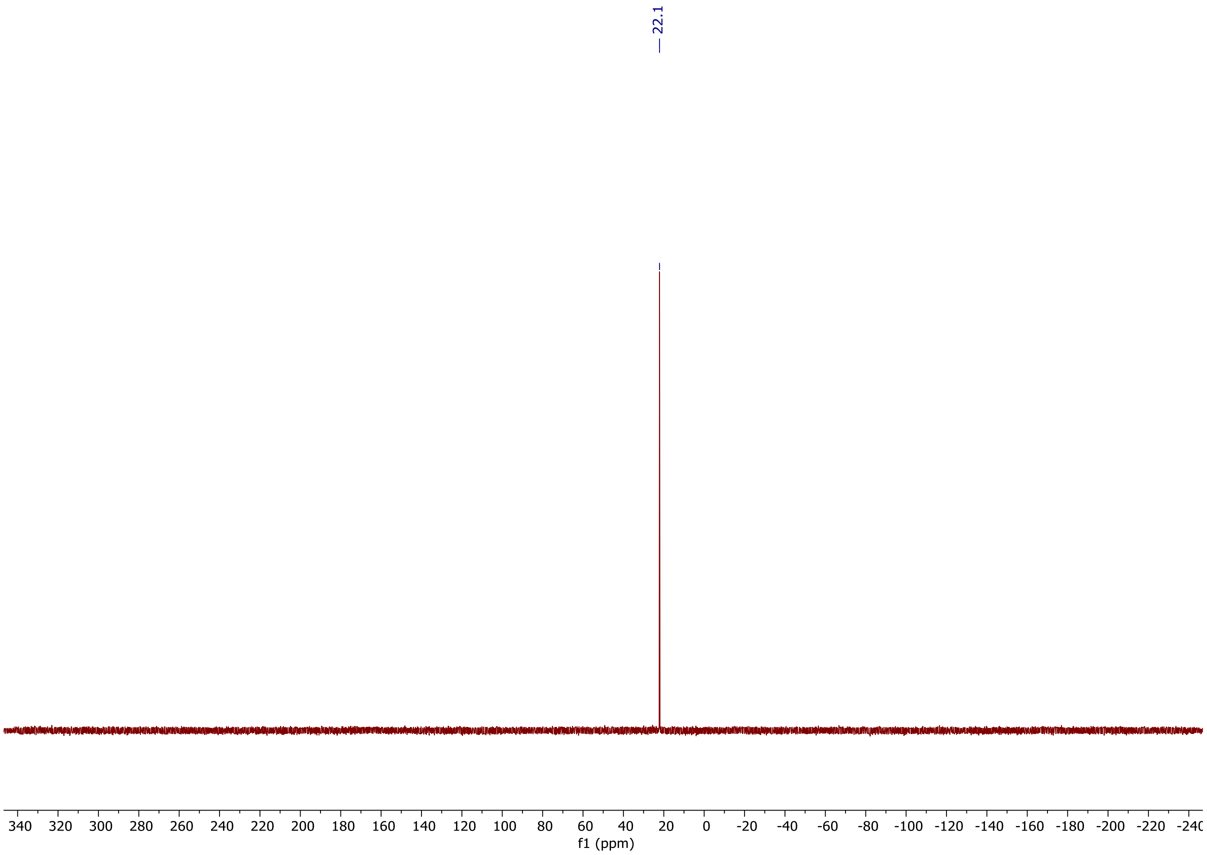

Figure S1.45. ^{31}P NMR spectrum (162 MHz, $CDCl_3$) of **1-HMPA**.

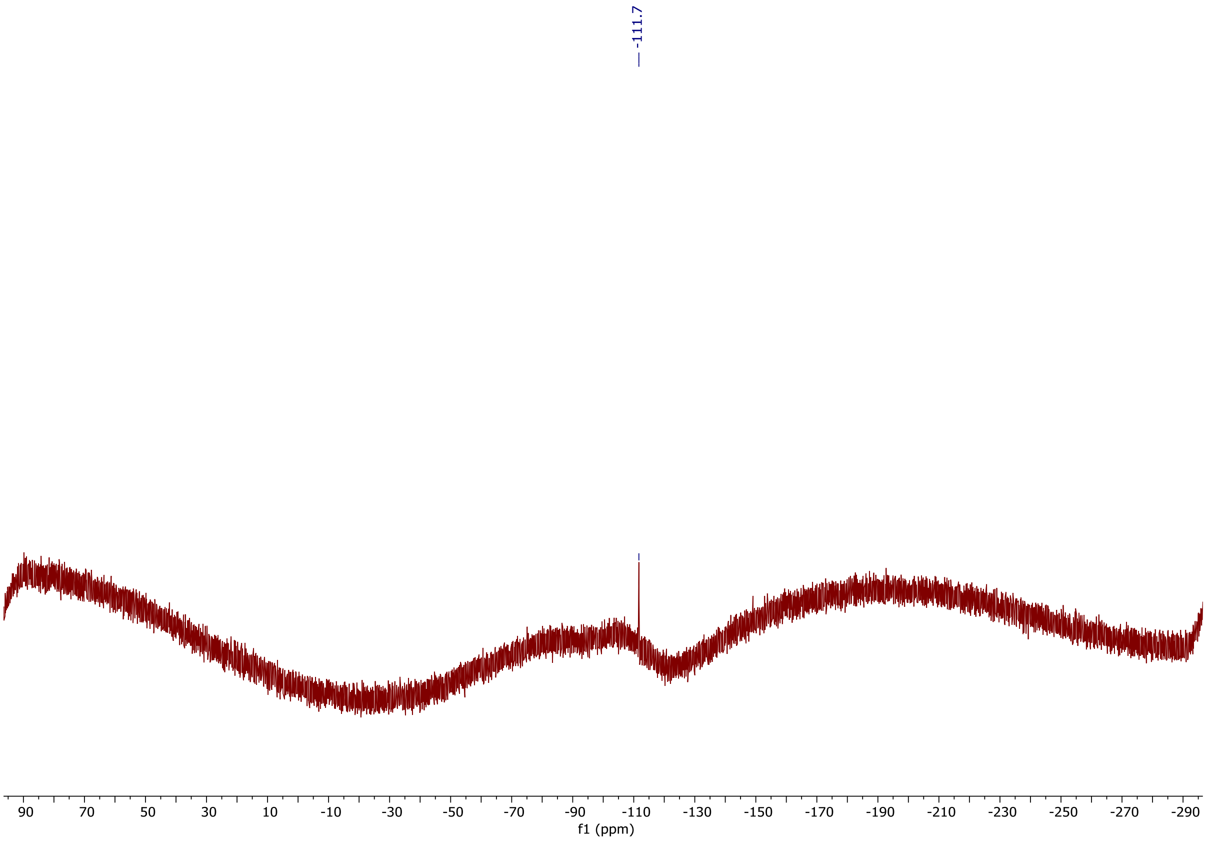

Figure S1.46. ^{29}Si NMR spectrum (79 MHz, $CDCl_3$) of **1-HMPA**.

1.17 1-DABCO

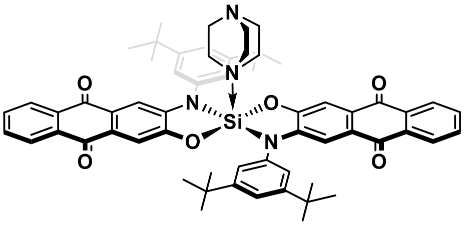

1 (40.0 mg, 45.5 µmol, 1.0 eq) was dissolved in DCM (1 ml). DABCO (6.6 mg, 59 µmol, 1.3 eq) was added, resulting in an orange solution. *n*-pentane (4 ml) was added and the solution was stored at –40 °C overnight, resulting in the precipitation of an orange solid. The solid was filtered off, washed with *n*-pentane (3x1 ml) and dried at atmospheric pressure for 4 hours (35.2 mg, 35.5 µmol, 78 %).

Note: Drying in vacuo *leads to decomposition of the product, likely related to sublimation and loss of DABCO. Therefore, drying was not complete and small amounts of n-pentane are still present in the sample.*

^{1}H NMR (400 MHz, CD_2Cl_2) δ 8.22 – 8.10 (m, 4H), 7.76 – 7.65 (m, 4H), 7.44 (s, 2H), 7.36 (s, 2H), 7.27 (s, 2H), 7.00 (s, 4H), 3.07 (s, 12H), 1.32 (s, 36H).

^{13}C NMR (101 MHz, CD_2Cl_2) δ 183.1, 182.6, 152.6, 152.3, 147.1, 141.8, 134.4, 134.2, 133.7, 133.5, 128.7, 127.5, 126.9, 126.8, 122.9, 120.5, 108.7, 107.8, 47.4, 35.3, 31.6.

^{29}Si NMR (80 MHz, CD_2Cl_2) δ -101.2.

UV-vis (DCM): λ_{max} (ε) = 449 nm (8200 $M^{-1}cm^{-1}$).

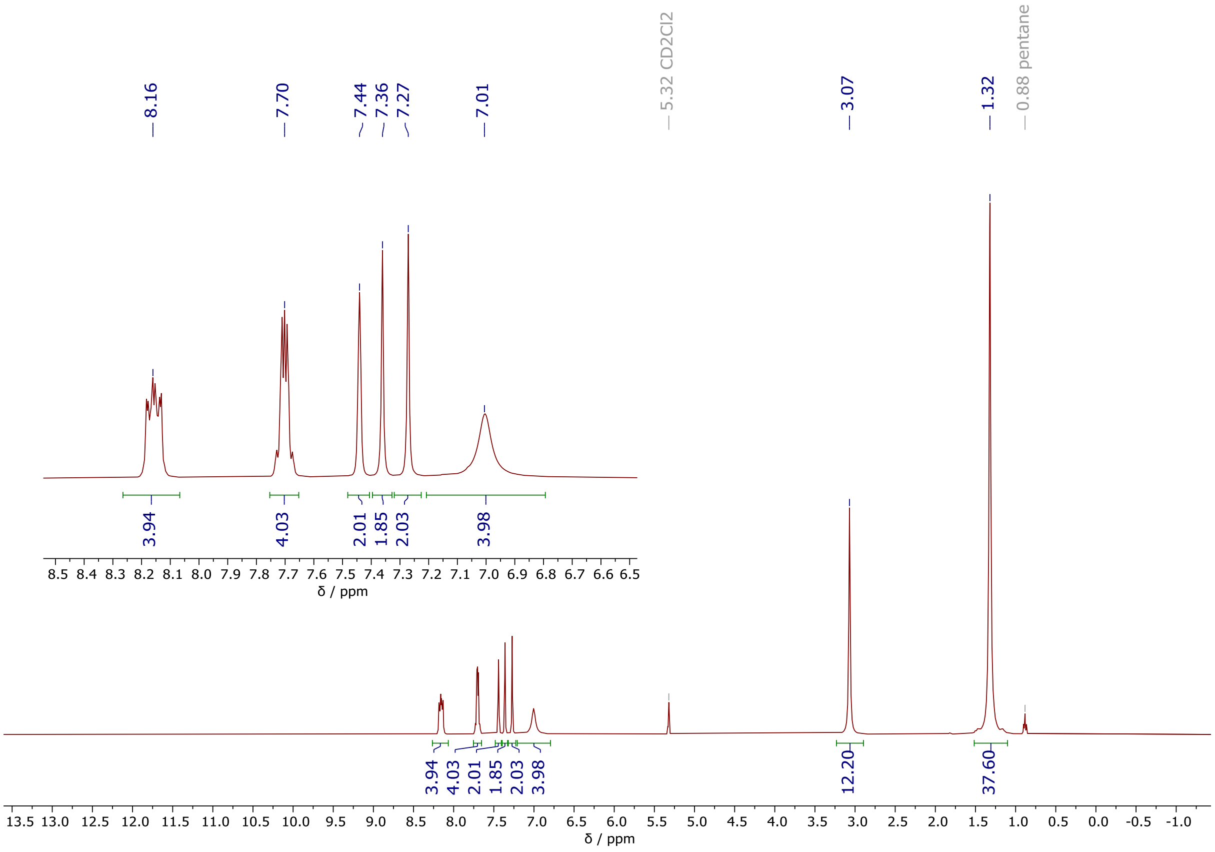

Figure S1.47. ^{1}H NMR spectrum (400 MHz, CD_2Cl_2) of **1-DABCO**.

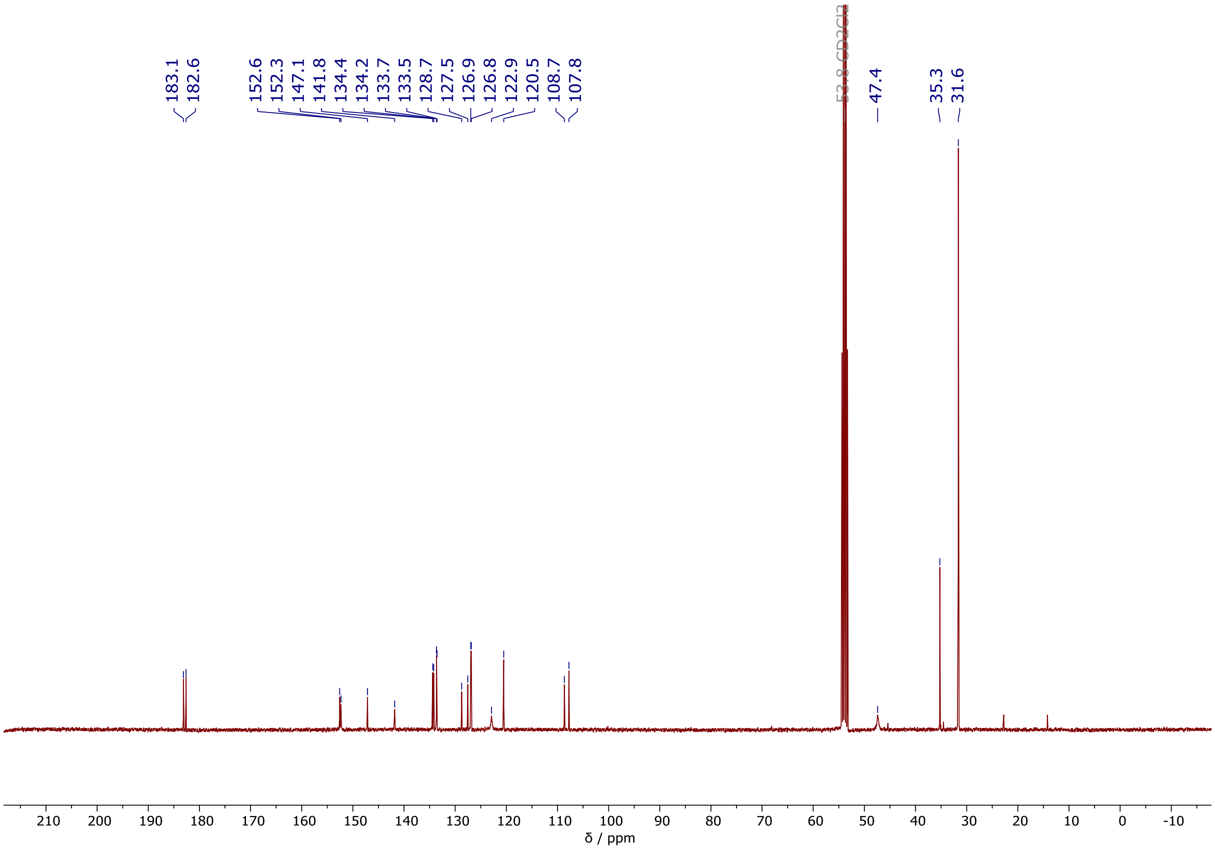

Figure S1.48. ^{13}C NMR spectrum (101 MHz, CD_2Cl_2) of **1-DABCO**.

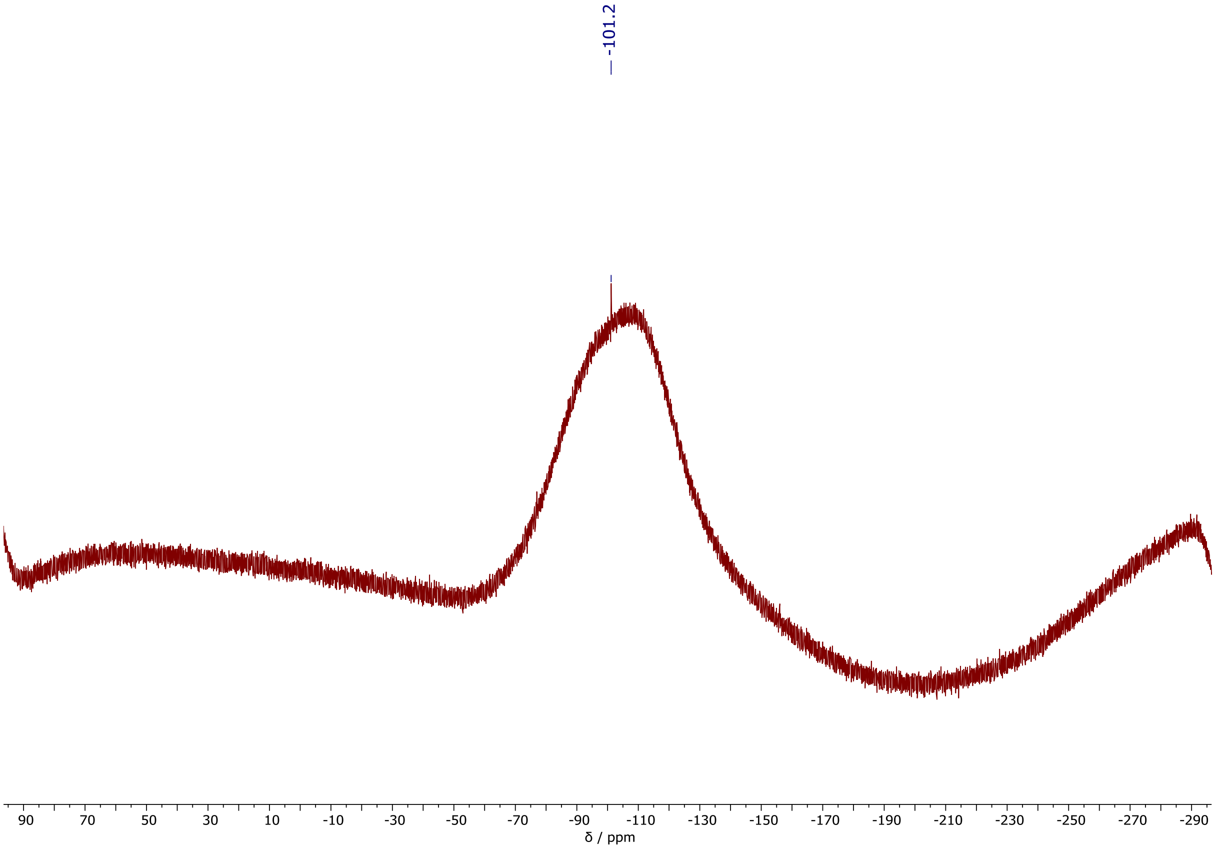

Figure S1.49. ^{29}Si NMR spectrum (80 MHz, CD_2Cl_2) of **1-DABCO**.

1.18 1-^{iPr}NHC

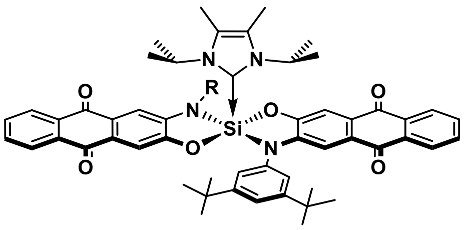

1 (40.0 mg, 45.5 µmol, 1.00 eq) was dissolved in DCM (2 ml). 4,5-Me_2-1,3-$(iPr)_2$-NHC (8.2 mg, 45.5 µmol, 1.00 eq) was added, resulting in an orange solution. Pentane (4 ml) was added, giving a clear solution, which was stored at –40 °C overnight. The formed crystals were filtered off and washed with a 5:1 n-pentane/DCM mixture (3 x 2 ml). The product was isolated as an orange solid after drying *in vacuo* (33.6 mg, 31.7 µmol, 70 %).

^{1}H NMR (400 MHz, CD_2Cl_2) δ 8.25 – 8.06 (m, 4H), 7.71 – 7.62 (m, 4H), 7.38 (s, 2H), 7.22 (overlapping: s, 2H; br, 2H, combined 4H), 7.04 (s, 2H), 6.65 (br, 2H), 5.37 (sept, J = 7.0 Hz, 2H), 2.25 (s, 6H), 1.54 (d, J = 6.9 Hz, 6H), 1.48 (d, J = 7.0 Hz, 6H), 1.42 – 0.79 (br, 36H).

Note: Signals at 1.54, 1.48, and signals of residual pentane are overlapping with the broad signal of the tBu groups to give an overall integral of 53H.

^{13}C NMR (101 MHz, CD_2Cl_2) δ 183.2, 182.9, 155.7, 155.4, 152.3, 147.1, 142.2, 134.5, 134.4, 133.3, 127.8, 127.5, 126.8, 126.8, 126.7, 123.2, 122.0, 120.6, 108.3, 107.1, 52.5, 35.1, 31.5, 22.8, 21.6, 10.9.

^{29}Si NMR (80 MHz, CD_2Cl_2) δ -102.3.

UV-vis (DCM): λ_{max} (ε) = 468 nm (7000 $M^{-1}cm^{-1}$).

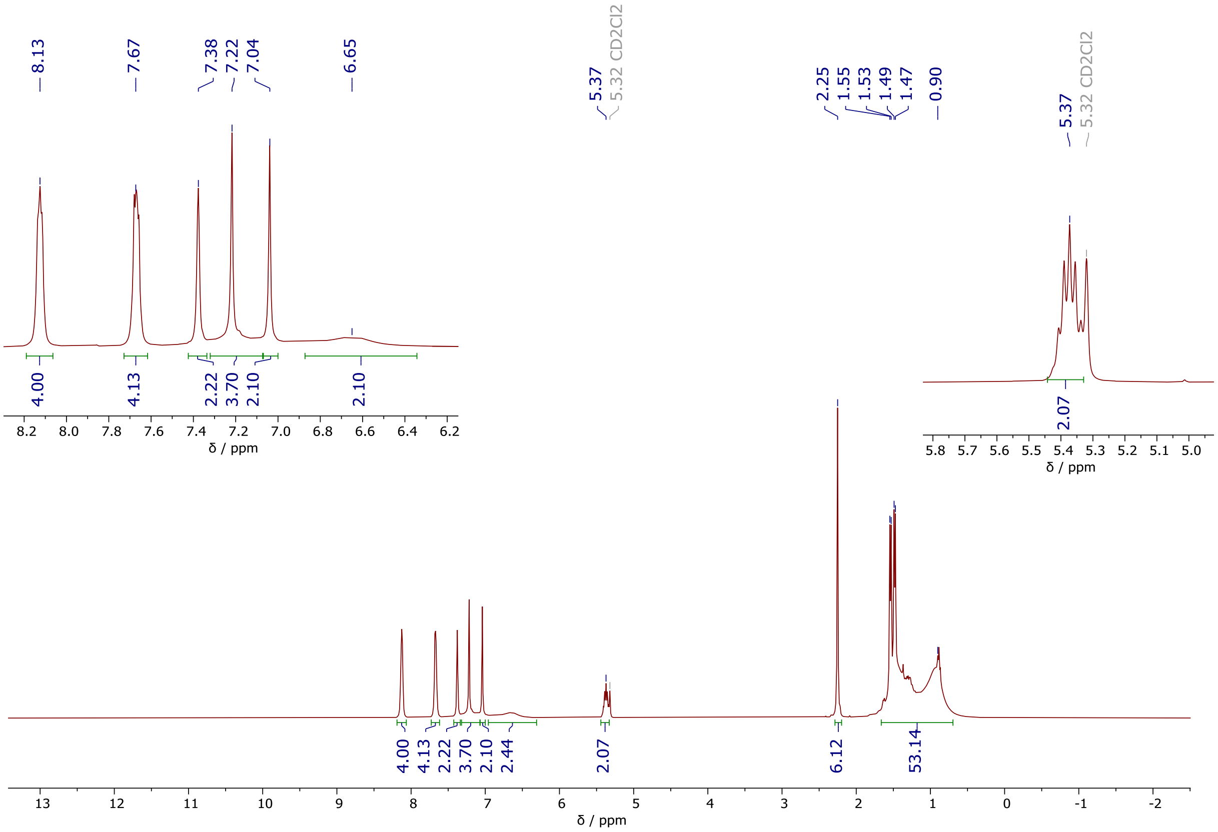

Figure S1.50. ^{1}H NMR spectrum (400 MHz, CD_2Cl_2) of **1-^{iPr}NHC**.

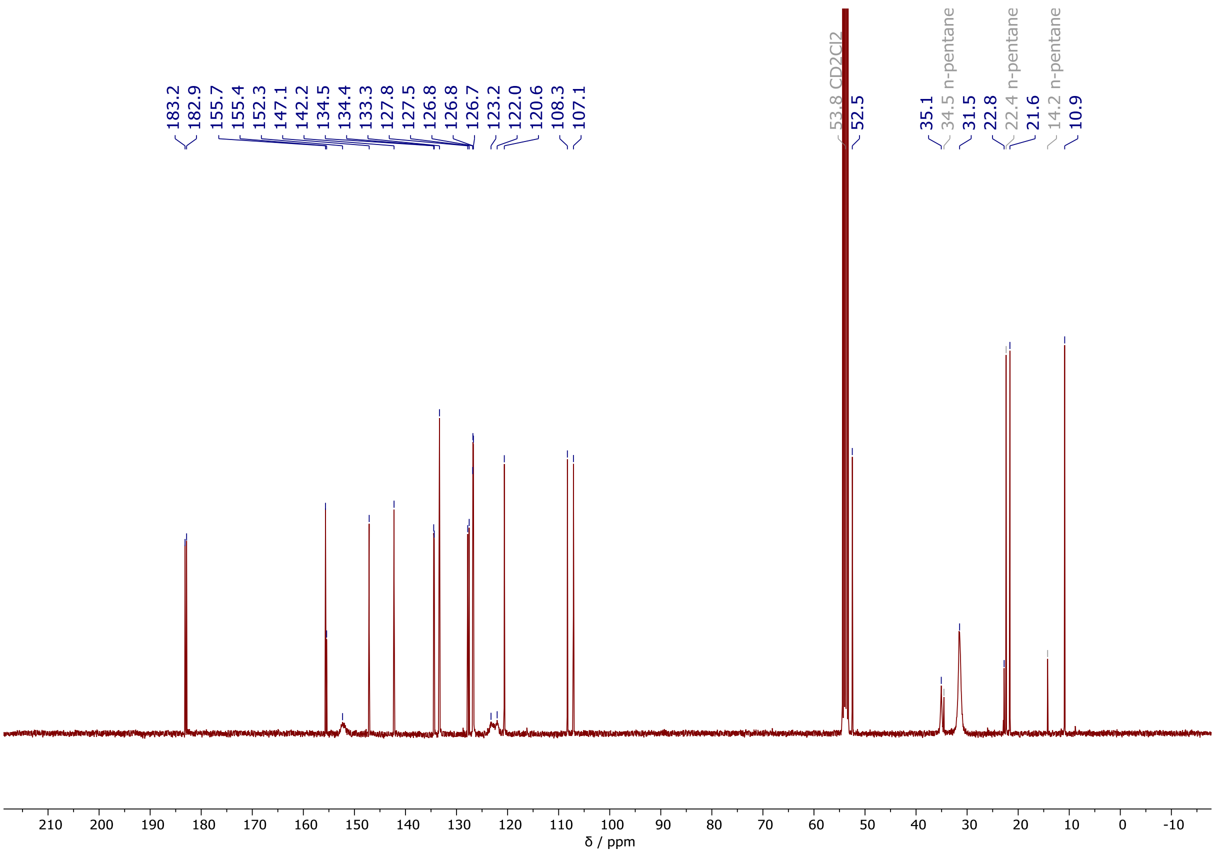

Figure S1.51. ^{1}H NMR spectrum (101 MHz, CD_2Cl_2) of **1-^{iPr}NHC**.

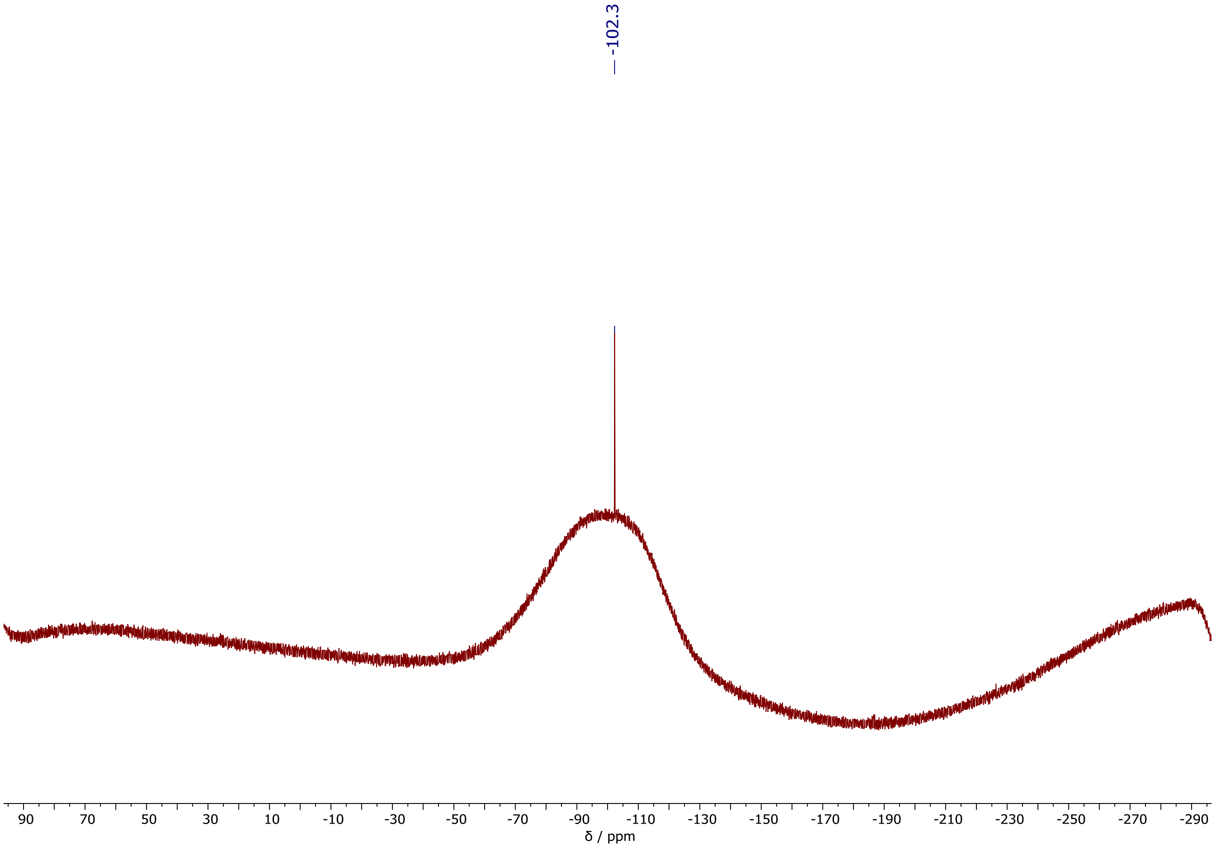

Figure S1.52. ^{29}Si NMR spectrum (80 MHz, CD_2Cl_2) of **1-^{iPr}NHC**.

1.19 1-PCy$_3$

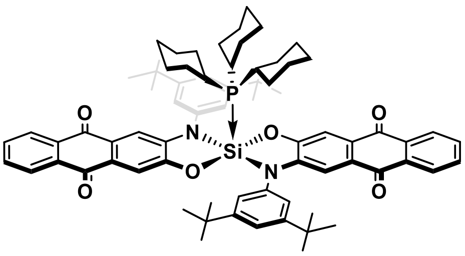

1 (40.0 mg, 45.5 µmol, 1.00 eq) was suspended in DCM (1 ml). Tricyclohexylphosphine (12.8 mg, 45.5 µmol, 1.00 eq) was added, resulting in an orange solution. The solution was layered with *n*-pentane and stored at –40 °C for 7 days, resulting in the formation of orange crystals suitable for scXRD. The supernatant solution was decanted off and the orange solid dried *in vacuo* (26.3 mg, 24.3 µmol, 53 %).

^{1}H NMR (400 MHz, CD_2Cl_2) δ 8.27 – 8.07 (m, 4H), 7.75 – 7.67 (m, 4H), 7.45 (br, 4H), 7.34 (s, 2H), 6.97 (br, 4H), 2.25 – 0.79 (m, 69H*, cyclohexyl, 33H, and tBu, 36H*).

*Note: The dynamics of **1-PCy$_3$** result in line broadening in the ^{1}H and ^{13}C NMR spectra. Due to the broadening, one expected signal is not observed in the ^{13}C NMR spectrum.*

^{13}C NMR (101 MHz, CD_2Cl_2) δ 183.0, 182.5, 152.7, 145.5, 140.0, 134.3, 134.1, 133.8, 133.7, 129.3, 127.9, 127.0, 126.9, 121.5, 120.9, 109.6, 108.1, 35.1, 32.5 (d, *J* = 10.1 Hz), 31.4, 29.7, 27.9 (d, *J* = 9.8 Hz), 26.8.

^{29}Si NMR (80 MHz, CD_2Cl_2) *No signal observed, likely due to line broadening resulting from dynamic binding of PCy$_3$.*

^{31}P NMR (162 MHz, CD_2Cl_2) δ 8.5.

UV-vis (DCM): λ_{max} (ε) = 461 nm (8000 $M^{-1}cm^{-1}$).

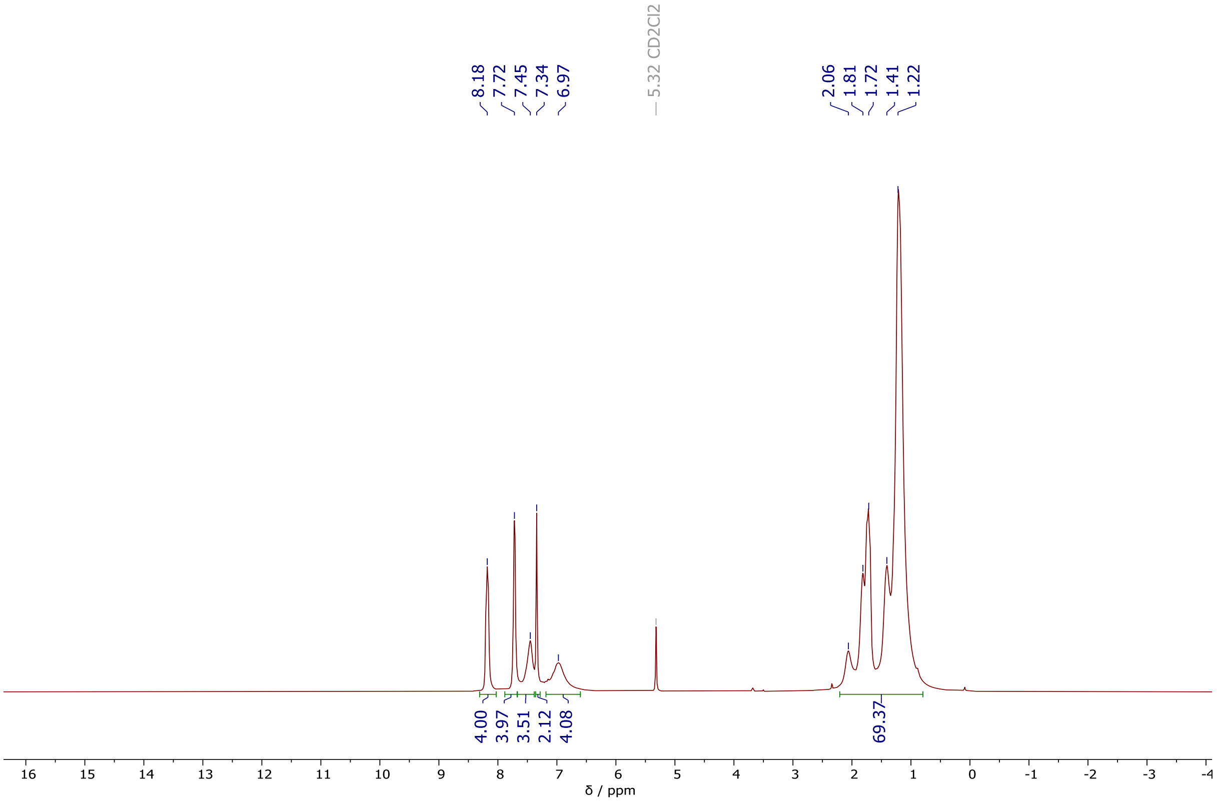

Figure S1.53. ^{1}H NMR spectrum (400 MHz, CD_2Cl_2) of **1-PCy$_3$**.

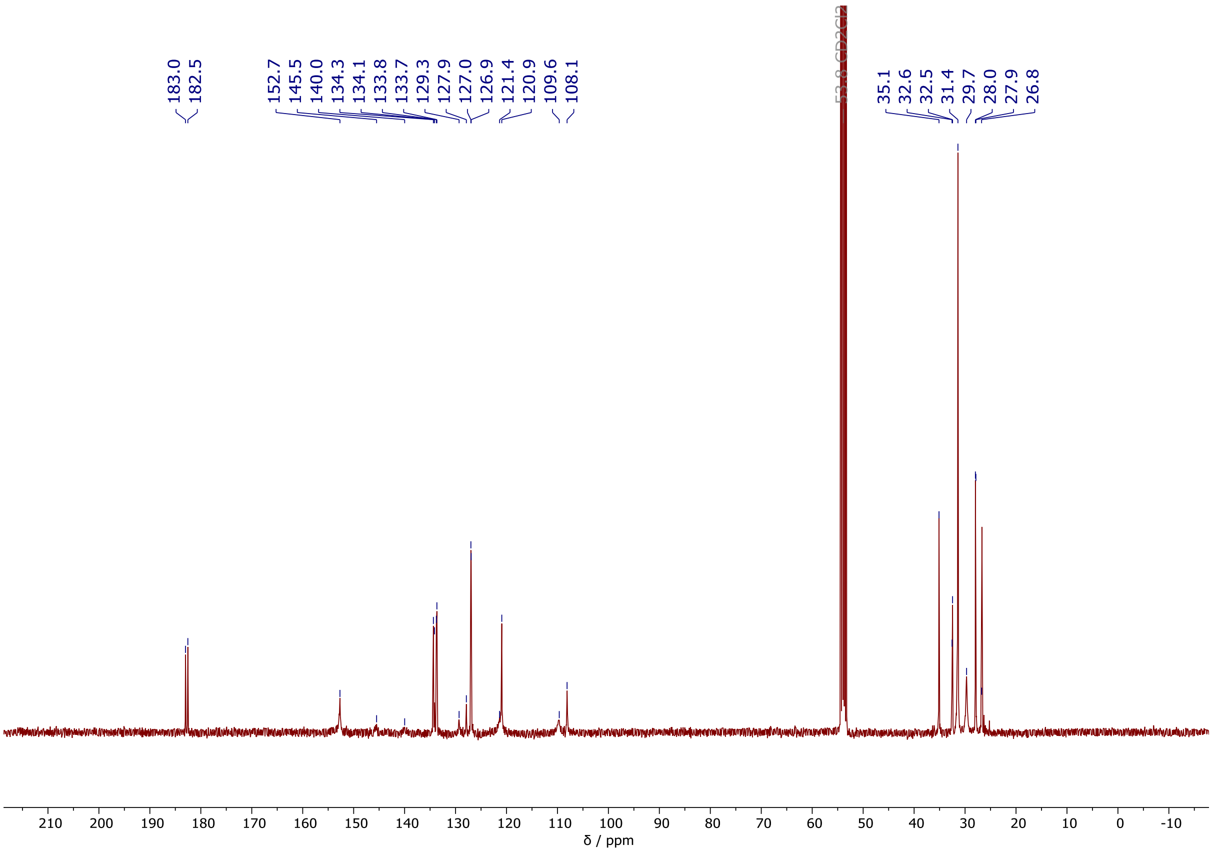

Figure S1.54. ^{13}C NMR spectrum (101 MHz, CD_2Cl_2) of **1-PCy$_3$**.

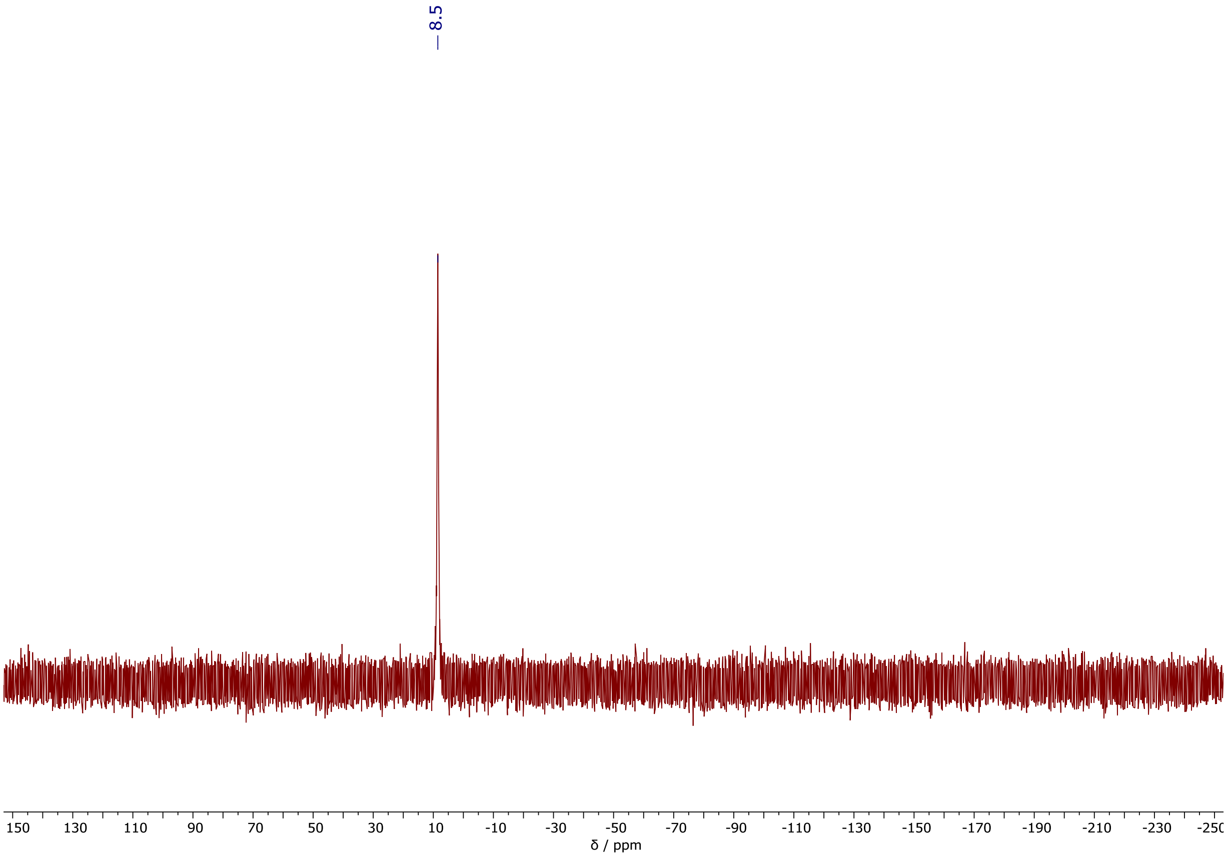

Figure S1.55. ^{31}P NMR spectrum (162 MHz, CD_2Cl_2) of **1-PCy$_3$**.

1.20 1-SIMes

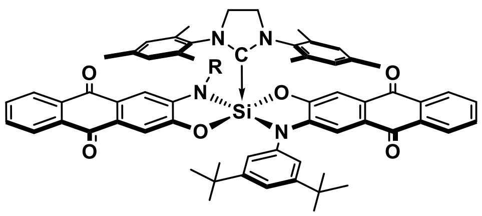

1 (40.0 mg, 45.5 µmol, 1.00 eq) was dissolved in DCM (1.5 ml). 1,3-Dimesityl-imidazol-4,5-dihydro-2-ylidene (SIMes) (13.9 mg, 45.5 µmol, 1.00 eq) was added, resulting in a dark orange solution. The solution was layered with *n*-pentane (4 ml) and stored at –40 °C for 4 days. The supernatant was decanted from the formed crystals, which were washed with a 5:1 n-pentane/DCM mixture (2 x 2 ml). The obtained orange solid was dried in vacuo (27.5 mg, 23.2 µmol, 51 %).

^{1}H NMR (600 MHz, CD_2Cl_2) δ 8.19 – 8.15 (m, 2H), 8.14 – 8.10 (m, 2H), 7.73 – 7.65 (m, 4H), 7.23 (s, 2H), 7.21 (s, 2H), 7.11 (s, 2H), 7.03 (s, 2H), 6.82 (s, 2H), 6.15 (s, 2H), 6.13 (s, 2H), 4.27 – 3.94 (m, 4H), 2.49 (s, 6H), 2.25 (s, 6H), 1.90 (s, 6H), 1.56 (s, 18H), 0.63 (s, 18H).

^{13}C NMR (101 MHz, CD_2Cl_2) δ 183.0, 182.8, 181.8, 155.8, 152.1, 150.7, 146.1, 142.6, 140.2, 136.5, 136.4, 134.6, 134.3, 133.3, 133.3, 132.4, 129.4, 128.9, 127.2, 127.1, 126.8, 126.7, 122.5, 122.3, 119.9, 108.2, 106.8, 51.3, 35.5, 34.5, 32.0, 30.8, 20.8, 18.6, 18.1.

^{29}Si NMR (80 MHz, CH_2Cl_2) δ -102.3.

UV-vis (DCM): λ_{max} (ε) = 472 nm (7000 $M^{-1}cm^{-1}$).

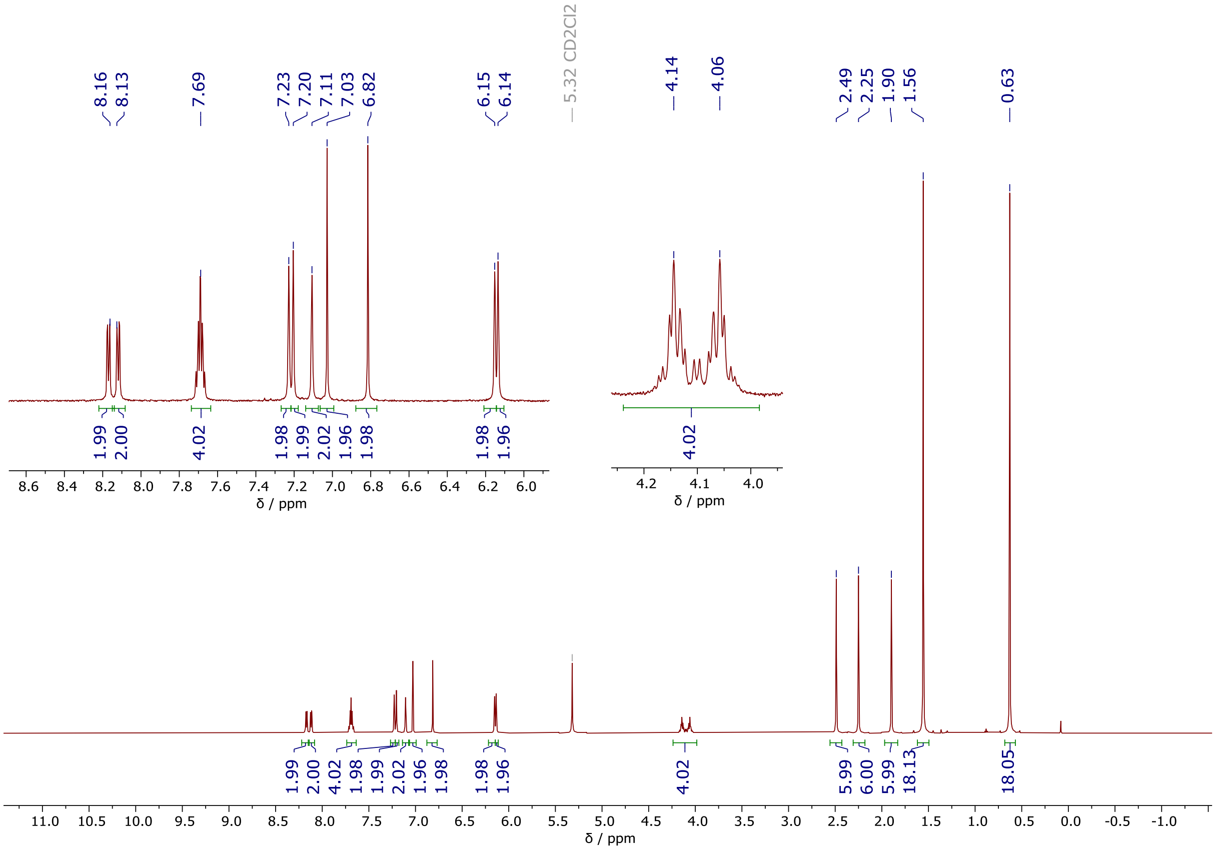

Figure S1.56. ^{1}H NMR spectrum (600 MHz, CD_2Cl_2) of **1-SIMes**.

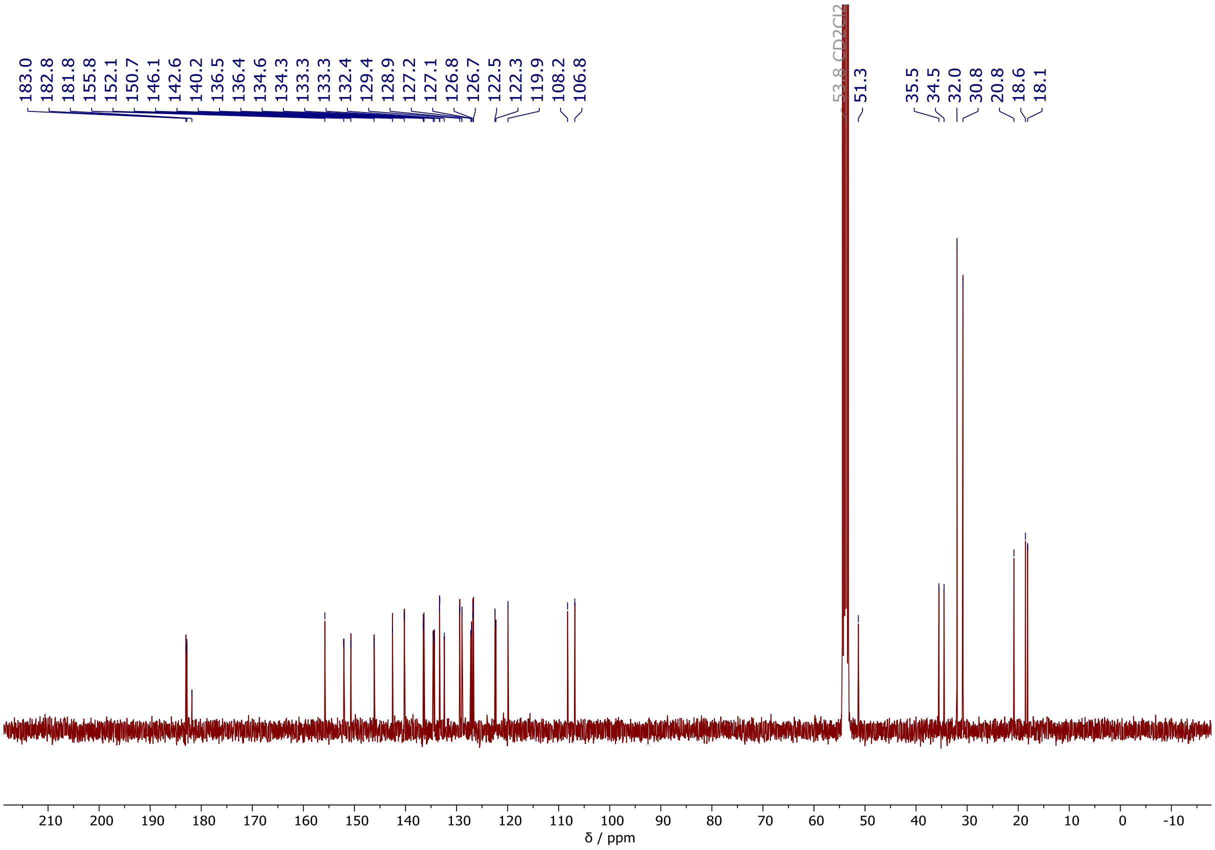

Figure S1.57. ^{13}C NMR spectrum (101 MHz, CD_2Cl_2) of **1-SIMes**.

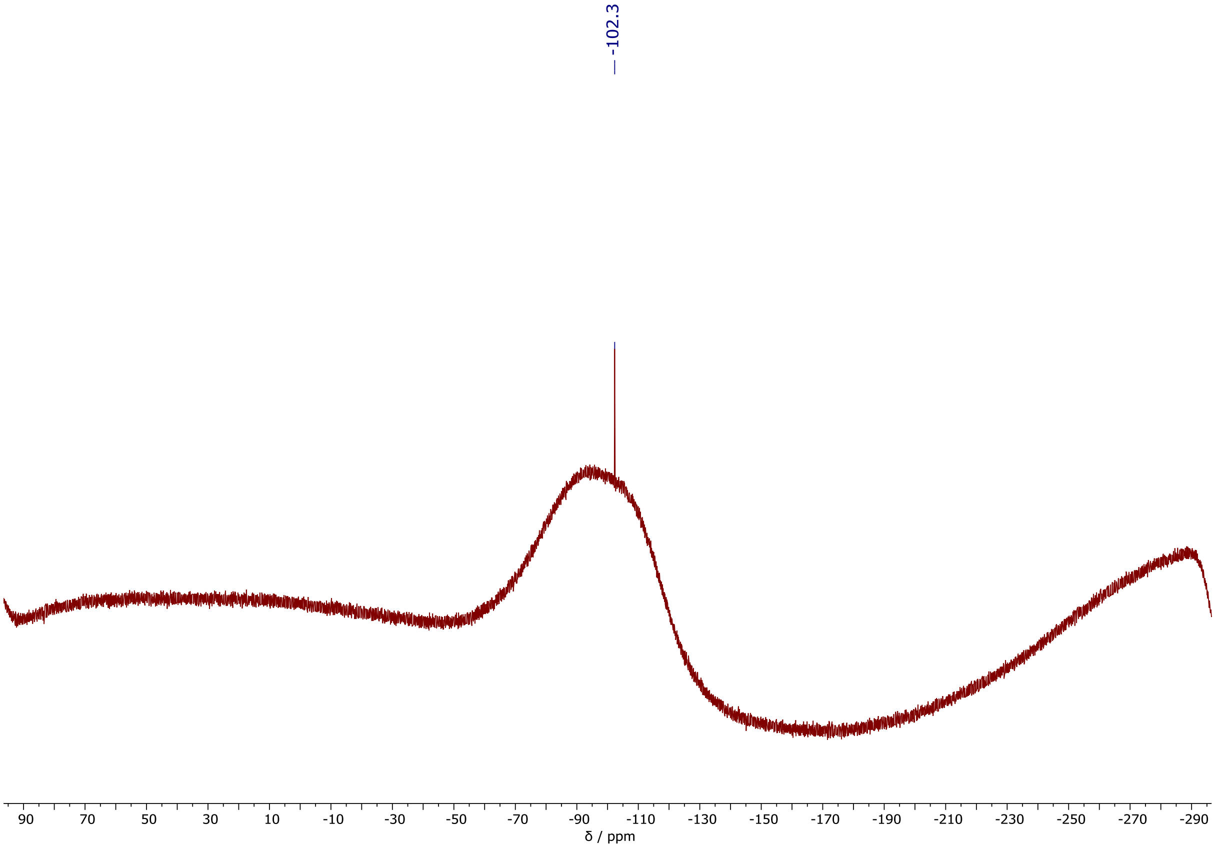

Figure S1.58. ^{29}Si NMR spectrum (80 MHz, CH_2Cl_2) of **1-SIMes**.

1.21 Coordination State of 1-pyridine in Solution

When isolating the pyridine-coordinated silicon complex, two equivalents of pyridine are present in the bulk solid. This, along with the much darker red colour of the solid, indicated the formation of the pyridine bis-adduct in the solid state. Because the interpretations of the UV-vis data are based on the mono-adduct, it is crucial to ensure that only the mono-adduct is present in dilute solutions.

The first indication is the ^{29}Si NMR shift of -104.9 ppm, which is consistent with similar pentacoordinate silicon species in literature[2] and in this work.

Secondly, the UV-vis spectrum of 1-pyridine in solution gives an absorption spectrum in line with TD-DFT calculations on the mono-adduct. Given the good agreement of TD-DFT calculations with all other complexes (see computational section), they are reasonably good predictors of coordination states for these complexes. Adding a large excess of pyridine (200 equivalents) results in λ_{max} redshifted by 50 nm, which corresponds to the calculated absorption spectrum of 1-(pyridine)$_2$. The ability to bind a second pyridine molecule is corroborated by calculated thermodynamics, which give a higher affinity for the binding of one pyridine equivalent, but a still slightly exergonic binding of the second pyridine (ΔG = -24 kJ/mol and -6 kJ/mol, respectively, see computational section for further details).

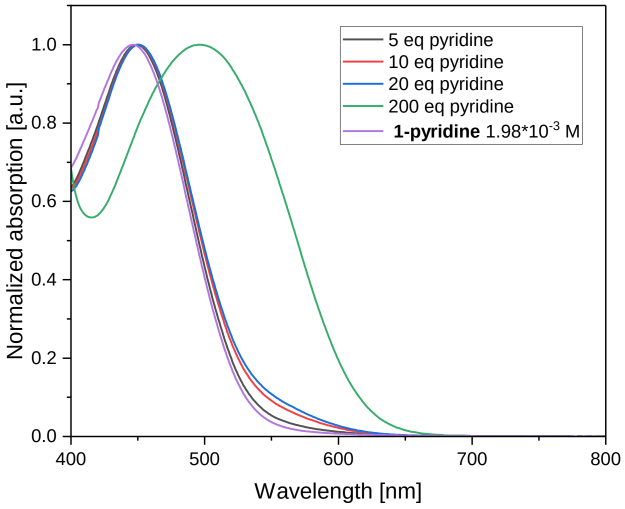

Figure S1.59. Normalised absorption spectrum of **1-pyridine** in DCM with additional equivalents of pyridine. λ_{max} (1-pyridine) = 452 nm, λ_{max} (1-(pyridine)$_2$) = 496 nm.

λ_{max} (exp, DCM, 1-pyridine) = 452 nm,

λ_{max} (exp, DCM, 1-(pyridine)$_2$) = 496 nm.

λ_{max} (ωB97X-D3, DCM, 1-pyridine) = 444 nm,

λ_{max} (ωB97X-D3, DCM, 1-(pyridine)$_2$) = 491 nm.

2 UV-vis Spectroscopy

UV-vis spectra were measured on a JASCO V-570 UV/VIS/NIR spectrophotometer. Measurements were carried out in 2 mm quartz cuvette equipped with a J-Young valve with a volume of 700 µL. A stock solution in 1000 µL DCM was prepared for all compounds. Different concentrations were used to obtain a more accurate estimate for the extinction coefficient of the CT band. Additionally, measurements at differing concentrations provided a method to determine if dissociation of the acid-base adducts occurs at low concentrations. If dissociation was observed, additional equivalents of base were added to obtain spectra of the fully associated complexes. All data analysis was done with OriginLab 2024b.

2.1 CT Absorption Bands of all Compounds

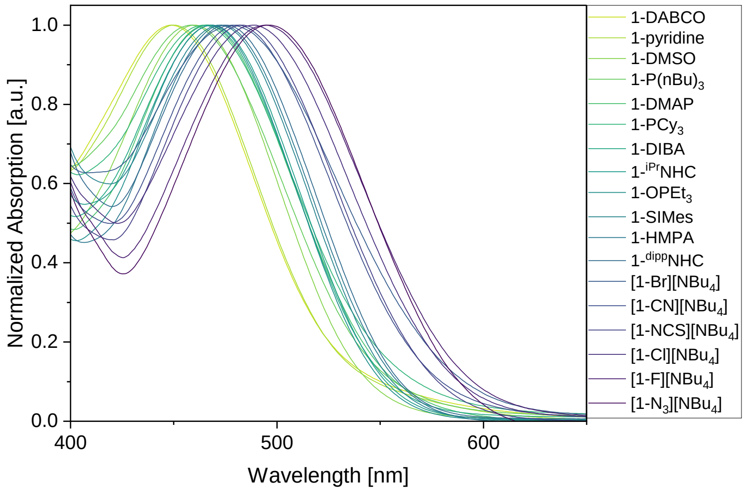

Figure S2.1. Normalised UV-vis absorption spectra of adducts **1-X** (CT band, DCM).

2.2 1

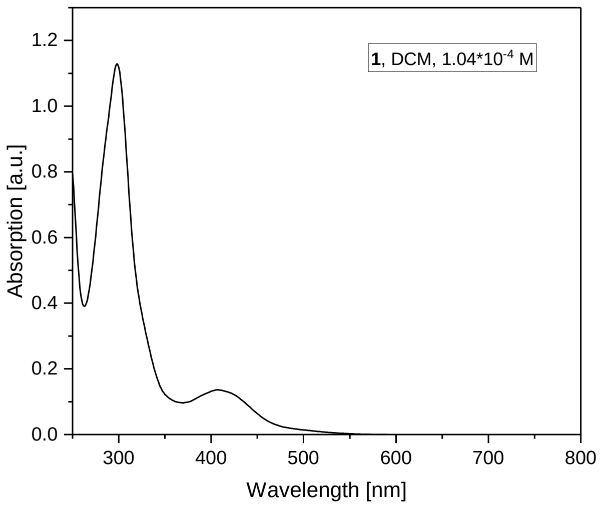

Figure S2.2. UV-vis absorption spectrum of **1** ($1.04\cdot10^{-4}$ M, DCM).

Stock solution:

6.5 mg **1** in 1000 µL DCM.

<u>Cuvette:</u> d = 2 mm, V = 700 µL

<u>Aliquots:</u> 10 µL, 30 µL, 60 µL

Concentrations:

i) $$\frac{\left(\frac{0.0065g}{878\ g/mol}\right)}{0.001\ l}\cdot\frac{10\ \mu\text{L}}{710\ \mu\text{L}} = 1.043\cdot10^{-4}\ M$$

ii) $$\frac{\left(\frac{0.0065g}{878\ g/mol}\right)}{0.001\ l}\cdot\frac{30\ \mu\text{L}}{730\ \mu\text{L}} = 3.042\cdot10^{-4}\ M$$

iii) $$\frac{\left(\frac{0.0065g}{878\ g/mol}\right)}{0.001\ l}\cdot\frac{60\ \mu\text{L}}{760\ \mu\text{L}} = 5.844\cdot10^{-4}\ M$$

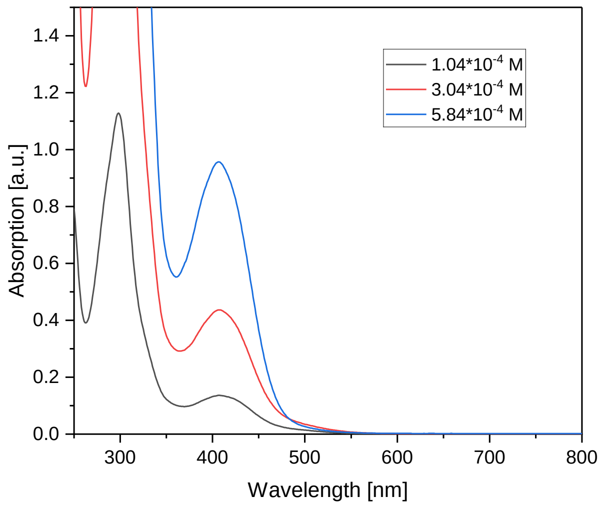

Figure S2.3. UV-vis absorption spectrum of **1**($1.04\cdot10^{-4}$ M, $3.04\cdot10^{-4}$ M, $5.84\cdot10^{-4}$ M, DCM).

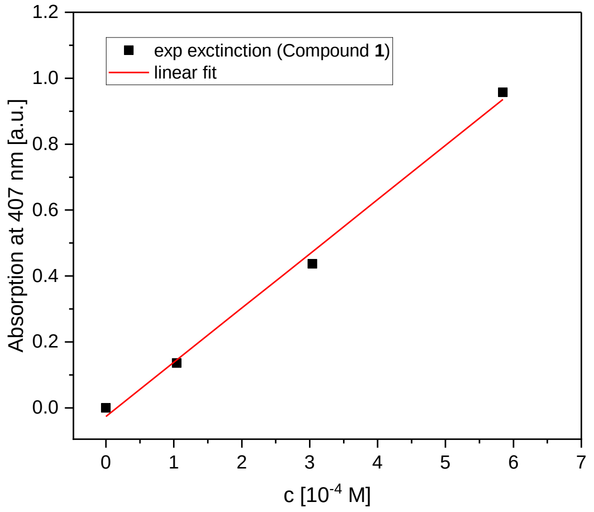

Figure S2.4. Extinction at 407 nm (CT band) of **1** (0 M, $1.04\cdot10^{-4}$ M, $3.04\cdot10^{-4}$ M, $5.84\cdot10^{-4}$ M, DCM). Linear Fit: y = 1644.8x - 0.02584. R^2 = 0.993. $\varepsilon = \frac{1644.8\ M^{-1}}{0.2\ cm} \approx 8200\ M^{-1}cm^{-1}$.

2.3 [1-CN][NBu₄]

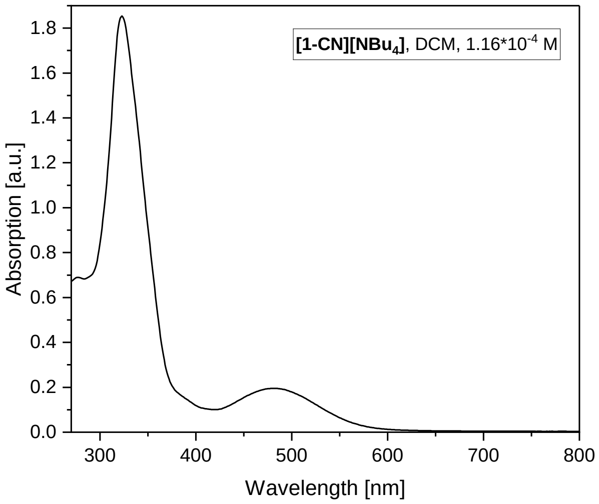

Figure S2.5. UV-vis absorption spectrum of **[1-CN][NBu$_4$]** ($1.16 \cdot 10^{-4}$ M, DCM).

Stock solution:

2.0 mg **[1-CN][NBu$_4$]** in 1000 µL DCM.

<u>Cuvette:</u> d = 2 mm, V = 700 µL

<u>Aliquots:</u> 50 µL, 100 µL, 200 µL

Concentrations:

i) $$\frac{\left(\frac{0.002g}{1147\ g/mol}\right)}{0.001\ l} \cdot \frac{50\ \mu L}{750\ \mu L} = 1.162 \cdot 10^{-4}\ M$$

ii) $$\frac{\left(\frac{0.002g}{1147\ g/mol}\right)}{0.001\ l} \cdot \frac{100\ \mu L}{800\ \mu L} = 2.180 \cdot 10^{-4}\ M$$

iii) $$\frac{\left(\frac{0.002g}{1147\ g/mol}\right)}{0.001\ l} \cdot \frac{200\ \mu L}{900\ \mu L} = 3.875 \cdot 10^{-4}\ M$$

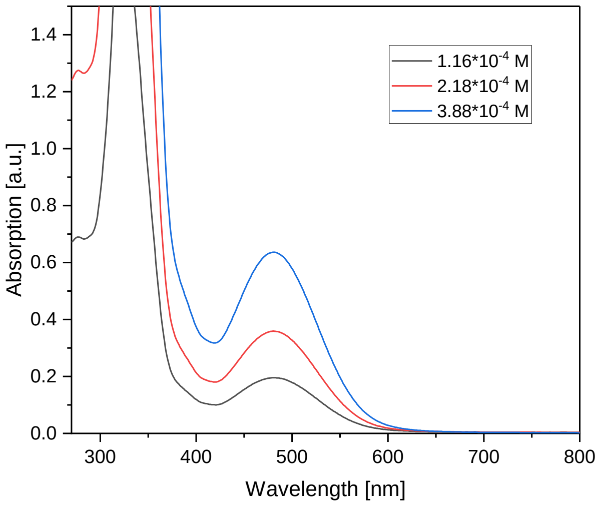

Figure S2.6. UV-vis absorption spectrum of **[1-CN][NBu$_4$]** ($1.16 \cdot 10^{-4}$ M, $2.18 \cdot 10^{-4}$ M, $3.88 \cdot 10^{-4}$ M, DCM). No change of λ_{max} is observed at different concentrations, suggesting negligible dissociation at low concentrations.

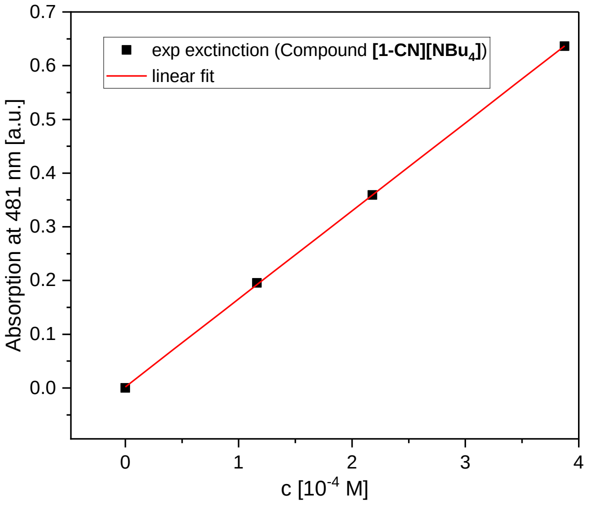

Figure S2.7. Extinction at 481 nm (CT band) of **[1-CN][NBu$_4$]** (0 M, $1.16 \cdot 10^{-4}$ M, $2.18 \cdot 10^{-4}$ M, $3.88 \cdot 10^{-4}$ M, DCM). Linear Fit: y = 1638.1x - 0.00205. R^2 = 0.999. $\varepsilon = \frac{1638.1\ M^{-1}}{0.2\ cm} \approx 8200\ M^{-1}cm^{-1}$.

2.4 [1-Cl][PPh₄]

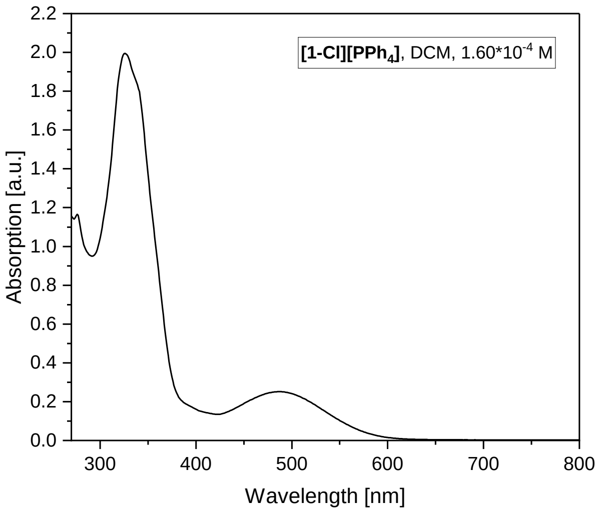

Figure S2.8. UV-vis absorption spectrum of **[1-Cl][PPh₄]** ($1.60{\cdot}10^{-4}$ M, DCM).

Stock solution:

3.0 mg **[1-Cl][PPh₄]** in 1000 µL DCM.

Cuvette: d = 2 mm, V = 700 µL

Aliquots: 50 µL, 100 µL, 200 µL

Concentrations:

i) $\frac{\left(\frac{0.003g}{1252\ g/mol}\right)}{0.001\ l} \cdot \frac{50\ \mu L}{750\ \mu L} = 1.597 \cdot 10^{-4}\ M$

ii) $\frac{\left(\frac{0.003g}{1252\ g/mol}\right)}{0.001\ l} \cdot \frac{100\ \mu L}{800\ \mu L} = 3.000 \cdot 10^{-4}\ M$

iii) $\frac{\left(\frac{0.003g}{1252\ g/mol}\right)}{0.001\ l} \cdot \frac{200\ \mu L}{900\ \mu L} = 5.325 \cdot 10^{-4}\ M$

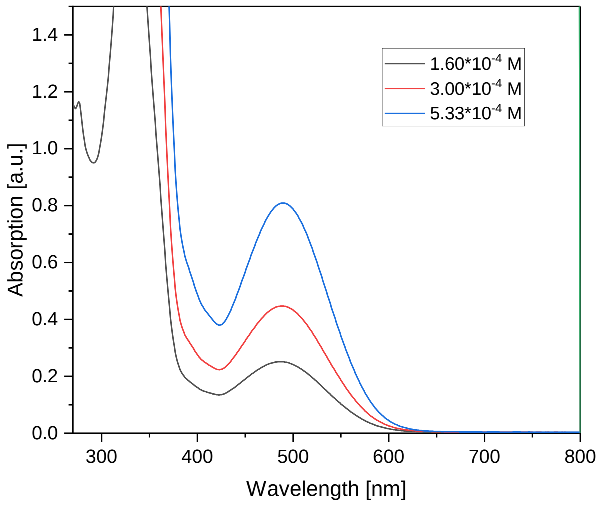

Figure S2.9. UV-vis absorption spectrum of **[1-Cl][PPh₄]** ($1.60{\cdot}10^{-4}$ M, $3.00{\cdot}10^{-4}$ M, $5.33{\cdot}10^{-4}$ M, DCM). No change of λ_{max} is observed at different concentrations, suggesting negligible dissociation at low concentrations.

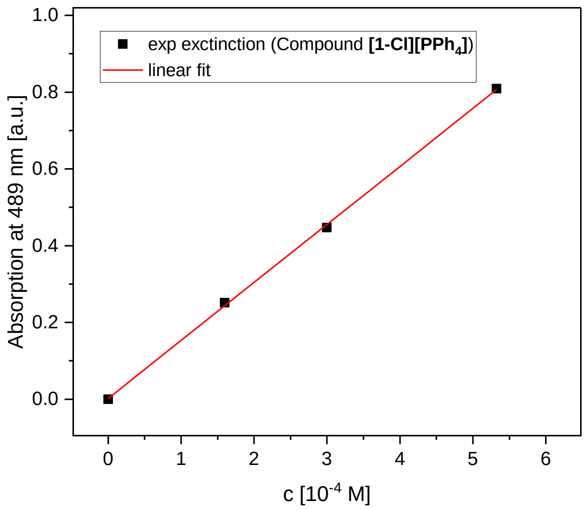

Figure S2.10. Extinction at 489 nm (CT band) of **[1-Cl][PPh₄]** (0 M, $1.60{\cdot}10^{-4}$ M, $3.00{\cdot}10^{-4}$ M, $5.33{\cdot}10^{-4}$ M, DCM). Linear Fit: y = 1511.1x - 0.00203. R^2 = 0.999. ε (489 nm) = $\frac{1511.1\ M^{-1}}{0.2\ cm} \approx 7600\ M^{-1}cm^{-1}$.

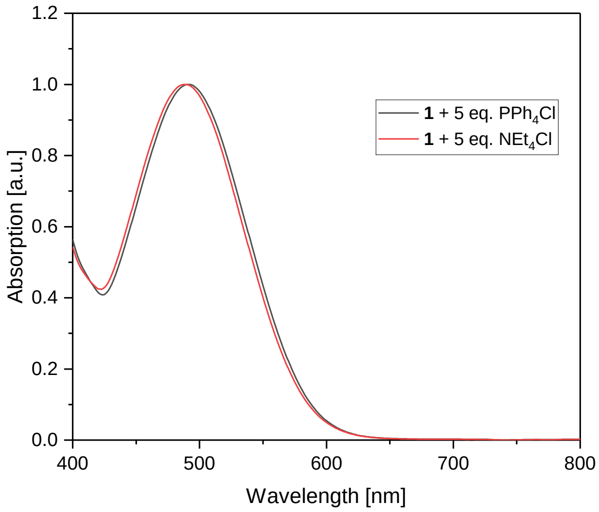

Figure S2.11. Normalised UV-vis absorption spectrum of **1** (ca. 10^{-3} M) with 5 eq. NEt_4Cl or PPh_4Cl added. λ_{max}(**1** + NEt_4Cl) = 489 nm. λ_{max}(**1** + PPh_4Cl) = 490 nm.

2.5 [1-NCS][NBu₄]

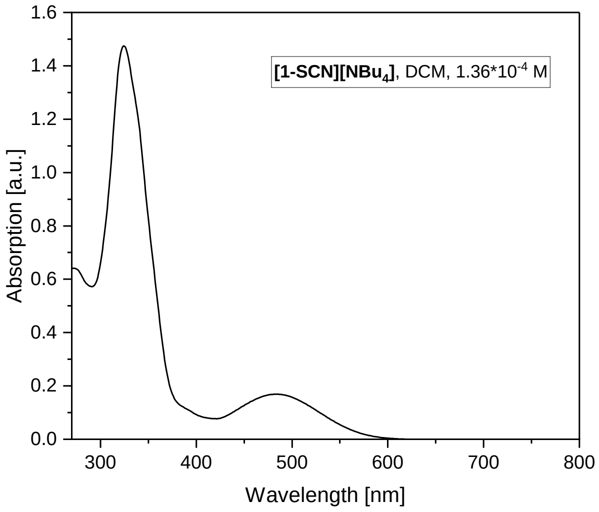

Figure S2.12. UV-vis absorption spectrum of **[1-NCS][NBu$_4$]** ($1.36 \cdot 10^{-4}$ M, DCM).

Stock solution:

2.4 mg **[1-NCS][NBu$_4$]** in 1000 µL DCM.

<u>Cuvette</u>: d = 2 mm, V = 700 µL

<u>Aliquots:</u> 50 µL, 100 µL, 250 µL

Concentrations:

i) $\frac{\left(\frac{0.0024g}{1179\ g/mol}\right)}{0.001\ l} \cdot \frac{50\ \mu\text{L}}{750\ \mu\text{L}} = 1.357 \cdot 10^{-4}\ M$

ii) $\frac{\left(\frac{0.0024g}{1179\ g/mol}\right)}{0.001\ l} \cdot \frac{100\ \mu\text{L}}{800\ \mu\text{L}} = 2.545 \cdot 10^{-4}\ M$

iii) $\frac{\left(\frac{0.0024g}{1179\ g/mol}\right)}{0.001\ l} \cdot \frac{250\ \mu\text{L}}{950\ \mu\text{L}} = 5.357 \cdot 10^{-4}\ M$

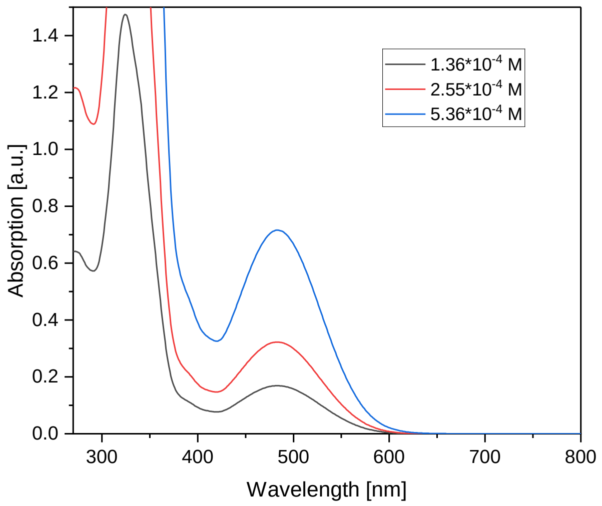

Figure S2.13. UV-vis absorption spectrum of **[1-NCS][PPh$_4$]** ($1.36 \cdot 10^{-4}$ M, $2.55 \cdot 10^{-4}$ M, $5.36 \cdot 10^{-4}$ M, DCM). No change of λ_{max} is observed at different concentrations, suggesting negligible dissociation at low concentrations.

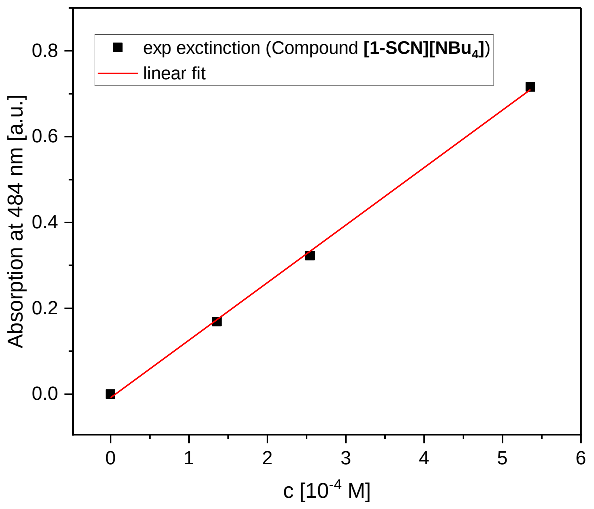

Figure S2.14. Extinction at 489 nm (CT band) of **[1-NCS][PPh$_4$]** (0 M, $1.36 \cdot 10^{-4}$ M, $2.55 \cdot 10^{-4}$ M, $5.36 \cdot 10^{-4}$ M, DCM) Linear Fit: y = 1340.7x - 0.00862. R^2 = 0.999. ε (484 nm) = $\frac{1340.7\ M^{-1}}{0.2\ cm} \approx 6700\ M^{-1}cm^{-1}$.

2.6 [1-N$_3$][NBu$_4$]

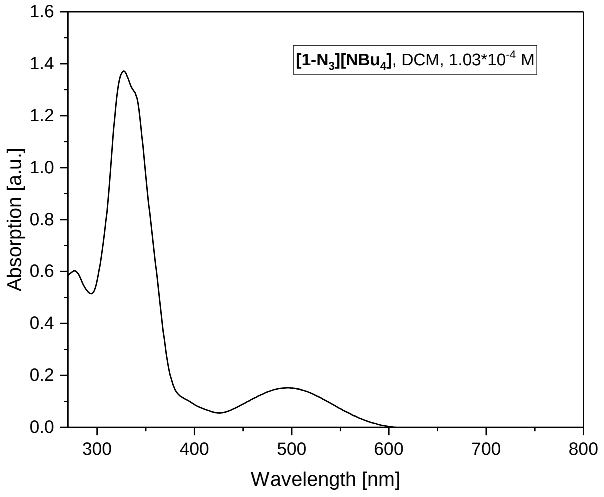

Stock solution:

1.8 mg **[1-N$_3$][NBu$_4$]** in 1000 µL DCM.

Cuvette: d = 2 mm, V = 700 µL

Aliquots: 50 µL, 100 µL, 200 µL

Concentrations:

i) $$\frac{\left(\frac{0.0018g}{1163\ g/mol}\right)}{0.001\ l} \cdot \frac{50\ \mu L}{750\ \mu L} = 1.032 \cdot 10^{-4}\ M$$

ii) $$\frac{\left(\frac{0.0018g}{1163\ g/mol}\right)}{0.001\ l} \cdot \frac{100\ \mu L}{800\ \mu L} = 1.935 \cdot 10^{-4}\ M$$

iii) $$\frac{\left(\frac{0.0018g}{1163\ g/mol}\right)}{0.001\ l} \cdot \frac{200\ \mu L}{900\ \mu L} = 3.439 \cdot 10^{-4}\ M$$

Figure S2.15. UV-vis absorption spectrum of **[1-N$_3$][NBu$_4$]** ($1.03 \cdot 10^{-4}$ M, DCM).

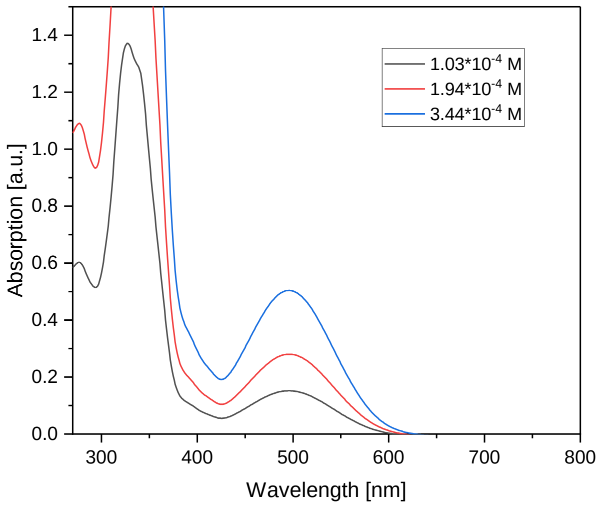

Figure S2.16. UV-vis absorption spectrum of **[1-N$_3$][NBu$_4$]** ($1.03 \cdot 10^{-4}$ M, $1.94 \cdot 10^{-4}$ M, $3.44 \cdot 10^{-4}$ M, DCM). No change of λ_{max} is observed at different concentrations, suggesting negligible dissociation at low concentrations.

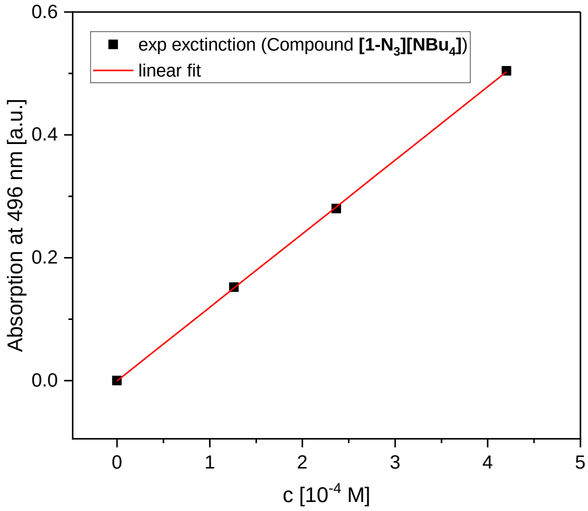

Figure S2.17. Extinction at 496 nm (CT band) of **[1-N$_3$][NBu$_4$]** (0 M, $1.03 \cdot 10^{-4}$ M, $1.94 \cdot 10^{-4}$ M, $3.44 \cdot 10^{-4}$ M, DCM). Linear Fit: y = 1463.3x - 0.00036. R^2 = 0.999. ε (496 nm)= $\frac{1463.3\ M^{-1}}{0.2\ cm} \approx 7300\ M^{-1}cm^{-1}$.

2.7 [1-F][NBu$_4$]

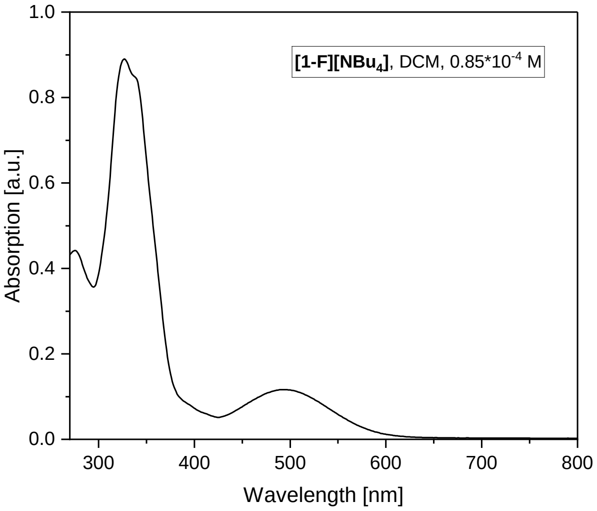

Figure S2.18. UV-vis absorption spectrum of **[1-F][NBu$_4$]** ($0.85\cdot10^{-4}$ M, DCM).

Stock solution:

1.0 mg **[1-F][NBu$_4$]** in 1000 µL DCM.

<u>Cuvette</u>: d = 2 mm, V = 700 µL

<u>Aliquots</u>: 75 µL, 200 µL, 400 µL

Concentrations:

i) $$\frac{\left(\frac{0.001 g}{1140\ g/mol}\right)}{0.001\ l}\cdot\frac{75\ \mu\text{L}}{775\ \mu\text{L}} = 8.498\cdot10^{-5}\ M$$

ii) $$\frac{\left(\frac{0.001 g}{1140\ g/mol}\right)}{0.001\ l}\cdot\frac{200\ \mu\text{L}}{900\ \mu\text{L}} = 1.949\cdot10^{-4}\ M$$

iii) $$\frac{\left(\frac{0.001 g}{1140\ g/mol}\right)}{0.001\ l}\cdot\frac{400\ \mu\text{L}}{1100\ \mu\text{L}} = 3.190\cdot10^{-4}\ M$$

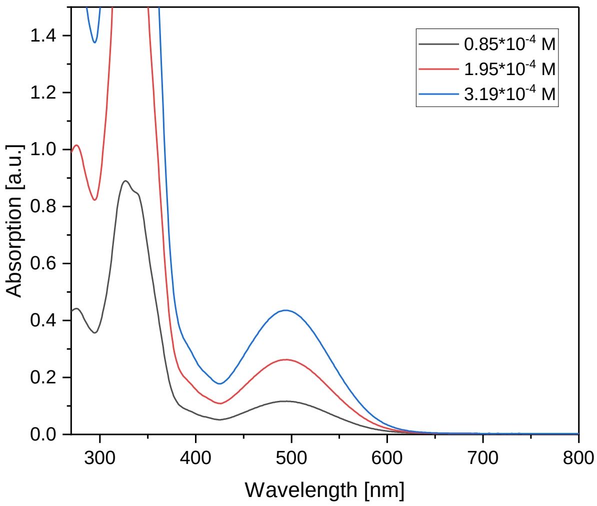

Figure S2.19. UV-vis absorption spectrum of **[1-F][NBu$_4$]** ($0.85\cdot10^{-4}$ M, $1.95\cdot10^{-4}$ M, $3.19\cdot10^{-4}$ M, DCM). No change of λ_{max} is observed at different concentrations, suggesting negligible dissociation at low concentrations.

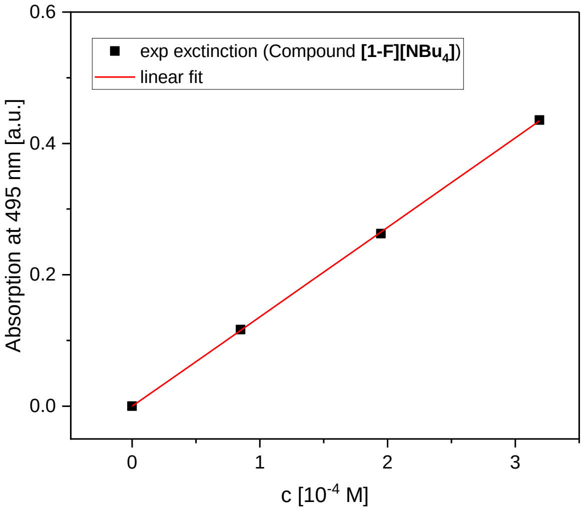

Figure S2.20. Extinction at 495 nm (CT band) of **[1-F][NBu$_4$]** (0 M, $0.85\cdot10^{-4}$ M, $1.95\cdot10^{-4}$ M, $3.19\cdot10^{-4}$ M, DCM). Linear Fit: y = 1362.1x - 0.00032. R^2 = 0.999. ε (502 nm) = $\frac{1362.1\ M^{-1}}{0.2\ cm}\approx 6800\ M^{-1}cm^{-1}$.

2.8 1-HMPA

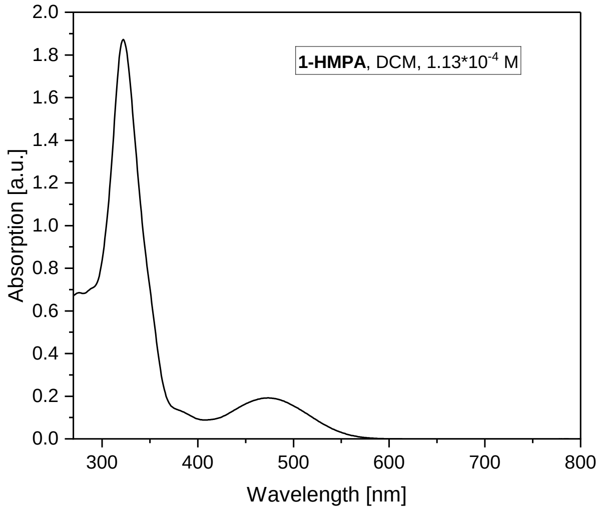

Figure S2.21. UV-vis absorption spectrum of **1-HMPA** ($1.13{\cdot}10^{-4}$ M, DCM).

Stock solution:

1.8 mg **1-HMPA** in 1000 µL DCM.

Cuvette: d = 2 mm, V = 700 µL

Aliquots: 50 µL, 100 µL, 200 µL

Concentrations:

i) $\frac{\left(\frac{0.0018g}{1058\ g/mol}\right)}{0.001\ l} \cdot \frac{50\ \mu L}{750\ \mu L} = 1.134 \cdot 10^{-4}\ M$

ii) $\frac{\left(\frac{0.0018g}{1058\ g/mol}\right)}{0.001\ l} \cdot \frac{100\ \mu L}{800\ \mu L} = 2.123 \cdot 10^{-4}\ M$

iii) $\frac{\left(\frac{0.0018g}{1058\ g/mol}\right)}{0.001\ l} \cdot \frac{200\ \mu L}{900\ \mu L} = 3.871 \cdot 10^{-4}\ M$

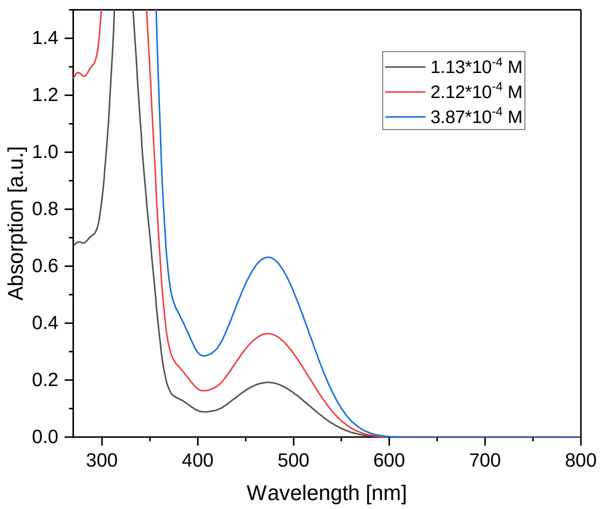

Figure S2.22. UV-vis absorption spectrum of **1-HMPA** ($1.13{\cdot}10^{-4}$ M, $2.12{\cdot}10^{-4}$ M, $3.87{\cdot}10^{-4}$ M, DCM). No change of λ_{max} is observed at different concentrations, suggesting negligible dissociation at low concentrations.

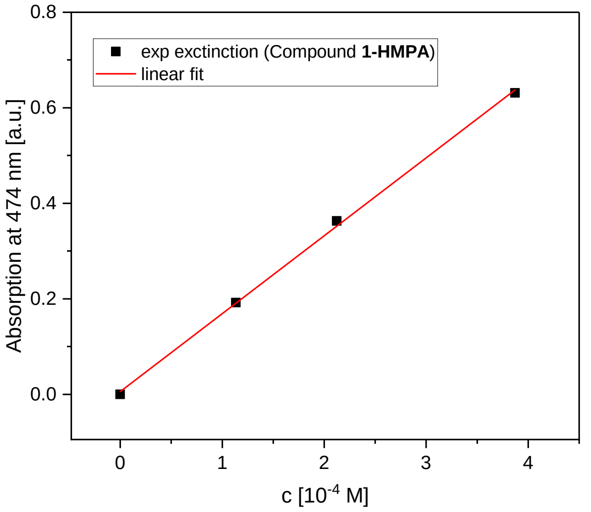

Figure S2.23. Extinction at 474 nm (CT band) of **1-HMPA** (0 M, $1.13{\cdot}10^{-4}$ M, $2.12{\cdot}10^{-4}$ M, $3.87{\cdot}10^{-4}$ M, DCM). Linear Fit: y = 1631.9x + 0.00786. R^2 = 0.999. ε (474 nm) = $\frac{1631.9\ M^{-1}}{0.2\ cm}$ ≈ 8200 $M^{-1}cm^{-1}$.

2.9 1-(pyridine)₂

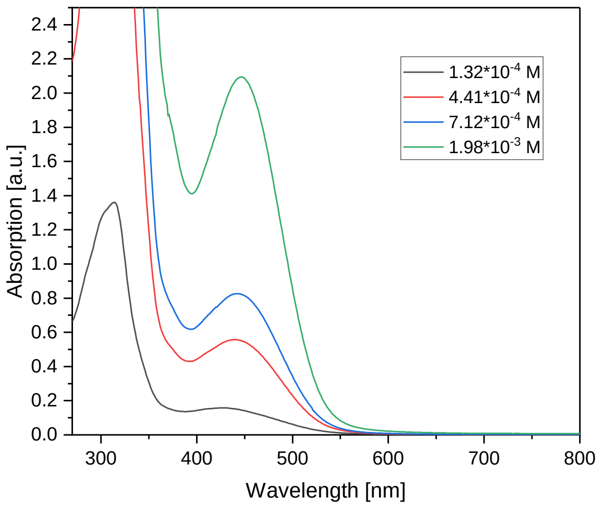

Figure S2.24. UV-vis absorption spectrum of **1-(pyridine)$_2$** ($1.32\cdot10^{-4}$ M, $4.41\cdot10^{-4}$ M, $7.12\cdot10^{-4}$ M, $1.98\cdot10^{-3}$ M DCM). λ_{max} (c = $1.32\cdot10^{-4}$ M) = 426 nm, λ_{max} (c = $4.41\cdot10^{-4}$ M) = 439 nm, λ_{max} (c = $7.12\cdot10^{-4}$ M) = 443 nm, λ_{max} (c = $1.98\cdot10^{-3}$ M) = 448 nm.

Stock solution:

1.9 mg **1-(pyridine)$_2$** in 1000 µL DCM.

Cuvette: d = 2 mm, V = 700 µL

Aliquots: 50 µL, 200 µL, 400 µL, stock solution

Concentrations:

i) $\frac{\left(\frac{0.0019g}{958\ g/mol}\right)}{0.001\ l}\cdot\frac{50\ \mu L}{750\ \mu L} = 1.322\cdot10^{-4}\ M$

ii) $\frac{\left(\frac{0.0019g}{958\ g/mol}\right)}{0.001\ l}\cdot\frac{200\ \mu L}{900\ \mu L} = 4.407\cdot10^{-4}\ M$

iii) $\frac{\left(\frac{0.0019g}{958g/mol}\right)}{0.001\ l}\cdot\frac{400\ \mu L}{1100\ \mu L} = 7.120\cdot10^{-4}\ M$

iv) $\frac{\left(\frac{0.0019g}{958g/mol}\right)}{0.001\ l} = 1.983\cdot10^{-3}\ M$

The absorption spectrum (λ_{max}) of **1-pyridine** shows significant concentration dependence (cf. figure S2.24), suggesting dissociation at low concentrations. Therefore, additional equivalents of pyridine were added to ensure complete conversion to the pentacoordinated silicon complex.

Due to partial formation of the bis-adduct, a low energy shoulder appears at high pyridine concentrations. However, the influence of this formation on λ_{max} is minimal, as no change in λ_{max} was observed between 10 and 20 equivalents of pyridine.

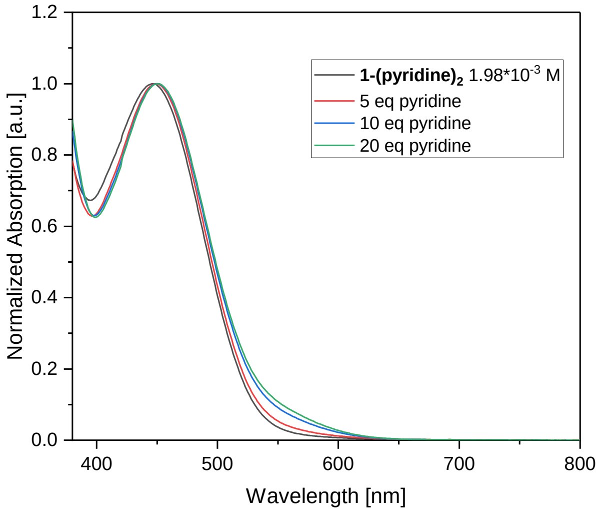

Figure S2.25. Normalised UV-vis absorption spectrum of **1-(pyridine)$_2$** ($1.98\cdot10^{-3}$ M; $6.26\cdot10^{-4}$ M +5/10/20 eq. pyridine, normalised). A shift of the spectrum is observed when adding 5 equivalents of pyridine, but no further change of λ_{max} (452 nm) with 10 and 20 equivalents pyridine. A low-energy shoulder appears due to partial formation of the bis-adduct at high pyridine concentrations.

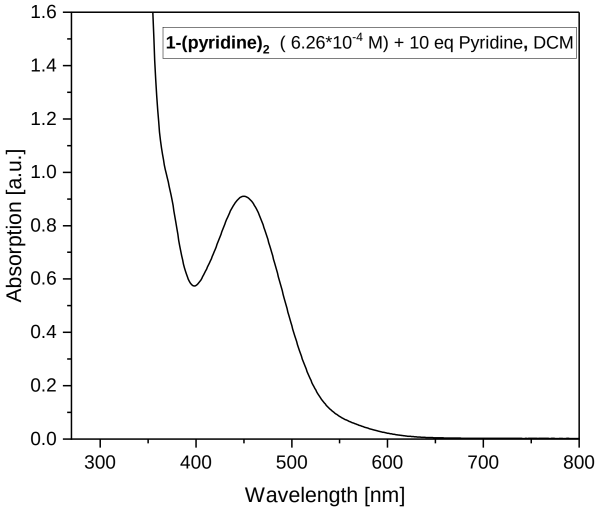

Figure S2.26. UV-vis absorption spectrum of **1-(pyridine)$_2$** ($6.26\cdot10^{-4}$ M + 10 eq pyridine, DCM). ε (452 nm) = $\frac{0.90998}{6.26\cdot10^{-4}\ M\cdot0.2\ cm}\approx 7300\ M^{-1}cm^{-1}$.

2.10 1-DMAP

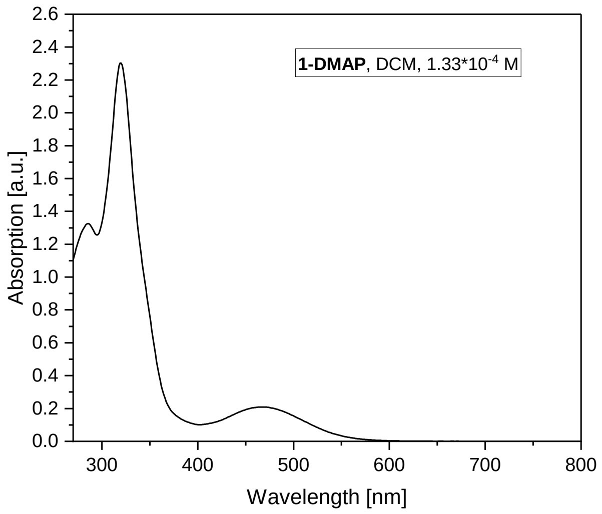

Figure S2.27. UV-vis absorption spectrum of **1-DMAP** (1.33·10^{-4} M, DCM).

Stock solution:

2.0 mg **1-DMAP** in 1000 µL DCM.

<u>Cuvette</u>: d = 2 mm, V = 700 µL

<u>Aliquots</u>: 50 µL, 100 µL, 200 µL

Concentrations:

i) $$\frac{\left(\frac{0.0020g}{1001\ g/mol}\right)}{0.001\ l} \cdot \frac{50\ \mu L}{750\ \mu L} = 1.332 \cdot 10^{-4}\ M$$

ii) $$\frac{\left(\frac{0.0020g}{1001\ g/mol}\right)}{0.001\ l} \cdot \frac{100\ \mu L}{800\ \mu L} = 2.498 \cdot 10^{-4}\ M$$

iii) $$\frac{\left(\frac{0.0020g}{1001\ g/mol}\right)}{0.001\ l} \cdot \frac{200\ \mu L}{900\ \mu L} = 4.440 \cdot 10^{-4}\ M$$

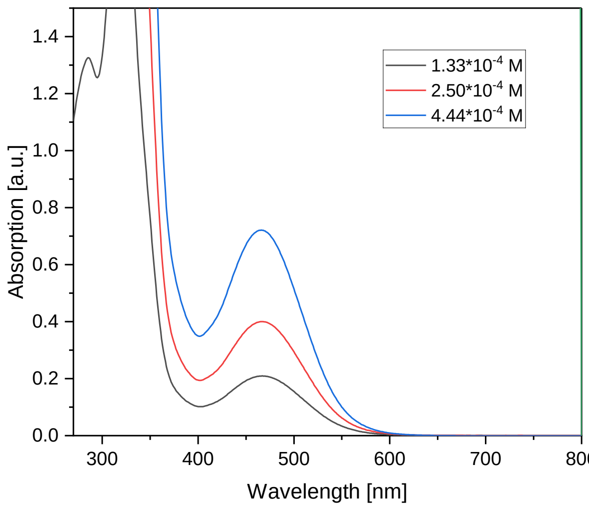

Figure S2.28. UV-vis absorption spectrum of **1-DMAP** (1.33·10^{-4} M, 2.50·10^{-4} M, 4.44·10^{-4} M, DCM). No change of λ_{max} is observed at different concentrations, suggesting negligible dissociation at low concentrations.

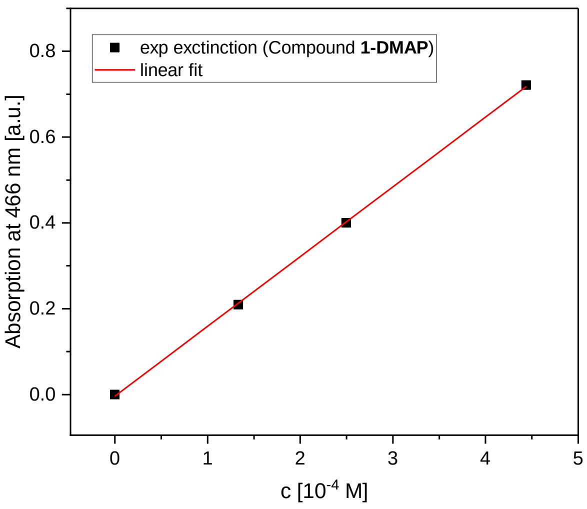

Figure S2.29. Extinction at 466 nm (CT band) of **1-DMAP** (0 M, 1.33·10^{-4} M, 2.50·10^{-4} M, 4.44·10^{-4} M, DCM). Linear Fit: y = 1626.2x - 0.00369. R^2 = 0.999. ε (474 nm) = $\frac{1626.2\ M^{-1}}{0.2\ cm}$ ≈ 8100 $M^{-1}cm^{-1}$.

2.11 1-OPEt$_3$

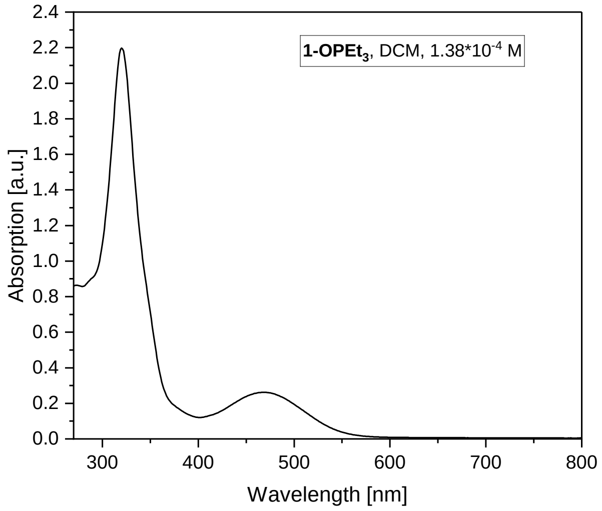

Figure S2.30. UV-vis absorption spectrum of **1-OPEt$_3$** (1.38·10^{-4} M, DCM).

Stock solution:

2.1 mg **1-OPEt$_3$** in 1000 µL DCM.

<u>Cuvette</u>: d = 2 mm, V = 700 µL

<u>Aliquots</u>: 50 µL, 100 µL, 200 µL

Concentrations:

i) $$\frac{\left(\frac{0.0021g}{1012\ g/mol}\right)}{0.001\ l} \cdot \frac{50\ \mu L}{750\ \mu L} = 1.383 \cdot 10^{-4}\ M$$

ii) $$\frac{\left(\frac{0.0021g}{1012\ g/mol}\right)}{0.001\ l} \cdot \frac{100\ \mu L}{800\ \mu L} = 2.594 \cdot 10^{-4}\ M$$

iii) $$\frac{\left(\frac{0.0021g}{1012\ g/mol}\right)}{0.001\ l} \cdot \frac{200\ \mu L}{900\ \mu L} = 4.611 \cdot 10^{-4}\ M$$

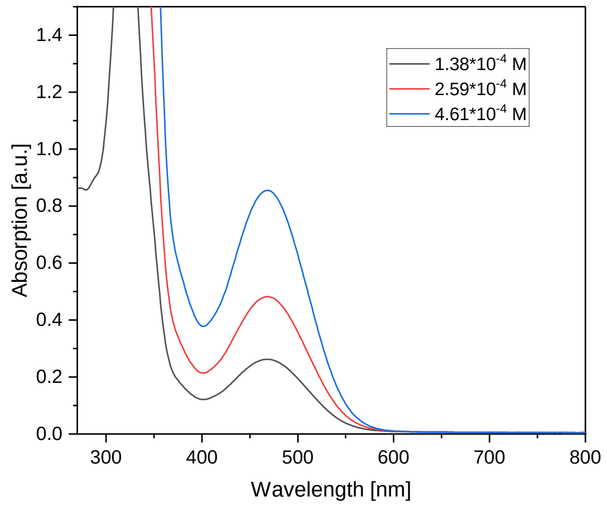

Figure S2.31. UV-vis absorption spectrum of **1-OPEt$_3$** (1.38·10^{-4} M, 2.59·10^{-4} M, 4.61·10^{-4} M, DCM). No change of λ_{max} is observed at different concentrations, suggesting negligible dissociation at low concentrations.

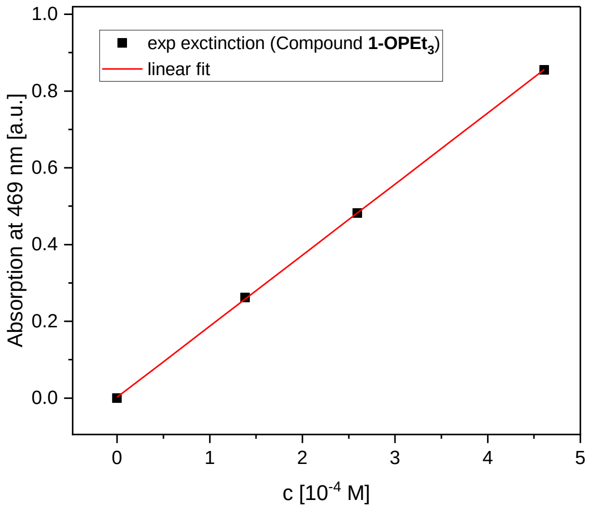

Figure S2.32. Extinction at 469 nm (CT band) of **1-OPEt$_3$** (0 M, 1.38·10^{-4} M, 2.59·10^{-4} M, 4.61·10^{-4} M, DCM). Linear Fit: y = 1851.1x + 0.0023. R^2 = 0.999. ε (474 nm) = $\frac{1851.1\ M^{-1}}{0.2\ cm}$ ≈ 9300 M^{-1}cm^{-1}.

2.12 1-DMSO

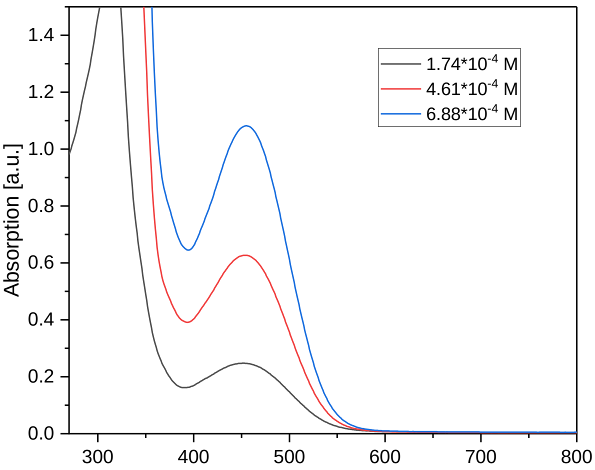

Figure S2.33. UV-vis absorption spectrum of **1-DMSO** ($1.74\cdot10^{-4}$ M, $4.61\cdot10^{-4}$ M, $6.88\cdot10^{-4}$ M, DCM). λ_{max} (c = $1.74\cdot10^{-4}$ M) = 452 nm, λ_{max} (c = $4.61\cdot10^{-4}$ M) = 454 nm, λ_{max} (c = $6.88\cdot10^{-4}$ M) = 456 nm.

Stock solution:

2.5 mg **1-DMSO** in 1000 µL DCM.

<u>Cuvette</u>: d = 2 mm, V = 700 µL

<u>Aliquots</u>: 50 µL, 150 µL, 250 µL

Concentrations:

i) $$\frac{\left(\frac{0.0025g}{956\ g/mol}\right)}{0.001\ l}\cdot\frac{50\ \mu L}{750\ \mu L}=1.743\cdot10^{-4}\ M$$

ii) $$\frac{\left(\frac{0.0025g}{956\ g/mol}\right)}{0.001\ l}\cdot\frac{150\ \mu L}{850\ \mu L}=4.615\cdot10^{-4}\ M$$

iii) $$\frac{\left(\frac{0.0025g}{956\ g/mol}\right)}{0.001\ l}\cdot\frac{250\ \mu L}{950\ \mu L}=6.881\cdot10^{-4}\ M$$

Due to dissociation at low concentrations (concentration dependence of λ_{max}, figure S2.33), additional equivalents of DMSO were added to obtain the spectrum of the fully associated complex.

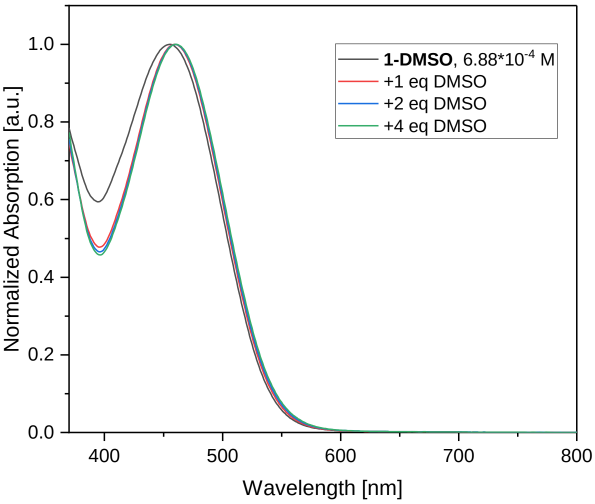

Figure S2.34. Normalised UV-vis absorption spectrum of **1-DMSO** ($6.88\cdot10^{-4}$ M, +1/2/4 eq. DMSO, DCM). No significant further change of the absorption spectrum observed between 2 and 4 equivalents of DMSO.

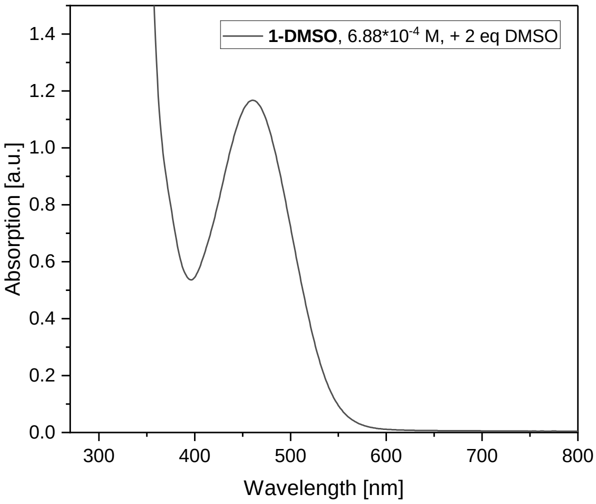

Figure S2.35. UV-vis absorption spectrum of **1-DMSO** ($6.88\cdot10^{-4}$ M + 2 eq DMSO, DCM). ε (460 nm) = $\frac{1.16703}{6.88\cdot10^{-4}\ M\cdot0.2\ cm}\approx 8500\ M^{-1}cm^{-1}$.

2.13 1-DIBA

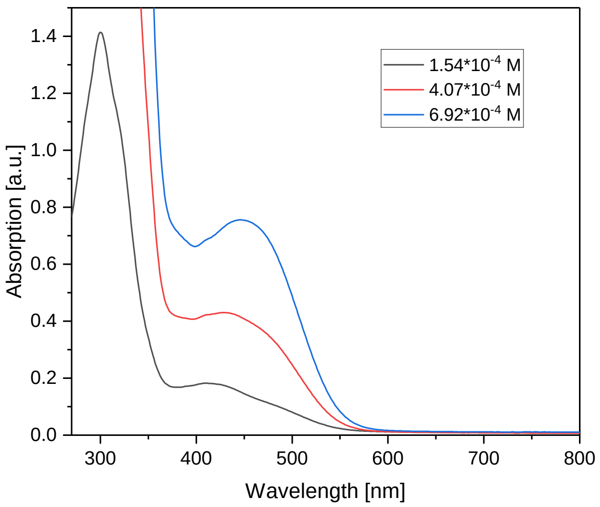

Figure S2.36. UV-vis absorption spectrum of **1-DIBA** ($1.54\cdot10^{-4}$ M, $4.07\cdot10^{-4}$ M, $6.92\cdot10^{-4}$ M, DCM). λ_{max} (c = $1.54\cdot10^{-4}$ M) = 411 nm, λ_{max} (c = $4.07\cdot10^{-4}$ M) = 430 nm, λ_{max} (c = $6.92\cdot10^{-4}$ M) = 446 nm.

Stock solution:

2.5 mg **1-DIBA** in 1000 µL DCM.

Cuvette: d = 2 mm, V = 700 µL

Aliquots: 50 µL, 150 µL, 300 µL

Concentrations:

i) $$\frac{\left(\frac{0.0025g}{1084\ g/mol}\right)}{0.001\ l}\cdot\frac{50\ \mu\text{L}}{750\ \mu\text{L}} = 1.538\cdot10^{-4}\ M$$

ii) $$\frac{\left(\frac{0.0025g}{1084\ g/mol}\right)}{0.001\ l}\cdot\frac{150\ \mu\text{L}}{850\ \mu\text{L}} = 4.070\cdot10^{-4}\ M$$

iii) $$\frac{\left(\frac{0.0025g}{1084\ g/mol}\right)}{0.001\ l}\cdot\frac{300\ \mu\text{L}}{1000\ \mu\text{L}} = 6.919\cdot10^{-4}\ M$$

Due to significant dissociation at low concentrations (concentration dependence of λ_{max}, figure S2.36), additional equivalents of DIBA were added to obtain the spectrum of the fully associated complex.

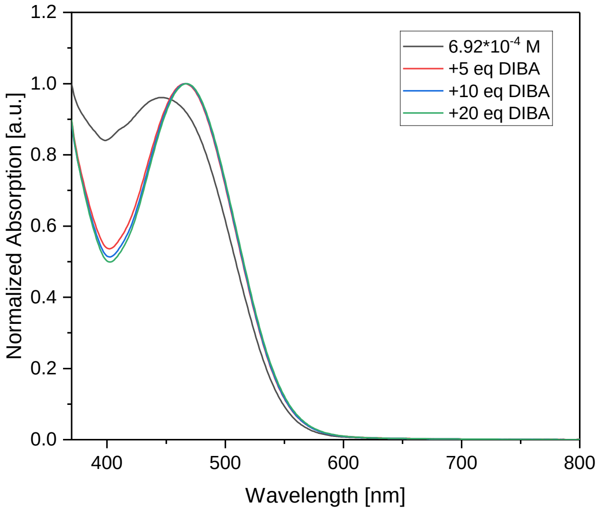

Figure S2.37. Normalised UV-vis absorption spectrum of **1-DIBA** ($6.92\cdot10^{-4}$ M, +5/10/20 eq. DIBA, DCM). No significant further change of the absorption spectrum observed between 10 and 20 equivalents of DIBA.

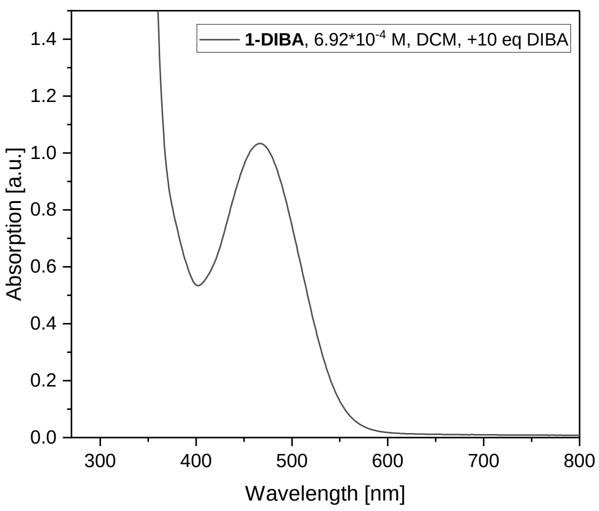

Figure S2.38. UV-vis absorption spectrum of **1-DIBA** ($6.92\cdot10^{-4}$ M + 10 eq DIBA, DCM). ε (467 nm) = $\frac{1.03329}{6.92\cdot10^{-4}\ M\cdot0.2\ cm}\approx 7500\ M^{-1}cm^{-1}$.

2.14 1-^{dipp}NHC

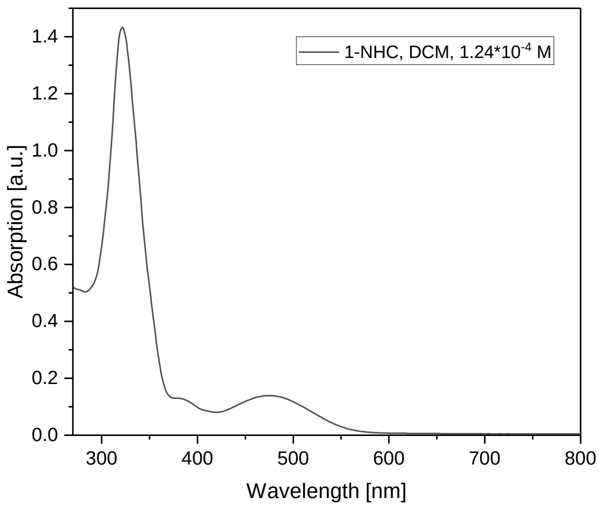

Figure S2.39. UV-vis absorption spectrum of **1-^{dipp}NHC** (1.24·10^{-4} M, DCM).

Stock solution:

2.4 mg **1-^{dipp}NHC** in 1000 µL DCM.

<u>Cuvette</u>: d = 2 mm, V = 700 µL

<u>Aliquots</u>: 50 µL, 150 µL, 250 µL

Concentrations:

i) $$\frac{\left(\frac{0.0024 g}{1296\ g/mol}\right)}{0.001\ l} \cdot \frac{50\ \mu L}{750\ \mu L} = 1.235 \cdot 10^{-4}\ M$$

ii) $$\frac{\left(\frac{0.0024 g}{1296\ g/mol}\right)}{0.001\ l} \cdot \frac{150\ \mu L}{850\ \mu L} = 3.268 \cdot 10^{-4}\ M$$

iii) $$\frac{\left(\frac{0.0024 g}{1296\ g/mol}\right)}{0.001\ l} \cdot \frac{250\ \mu L}{1000\ \mu L} = 4.630 \cdot 10^{-4}\ M$$

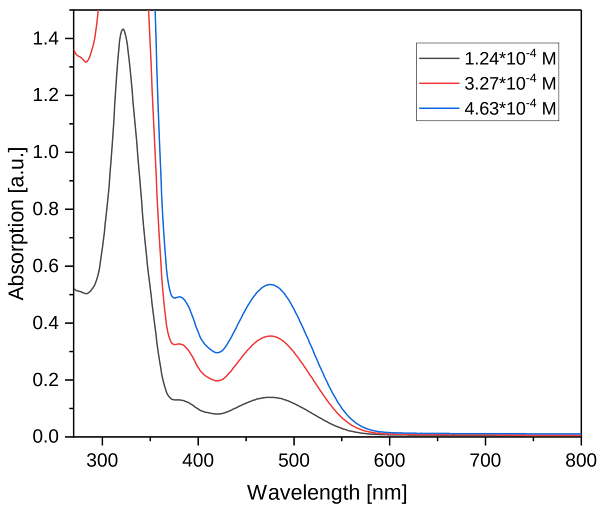

Figure S2.40. UV-vis absorption spectrum of **1-^{dipp}NHC** (1.24·10^{-4} M, 3.27·10^{-4} M, 4.63·10^{-4} M, DCM). No change of λ_{max} is observed at different concentrations, suggesting negligible dissociation at low concentrations.

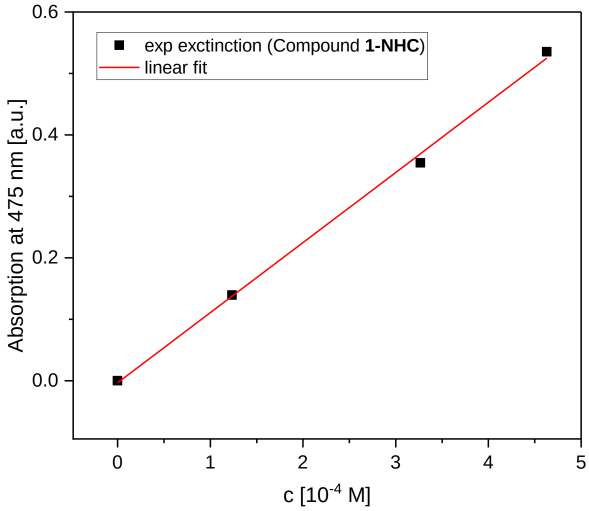

Figure S2.41. Extinction at 475 nm (CT band) of **1-^{dipp}NHC** (0 M, 1.24·10^{-4} M, 3.27·10^{-4} M, 4.63·10^{-4} M, DCM). Linear Fit: y = 1140.9x - 0.00323. R^2 = 0.997. ε (475 nm) = $\frac{1140.9\ M^{-1}}{0.2\ cm}$ ≈ 5700 M^{-1}cm^{-1}.

2.15 [1-Br][NBu4]

The bromide adduct of **1** could not be isolated, but UV-vis spectra could be measured with a large excess of NBu_4Br. A $2.3·10^{-4}$ M of **1** was prepared, and NBu_4Br was added as a solid until no further changes were observed, indicating quantitative conversion to **[1-Br][NBu4]**.

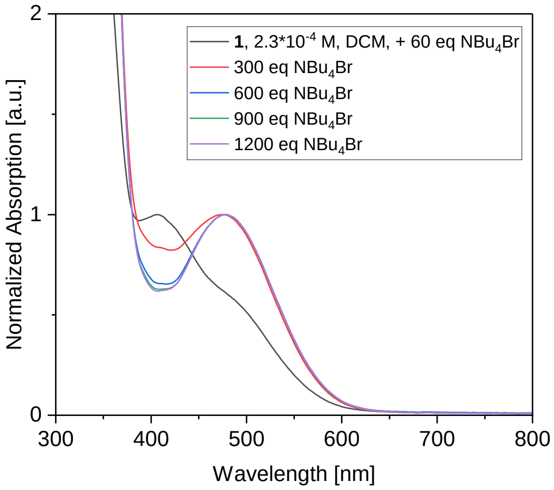

Figure S2.42. Normalised UV-vis absorption spectrum of **1** ($2.3·10^{-4}$ M, +60/300/600/900/1200 eq. NBu_4Br, DCM). No significant further change of the absorption spectrum observed between 900 and 1200 equivalents of NBu_4Br. λ_{max}= 479 nm.

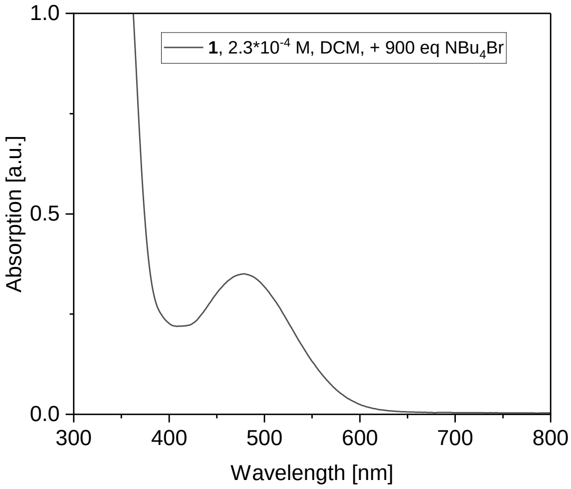

Figure S2.43. UV-vis absorption spectrum of **[1-Br][NBu4]** ($2.3·10^{-4}$ M + 900 eq NBu_4Br, DCM). ε (479 nm) = $\frac{0.3503}{2.3·10^{-4}\ M \cdot 0.2\ cm} \approx 7600\ M^{-1}cm^{-1}$.

2.16 1-P(*n*Bu)$_3$

1-P(*n*Bu)$_3$ could not be isolated. However, the UV-vis spectrum could be measured by using a large excess of tributylphosphine. To a 5.5·10^{-4} M solution of **1** in DCM were added variable amounts of tributylphosphine. With 150 equivalents, quantitative conversion to 1-P(*n*Bu)$_3$ was observed.

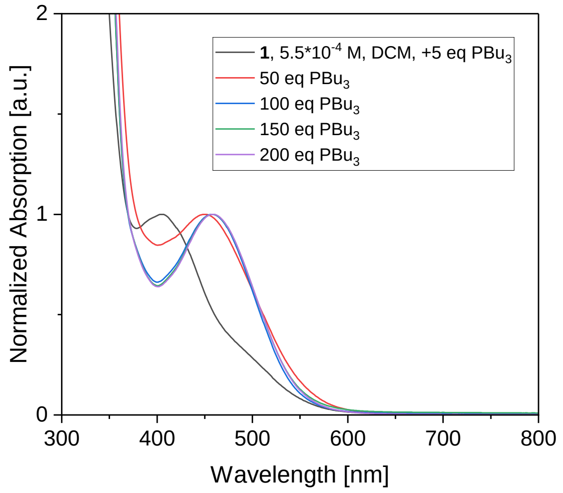

Figure S2.44. Normalised UV-vis absorption spectrum of **1** (5.5 ·10^{-4} M, +5/50/100/150/200 eq. P(*n*Bu)$_3$, DCM). No significant further change of the absorption spectrum observed between 150 and 200 equivalents of P(*n*Bu)$_3$. λ_{max} = 458 nm.

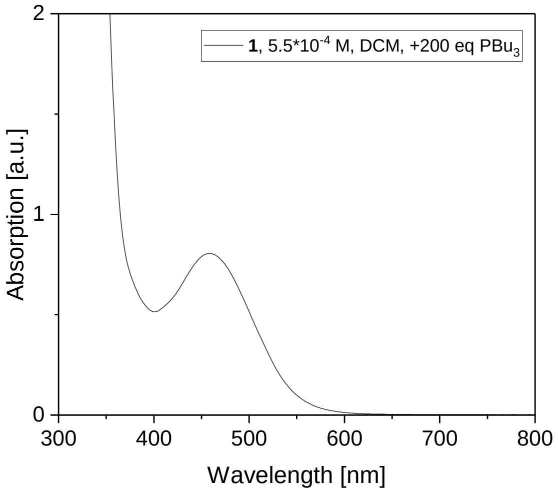

Figure S2.45. UV-vis absorption spectrum of **1-P(*n*Bu)$_3$** (5.5·10^{-4} M + 200 eq P(*n*Bu)$_3$, DCM). ε (458 nm) = $\frac{0.8052}{5.5 \cdot 10^{-4}\, M \cdot 0.2\, cm} \approx 7300$ M^{-1}cm^{-1}.

2.17 1-DABCO

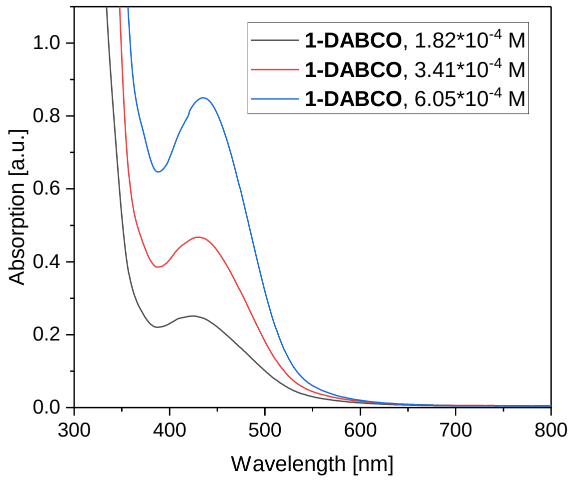

Figure S2.46. UV-vis absorption spectrum of **1-DABCO** (1.82·10^{-4} M, 3.41·10^{-4} M, 6.05·10^{-4} M, DCM). λ_{max} (c = 1.82·10^{-4} M) = 424 nm, λ_{max} (c = 3.41·10^{-4} M) = 430 nm, λ_{max} (c = 6.05·10^{-4} M) = 435 nm.

Stock solution:

2.7 mg **1-DABCO** in 1000 µL DCM.

Cuvette: d = 2 mm, V = 700 µL

Aliquots: 50 µL, 100 µL, 200 µL

Concentrations:

i) $$\frac{\left(\frac{0.0027g}{991\ g/mol}\right)}{0.001\ l} \cdot \frac{50\ \mu\text{L}}{750\ \mu\text{L}} = 1.816 \cdot 10^{-4}\ M$$

ii) $$\frac{\left(\frac{0.0027g}{991\ g/mol}\right)}{0.001\ l} \cdot \frac{100\ \mu\text{L}}{800\ \mu\text{L}} = 3.406 \cdot 10^{-4}\ M$$

iii) $$\frac{\left(\frac{0.0027g}{991\ g/mol}\right)}{0.001\ l} \cdot \frac{200\ \mu\text{L}}{900\ \mu\text{L}} = 6.054 \cdot 10^{-4}\ M$$

Due to dissociation at low concentrations (concentration dependence of λ_{max}, figure S2.46), additional equivalents of DABCO were added to obtain the spectrum of the fully associated complex.

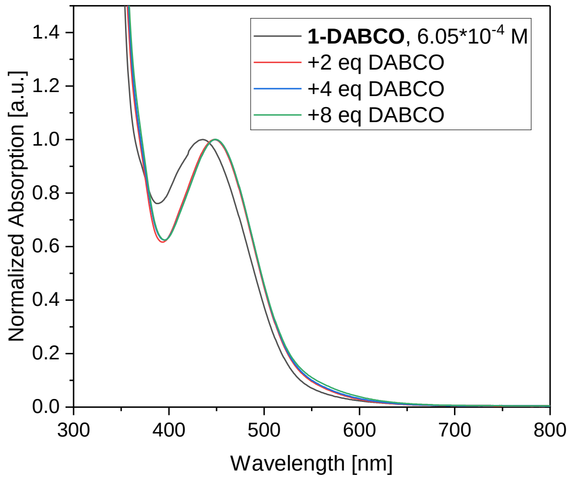

Figure S2.47. Normalised UV-vis absorption spectrum of **1-DABCO** (6.05 ·10^{-4} M, +2/4/8 eq. DABCO, DCM). No significant further change of the absorption spectrum observed between 4 and 8 equivalents of DABCO.

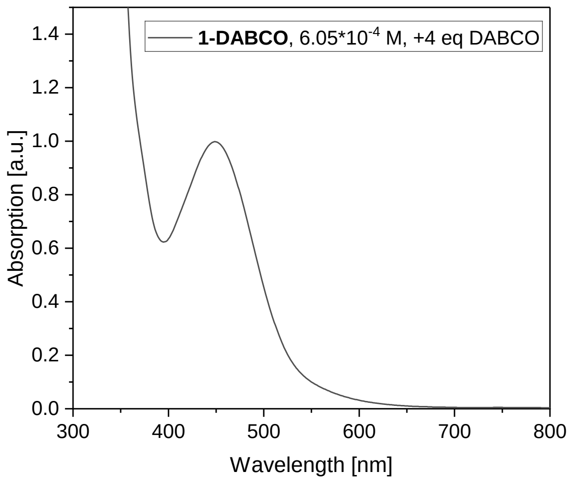

Figure S2.48. UV-vis absorption spectrum of **1-DABCO** (6.05·10^{-4} M + 2 eq DMSO, DCM). ε (449 nm) = $\frac{0.99803}{6.05 \cdot 10^{-4}\ M \cdot 0.2\ cm} \approx$ 8200 M^{-1}cm^{-1}.

2.18 1-^{iPr}NHC

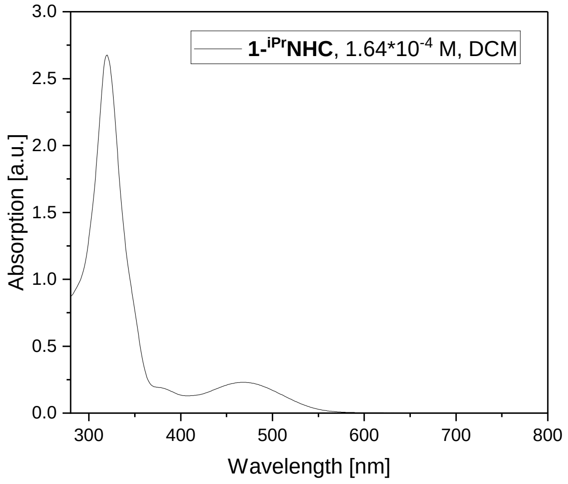

Figure S2.49. UV-vis absorption spectrum of **1-^{iPr}NHC** (1.64·10^{-4} M, DCM).

Stock solution:

2.6 mg **1-^{iPr}NHC** in 1000 µL DCM.

Cuvette: d = 2 mm, V = 700 µL

Aliquots: 50 µL, 100 µL, 200 µL

Concentrations:

i) $$\frac{\left(\frac{0.0026g}{1059\ g/mol}\right)}{0.001\ l}\cdot\frac{50\ \mu L}{750\ \mu L}=1.637\cdot 10^{-4}\ M$$

ii) $$\frac{\left(\frac{0.0026g}{1059\ g/mol}\right)}{0.001\ l}\cdot\frac{100\ \mu L}{800\ \mu L}=3.069\cdot 10^{-4}\ M$$

iii) $$\frac{\left(\frac{0.0026g}{1059\ g/mol}\right)}{0.001\ l}\cdot\frac{200\ \mu L}{900\ \mu L}=5.456\cdot 10^{-4}\ M$$

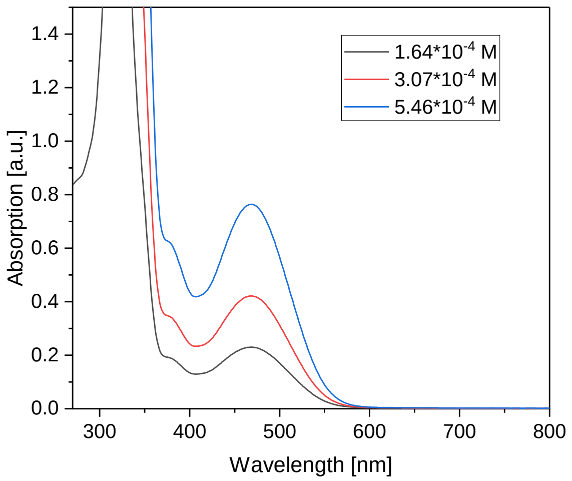

Figure S2.50. UV-vis absorption spectrum of **1-^{iPr}NHC** (1.64·10^{-4} M, 3.07·10^{-4} M, 5.46·10^{-4} M, DCM). No concentration-dependant dissociation was observed.

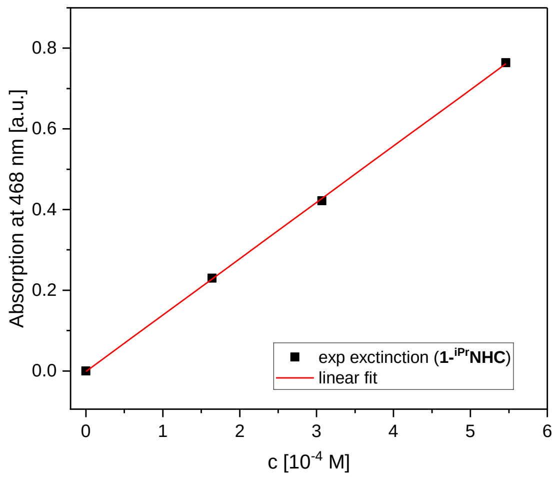

Figure S2.51. Extinction at 468 nm (CT band) of **1-^{iPr}NHC** (0 M, 1.64·10^{-4} M, 3.07·10^{-4} M, 5.46·10^{-4} M, DCM). Linear Fit: y = 1396.5x - 0.0012. R^2 = 0.999. ε (468 nm) = $\frac{1396.5\ M^{-1}}{0.2\ cm}$ ≈ 7000 $M^{-1}cm^{-1}$.

2.19 1-PCy$_3$

Due to dissociation of **1-PCy$_3$** in solution, the UV-vis spectrum of was measured by using a large excess of PCy$_3$. To a 3.9·10^{-4} M solution of **1-PCy$_3$** in DCM were added increasing amounts of PCy$_3$. With 120 equivalents, quantitative conversion to **1-PCy$_3$** was observed.

PCy$_3$ was purified by precipitation as its CS_2 adduct from diethylether, which was filtered off, washed with diethylether and heated to 70 °C under vacuum overnight to obtain oxide-free PCy$_3$. It was further purified by recrystallisation from diethylether at -40 °C. Even with these precautions, a low energy shoulder band appears due to the formation of the corresponding phosphine oxide adduct. However, this impurity was found to not impact the absorption maximum.

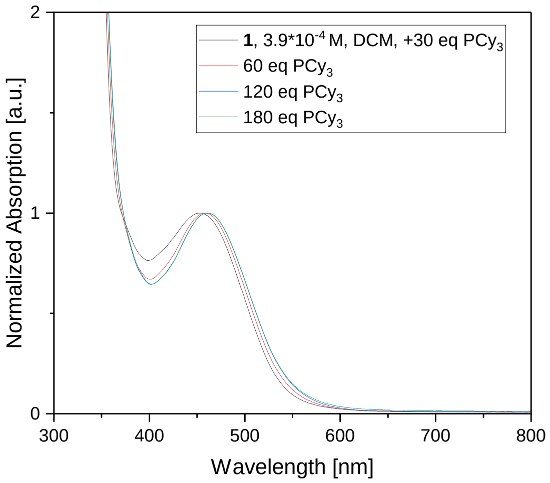

Figure S2.52. Normalised UV-vis absorption spectrum of **1-PCy$_3$** (3.9·10^{-4} M, +30/60/120/180 eq. PCy$_3$, DCM). No significant further change of the absorption spectrum observed between 120 and 180 equivalents of PCy$_3$. λ_{max} = 461 nm.

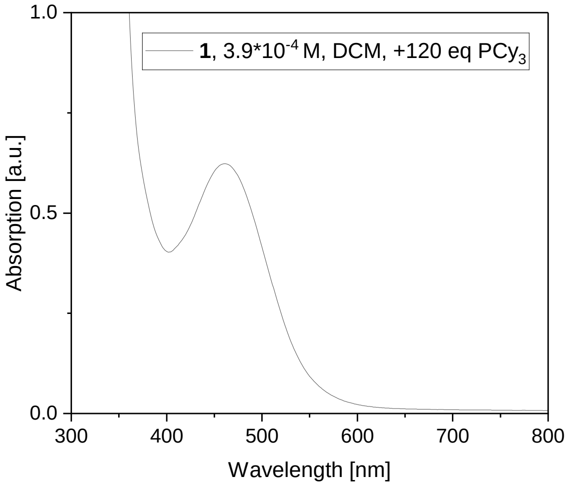

Figure S2.53. UV-vis absorption spectrum of **1-PCy$_3$** (3.9·10^{-4} M + 120 eq PCy$_3$, DCM). $\varepsilon\,(461\text{ nm}) = \frac{0.62318}{3.9\cdot10^{-4}\,M\cdot 0.2\,cm} \approx 8000\ M^{-1}cm^{-1}$.

2.20 1-SIMes

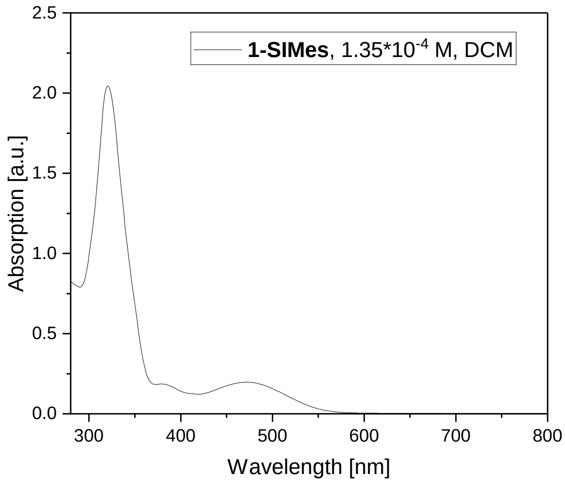

Figure S2.54. UV-vis absorption spectrum of **1-SIMes** (1.35·10⁻⁴ M, DCM).

Stock solution:

2.4 mg **1-SIMes** in 1000 µL DCM.

<u>Cuvette</u>: d = 2 mm, V = 700 µL

<u>Aliquots</u>: 50 µL, 100 µL, 200 µL

Concentrations:

i) $$\frac{\left(\frac{0.0024g}{1186\ g/mol}\right)}{0.001\ l}\cdot\frac{50\ \mu L}{750\ \mu L}=1.349\cdot10^{-4}\ M$$

ii) $$\frac{\left(\frac{0.0024g}{1186\ g/mol}\right)}{0.001\ l}\cdot\frac{100\ \mu L}{800\ \mu L}=2.530\cdot10^{-4}\ M$$

iii) $$\frac{\left(\frac{0.0024g}{1186\ g/mol}\right)}{0.001\ l}\cdot\frac{200\ \mu L}{900\ \mu L}=4.497\cdot10^{-4}\ M$$

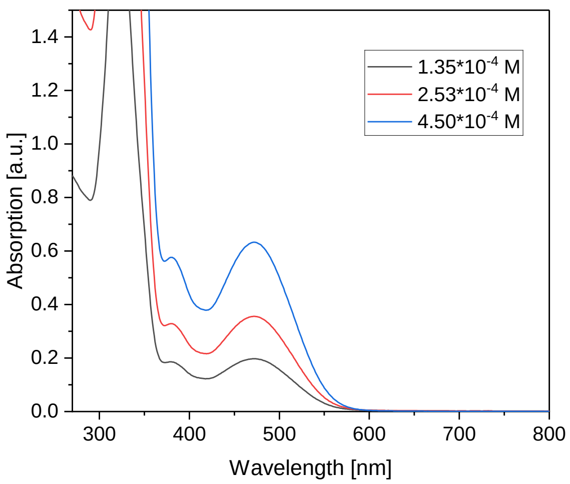

Figure S2.55. UV-vis absorption spectrum of **1-SIMes** (0 M, 1.35·10⁻⁴ M, 2.53·10⁻⁴ M, 4.50·10⁻⁴ M, DCM). No concentration-dependant dissociation was observed.

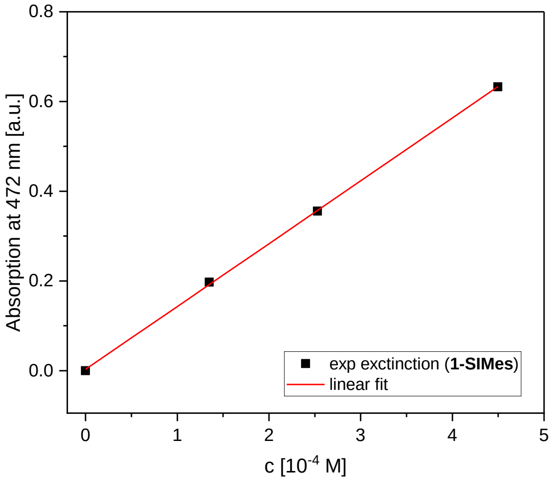

Figure S2.56. Extinction at 472 nm (CT band) of **1-SIMes** (0 M, 1.35·10⁻⁴ M, 2.53·10⁻⁴ M, 4.50·10⁻⁴ M, DCM). Linear Fit: y = 1401.0x - 0.0037. R^2 = 0.999. ε (468 nm) = $\frac{1401.0\ M^{-1}}{0.2\ cm}$ ≈ 7000 $M^{-1}cm^{-1}$.

2.21 2-((3,5-di-*tert*-butylphenyl)amino)-3-hydroxyanthraquinone (L_1)

The absorption spectrum of ligand 2-((3,5-di-*tert*-butylphenyl)amino)-3-hydroxyanthraquinone (**L_1**) was measured in THF due to low solubility in DCM.

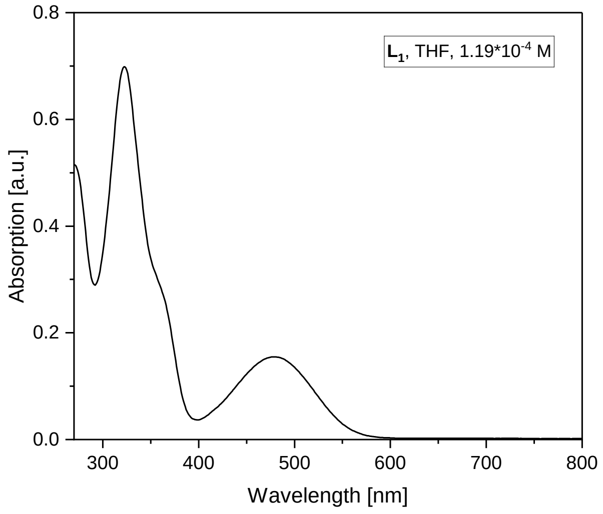

Figure S2.57. UV-vis absorption spectrum of **L_1** ($1.19 \cdot 10^{-4}$ M, THF).

Stock solution:

13.2 mg **L_1** in 20 ml THF.

Cuvette: d = 2 mm, V = 600 µL

Aliquots: 50 µL, 100 µL, 200 µL

Concentrations:

i) $\frac{\left(\frac{0.0132g}{427\ g/mol}\right)}{0.02\ l} \cdot \frac{50\ \mu L}{650\ \mu L} = 1.189 \cdot 10^{-4}\ M$

ii) $\frac{\left(\frac{0.0132g}{427\ g/mol}\right)}{0.02\ l} \cdot \frac{100\ \mu L}{700\ \mu L} = 2{,}208 \cdot 10^{-4}\ M$

iii) $\frac{\left(\frac{0.0132g}{427\ g/mol}\right)}{0.02\ l} \cdot \frac{200\ \mu L}{800\ \mu L} = 3.864 \cdot 10^{-4}\ M$

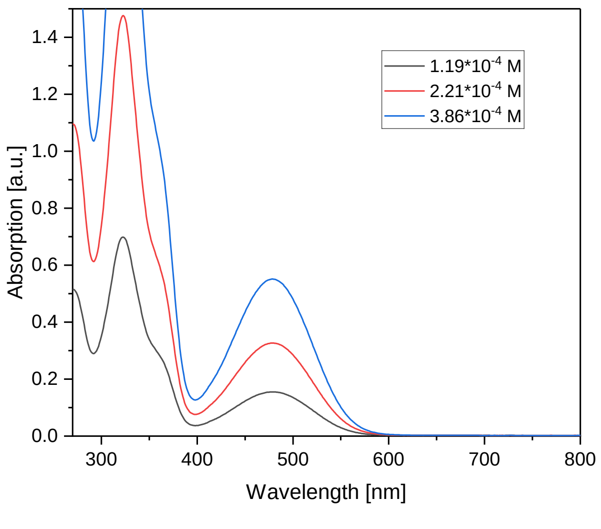

Figure S2.58. Normalised UV-vis absorption spectrum of **L_1** ($1.19 \cdot 10^{-4}$ M, $2.21 \cdot 10^{-4}$ M, $3.86 \cdot 10^{-4}$ M, THF).

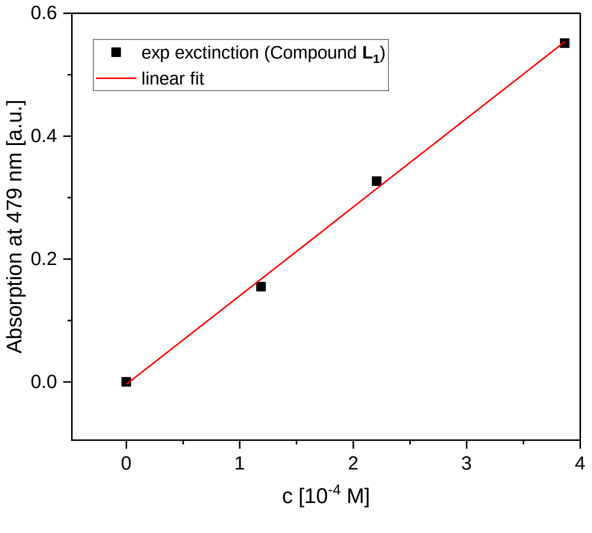

Figure S2.59. Extinction at 479 nm (CT band) of **L_1** (0 M, $1.19 \cdot 10^{-4}$ M, $2.21 \cdot 10^{-4}$ M, $3.86 \cdot 10^{-4}$ M, THF). Linear Fit: y = 1443.1x - 0.00386. R^2 = 0.997. ε (479 nm) = $\frac{1443.1\ M^{-1}}{0.2\ cm}$ ≈ 7200 $M^{-1}cm^{-1}$.

2.22 UV-vis titration of 1 with PPh_4Cl

To a solution of **1** in DCM (0.58 mM, 760 µL), 0.1 equivalents of PPh_4Cl were added in portions. A 12.5 mM solution of PPh_4Cl in DCM was prepared, of which 3.5 µL were added with each step. The absorption at 489 nm was fitted to a 1:1 binding isotherm with Musketeer 1.8.0,[1] which also provided the graphic for the fitted curve and the RMSD plot.

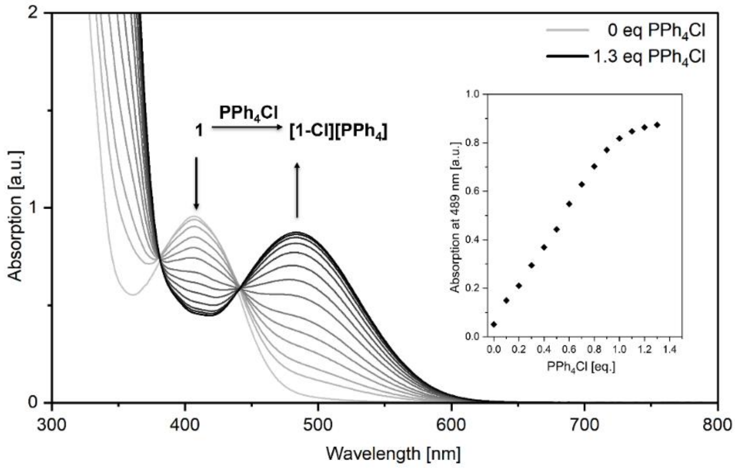

Figure S2.60. UV-vis titration of **1** (0.58 mM, DCM) with PPh_4Cl (12.5 mM, DCM) in steps of 0.1 equivalents PPh_4Cl (3.5 µL of stock solution).

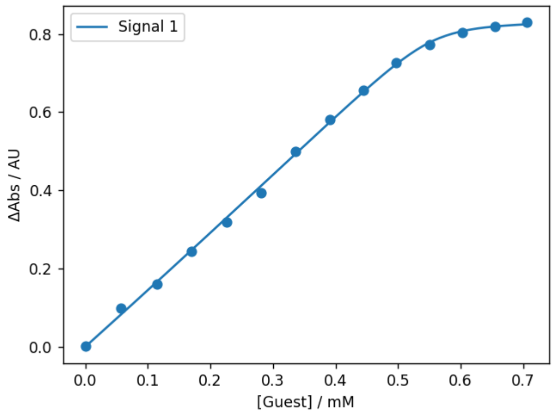

Figure S2.61. Binding isotherm for the absorption at 489 nm, fitted and plotted with *Musketeer 1.8.0*.[1] $K \approx 360000\ M^{-1}$.

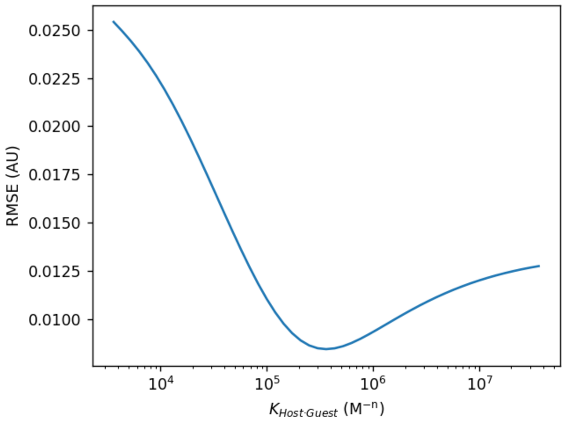

Figure S2.62. RMSD plot for the fitted curve.

1 + PPh_4Cl ⟶ [1-Cl][PPh_4]

$$K_c = \frac{[(1-Cl)(PPh_4)]}{[1][PPh_4Cl]} \approx 360000\ M^{-1}$$

3 Computational Section

3.1 General Information

All quantum chemical calculations were carried out with the Orca 5.0.3 program package.[3] The resources of JUSTUS2 (Ulm University) within the Baden-Württemberg High Performance Computing Program (bwHPC) were used. For all calculations, the RI approximation for the coulomb integral (RIJCOSX) together with the corresponding auxiliary basis sets were used.[4] Geometry Optimisations and subsequent frequency calculations were performed with the r^2scan-3c[5] composite method, confirming the final structures as energetic minima by the absence of imaginary frequencies. If possible, experimental solid-state structures were used as the initial structures for geometry optimisations. Single Point calculations for the calculation of FIA and HIA were performed at the DSD-BLYP(D3BJ)/def2-QZVPP[6, 7] level of theory, with DCM solvation accounted for with the COSMO-RS solvent model.[8] All other single point calculations were carried out at the DSD-BLYP(D3BJ)/def2-TZVPP[9] level of theory. Solvent corrected values were obtained with the SMD solvent model and DCM as solvent.[10] Gibbs free energy values were obtained from frequency calculations on the r^2scan-3c level of theory using the rigid rotor harmonic oscillator (RRHO) approximation.[11] Time-dependant calculations with the Tamm-Dancoff approximation (TDA)[12] were carried out with the long-range corrected hybrid functional ωB97X-D3,[13] the def2-TZVPP basis set, and DCM solvation correction with the SMD solvent model. The first 50 electronic transitions were calculated. Molecular Orbitals were visualised with IboView v20211019-RevA. NBO calculations were conducted on the PBE0[14]/def2-TZVPP[9] level of theory with NBO6.0.18_i4[15] as implemented in Orca 5.0.3. Natural Bond Orbitals were visualised with Chemcraft.

Inputs

<u>Geometry Optimsation</u>

```
! r2SCAN-3c RIJCOSX AutoAux TightSCF defgrid3 TightOPT FREQ
```

<u>Single Point Calculation (DCM)</u>

```
! DSD-BLYP D3BJ def2-TZVPP RIJCOSX AutoAux VeryTightSCF

%cpcm
smd true
SMDsolvent "CH2Cl2"
End
```

<u>NBO Calculation</u>

```
! RKS PBE0 D4 def2-TZVPP NBO VeryTightSCF
```

<u>TD-DFT (DCM)</u>

```
! UKS wB97X-D3 def2-TZVPP RIJCOSX AutoAux TightSCF Engrad KeepDens Largeprint

%cpcm
smd true
SMDsolvent "CH2Cl2"
End

%tddft
  maxdim 5
  nroots 50
end
```

3.2 Fluoride and Hydride Ion Affinities

Fluoride and hydride ion affinities (FIA/HIA) were calculated utilizing isodesmic reactions with the TMS/TMSF and the TMS/TMSH anchor system, respectively, as proposed by *Krossing*[16] and *Greb*.[17,18] Solvent (DCM) enthalpy correction was applied using the COSMO-RS solvent model. The FIA and HIA values are given as the negative binding enthalpy (FIA/HIA = -ΔH(1 + X → 1-X)). For comparison, affinities for Si(am^{F}ph^F)$_2$,[19] Si(cat^{Cl})$_2$,[20] and B(C_6F_5)$_3$ (BCF) were calculated in addition to compound **1**.

Reaction 1

LA + TMSX → TMS^+ + LA-X^- ΔH (DSD-BLYP/def2-QZVPP//r^{2}scan-3c)

Reaction 2

TMSX → TMS^+ + X^- (X = F: ΔH = 952.5 kJ/mol[18]; X = H: ΔH = 924 kJ/mol[17])

FIA/HIA (vacuum) = ΔH(reaction 2) – ΔH(reaction 1)

FIA/HIA (DCM) = FIA/HIA(vacuum) - ΔH_{solv}(LA-X^-) + ΔH_{solv}(LA) + ΔH_{solv}(X^-)

Table S3.1. Computed energies for the calculation of ion affinities in vacuum (DSD-BLYP(D3BJ)/def2-TZVPP//r^{2}scan-3c) and in DCM (DSD-BLYP(D3BJ)/def2-TZVPP/COSMO-RS(DCM)//r^{2}scan-3c).

	**Charge/ multiplicity**	**Total Correction [kJ/mol]** r^{2}scan-3c	**Final Single Point Energy [kJ/mol]** DSD-BLYP/def2-QZVPP	**Final Enthalpy [kJ/mol]** DSD-BLYP/def2-TZVPP//r^{2}scan-3c	**Enthalpy Correction (DCM) [kJ/mol]** COSMO-RS(DCM)
**1**	0/1	2691.90	-7915827.75	-7913133.38	-154.83
**1-H**	-1/1	2710.86	-7917617.58	-7914904.24	-272.83
**1-F**	-1/1	2699.12	-8178350.42	-8175648.82	-277.98
**Si(am^{F}ph^F)$_2$**	0/1	670.96	-8558437.37	-8557763.92	-57.35
**Si(am^{F}ph^F)$_2$-H**	-1/1	689.45	-8560283.03	-8559591.11	-158.96
**Si(am^{F}ph^F)$_2$-F**	-1/1	677.61	-8821016.78	-8820336.69	-163.18
**Si(cat^{Cl})$_2$**	0/1	324.35	-12411413.54	-12411086.71	-74.59
**Si(cat^{Cl})$_2$-H**	-1/1	345.27	-12413251.91	-12412904.17	-196.96
**Si(cat^{Cl})$_2$-F**	-1/1	333.40	-12673986.2	-12673650.32	-199.67
**BCF**	0/1	478.66	-5795523.71	-5795042.57	-46.37
**BCF-H**	-1/1	500.09	-5797387.46	-5796884.90	-159.45
**BCF-F**	-1/1	484.17	-6058042.92	-6057556.27	-167.06
**TMS**	1/1	305.17	-1073010.79	-1072703.15	
**TMS-H**	0/1	330.35	-1075326.72	-1074993.90	
**TMS-F**	0/1	318.21	-1336052.36	-1335731.66	
**H**	-1/1				-204.91
**F**	-1/1				-343.05

Table S3.2. Computed ion affinities for **1**, Si(am^{F}ph^F)$_2$, Si(cat^{Cl})$_2$, and BCF.

**Lewis Acid**	**FIA(vacuum) [kJ/mol]**	**FIA(DCM) [kJ/mol]**	**HIA(vacuum) [kJ/mol]**	**HIA(DCM) [kJ/mol]**
**1**	439	220	404	317
**Si(am^{F}ph^F)$_2$**	497	260	460	357
**Si(cat^{Cl})$_2$**	488	270	451	368
**BCF**	438	215	476	384

3.3 Thermodynamics

Structures of mono- and bis-adducts were optimised as described above, and single point calculations were carried out with DCM corrections by the SMD solvent model. Geometry optimisation of **1-I$_2$, 1-(MeCN)$_2$, 1-(^{iPr}NHC)$_2$, 1-(SIMes)$_2$, 1-(PBu$_3$)$_2$, 1-DABCO and 1-(PCy$_3$)$_2$** resulted in dissociation of one of the donors, suggesting unstable bis-adducts. Convergence failed for **1-(DIBA)$_2$** and **1-(^{dipp}NHC)$_2$**.

Table S3.3. Computed energies for the calculation of thermodynamics of mono- and bis-adduct formation in DCM (DSD-BLYP(D3BJ)/def2-TZVPP/SMD(DCM)//r^{2}scan-3c).

	Charge/ multiplicity	Total Correction [kJ/mol] r^{2}scan-3c	Entropy Term (T*S) [kJ/mol] r^{2}scan-3c	Final Single Point Energy [kJ/mol] DSD-BLYP/def2-TZVPP/SMD(DCM)	Final Enthalpy [kJ/mol] DSD-BLYP/def2-TZVPP/SMD(DCM)// r^{2}scan-3c	Final Gibbs Free Energy [kJ/mol] DSD-BLYP/def2-TZVPP/SMD(DCM)/ / r^{2}scan-3c
**1**	0/1	2691.93	376.26	-7914781.73	-7912087.31	-7912463.57
**1-F**	-1/1	2699.03	379.85	-8177358.41	-8174656.90	-8175036.76
**1-Cl**	-1/1	2696.91	383.29	-9122968.37	-9120268.98	-9120652.27
**1-Br**	-1/1	2696.55	385.63	-14671962.86	-14669263.83	-14669649.46
**1-I**	-1/1	2696.27	388.32	-8696161.48	-8693462.74	-8693851.06
**1-N$_3$**	-1/1	2733.09	390.43	-8346033.55	-8343297.98	-8343688.42
**1-NCS**	-1/1	2728.38	393.46	-9203773.16	-9201042.29	-9201435.76
**1-CN**	-1/1	2717.19	386.42	-8158754.49	-8156034.82	-8156421.24
**1-MeCN**	0/1	2827.27	403.48	-8263073.81	-8260244.06	-8260647.54
**1-Et$_2$O**	0/1	3076.11	412.18	-8527759.11	-8524680.52	-8525092.69
**1-THF**	0/1	3019.52	405.30	-8524607.24	-8521585.23	-8521990.53
**1-pyridine**	0/1	2945.12	403.87	-8566161.20	-8563213.61	-8563617.48
**1-DMAP**	0/1	3147.53	427.36	-8917621.67	-8914471.66	-8914899.02
**1-OPEt$_3$**	0/1	3263.32	436.42	-9631538.71	-9628272.92	-9628709.34
**1-HMPA**	0/1	3406.33	459.28	-10067234.50	-10063825.69	-10064284.97
**1-DMSO**	0/1	2921.68	407.80	-9366471.80	-9363547.64	-9363955.44
**1-DIBA**	0/1	3517.51	454.65	-9585361.54	-9581841.55	-9582296.20
**1-^{dipp}NHC**	0/1	4422.81	535.51	-11164084.27	-11159658.98	-11160194.49
**1-SIMes**	0/1	3862.91	484.08	-10342140.89	-10338484.84	-10338968.92
**1-^{iPr}NHC**	0/1	3514.62	448.98	-9333247.78	-9329730.69	-9330179.67
**1-P(nBu)$_3$**	0/1	3715.98	463.75	-10052452.89	-10048924.30	-10049388.05
**1-PCy$_3$**	0/1	4013.84	470.01	-10661711.95	-10657903.00	-10658373.01
**1-DABCO**	0/1	3198.77	405.74	-8820534.58	-8817536.44	-8817942.18
**1-F$_2$**	-2/1	2701.60	386.58	-8439760.53	-8437056.45	-8437443.04
**1-Cl$_2$**	-2/1	2699.10	394.95	-10331036.73	-10328335.15	-10328730.10
**1-Br$_2$**	-2/1	2698.78	401.43	-21429036.42	-21426335.16	-21426736.59
**1-I$_2$**	-2/1					
**1-(N$_3$)$_2$**	-2/1	2769.67	409.78	-8777157.85	-8774385.70	-8774795.48
**1-(NCS)$_2$**	-2/1	2760.83	416.38	-10492655.43	-10489892.12	-10490308.50
**1-(CN)$_2$**	-2/1	2738.81	401.48	-8402614.55	-8399873.26	-8400274.74
**1-(MeCN)$_2$**	0/1					
**1-(Et$_2$O)$_2$**	0/1	3460.67	447.66	-9140713.18	-9137250.04	-9137697.70
**1-(THF)$_2$**	0/1	3346.18	434.43	-9134437.95	-9131089.29	-9131523.72
**1-(pyridine)$_2$**	0/1	3196.91	429.12	-9217523.30	-9214323.91	-9214753.04
**1-(DMAP)$_2$**	0/1	3598.69	471.51	-9920429.19	-9916828.02	-9917299.53
**1-(OPEt$_3$)$_2$**	0/1	3832.24	497.20	-11348211.94	-11344377.22	-11344874.42
**1-(HMPA)$_2$**	0/1	4118.13	532.80	-12219622.14	-12215501.53	-12216034.34
**1-(DMSO)$_2$**	0/1	3150.04	444.00	-10818122.56	-10814970.04	-10815414.04
**1-(DIBA)$_2$**	0/1					
**1-(^{dipp}NHC)$_2$**	0/1					
**1-(SIMes)$_2$**	0/1					
**1-(^{iPr}NHC)$_2$**	0/1					
**1-(P(nBu)$_3$)$_2$**	0/1					
**1-(PCy$_3$)$_2$**	0/1					
**1-(DABCO)$_2$**	0/1					
**F**	-1/1	3.72	43.37	-262338.92	-262332.73	-262376.10
**Cl**	-1/1	3.72	45.69	-1208090.35	-1208084.16	-1208129.85
**Br**	-1/1	3.72	48.71	-6757090.16	-6757083.96	-6757132.67
**I**	-1/1	3.72	50.43	-781357.71	-781351.51	-781401.94
**N$_3$**	-1/1	35.55	63.30	-431123.49	-431085.46	-431148.76
**NCS**	-1/1	28.34	51.40	-1288879.35	-1288848.53	-1288899.93
**CN**	-1/1	18.92	58.66	-243851.12	-243829.72	-243888.38
**MeCN**	0/1	127.66	72.33	-348270.81	-348140.67	-348213.00

**Et_2O**	0/1	373.99	98.93	-612936.36	-612559.89	-612658.82
**THF**	0/1	318.33	88.73	-609773.99	-609453.17	-609541.91
**Pyridine**	0/1	243.07	84.03	-651291.01	-651045.47	-651129.49
**DMAP**	0/1	444.67	111.79	-1002724.45	-1002277.29	-1002389.08
**$OPEt_3$**	0/1	561.89	127.63	-1716634.91	-1716070.54	-1716198.18
**HMPA**	0/1	703.96	148.44	-2152323.02	-2151616.58	-2151765.03
**DMSO**	0/1	221.42	92.02	-1451602.32	-1451378.42	-1451470.44
**DIBA**	0/1	816.62	149.08	-1670474.17	-1669655.06	-1669804.14
**^{dipp}NHC**	0/1	1720.25	242.01	-3249104.24	-3247381.51	-3247623.52
**SIMes**	0/1	656.73	131.33	-1211978.69	-2426170.76	-2426366.86
**^{iPr}NHC**	0/1	811.34	146.62	-1418280.44	-1417466.63	-1417613.25
**$P(nBu)_3$**	0/1	1014.69	168.04	-2137702.66	-2136724.40	-2136892.44
**PCy_3**	0/1	1312.68	170.04	-2746943.38	-2745680.86	-2745850.90
**DABCO**	0/1	494.80	98.40	-905811.95	-905351.75	-905450.15

Table S3.4. Computed thermodynamics of mono- and bis-adduct formation of **1** with donors **X** in DCM (DSD-BLYP(D3BJ)/def2-TZVPP/SMD(DCM)//r2scan-3c).

**X**	**ΔH (DCM) [kJ/mol]**<br>**1 + X → 1-X**	**ΔG (DCM) [kJ/mol]**<br>**1 + X → 1-X**	**ΔH (DCM) [kJ/mol]**<br>**1-X + X → 1-X_2**	**ΔG (DCM) [kJ/mol]**<br>**1-X + X → 1-X_2**
**F-**	-236.9	-197.1	-66.8	-30.2
**Cl-**	-97.5	-58.9	18.0	52.0
**Br-**	-92.6	-53.2	12.6	45.5
**I-**	-23.9	14.5		
**N_3-**	-125.2	-76.1	-2.3	41.7
**NCS-**	-106.5	-72.3	-1.3	27.2
**CN-**	-117.8	-69.3	-8.7	34.9
**MeCN**	-16.1	29.0		
**Et_2O**	-33.3	29.7	-9.6	53.8
**THF**	-44.7	14.9	-50.9	8.7
**Pyridine**	-80.8	-24.4	-64.8	-6.1
**DMAP**	-107.1	-46.4	-79.1	-11.4
**$OPEt_3$**	-115.1	-47.6	-33.8	33.1
**HMPA**	-121.8	-56.4	-59.3	15.7
**DMSO**	-81.9	-21.4	-44.0	11.8
**DIBA**	-99.2	-28.5		
**^{dipp}NHC**	-190.2	-107.4		
**SIMes**	-226.8	-138.5		
**^{iPr}NHC**	-176.8	-102.9		
**$P(nBu)_3$**	-112.6	-32.0		
**$P(Cy)_3$**	-134.8	-58.5		
**DABCO**	-97.4	-28.5		

Interaction Energy (E_{INT}) and Deformation Energy (E_{def}) for Experimentally Realised Complexes of 1

For the interaction energy, the Lewis acid and base fragments were taken from the optimised structures of the adducts and the single point energy was calculated in the gas phase for each fragment.

E_{def} = E(deformed)-E(Opt)

E_{INT} = E(adduct)-E(deformed base)-E(deformed acid) = E(adduct)-E(acid)-E(base)-E_{def}(acid)-E_{def}(base)

Table S3.5. Deformation energy (**E_{def}**) for acid/base fragments and interaction energy (**E_{INT}**) for Lewis adducts **1-X** (DSD-BLYP(D3BJ)/def2-TZVPP).

Adduct	SP energy (adduct) [kJ/mol]	SP energy (acid) [kJ/mol]	SP energy (base) [kJ/mol]	SP energy (deformed acid) [kJ/mol]	SP energy (deformed base) [kJ/mol]	E_{def} (acid) [kJ/mol]	E_{def} (base) [kJ/mol]	E_{INT} [kJ/mol]
**1-F**	-8177100.26	-7914624.21	-261991.10	-7914434.67	-261991.10	189.5	0.0	-674.5
**1-Cl**	-9122708.25	-7914624.21	-1207829.25	-7914439.88	-1207829.25	184.3	0.0	-439.1
**1-Br**	-14671706.02	-7914624.21	-6756878.62	-7914447.75	-6756878.62	176.5	0.0	-379.7
**1-N_3**	-8345790.97	-7914624.21	-430894.33	-7914437.06	-430878.53	187.2	15.8	-475.4
**1-NCS**	-9203523.74	-7914624.21	-1288665.14	-7914445.51	-1288658.44	178.7	6.7	-419.8
**1-CN**	-8158499.97	-7914624.21	-243593.83	-7914446.58	-243591.94	177.6	1.9	-461.5
**1-pyridine**	-8565960.65	-7914624.21	-651261.51	-7914490.80	-651257.62	133.4	3.9	-212.2
**1-DMAP**	-8917409.43	-7914624.21	-1002681.09	-7914482.39	-1002674.58	141.8	6.5	-252.5
**1-$OPEt_3$**	-9631327.09	-7914624.21	-1716591.55	-7914471.60	-1716548.73	152.6	42.8	-306.8
**1-HMPA**	-10067035.08	-7914624.21	-2152283.84	-7914468.70	-2152257.95	155.5	25.9	-308.4
**1-DMSO**	-9366273.53	-7914624.21	-1451569.82	-7914488.50	-1451549.56	135.7	20.3	-235.5
**1-DIBA**	-9585154.37	-7914624.21	-1670405.06	-7914468.07	-1670395.20	156.1	9.9	-291.1
**1-^{dipp}NHC**	-11163857.69	-7914624.21	-3249014.51	-7914455.15	-3248973.22	169.1	41.3	-429.3
**1-SIMes**	-10342140.89	-7914624.21	-2427239.34	-7914473.45	-2427228.31	150.8	11.0	-439.1
**1-^{iPr}NHC**	-9333044.13	-7914624.21	-1418210.88	-7914468.39	-1418200.61	155.8	10.3	-375.1
**1-$P(nBu)_3$**	-10052452.89	-7914624.21	-2137702.66	-7914492.78	-2137686.87	131.4	15.8	-273.2
**1-PCy_3**	-10661711.95	-7914624.21	-2746943.38	-7914483.51	-2746927.28	140.7	16.1	-301.2
**1-DABCO**	-8820534.58	-7914624.21	-905811.95	-7914482.79	-905802.71	141.4	9.2	-249.1

Vacuum Affinities of 1

Table S3.6. Computed energies for the calculation of thermodynamics of mono-adduct formation in vacuum (DSD-BLYP(D3BJ)/def2-TZVPP//r^{2}scan-3c.

	**Charge/ multiplicity**	**Total Correction [kJ/mol]** r^{2}scan-3c	**Entropy Term (T*S) [kJ/mol]** r^{2}scan-3c	**Final Single Point Energy [kJ/mol]** DSD-BLYP/def2-TZVPP	**Final Enthalpy [kJ/mol]** DSD-BLYP/def2-TZVPP//r^{2}scan-3c	**Final Gibbs Free Energy [kJ/mol]** DSD-BLYP/def2-TZVPP//r^{2}scan-3c
**1**	0/1	2691.93	376.26	-7914623.05	-7911928.64	-7912304.90
**1-F**	-1/1	2699.03	379.85	-8177099.06	-8174397.55	-8174777.40
**1-Cl**	-1/1	2696.91	383.29	-9122706.91	-9120007.52	-9120390.81
**1-N$_3$**	-1/1	2733.09	390.43	-8345789.74	-8343054.17	-8343444.60
**1-NCS**	-1/1	2728.38	393.46	-9203522.38	-9200791.52	-9201184.98
**1-CN**	-1/1	2717.19	386.42	-8158498.77	-8155779.10	-8156165.52
**1-Br**	-1/1	2696.55	385.63	-14671706.02	-14669007.08	-14669391.29
**1-pyridine**	0/1	2945.12	403.87	-8565959.39	-8563011.80	-8563415.67
**1-DMAP**	0/1	3147.53	427.36	-8917408.12	-8914258.11	-8914685.47
**1-OPEt$_3$**	0/1	3263.32	436.42	-9631325.68	-9628059.88	-9628496.31
**1-HMPA**	0/1	3406.33	459.28	-10067033.60	-10063624.79	-10064084.07
**1-DMSO**	0/1	2921.68	407.80	-9366272.16	-9363348.00	-9363755.80
**1-DIBA**	0/1	3517.51	454.65	-9585152.96	-9581632.97	-9582087.62
**1-^{dipp}NHC**	0/1	4422.81	535.51	-11163872.38	-11159447.09	-11159982.61
**1-SIMes**	0/1	3862.91	484.08	-10342140.89	-10338275.5	-10338759.58
**1-^{iPr}NHC**	0/1	3514.62	448.98	-9333044.13	-9329527.04	-9329976.02
**1-P(nBu)$_3$**	0/1	3715.98	463.75	-10052452.89	-10048734.44	-10049198.19
**1-PCy$_3$**	0/1	4013.84	470.01	-10661711.95	-10657695.63	-10658165.64
**1-DABCO**	0/1	3198.77	405.74	-8820534.58	-8817333.33	-8817739.07
**F**	-1/1	3.72	43.37	-261991.06	-261984.86	-262028.23
**Cl**	-1/1	3.72	45.69	-1207829.07	-1207822.88	-1207868.57
**N$_3$**	-1/1	35.55	63.30	-430894.27	-430856.24	-430919.54
**NCS**	-1/1	28.34	51.40	-1288664.95	-1288634.14	-1288685.53
**CN**	-1/1	18.92	58.66	-243593.80	-243572.40	-243631.06
**Br**	-1/1	3.72	48.71	-6756878.62	-6756872.42	-6756921.13
**Pyridine**	0/1	243.07	84.03	-651261.41	-651015.87	-651099.89
**DMAP**	0/1	444.67	111.79	-1002680.94	-1002233.79	-1002345.58
**OPEt$_3$**	0/1	561.89	127.63	-1716591.30	-1716026.93	-1716154.56
**HMPA**	0/1	703.96	148.44	-2152283.52	-2151577.08	-2151725.52
**DMSO**	0/1	221.42	92.02	-1451569.61	-1451345.71	-1451437.73
**DIBA**	0/1	816.62	149.08	-1670420.22	-1669601.12	-1669750.19
**^{dipp}NHC**	0/1	1720.25	242.01	-3249014.03	-3247291.31	-3247533.32
**SIMes**	0/1	656.73	131.33	-2427239.34	-2426078.67	-2426274.77
**^{iPr}NHC**	0/1	811.34	146.62	-1418210.88	-1417397.07	-1417543.69
**P(nBu)$_3$**	0/1	1014.69	168.04	-2137702.66	-2136685.49	-2136853.53
**PCy$_3$**	0/1	1312.68	170.04	-2746943.38	-2745628.22	-2745798.26
**DABCO**	0/1	494.80	98.40	-905811.95	-905314.67	-905413.07

Table S3.7. Computed thermodynamics of mono-adduct (**1-X**) formation in vacuum (DSD-BLYP(D3BJ)/def2-TZVPP//r2scan-3c).

**X**	**ΔH [kJ/mol] 1 + X → 1-X**	**ΔG [kJ/mol] 1 + X → 1-X**
**F-**	-484.0	-444.3
**Cl-**	-256.0	-217.3
**Br-**	-206.0	-165.3
**N$_3$-**	-269.3	-220.2
**NCS-**	-228.7	-194.6
**CN-**	-278.1	-229.6
**Pyridine**	-67.3	-10.9
**DMAP**	-95.7	-35.0
**OPEt$_3$**	-104.3	-36.8
**HMPA**	-119.1	-53.7
**DMSO**	-73.7	-13.2
**DIBA**	-103.2	-32.5
**^{dipp}NHC**	-227.1	-144.4
**SIMes**	-268.2	-179.9
**^{iPr}NHC**	-201.3	-127.4
**P(nBu)$_3$**	-120.3	-39.8
**PCy$_3$**	-138.8	-62.5
**DABCO**	-90.0	-21.1

Thermodynamics of Adduct Formation: COSMO-RS Solvation

Table S3.8. Computed energies for the calculation of thermodynamics of mono-adduct formation in vacuum (DSD-BLYP(D3BJ)/def2-TZVPP//r^{2}scan-3c.

	**Charge/ multiplicity**	**Final Enthalpy (Vacuum) [kJ/mol]** DSD-BLYP/def2-TZVPP//r^{2}scan-3c	**Final Gibbs Free Energy (Vacuum) [kJ/mol]** DSD-BLYP/def2-TZVPP//r^{2}scan-3c	**Enthalpy Correction [kJ/mol]** COSMO-RS (DCM)	**Gibbs Free Energy Correction [kJ/mol]** COSMO-RS (DCM)	**Final Enthalpy (DCM) [kJ/mol]** DSD-BLYP/def2-TZVPP/ COSMO-RS(DCM)//r^{2}scan-3c	**Final Gibbs Free Energy (Vacuum) [kJ/mol]** DSD-BLYP/def2-TZVPP/ COSMO-RS(DCM)//r^{2}scan-3c
**1**	0/1	-7911928.64	-7912304.90	-154.19	-141.28	-7912082.83	-7912446.18
**1-F**	-1/1	-8174397.55	-8174777.40	-277.70	-259.84	-8174675.25	-8175037.24
**1-Cl**	-1/1	-9120007.52	-9120390.81	-280.18	-262.94	-9120287.70	-9120653.75
**1-N_3**	-1/1	-8343054.17	-8343444.60	-274.76	-257.58	-8343328.93	-8343702.18
**1-NCS**	-1/1	-9200791.52	-9201184.98	-272.93	-258.29	-9201064.45	-9201443.27
**1-CN**	-1/1	-8155779.10	-8156165.52	-283.53	-264.83	-8156062.63	-8156430.35
**1-pyridine**	0/1	-8563011.80	-8563415.67	-198.26	-181.04	-8563210.06	-8563596.71
**1-DMAP**	0/1	-8914258.11	-8914685.47	-216.39	-198.65	-8914474.50	-8914884.12
**1-$OPEt_3$**	0/1	-9628059.88	-9628496.31	-215.10	-195.95	-9628274.98	-9628692.26
**1-HMPA**	0/1	-10063624.79	-10064084.07	-208.90	-191.40	-10063833.69	-10064275.47
**1-DMSO**	0/1	-9363348.00	-9363755.80	-205.10	-185.75	-9363553.10	-9363941.55
**1-DIBA**	0/1	-9581632.97	-9582087.62	-203.84	-186.76	-9581836.81	-9582274.38
**1-^{dipp}NHC**	0/1	-11159447.09	-11159982.61	-196.86	-180.33	-11159643.95	-11160162.94
**F**	-1/1	-261984.86	-262028.23	-343.05	-317.14	-262327.91	-262345.37
**Cl**	-1/1	-1207822.88	-1207868.57	-304.70	-277.22	-1208127.58	-1208145.79
**N_3**	-1/1	-430856.24	-430919.54	-269.02	-243.41	-431125.26	-431162.95
**NCS**	-1/1	-1288634.14	-1288685.53	-249.61	-226.64	-1288883.75	-1288912.17
**CN**	-1/1	-243572.40	-243631.06	-288.02	-261.14	-243860.42	-243892.20
**Pyridine**	0/1	-651015.87	-651099.89	-27.98	-18.91	-651043.85	-651118.80
**DMAP**	0/1	-1002233.79	-1002345.58	-45.57	-34.62	-1002279.36	-1002380.20
**$OPEt_3$**	0/1	-1716026.93	-1716154.56	-55.24	-42.51	-1716082.17	-1716197.07
**HMPA**	0/1	-2151577.08	-2151725.52	-54.20	-42.96	-2151631.28	-2151768.48
**DMSO**	0/1	-1451345.71	-1451437.73	-46.90	-33.25	-1451392.61	-1451470.98
**DIBA**	0/1	-1669601.12	-1669750.19	-58.09	-47.39	-1669659.21	-1669797.58
**NHC**	0/1	-3247291.31	-3247533.32	-76.31	-34.62	-3247367.62	-3247567.94

Table S3.9. Computed thermodynamics of mono-adduct (**1-X**) formation in DCM (DSD-BLYP(D3BJ)/def2-TZVPP/COSMO-RS(DCM)//r2scan-3c).

**X**	**ΔH [kJ/mol] 1 + X → 1-X**	**ΔG [kJ/mol] 1 + X → 1-X**
**F-**	-264.5	-245.7
**Cl-**	-77.3	-61.8
**N_3-**	-120.8	-93.0
**NCS-**	-97.9	-84.9
**CN-**	-119.4	-92.0
**Pyridine**	-83.4	-31.7
**DMAP**	-112.3	-57.7
**$OPEt_3$**	-110.0	-49.0
**HMPA**	-119.6	-60.8
**DMSO**	-77.7	-24.4
**DIBA**	-94.8	-30.6
**NHC**	-193.5	-148.8

Thermodynamics of Adduct Formation: Explicit Cations

For geometry optimisations of ion pairs, SMD solvation modelling with DCM as solvent was used and numerical frequency analysis was performed. Not all imaginary frequencies could be removed, giving unreliable values for entropy and Gibbs free energy. However, imaginary frequencies were found sufficiently small to give estimates of reaction enthalpies.

Table S3.10. Computed energies for the calculation of thermodynamics of mono-adduct formation with explicit cations in DCM (DSD-BLYP(D3BJ)/def2-TZVPP/SMD(DCM)//r^{2}scan-3c/SMD(DCM).

	**Charge/ multiplicity**	**Total Correction [kJ/mol]** r^{2}scan-3c/SMD(DCM)	**Entropy Term (T*S) [kJ/mol]** r^{2}scan-3c/ SMD(DCM)	**Final Single Point Energy [kJ/mol]** DSD-BLYP/def2-TZVPP/ SMD(DCM)	**Final Enthalpy [kJ/mol]** DSD-BLYP/def2-TZVPP//r^{2}scan-3c/ SMD(DCM)	**Final Gibbs Free Energy [kJ/mol]** DSD-BLYP/def2-TZVPP//r^{2}scan-3c/ SMD(DCM)
**1**	0/1	2679.3489	367.3269	-7914781.3586	-7912099.5308	-7912466.858
**[1-F][NBu$_4$]**	0/1	4074.7763	481.4077	-9976711.1285	-9972633.8732	-9973115.281
**[1-Cl][PPh$_4$]**	0/1	3711.7016	492.5844	-12448919.6054	-12445205.4249	-12445698.01
**[1-N3][NBu$_4$]**	0/1	4111.3101	501.1021	-10145390.9150	-10141277.1259	-10141778.23
**[1-NCS][NBu$_4$]**	0/1	4103.3370	513.6363	-11003129.3359	-10999023.5200	-10999537.16
**[1-CN][NBu$_4$]**	0/1	4092.7311	501.5246	-9958118.5382	-9954023.3282	-9954524.853
**NBu$_4$F**	0/1	1385.4121	200.4735	-2061692.1510	-2060304.2599	-2060504.733
**PPh$_4$Cl**	0/1	1021.1276	194.8645	-4534010.2040	-4532986.5975	-4533181.462
**NBu$_4$N$_3$**	0/1	1420.2356	213.4844	-2230468.8926	-2229046.1781	-2229259.663
**NBu$_4$NCS**	0/1	1414.5993	221.3629	-3088227.7025	-3086811.4526	-3087025.166
**NBu$_4$CN**	0/1	1402.8911	212.7395	-2043184.2791	-2041778.9090	-2041991.649

Table S3.11. Computed Enthalpy of mono-adduct formation with explicit cations (**[1-X][Cation]**) in DCM (DSD-BLYP(D3BJ)/def2-TZVPP/SMD(DCM)//r2scan-3c/SMD(DCM)).

**X**	**ΔH [kJ/mol] 1 + X → 1-X**
NBu$_4$F	-230.1
PPh$_4$Cl	-119.3
NBu$_4$N$_3$	-131.4
NBu$_4$NCS	-112.5
NBu$_4$CN	-144.9

Computed Structural Parameters

Table S3.12. Computed structural parameters of **1** and mono-adducts **1-X**, optimised on the r2scan-3c level of theory.

1-X	Average Si-N bond length [Å]	Average Si-O bond length [Å]	Si-X bond length [Å]	N-Si-N bond angle [°]
**1**	1.65974	1.69568		121.012
**1-F**	1.78286	1.78729	1.62247	118.157
**1-Cl**	1.77668	1.78368	2.13704	118.345
**1-Br**	1.79132	1.77168	2.32056	117.898
**1-N_3**	1.79306	1.78348	1.78935	117.107
**1-NCS**	1.77500	1.78285	1.76389	118.813
**1-CN**	1.77798	1.78236	1.88619	118.186
**1-pyridine**	1.75017	1.75758	1.93397	132.547
**1-DMAP**	1.75671	1.76237	1.91015	127.657
**1-$OPEt_3$**	1.76603	1.76779	1.76480	123.175
**1-HMPA**	1.76109	1.76954	1.76459	125.825
**1-DMSO**	1.75018	1.76060	1.82686	124.358
**1-DIBA**	1.75781	1.76870	1.78578	128.580
**1-^{dipp}NHC**	1.74881	1.77334	1.99259	120.275
**1-SIMes**	1.78141	1.76753	1.98452	119.357
**1-^{iPr}NHC**	1.77903	1.77559	1.93972	120.954
**1-$P(nBu)_3$**	1.77569	1.75407	2.41544	119.582
**1-PCy_3**	1.77766	1.76434	2.37355	120.330
**1-DABCO**	1.77022	1.74949	2.00216	126.368

Para-Fluorophenol Hydrogen Bonding Thermodynamics

Table S3.13. Computed energies for the calculation of thermodynamics of hydrogen bond complex formation between para-fluorophenol and Lewis bases in vacuum, DCM and CCl_4 (DSD-BLYP(D3BJ)/def2-TZVPP//r^{2}scan-3c with corresponding SMD solvation correction).

	**Charge/ multiplicity**	**Total Correction [kJ/mol]** r^{2}scan-3c	**Entropy Term (T*S) [kJ/mol]** r^{2}scan-3c	**Final Single Point Energy Vacuum [kJ/mol]** DSD-BLYP/def2-TZVPP	**Final Single Point Energy DCM [kJ/mol]** DSD-BLYP/def2-TZVPP/SMD(DCM)	**Final Single Point Energy CCl_4 [kJ/mol]** DSD-BLYP/def2-TZVPP/SMD(CCl_4)
p-F-PhOH	0/1	268.89	98.85	-1067013.1	-1067048.52	-1067040.21
p-F-PhOH-Br	-1/1	273.69	115.74	-7824001.21	-7824189.66	-7824127.68
p-F-PhOH-Cl	-1/1	273.17	112.73	-2274974.83	-2275185.37	-2275112.84
p-F-PhOH-CN	-1/1	288.02	118.26	-1310730.67	-1310928.07	-1310856.26
p-F-PhOH-F	-1/1	268.84	109.86	-1329264.37	-1329472.22	-1329397.54
p-F-PhOH-N_3	-1/1	306.45	122.96	-1498024.52	-1498207.8	-1498138.01
p-F-PhOH-NCS	-1/1	303.76	127.44	-2355780.14	-2355963.98	-2355901.71
p-F-PhOH-DABCO	0/1	772.09	147.71	-1972877.95	-1972942.76	-1972930.16
p-F-PhOH-DIBA	0/1	1094.08	193.9	-2737495.33	-2737567.87	-2737556.89
p-F-PhOH-DMAP	0/1	721.93	163.72	-2069747.91	-2069815.56	-2069804.25
p-F-PhOH-DMSO	0/1	498.8	141.34	-2518641.35	-2518690.89	-2518679.69
p-F-PhOH-HMPA	0/1	981.6	196.75	-3219364.38	-3219420.24	-3219411.49
p-F-PhOH-^{iPr}NHC	0/1	1086.2	192.66	-2485296.56	-2485371.7	-2485360.61
p-F-PhOH-^{dipp}NHC	0/1	1995.42	285.06	-4316114.76	-4316214.98	-4316200.83
p-F-PhOH-$OPEt_3$	0/1	839.66	172.7	-2783652.78	-2783716.49	-2783703.28
p-F-PhOH-PBu_3	0/1	1290.85	213.01	-3204764.35	-3204825.51	-3204820.38
p-F-PhOH- PCy_3	0/1	1589.22	214.85	-3814000.29	-3814074.18	-3814069.53
p-F-PhOH-Pyridine	0/1	520.46	138.51	-1718320.82	-1718376.75	-1718368.91
p-F-PhOH-SIMes	0/1	1433.63	235.08	-3494337.14	-3494434.58	-3494420.56
Br	-1/1	3.72	48.71	-6756878.62	-6757090.16	-6757014.72
Cl	-1/1	3.72	45.69	-1207829.25	-1208090.53	-1207994.25
CN	-1/1	18.92	58.66	-243593.83	-243851.29	-243751.68
F	-1/1	3.72	43.37	-261991.08	-262338.94	-262206.55
N3	-1/1	35.55	63.3	-430894.34	-431123.63	-431029.83
NCS	-1/1	28.34	51.4	-1288665.13	-1288879.67	-1288798.77
DABCO	0/1	494.8	98.4	-905811.95	-905849.03	-905839.78
DIBA	0/1	808.72	142.96	-1670405.03	-1670458.69	-1670450.46
DMAP	0/1	444.67	111.79	-1002681.15	-1002724.75	-1002714.11
DMSO	0/1	221.42	92.02	-1451569.8	-1451602.54	-1451591.47
HMPA	0/1	703.96	148.44	-2152283.78	-2152323.36	-2152314.8
^{iPr}NHC	0/1	811.34	146.62	-1418210.88	-1418280.44	-1418269.31
^{dipp}NHC	0/1	1720.25	242.01	-3249014.48	-3249104.77	-3249093.03
$OPEt_3$	0/1	561.89	127.63	-1716591.55	-1716635.19	-1716623.33
PBu_3	0/1	1014.69	168.04	-2137702.66	-2137741.57	-2137740.14
PCy_3	0/1	1312.68	170.04	-2746943.38	-2746996.02	-2746993.9
Pyridine	0/1	243.07	84.03	-651261.52	-651291.1	-651285.03
SIMes	0/1	1158.2	196.1	-2427239.34	-2427331.43	-2427319.52

	**Final Enthalpy (vacuum)** DSD-BLYP/def2-TZVPP//r^{2}scan-3c	**Final Gibbs Free Energy (vacuum)** DSD-BLYP/def2-TZVPP//r^{2}scan-3c	**Final Enthalpy (DCM)** DSD-BLYP/def2-TZVPP/ SMD(DCM)//r^2 scan-3c	**Final Gibbs Free Energy (DCM)** DSD-BLYP/def2-TZVPP/ SMD(DCM)//r^{2}scan -3c	**Final Enthalpy (CCl_4)** DSD-BLYP/def2-TZVPP/SMD(CCl_4)//r^{2}scan-3c	**Final Gibbs Free Energy (CCl_4)** DSD-BLYP/def2-TZVPP/SMD(CCl_4)//r^{2}scan-3c
p-F-PhOH	-1066741.73	-1066840.59	-1066777.15	-1066876.01	-1066768.84	-1066867.7
p-F-PhOH-Br	-7823725.04	-7823840.78	-7823913.49	-7824029.23	-7823851.51	-7823967.25
p-F-PhOH-Cl	-2274699.18	-2274811.91	-2274909.72	-2275022.45	-2274837.19	-2274949.92
p-F-PhOH-CN	-1310440.18	-1310558.44	-1310637.58	-1310755.84	-1310565.77	-1310684.03
p-F-PhOH-F	-1328993.05	-1329102.91	-1329200.9	-1329310.76	-1329126.22	-1329236.08
p-F-PhOH-N_3	-1497715.59	-1497838.54	-1497898.87	-1498021.82	-1497829.08	-1497952.03
p-F-PhOH-NCS	-2355473.9	-2355601.34	-2355657.74	-2355785.18	-2355595.47	-2355722.91
p-F-PhOH-DABCO	-1972103.38	-1972251.09	-1972168.19	-1972315.9	-1972155.59	-1972303.3
p-F-PhOH-DIBA	-2736398.77	-2736592.67	-2736471.31	-2736665.21	-2736460.33	-2736654.23
p-F-PhOH-DMAP	-2069023.5	-2069187.22	-2069091.15	-2069254.87	-2069079.84	-2069243.56
p-F-PhOH-DMSO	-2518140.08	-2518281.42	-2518189.62	-2518330.96	-2518178.42	-2518319.76
p-F-PhOH-HMPA	-3218380.3	-3218577.05	-3218436.16	-3218632.91	-3218427.41	-3218624.16
p-F-PhOH-^{iPr}NHC	-2484207.88	-2484400.53	-2484283.02	-2484475.67	-2484271.93	-2484464.58
p-F-PhOH-^{dipp}NHC	-4314116.86	-4314401.92	-4314217.08	-4314502.14	-4314202.93	-4314487.99
p-F-PhOH-$OPEt_3$	-2782810.64	-2782983.34	-2782874.35	-2783047.05	-2782861.14	-2783033.84

p-F-PhOH-PBu$_3$	-3203471.03	-3203684.03	-3203532.19	-3203745.19	-3203527.06	-3203740.06
p-F-PhOH- PCy$_3$	-3812408.59	-3812623.44	-3812482.48	-3812697.33	-3812477.83	-3812692.68
p-F-PhOH-Pyridine	-1717797.89	-1717936.39	-1717853.82	-1717992.32	-1717845.98	-1717984.48
p-F-PhOH-SIMes	-3492901.02	-3493136.11	-3492998.46	-3493233.55	-3492984.44	-3493219.53
Br	-6756872.42	-6756921.13	-6757083.96	-6757132.67	-6757008.52	-6757057.23
Cl	-1207823.05	-1207868.74	-1208084.33	-1208130.02	-1207988.05	-1208033.74
CN	-243572.43	-243631.1	-243829.89	-243888.56	-243730.28	-243788.95
F	-261984.89	-262028.26	-262332.75	-262376.12	-262200.36	-262243.73
N3	-430856.31	-430919.61	-431085.6	-431148.9	-430991.8	-431055.1
NCS	-1288634.31	-1288685.71	-1288848.85	-1288900.25	-1288767.95	-1288819.35
DABCO	-905314.67	-905413.07	-905351.75	-905450.15	-905342.5	-905440.9
DIBA	-1669593.84	-1669736.8	-1669647.5	-1669790.46	-1669639.27	-1669782.23
DMAP	-1002234	-1002345.78	-1002277.6	-1002389.38	-1002266.96	-1002378.74
DMSO	-1451345.9	-1451437.92	-1451378.64	-1451470.66	-1451367.57	-1451459.59
HMPA	-2151577.34	-2151725.78	-2151616.92	-2151765.36	-2151608.36	-2151756.8
^{iPr}NHC	-1417397.07	-1417543.69	-1417466.63	-1417613.25	-1417455.5	-1417602.12
^{dipp}NHC	-3247291.75	-3247533.76	-3247382.04	-3247624.05	-3247370.3	-3247612.31
OPEt$_3$	-1716027.19	-1716154.82	-1716070.83	-1716198.46	-1716058.97	-1716186.6
PBu$_3$	-2136685.49	-2136853.53	-2136724.4	-2136892.44	-2136722.97	-2136891.01
PCy$_3$	-2745628.22	-2745798.26	-2745680.86	-2745850.9	-2745678.74	-2745848.78
Pyridine	-651015.98	-651100.01	-651045.56	-651129.59	-651039.49	-651123.52
SIMes	-2426078.67	-2426274.77	-2426170.76	-2426366.86	-2426158.85	-2426354.95

Table S3.14. Computed thermodynamics of hydrogen bond formation between Lewis bases and para-fluorophenol (**p-F-PhOH-X**) in vacuum, DCM, and CCl$_4$ (DSD-BLYP(D3BJ)/def2-TZVPP//r^{2}scan-3c with corresponding SMD solvation correction).

**X**	**ΔH (vacuum) [kJ/mol] p-F-PhOH + X → p-F-PhOH-X**	**ΔG (vacuum) [kJ/mol] p-F-PhOH + X → p-F-PhOH-X**	**ΔH (DCM) [kJ/mol] p-F-PhOH + X → p-F-PhOH-X**	**ΔG (DCM) [kJ/mol] p-F-PhOH + X → p-F-PhOH-X**	**ΔH (CCl$_4$) [kJ/mol] p-F-PhOH + X → p-F-PhOH-X**	**ΔG (CCl$_4$) [kJ/mol] p-F-PhOH + X → p-F-PhOH-X**
Br	-110.9	-79.1	-52.4	-20.6	-74.2	-42.3
Cl	-134.4	-102.6	-48.2	-16.4	-80.3	-48.5
CN	-126.0	-86.7	-30.5	8.7	-66.6	-27.4
F	-266.4	-234.1	-91.0	-58.6	-157.0	-124.7
N3	-117.5	-78.3	-36.1	3.1	-68.4	-29.2
NCS	-97.9	-75.0	-31.7	-8.9	-58.7	-35.9
DABCO	-47.0	2.6	-39.3	10.3	-44.2	5.3
DIBA	-63.2	-15.3	-46.7	1.3	-52.2	-4.3
DMAP	-47.8	-0.8	-36.4	10.5	-44.0	2.9
DMSO	-52.4	-2.9	-33.8	15.7	-42.0	7.5
HMPA	-61.2	-10.7	-42.1	8.5	-50.2	0.3
^{iPr}NHC	-69.1	-16.3	-39.2	13.6	-47.6	5.2
^{dipp}NHC	-83.4	-27.6	-57.9	-2.1	-63.8	-8.0
OPEt$_3$	-41.7	12.1	-26.4	27.4	-33.3	20.5
PBu$_3$	-43.8	10.1	-30.6	23.3	-35.2	18.6
PCy$_3$	-38.6	15.4	-24.5	29.6	-30.2	23.8
Pyridine	-40.2	4.2	-31.1	13.3	-37.6	6.7
SIMes	-80.6	-20.8	-50.5	9.3	-56.7	3.1

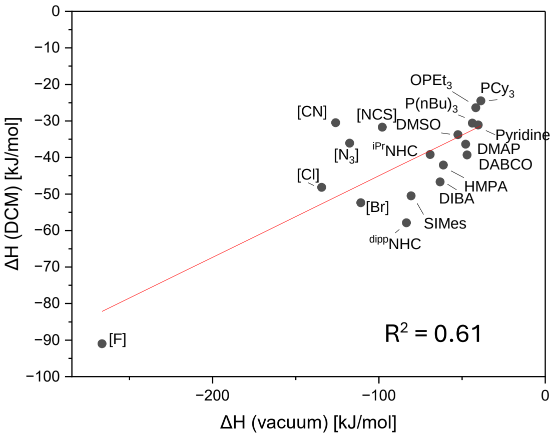

Figure S3.1. Correlation between vacuum and solution (DCM) enthalpies for hydrogen bond formation between Lewis bases and para-fluorophenol.

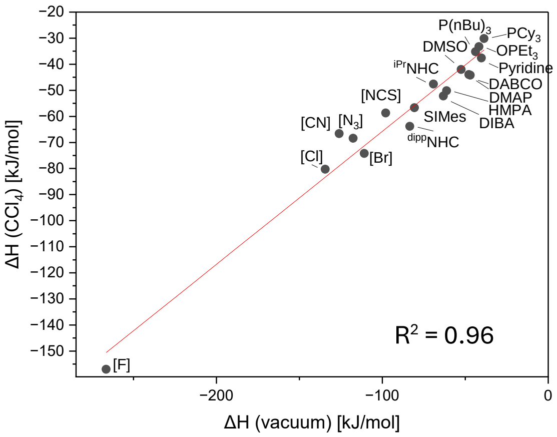

Figure S3.2. Correlation between vacuum and solution (CCl$_4$) enthalpies for hydrogen bond formation between Lewis bases and para-fluorophenol.

Relative Stability of Pyridine Bis-Adduct Conformations

For the conformer search, the relative Gibbs free energies of three conformations of the pyridine bis-adduct were calculated on the r^{2}scan-3c level of theory.

Table S3.15. Conformer search of pyridine bis-adducts of **1** on the r2scan-3c level of theory.

	**Total Correction [kJ/mol]** r^{2}scan-3c	**Entropy Term (T*S) [kJ/mol]** r^{2}scan-3c	**Single Point Energy [kJ/mol]** r^{2}scan-3c	**Enthalpy [kJ/mol]** r^{2}scan-3c	**Gibbs Free Energy [kJ/mol]** r^{2}scan-3c
**1-(pyridine)$_2$ trans**	3196.91	429.12	-9223407.97	-9220208.58	-9220637.70
**1-(pyridine)$_2$ cis**	3196.05	422.58	-9223409.05	-9220210.53	-9220633.11
**1-(pyridine)$_2$ cis 2**	3196.79	426.25	-9223348.73	-9220149.45	-9220575.70

Relative energy

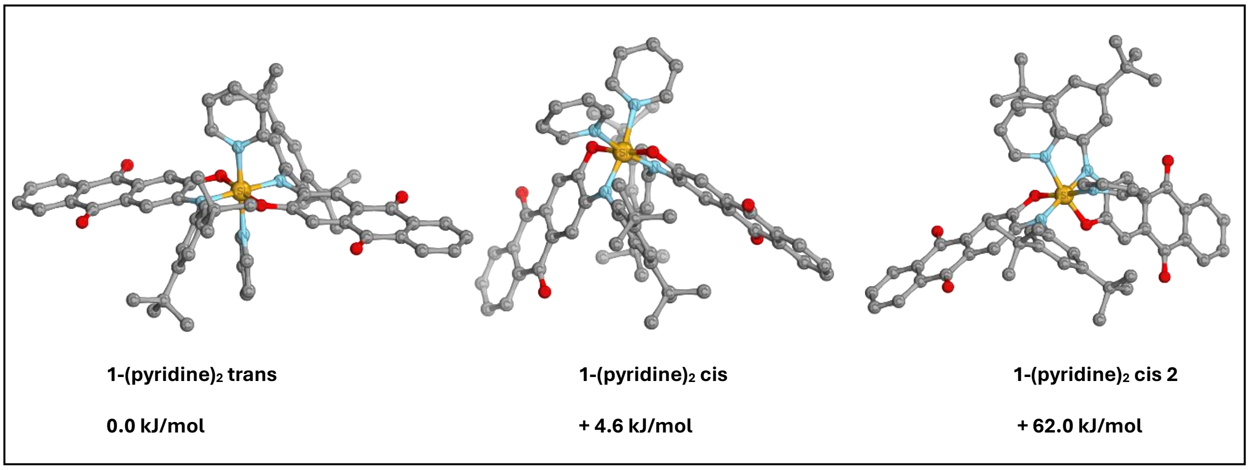

Figure S3.3. Optimised structures and relative Gibbs free energies of pyridine bis-adducts of **1**.

3.4 NBO Analysis of Negative Hyperconjugation in 1 and [1-Cl]

NBO analysis was done for **1** and the chloride adduct **[1-Cl]**. Only one set of orbital interactions of the symmetric complex is shown. Second order perturbation theory stabilisation energies of the LP(N/O) → LV(Si) negative hyperconjugation are given. Below, the visualisation of participating NBOs (Chemcraft) and occupancies (Occ.) are given.

1

**Orbital Interaction**			**Stabilisation Energy [kcal/mol]**
75. LP (2) O 23	→	234. LV (1)Si119	0.31
75. LP (2) O 23	→	235. LV (2)Si119	8.43
75. LP (2) O 23	→	236. LV (3)Si119	14.81
75. LP (2) O 23	→	237. LV (4)Si119	2.18
77. LP (1) N 24	→	234. LV (1)Si119	0.76
77. LP (1) N 24	→	235. LV (2)Si119	8.69
77. LP (1) N 24	→	236. LV (3)Si119	16.73
77. LP (1) N 24	→	237. LV (4)Si119	2.38
			**Total Stabilisation Energy [kcal/mol]**
2x LP(O/N) → LV (Si) negative hyperconjugation			**108.58**

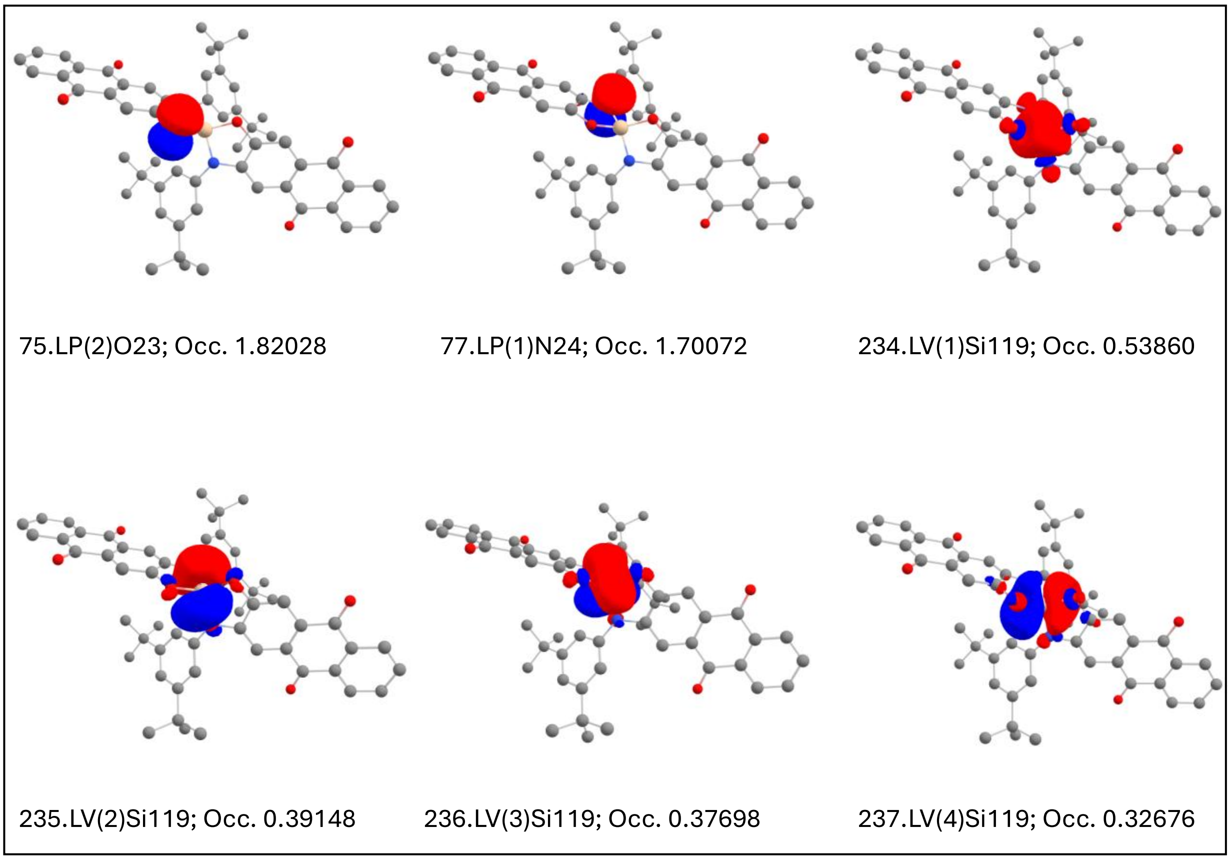

Figure S3.4. Natural bond orbitals involved in the LP(O/N) → LV(Si) negative hyperconjugation in **1**.

[1-Cl]

**Orbital Interaction**			**Stabilisation Energy [kcal/mol]**
80. LP (2) O 23	→	243. LV (1)Si119	5.91
80. LP (2) O 23	→	244. LV (2)Si119	3.05
80. LP (2) O 23	→	245. LV (3)Si119	0.61
80. LP (2) O 23	→	392. BD*(1)Si119-Cl120	3.21
82. LP (1) N 24	→	243. LV (1)Si119	21.26
82. LP (1) N 24	→	244. LV (2)Si119	1.57
82. LP (1) N 24	→	245. LV (3)Si119	0.37
82. LP (1) N 24	→	392. BD*(1)Si119-Cl120	6.71
			**Total Stabilisation Energy [kcal/mol]**
2x LP(O/N) → LV/BD*(Si-Cl) negative hyperconjugation			**85.38**

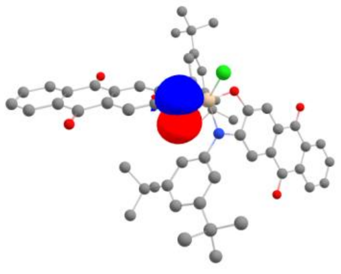

80.LP(2)O23; Occ. 1.78051

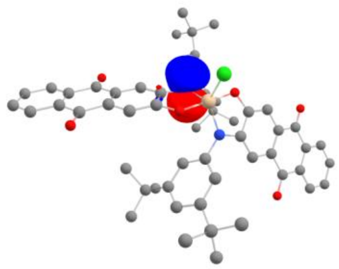

82.LP(1)N24; Occ. 1.70119

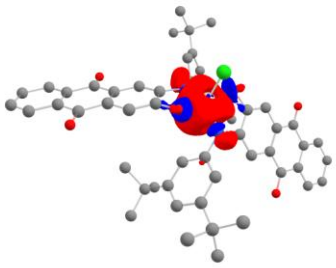

243. LV(1)Si119; Occ. 0.49498

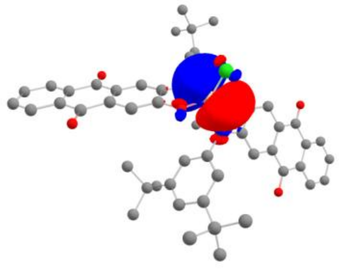

244.LV(2)Si119; Occ. 0.41919

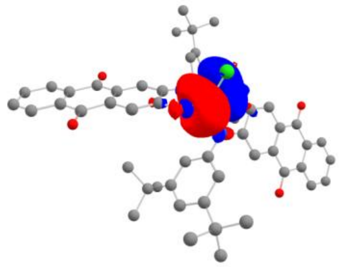

245.LV(3)Si119; Occ. 0.37993

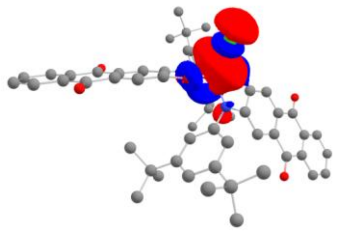

392.BD*(1)Si119-Cl120; Occ. 0.15007

Figure S3.5. Natural bond orbitals involved in the LP(O/N) → LV(Si) negative hyperconjugation in **[1-Cl]**.

3.5 TD-DFT/TDA Calculated Excitations

Time-dependant calculations were carried out with structures optimised with the r2scan-3c composite method. The first 50 electronic transitions were calculated. For comparisons with experimental values, a systematic shift of -0.56 eV was applied to all energies given in the following section. The largest molecular orbital contributions for the low-energy excitations are visualised. For the simulated spectrum, a gaussian line broadening was applied as implemented in the Chemcraft software.

1

Table S3.16. Calculated TD-DFT/TDA (*ωB97X-D3/def2-TZVPP/SMD(DCM))* electronic transitions for compound **1**.

Wavelength [nm]	Oscillator strength (f)
441.5	0
441.1	0
404.3	0
403.8	0
374.2	0
373.8	0
367.6	0
367.3	0
354.4	4.8396E-5
354.1	7.4926E-5
347.9	0
347.7	0
344.2	0.295656916
343.2	0.087280005
330.3	0.001334044
330.1	0.001123268
327.5	0
327.3	0
321.6	0
321.4	0
303.1	0
303	0
290.6	0.139986246
290.3	1.6962E-5
282.9	0
282.7	0
280.4	0
280.3	0
276.7	0.618652193
276.5	0.021707714
268.2	0
267.9	0
265.9	2.253580904
264.8	0
264.7	0
262.8	0.240132824
252.7	0
252.5	0
252	0.897900902
251.2	0.298566906
248.7	0
248	0
247.5	0
247.4	0
241.5	0
241.3	0.007951568
240.8	0.043093286
240.7	0
238.3	0.006908931
238.2	0.005599111

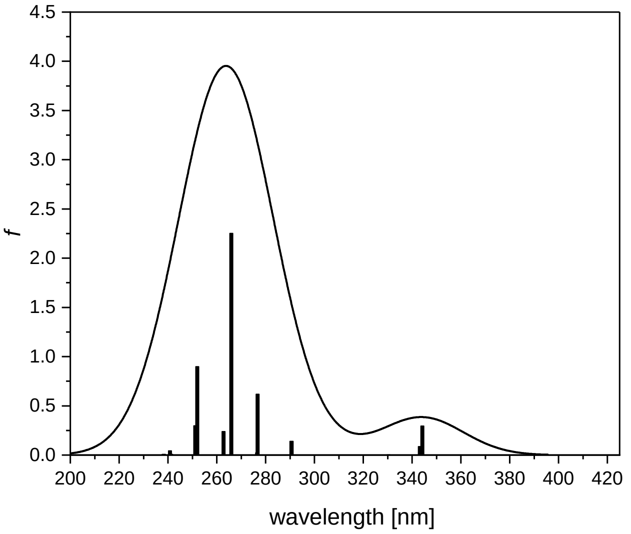

Figure S3.6. Simulated UV-vis spectrum of **1** in DCM (ωB97X-D3/def2-TZVPP/SMD(DCM)). Gaussian line broadening with 40 nm FWHM.

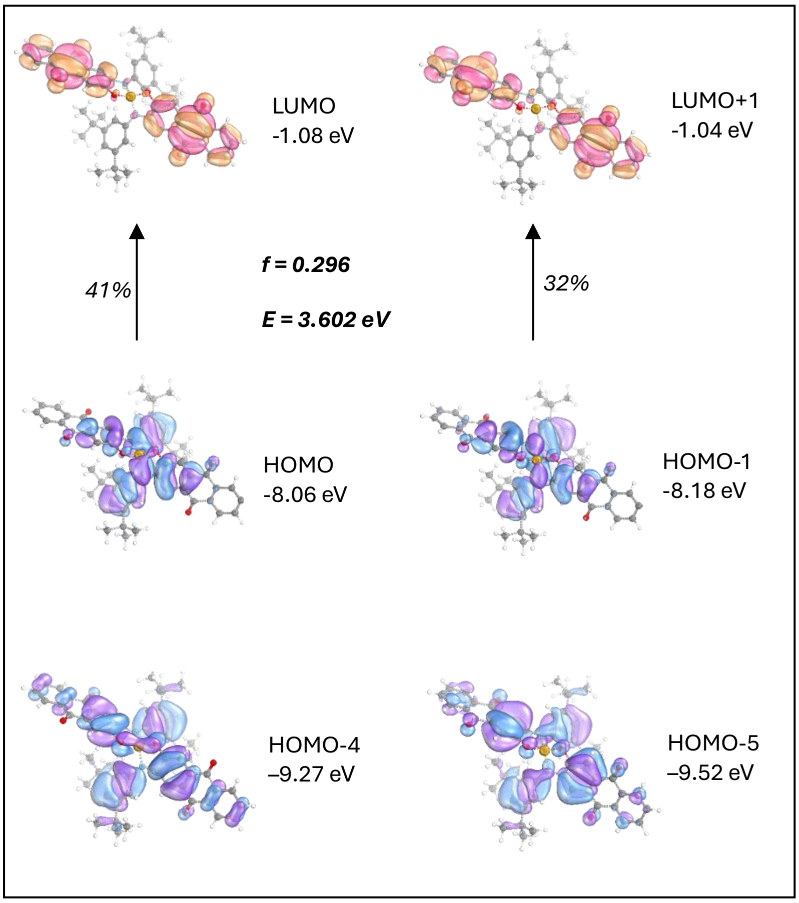

Figure S3.7. Molecular orbitals involved in the low energy CT transition of **1**. HOMO -> LUMO 41%, HOMO-1->LUMO+1 32%, HOMO-4->LUMO 6%, HOMO-5->LUMO+1 6%.

[1-F]

Table S3.17. Calculated TD-DFT/TDA (*ωB97X-D3/def2-TZVPP/SMD(DCM))* electronic transitions for compound **[1-F]**.

Wavelength [nm]	Oscillator strength (f)
533.2	0
532.6	0
403.1	0
402.7	0
399.2	0.025802132
398.1	0.293075708
377.8	0
377.7	0
371.7	0
371.5	0
361.4	0
361.3	0
354.6	4.361E-5
354.4	4.8983E-5
350	0
349.8	0
330.4	0.001736554
330.2	0.00164204
323.6	0
323.4	0
311.3	0.094632135
311.2	0.037957755
302	0
302	0
289.1	0
289	0
278.7	0
278.6	0
278.3	3.140121082
272.3	0.107028194
271.8	0.018919125
268.5	0.185208177
263	0
261.5	0
261	0
260.9	0
259.5	0
259.4	0
258.8	0.220276148
258.6	0.128340056
256	0
254.7	0
253.5	0
253.4	0
251.9	0.134953974
251.1	0.571394804
244.7	0
244.2	0
234	0.001998585
233.5	0.004467563

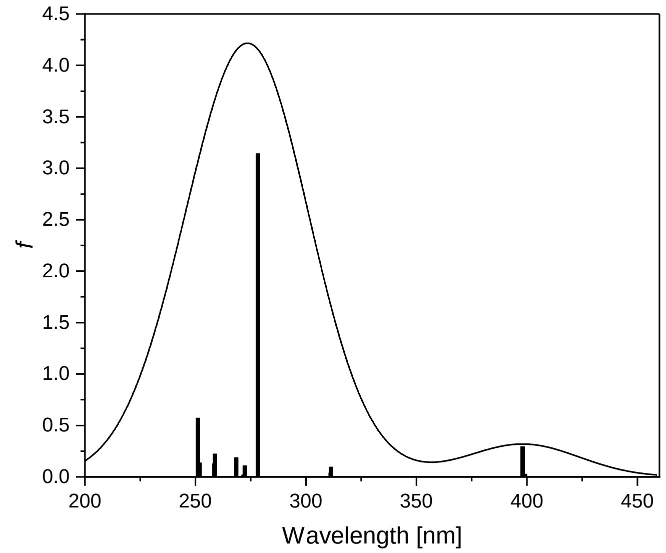

Figure S3.8. Simulated UV-vis spectrum of **[1-F]** in DCM (ωB97X-D3/def2-TZVPP/SMD(DCM)). Gaussian line broadening with 60 nm FWHM.

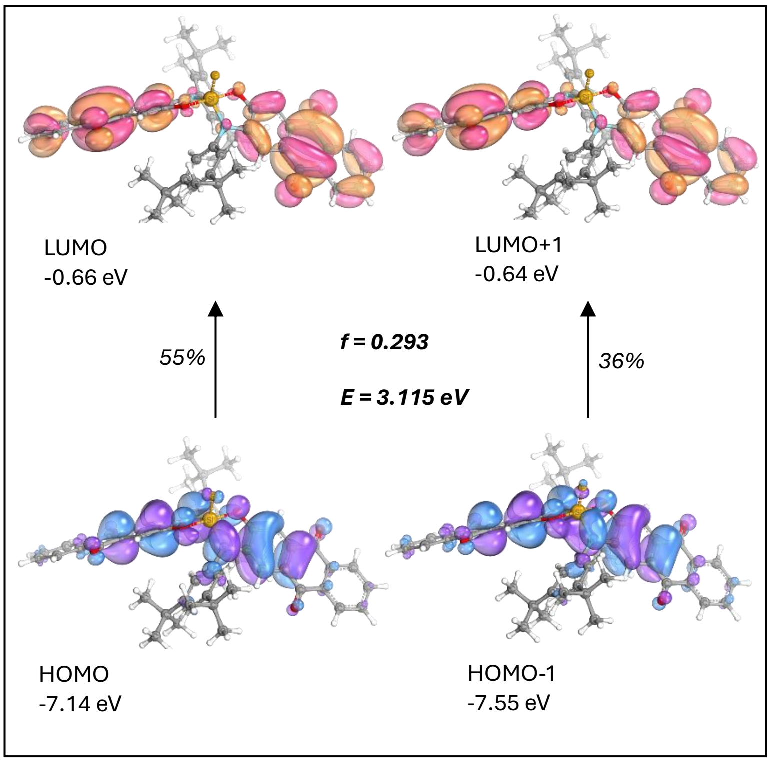

Figure S3.9. Molecular orbitals involved in the low energy CT transition of **[1-F]**. HOMO->LUMO 55%, HOMO-1->LUMO+1 36%.

[1-Cl]

Table S3.18. Calculated TD-DFT/TDA (*ωB97X-D3/def2-TZVPP/SMD(DCM))* electronic transitions for compound **[1-Cl]**.

Wavelength [nm]	Oscillator strength (f)
529.3	0
528.9	0
403.3	0
402.7	0
397.4	0.025805318
396.2	0.288521559
376.5	0
376.5	0
371.5	0
371.3	0
361.6	0
361.6	0
354.7	3.9946E-5
354.3	4.7219E-5
349	0
348.9	0
330.4	0.001686103
330.2	0.001638619
323.8	0
323.7	0
310.5	0.066185802
310.5	0.058732972
302.5	0
302.5	0
289.2	0
289.1	0
278.8	0
278.7	0
278.4	3.228361991
272.5	0.110562274
272	0.012497789
268.7	0.17760997
263	0
261.9	0
261.4	0
261.3	0
259.5	0
259.3	0
258.7	0.063301244
258.6	0.263570194
256.4	0
255.3	0
253.4	0
253.3	0
252.9	0.142052311
252.3	0.57727372
245.1	0
244.6	0
234.3	0.001504853
233.9	0.003991975

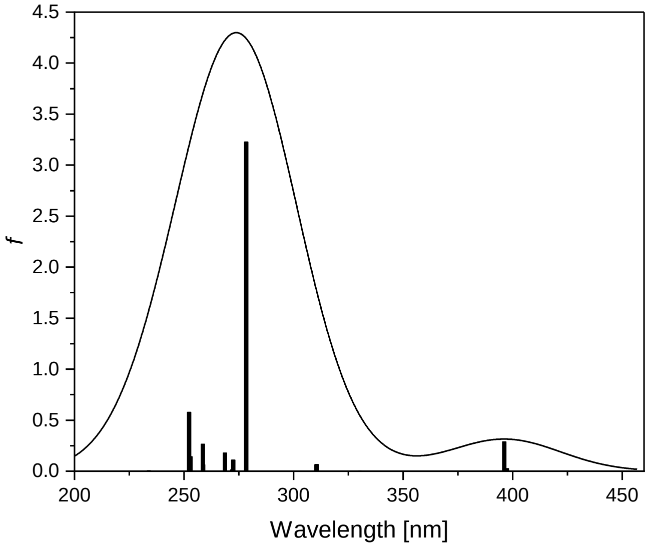

Figure S3.10. Simulated UV-vis spectrum of **[1-Cl]** in DCM (ωB97X-D3/def2-TZVPP/SMD(DCM)). Gaussian line broadening with 60 nm FWHM.

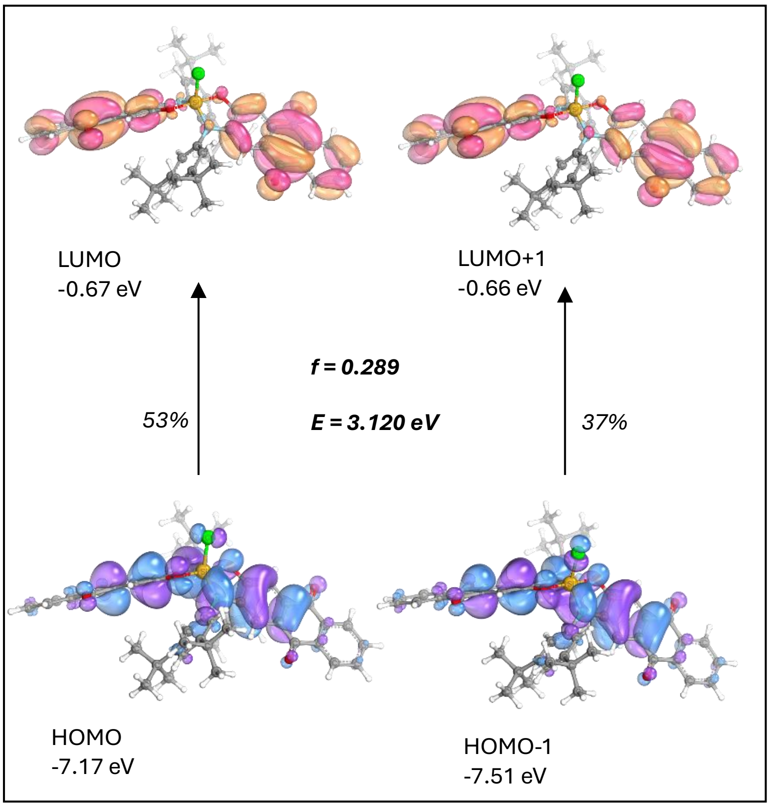

Figure S3.11. Molecular orbitals involved in the low energy CT transition of **[1-Cl]**. HOMO->LUMO 53%, HOMO-1->LUMO+1 37%.

[1-N_3]

Table S3.19. Calculated TD-DFT/TDA (*ωB97X-D3/def2-TZVPP/SMD(DCM))* electronic transitions for compound **[1-N_3]**.

Wavelength [nm]	Oscillator strength (f)
532.6	0
529.9	0
403.1	0
402.8	0
398.6	0.073230648
397.1	0.246884016
376.6	0
375.9	0
371.5	0
371	0
361.6	0
361.5	0
354.6	3.002E-5
354.4	5.4681E-5
349.9	0
349.5	0
330.3	0.001633076
330.2	0.001556745
323.8	0
323.6	0
310.7	0.06372911
310.2	0.058074431
302.1	0
302	0
288.7	0
288.5	0
278.8	0
278.6	0
278.3	3.119970084
272.5	0.136738991
271.9	0.01725935
269	0.22031698
266.7	0
263.2	0
261.5	0
261.2	0
261.1	0
259.7	0
259.5	0
259	0.06136341
259	0.234703321
257.1	0
256.4	1E-9
255.6	0
253.4	0
253.4	0
253	0.160128394
252.3	0.603425892
245.3	1E-9
244.7	0

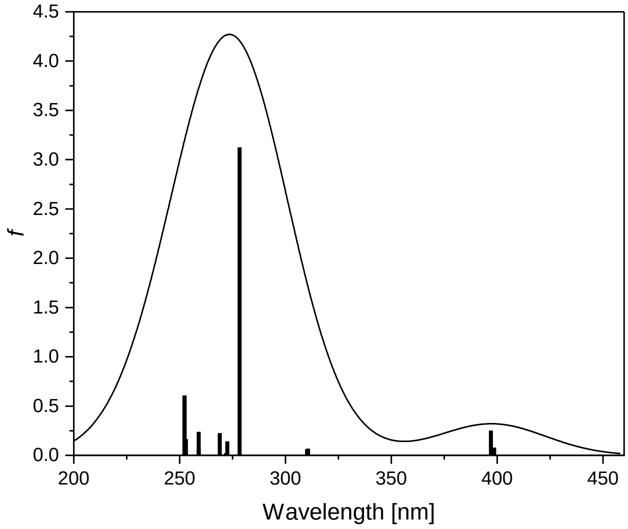

Figure S3.12. Simulated UV-vis spectrum of **[1-N_3]** in DCM (ωB97X-D3/def2-TZVPP/SMD(DCM)). Gaussian line broadening with 60 nm FWHM.

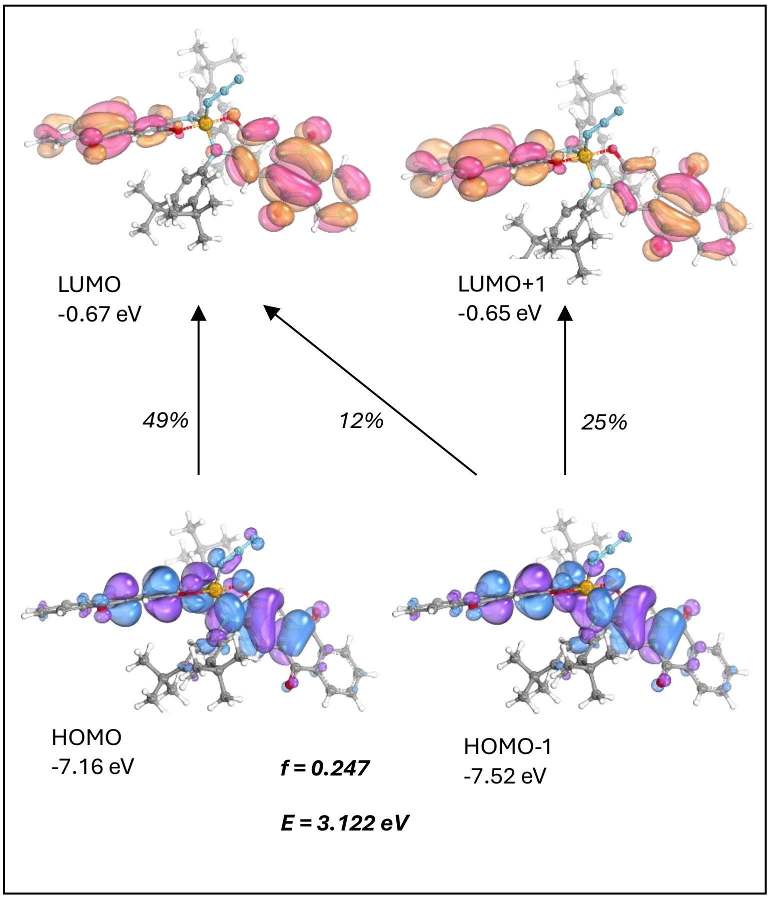

Figure S3.13. Molecular orbitals involved in the low energy CT transition of **[1-N_3]**. HOMO->LUMO 49%, HOMO-1->LUMO+1 25%, HOMO-1 -> LUMO 12%.

[1-NCS]

Table S3.20. Calculated TD-DFT/TDA (*ωB97X-D3/def2-TZVPP/SMD(DCM))* electronic transitions for compound **[1-NCS]**.

Wavelength [nm]	Oscillator strength (f)
520.1	0
519.9	0
403.5	0
403.1	0
391.1	0.025159467
390	0.285337077
374.7	0
374.6	0
370.3	0
370.3	0
362	0
362	0
354.7	3.9076E-5
354.5	4.6615E-5
345.1	0
344.8	0
330.5	0.001442794
330.4	0.001371714
324.3	0
324.1	0
308.4	0.039288127
308.2	0.101147587
301.9	0
301.8	0
288.3	0
288.2	0
278.9	0
278.9	0
276.2	3.15977973
272.8	0.097839368
272	0.042361339
266.6	0.250136649
261.7	0
261.4	0
260.9	0
260.7	0
258.7	0
258.6	0
256.3	0.144279931
255.9	0.475212573
254.2	0
253.6	0
253.1	0
253	0
252.7	0.109810115
252.2	0.325587338
244.8	0
244.2	0
233.5	0.000995246
233	0.00143284

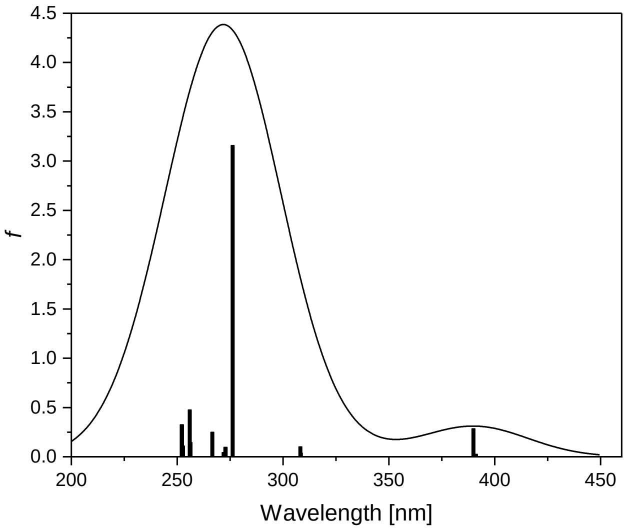

Figure S3.14. Simulated UV-vis spectrum of **[1-NCS]** in DCM (ωB97X-D3/def2-TZVPP/SMD(DCM)). Gaussian line broadening with 60 nm FWHM.

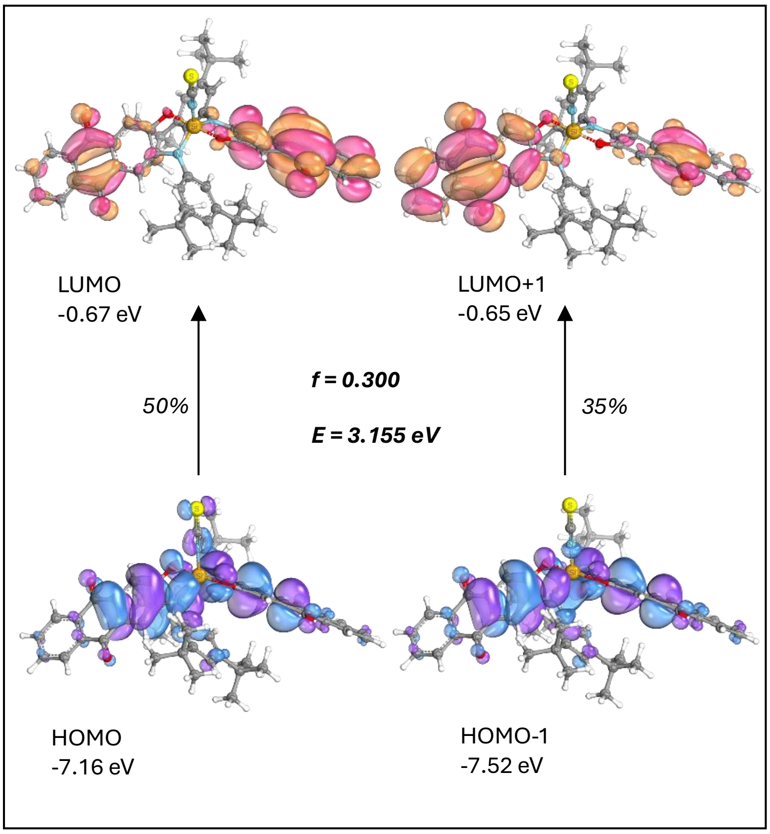

Figure S3.15. Molecular orbitals involved in the low energy CT transition of **[1-NCS]**. HOMO->LUMO 50%, HOMO-1->LUMO+1 35%.

[1-CN]

Table S3.21. Calculated TD-DFT/TDA (*ωB97X-D3/def2-TZVPP/SMD(DCM))* electronic transitions for compound **[1-CN]**.

Wavelength [nm]	Oscillator strength (f)
520.1	0
519.9	0
403.5	0
403.1	0
391.1	0.025159467
390	0.285337077
374.7	0
374.6	0
370.3	0
370.3	0
362	0
362	0
354.7	3.9076E-5
354.5	4.6615E-5
345.1	0
344.8	0
330.5	0.001442794
330.4	0.001371714
324.3	0
324.1	0
308.4	0.039288127
308.2	0.101147587
301.9	0
301.8	0
288.3	0
288.2	0
278.9	0
278.9	0
276.2	3.15977973
272.8	0.097839368
272	0.042361339
266.6	0.250136649
261.7	0
261.4	0
260.9	0
260.7	0
258.7	0
258.6	0
256.3	0.144279931
255.9	0.475212573
254.2	0
253.6	0
253.1	0
253	0
252.7	0.109810115
252.2	0.325587338
244.8	0
244.2	0
233.5	0.000995246
233	0.00143284

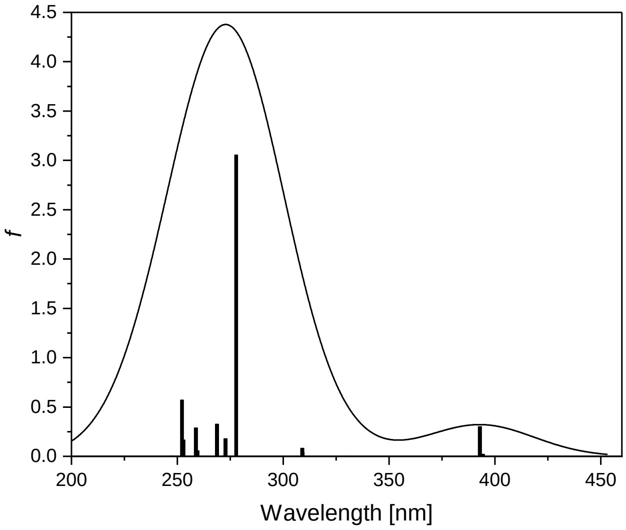

Figure S3.16. Simulated UV-vis spectrum of **[1-CN]** in DCM (ωB97X-D3/def2-TZVPP/SMD(DCM)). Gaussian line broadening with 60 nm FWHM.

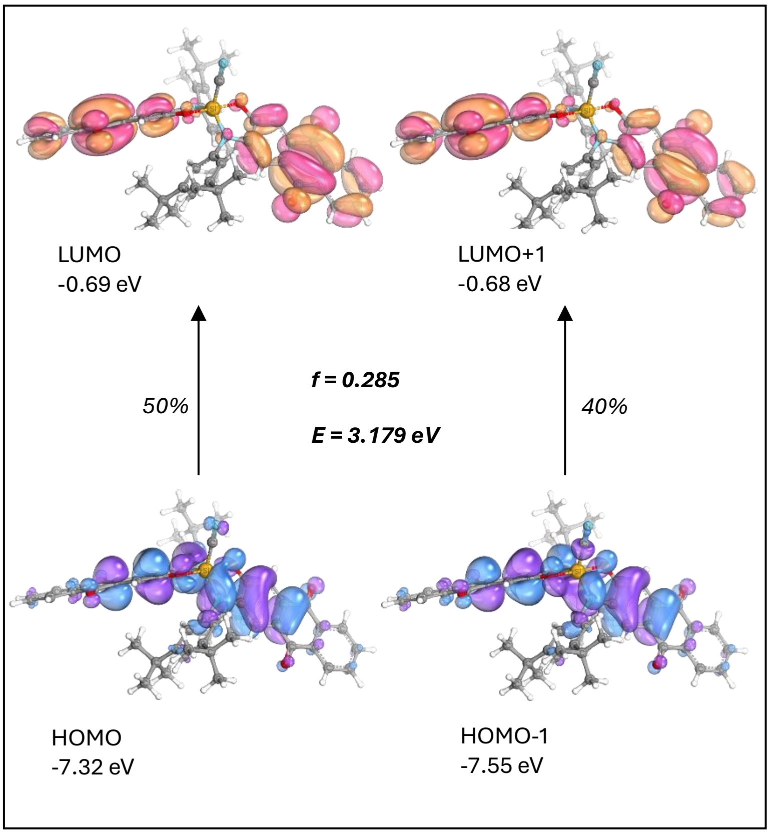

Figure S3.17. Molecular orbitals involved in the low energy CT transition of **[1-CN]**. HOMO->LUMO 50%, HOMO-1->LUMO+1 40%.

[1-Br]

Table S3.22. Calculated TD-DFT/TDA (*ωB97X-D3/def2-TZVPP/SMD(DCM))* electronic transitions for compound **[1-Br]**.

Wavelength [nm]	Oscillator strength (f)
529.2	0
528.4	0
402.8	0
402.1	0
398	0.049951323
396.6	0.266287253
376.2	0
375.2	0
371	0
370.8	0
361.5	0
361.4	0
354.4	6.4963E-5
353.9	4.9516E-5
350.4	0
350.1	0
330.3	0.001250147
329.9	0.001670834
323.7	0
323.5	0
310.8	0.048901049
309.9	0.057584092
306.2	0
302.2	0
289.6	0
288.5	0
279.7	3.135070573
278.6	0
278.6	0
272.6	0.160496998
272	0.027552118
269.4	0.198909285
263.9	0
262.7	0
261.8	0
261.8	0.098893876
261.2	0
260	0
259.2	0
259	0.147851784
258.1	0
256.4	0
254.2	0.272268629
253.6	0
253.5	0
253.4	0.543782039
246	0
245	0
235	0.001353489
234.1	0.00285886

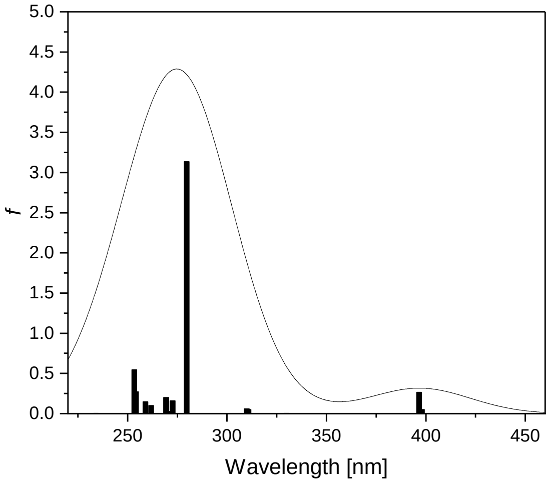

Figure S3.18. Simulated UV-vis spectrum of **[1-Br]** in DCM (ωB97X-D3/def2-TZVPP/SMD(DCM)). Gaussian line broadening with 60 nm FWHM.

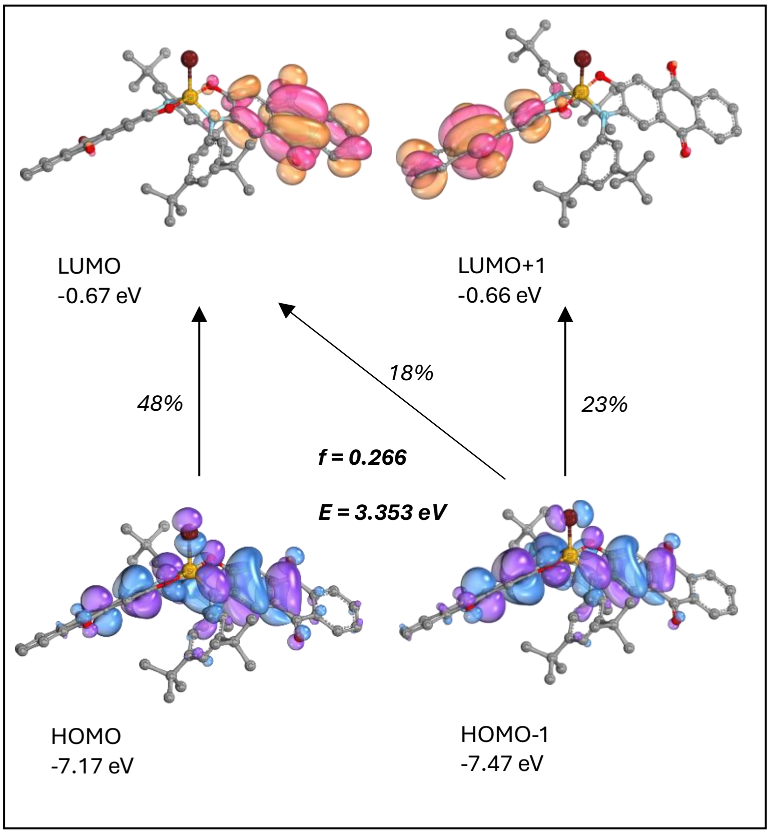

Figure S3.19. Molecular orbitals involved in the low energy CT transition of **[1-Br]**. HOMO->LUMO 48%, HOMO-1->LUMO+1 23%. HOMO-1->LUMO 18%.

1-pyridine

Table S3.23. Calculated TD-DFT/TDA (*ωB97X-D3/def2-TZVPP/SMD(DCM))* electronic transitions for compound **1-pyridine**.

Wavelength [nm]	Oscillator strength (f)
483.3	0
483.1	0
402.7	0
402.3	0
372.5	0
372.2	0
371.1	0.016970852
369.8	0.274947103
364.2	0
364	0
360.8	0
360.8	0
353.7	0.000153788
353.5	0.00018907
334.3	0
333.9	0
329.5	0.000768816
329.3	0.000707551
326.2	0
326.2	0
304	0
303.9	0
301.6	0.123837256
301.5	0.008874665
293.7	0
288.8	0
288.7	0
287.5	0
279.4	0
279.4	0
274.8	1.681723192
274	0.045133681
270.5	1.694062133
264.8	0.135528855
263.2	0
263.1	0
262.7	0
262.5	0
260.1	0.033434062
259.9	0
255.4	0.14890743
255.3	0.809717086
253.8	0
253.7	0
252.7	0
252.7	0
249.3	0
246.2	9.35E-7
246	2.252E-6
245.9	0.008528666

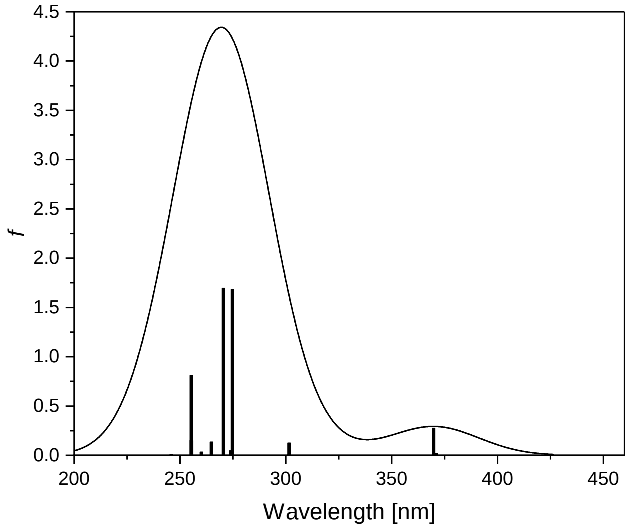

Figure S3.20. Simulated UV-vis spectrum of **1-pyridine** in DCM (ωB97X-D3/def2-TZVPP/SMD(DCM)). Gaussian line broadening with 50 nm FWHM.

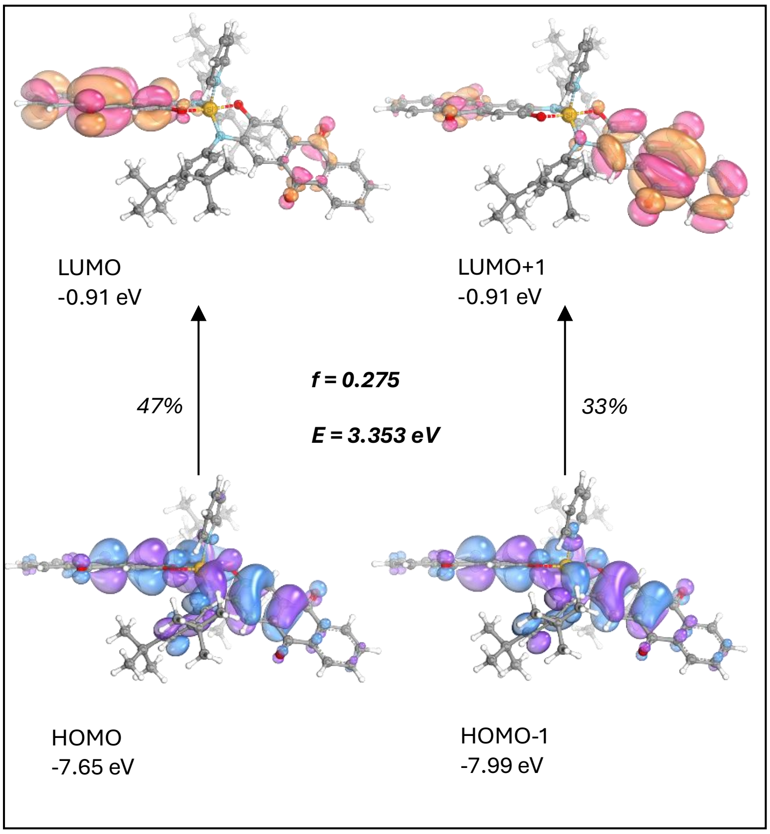

Figure S3.21. Molecular orbitals involved in the low energy CT transition of **1-pyridine**. HOMO->LUMO 47%, HOMO-1->LUMO+1 33%.

1-DMAP

Table S3.24. Calculated TD-DFT/TDA (*ωB97X-D3/def2-TZVPP/SMD(DCM)*) electronic transitions for compound **1-DMAP**.

Wavelength [nm]	Oscillator strength (f)
491.9	0
491.7	0
403.2	0
402.3	0
376	0.021298575
374.9	0.270769199
372.9	0
372.3	0
363.7	0
363.7	0
363	0
363	0
354.1	0.000148322
353.7	0.000169759
336.9	0
336.8	0
336	0
329.7	0.00088639
329.4	0.000830724
326	0
325.9	0
303.3	0
303.2	0
303.2	0.104842753
303.2	0.03152555
288.9	0
288.8	0
283.4	0
279.2	0
279.2	0
274.7	1.908440697
273.7	0.069481417
271	1.409159086
265.2	0.243351212
262.6	0
262.4	0
262.2	1E-9
261.9	1E-9
257.5	0
256	0.233883416
255.8	0.685577652
255.1	1E-9
254.7	0
252.8	1E-9
252.7	1E-9
247.2	4E-9
246.6	3E-9
246.1	0.46055255
245.5	0.186135296
245	0.289665931

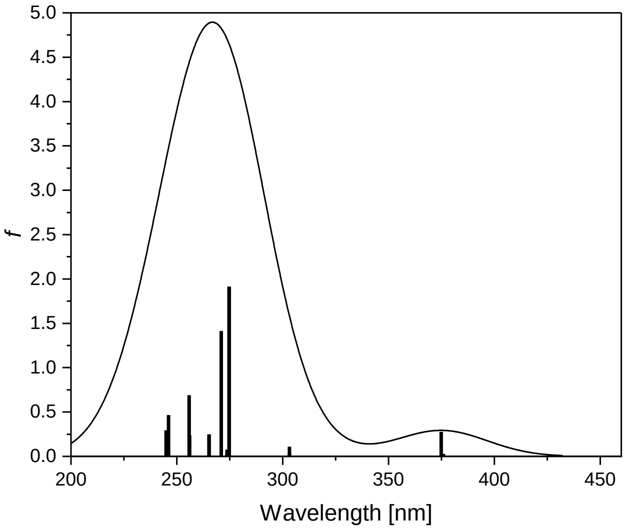

Figure S3.22. Simulated UV-vis spectrum of **1-DMAP** in DCM (ωB97X-D3/def2-TZVPP/SMD(DCM). Gaussian line broadening with 50 nm FWHM.

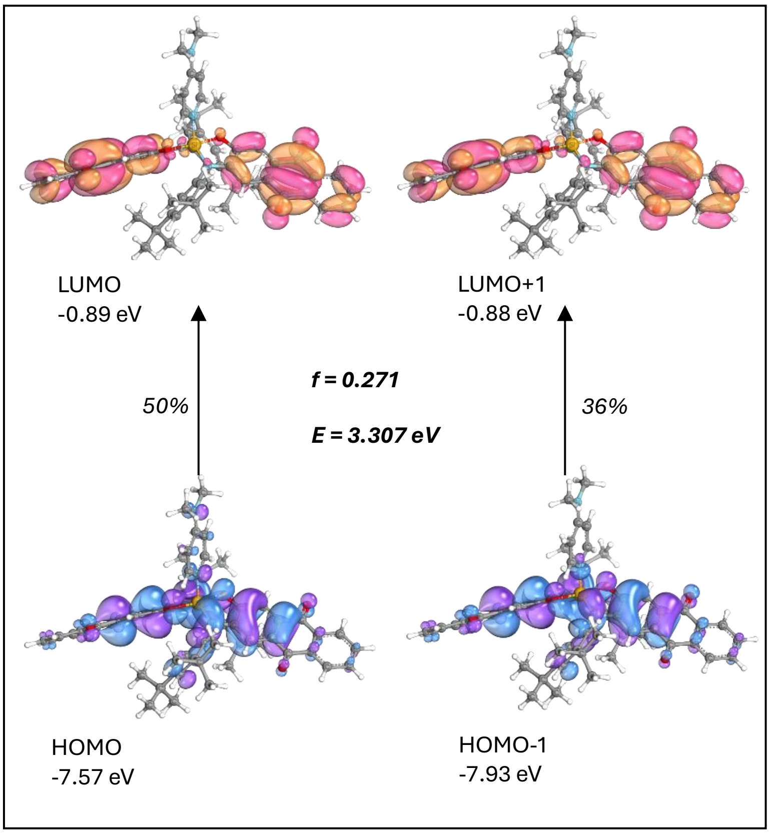

Figure S3.23. Molecular orbitals involved in the low energy CT transition of **1-DMAP**. HOMO->LUMO 50%, HOMO-1->LUMO+1 36%.

1-OPEt$_3$

Table S3.25. Calculated TD-DFT/TDA (*ωB97X-D3/def2-TZVPP/SMD(DCM)*) electronic transitions for compound **1-OPEt$_3$**.

Wavelength [nm]	Oscillator strength (f)
497.9	0
494.4	0
403.2	0
402.6	0
378.9	0.102247235
377	0.206197776
372.6	0
372.5	0
364.6	0
364.1	0
363.3	0
362.8	0
354.3	0.000200295
353.8	8.8693E-5
339.5	0
338.3	0
329.7	0.000462897
329.5	0.001028722
326.3	0
325.7	0
304.5	0.065229517
303.4	0.064934387
303.1	0
302.2	0
288.8	0
288.5	0
279.1	0
279.1	0
274.8	1.986582888
273.7	0.156103364
271.3	1.143552997
264.6	0.135927957
262.7	0
262.4	0
261.2	0
260.9	0
256.1	0.450549779
256	0
255.4	0.443175597
255.1	0
252.9	0
252.7	0
249.7	0
248.2	0.154547549
245.6	0
244.8	0
244.2	0
243.4	0.076804383
234.4	0.002983195
233.2	0.001408145

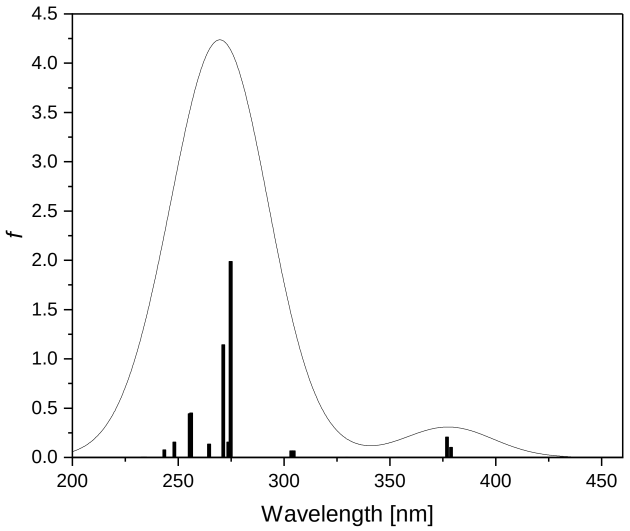

Figure S3.24. Simulated UV-vis spectrum of **1-OPEt$_3$** in DCM (ωB97X-D3/def2-TZVPP/SMD(DCM). Gaussian line broadening with 50 nm FWHM. Two low-energy transitions have significant oscillator strength. The maximum was determined from the broadened spectrum to be λ_{max} = 377.5 nm.

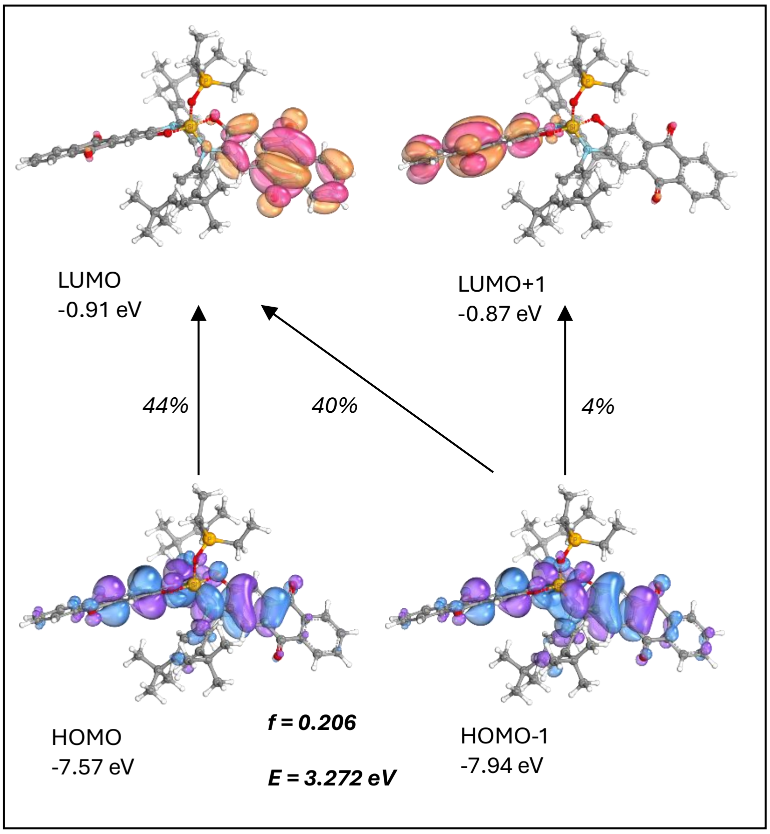

Figure S3.25. Molecular orbitals involved in the low energy CT transition of **1-OPEt$_3$**. HOMO->LUMO 44%, HOMO-1->LUMO 40%, HOMO-1->LUMO+1 4%.

1-HMPA

Table S3.26. Calculated TD-DFT/TDA (*ωB97X-D3/def2-TZVPP/SMD(DCM)*) electronic transitions for compound **1-HMPA**.

Wavelength [nm]	Oscillator strength (f)
500	0
498.2	0
403.2	0
402.3	0
381.2	0.080370104
379.1	0.228250048
373.1	0
372.2	0
365.8	0
365.6	0
363.6	0
363.4	0
354.2	0.000113913
353.8	0.000146997
341.7	0
339.1	0
329.8	0.001163103
329.4	0.000625599
326.1	0
325.9	0
305.9	0.062634801
304.9	0.067922569
304.7	0
302.5	0
289.8	0
289.1	0
279.1	0
279.1	0
275.6	2.721545079
273.6	0.073732821
272.1	0.403223887
265.7	0.227438416
264	0
263.6	0
261.9	0
261.4	0
256.3	0.244825591
256.1	0
256	0.584995586
255.5	0
252.9	0
252.8	0
249.7	0
248	0.145315433
247.4	0
246.7	0
245.8	0.119380987
245	0
235.8	0.00182637
233.7	0.001715917

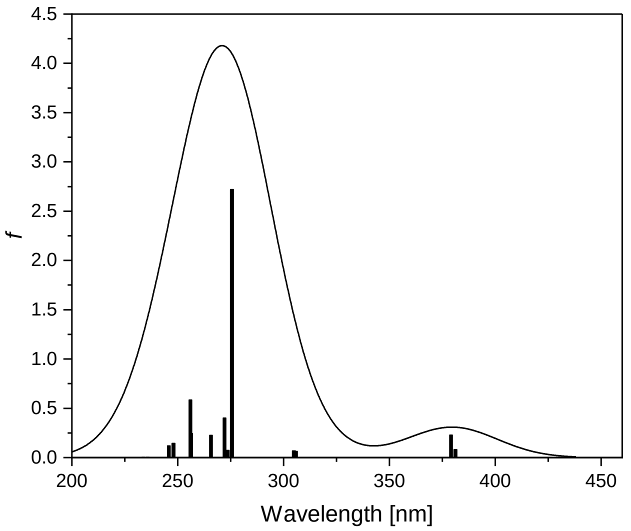

Figure S3.26. Simulated UV-vis spectrum of **1-HMPA** in DCM (ωB97X-D3/def2-TZVPP/SMD(DCM). Gaussian line broadening with 50 nm FWHM. Two low-energy transitions have significant oscillator strength. The maximum was determined from the broadened spectrum to be λ_{max} = 379.5 nm.

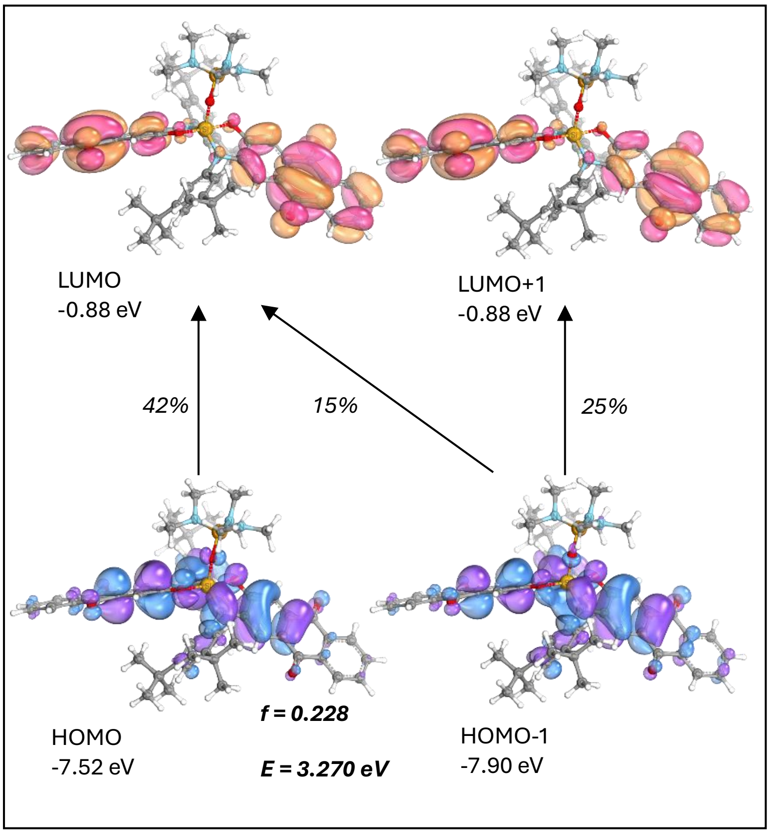

Figure S3.27. Molecular orbitals involved in the low energy CT transition of **1-HMPA**. HOMO->LUMO 44%, HOMO-1->LUMO 15%, HOMO-1->LUMO+1 25%.

1-DMSO

Table S3.27. Calculated TD-DFT/TDA (*ωB97X-D3/def2-TZVPP/SMD(DCM))* electronic transitions for compound **1-DMSO**.

Wavelength [nm]	Oscillator strength (f)
489.4	0
484.5	0
403.2	0
402.4	0
374.2	0.119425222
372.9	0
372.1	0
371	0.188668851
364.5	0
363.8	0
362.4	0
359.8	0
354.1	0.00014697
353.7	0.000283979
337.1	0
334.2	0
329.7	0.000977567
329.4	0.000407797
326.7	0
326	0
303.7	0
303.1	0.060518529
302.8	0
301.1	0.06435606
289.3	0
287.8	0
279.4	0
279.2	0
274.9	1.614711304
274	0.156276581
270.8	1.513276487
264	0.118359406
262.9	0
262.8	0
262	0
261.4	0
255.4	0.568223147
255.4	0.379537592
254.8	0
254	0
252.7	0
252.6	0
248.6	0
247.4	0.138359404
245.9	0
245.2	0
242.3	0
241.3	0.054251447
234.9	0.003805773
233.8	0.001047039

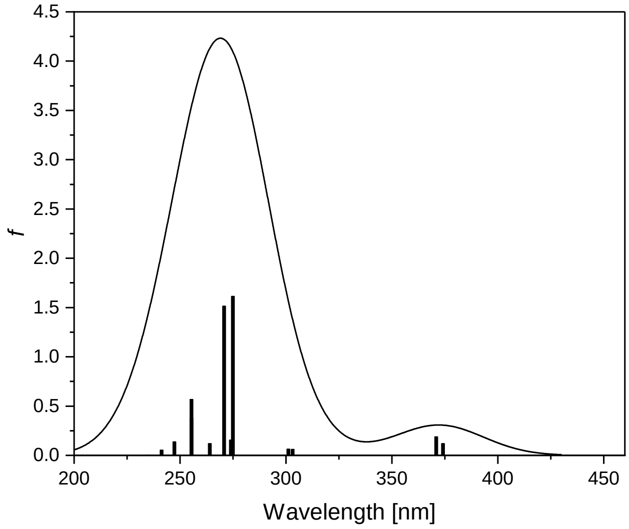

Figure S3.28. Simulated UV-vis spectrum of **1-DMSO** in DCM (ωB97X-D3/def2-TZVPP/SMD(DCM). Gaussian line broadening with 50 nm FWHM. Two low-energy transitions have significant oscillator strength. The maximum was determined from the broadened spectrum to be λ_{max} = 372.1 nm.

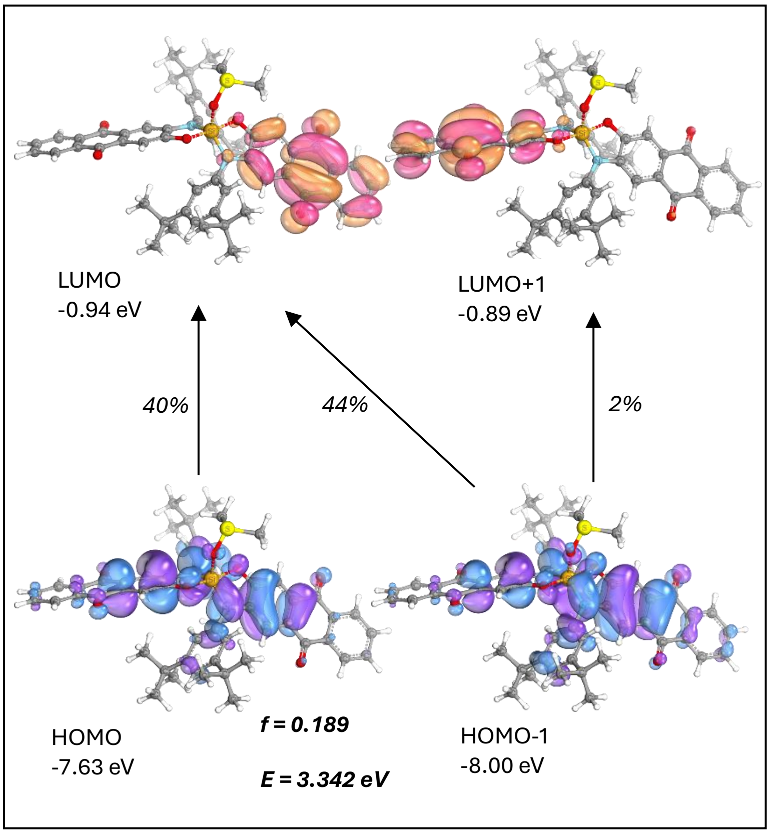

Figure S3.29. Molecular orbitals involved in the low energy CT transition of **1-DMSO**. HOMO->LUMO 40%, HOMO-1->LUMO 44%, HOMO-1->LUMO+1 2%.

1-DIBA

Table S3.28. Calculated TD-DFT/TDA (*ωB97X-D3/def2-TZVPP/SMD(DCM))* electronic transitions for compound **1-DIBA**.

Wavelength [nm]	Oscillator strength (f)
495.4	0
492.7	0
403	0
402.1	0
378.6	0.028609959
377.1	0.293895052
373.4	0
371.9	0
365.9	0
364.6	0
363.8	0
363.6	0
354.1	9.885E-6
353.6	0.000277779
342.1	0
340.9	0
329.9	0.001619901
329.3	0.000137246
326.3	0
326.1	0
310.3	0
307.4	0
306	0.064882755
304.7	0.046164268
303.1	0
291.4	0
290.4	0
279.2	0
279.2	0
278.6	0
277.4	2.890655567
274	0.098714576
272.6	0.064619981
268.4	0.291910811
267.7	0
266.9	0
265.5	0
265.1	0.000554392
263.1	0
262.4	0
261.5	0
258.4	0
256.7	0.27537162
255.7	0.553446569
255.5	0
255.1	0
253	0
252.8	0
251.1	0
249.5	0.156129122

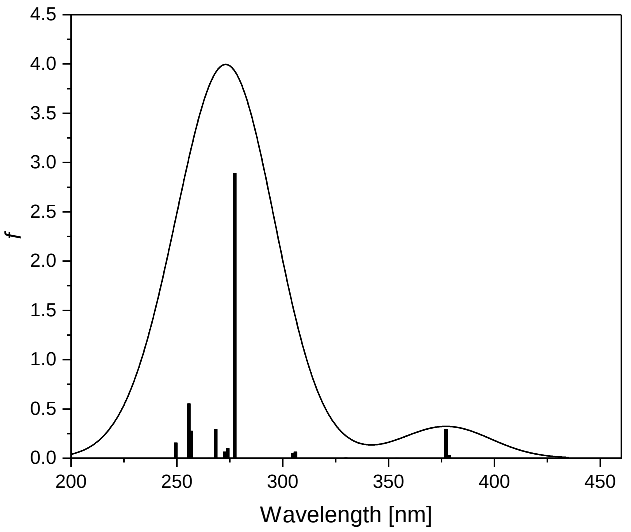

Figure S3.30. Simulated UV-vis spectrum of **1-DIBA** in DCM (ωB97X-D3/def2-TZVPP/SMD(DCM). Gaussian line broadening with 50 nm FWHM.

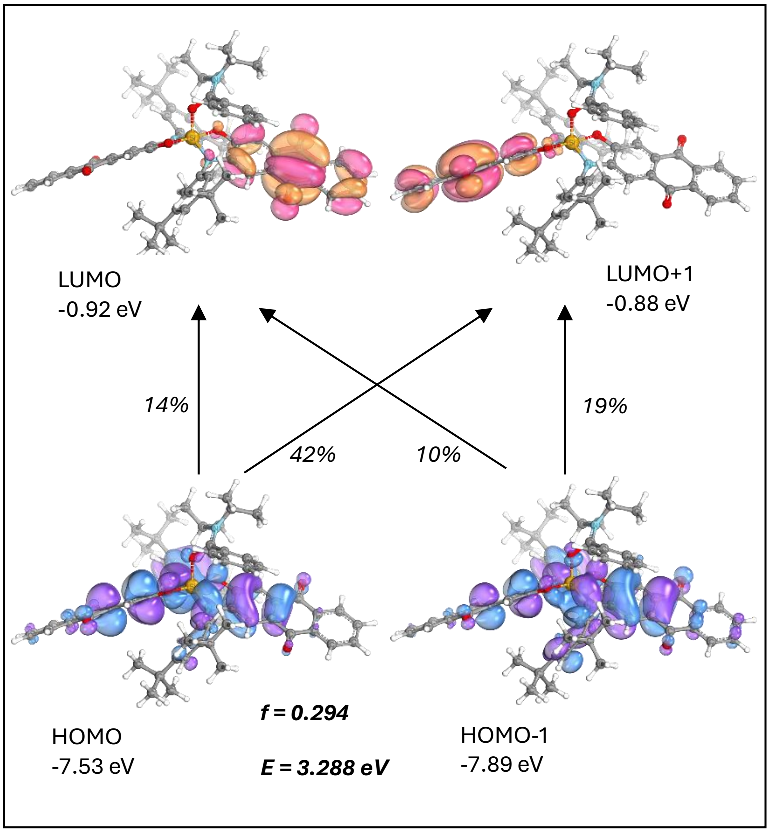

Figure S3.31. Molecular orbitals involved in the low energy CT transition of **1-DIBA**. HOMO->LUMO 14%, HOMO-1->LUMO 10%, HOMO-1->LUMO+1 19%, HOMO -> LUMO+1 42%.

1-^{dipp}NHC

Table S3.29. Calculated TD-DFT/TDA (*ωB97X-D3/def2-TZVPP/SMD(DCM))* electronic transitions for compound **1-^{dipp}NHC**.

Wavelength [nm]	Oscillator strength (f)
506.4	0
499.8	0
402.8	0
402.8	0
386.3	0.117731782
381.9	0.194055568
372.5	0
372.5	0
372.4	0
368.3	0
363.3	0
363	0
354.3	0.00031863
354.2	0.000208581
342.4	0
339.7	0
329.9	0.000269825
329.8	0.000498366
325.9	0
325.7	0
311.6	0
310	0.079376332
308.4	0
306.9	0.076954866
304.4	0
304.3	0
303.9	0
291.6	0
289.2	0
278.9	0
278.8	0
277.7	2.783039868
273.5	0.181659116
272.5	0.009612058
269.4	0.280574247
267.4	0
264.7	0
264.6	0
264.2	0
263.5	0
263.4	0
263.3	0
262.4	0
259.5	0
258.9	0.074956339
257.8	0
257.6	0.40053585
255.7	0
254.9	0.329252292
254	0

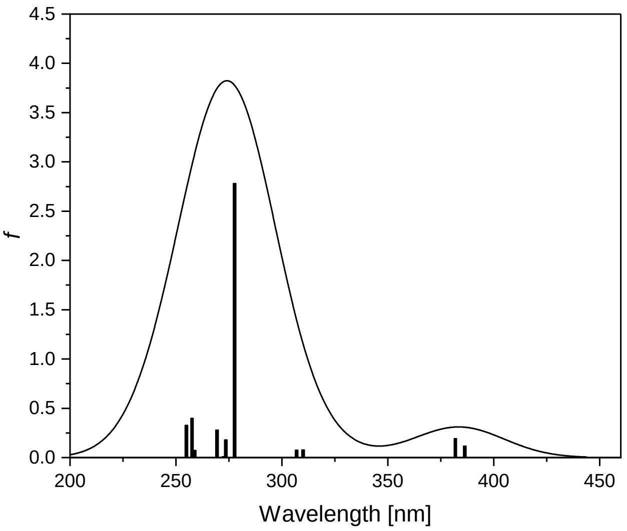

Figure S3.32. Simulated UV-vis spectrum of **1-^{dipp}NHC** in DCM (ωB97X-D3/def2-TZVPP/SMD(DCM). Gaussian line broadening with 50 nm FWHM. Two low-energy transitions have significant oscillator strength. The maximum was determined from the broadened spectrum to be λ_{max} = 383.5 nm.

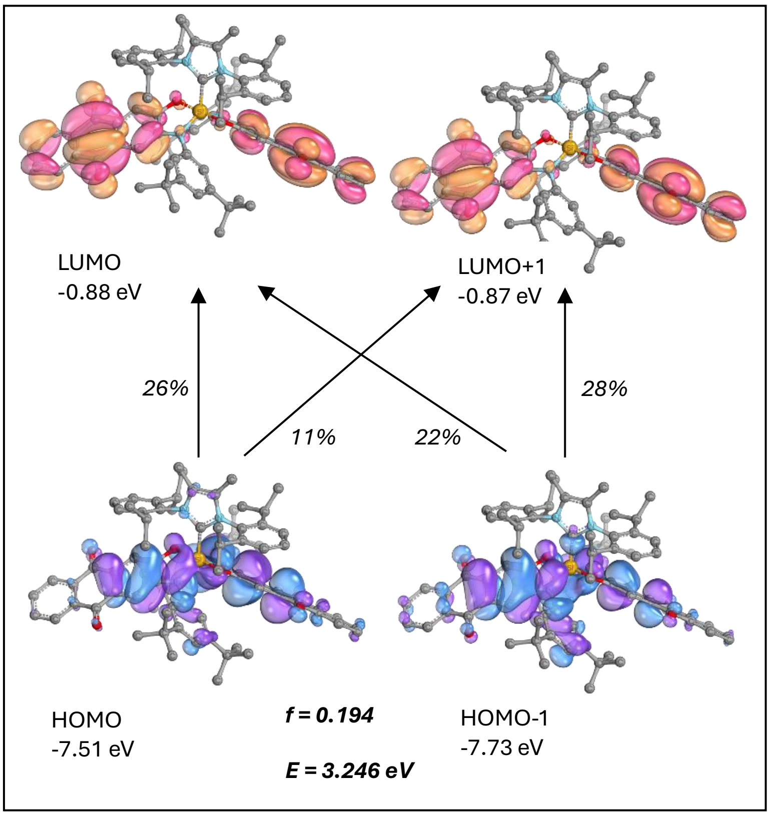

Figure S3.33. Molecular orbitals involved in the low energy CT transition of **1-^{dipp}NHC**. HOMO->LUMO 26%, HOMO-1->LUMO 22%, HOMO-1->LUMO+1 28%, HOMO -> LUMO+1 11%.

1-SIMes

Table S3.30. Calculated TD-DFT/TDA (*ωB97X-D3/def2-TZVPP/SMD(DCM)*) electronic transitions for compound **1-SIMes**.

Wavelength [nm]	Oscillator strength (f)
501.5	0
501.1	0
403.3	0
403.2	0
383.4	0.025102775
382.6	0.243971259
373.8	0
373.7	0
368.9	0
368.6	0
363.9	0
363.7	0
354.6	0.000696485
354.5	0.000833841
342.1	0
341.6	0
330.3	0.001567409
330.3	0.001546263
326.5	0
326.4	0
308.1	0.044309426
307.8	0.130724604
306.8	0
306.6	0
306.6	0
306.2	0
290.6	0
290.4	0
290	0
279.1	0
279	0
277.3	3.010884782
274.1	0.061292152
273.3	0.030784605
269	0.054541686
266.2	0
266.1	0
264.5	0
264.4	0
263.4	0
263.3	0
261.9	0
261.1	0
260.5	0
260	0
258.3	0.128191735
258	0.55954596
254.6	0
254.6	0
254.5	0.03759143

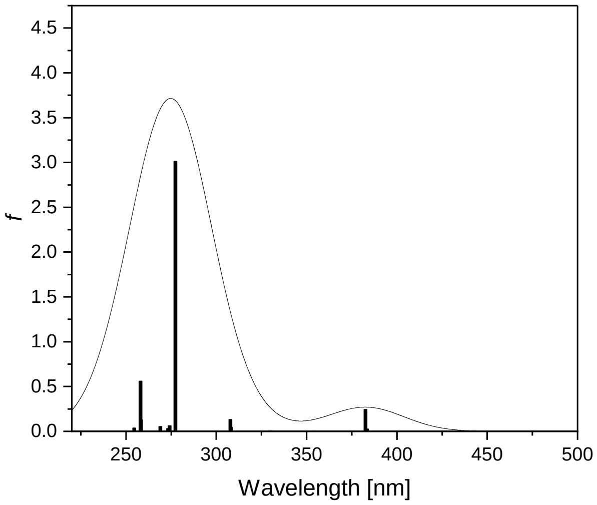

Figure S3.34. Simulated UV-vis spectrum of **1-SIMes** in DCM (ωB97X-D3/def2-TZVPP/SMD(DCM). Gaussian line broadening with 50 nm FWHM. λ_{max} = 382.5 nm.

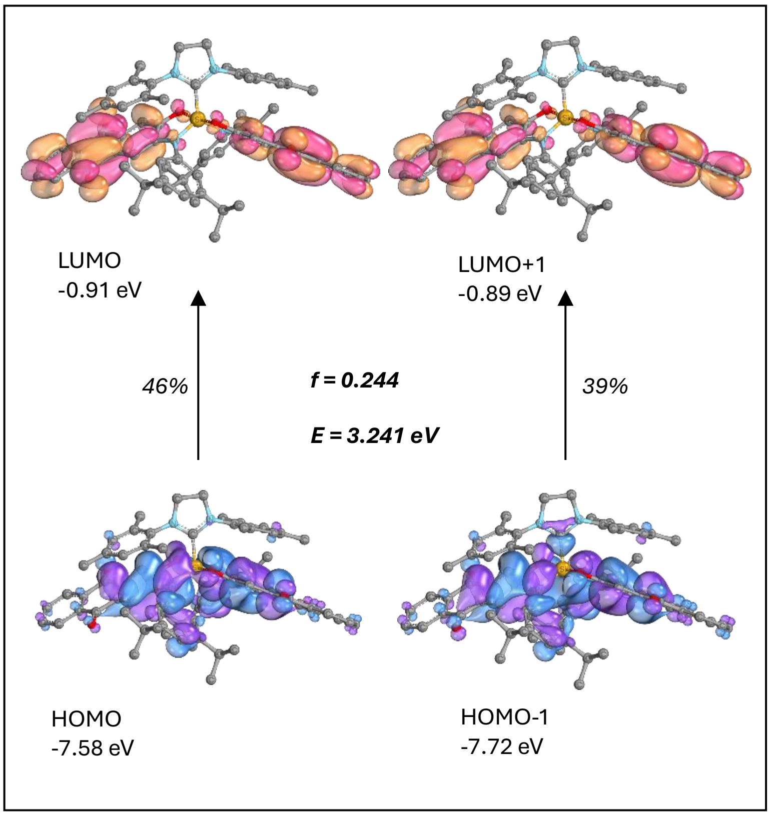

Figure S3.35. Molecular orbitals involved in the low energy CT transition of **1-SIMes**. HOMO->LUMO 46%, HOMO-1->LUMO+1 39%.

1-^{iPr}NHC

Table S3.31. Calculated TD-DFT/TDA (*ωB97X-D3/def2-TZVPP/SMD(DCM))* electronic transitions for compound **1-^{iPr}NHC**.

Wavelength [nm]	Oscillator strength (f)
500.9	0
499.9	0
402.3	0
402.3	0
381.9	0.04728963
380.8	0.251919463
372.4	0
372.3	0
363.7	0
363.7	0
363.5	0
363.5	0
353.8	0.000132128
353.8	0.000256024
342.7	0
342	0
329.5	0.000987088
329.5	0.000875121
325.9	0
325.8	0
306.1	0
305.7	0
305	0.013575356
304.8	0.085520644
288.8	0
288.8	0
279	0
279	0
276.6	2.98213764
273.8	0.130330215
272.9	0
272.5	0.19366517
267.7	0.247474881
262.5	0
262.4	0
260.8	0
260.3	0
256.9	0
256.4	0.201961164
256.3	0.606452701
256.2	0
253.1	0
253	0
252.8	0
252.3	0.079398086
252.1	0
251.5	0.222725174
250.9	1E-9
246.1	0
246	0

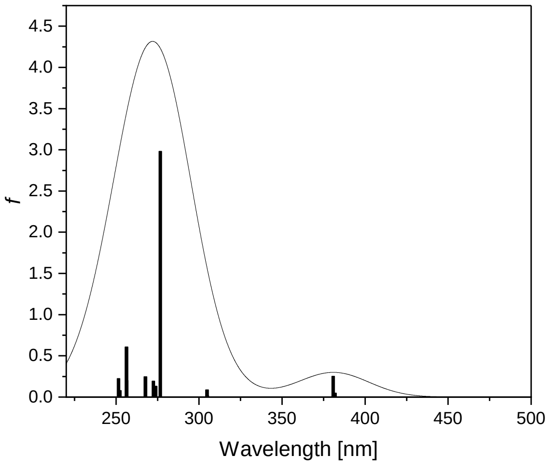

Figure S3.36. Simulated UV-vis spectrum of **1-^{iPr}NHC** in DCM (ωB97X-D3/def2-TZVPP/SMD(DCM). Gaussian line broadening with 50 nm FWHM. λ_{max} = 380.9 nm.

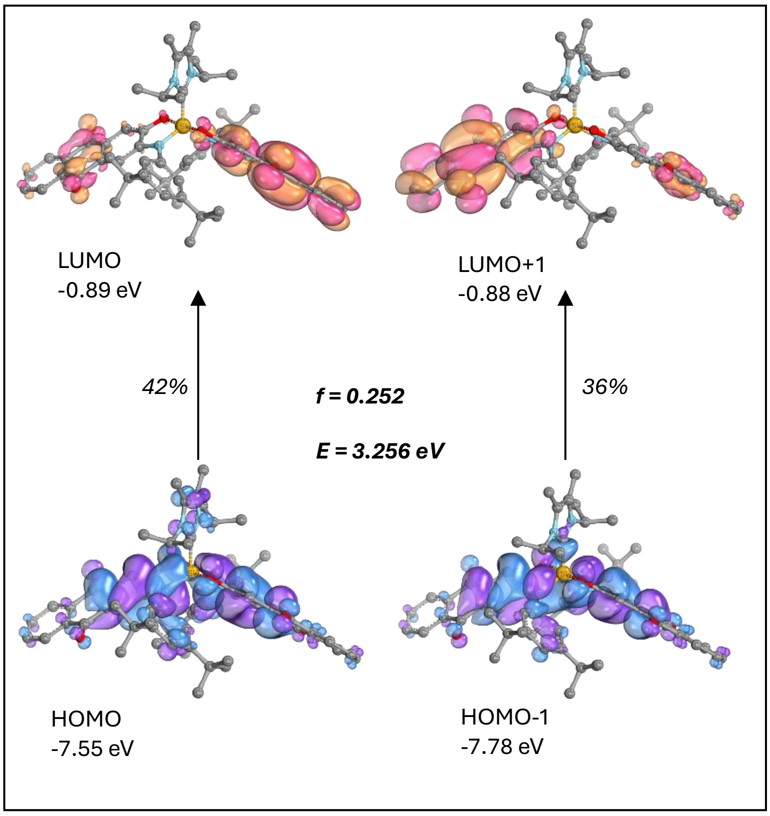

Figure S3.37. Molecular orbitals involved in the low energy CT transition of **1-^{iPr}NHC**. HOMO->LUMO 42%, HOMO-1->LUMO+1 36%.

1-P(*n*Bu)$_3$

Table S3.32. Calculated TD-DFT/TDA (*ωB97X-D3/def2-TZVPP/SMD(DCM))* electronic transitions for compound **1-P(*n*Bu)$_3$**.

Wavelength [nm]	Oscillator strength (f)
494	0
485.3	0
403.8	0
402.8	0
379.4	0.141867323
373.4	0
372.6	0.164280691
372.6	0
364.8	0
364.2	0
361.4	0
359.9	0
354.6	0.000329789
353.9	0.000182179
344.1	0
337.2	0
330.1	0.00119214
329.6	0.000809418
327	0
326.2	0
307.1	0
305	0.020531628
303.5	0
302.2	0.059609563
291.1	0
288.7	0
279.5	0
279.1	0
278.1	2.75876953
274.4	0.206443475
273.3	0.078805244
267.8	0.382284663
266.4	0
264.9	0
263.8	0
263	0
257.5	0
257.3	0.440870617
255.7	0
255.5	0.315260274
254.6	0.298176484
254.1	0
253.1	0
252.6	0
250.4	0
250.4	0.028559012
248.8	0
246.3	0
238.3	0.034856039
233.6	0

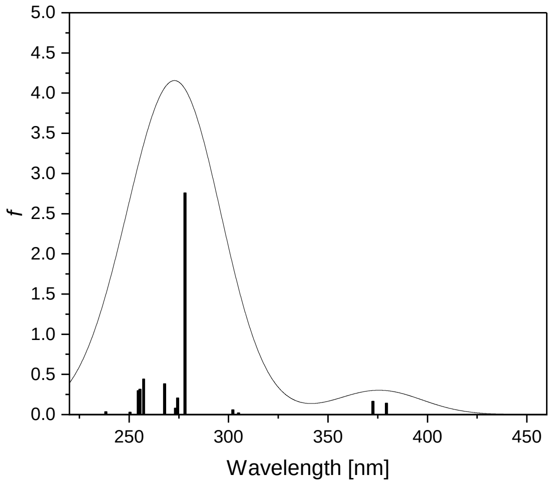

Figure S3.38. Simulated UV-vis spectrum of **1-P(nBu)$_3$** in DCM (ωB97X-D3/def2-TZVPP/SMD(DCM). Gaussian line broadening with 50 nm FWHM. λ_{max} = 375.6 nm.

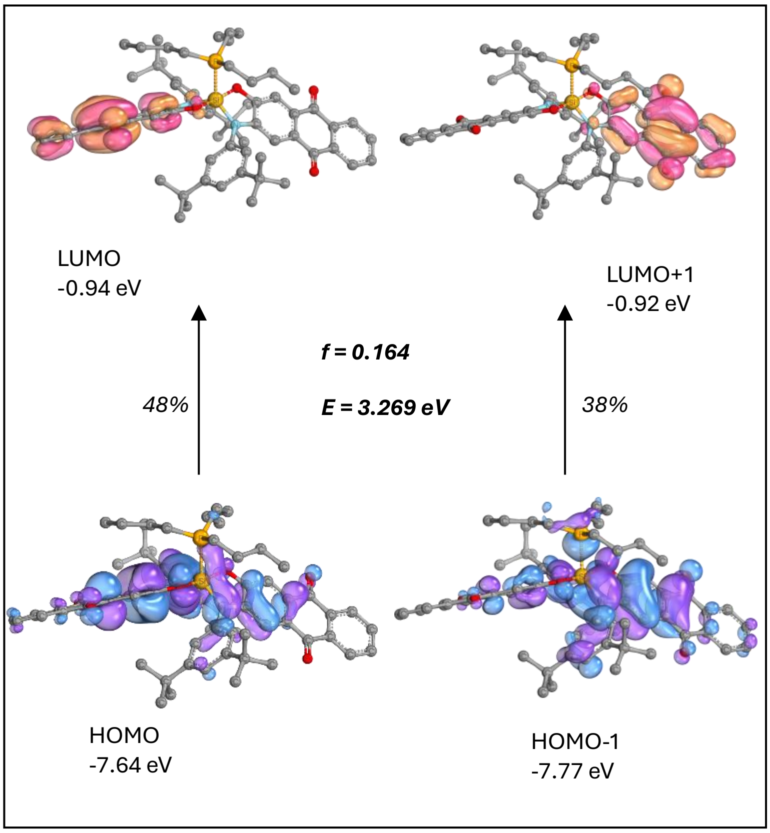

Figure S3.39. Molecular orbitals involved in the low energy CT transition of **1-P(nBu)$_3$**. HOMO->LUMO 48%, HOMO-1->LUMO+1 38%. Transition at 3.261 eV: HOMO->LUMO+1 46%, HOMO-1->LUMO 40%

1-PCy_3

Table S3.33. Calculated TD-DFT/TDA (*ωB97X-D3/def2-TZVPP/SMD(DCM))* electronic transitions for compound **1-PCy_3**.

Wavelength [nm]	Oscillator strength (f)
496.7	0
488.9	0
402.4	0
402.3	0
379.7	0.129711599
374.9	0.168019321
372.3	0
372.2	0
363.9	0
363.9	0
361.4	0
360.9	0
353.8	0.000198702
353.6	0.000259009
341.7	0
338.6	0
329.5	0.000856829
329.5	0.000471743
326	0
326	0
306.7	0
303.9	0.031167341
303.8	0
303	0.061812832
288.9	0
288.5	0
279.1	0
278.8	0
276.2	2.801202883
274.1	0.101165958
272.4	0.418666565
266.8	0.187372058
263.1	0
262.9	0
262.5	0
260.6	0
256.7	0
256.1	0.465513288
255.3	0.541644726
254.9	0
254	0
253.8	0.060608545
252.8	0
252.7	0
251.7	0.034543077
251.5	0
246.3	0
245.7	0
234.8	0.006809999
234.5	0.009860696

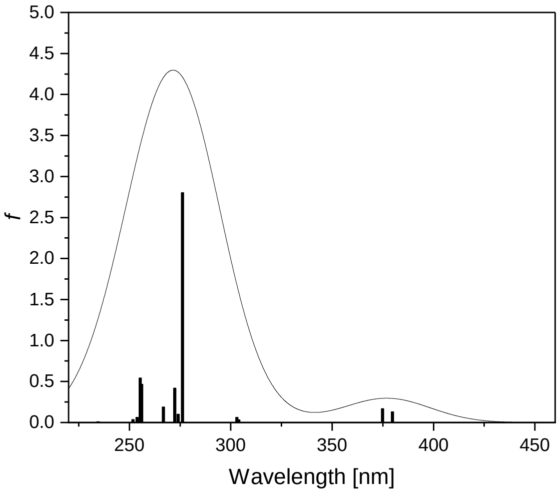

Figure S3.40. Simulated UV-vis spectrum of **1-PCy_3** in DCM (ωB97X-D3/def2-TZVPP/SMD(DCM). Gaussian line broadening with 50 nm FWHM. λ_{max} = 376.9 nm.

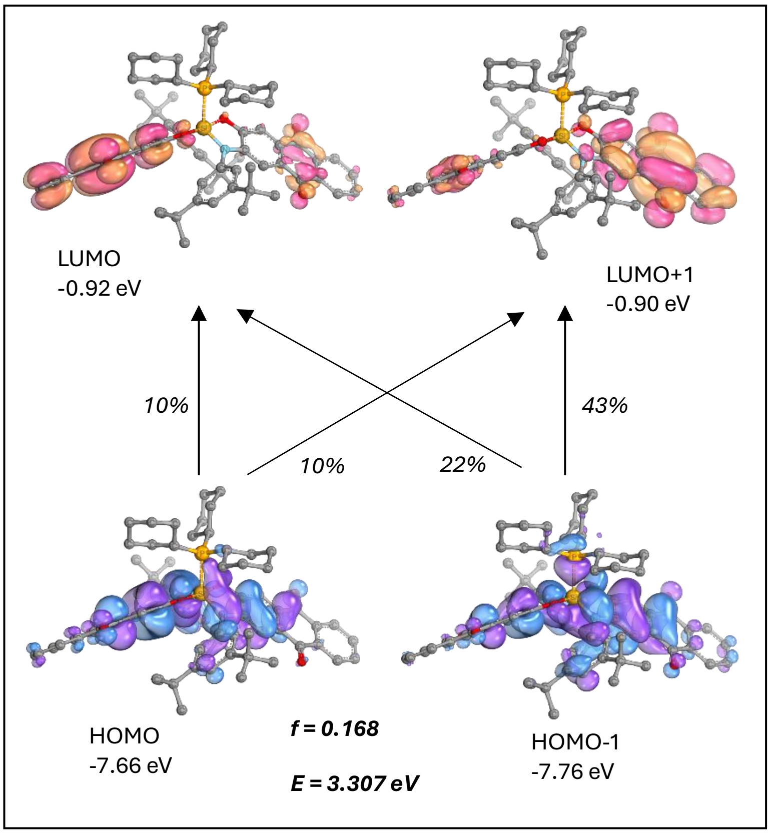

Figure S3.41. Molecular orbitals involved in the low energy CT transition of **1-PCy_3**. HOMO->LUMO 10%, HOMO-1->LUMO+1 43%, HOMO->LUMO+1 10%, HOMO-1->LUMO 22%. Transition at 3.265 eV: HOMO->LUMO 42%, HOMO-1->LUMO+1 4%, HOMO->LUMO+1 28%, HOMO-1->LUMO 12%.

1-DABCO

Table S3.34. Calculated TD-DFT/TDA (*ωB97X-D3/def2-TZVPP/SMD(DCM))* electronic transitions for compound **1-DABCO**.

Wavelength [nm]	Oscillator strength (f)
482.7	0
479.8	0
402.5	0
402.1	0
372.5	0
372.2	0
370	0.077217448
368.1	0.219262257
364.3	0
364.2	0
358.6	0
358.5	0
353.7	0.000428381
353.5	0.000344328
334.5	0
333.4	0
329.6	0.000676977
329.4	0.000677398
326.1	0
325.9	0
302.7	0
302.6	0
300.7	0.065293901
300.3	0.062208209
287.9	0
287.7	0
279.2	0
279	0
274.6	1.147756885
274.1	0.08778132
269.4	1.991034153
262.6	0.173247649
262.1	0
262	0
261.7	0
261.6	0
255.3	0.339116828
255	0.709680142
253.8	0
253.2	0
252.6	0
252.6	0
245.7	0
245.2	0
244.6	0
244.3	0.057167081
243.8	0
243.3	0.064359824
233.7	0.006838201
233.5	0.000735247

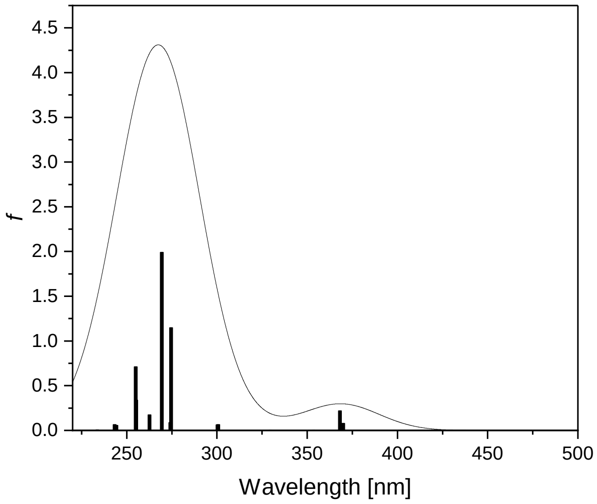

Figure S3.42. Simulated UV-vis spectrum of **1-DABCO** in DCM (ωB97X-D3/def2-TZVPP/SMD(DCM). Gaussian line broadening with 50 nm FWHM. λ_{max} = 368.4 nm.

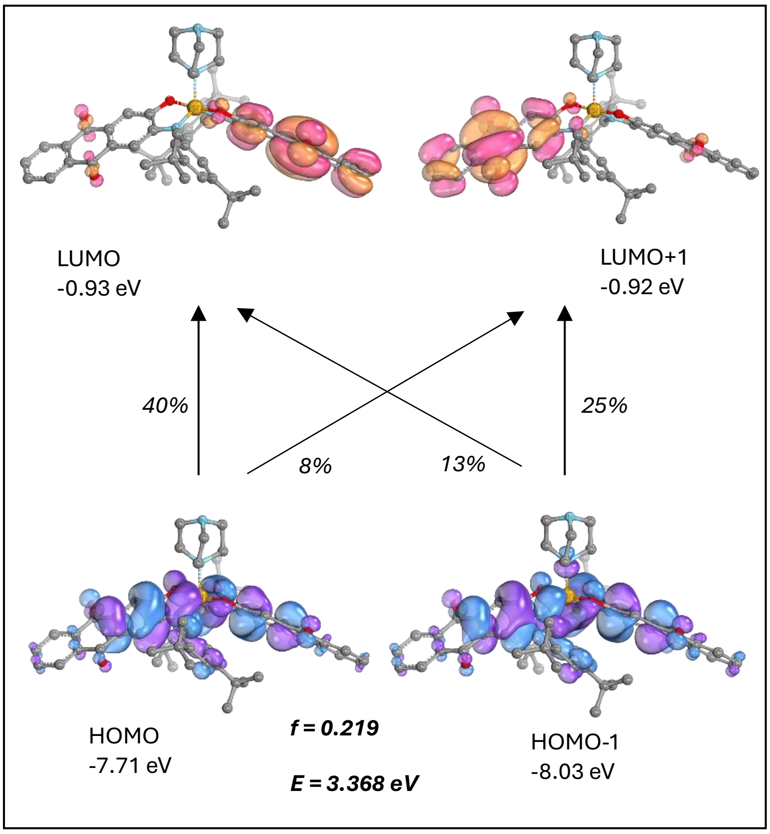

Figure S3.43. Molecular orbitals involved in the low energy CT transition of **1-DABCO**. HOMO->LUMO 40%, HOMO-1->LUMO+1 25%, HOMO->LUMO+1 8%, HOMO-1->LUMO 13%.

1-(pyridine)$_2$

Table S3.35. Calculated TD-DFT/TDA (*ωB97X-D3/def2-TZVPP/SMD(DCM))* electronic transitions for compound **1-(pyridine)$_2$**.

Wavelength [nm]	Oscillator strength (f)
530.7	0
530.5	0
404	0.002020827
402.5	0
402	0
401.9	0.430672072
371.6	0
371.2	0
368.2	0
368.1	0
363.6	0
363.4	0
360.3	0
360.2	0
353.8	7.1084E-5
353.5	7.1703E-5
329.2	7.7739E-5
328.9	9.0128E-5
325.7	0
325.6	0
311.2	0.076418422
310.9	0.000126911
310.3	0
310.2	0
289.5	0
289.4	0
288.7	0
288.5	2.749077853
288.3	0
288.2	0
284.3	0.035436076
283.1	0
280.6	0
279.6	0
278.8	0
278.5	0
276.8	0.071595775
275.5	0.353872657
273	0
272.8	0.002906425
272.6	0.043030254
271	0.002713319
270.2	0
269.8	0
268	0.092059306
267.1	0
265.9	0
263.9	0
262	0
261.2	0.002146404

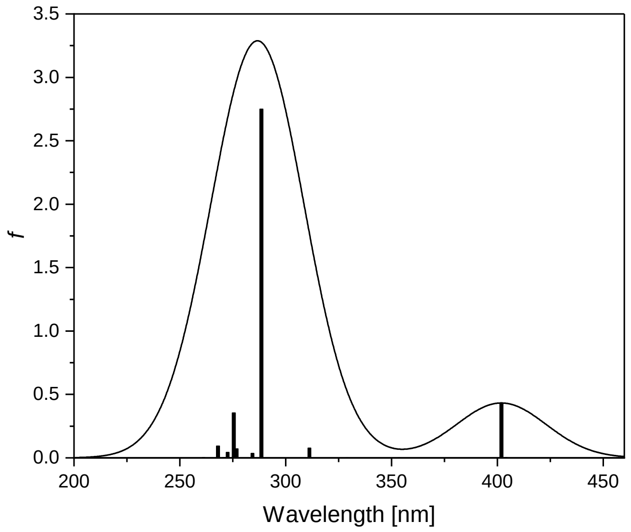

Figure S3.44. Simulated UV-vis spectrum of **1-(pyridine)$_2$** in DCM (ωB97X-D3/def2-TZVPP/SMD(DCM). Gaussian line broadening with 50 nm FWHM.

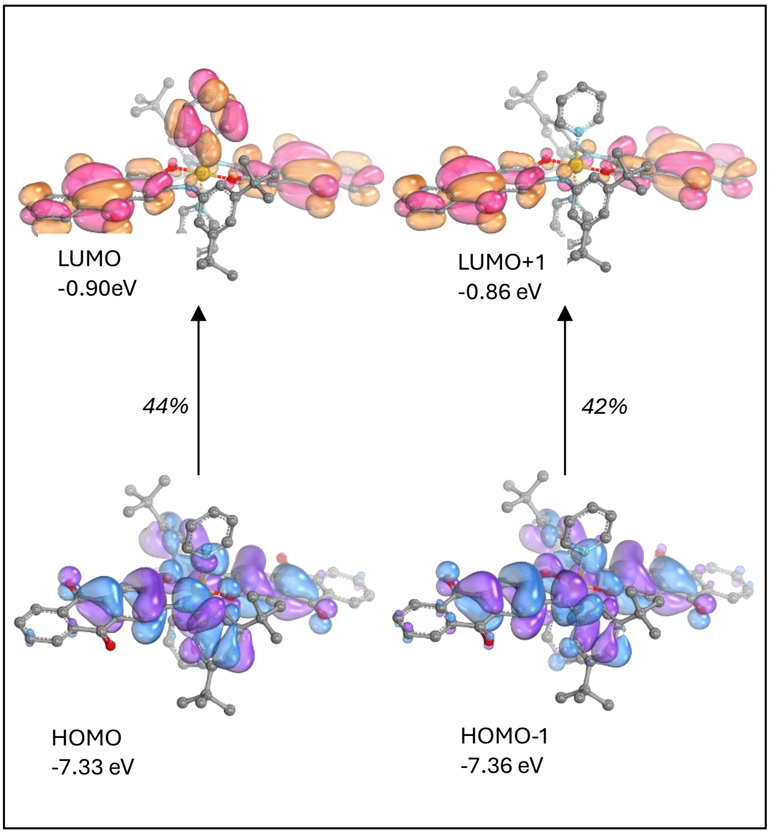

Figure S3.45. Molecular orbitals involved in the low energy CT transition of **1-(pyridine)$_2$**. HOMO->LUMO 44%, HOMO-1->LUMO+1 42%.

3.6 Correlation between TD-DFT/TDA excitations and experimental UV-vis spectra

For the correlation between experimental spectra and calculated transitions, all calculated energies were shifted by -0.56 eV to match λ_{max} of **1**.

Table S3.36. Calculated and experimental absorption energies and maximum absorption wavelengths for **1** and adducts **1-X**.

**Compound**	**E (at λ_{max}, TD-DFT) [eV]** *ωB97X-D3/def2-TZVPP/SMD(DCM)*	**E_{corr} (at λ_{max}, TD-DFT, -0.56 eV) [eV]** *ωB97X-D3/def2-TZVPP/SMD(DCM)*	**$\lambda_{max, corr}$(TD-DFT) [nm]** *ωB97X-D3/def2-TZVPP/SMD(DCM)*	**λ_{max}(exp, DCM) [nm]**	**E (at λ_{max}, exp, DCM) [eV]**
**1**	3.602	3.042	407.6	407	3.05
**[1-F]**	3.114	2.554	485.4	495	2.50
**[1-Cl]**	3.129	2.569	482.6	489	2.54
**[1-N3]**	3.122	2.562	483.9	496	2.50
**[1-NCS]**	3.169	2.609	475.2	484	2.56
**[1-CN]**	3.179	2.619	473.4	481	2.58
**[1-Br]**	3.124	2.564	483.5	479	2.59
**1-pyridine**	3.353	2.793	443.9	452	2.74
**1-DMAP**	3.307	2.747	451.3	466	2.66
**1-OPEt**	3.284	2.724	455.1	469	2.64
**1-HMPA**	3.267	2.707	458.0	474	2.62
**1-DMSO**	3.332	2.772	447.3	460	2.70
**1-DIBA**	3.288	2.728	454.5	467	2.65
**1-^{dipp}NHC**	3.233	2.673	463.8	475	2.61
**1-SIMes**	3.240	2.680	462.6	472	2.63
**1-^{iPr}NHC**	3.254	2.694	460.2	468	2.65
**1-P(nBu)$_3$**	3.300	2.74	452.5	458	2.71
**1-PCy$_3$**	3.290	2.73	454.1	461	2.69
**1-DABCO**	3.363	2.803	442.3	449	2.76

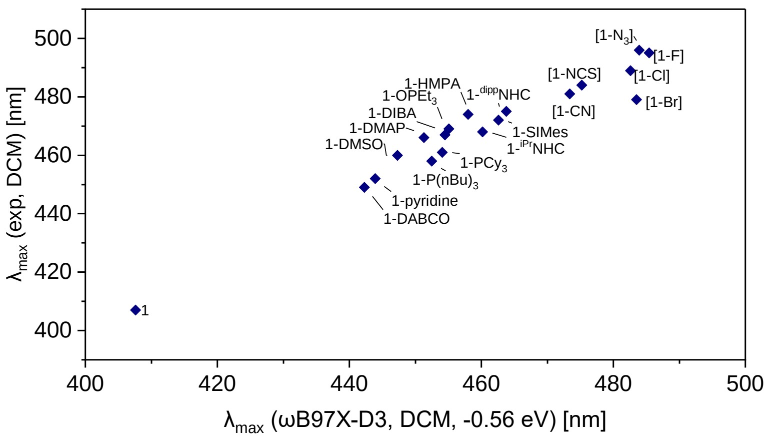

Figure S3.46. Correlation between calculated and experimental absorption spectra. Calculated λ_{max} values were redshifted by 0.56 eV.

3.7 Correlations between UV-vis Data and Calculated Thermodynamics

Correlation between experimental UV-vis absorption energies and computed thermodynamics for 1

All data was fitted with OriginLab 2024b.

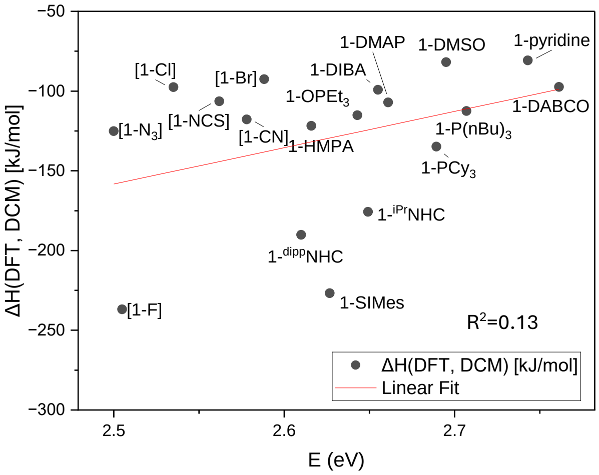

Figure S3.47. Correlation between experimental absorption energies and enthalpy of acid-base association for adducts **1-X** in DCM (DSD-BLYP(D3BJ)/def2-TZVPP/SMD(DCM)//r2scan-3c).

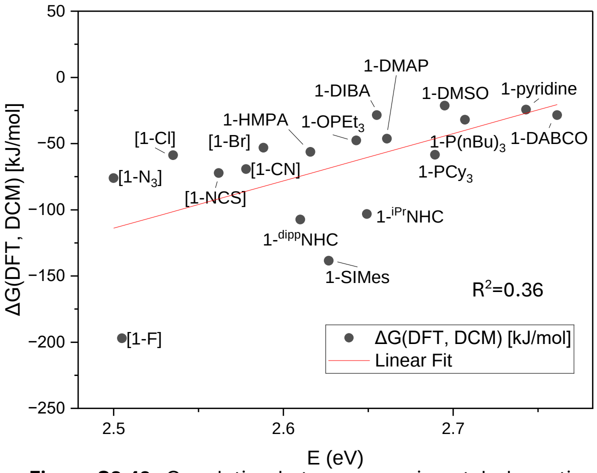

Figure S3.48. Correlation between experimental absorption energies and Gibbs free energies of acid-base association for adducts **1-X** in DCM (DSD-BLYP(D3BJ)/def2-TZVPP/SMD(DCM)//r2scan-3c).

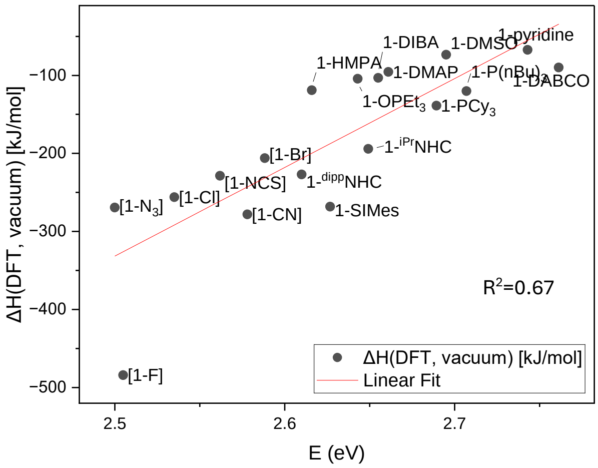

Figure S3.49. Correlation between experimental absorption energies and enthalpy of acid-base association for adducts **1-X** in vacuum (DSD-BLYP(D3BJ)/def2-TZVPP//r2scan-3c).

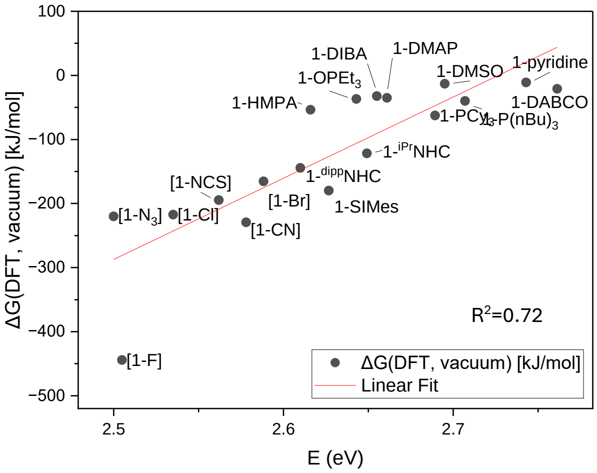

Figure S3.50. Correlation between experimental absorption energies and Gibbs free energies of acid-base association for adducts **1-X** in vacuum (DSD-BLYP(D3BJ)/def2-TZVPP//r2scan-3c).

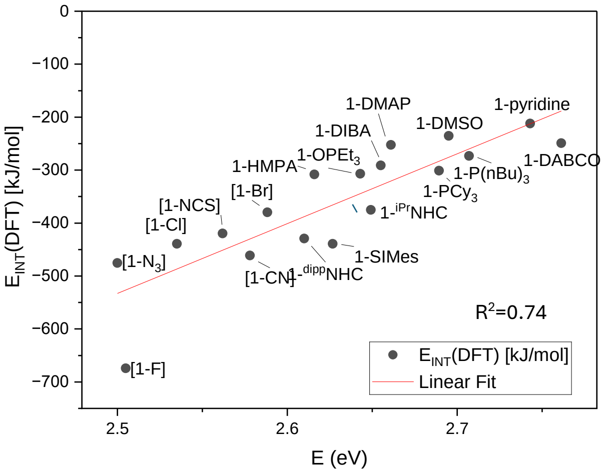

Figure S3.51. Correlation between experimental absorption energies and interaction energies between acid and base fragments adducts **1-X** (DSD-BLYP(D3BJ)/def2-TZVPP).

To compare the obtained data with alternative methods, COSMO-RS solvation correction and anions with explicit counterions were calculated on a subset of the bases.

SMD vs COSMO-RS solvation model

The COSMO-RS solvation model was used to obtain solvent corrected affinities (ΔH/ΔG). Correlations with experimental absorption energies are similarly weak as the SMD model.

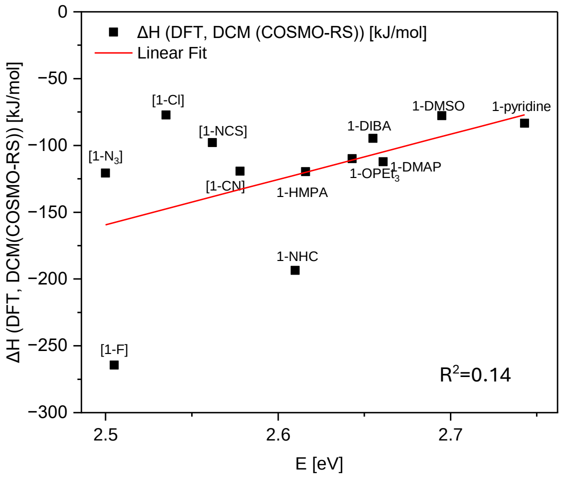

Figure S3.52. Correlation between experimental absorption energies and enthalpy of acid-base association for adducts **1-X** in DCM (DSD-BLYP(D3BJ)/def2-TZVPP/COSMO-RS(DCM)//r2scan-3c).

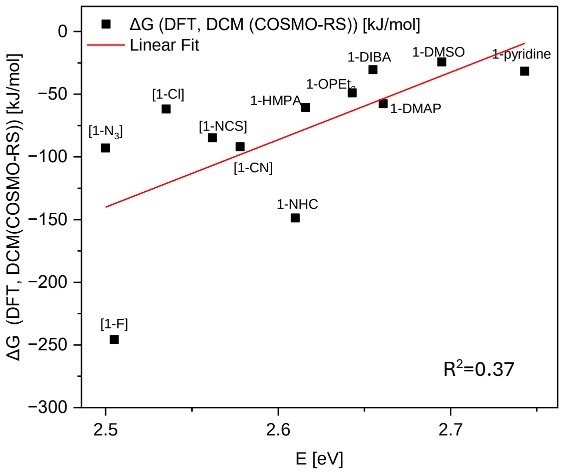

Figure S3.53. Correlation between experimental absorption energies and Gibbs free energies of acid-base association for adducts **1-X** in DCM (DSD-BLYP(D3BJ)/def2-TZVPP/COSMO-RS(DCM))//r2scan-3c).

Explicit counterions

For calculations with explicit counterions, only the correlation of absorption energies with calculated reaction enthalpies is given due to entropic errors resulting from small imaginary frequencies.

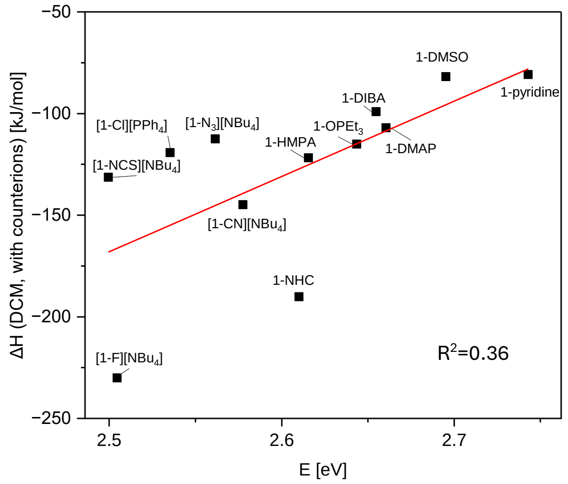

Figure S3.54. Correlation between experimental absorption energies and enthalpy of acid-base association for adducts **[1-X][Cation]** or **1-X** for neutral donors in DCM (DSD-BLYP(D3BJ)/def2-TZVPP/SMD(DCM)//r2scan-3c).

Correlation between ΔΔH (difference between first and second binding enthalpy) and eLB/gLB

The binding of a Lewis base results in a deactivation of the Lewis acidic silicon centre towards a second binding event. The extent of this deactivation is a function of the effect of a given Lewis base (effective Lewis basicity, eLB) and can be estimated by the difference in enthalpy between the two binding events. For example, the highest deactivation is observed for fluoride, where the second coordination is less favourable than the first by 170 kJ/mol.

To assess whether this deactivation is a function of effective Lewis basicity as proposed in this work, the deactivation was correlated with the absorption energy of the corresponding silicon complexes (eLB) and the enthalpy of the first base coordination (gLB). A much stronger correlation is observed for eLB (R^2 = 0.88, figure 3.43) than for gLB (R^2 = 0.49, figure 3.44).

It is important to note that steric effects play a much larger role in the second binding event. This is evidenced by failed convergence or dissociation during geometry optimisations for several of the bulkier Lewis base adducts. Therefore, the discussion is restricted to sterically undemanding bases.

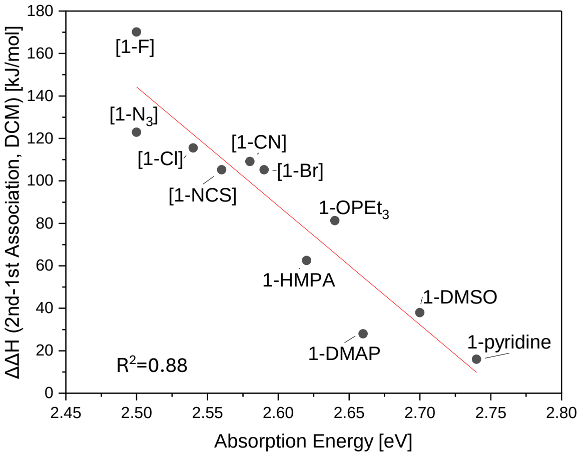

Figure S3.55. Correlation between experimental absorption energies (eLB) and ΔΔH in DCM (difference between first and second binding enthalpy, DSD-BLYP(D3BJ)/def2-TZVPP/SMD(DCM)//r2scan-3c).

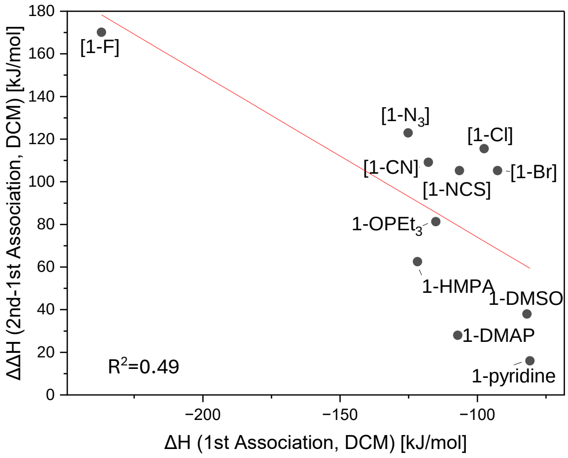

Figure S3.56. Correlation between the first binding enthalpy and ΔΔH in DCM (difference between first and second binding enthalpy, DSD-BLYP(D3BJ)/def2-TZVPP/SMD(DCM)//r2scan-3c).

3.8 Impact of Dipole Moments on Solvation Effects in Adducts 1-X

For solvation processes of free bases and the formed adducts in DCM, significant differences between neutral and anionic bases arise from charges and the emerging dipole moments in the formed adducts. For anionic adducts, dipole moments are oriented in the direction donor→Si. Local charge density and the solvent accessible surface area is reduced compared to the fragments. Consequently, the separated fragments receive higher ΔHSolv than the adduct (Table S3.30, entries 1-5). In contrast, dipole moments are oriented in the direction Si→donor for neutral adducts, with an increase in dipole moment. This leads to a pronounced solvent stabilisation for the formed adducts compared to the separated fragments for small bases (Table S3.30, entries 6,7,8,10). Similar diverging effects of neutral and charged species have been elucidated for neutral amine-borane adducts and charged transition metal complexes.[21, 22] This effect is only relevant for small bases. For large bases (e.g. NHC, DIBA), the fragments receive higher solvent stabilisation despite the increasing dipole moment, presumably because of a large reduction in surface area in the formed adduct.

For anionic bases, the solvation enthalpy of the adducts 1-X are almost identical (mean deviation 5 kJ/mol), suggesting that desolvation of the individual bases is the pivotal effect responsible for observed differences (mean deviation for ΔH_{Solv} of anionic bases 35 kJ/mol). Similarly, for neutral bases, the variance in ΔH_{Solv} is larger for individual bases (16 kJ/mol) than for the adducts (5 kJ/mol). Overall, the variance in solvation energies for bases is 90 kJ/mol and 22 kJ/mol for the adducts.

Table S3.37. Calculated dipole moments of complexes **1-X** (DSD-BLYP/def2-TZVPP//r2scan-3c), ΔH_{Solv} of adducts (**1-X**), and ΔH_{Solv} of the separated acid and base fragments (**1** + **X**). (SMD(DCM)).

Entry	Compound	Dipole Moment (1-X) [D]	ΔH_{Solv} (X) [kJ/mol]	$\sum\Delta H_{Solv}$ (1 + X) [kJ/mol]	ΔH_{Solv} (1-X) [kJ/mol]	$\Delta\Delta H_{Solv}$ [kJ/mol]
**1**	1-F	6.24622	-347.87	-506.54	-259.35	247.19
**2**	1-Cl	6.68931	-261.28	-419.95	-261.46	158.49
**3**	1-Br	6.22181	-211.54	-370.21	-256.75	113.46
**4**	1-N_3	7.12006	-229.22	-387.89	-243.81	144.08
**5**	1-NCS	8.47473	-214.39	-373.06	-250.77	122.29
**6**	1-CN	9.59049	-257.32	-415.99	-255.72	160.27
**7**	1-pyridine	7.27340	-29.60	-188.27	-201.81	-13.54
**8**	1-DMAP	11.95456	-43.50	-202.17	-213.55	-11.38
**9**	1-$OPEt_3$	10.58696	-43.61	-202.28	-213.04	-10.76
**10**	1-HMPA	10.64565	-39.50	-198.17	-200.90	-2.73
**11**	1-DMSO	8.15066	-32.71	-191.38	-199.64	-8.26
**12**	1-DIBA	9.73996	-53.94	-212.61	-208.58	4.03
**13**	1-^{dipp}NHC	8.84686	-90.20	-248.87	-211.89	36.98
**14**	1-SIMes	6.96345	-92.09	-250.76	-209.34	41.42
**15**	1-^{iPr}NHC	8.72549	-69.56	-228.23	-203.65	24.58
**16**	1-$P(nBu)_3$	5.24454	-38.91	-197.58	-189.86	7.72
**17**	1-PCy_3	6.88354	-52.64	-211.31	-207.37	3.94
**18**	1-DABCO	5.07830	-37.08	-195.75	-203.11	-7.36

3.9 Comparison of Absorption Energy and pK_A Values

Not for all bases pK_A values were available. No general correlation was found between the available pK_A values and the absorption energy of the corresponding adducts **1-X**. However, the order of the base strength within structurally similar bases is maintained. Similar observations were made for gLB (ΔH in DCM for the formation of **1-X**), which differs from pK_A (gLB towards H^+ in H_2O) due to the change in both reference Lewis acid (**1** vs. H^+) and solvation contributions (DCM vs. H_2O).

F^- > Cl^- > Br^-; N_3^- > NCS^-; HMPA > DMSO; DMAP > pyridine; PCy_3 > PBu_3

Table S3.38. Absorption energy of complexes **1-X** and available pK_A values (**HX**) in H_2O.

Base	Absorption energy (1-X) [eV]	pK_A (conjugated acid HX, H_2O)
[F]	2.505	3.2[23]
[Cl]	2.535	-7.3[23]
[Br]	2.588	-9.8[23]
[N_3]	2.500	4.65[24]
[NCS]	2.562	-1.28[25]
[CN]	2.578	9.36[26]
pyridine	2.743	5.2[27]
DMAP	2.661	9.87[28]
$OPEt_3$	2.643	-
HMPA	2.616	-0.97[29]
DMSO	2.695	-1.54[29]
DIBA	2.655	-
^{dipp}NHC	2.610	-
SIMes	2.627	-
^{iPr}NHC	2.649	-
P(nBu)$_3$	2.707	8.4[30]
PCy_3	2.689	9.7[30]
DABCO	2.761	8.7[31]

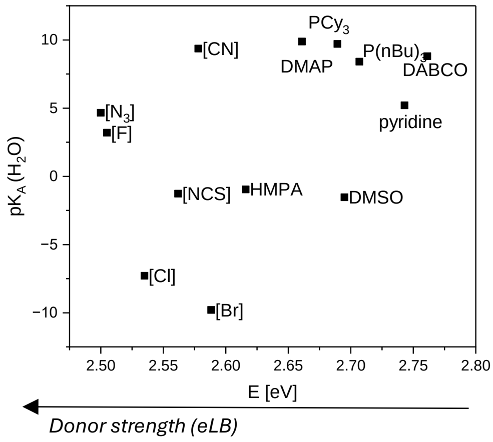

Figure S3.57. Experimental absorption energies (eLB) and available pK_A values.

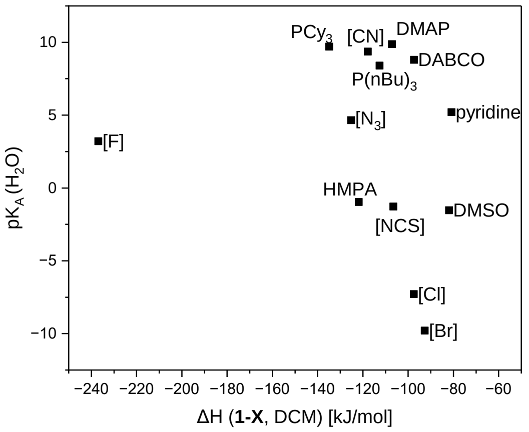

Figure S3.58. Computed enthalpy of adduct formation in DCM (gLB) and available pK_A values.

4 X-Ray Crystallography

Crystals were immersed in perfluorinated polyether oil and fixed on a cryo-loop. Data were collected from a shock-cooled single crystal at 100(2) K on a Bruker D8 VENTURE dual wavelength Mo/Cu four-circle diffractometer with a microfocus sealed X-ray tube using a mirror optics as monochromator and a Bruker PHOTON III detector. The diffractometer was equipped with an Oxford Cryostream 700 low temperature device and used MoK_α radiation (λ = 0.71073 Å). All data were integrated with SAINT and a multi-scan absorption correction using SADABS was applied.[32, 33] The structure was solved by direct methods using SHELXT and refined by full-matrix least-squares methods against F^2 by SHELXL-2019/2.[34, 35] All non-hydrogen atoms were refined with anisotropic displacement parameters. All hydrogen atoms were refined isotropic on calculated positions using a riding model with their U_{iso} values constrained to 1.5 times the U_{eq} of their pivot atoms for terminal sp^3 carbon atoms and 1.2 times for all other carbon atoms. Crystallographic data for the structures reported here have been deposited with the Cambridge Crystallographic Data Centre.[36] CCDC 2403101-2403102 contain the supplementary crystallographic data for this paper. These data can be obtained free of charge from The Cambridge Crystallographic Data Centre via www.ccdc.cam.ac.uk/structures. This report and the CIF file were generated using FinalCif.[37]

1

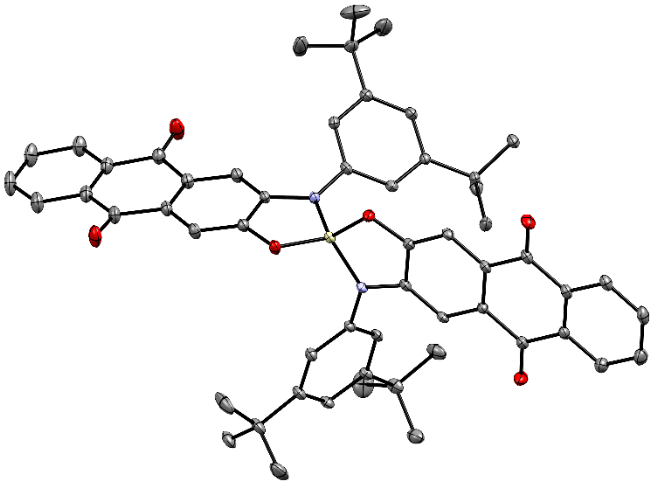

Figure S4.1. Solid-state structure of **1**, crystallised from DCM/*n*-pentane at room temperature. Thermal ellipsoids are displayed at the 50% probability level. Hydrogen atoms and cocrystallised DCM molecules are omitted for clarity.

**CCDC number**	2403101
**Empirical formula**	$C_{56}H_{54}Cl_0N_2O_6Si$
**Formula weight**	879.10
**Temperature [K]**	100(2)
**Crystal system**	triclinic
**Space group (number)**	$P\bar{1}$ (2)
***a* [Å]**	8.954(3)
***b* [Å]**	14.794(6)
***c* [Å]**	20.837(8)
**α [°]**	106.67(2)
**β [°]**	93.268(11)
**γ [°]**	90.445(12)
**Volume [Å^3]**	2639.0(17)
Z	2
**ρ_{calc} [gcm^{-3}]**	1.106
**μ [mm^{-1}]**	0.093
***F*(000)**	932
**Crystal size [mm^3]**	0.885×0.214×0.182
**Crystal colour**	yellow
**Crystal shape**	block
**Radiation**	MoK_α (λ=0.71073 Å)
**2θ range [°]**	3.98 to 58.49 (0.73 Å)
**Index ranges**	−12 ≤ h ≤ 12<br>−20 ≤ k ≤ 20<br>−28 ≤ l ≤ 28
**Reflections collected**	202726
**Independent reflections**	14321<br>R_{int} = 0.0695<br>R_{sigma} = 0.0292
**Completeness to θ = 25.242°**	99.9 %
**Data / Restraints / Parameters**	14321/0/601
**Absorption correction T_{min}/T_{max} (method)**	0.6714/0.7458<br>(multi-scan)
**Goodness-of-fit on F^2**	1.074
**Final *R* indexes [$I \geq 2\sigma(I)$]**	R_1 = 0.0616<br>wR_2 = 0.1580
**Final *R* indexes [all data]**	R_1 = 0.0693<br>wR_2 = 0.1631
**Largest peak/hole [eÅ^{-3}]**	0.73/−0.48

[1-Cl][PPh$_4$]

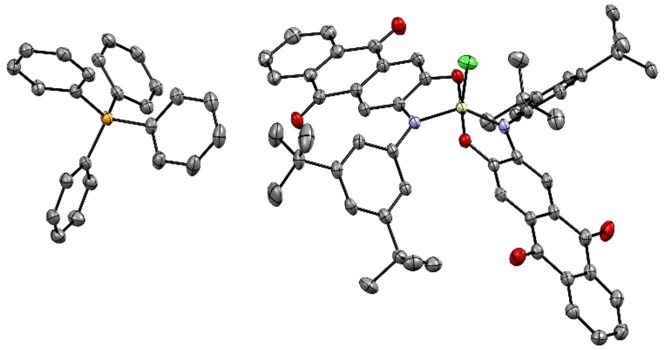

Figure S4.2. Solid-state structure of **[1-Cl][PPh$_4$]**, crystallised from DCM/*n*-pentane at -40 °C. Thermal ellipsoids are displayed at the 50% probability level. Hydrogen atoms and cocrystallised DCM molecules are omitted for clarity.

**CCDC number**	2403102
**Empirical formula**	$C_{83.92}H_{81.84}Cl_{8.84}N_2O_6PSi$
**Formula weight**	1586.81
**Temperature [K]**	100(2)
**Crystal system**	monoclinic
**Space group (number)**	$P2_1/c$ (14)
***a* [Å]**	23.7594(15)
***b* [Å]**	12.8395(8)
***c* [Å]**	27.1540(16)
**α [°]**	90
**β [°]**	101.651(2)
**γ [°]**	90
**Volume [Å^3]**	8112.9(9)
Z	4
**ρ_{calc} [gcm^{-3}]**	1.299
**μ [mm^{-1}]**	0.393
***F*(000)**	3307
**Crystal size [mm^3]**	0.22×0.10×0.05
**Crystal colour**	red
**Crystal shape**	plate
**Radiation**	MoK_α (λ=0.71073 Å)
**2θ range [°]**	3.79 to 52.94 (0.80 Å)
**Index ranges**	−29 ≤ h ≤ 29<br>−16 ≤ k ≤ 16<br>−34 ≤ l ≤ 33
**Reflections collected**	511533
**Independent reflections**	16709<br>R_{int} = 0.0981<br>R_{sigma} = 0.0259
**Completeness to θ = 25.242°**	100.0 %
**Data / Restraints / Parameters**	16709/531/1034
**Absorption correction T_{min}/T_{max} (method)**	0.6796/0.7454 (multi-scan)
**Goodness-of-fit on *F*^{2}**	1.037
**Final *R* indexes [*I*≥2σ(*I*)]**	R_1 = 0.0507<br>wR_2 = 0.1242
**Final *R* indexes [all data]**	R_1 = 0.0672<br>wR_2 = 0.1357
**Largest peak/hole [eÅ^{-3}]**	0.71/−0.78

1-PCy_3

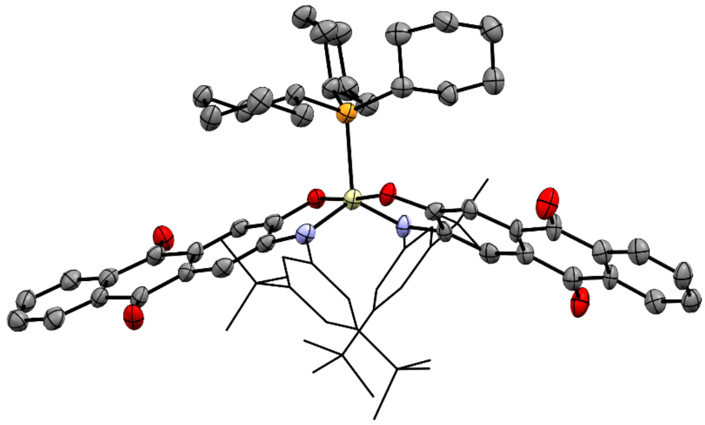

Figure S4.3. Solid-state structure of **1-PCy_3**, crystallised from DCM/*n*-pentane at -40 °C. Thermal ellipsoids are displayed at the 50% probability level. Hydrogen atoms and cocrystallised DCM and *n*-pentane molecules are omitted for clarity. Nitrogen substituents displayed as wireframe for clarity.

**CCDC number**	2466565
**Empirical formula**	$C_{84.50}H_{111}Cl_6N_2O_6PSi$
**Formula weight**	1522.51
**Temperature [K]**	100(2)
**Crystal system**	triclinic
**Space group (number)**	$P\bar{1}$ (2)
***a* [Å]**	15.0843(17)
***b* [Å]**	15.9059(17)
**c [Å]**	20.643(2)
**α [°]**	108.857(4)
**β [°]**	95.289(4)
**γ [°]**	114.939(3)
**Volume [Å^3]**	4096.6(8)
Z	2
**ρ_{calc} [gcm^{-3}]**	1.234
**μ [mm^{-1}]**	0.296
***F*(000)**	1622
**Crystal size [mm^3]**	0.12×0.13×0.27
**Crystal colour**	red
**Crystal shape**	block
**Radiation**	MoK_α (λ=0.71073 Å)
**2θ range [°]**	4.05 to 50.05 (0.84 Å)
**Index ranges**	$-17 \le h \le 17$<br>$-18 \le k \le 17$<br>$0 \le l \le 24$
**Reflections collected**	14310
**Independent reflections**	14310<br>R_{int} = 0.0923<br>R_{sigma} = 0.1327
**Completeness to θ = 25.027°**	99.0 %
**Data / Restraints / Parameters**	14310 / 460 / 1049
**Absorption correction T_{min}/T_{max} (method)**	0.5968 / 0.7453 (multi-scan)
**Goodness-of-fit on F^2**	1.038
**Final *R* indexes [$I \geq 2\sigma(I)$]**	R_1 = 0.1148<br>wR_2 = 0.2872
**Final *R* indexes [all data]**	R_1 = 0.1813<br>wR_2 = 0.3177
**Largest peak/hole [eÅ^{-3}]**	0.61/−0.54

Unrefined structures of 1-^{dipp}NHC, [1-NCS][NBu$_4$] and 1-HMPA

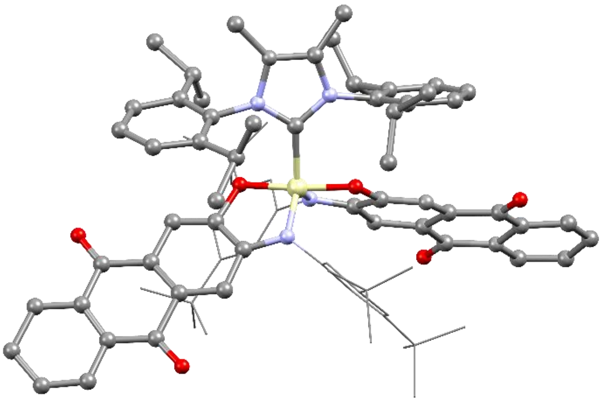

Figure S4.4. Unrefined solid-state structure of **1-^{dipp}NHC**, crystallised from DCM at -40 °C.

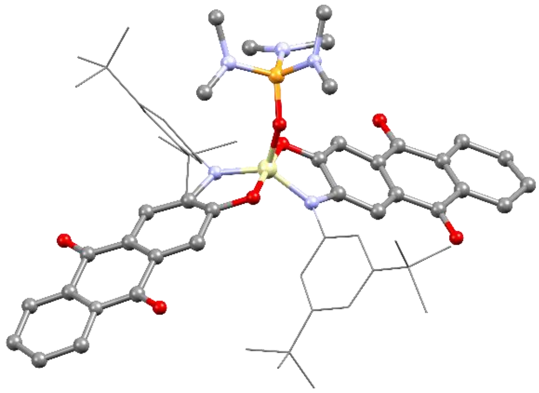

Figure S4.5. Unrefined solid-state structure of **1-HMPA**, crystallised from THF/*n*-pentane at -40 °C. Cocrystallised THF molecules omitted for clarity.

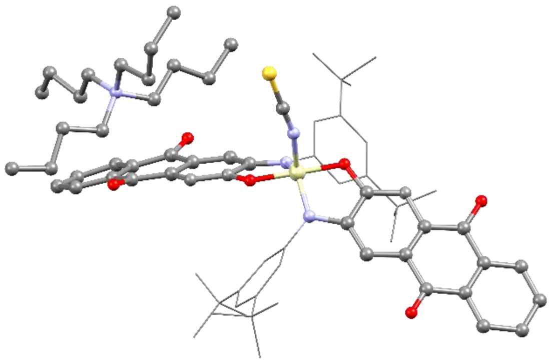

Figure S4.6. Unrefined solid-state structure of [**1-NCS**][**NBu$_4$**], crystallised from 1,2-difluorobenzene/*n*-pentane at -40 °C. Cocrystallised oDFB molecules omitted for clarity.

5 IR Spectra of Isolated Compounds

ATR-IR spectra were measured on an *Agilent Cary 630* spectrometer inside a nitrogen-filled glovebox and subsequently plotted and analysed with OriginLab 2024.

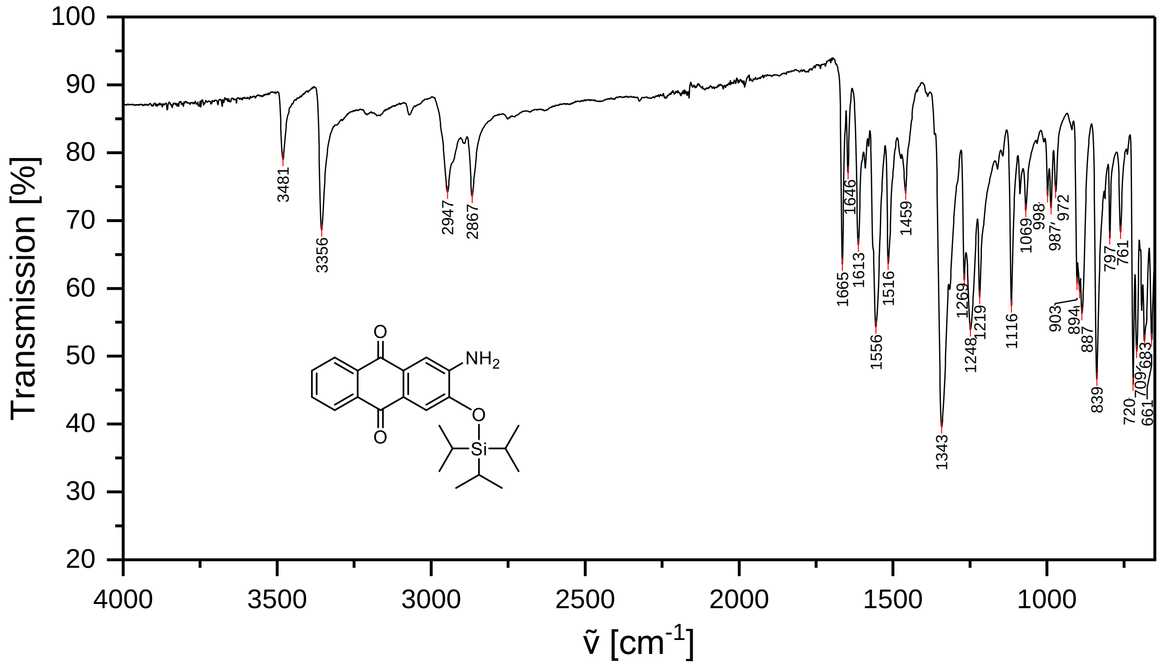

Figure S5.1. ATR-IR spectrum of 2-amino-3-(triisopropylsilyloxy)anthraquinone.

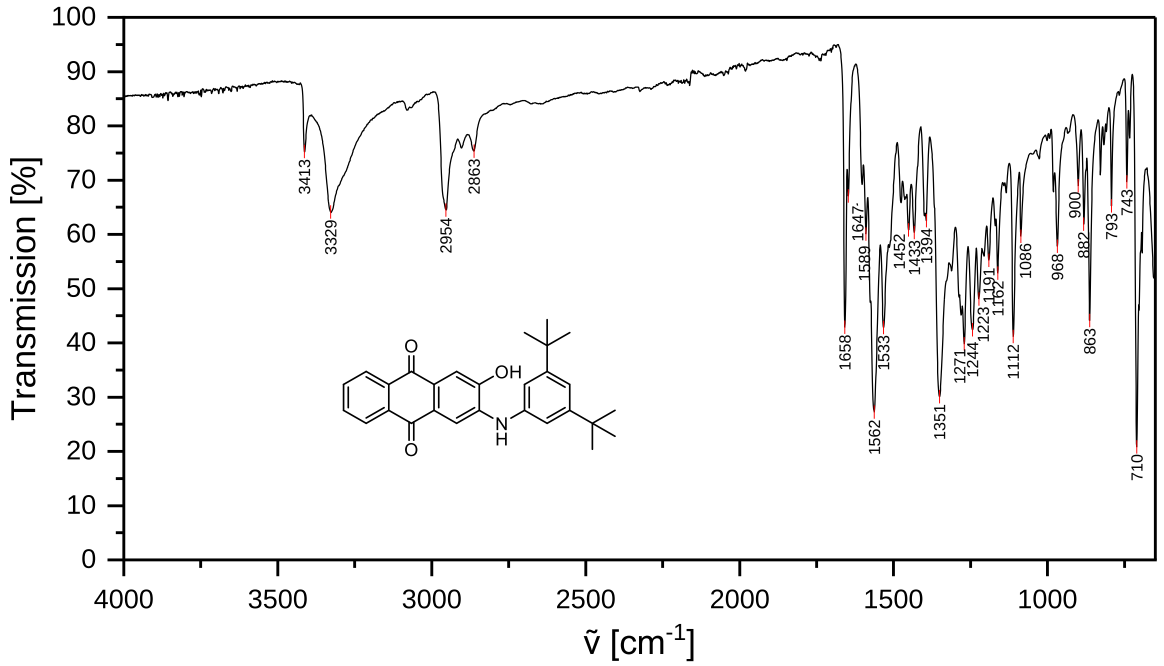

Figure S5.2. ATR-IR spectrum of L_1.

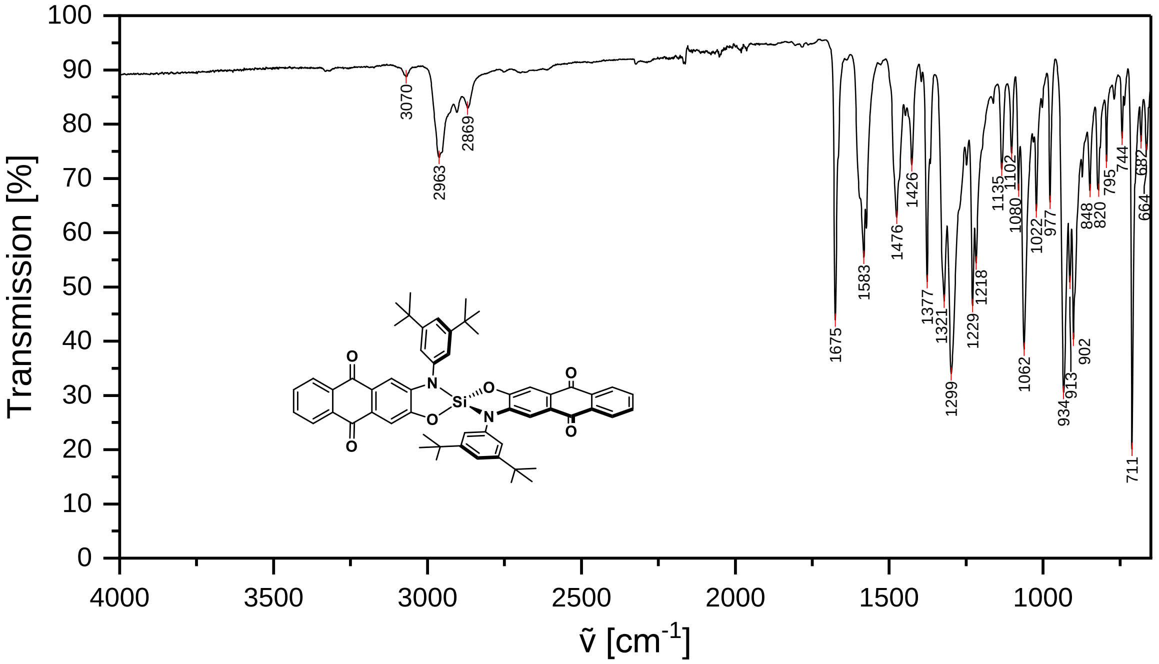

Figure S5.3. ATR-IR spectrum of **1**.

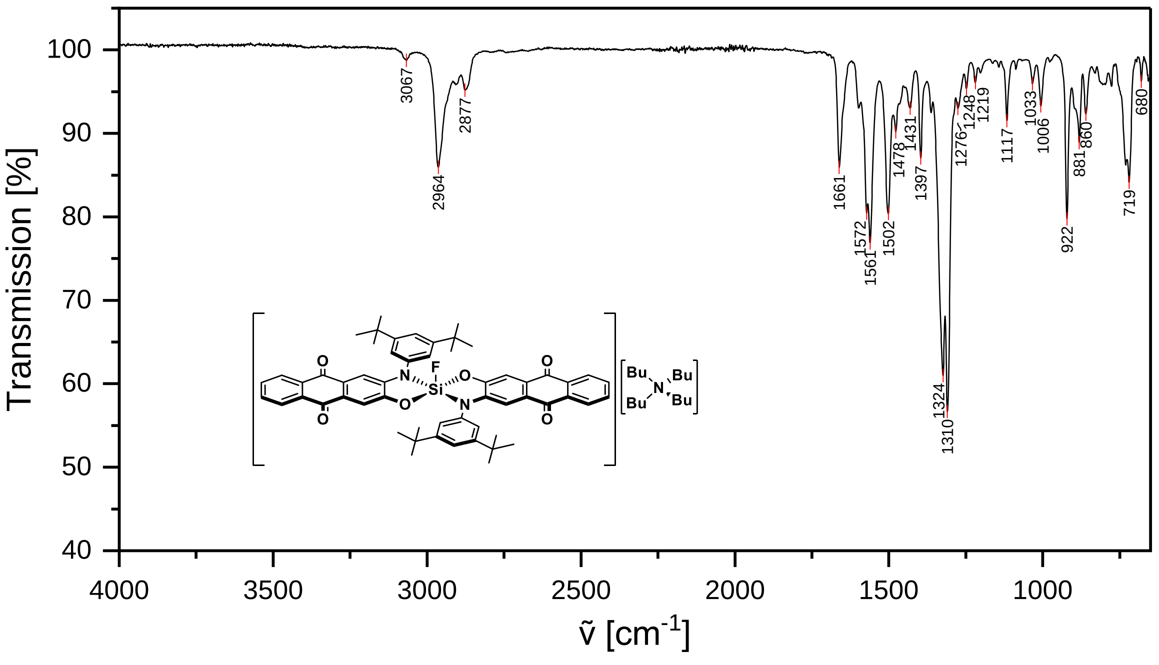

Figure S5.4. ATR-IR spectrum of **[1-F][NBu$_4$]**.

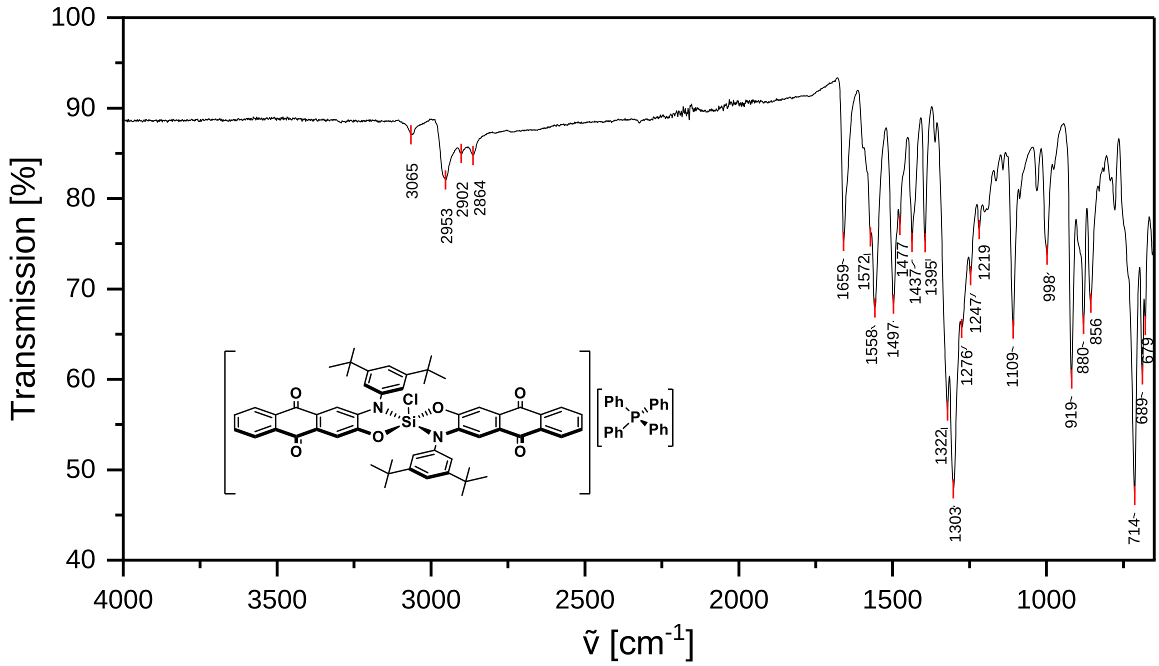

Figure S5.5. ATR-IR spectrum of **[1-Cl][PPh₄]**.

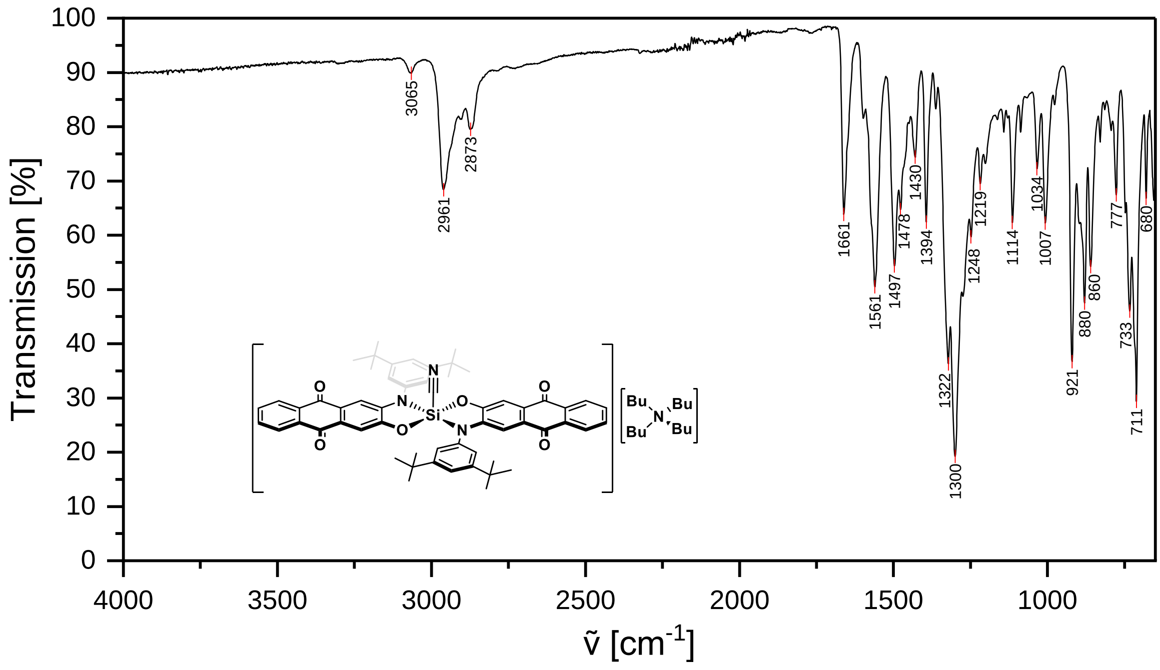

Figure S5.6. ATR-IR spectrum of **[1-CN][NBu₄]**.

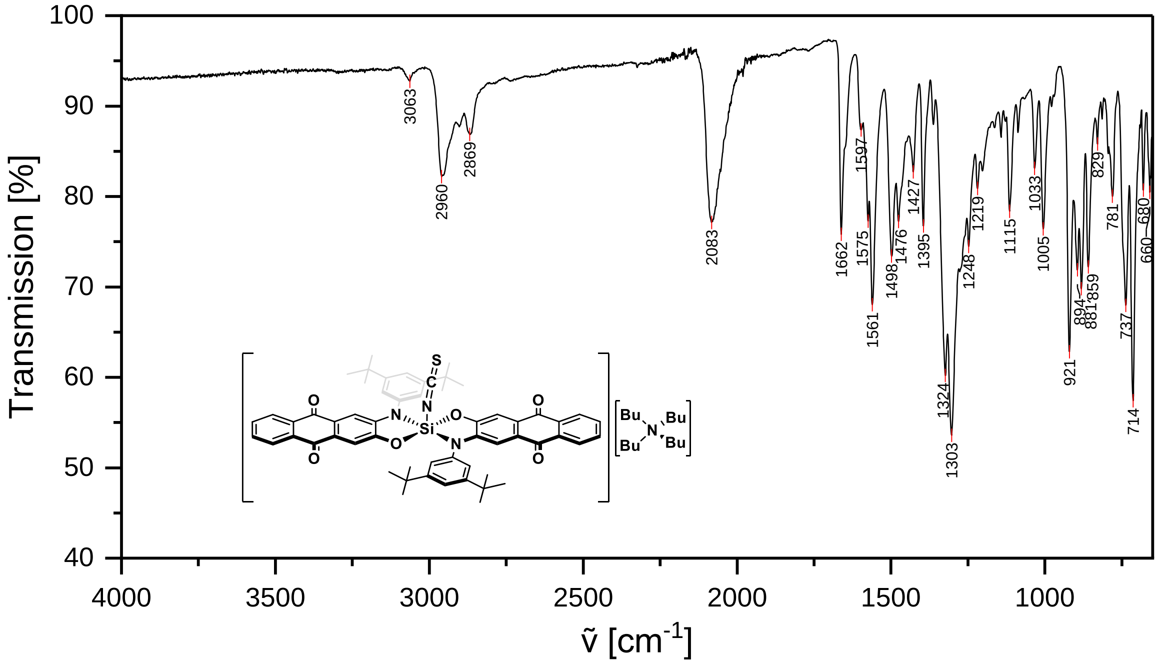

Figure S5.7. ATR-IR spectrum of **[1-NCS][NBu4]**.

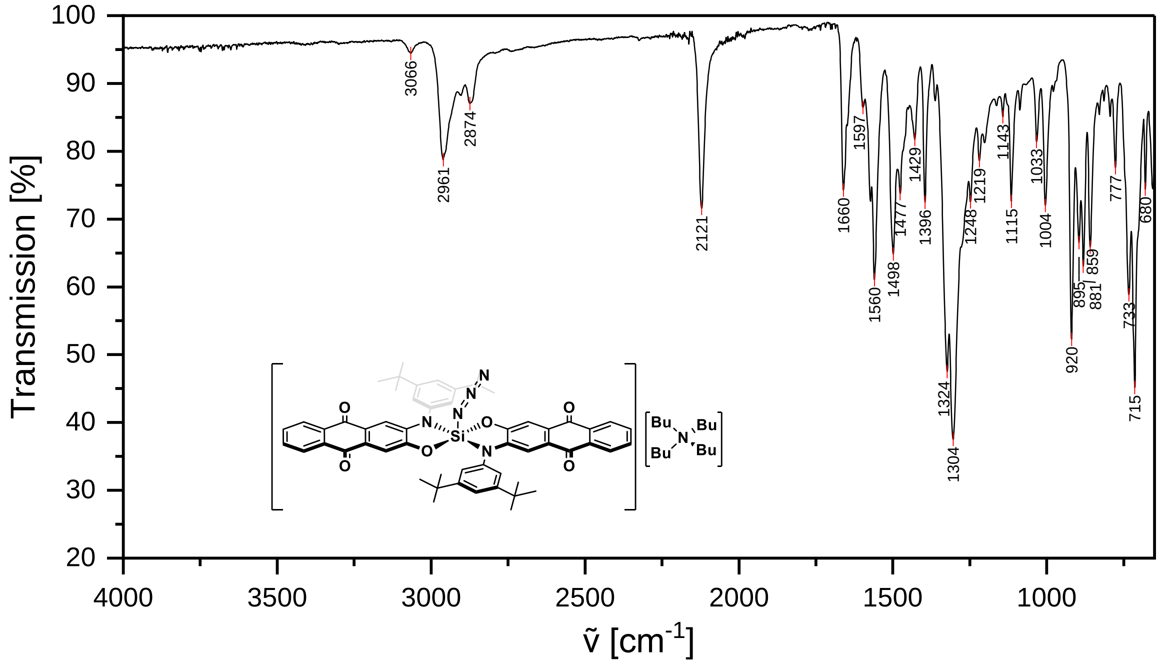

Figure S5.8. ATR-IR spectrum of **[1-N_3][NBu4]**.

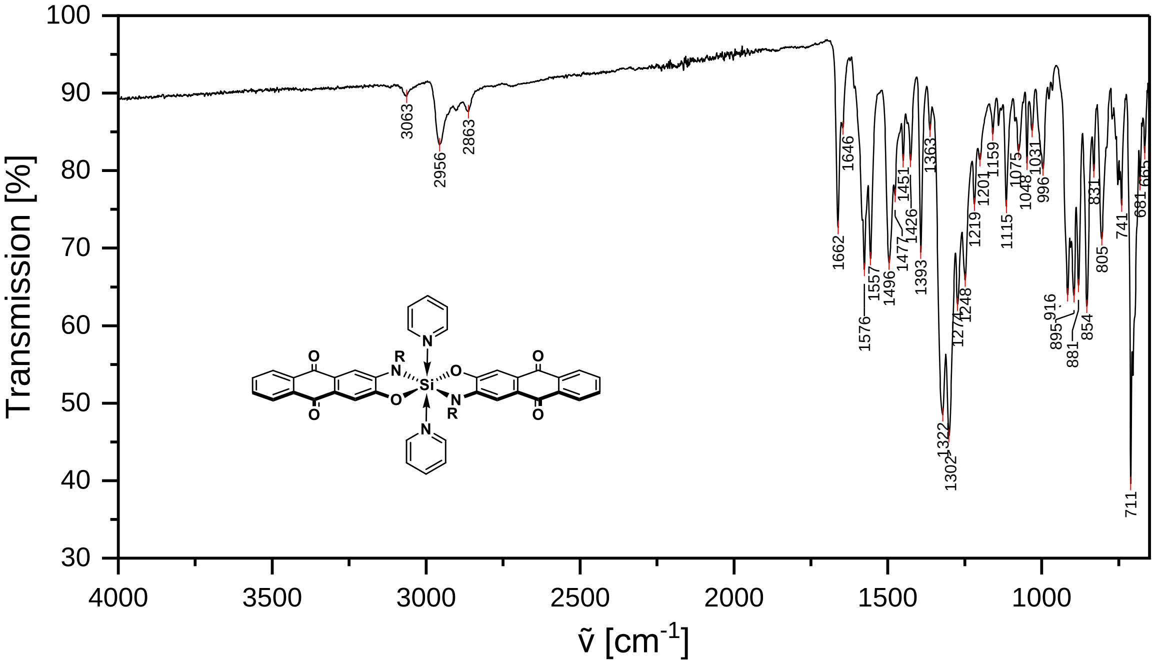

Figure S5.9. ATR-IR spectrum of **1-(pyridine)₂**.

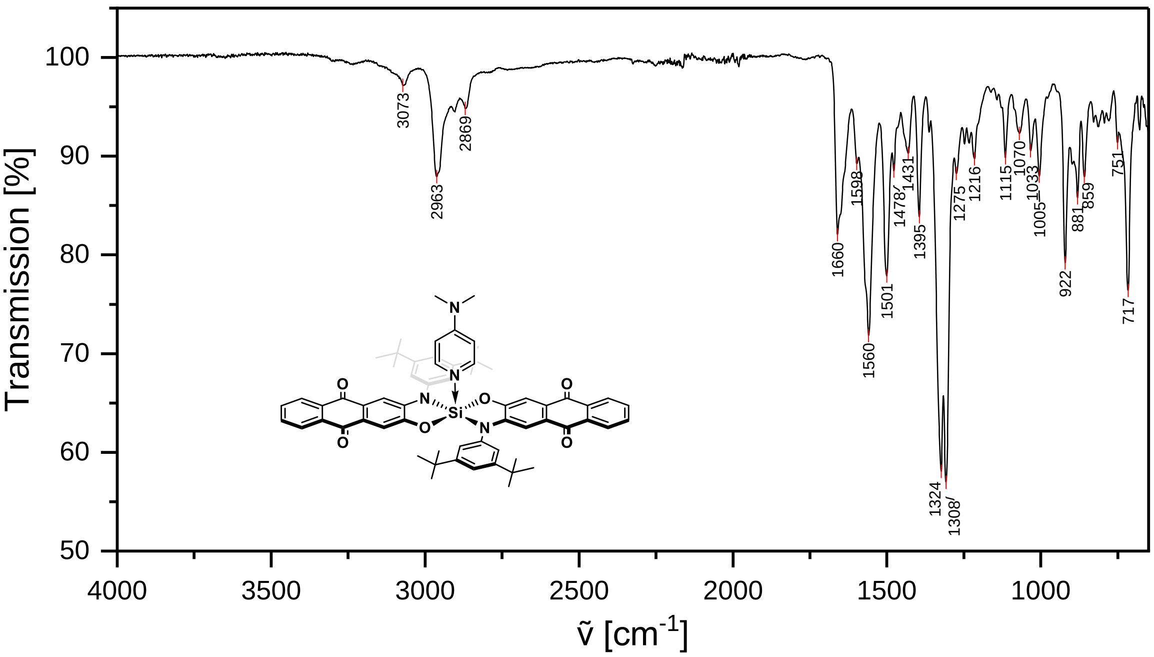

Figure S5.10. ATR-IR spectrum of **1-DMAP**.

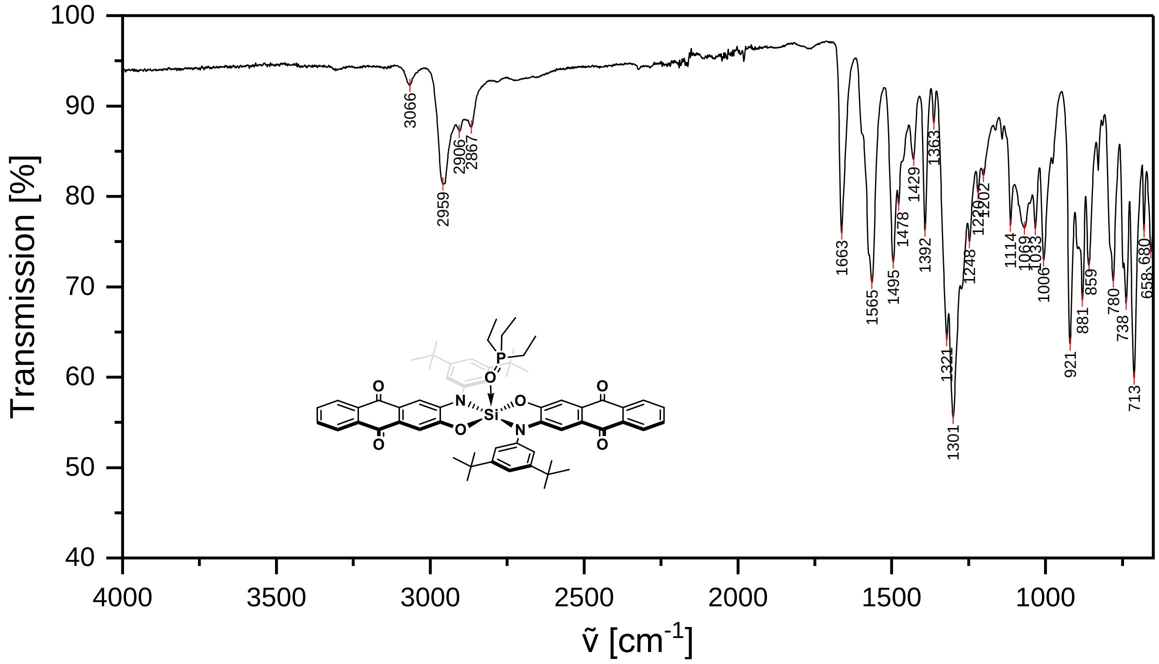

Figure S5.11. ATR-IR spectrum of **1-OPEt$_3$**.

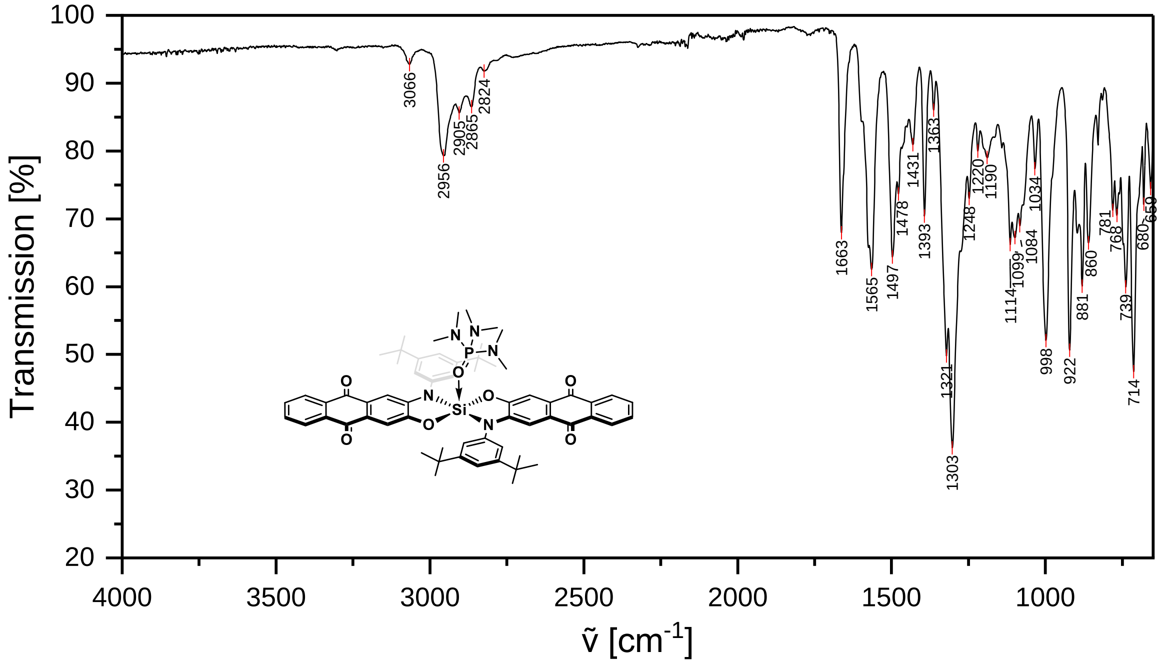

Figure S5.12. ATR-IR spectrum of **1-HMPA**.

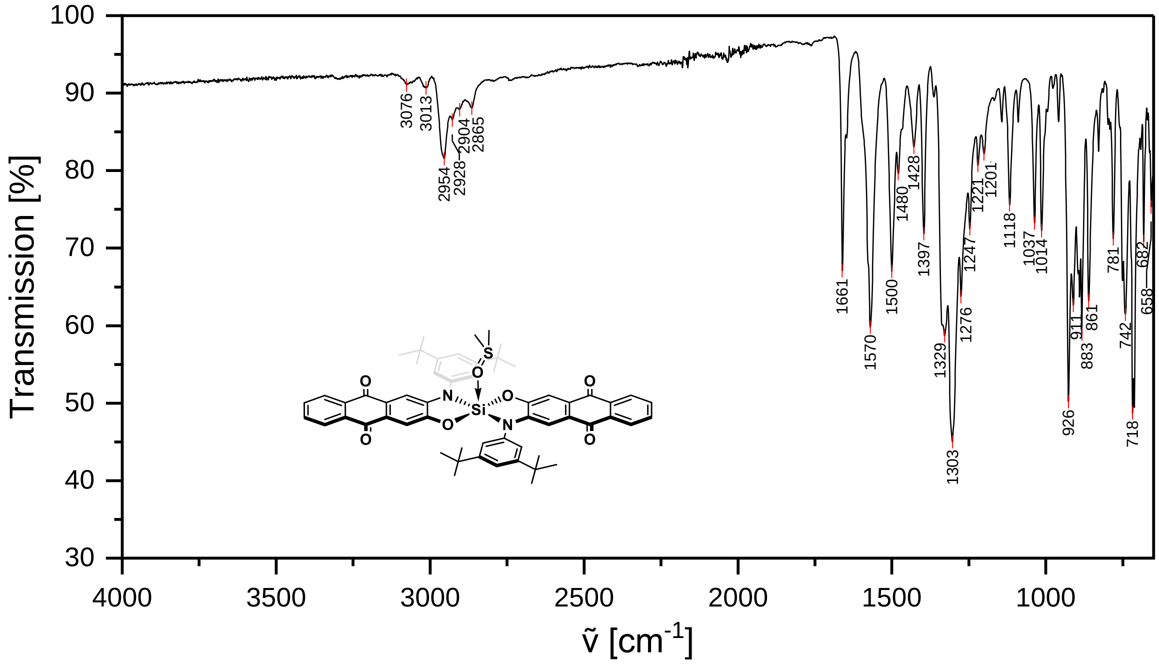

Figure S5.13. ATR-IR spectrum of **1-DMSO**.

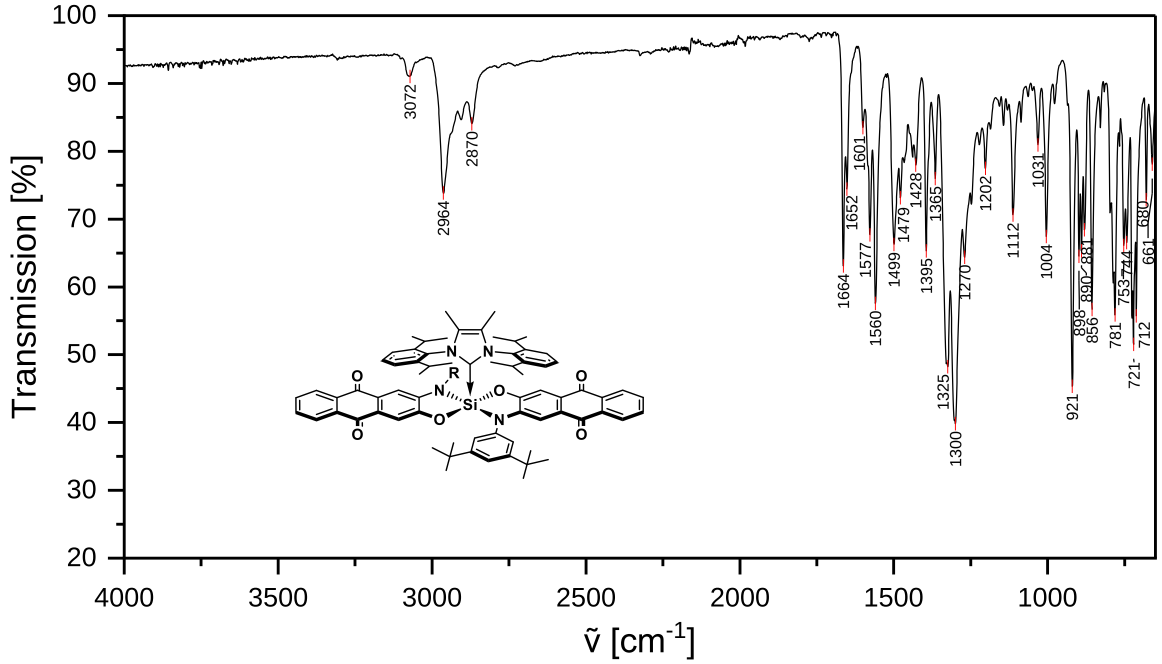

Figure S5.14. ATR-IR spectrum of **1-^{dipp}NHC**.

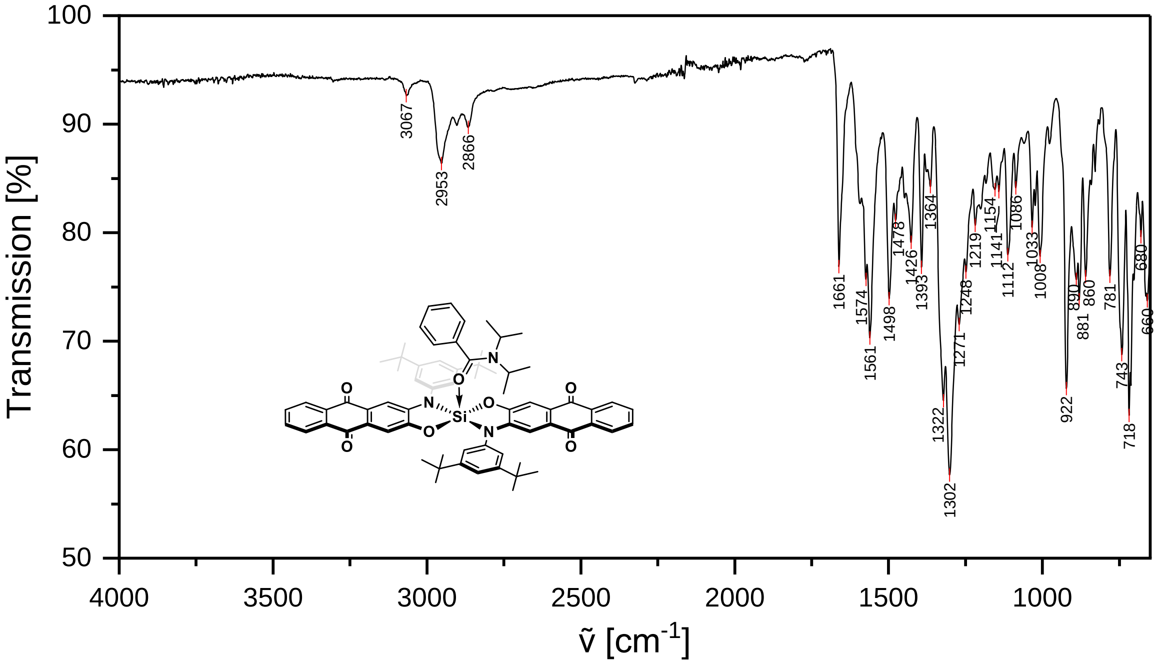

Figure S5.15. ATR-IR spectrum of **1-DIBA**.

6 Coordinates

1

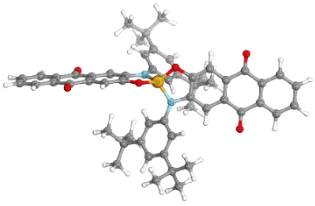

119
xyz, charge: 0, multiplicity: 1

C	-1.162002	2.932379	-0.949295
C	-1.522693	2.319562	0.251783
C	0.148270	2.862885	-1.402219
C	1.110550	2.179219	-0.657911
C	0.747531	1.562284	0.550664
C	-0.572498	1.637324	0.998582
H	-1.908129	3.464959	-1.531945
H	-2.549004	2.375731	0.602926
H	0.452885	3.331241	-2.332833
H	-0.828559	1.151713	1.934960
C	2.508809	2.121429	-1.164123
C	3.499488	1.387885	-0.350009
C	3.140042	0.771327	0.862175
C	1.746020	0.822392	1.365617
C	4.828020	1.340043	-0.797132
C	5.749073	0.671548	-0.032381
C	5.398015	0.030135	1.182677
C	4.089188	0.094559	1.633730
H	3.780725	-0.343508	2.576200
H	5.108443	1.836244	-1.719934
O	2.823012	2.668627	-2.215267
O	1.427346	0.274468	2.414519
O	7.090752	0.610125	-0.341398
N	6.515785	-0.531114	1.799663
C	6.507594	-1.291582	3.000697
C	5.534479	-2.267852	3.226804
C	5.574568	-3.028876	4.389730
C	6.627212	-2.817607	5.294838
C	7.619934	-1.868417	5.071874
C	7.532942	-1.088396	3.914371
H	8.266998	-0.309803	3.726998
H	6.665630	-3.421137	6.193881
H	4.772396	-2.434897	2.475650
C	4.518212	-4.091378	4.701261
C	8.794917	-1.642293	6.024839
C	10.108437	-1.945567	5.275810
H	10.121752	-2.980024	4.915943
H	10.965837	-1.800201	5.943072
H	10.244460	-1.287275	4.411784
C	8.801387	-0.174572	6.494265
H	8.924504	0.522518	5.659037
H	9.631507	-0.007092	7.189661
H	7.865582	0.073787	7.006722
C	8.722554	-2.544504	7.261652
H	8.755430	-3.605774	6.992130
H	7.814358	-2.359947	7.846162
H	9.581445	-2.341796	7.909580
C	5.199977	-5.466489	4.833922
H	5.716310	-5.734393	3.905929
H	4.451338	-6.238017	5.047011
H	5.934777	-5.479784	5.644644
C	3.446754	-4.186809	3.608745
H	3.874830	-4.478452	2.643380
H	2.908019	-3.240983	3.481932
H	2.712472	-4.949765	3.886851
C	3.821245	-3.732031	6.027843
H	4.527567	-3.702381	6.863092
H	3.053950	-4.478449	6.262957
H	3.337549	-2.751904	5.957851
H	11.856014	4.720563	0.361762
H	6.059235	6.401201	3.009718
H	5.148112	4.491631	1.558389
H	9.267519	7.224727	1.495274
H	10.203973	6.722703	3.839855
H	5.849712	2.366200	4.597495
C	13.766331	-0.005306	3.201934
C	11.213777	-2.011765	1.226441
C	10.201600	-1.088402	1.172028
C	8.946001	2.276580	2.026104
C	10.322682	0.199382	1.753372
C	11.488124	0.537522	2.422814
C	12.528468	-0.394210	2.483677
C	14.860543	-1.008014	3.278346
C	16.037783	-0.677915	3.952069
C	14.729047	-2.272930	2.681893
C	15.776864	-3.190587	2.767263
C	9.923244	3.249670	1.803915
C	12.400959	-1.661117	1.885856
C	13.487416	-2.659443	1.958200
C	17.074560	-1.597006	4.031976
C	16.943988	-2.853798	3.438975
C	7.447640	3.940284	2.946752
C	5.837671	3.441544	4.802833
C	7.726543	2.615202	2.597064
C	10.734309	5.666133	1.988491
C	4.992390	3.926297	2.483543
C	5.938506	5.757956	3.888177
C	11.080288	6.275064	3.361179
C	12.028570	5.135154	1.361054
C	10.162471	6.761693	1.068654
C	6.072545	4.274384	3.527769
C	8.442749	4.891641	2.744623
C	9.687189	4.566914	2.181072
N	9.144459	0.931891	1.610029
O	13.373381	-3.768081	1.447335
O	8.972433	-1.342797	0.603113
O	13.884630	1.102255	3.713133
H	4.860582	3.684836	5.235113
H	17.757550	-3.570670	3.502504
H	6.670400	6.059382	4.645793
H	9.894443	6.346377	0.091309
H	11.480311	5.509409	4.034433
H	4.999345	2.860961	2.232003
H	15.651682	-4.161417	2.298196
H	17.989400	-1.336829	4.556520
H	8.254441	5.921189	3.024891
H	11.107788	-2.997525	0.786604
H	6.988734	1.838664	2.778255
H	10.847020	2.965922	1.315104
H	16.115834	0.305936	4.403871
H	11.612606	1.492827	2.919959
H	12.744949	5.956511	1.257045
H	12.497758	4.365094	1.983806
H	4.940554	5.943270	4.298503
H	11.836702	7.059417	3.243853
H	3.997491	4.169143	2.874288
H	10.907752	7.550490	0.915509
H	6.609109	3.651327	5.551758
Si	7.932098	-0.086209	0.908460

[1-H]

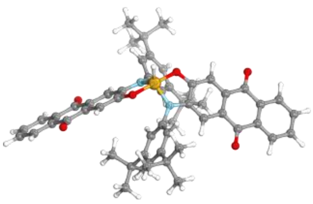

120
xyz, charge: -1, multiplicity: 1

C	-0.797851	4.008743	1.693710
C	-1.040564	3.014757	2.642312
C	0.396706	4.017139	0.984700
C	1.357354	3.032173	1.216437
C	1.113671	2.033820	2.169999
C	-0.088054	2.031621	2.878794
H	-1.543433	4.777680	1.508999
H	-1.974945	3.009297	3.197419
H	0.614137	4.778677	0.242006
H	-0.248847	1.245950	3.610707
C	2.636428	3.064023	0.445291
C	3.609957	1.998467	0.698484
C	3.380190	1.011687	1.685646
C	2.121850	0.964543	2.439139
C	4.823579	2.018155	-0.005321
C	5.783385	1.060061	0.261948
C	5.542450	0.045566	1.237527
C	4.353089	0.036775	1.952132
H	4.156700	-0.708119	2.716273
H	5.011322	2.799756	-0.733571
O	2.840581	3.970821	-0.363212
O	1.879260	0.090467	3.271313
O	6.972328	0.989035	-0.312749
N	6.619290	-0.803186	1.352765
C	6.635721	-1.740839	2.426130
C	5.931673	-2.939703	2.314914
C	5.920945	-3.845897	3.370138
C	6.635127	-3.521365	4.533192
C	7.342822	-2.329165	4.665459
C	7.328484	-1.436819	3.588021
H	7.868799	-0.494773	3.634912
H	6.630783	-4.226434	5.356617
H	5.403265	-3.132599	1.387931
C	5.160512	-5.173137	3.304509
C	8.111919	-1.955623	5.935361
C	9.598773	-1.750811	5.592376
H	10.032345	-2.663777	5.171096
H	10.162458	-1.483094	6.494509
H	9.740596	-0.951578	4.858872
C	7.535317	-0.645094	6.505155
H	7.643813	0.181558	5.796506
H	8.064100	-0.368422	7.425171
H	6.469541	-0.755193	6.734241
C	8.010735	-3.035738	7.018783
H	8.434811	-3.987395	6.679739
H	6.973180	-3.205006	7.329033
H	8.575638	-2.717218	7.901665
C	6.149963	-6.339626	3.487044
H	6.916533	-6.319748	2.705039
H	5.620638	-7.298835	3.431168
H	6.658523	-6.290621	4.454773
C	4.437455	-5.363125	1.965894
H	5.140008	-5.370929	1.125608
H	3.697618	-4.574443	1.791798
H	3.909056	-6.323017	1.969637
C	4.106857	-5.212455	4.427956
H	4.569164	-5.138778	5.417178
H	3.543370	-6.152781	4.387184
H	3.401911	-4.380633	4.323650
H	10.974416	4.227199	-1.775987
H	6.481637	6.495389	2.499503
H	5.163962	4.371614	1.938027
H	8.832965	6.942878	-0.269027
H	10.589548	6.908055	1.609542
H	7.167432	2.852563	4.746165
C	13.168973	0.549938	4.005742
C	11.000468	-2.063086	2.315844
C	10.085495	-1.247103	1.677426
C	9.004051	2.202256	1.242318
C	10.187056	0.173604	1.779726
C	11.185367	0.745495	2.555332
C	12.110172	-0.081738	3.209868
C	14.119478	-0.347384	4.729306
C	15.128125	0.216553	5.511088
C	14.013169	-1.741737	4.630184
C	14.916254	-2.555031	5.315552
C	9.792077	3.098497	0.520764
C	12.025620	-1.488019	3.082108
C	12.940515	-2.375093	3.805534
C	16.024358	-0.599135	6.190163
C	15.918355	-1.986891	6.091927
C	7.871451	4.037107	2.342765
C	7.174132	3.946761	4.743382
C	8.059582	2.664921	2.145741
C	10.471783	5.497488	-0.067049
C	5.435056	3.994253	2.929493
C	6.758319	6.038335	3.456041
C	11.258167	6.362248	0.936694
C	11.477162	4.836986	-1.017654
C	9.540286	6.397362	-0.901200
C	6.822630	4.509967	3.352712

C 8.666602 4.915211 1.610490
C 9.633694 4.469193 0.697317
N 9.185030 0.798310 1.074035
O 12.848417 -3.602746 3.760542
O 9.061357 -1.667381 0.953696
O 13.291739 1.771443 4.096065
H 6.441778 4.287496 5.485099
H 16.619859 -2.624694 6.623316
H 7.713161 6.464338 3.785035
H 8.960540 5.797675 -1.610987
H 11.915835 5.737376 1.550505
H 5.403031 2.901520 2.884516
H 14.808103 -3.631277 5.221814
H 16.807957 -0.155268 6.798594
H 8.538517 5.982807 1.748342
H 10.915854 -3.142614 2.253071
H 7.460907 1.935451 2.685223
H 10.517974 2.689698 -0.173231
H 15.185770 1.299228 5.568716
H 11.267378 1.821416 2.669705
H 12.050998 5.612141 -1.537713
H 12.186555 4.201662 -0.476497
H 5.995945 6.320096 4.190516
H 11.874487 7.096651 0.403614
H 4.673345 4.323847 3.646712
H 10.127228 7.132903 -1.465016
H 8.169958 4.278402 5.057772
Si 8.017034 -0.343280 0.311309
H 8.191007 -0.864399 -1.068207

[1-F]

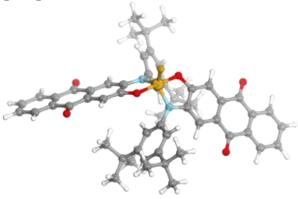

120
xyz, charge: -1, multiplicity: 1
C -0.881759 3.925368 1.564495
C -1.147329 2.913244 2.487581
C 0.337155 3.958130 0.899271
C 1.299618 2.979394 1.149370
C 1.033013 1.962863 2.077318
C -0.193218 1.936474 2.742438
H -1.628788 4.689257 1.365413
H -2.100962 2.888743 3.008313
H 0.572552 4.734151 0.177352
H -0.371578 1.137286 3.455386
C 2.604707 3.037537 0.425109
C 3.578419 1.974473 0.691631
C 3.325055 0.969818 1.654843
C 2.042362 0.899932 2.365463
C 4.811997 2.013907 0.025219
C 5.769037 1.059176 0.310628
C 5.507807 0.031026 1.263873
C 4.296570 -0.001235 1.938454
H 4.082691 -0.758305 2.685669
H 5.016830 2.807640 -0.684992
O 2.829849 3.961347 -0.357512
O 1.781413 0.011169 3.175823
O 6.982050 1.016662 -0.215530
N 6.598124 -0.798332 1.405720
C 6.608767 -1.740990 2.475358
C 5.890375 -2.930911 2.364432
C 5.877286 -3.840353 3.417025
C 6.606354 -3.529602 4.574568
C 7.329613 -2.346347 4.705230
C 7.313547 -1.448530 3.632919
H 7.864234 -0.512353 3.679190
H 6.602005 -4.238630 5.394569
H 5.355085 -3.116190 1.439872
C 5.100476 -5.158106 3.352389
C 8.117390 -1.988013 5.968190
C 9.601838 -1.791233 5.609134
H 10.023834 -2.703064 5.173777
H 10.177979 -1.537143 6.507264
H 9.741622 -0.985662 4.882109
C 7.557287 -0.677860 6.555026
H 7.661245 0.153117 5.850706
H 8.100031 -0.411107 7.469844
H 6.494073 -0.783086 6.797857
C 8.021727 -3.075866 7.044327
H 8.433122 -4.028221 6.691950
H 6.987055 -3.239084 7.367070
H 8.600850 -2.769197 7.922164
C 6.077491 -6.337156 3.520743
H 6.836992 -6.323044 2.731769
H 5.536041 -7.289557 3.465321
H 6.595262 -6.298863 4.484096
C 4.362947 -5.333138 2.019647
H 5.057419 -5.346223 1.172782
H 3.631348 -4.534632 1.855703
H 3.822610 -6.286321 2.024262
C 4.056773 -5.189664 4.485327
H 4.528796 -5.125451 5.470642
H 3.482079 -6.123204 4.445884
H 3.360397 -4.349508 4.390528
H 11.050539 4.247438 -1.705943
H 6.486522 6.537288 2.477551
H 5.175185 4.407188 1.910147
H 8.904856 6.979144 -0.233305
H 10.628932 6.928629 1.675256
H 7.138407 2.909544 4.758851
C 13.264717 0.555490 3.921139
C 10.998037 -2.040329 2.335719
C 10.082450 -1.214416 1.712439
C 9.017125 2.236664 1.279821
C 10.211794 0.203720 1.792019
C 11.243397 0.768725 2.526628
C 12.170047 -0.067195 3.166743
C 14.219360 -0.349503 4.629142
C 15.263738 0.206146 5.368921
C 14.081547 -1.742835 4.557574
C 14.989218 -2.563406 5.228137
C 9.822261 3.126665 0.570184
C 12.053641 -1.473424 3.065038
C 12.970636 -2.367493 3.778504
C 16.164305 -0.616736 6.033141
C 16.026912 -2.003503 5.962347
C 7.876745 4.078366 2.359588
C 7.142363 4.003775 4.749529
C 8.061885 2.705337 2.168614
C 10.528752 5.520716 -0.004819
C 5.430463 4.039587 2.909501
C 6.746316 6.086718 3.441864
C 11.304625 6.378554 1.012979
C 11.544886 4.852881 -0.938703
C 9.618429 6.428314 -0.853781
C 6.812100 4.557688 3.349894
C 8.689991 4.951166 1.640563
C 9.670290 4.498474 0.744952
N 9.189127 0.830615 1.115873
O 12.849400 -3.592887 3.760307
O 9.020832 -1.626161 1.038685
O 13.413372 1.775354 3.987856
H 6.397662 4.347433 5.477492
H 16.731984 -2.646858 6.482136
H 7.695370 6.514853 3.784435
H 9.046298 5.833704 -1.573926
H 11.947587 5.748201 1.636707
H 5.396658 2.946457 2.875342
H 14.856233 -3.638565 5.156560
H 16.975925 -0.179380 6.608613
H 8.565676 6.019654 1.774833
H 10.890087 -3.118658 2.291998
H 7.450997 1.979764 2.699819
H 10.554529 2.713457 -0.114428
H 15.345364 1.288158 5.406189
H 11.348660 1.844306 2.623177
H 12.133443 5.623845 -1.448366
H 12.240207 4.211892 -0.386067
H 5.971586 6.373599 4.161251
H 11.935143 7.108842 0.491023
H 4.658346 4.377996 3.611232
H 10.220649 7.159511 -1.406957
H 8.132257 4.339945 5.077533
Si 8.001465 -0.307429 0.405574
F 8.190265 -0.877308 -1.101742

[1-F_2]

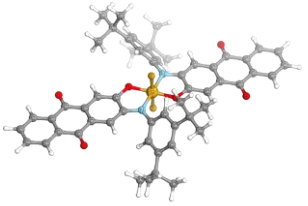

121
xyz, charge: -2, multiplicity: 1
C -1.746229 2.685171 0.542721
C -2.058973 1.403577 0.994751
C -0.432246 3.138989 0.589087
C 0.581161 2.317685 1.086058
C 0.265827 1.029150 1.539911
C -1.053626 0.579459 1.490591
H -2.529800 3.331455 0.153484
H -3.086012 1.047672 0.959530
H -0.155984 4.131839 0.246541
H -1.261450 -0.424742 1.848291
C 1.986215 2.833958 1.133185
C 3.010956 1.941994 1.644883
C 2.695294 0.641906 2.127337
C 1.331651 0.119861 2.068515
C 4.340708 2.392033 1.713643
C 5.341557 1.577290 2.217460
C 5.035736 0.239892 2.668460
C 3.701451 -0.185500 2.634774
H 3.415078 -1.160234 3.013040
H 4.583685 3.391139 1.368071
O 2.213584 3.987869 0.741426
O 1.023970 -1.021414 2.428409
O 6.591481 1.958349 2.341854
N 6.124830 -0.402206 3.145007
C 6.064259 -1.713856 3.628126
C 5.430998 -2.734372 2.901004
C 5.387675 -4.034190 3.387416
C 6.020039 -4.317852 4.607867
C 6.677392 -3.326290 5.335614
C 6.674567 -2.021429 4.842735
H 7.167664 -1.213121 5.371215
H 6.001305 -5.335218 4.983474
H 5.001059 -2.478156 1.940344
C 4.681804 -5.163087 2.628309
C 7.421362 -3.612965 6.643227
C 8.911147 -3.270795 6.450409
H 9.348933 -3.889945 5.660089
H 9.470573 -3.447633 7.377943
H 9.049146 -2.225955 6.157836
C 6.838120 -2.741098 7.771113
H 6.937463 -1.676024 7.544386
H 7.363832 -2.938117 8.714461
H 5.772790 -2.956732 7.913099
C 7.314386 -5.081237 7.072774
H 7.749743 -5.751668 6.323345
H 6.272567 -5.378703 7.241778
H 7.863694 -5.227821 8.010282
C 5.698053 -6.267489 2.281287
H 6.505650 -5.861588 1.662588
H 5.208068 -7.079501 1.727842
H 6.150861 -6.694212 3.181972
C 4.037746 -4.675693 1.324697
H 4.785559 -4.274643 0.632409
H 3.289574 -3.897440 1.510885
H 3.536024 -5.515343 0.828752
C 3.568287 -5.753602 3.514799
H 3.971541 -6.162733 4.446714
H 3.045353 -6.560952 2.985247
H 2.838391 -4.979124 3.774361
H 11.353768 3.319255 -1.291432
H 7.444187 7.793422 1.531510
H 5.876539 5.816489 1.842693
H 9.707556 6.708048 -1.108186
H 11.548880 7.201438 0.623498
H 7.678471 5.403281 5.136241
C 14.139472 1.021300 3.244082
C 11.111333 -1.192542 3.766104
C 10.089891 -0.316374 3.437200
C 9.332783 3.077081 2.317964
C 10.392315 1.029985 3.011382

C 11.732270 1.438300 2.995215
C 12.758721 0.550648 3.331381
C 15.222337 0.061566 3.629624
C 16.552608 0.478933 3.587264
C 14.914603 -1.249271 4.020441
C 15.946103 -2.124485 4.364679
C 10.171424 3.469605 1.262643
C 12.455789 -0.786880 3.708041
C 13.496438 -1.726214 4.085065
C 17.575816 -0.398307 3.932614
C 17.270700 -1.702387 4.321085
C 8.506760 5.352495 2.493485
C 7.811529 6.384942 4.673412
C 8.513446 4.026411 2.925968
C 11.133609 5.253683 -0.294983
C 6.096131 5.847971 2.915272
C 7.667739 7.758075 2.603622
C 12.094247 6.331791 0.242987
C 11.980691 4.108052 -0.862321
C 10.292375 5.845015 -1.441966
C 7.537641 6.334365 3.158536
C 9.367412 5.723853 1.460855
C 10.209507 4.790598 0.836552
N 9.277732 1.747887 2.751178
O 13.271561 -2.887358 4.456400
O 8.816807 -0.627283 3.512489
O 14.448511 2.157946 2.870991
H 7.122309 7.086568 5.161041
H 18.068497 -2.390869 4.590674
H 8.674088 8.163708 2.762250
H 9.591684 5.095876 -1.826114
H 12.698291 5.927352 1.062419
H 5.940563 4.839032 3.307997
H 15.674880 -3.132489 4.664315
H 18.611029 -0.066797 3.898584
H 9.384584 6.753978 1.121738
H 10.873054 -2.204782 4.074674
H 7.866305 3.686249 3.726776
H 10.766380 2.704463 0.778925
H 16.754742 1.500164 3.277773
H 12.004432 2.454964 2.735312
H 12.629657 4.492983 -1.658155
H 12.619243 3.659404 -0.093551
H 6.954255 8.415389 3.114535
H 12.770442 6.673271 -0.551939
H 5.375502 6.521781 3.396164
H 10.941845 6.170843 -2.265213
H 8.839354 6.712186 4.868470
Si 7.701304 0.673363 2.950125
F 7.884748 0.113975 1.366025
F 7.516840 1.234928 4.540997

[1-Cl]

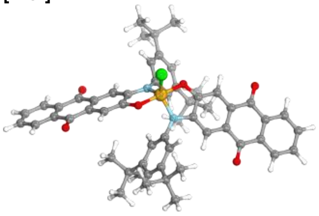

120
xyz, charge: -1, multiplicity: 1
C -0.943126 3.912469 1.511369
C -1.220836 2.885781 2.414628
C 0.290214 3.964516 0.874654
C 1.255145 2.990493 1.133519
C 0.976342 1.959317 2.041583
C -0.264408 1.913696 2.678222
H -1.692102 4.672595 1.305384
H -2.185853 2.846274 2.912950
H 0.535133 4.752217 0.168743
H -0.452155 1.103518 3.376178
C 2.575745 3.069607 0.440276
C 3.551456 2.009620 0.712889
C 3.285586 0.990086 1.656595
C 1.987610 0.900879 2.338172
C 4.798364 2.066845 0.072950
C 5.755547 1.117206 0.370179
C 5.483547 0.074305 1.302037
C 4.257743 0.022586 1.948677
H 4.034099 -0.745652 2.681424
H 5.012556 2.870423 -0.623163
O 2.812149 4.006818 -0.322495
O 1.716963 -0.000872 3.130390
O 6.984308 1.096415 -0.126586
N 6.579842 -0.742941 1.458766
C 6.577690 -1.719762 2.496130
C 5.847271 -2.897951 2.345482
C 5.829728 -3.843713 3.365383
C 6.567602 -3.581686 4.529529
C 7.303632 -2.411189 4.698678
C 7.290311 -1.474994 3.659855
H 7.849611 -0.545927 3.736299
H 6.560797 -4.320162 5.323109
H 5.309153 -3.046750 1.415976
C 5.040888 -5.151382 3.256930
C 8.103042 -2.106561 5.968494
C 9.586110 -1.903079 5.606501
H 10.000364 -2.798653 5.131734
H 10.169933 -1.688621 6.509944
H 9.725605 -1.069523 4.911658
C 7.553719 -0.817865 6.610385
H 7.654341 0.039162 5.937502
H 8.105437 -0.588551 7.529998
H 6.492345 -0.928818 6.858713
C 8.010038 -3.235529 7.001642
H 8.412100 -4.175602 6.607948
H 6.977263 -3.405199 7.327090
H 8.598762 -2.967405 7.885680
C 6.008358 -6.344081 3.377462
H 6.763962 -6.307818 2.585488
H 5.458256 -7.289084 3.290280
H 6.531078 -6.345319 4.338939
C 4.295608 -5.271314 1.922414
H 4.985828 -5.260304 1.072082
H 3.570665 -4.460800 1.790915
H 3.746372 -6.219007 1.895387
C 4.002369 -5.214834 4.393268
H 4.479395 -5.189868 5.377976
H 3.419745 -6.141603 4.323183
H 3.312499 -4.366262 4.331749
H 11.164310 4.261684 -1.574416
H 6.518438 6.620479 2.471273
H 5.198714 4.492995 1.898910
H 9.033019 7.033170 -0.154367
H 10.707635 6.949790 1.797026
H 7.092446 3.012282 4.804165
C 13.307595 0.529919 3.925524
C 10.993988 -2.021212 2.334835
C 10.073754 -1.176526 1.746483
C 9.028644 2.288621 1.365421
C 10.213386 0.237788 1.847360
C 11.262913 0.782480 2.572075
C 12.194245 -0.071597 3.180067
C 14.268735 -0.394257 4.598618
C 15.331479 0.140709 5.327448
C 14.119416 -1.785137 4.504822
C 15.034087 -2.623961 5.142492
C 9.864454 3.164120 0.673204
C 12.065906 -1.474762 3.055998
C 12.988771 -2.388028 3.737340
C 16.238910 -0.700279 5.958723
C 16.090079 -2.084580 5.865775
C 7.888457 4.149664 2.410649
C 7.102010 4.106335 4.784259
C 8.058596 2.773490 2.229040
C 10.623821 5.545097 0.113266
C 5.429496 4.136338 2.908145
C 6.750536 6.178231 3.446447
C 11.389561 6.388491 1.150682
C 11.650401 4.858760 -0.795450
C 9.751307 6.469070 -0.757273
C 6.806043 4.647830 3.372130
C 8.733333 5.008073 1.710861
C 9.729529 4.538326 0.841828
N 9.179468 0.880709 1.205713
O 12.856164 -3.611633 3.701595
O 8.992572 -1.571145 1.088809
O 13.465586 1.747200 4.010844
H 6.343369 4.461328 5.492149
H 16.800656 -2.742067 6.359697
H 7.694211 6.602605 3.807942
H 9.186464 5.885103 -1.491731
H 12.006146 5.746457 1.788956
H 5.385608 3.043278 2.887594
H 14.891982 -3.696716 5.054382
H 17.064915 -0.279034 6.525624
H 8.621633 6.078719 1.838896
H 10.877369 -3.097609 2.271960
H 7.425027 2.058038 2.747190
H 10.605249 2.738286 0.005692
H 15.421855 1.221255 5.382492
H 11.377463 1.855280 2.686585
H 12.264889 5.618798 -1.290670
H 12.320596 4.205927 -0.225897
H 5.960842 6.478892 4.143579
H 12.045347 7.107949 0.645106
H 4.645639 4.491082 3.588396
H 10.380462 7.188973 -1.294995
H 8.086134 4.441080 5.130581
Si 7.988856 -0.239662 0.474926
Cl 8.242901 -0.958392 -1.521528

[1-Cl_2]

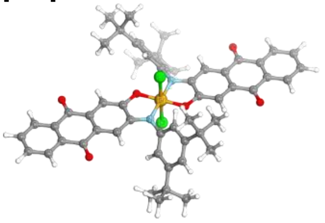

121
xyz, charge: -2, multiplicity: 1
C -1.765205 2.846262 0.999809
C -2.070092 1.548993 1.411868
C -0.449100 3.293842 1.024187
C 0.574709 2.450680 1.459382
C 0.267464 1.147057 1.872852
C -1.054859 0.703453 1.845756
H -2.557221 3.509268 0.659103
H -3.099264 1.198300 1.393694
H -0.179009 4.298104 0.711405
H -1.256807 -0.312866 2.171010
C 1.982466 2.960026 1.483669
C 3.019008 2.046854 1.933903
C 2.713901 0.730203 2.374291
C 1.343691 0.215176 2.335245
C 4.352438 2.493099 1.971659
C 5.353425 1.652212 2.413704
C 5.056654 0.315411 2.841607
C 3.728428 -0.119930 2.823392
H 3.460469 -1.114782 3.161517
H 4.590330 3.499476 1.645282
O 2.205209 4.124478 1.125664
O 1.044465 -0.936458 2.661275
O 6.626707 1.996655 2.492042
N 6.163746 -0.336842 3.261279
C 6.117525 -1.689234 3.648737
C 5.591927 -2.653654 2.780516
C 5.556964 -3.991669 3.151226
C 6.083009 -4.355030 4.400845
C 6.627387 -3.410912 5.269996
C 6.622896 -2.068907 4.885962
H 7.031897 -1.289944 5.520780
H 6.072084 -5.401228 4.687634
H 5.244864 -2.319622 1.809849
C 4.984739 -5.076053 2.232594
C 7.250604 -3.782122 6.618264
C 8.738126 -3.380609 6.603955
H 9.273563 -3.913116 5.810528
H 9.210950 -3.623798 7.564055
H 8.862385 -2.309137 6.421827
C 6.527696 -3.021127 7.745652
H 6.608412 -1.938682 7.612557
H 6.966132 -3.277692 8.718782
H 5.462616 -3.279552 7.763479
C 7.156945 -5.283235 6.916973
H 7.690244 -5.876819 6.165905
H 6.115294 -5.623813 6.953520
H 7.614021 -5.490578 7.891736
C 6.087079 -6.097600 1.894373
H 6.927352 -5.600398 1.398184
H 5.696267 -6.875651 1.225390
H 6.472647 -6.585686 2.795254

C 4.446722 -4.497990 0.918178
H 5.235967 -4.000488 0.344994
H 3.644266 -3.773988 1.096643
H 4.040266 -5.308526 0.301367
C 3.824071 -5.793132 2.948449
H 4.156888 -6.270428 3.875812
H 3.396664 -6.569359 2.300100
H 3.033407 -5.078612 3.202156
H 10.931327 2.900527 -1.448918
H 7.527890 7.786531 1.353027
H 5.918978 5.918238 1.994154
H 9.446026 6.367040 -1.403405
H 11.482406 6.921171 0.072473
H 8.048564 5.678147 5.102190
C 14.075865 1.088963 3.443251
C 11.046831 -1.125075 3.988079
C 10.039066 -0.262520 3.607543
C 9.279711 3.064463 2.331116
C 10.337695 1.068426 3.163013
C 11.670565 1.489062 3.139417
C 12.692379 0.615815 3.522971
C 15.154989 0.147507 3.878792
C 16.484448 0.568537 3.842237
C 14.845493 -1.149331 4.311473
C 15.874033 -2.007981 4.703013
C 10.029155 3.320071 1.176504
C 12.389371 -0.707691 3.943903
C 13.428542 -1.629531 4.369718
C 17.504202 -0.291778 4.234497
C 17.197136 -1.582567 4.664893
C 8.581711 5.379712 2.403981
C 8.170903 6.613427 4.548781
C 8.569852 4.091300 2.940962
C 10.897161 4.922672 -0.616691
C 6.253854 6.030078 3.031038
C 7.863198 7.825578 2.395569
C 11.949892 5.996531 -0.281311
C 11.631485 3.692723 -1.163958
C 9.958207 5.451870 -1.717381
C 7.732805 6.459647 3.080019
C 9.349068 5.620400 1.265428
C 10.082608 4.600049 0.640042
N 9.217405 1.762277 2.861897
O 13.200464 -2.777441 4.774709
O 8.754404 -0.570857 3.631718
O 14.384111 2.212655 3.037765
H 7.568041 7.384275 5.046160
H 17.992880 -2.257696 4.971385
H 8.897108 8.190335 2.414692
H 9.192828 4.705683 -1.955904
H 12.623125 5.637321 0.504678
H 6.098481 5.069819 3.531431
H 15.601723 -3.005720 5.034208
H 18.538721 0.041988 4.204867
H 9.375992 6.618185 0.840451
H 10.808512 -2.130524 4.317025
H 7.992830 3.844763 3.826024
H 10.535096 2.482715 0.710539
H 16.688463 1.578793 3.499842
H 11.934974 2.495027 2.832841
H 12.204085 3.975452 -2.055468
H 12.332036 3.282736 -0.428388
H 7.237632 8.557607 2.919845
H 12.548758 6.235597 -1.170029
H 5.619560 6.780067 3.520603
H 10.525918 5.674960 -2.630349
H 9.226266 6.903703 4.607586
Si 7.690628 0.712570 3.060929
Cl 7.934053 -0.059252 0.867847
Cl 7.443275 1.496155 5.287640

[1-Br]

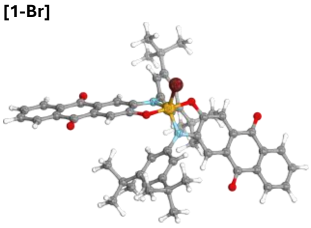

120
xyz, charge: -1, multiplicity: 1
C 2.813398 -2.605114 2.005850
C 4.170475 -2.719667 1.702237
C 4.822213 -1.692996 1.031603
C 4.123843 -0.544150 0.658924
C 2.759940 -0.429568 0.962859
C 2.111896 -1.464427 1.637882
C 4.846108 0.546477 -0.061542
C 4.080929 1.748915 -0.418603
C 2.696604 1.846250 -0.144753
C 1.985619 0.787958 0.579625
C 4.754682 2.801266 -1.054015
C 4.050400 3.941237 -1.412076
C 2.658642 4.039028 -1.125851
C 1.989197 3.003666 -0.504638
O 0.795610 0.878917 0.882167
O 6.039061 0.418314 -0.331792
O 2.113394 5.200352 -1.462618
N 4.516202 5.100430 -1.988060
C 5.918271 5.273571 -2.175554
C 6.564401 4.634173 -3.232366
C 7.934859 4.792361 -3.410233
C 8.632971 5.610848 -2.509699
C 8.004588 6.258837 -1.448828
C 6.628312 6.068976 -1.289982
C 7.607331 15.083391 -0.366216
C 6.859558 15.119032 0.811190
C 5.984793 14.082830 1.111086
C 5.850063 13.003508 0.237269
C 6.600700 12.967941 -0.946212
C 7.478727 14.011661 -1.240230
C 4.901035 11.902727 0.579462
C 4.796501 10.778210 -0.359944
C 5.524513 10.762860 -1.572080
C 6.482404 11.823715 -1.899076
C 3.935384 9.721142 -0.035226
C 3.803572 8.656823 -0.914458
C 4.556089 8.634882 -2.123813
C 5.397560 9.678539 -2.453067
O 7.182198 11.794346 -2.911555
O 4.244122 11.959532 1.618025
O 4.395727 7.532776 -2.846683
N 3.055383 7.509924 -0.779758
C 2.365914 7.244556 0.437319
C 1.244172 7.987798 0.788075
C 0.566394 7.712401 1.976075
C 1.033059 6.666061 2.776209
C 2.156980 5.906524 2.440611
C 2.830792 6.226836 1.264741
Si 3.261616 6.367435 -2.143659
C 8.754050 7.148440 -0.453542
C 8.151511 8.565755 -0.476586
C 8.604471 6.554759 0.960763
C 10.249372 7.253358 -0.774580
C 8.697255 4.110618 -4.549578
C 9.372657 5.181205 -5.426948
C 9.771034 3.179193 -3.955489
C 7.776420 3.271080 -5.443195
C 2.602757 4.755352 3.346644
C 1.478668 3.704081 3.414663
C 3.870309 4.064880 2.827239
C 2.890945 5.297423 4.759311
C -0.667441 8.505854 2.416186
C -1.868877 7.551478 2.550616
C -0.383508 9.170971 3.776417
C -1.039766 9.606229 1.415314
H 2.304102 -3.408976 2.530686
H 4.718676 -3.612511 1.990944
H 5.877220 -1.754216 0.782636
H 1.056025 -1.346896 1.861563
H 5.819277 2.709588 -1.242222
H 0.933423 3.088103 -0.271204
H 5.964755 4.027677 -3.901745
H 9.700129 5.740173 -2.649825
H 6.085762 6.551900 -0.481283
H 8.291402 15.894686 -0.600612
H 6.961463 15.957770 1.494697
H 5.390669 14.083682 2.019827
H 8.048602 13.955911 -2.162633
H 3.394350 9.752473 0.904468
H 5.983277 9.650645 -3.365353
H 0.913050 8.766341 0.109913
H 0.498991 6.430533 3.692679
H 3.711996 5.676508 0.954018
H -2.077479 7.059454 1.594658
H -2.763123 8.107387 2.858139
H -1.684765 6.770216 3.294561
H -1.256367 9.747292 4.106911
H 0.471897 9.851002 3.700961
H -0.155968 8.429625 4.548799
H -1.284565 9.190880 0.431875
H -0.229005 10.332485 1.292824
H -1.920980 10.146162 1.779046
H 1.261390 3.301123 2.420078
H 0.553546 4.128537 3.818110
H 1.779076 2.869894 4.060227
H 2.001501 5.747647 5.211643
H 3.675089 6.061788 4.726218
H 3.227077 4.484697 5.414382
H 3.716435 3.629447 1.834240
H 4.145717 3.249808 3.505410
H 4.718322 4.757191 2.775332
H 8.365937 2.807024 -6.241585
H 7.001471 3.886225 -5.912768
H 7.287720 2.469677 -4.878824
H 9.913690 4.706831 -6.254849
H 10.088685 5.781216 -4.856872
H 8.625066 5.862683 -5.846687
H 10.323777 2.676258 -4.758418
H 9.308364 2.414865 -3.321799
H 10.491180 3.731631 -3.343818
H 10.420236 7.689437 -1.765075
H 10.743064 6.275624 -0.733255
H 10.733922 7.903216 -0.037766
H 8.231081 9.011665 -1.473573
H 8.679769 9.212591 0.234442
H 7.092064 8.561851 -0.203048
H 9.004424 5.535474 0.998528
H 7.555837 6.516455 1.270767
H 9.147388 7.169971 1.688383
Br 1.936171 6.534510 -4.041150

[1-Br$_2$]

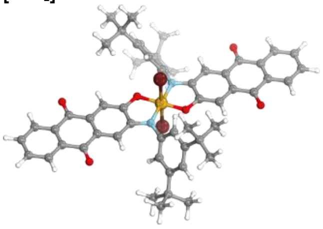

121
xyz, charge: -2, multiplicity: 1
C -1.757012 2.892535 1.072236
C -2.062961 1.592576 1.475511
C -0.439449 3.335312 1.089301
C 0.584790 2.484661 1.508532
C 0.276613 1.178655 1.913159
C -1.047526 0.739799 1.893434
H -2.549572 3.561153 0.744180
H -3.093477 1.245748 1.462977
H -0.168591 4.341314 0.782885
H -1.250593 -0.278457 2.211766
C 1.994480 2.988509 1.524206
C 3.032382 2.067981 1.959599
C 2.726759 0.748364 2.389483
C 1.352817 0.239401 2.359576
C 4.367141 2.510892 1.987407
C 5.365440 1.660475 2.412478
C 5.067797 0.325771 2.835277
C 3.741117 -0.109985 2.823020
H 3.476098 -1.108807 3.151827
H 4.605858 3.519015 1.667362
O 2.219603 4.153461 1.172239
O 1.052856 -0.912434 2.681337
O 6.646631 1.990611 2.478393
N 6.180108 -0.331117 3.238082
C 6.130803 -1.687511 3.624785
C 5.657174 -2.651652 2.729248
C 5.625820 -3.991867 3.093802
C 6.098442 -4.351190 4.365804
C 6.587942 -3.403558 5.263141
C 6.583602 -2.060421 4.882263

H 6.952842 -1.276061 5.535683
H 6.090350 -5.398716 4.648192
H 5.352774 -2.315965 1.744756
C 5.118704 -5.081598 2.144188
C 7.153801 -3.769074 6.637585
C 8.635052 -3.347462 6.691137
H 9.212852 -3.869609 5.920812
H 9.066952 -3.588297 7.670916
H 8.752215 -2.273241 6.520031
C 6.371022 -3.018065 7.731218
H 6.445841 -1.934457 7.603328
H 6.767351 -3.271282 8.723056
H 5.309298 -3.288720 7.700044
C 7.066265 -5.271802 6.929564
H 7.641263 -5.857155 6.203145
H 6.028580 -5.626017 6.917870
H 7.480648 -5.474919 7.924061
C 6.255485 -6.080605 1.856113
H 7.109033 -5.565164 1.403168
H 5.912121 -6.862301 1.165755
H 6.605964 -6.566151 2.772610
C 4.636861 -4.505906 0.807104
H 5.443842 -3.988399 0.278009
H 3.811863 -3.799322 0.948591
H 4.278774 -5.320387 0.166040
C 3.938026 -5.823342 2.799321
H 4.234295 -6.300433 3.739145
H 3.556294 -6.603132 2.127223
H 3.123263 -5.124681 3.018709
H 10.736989 2.832985 -1.525171
H 7.566834 7.799965 1.410411
H 5.953657 5.959882 2.128085
H 9.309335 6.322344 -1.432939
H 11.427499 6.856930 -0.069750
H 8.214484 5.683813 5.135989
C 14.059594 1.091809 3.492069
C 11.029250 -1.128762 4.012574
C 10.027722 -0.269615 3.613488
C 9.270921 3.048994 2.318068
C 10.327272 1.058015 3.170289
C 11.656887 1.484540 3.154489
C 12.675236 0.615186 3.555622
C 15.135286 0.155282 3.945436
C 16.464120 0.579570 3.922590
C 14.823829 -1.139467 4.382445
C 15.849382 -1.993203 4.792060
C 9.973859 3.283290 1.132021
C 12.370344 -0.705540 3.982688
C 13.407715 -1.623289 4.425129
C 17.480670 -0.275655 4.332730
C 17.171512 -1.564622 4.767613
C 8.621040 5.374027 2.417283
C 8.331839 6.616026 4.576147
C 8.608483 4.086807 2.957836
C 10.778638 4.862273 -0.710867
C 6.337155 6.069499 3.148281
C 7.950887 7.832966 2.436258
C 11.862740 5.922425 -0.438516
C 11.463468 3.616437 -1.285484
C 9.791690 5.397622 -1.765713
C 7.824881 6.470765 3.128820
C 9.338911 5.595452 1.243213
C 10.024131 4.560475 0.587637
N 9.205341 1.743867 2.851366
O 13.176853 -2.769411 4.831072
O 8.739169 -0.576883 3.613776
O 14.368778 2.214197 3.085992
H 7.768031 7.399602 5.098734
H 17.965094 -2.235614 5.088266
H 8.991759 8.176651 2.406801
H 9.004894 4.660781 -1.959128
H 12.569827 5.559753 0.315530
H 6.186284 5.115293 3.661739
H 15.575661 -2.989599 5.126058
H 18.514638 0.060460 4.313859
H 9.363362 6.591047 0.812737
H 10.789649 -2.132981 4.344073
H 8.066585 3.850487 3.868372
H 10.440017 2.432732 0.648451
H 16.670027 1.588216 3.576668
H 11.919645 2.488645 2.840265
H 11.992196 3.882862 -2.208547
H 12.195668 3.201393 -0.584411
H 7.365406 8.578114 2.987601
H 12.418999 6.145407 -1.358502
H 5.740747 6.834147 3.662194
H 10.315053 5.606209 -2.708091
H 9.394544 6.884399 4.587100
Si 7.694511 0.701258 3.030185
Br 7.963926 -0.138254 0.637247
Br 7.416570 1.566502 5.499908

[1-I]

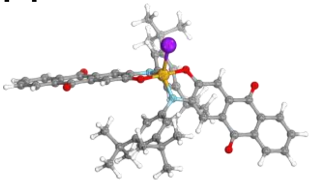

120
xyz, charge: -1, multiplicity: 1
C 2.490802 -2.812229 1.512677
C 3.848169 -2.945325 1.217941
C 4.539688 -1.891947 0.634167
C 3.881034 -0.697538 0.340196
C 2.517017 -0.564038 0.635641
C 1.828948 -1.626067 1.223211
C 4.644587 0.419992 -0.290225
C 3.919238 1.666916 -0.569856
C 2.535173 1.786654 -0.305916
C 1.785064 0.703241 0.338208
C 4.631448 2.738546 -1.125799
C 3.965265 3.920265 -1.414208
C 2.577803 4.044619 -1.119791
C 1.868210 2.987922 -0.586793
O 0.597096 0.811006 0.641205
O 5.837653 0.277009 -0.553176
O 2.072122 5.248172 -1.369091
N 4.467815 5.097509 -1.921198
C 5.869809 5.244518 -2.117915
C 6.508577 4.557316 -3.149662
C 7.880743 4.690825 -3.332353
C 8.589038 5.537124 -2.466011
C 7.967971 6.236352 -1.433807
C 6.590058 6.067874 -1.266146
C 7.509116 15.191683 -0.561655
C 6.813735 15.228185 0.647511
C 5.979009 14.176291 1.001666
C 5.832012 13.080272 0.150776
C 6.530113 13.043837 -1.064452
C 7.368344 14.103387 -1.412917
C 4.925198 11.963653 0.550573
C 4.801584 10.824771 -0.369614
C 5.480261 10.804707 -1.609937
C 6.397869 11.882514 -1.994395
C 3.971407 9.758857 0.003074
C 3.821298 8.682152 -0.857805
C 4.530303 8.653090 -2.092483
C 5.338362 9.706098 -2.470528
O 7.051663 11.853923 -3.036755
O 4.315587 12.019567 1.617461
O 4.363692 7.534999 -2.791749
N 3.089941 7.529563 -0.683103
C 2.439376 7.278017 0.557683
C 1.309517 8.011434 0.918782
C 0.678459 7.767229 2.133757
C 1.200353 6.766043 2.966748
C 2.326442 6.022282 2.623036
C 2.947390 6.302366 1.401733
Si 3.238715 6.388655 -2.050316
C 8.727863 7.158441 -0.476502
C 8.130355 8.576566 -0.545130
C 8.586511 6.617565 0.959575
C 10.220816 7.246706 -0.813041
C 8.635025 3.947781 -4.438329
C 9.352579 4.963921 -5.346449
C 9.673217 3.007694 -3.796190
C 7.699706 3.103048 -5.311661
C 2.913244 4.933757 3.525928
C 2.935731 3.593288 2.767481
C 4.352615 5.324431 3.912815
C 2.103552 4.743035 4.813922
C -0.562659 8.543440 2.582192
C -1.732836 7.561212 2.781072
C -0.261606 9.260448 3.912148
C -0.991064 9.599528 1.556691
H 1.950317 -3.637134 1.969227
H 4.365427 -3.873592 1.445490
H 5.595782 -1.967191 0.393816
H 0.774047 -1.493185 1.442787
H 5.694091 2.624947 -1.312190
H 0.814628 3.090599 -0.351271
H 5.902777 3.933649 -3.797394
H 9.657549 5.647773 -2.610989
H 6.055352 6.589065 -0.476193
H 8.161774 16.015359 -0.838527
H 6.925059 16.080053 1.313047
H 5.425713 14.177662 1.935803
H 7.897910 14.046765 -2.358987
H 3.465875 9.794153 0.962226
H 5.886229 9.676526 -3.405918
H 0.942853 8.758286 0.223510
H 0.704816 6.567334 3.910201
H 3.830306 5.753366 1.084779
H -1.953856 7.033371 1.847331
H -2.633480 8.101745 3.097066
H -1.503939 6.810565 3.543782
H -1.137885 9.832335 4.241024
H 0.579471 9.952471 3.795598
H -0.006174 8.550480 4.704930
H -1.250645 9.146223 0.594073
H -0.204195 10.342831 1.388322
H -1.876738 10.127519 1.926920
H 3.549710 3.645238 1.863215
H 1.926030 3.299773 2.461896
H 3.347750 2.803203 3.406949
H 1.074252 4.434443 4.600162
H 2.076600 5.656925 5.418387
H 2.566990 3.956649 5.419413
H 4.994395 5.412887 3.030899
H 4.788188 4.561622 4.569328
H 4.367132 6.285876 4.438211
H 8.282710 2.599087 -6.090381
H 6.943239 3.721713 -5.806176
H 7.187825 2.331636 -4.726383
H 9.888776 4.442023 -6.148404
H 10.080406 5.563465 -4.791097
H 8.631156 5.650729 -5.801857
H 10.221187 2.459659 -4.572519
H 9.179538 2.281038 -3.141915
H 10.401010 3.561405 -3.194587
H 10.383952 7.645012 -1.820655
H 10.712998 6.270138 -0.739652
H 10.713262 7.922317 -0.105208
H 8.194866 8.982373 -1.560067
H 8.672826 9.248294 0.131170
H 7.075897 8.588754 -0.253338
H 8.982239 5.598483 1.032628
H 7.539672 6.596175 1.277481
H 9.136690 7.256819 1.660485
I 1.728382 6.616147 -4.120901

[1-CN]

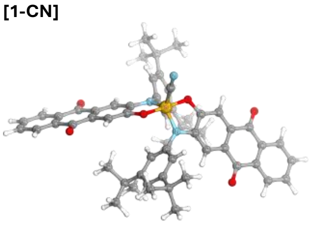

121
xyz, charge: -1, multiplicity: 1
C -0.867208 3.946599 1.581435
C -1.125678 2.948585 2.521868
C 0.346170 3.969082 0.906031
C 1.310149 2.993605 1.162982
C 1.050680 1.991226 2.108303
C -0.170067 1.975433 2.783920
H -1.615710 4.707476 1.376736
H -2.075172 2.932309 3.050338
H 0.575706 4.734155 0.170697
H -0.343256 1.187326 3.510316
C 2.609172 3.040878 0.427947
C 3.583940 1.978723 0.699674
C 3.338154 0.988749 1.678568

C 2.061416 0.932567 2.404004
C 4.811480 2.007447 0.021173
C 5.766911 1.055277 0.313108
C 5.514562 0.041927 1.281788
C 4.310094 0.020207 1.968028
H 4.102389 -0.726237 2.727563
H 5.010452 2.788903 -0.704060
O 2.831646 3.954351 -0.366458
O 1.810046 0.057534 3.231210
O 6.977984 0.997112 -0.228980
N 6.604688 -0.789917 1.425058
C 6.616177 -1.739587 2.488691
C 5.897832 -2.928549 2.368225
C 5.888297 -3.846937 3.412825
C 6.619712 -3.544818 4.571438
C 7.342666 -2.362681 4.711086
C 7.324610 -1.455959 3.645893
H 7.873968 -0.519351 3.699258
H 6.617707 -4.260962 5.385199
H 5.361475 -3.106729 1.442868
C 5.114888 -5.165960 3.337660
C 8.132449 -2.014552 5.975528
C 9.615843 -1.813220 5.614745
H 10.037505 -2.720984 5.170626
H 10.193915 -1.566466 6.513641
H 9.752991 -1.000896 4.894757
C 7.572114 -0.710093 6.574691
H 7.673546 0.127247 5.877525
H 8.116442 -0.450707 7.490624
H 6.509613 -0.818737 6.819066
C 8.040145 -3.111964 7.042121
H 8.451627 -4.060793 6.680511
H 7.006384 -3.278944 7.365762
H 8.620862 -2.812532 7.921351
C 6.096467 -6.343339 3.490745
H 6.852793 -6.319184 2.699030
H 5.557643 -7.296633 3.427592
H 6.617479 -6.313307 4.452681
C 4.373488 -5.329516 2.005657
H 5.064761 -5.332935 1.156149
H 3.639123 -4.531492 1.851954
H 3.835536 -6.283955 2.002720
C 4.075517 -5.211849 4.474078
H 4.550529 -5.156522 5.458513
H 3.503692 -6.146675 4.426922
H 3.375987 -4.373151 4.390198
H 11.049756 4.232914 -1.709455
H 6.477984 6.552615 2.449098
H 5.169546 4.417621 1.894604
H 8.897408 6.973431 -0.261892
H 10.619511 6.942382 1.648914
H 7.126939 2.942283 4.759319
C 13.239860 0.559710 3.971748
C 11.008066 -2.034461 2.332620
C 10.092921 -1.206766 1.714475
C 9.013773 2.245526 1.289452
C 10.208327 0.209583 1.812289
C 11.228710 0.774499 2.561776
C 12.156713 -0.062612 3.197797
C 14.197012 -0.346178 4.673885
C 15.231439 0.208603 5.428297
C 14.071782 -1.739631 4.582693
C 14.981930 -2.561396 5.248444
C 9.820347 3.129728 0.574358
C 12.053010 -1.467437 3.077524
C 12.971526 -2.363383 3.788713
C 16.134568 -0.615439 6.087214
C 16.009719 -2.002337 5.996836
C 7.869458 4.093517 2.352588
C 7.130295 4.036433 4.741456
C 8.055150 2.718880 2.171678
C 10.523840 5.519745 -0.019454
C 5.422518 4.057462 2.897254
C 6.736010 6.109364 3.417241
C 11.296738 6.387718 0.992014
C 11.541895 4.845544 -0.946576
C 9.612238 6.418057 -0.876896
C 6.802726 4.579784 3.337057
C 8.683953 4.960777 1.628721
C 9.666457 4.502529 0.738057
N 9.186249 0.838368 1.133984
O 12.856850 -3.588463 3.756300
O 9.038868 -1.617647 1.018300
O 13.373854 1.779487 4.056926
H 6.383860 4.385211 5.465109
H 16.717098 -2.646437 6.512432
H 7.684055 6.540567 3.758653
H 9.041955 5.816303 -1.592496
H 11.940725 5.764250 1.621589
H 5.389666 2.964057 2.871681
H 14.859058 -3.636624 5.161772
H 16.938659 -0.179020 6.673778
H 8.558715 6.030145 1.754547
H 10.910583 -3.112899 2.271864
H 7.444257 1.996766 2.707610
H 10.553705 2.711799 -0.106228
H 15.303693 1.290623 5.480864
H 11.323751 1.849661 2.672424
H 12.129604 5.613112 -1.462111
H 12.238059 4.210624 -0.388003
H 5.959692 6.401223 4.132834
H 11.926112 7.114664 0.464247
H 4.648429 4.400555 3.594464
H 10.213782 7.145164 -1.435962
H 8.119164 4.375818 5.069172
Si 8.003174 -0.298869 0.427313
C 8.220632 -0.963245 -1.324550
N 8.352735 -1.371866 -2.400724

[1-CN_2]

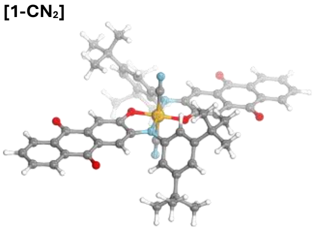

123
xyz, charge: -2, multiplicity: 1
C -1.754856 2.788449 0.801330
C -2.067402 1.498496 1.229909
C -0.440488 3.239390 0.849546
C 0.573499 2.406530 1.325092
C 0.258582 1.110227 1.755426
C -1.061790 0.663225 1.704286
H -2.539393 3.443200 0.429005
H -3.095153 1.145251 1.192959
H -0.164912 4.238323 0.524781
H -1.270188 -0.347239 2.043321
C 1.979284 2.919126 1.375241
C 3.006979 2.015767 1.867377
C 2.693980 0.706894 2.319944
C 1.324867 0.189624 2.260779
C 4.338278 2.464699 1.929614
C 5.332285 1.635340 2.411002
C 5.027390 0.302590 2.850984
C 3.699533 -0.132993 2.807661
H 3.423468 -1.121320 3.158033
H 4.582023 3.466872 1.594458
O 2.210179 4.076947 1.003590
O 1.020708 -0.955588 2.603383
O 6.600985 1.995543 2.518448
N 6.122744 -0.346666 3.315696
C 6.069118 -1.691171 3.722034
C 5.539500 -2.673812 2.875107
C 5.515029 -4.005713 3.267219
C 6.054392 -4.347170 4.517373
C 6.603599 -3.387645 5.366276
C 6.588919 -2.052278 4.960341
H 7.000962 -1.267087 5.585231
H 6.050653 -5.388604 4.820471
H 5.180590 -2.360707 1.901681
C 4.940605 -5.107170 2.370533
C 7.244405 -3.736544 6.712308
C 8.733302 -3.342033 6.668410
H 9.254706 -3.891855 5.877452
H 9.218767 -3.569009 7.626054
H 8.859720 -2.274815 6.464306
C 6.541470 -2.953478 7.837222
H 6.628118 -1.873296 7.690060
H 6.990760 -3.199504 8.807946
H 5.474994 -3.204042 7.872759
C 7.149366 -5.231864 7.038585
H 7.671544 -5.840856 6.292111
H 6.107061 -5.567800 7.093983
H 7.617649 -5.423266 8.011197
C 6.045940 -6.127475 2.038385
H 6.878469 -5.633717 1.526036
H 5.653169 -6.917912 1.385441
H 6.442626 -6.599352 2.943080
C 4.387137 -4.553081 1.052187
H 5.167970 -4.061708 0.462407
H 3.582145 -3.830701 1.226142
H 3.979062 -5.375757 0.452925
C 3.790812 -5.819339 3.108610
H 4.134479 -6.280052 4.040395
H 3.362569 -6.608139 2.476361
H 2.997876 -5.105712 3.357726
H 10.961724 2.865214 -1.435748
H 7.536790 7.754983 1.333314
H 5.936946 5.881482 1.973018
H 9.442997 6.317760 -1.414982
H 11.478930 6.904830 0.049171
H 8.042700 5.673674 5.101276
C 14.111069 0.996526 3.346616
C 11.064906 -1.168202 3.980078
C 10.057491 -0.293193 3.623739
C 9.316700 3.046561 2.349057
C 10.364778 1.031300 3.161061
C 11.703763 1.429336 3.101409
C 12.723037 0.542968 3.461886
C 15.187570 0.041138 3.756886
C 16.521504 0.442419 3.683194
C 14.870945 -1.249795 4.201864
C 15.896542 -2.122416 4.568787
C 10.061611 3.300772 1.189831
C 12.411722 -0.771704 3.898585
C 13.449057 -1.707976 4.300609
C 17.538525 -0.431877 4.050957
C 17.224296 -1.716634 4.493699
C 8.598966 5.359418 2.402715
C 8.167898 6.603394 4.539173
C 8.599362 4.075313 2.949838
C 10.910380 4.894308 -0.619881
C 6.264117 6.000062 3.011611
C 7.867553 7.800798 2.376992
C 11.953767 5.981233 -0.297465
C 11.654633 3.666365 -1.158170
C 9.962742 5.404522 -1.722079
C 7.740382 6.438076 3.068538
C 9.362531 5.596253 1.260898
C 10.102672 4.576349 0.642479
N 9.248617 1.750394 2.888559
O 13.213221 -2.849345 4.717346
O 8.769567 -0.588469 3.694417
O 14.423289 2.114814 2.930267
H 7.560145 7.377123 5.025680
H 18.017951 -2.402514 4.780927
H 8.899658 8.170413 2.398805
H 9.204046 4.648902 -1.951942
H 12.633302 5.635741 0.489264
H 6.110099 5.041481 3.515539
H 15.618940 -3.115059 4.910647
H 18.576641 -0.113603 3.992386
H 9.380531 6.590374 0.827429
H 10.820163 -2.167681 4.322518
H 8.025036 3.840074 3.839565
H 10.574541 2.466107 0.726480
H 16.731396 1.448418 3.332026
H 11.976155 2.430249 2.785578
H 12.222407 3.946968 -2.053322
H 12.361298 3.269321 -0.421251
H 7.236334 8.532508 2.894725
H 12.547222 6.217455 -1.190446
H 5.622847 6.748912 3.493556
H 10.525719 5.624467 -2.638595
H 9.222183 6.896335 4.603236
Si 7.684260 0.706730 3.115487
C 7.882933 0.034736 1.271587
C 7.485011 1.379206 4.959150
N 7.999260 -0.359154 0.184608
N 7.366367 1.778144 6.044546

[1-N$_3$]

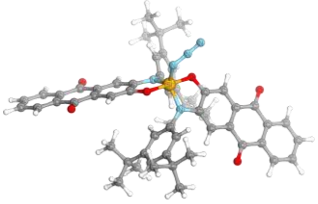

122
xyz, charge: -1, multiplicity: 1

C -0.852382 3.943076 1.552263
C -1.115674 2.939121 2.484967
C 0.364384 3.969676 0.883038
C 1.326991 2.992437 1.138367
C 1.062650 1.984094 2.075917
C -0.161429 1.964107 2.745342
H -1.599723 4.705462 1.348789
H -2.067725 2.919668 3.008720
H 0.597851 4.739444 0.153862
H -0.338183 1.171318 3.465772
C 2.630040 3.044584 0.410359
C 3.602387 1.980510 0.679129
C 3.352373 0.984584 1.651322
C 2.071808 0.923179 2.369376
C 4.833505 2.012609 0.006785
C 5.788277 1.060114 0.299290
C 5.532264 0.040378 1.260679
C 4.323035 0.015055 1.940188
H 4.111259 -0.735710 2.694281
H 5.035963 2.797928 -0.713366
O 2.856244 3.964461 -0.376020
O 1.815360 0.042098 3.188747
O 7.002926 1.008998 -0.234161
N 6.620585 -0.790372 1.404759
C 6.629302 -1.734993 2.472516
C 5.910911 -2.924580 2.357773
C 5.896739 -3.836258 3.408326
C 6.624034 -3.527443 4.567596
C 7.347100 -2.344577 4.701925
C 7.333274 -1.444792 3.631074
H 7.883630 -0.508577 3.679995
H 6.618353 -4.238066 5.386183
H 5.376764 -3.107997 1.432159
C 5.120956 -5.154344 3.339710
C 8.132525 -1.988712 5.966976
C 9.617720 -1.792770 5.610776
H 10.039608 -2.704537 5.175167
H 10.192533 -1.540188 6.510167
H 9.759242 -0.986356 4.885039
C 7.572498 -0.678811 6.554459
H 7.678978 0.153232 5.851760
H 8.113512 -0.414102 7.470856
H 6.508672 -0.783243 6.794903
C 8.033763 -3.077992 7.041344
H 8.445311 -4.030146 6.688628
H 6.998296 -3.240926 7.361638
H 8.611154 -2.772927 7.920852
C 6.098794 -6.332892 3.506872
H 6.859360 -6.316555 2.718986
H 5.558126 -7.285510 3.448529
H 6.615084 -6.296443 4.471096
C 4.385510 -5.326945 2.005521
H 5.081116 -5.337838 1.159560
H 3.653426 -4.528744 1.842232
H 3.845890 -6.280492 2.007239
C 4.075815 -5.189133 4.471225
H 4.546362 -5.126867 5.457374
H 3.502014 -6.123039 4.428835
H 3.378764 -4.349431 4.377397
H 11.058343 4.246576 -1.697426
H 6.481112 6.538464 2.469786
H 5.175143 4.406344 1.900517
H 8.902754 6.976948 -0.236625
H 10.622679 6.935874 1.675830
H 7.128703 2.914754 4.759029
C 13.257669 0.551902 3.957662
C 11.012348 -2.039291 2.333126
C 10.099627 -1.211168 1.710956
C 9.023552 2.239094 1.289831
C 10.220732 0.206066 1.804353
C 11.245577 0.769214 2.550062
C 12.171010 -0.068815 3.188616
C 14.210539 -0.355257 4.664633
C 15.247085 0.198113 5.417075
C 14.078974 -1.748476 4.579546
C 14.985070 -2.571285 5.249500
C 9.829303 3.128970 0.580507
C 12.060951 -1.474190 3.074611
C 12.976394 -2.370744 3.787105
C 16.146078 -0.627007 6.080512
C 16.014916 -2.013651 5.996291
C 7.874757 4.080695 2.360280
C 7.132023 4.008977 4.747738
C 8.063071 2.707384 2.173131
C 10.529897 5.523152 -0.000779
C 5.426992 4.040134 2.901242
C 6.738529 6.089568 3.435491
C 11.301204 6.385903 1.016345
C 11.549557 4.855316 -0.930789
C 9.618649 6.425740 -0.854091
C 6.806248 4.560503 3.346107
C 8.688277 4.953249 1.641467
C 9.672675 4.500810 0.750112
N 9.198712 0.833572 1.128899
O 12.859447 -3.596209 3.757600
O 9.044508 -1.622478 1.022121
O 13.399396 1.771553 4.036210
H 6.384592 4.353380 5.472504
H 16.718835 -2.658723 6.515467
H 7.686050 6.519244 3.780320
H 9.049344 5.827705 -1.573595
H 11.944987 5.759253 1.642968
H 5.395064 2.946895 2.868662
H 14.857235 -3.646306 5.167438
H 16.951652 -0.191572 6.665801
H 8.560876 6.021775 1.772275
H 10.909596 -3.117590 2.278392
H 7.452275 1.981671 2.704231
H 10.565636 2.715773 -0.099825
H 15.324099 1.280048 5.464643
H 11.345031 1.844371 2.656957
H 12.136886 5.626395 -1.441583
H 12.245815 4.217940 -0.375126
H 5.961325 6.376650 4.152094
H 11.930461 7.116607 0.493549
H 4.651747 4.378358 3.599600
H 10.220157 7.156587 -1.408387
H 8.120482 4.346426 5.078706
Si 8.026288 -0.309917 0.399910
N 8.287016 -0.975513 -1.240451
N 7.776818 -0.561327 -2.260491
N 7.356903 -0.256011 -3.272764

[1-(N$_3$)$_2$]

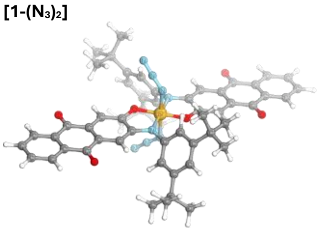

125
xyz, charge: -2, multiplicity: 1

C -1.695486 2.852160 1.111898
C -1.991479 1.520835 1.404919
C -0.388992 3.315189 1.218279
C 0.634108 2.453612 1.617272
C 0.335860 1.115910 1.911361
C -0.977096 0.656917 1.802341
H -2.487035 3.529579 0.799871
H -3.013272 1.158168 1.322321
H -0.126169 4.345741 0.998770
H -1.172534 -0.385593 2.035550
C 2.031109 2.980313 1.732361
C 3.068910 2.046823 2.140701
C 2.771563 0.694740 2.457996
C 1.411748 0.164926 2.331751
C 4.391549 2.506227 2.263979
C 5.392721 1.645120 2.666248
C 5.107013 0.270214 2.965586
C 3.784714 -0.175577 2.868387
H 3.517702 -1.196455 3.117507
H 4.624452 3.540513 2.036158
O 2.245801 4.172794 1.482416
O 1.123407 -1.014140 2.552547
O 6.657721 2.009418 2.819723
N 6.208403 -0.406874 3.370624
C 6.162182 -1.781818 3.654004
C 5.622601 -2.685612 2.727948
C 5.584682 -4.045933 3.003974
C 6.126436 -4.499803 4.216659
C 6.686672 -3.620885 5.142282
C 6.680612 -2.254925 4.855199
H 7.098887 -1.528255 5.542727
H 6.112969 -5.563294 4.429367
H 5.257985 -2.287850 1.788176
C 4.983100 -5.059184 2.024678
C 7.324980 -4.090544 6.452536
C 8.810792 -3.683029 6.454524
H 9.339979 -4.151920 5.618000
H 9.294603 -3.995436 7.388587
H 8.930276 -2.600675 6.351493
C 6.610053 -3.422335 7.642031
H 6.684721 -2.332438 7.592555
H 7.059031 -3.750667 8.588276
H 5.546280 -3.686366 7.650097
C 7.240570 -5.610485 6.637323
H 7.765501 -6.142657 5.836104
H 6.200948 -5.958032 6.662448
H 7.712005 -5.888969 7.587093
C 6.061303 -6.076880 1.606668
H 6.904632 -5.565124 1.130706
H 5.646772 -6.802952 0.895106
H 6.449274 -6.629274 2.468465
C 4.437188 -4.390611 0.757207
H 5.226577 -3.870763 0.204201
H 3.648111 -3.667685 0.991017
H 4.010022 -5.154247 0.096374
C 3.818813 -5.799385 2.710804
H 4.155113 -6.338399 3.602360
H 3.369236 -6.526409 2.021803
H 3.044511 -5.088276 3.018566
H 10.507696 2.185111 -1.260358
H 7.571731 7.615845 1.045217
H 6.000072 5.898938 2.084371
H 8.942026 5.570199 -1.639435
H 11.171014 6.422843 -0.657974
H 8.358363 6.062868 5.012994
C 14.123042 1.149764 3.844028
C 11.117140 -1.128821 4.240435
C 10.098160 -0.262977 3.896577
C 9.335077 3.035580 2.572954
C 10.384514 1.097925 3.538436
C 11.713942 1.530687 3.525784
C 12.744534 0.654445 3.877875
C 15.209663 0.198474 4.237984
C 16.534368 0.635540 4.232664
C 14.911731 -1.121679 4.603238
C 15.947344 -1.987292 4.959850
C 9.970243 3.089161 1.326899
C 12.453717 -0.688917 4.240013
C 13.500486 -1.619548 4.626413
C 17.561055 -0.231994 4.588985
C 17.265576 -1.545896 4.952683
C 8.673397 5.351162 2.346266
C 8.471442 6.896187 4.313602
C 8.703021 4.162168 3.079159
C 10.555323 4.324064 -0.830710
C 6.422258 6.160091 3.060780
C 8.008244 7.790628 2.034961
C 11.607702 5.446555 -0.892494
C 11.229973 3.007939 -1.235675
C 9.427802 4.609848 -1.841644
C 7.908317 6.544827 2.923364
C 9.302308 5.381509 1.103673
C 9.952529 4.255171 0.575076
N 9.264247 1.807239 3.268325
O 13.281760 -2.789189 4.970905
O 8.821179 -0.603790 3.860615
O 14.419747 2.298423 3.508255
H 7.937349 7.759992 4.730104
H 18.066816 -2.226518 5.231375
H 9.048914 8.110764 1.904270
H 8.665609 3.825487 -1.784292
H 12.413248 5.256798 -0.174262

H	6.292442	5.298284	3.722422
H	15.684301	-3.003182	5.239278
H	18.591888	0.114078	4.583402
H	9.273124	6.290693	0.512844
H	10.894767	-2.158828	4.497101
H	8.201709	4.085509	4.038095
H	10.422748	2.177543	0.953645
H	16.729283	1.663929	3.943013
H	11.963101	2.551055	3.253728
H	11.658499	3.112014	-2.239745
H	12.040098	2.741516	-0.547661
H	7.456410	8.615570	2.500689
H	12.044656	5.503755	-1.898034
H	5.847059	6.998327	3.474406
H	9.830451	4.639004	-2.862897
H	9.537281	7.143913	4.247204
Si	7.738758	0.696758	3.339334
N	8.076008	0.154267	1.510182
N	7.452142	1.118449	5.213503
N	7.773452	0.757466	0.518209
N	7.225764	2.217269	5.631952
N	7.524368	1.274215	-0.481169
N	7.015088	3.253221	6.092591

[1-NCS]

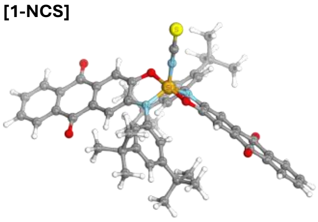

122
xyz, charge: -1, multiplicity: 1

C	-1.825208	1.934353	-2.214211
C	-2.126557	0.583216	-2.389730
C	-0.844473	2.316742	-1.308202
C	-0.157860	1.352619	-0.569254
C	-0.460177	-0.005134	-0.746115
C	-1.446448	-0.381912	-1.658320
H	-2.358050	2.688901	-2.786765
H	-2.893714	0.285010	-3.099350
H	-0.588071	3.360036	-1.151629
H	-1.659611	-1.440167	-1.774096
C	0.894363	1.787367	0.396320
C	1.582144	0.746394	1.167404
C	1.302903	-0.625180	0.965560
C	0.256056	-1.060771	0.030238
C	2.584019	1.133266	2.070857
C	3.285648	0.163017	2.758088
C	3.000548	-1.219116	2.565574
C	2.016444	-1.606305	1.668171
H	2.815781	2.184188	2.206007
H	1.792778	-2.652482	1.486857
O	1.163648	2.982931	0.514541
O	-0.036538	-2.244776	-0.128022
O	4.285818	0.399497	3.597190
N	3.847754	-2.006385	3.314834
C	3.862923	-3.416594	3.103135
C	4.915855	-3.981357	2.382221
C	4.980310	-5.355081	2.187472
C	3.937382	-6.143232	2.697953
C	2.859599	-5.595870	3.389657
C	2.842469	-4.211166	3.598353
H	2.040552	-3.733703	4.154421
H	3.979253	-7.215603	2.545608
H	5.689098	-3.321613	2.003595
C	6.148391	-6.021209	1.455805
C	7.160988	-4.997599	0.927486
H	6.703172	-4.306340	0.210812
H	7.974155	-5.521840	0.413505
H	7.607423	-4.412558	1.738392
C	6.881750	-6.961893	2.431146
H	7.271570	-6.406667	3.290261
H	7.728195	-7.443926	1.926992
H	6.219536	-7.748332	2.807966
C	5.612989	-6.832483	0.260757
H	5.072277	-6.183018	-0.436490
H	4.930307	-7.626252	0.580374
H	6.445005	-7.301991	-0.277578
C	1.707028	-6.444565	3.932194
C	0.386258	-5.961510	3.302778
H	0.182330	-4.914033	3.543637
H	-0.452950	-6.563004	3.672901
H	0.422108	-6.049487	2.211620
C	1.634069	-6.288527	5.462952
H	1.459138	-5.248985	5.755164
H	2.570552	-6.613520	5.928841
H	0.815022	-6.895652	5.867468
C	1.874164	-7.934364	3.610363
H	1.908093	-8.112810	2.529877
H	1.021511	-8.491628	4.013932
H	2.784277	-8.346555	4.060001
H	7.471543	4.696101	3.152225
H	3.889185	2.541551	-0.238643
H	5.177740	3.461426	-1.034372
C	4.589358	2.539096	-1.080132
H	8.427568	3.601813	4.159720
C	8.468416	4.263682	3.289741
H	4.004211	2.555833	-2.007654
H	9.171648	5.074767	3.514858
N	6.630575	-0.681284	3.394076
C	6.834409	0.344723	2.424746
C	7.585170	-1.662300	3.551805
C	7.718250	1.380965	2.675351
C	7.108796	-2.705621	4.396358
O	5.874273	-2.519634	4.845063
C	6.136059	0.283624	1.218116
C	8.840086	-1.759304	2.969152
C	7.886632	-3.815830	4.657735
C	9.633065	-2.885621	3.229516
C	9.160380	-3.914587	4.076754
C	10.964075	-2.958737	2.610166
C	7.915239	2.388256	1.722559
H	8.059608	5.020084	0.685092
C	6.283012	1.284927	0.266887
C	7.182513	2.326989	0.539786
C	11.775493	-4.187538	2.858409
C	13.035698	-4.301471	2.270508
C	9.945561	-5.130796	4.312576
C	11.291071	-5.221406	3.672407
O	11.415157	-2.056468	1.906698
C	13.808310	-5.434669	2.489226
C	12.072362	-6.357425	3.886988
H	8.242393	1.393378	3.626860
C	13.325936	-6.463896	3.298713
O	9.528569	-6.062724	5.000721
C	5.489705	1.289176	-1.042343
H	5.460858	-0.550227	1.059079
C	8.912626	3.510215	2.021647
H	9.219328	-0.989015	2.305682
H	7.513983	-4.617146	5.286563
H	13.384882	-3.485176	1.645615
H	14.789130	-5.519265	2.028983
H	7.304938	3.109529	-0.200163
H	11.670821	-7.142516	4.520331
H	13.931283	-7.350140	3.469550
H	3.852049	-0.005304	-0.380098
C	9.017198	4.522867	0.875177
H	7.090841	2.215922	-2.227273
C	4.594950	0.051583	-1.182588
C	6.463792	1.318681	-2.235367
H	9.747371	5.296103	1.138567
H	4.050764	0.101692	-2.132105
C	10.307185	2.896412	2.250539
H	10.309847	2.201962	3.095843
H	5.903705	1.309043	-3.178099
H	9.353587	4.048520	-0.053413
H	11.039728	3.685646	2.459252
H	5.180374	-0.874857	-1.181304
H	7.124763	0.445106	-2.216628
H	10.636568	2.341991	1.365200
Si	5.081951	-1.065320	4.207980
N	4.768954	-0.532716	5.860148
C	4.609673	-0.002082	6.906629
S	4.406518	0.682224	8.333194

[1-(NCS)₂]

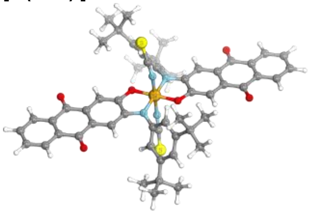

125
xyz, charge: -2, multiplicity: 1

C	-1.788888	2.807484	1.026864
C	-2.106468	1.534623	1.501432
C	-0.470729	3.248036	1.043119
C	0.542341	2.422056	1.532546
C	0.222509	1.143433	2.009294
C	-1.102395	0.706830	1.990328
H	-2.572851	3.456464	0.643602
H	-3.137481	1.189440	1.488947
H	-0.191211	4.233270	0.681977
H	-1.315110	-0.289771	2.365698
C	1.953073	2.922660	1.543965
C	2.979652	2.027942	2.056602
C	2.663044	0.734035	2.548908
C	1.286056	0.231618	2.535198
C	4.315687	2.465972	2.077187
C	5.307186	1.640412	2.566504
C	4.996329	0.332601	3.064078
C	3.668467	-0.100600	3.045316
H	3.395423	-1.081448	3.419372
H	4.563438	3.450992	1.696894
O	2.190698	4.061828	1.124757
O	0.975666	-0.893415	2.931757
O	6.589111	1.973364	2.616696
N	6.094082	-0.310116	3.535719
C	6.062584	-1.689794	3.823849
C	5.603761	-2.595550	2.860226
C	5.631641	-3.961628	3.105181
C	6.138446	-4.409678	4.335049
C	6.604212	-3.524957	5.306111
C	6.547246	-2.154949	5.039766
H	6.913588	-1.422664	5.751729
H	6.182015	-5.476952	4.522060
H	5.268787	-2.198229	1.908959
C	5.185121	-4.981522	2.053367
C	7.204109	-3.993366	6.634882
C	8.675122	-3.540246	6.704013
H	9.252706	-3.981515	5.884748
H	9.129082	-3.850551	7.653662
H	8.765393	-2.453339	6.620329
C	6.419996	-3.368573	7.804141
H	6.461032	-2.276131	7.777077
H	6.839814	-3.698980	8.762876
H	5.365884	-3.665869	7.763697
C	7.162300	-5.518036	6.794533
H	7.743742	-6.020532	6.013669
H	6.134980	-5.899603	6.762966
H	7.595949	-5.794883	7.762607
C	6.412141	-5.793114	1.595350
H	7.164927	-5.125535	1.162643
H	6.120590	-6.529889	0.835328
H	6.873114	-6.327954	2.432741
C	4.568517	-4.308872	0.820633
H	5.292906	-3.668049	0.306673
H	3.696581	-3.701805	1.089658
H	4.239432	-5.079162	0.112805
C	4.133917	-5.930740	2.658656
H	4.531927	-6.492392	3.510012
H	3.800527	-6.654704	1.904107
H	3.261531	-5.365390	3.004834
H	10.762661	2.536624	-1.474545
H	7.588190	7.681037	1.138352
H	5.962335	5.905733	1.997843
H	9.365472	6.038803	-1.594598
H	11.468915	6.629015	-0.231398
H	8.232096	5.828872	5.006477
C	14.053720	0.972810	3.427826
C	10.999627	-1.125062	4.231177
C	9.997255	-0.267169	3.828099
C	9.264284	2.987752	2.353763
C	10.307422	1.021827	3.280079
C	11.646741	1.408120	3.180547

C 12.662965 0.538793 3.587770
C 15.127157 0.038421 3.890065
C 16.463127 0.423878 3.776512
C 14.806510 -1.217410 4.423697
C 15.829722 -2.071367 4.837952
C 9.972138 3.148469 1.156174
C 12.347779 -0.742516 4.111766
C 13.382785 -1.657727 4.566495
C 17.477388 -0.431437 4.191735
C 17.159015 -1.681546 4.722666
C 8.629212 5.324093 2.301558
C 8.367461 6.711568 4.374547
C 8.608219 4.073100 2.921279
C 10.808844 4.607636 -0.770006
C 6.355760 6.087705 3.003908
C 7.977425 7.782158 2.157906
C 11.899789 5.671261 -0.540515
C 11.491407 3.326173 -1.263201
C 9.843518 5.092728 -1.868029
C 7.846892 6.472283 2.944515
C 9.352132 5.466579 1.118579
C 10.034289 4.388507 0.533452
N 9.189386 1.722883 2.971134
O 13.142383 -2.765781 5.061448
O 8.704930 -0.543164 3.924604
O 14.368249 2.057807 2.934076
H 7.822423 7.542710 4.840153
H 17.950944 -2.352566 5.047062
H 9.019816 8.117616 2.100928
H 9.052378 4.353942 -2.034586
H 12.592116 5.344637 0.243088
H 6.198409 5.179127 3.592422
H 15.549145 -3.036971 5.247988
H 18.516982 -0.125763 4.101785
H 9.386435 6.433304 0.627993
H 10.753184 -2.099191 4.639062
H 8.064879 3.918843 3.846885
H 10.446606 2.272611 0.728694
H 16.676515 1.402541 3.356927
H 11.921121 2.384137 2.795769
H 12.037577 3.536793 -2.190280
H 12.209047 2.944427 -0.528868
H 7.398916 8.566557 2.659500
H 12.471581 5.836552 -1.462982
H 5.772236 6.894768 3.464279
H 10.383673 5.244152 -2.811638
H 9.435065 6.959852 4.359425
Si 7.641898 0.713417 3.276810
N 7.811082 -0.066518 1.537666
N 7.460977 1.476129 5.012505
C 7.741249 -0.805154 0.622456
C 7.311689 2.290102 5.852143
S 7.653722 -1.800972 -0.652401
S 7.120009 3.404748 7.009077

1-MeCN

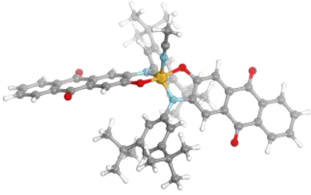

125
xyz, charge: 0, multiplicity: 1
C 2.312988 -2.970022 0.994793
C 3.695710 -3.091052 0.848833
C 4.436161 -2.037886 0.329485
C 3.800524 -0.854085 -0.047735
C 2.409608 -0.732567 0.098500
C 1.672801 -1.796101 0.621380
C 4.615572 0.260214 -0.605554
C 3.910514 1.505831 -0.973608
C 2.511367 1.616182 -0.848817
C 1.701058 0.517679 -0.292293
C 4.671235 2.574946 -1.463584
C 4.026282 3.744950 -1.827742
C 2.621636 3.856723 -1.673954
C 1.864734 2.809211 -1.201428
O 0.486224 0.621269 -0.146026
O 5.827829 0.138675 -0.750145
O 2.131645 5.069015 -1.996631
N 4.569120 4.929493 -2.310165
C 5.984214 5.114035 -2.364271
C 6.755328 4.353996 -3.243482
C 8.135276 4.524538 -3.283439
C 8.710915 5.487298 -2.440005
C 7.956813 6.258917 -1.559730
C 6.574068 6.047369 -1.526434
C 7.214660 15.212433 -1.499462
C 6.484478 15.301531 -0.313402
C 5.661502 14.254434 0.078287
C 5.562246 13.108509 -0.712079
C 6.296324 13.019133 -1.905392
C 7.121037 14.076396 -2.291886
C 4.670615 11.999870 -0.272749
C 4.609660 10.792945 -1.123537
C 5.328202 10.716132 -2.333201
C 6.214888 11.809557 -2.770302
C 3.806311 9.726520 -0.700141
C 3.726167 8.592778 -1.491133
C 4.473153 8.513484 -2.693179
C 5.257993 9.559218 -3.122017
O 6.872213 11.733643 -3.804802
O 4.015290 12.094333 0.760141
O 4.361652 7.331132 -3.328228
N 3.022339 7.416165 -1.265302
C 2.354369 7.193944 -0.022649
C 1.245693 7.966171 0.323338
C 0.606616 7.758402 1.541265
C 1.093716 6.747115 2.383718
C 2.194995 5.962060 2.052420
C 2.830478 6.211364 0.831017
Si 3.362424 6.193838 -2.479218
C 8.575627 7.298238 -0.621911
C 7.946475 8.677023 -0.897841
C 8.289244 6.891238 0.836701
C 10.093380 7.415009 -0.801862
C 9.035524 3.704324 -4.210284
C 9.775554 4.651979 -5.173235
C 10.061391 2.927642 -3.362565
C 8.242297 2.691448 -5.044216
C 2.744949 4.867546 2.970052
C 2.735288 3.519960 2.223676
C 4.192811 5.221836 3.361580
C 1.920695 4.714555 4.253238
C -0.597473 8.591049 1.987023
C -1.805180 7.663041 2.217745
C -0.248873 9.318973 3.299612
C -0.992246 9.645986 0.946891
H 1.735856 -3.795672 1.401451
H 4.194161 -4.010547 1.142333
H 5.512390 -2.106900 0.205712
H 0.598881 -1.676618 0.725399
H 5.747905 2.466295 -1.539173
H 0.792486 2.903207 -1.067193
H 6.253005 3.636481 -3.882859
H 9.784729 5.628649 -2.476303
H 5.942659 6.612768 -0.845187
H 7.857879 16.033291 -1.803732
H 6.560024 16.191393 0.305064
H 5.083281 14.299294 0.995871
H 7.678873 13.982154 -3.218348
H 3.272377 9.808397 0.240614
H 5.843183 9.489174 -4.032654
H 0.904909 8.722332 -0.375266
H 0.592123 6.577672 3.329285
H 3.704734 5.637467 0.533034
H -2.060628 7.124215 1.298937
H -2.677643 8.249297 2.528911
H -1.604599 6.921529 2.996960
H -1.096730 9.931285 3.628112
H 0.616931 9.975017 3.159566
H -0.012388 8.614301 4.102659
H -1.280733 9.187645 -0.005578
H -0.179013 10.356281 0.761394
H -1.852214 10.215402 1.314733
H 3.367615 3.544174 1.330888
H 1.721116 3.254148 1.906874
H 3.112214 2.724709 2.877359
H 0.882120 4.441410 4.036257
H 1.923988 5.632278 4.851875
H 2.351361 3.917150 4.867681
H 4.845627 5.277284 2.484627
H 4.599669 4.456416 4.032351
H 4.231851 6.188821 3.874720
H 8.930532 2.126180 -5.681297
H 7.515433 3.185480 -5.698484
H 7.710947 1.973044 -4.410245
H 10.418903 4.076642 -5.849127
H 10.406975 5.365142 -4.634661
H 9.062857 5.223718 -5.777378
H 10.706609 2.324111 -4.011487
H 9.554581 2.257231 -2.660226
H 10.702158 3.600533 -2.784751
H 10.357371 7.722945 -1.819595
H 10.604180 6.472008 -0.577355
H 10.482427 8.173752 -0.114956
H 8.109040 8.981709 -1.937222
H 8.394190 9.433275 -0.242491
H 6.867467 8.676041 -0.715695
H 8.707513 5.902477 1.054269
H 7.214607 6.857131 1.042388
H 8.737686 7.616742 1.525143
N 2.320490 6.237779 -4.148314
C 1.706699 6.260347 -5.118803
C 0.939014 6.288595 -6.335593
H 1.611182 6.177890 -7.191666
H 0.415448 7.246228 -6.414077
H 0.209850 5.473306 -6.334438

1-Et_2O

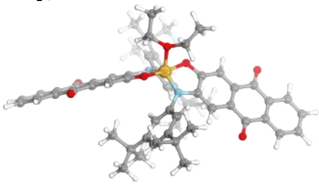

134
xyz, charge: 0, multiplicity: 1
C 2.396558 -2.970136 1.162697
C 3.786898 -3.089152 1.143478
C 4.564595 -2.082685 0.587539
C 3.958696 -0.947747 0.046900
C 2.560093 -0.828244 0.065837
C 1.785772 -1.844791 0.626331
C 4.813668 0.116460 -0.546807
C 4.137757 1.313781 -1.090392
C 2.733196 1.421420 -1.088438
C 1.882767 0.370573 -0.500451
C 4.933431 2.339604 -1.615731
C 4.317009 3.465867 -2.134800
C 2.904230 3.578353 -2.103976
C 2.113753 2.570020 -1.601588
O 0.659724 0.473970 -0.468218
O 6.033866 -0.003868 -0.578450
O 2.441377 4.749055 -2.582026
N 4.895895 4.604205 -2.682809
C 6.308848 4.805235 -2.594286
C 7.175633 4.028259 -3.360832
C 8.550774 4.213390 -3.256136
C 9.023368 5.205729 -2.383581
C 8.172017 5.996082 -1.615846
C 6.796155 5.769576 -1.727111
C 7.363449 14.947856 -2.043943
C 6.482622 15.088460 -0.970613
C 5.618439 14.054137 -0.638583
C 5.628344 12.869535 -1.376167
C 6.514034 12.728297 -2.456205
C 7.379080 13.773179 -2.783467
C 4.690281 11.775805 -1.002714
C 4.745278 10.527156 -1.792528
C 5.621049 10.395960 -2.888297
C 6.551268 11.476889 -3.262037
C 3.895115 9.476410 -1.425803
C 3.929272 8.302354 -2.159125
C 4.832621 8.167687 -3.241741
C 5.662025 9.199383 -3.618492
O 7.336619 11.356608 -4.198100
O 3.904395 11.913974 -0.071265
O 4.803618 6.955234 -3.825523
N 3.203246 7.133676 -1.974625
C 2.411190 6.965392 -0.795529
C 1.217392 7.669155 -0.647716
C 0.459601 7.517292 0.509565
C 0.924134 6.634446 1.496314

C 2.111842 5.919476 1.363451
C 2.859289 6.108314 0.196136
Si 3.707550 5.841123 -3.062004
C 8.674935 7.071102 -0.649404
C 8.057152 8.431206 -1.026487
C 8.244855 6.696917 0.782425
C 10.201303 7.208980 -0.674907
C 9.553754 3.374914 -4.051574
C 10.415337 4.302153 -4.929112
C 10.458861 2.607839 -3.068137
C 8.863938 2.354079 -4.964037
C 2.631503 4.957179 2.434017
C 2.760955 3.546410 1.827993
C 4.015743 5.434872 2.913129
C 1.700229 4.879027 3.648736
C -0.849875 8.275737 0.737672
C -1.995260 7.266837 0.948390
C -0.708421 9.162793 1.989494
C -1.214235 9.175706 -0.448499
H 1.790402 -3.759423 1.598150
H 4.262115 -3.970595 1.564380
H 5.647532 -2.151462 0.559874
H 0.706590 -1.728205 0.629461
H 6.012994 2.234298 -1.596337
H 1.033782 2.664842 -1.562150
H 6.749994 3.286034 -4.027058
H 10.093765 5.356407 -2.306344
H 6.089152 6.349450 -1.139083
H 8.038476 15.758894 -2.301917
H 6.472892 16.008647 -0.393437
H 4.924049 14.139217 0.191396
H 8.054504 13.638809 -3.622495
H 3.236890 9.601063 -0.572409
H 6.367216 9.089676 -4.435719
H 0.904973 8.329838 -1.449135
H 0.332627 6.509805 2.395578
H 3.797835 5.580348 0.045762
H -2.098966 6.611346 0.077101
H -2.942872 7.797344 1.096935
H -1.823964 6.635938 1.825600
H -1.635477 9.721686 2.162127
H 0.109435 9.880830 1.865910
H -0.502578 8.568460 2.884923
H -1.353171 8.595742 -1.367700
H -0.449614 9.939381 -0.628677
H -2.155706 9.693661 -0.237671
H 3.469578 3.525768 0.993697
H 1.794732 3.191739 1.453725
H 3.117983 2.840553 2.586973
H 0.704196 4.515577 3.372979
H 1.593919 5.850983 4.143378
H 2.116089 4.178769 4.380571
H 4.745859 5.436027 2.097785
H 4.393853 4.768832 3.697110
H 3.959287 6.450794 3.318801
H 9.621712 1.780246 -5.507705
H 8.223340 2.844153 -5.705719
H 8.256836 1.643429 -4.392496
H 11.130802 3.711917 -5.513281
H 10.984143 5.018111 -4.328404
H 9.788044 4.870444 -5.624259
H 11.176146 1.988225 -3.618818
H 9.862540 1.954167 -2.422565
H 11.026233 3.288805 -2.426412
H 10.566353 7.487040 -1.669727
H 10.698288 6.282670 -0.365864
H 10.506374 7.995932 0.022491
H 8.322712 8.714071 -2.050726
H 8.421769 9.211101 -0.347895
H 6.964786 8.416241 -0.958085
H 8.651652 5.720799 1.067832
H 7.155272 6.650329 0.876651
H 8.609868 7.446398 1.494000
O 2.952913 5.734406 -4.824739
C 2.299452 4.497114 -5.284368
C 2.399047 6.992511 -5.376909
C 3.254719 7.553289 -6.487376
H 2.341201 7.687565 -4.533765
H 1.377104 6.763688 -5.697446
H 2.833977 8.517765 -6.792356
H 4.273694 7.718529 -6.133817
H 3.280816 6.900147 -7.361879
C 2.421170 4.316569 -6.779275
H 2.823792 3.698199 -4.757657
H 1.260037 4.516496 -4.940103
H 3.466972 4.351734 -7.097151
H 2.020892 3.328446 -7.029089
H 1.847090 5.054593 -7.345593

1-(Et$_2$O)$_2$

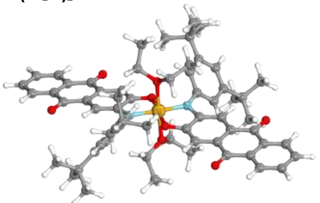

149
xyz, charge: 0, multiplicity: 1
C 3.128318 -3.603837 0.090916
C 4.398035 -3.822346 -0.445394
C 5.211407 -2.745245 -0.769987
C 4.762873 -1.440418 -0.559516
C 3.485133 -1.220529 -0.019796
C 2.673962 -2.309036 0.302611
C 5.650770 -0.302073 -0.921366
C 5.148393 1.065949 -0.664364
C 3.861392 1.280150 -0.132568
C 2.976532 0.159121 0.221096
C 5.983448 2.140524 -0.983557
C 5.529090 3.443573 -0.796571
C 4.245906 3.639470 -0.223551
C 3.417626 2.590054 0.100447
O 1.861067 0.341168 0.702809
O 6.754942 -0.499839 -1.418800
O 3.943226 4.930976 0.015040
N 6.169432 4.646493 -1.010012
C 7.477593 4.675304 -1.569230
C 7.775642 3.919848 -2.710955
C 9.048920 3.938070 -3.266771
C 10.022234 4.757349 -2.676685
C 9.746361 5.536412 -1.556774
C 8.465153 5.469611 -1.002403
C 6.325248 15.626167 -2.728074
C 5.152401 15.860822 -2.009170
C 4.469330 14.801849 -1.426793
C 4.953967 13.499482 -1.556851
C 6.134243 13.263337 -2.280218
C 6.813801 14.333509 -2.863012
C 4.200676 12.379997 -0.928489
C 4.756461 11.016301 -1.071609
C 5.935341 10.784863 -1.807482
C 6.681183 11.885871 -2.435541
C 4.065604 9.961210 -0.468855
C 4.534838 8.658176 -0.615604
C 5.743221 8.448343 -1.329676
C 6.435234 9.478941 -1.920984
O 7.720492 11.691173 -3.061708
O 3.155448 12.589527 -0.320609
O 6.171915 7.169353 -1.324006
N 4.041497 7.477761 -0.101177
C 2.826783 7.460075 0.643721
C 1.691467 8.124890 0.162420
C 0.498890 8.110285 0.875497
C 0.445946 7.378599 2.070281
C 1.556383 6.701733 2.566033
C 2.753854 6.772435 1.848496
Si 5.086105 6.062054 -0.587633
C 10.777808 6.469728 -0.917834
C 10.258451 7.918497 -0.999032
C 10.979710 6.079322 0.558621
C 12.139219 6.405849 -1.619199
C 9.410378 3.099304 -4.494966
C 9.882426 4.027905 -5.629658
C 10.544103 2.124775 -4.121875
C 8.223796 2.274583 -5.008154
C 1.523675 5.894195 3.866048
C 1.943503 4.441044 3.571547
C 2.504629 6.522905 4.873575
C 0.131459 5.868105 4.507092
C -0.746667 8.856609 0.390468
C -1.869454 7.840871 0.107563
C -1.207288 9.840969 1.482811
C -0.484566 9.661323 -0.888382
H 2.493326 -4.448294 0.343421
H 4.750936 -4.836454 -0.609753
H 6.202184 -2.888500 -1.189692
H 1.690684 -2.112700 0.718502
H 6.974302 1.924223 -1.364916
H 2.441045 2.761955 0.540782
H 6.991336 3.321587 -3.157437
H 11.014083 4.784242 -3.112306
H 8.216896 6.073104 -0.138405
H 6.857684 16.456294 -3.183614
H 4.772562 16.873184 -1.905068
H 3.554263 14.957501 -0.863972
H 7.723712 14.124838 -3.416975
H 3.177687 10.192524 0.107752
H 7.359071 9.296819 -2.460147
H 1.757335 8.655690 -0.777560
H -0.487289 7.344935 2.620077
H 3.642383 6.270155 2.210456
H -1.560382 7.127439 -0.664474
H -2.770550 8.358236 -0.241919
H -2.133869 7.270921 1.003741
H -2.090976 10.393295 1.142997
H -0.416116 10.563633 1.709258
H -1.471746 9.325391 2.410902
H -0.201604 9.015103 -1.726708
H 0.302678 10.410119 -0.744124
H -1.397931 10.191559 -1.177697
H 2.946328 4.384083 3.135802
H 1.250113 3.973726 2.864187
H 1.941235 3.849619 4.494397
H -0.611725 5.418152 3.839914
H -0.208503 6.872582 4.781856
H 0.162945 5.267453 5.422263
H 3.525733 6.544801 4.480313
H 2.512717 5.949321 5.807896
H 2.215308 7.554312 5.102487
H 8.536841 1.691283 -5.880329
H 7.390336 2.914093 -5.321546
H 7.859553 1.569722 -4.252192
H 10.135265 3.439375 -6.519137
H 10.769795 4.600644 -5.343606
H 9.095488 4.740568 -5.900043
H 10.814272 1.510501 -4.988615
H 10.228824 1.457902 -3.312270
H 11.442422 2.656289 -3.793117
H 12.068441 6.712396 -2.668300
H 12.569875 5.399286 -1.577714
H 12.837652 7.087511 -1.122586
H 10.106499 8.219036 -2.041063
H 10.978989 8.608044 -0.544301
H 9.302704 8.036148 -0.477782
H 11.327886 5.044219 0.642758
H 10.050937 6.169411 1.131666
H 11.724228 6.734453 1.025483
O 4.117134 6.013677 -2.466045
C 4.859972 5.449117 -3.582952
C 2.667738 5.850768 -2.523581
C 1.987600 7.045952 -3.164250
H 2.346472 5.743009 -1.485660
H 2.450307 4.910266 -3.046634
H 0.920578 7.018894 -2.918702
H 2.405855 7.973339 -2.762609
H 2.087958 7.068208 -4.250422
C 4.487281 6.033072 -4.928940
H 5.902567 5.675877 -3.358375
H 4.724971 4.357883 -3.565800
H 4.540613 7.125702 -4.910703
H 5.213526 5.669430 -5.663988
H 3.495306 5.723766 -5.267721
H 4.983901 8.574081 2.217580
C 5.935829 8.277816 2.663962
H 5.733558 7.770402 3.610666
H 6.508787 9.187333 2.875788
H 5.349548 5.216292 3.810078
C 6.733645 7.413420 1.711621
C 6.379839 5.031410 3.492580
O 6.012712 6.196971 1.330333
H 7.031278 5.785300 3.943161
H 6.945822 7.952684 0.785438
H 7.693823 7.107483 2.145555
C 6.489618 4.982070 1.983066
H 6.683340 4.055702 3.886401
H 5.859559 4.182242 1.592322

H 7.522458 4.794545 1.658726

1-THF

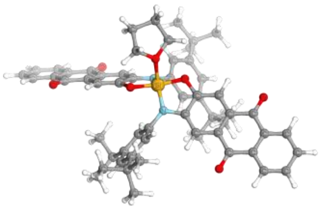

132
xyz, charge: 0, multiplicity: 1
C 2.172254 -2.956865 0.859938
C 3.559844 -3.097343 0.810536
C 4.348533 -2.059825 0.332588
C 3.756600 -0.872473 -0.099995
C 2.360692 -0.731514 -0.051076
C 1.575309 -1.779172 0.431353
C 4.623273 0.224738 -0.611478
C 3.961715 1.472492 -1.047981
C 2.559088 1.601971 -1.018990
C 1.698050 0.522911 -0.502665
C 4.768468 2.523835 -1.500298
C 4.164811 3.696038 -1.924813
C 2.756815 3.825132 -1.868135
C 1.954043 2.796990 -1.432457
O 0.478061 0.646474 -0.437021
O 5.841179 0.088761 -0.664539
O 2.308875 5.040031 -2.240238
N 4.749637 4.869036 -2.382624
C 6.166201 5.041981 -2.305199
C 7.011631 4.271522 -3.102074
C 8.390964 4.426699 -3.004150
C 8.890434 5.388504 -2.112560
C 8.060501 6.174837 -1.317469
C 6.680494 5.973193 -1.417224
C 7.239180 15.259378 -1.788882
C 6.406744 15.396713 -0.677268
C 5.568542 14.355889 -0.301872
C 5.556759 13.167785 -1.033848
C 6.393906 13.029833 -2.152464
C 7.232607 14.081465 -2.523440
C 4.647118 12.067280 -0.614038
C 4.683285 10.813006 -1.396812
C 5.509721 10.685025 -2.529857
C 6.406337 11.775434 -2.954525
C 3.862451 9.755717 -0.986281
C 3.870524 8.576755 -1.713667
C 4.727576 8.446372 -2.836186
C 5.531124 9.483160 -3.252205
O 7.144168 11.660499 -3.929012
O 3.897691 12.203714 0.347205
O 4.713940 7.221683 -3.401749
N 3.165634 7.403002 -1.485499
C 2.432430 7.202301 -0.277591
C 1.301075 7.971035 -0.002345
C 0.594035 7.769928 1.179165
C 1.038914 6.770940 2.058410
C 2.162501 5.990448 1.798375
C 2.862884 6.230456 0.611630
Si 3.573190 6.151196 -2.653944
C 8.589611 7.228004 -0.341311
C 8.014539 8.606930 -0.719448
C 8.140416 6.864624 1.087252
C 10.119590 7.319476 -0.357299
C 9.369951 3.586274 -3.827029
C 10.242641 4.514878 -4.691907
C 10.267910 2.781082 -2.867968
C 8.651580 2.598788 -4.753976
C 2.667616 4.909188 2.756135
C 2.694315 3.552696 2.026376
C 4.094825 5.269319 3.212836
C 1.781952 4.772395 3.999664
C -0.640924 8.596215 1.545023
C -1.853382 7.659756 1.705292
C -0.380650 9.334337 2.872174
C -0.978568 9.642427 0.476165
H 1.557384 -3.770429 1.234230
H 4.024341 -4.019954 1.146726
H 5.429613 -2.144328 0.283868
H 0.498569 -1.644770 0.459937
H 5.846045 2.400153 -1.499638
H 0.876448 2.905413 -1.371606
H 6.566195 3.557210 -3.785540
H 9.964156 5.517580 -2.042317
H 5.990695 6.550817 -0.806995
H 7.894132 16.075418 -2.080581
H 6.414568 16.319379 -0.104069
H 4.911674 14.438506 0.558346
H 7.870344 13.949757 -3.391847
H 3.247067 9.879405 -0.101659
H 6.202802 9.373672 -4.097312
H 0.997090 8.722796 -0.722892
H 0.485095 6.607220 2.975324
H 3.753090 5.656081 0.366669
O 2.490829 6.397934 -4.247460
H -2.046095 7.113632 0.775569
H -2.748340 8.240314 1.957205
H -1.696819 6.923549 2.499498
H -1.253866 9.937719 3.145817
H 0.483905 10.000645 2.781777
H -0.184695 8.636672 3.691915
H -1.213124 9.176344 -0.487620
H -0.157917 10.353576 0.330587
H -1.859057 10.213091 0.789322
H 3.374129 3.564827 1.168674
H 1.697799 3.286293 1.658431
H 3.032855 2.764271 2.708532
H 0.757263 4.487217 3.737038
H 1.749074 5.700312 4.581472
H 2.186630 3.989196 4.648884
H 4.790072 5.311166 2.368405
H 4.467350 4.513492 3.913600
H 4.110299 6.243534 3.713548
H 9.392420 2.021041 -5.316532
H 8.013799 3.116366 -5.479127
H 8.035402 1.888845 -4.191284
H 10.940562 3.923230 -5.295535
H 10.831357 5.205319 -4.080509
H 9.621333 5.111387 -5.368554
H 10.968778 2.159891 -3.437672
H 9.664282 2.124974 -2.231737
H 10.853125 3.437375 -2.216652
H 10.498506 7.599252 -1.346405
H 10.585571 6.373709 -0.059051
H 10.445540 8.087432 0.351772
H 8.292105 8.881739 -1.742843
H 8.399397 9.375424 -0.039020
H 6.921740 8.623353 -0.655982
H 8.512686 5.874673 1.372229
H 7.049573 6.855054 1.178202
H 8.527371 7.599526 1.802412
C 1.018109 6.553190 -4.055515
C 0.565565 7.452971 -5.201462
H 0.879894 7.008531 -3.071615
H 0.580768 5.554884 -4.060066
C 1.846636 8.174530 -5.632548
H -0.220829 8.138510 -4.875217
H 0.174244 6.853595 -6.030281
C 2.876800 7.076759 -5.501568
H 2.089596 8.996865 -4.949483
H 1.792240 8.568764 -6.650712
H 3.907073 7.404176 -5.390916
H 2.792110 6.329873 -6.299842

1-(THF)$_2$

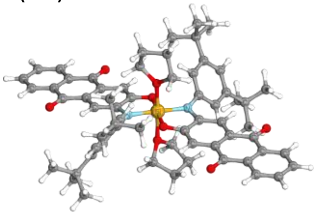

145
xyz, charge: 0, multiplicity: 1
C 3.174723 -3.640783 0.349662
C 4.439562 -3.858879 -0.198122
C 5.242608 -2.781162 -0.545879
C 4.788428 -1.476423 -0.347737
C 3.515827 -1.256935 0.203402
C 2.715090 -2.345870 0.549461
C 5.665828 -0.336975 -0.733206
C 5.157255 1.030389 -0.490969
C 3.873488 1.244220 0.050367
C 3.001775 0.123429 0.431725
C 5.981142 2.107225 -0.831890
C 5.520508 3.407961 -0.649090
C 4.234424 3.606875 -0.082288
C 3.418917 2.554830 0.262980
O 1.891071 0.303025 0.926668
O 6.767454 -0.536358 -1.236182
O 3.913264 4.900946 0.117535
N 6.162174 4.605945 -0.871652
C 7.448205 4.635873 -1.471152
C 7.683489 3.945863 -2.666097
C 8.936848 3.973335 -3.265805
C 9.946816 4.738200 -2.662806
C 9.731705 5.449792 -1.485581
C 8.471837 5.368130 -0.885436
C 6.278574 15.563359 -2.795121
C 5.048995 15.790632 -2.175560
C 4.348034 14.733547 -1.611182
C 4.871308 13.440612 -1.660018
C 6.108679 13.211895 -2.283443
C 6.805824 14.279971 -2.849018
C 4.098330 12.322774 -1.052888
C 4.694521 10.969919 -1.107444
C 5.931990 10.745468 -1.742897
C 6.697186 11.844532 -2.350787
C 3.983103 9.916065 -0.525704
C 4.492135 8.621731 -0.595764
C 5.755003 8.417645 -1.211833
C 6.468804 9.449104 -1.777714
O 7.783861 11.655916 -2.892936
O 3.005329 12.524909 -0.532790
O 6.192970 7.145711 -1.161662
N 3.985827 7.443923 -0.091597
C 2.756554 7.436695 0.623814
C 1.612764 8.023488 0.069419
C 0.405411 8.022220 0.757468
C 0.352963 7.388760 2.007421
C 1.470680 6.780783 2.572730
C 2.679153 6.834223 1.872450
Si 5.081569 6.021564 -0.467446
C 10.803237 6.334493 -0.843247
C 10.317159 7.796865 -0.872937
C 11.031011 5.900513 0.616666
C 12.146237 6.257234 -1.578303
C 9.241689 3.202629 -4.552512
C 9.703734 4.185308 -5.644894
C 10.361097 2.181501 -4.271804
C 8.020198 2.438217 -5.076437
C 1.420302 6.029198 3.905471
C 1.706602 4.538318 3.638538
C 2.487187 6.590380 4.863789
C 0.054857 6.144895 4.592553
C -0.852441 8.685014 0.191039
C -1.972942 7.636241 0.061064
C -1.303117 9.804910 1.148626
C -0.611422 9.306869 -1.189903
H 2.547654 -4.485571 0.620395
H 4.796773 -4.872977 -0.353256
H 6.229618 -2.923656 -0.974678
H 1.735459 -2.149505 0.973892
H 6.968117 1.898211 -1.228915
H 2.439087 2.723755 0.697423
H 6.863926 3.393017 -3.110689
H 10.921988 4.774029 -3.134005
H 8.261501 5.906933 0.032424
H 6.825004 16.391924 -3.236707
H 4.638961 16.795716 -2.134743
H 3.389211 14.883520 -1.124867
H 7.760044 14.077206 -3.325160
H 3.046505 10.140347 -0.028469
H 7.433134 9.274199 -2.242379
H 1.685737 8.481400 -0.909281
H -0.588910 7.372578 2.542989
H 3.569910 6.370046 2.279326
H -1.666260 6.821519 -0.603912
H -2.876365 8.098393 -0.353290
H -2.234988 7.198888 1.029168
H -2.200810 10.297850 0.757733
H -0.516181 10.558814 1.257995
H -1.539444 9.414680 2.143381
H -0.310446 8.553174 -1.927051

H 0.154720 10.089743 -1.156103
H -1.538116 9.766958 -1.548369
H 2.685048 4.393984 3.168209
H 0.953132 4.112500 2.967620
H 1.691227 3.971573 4.576790
H -0.743351 5.707197 3.983881
H -0.202481 7.187858 4.808337
H 0.077700 5.602216 5.543389
H 3.498167 6.454953 4.466175
H 2.438954 6.072046 5.828279
H 2.332357 7.660757 5.037333
H 8.291920 1.901044 -5.991105
H 7.194167 3.114853 -5.323661
H 7.661526 1.698843 -4.351753
H 9.915983 3.644342 -6.574321
H 10.613123 4.719476 -5.353445
H 8.927133 4.930475 -5.849625
H 10.589675 1.612410 -5.180420
H 10.053491 1.476547 -3.492089
H 11.281980 2.671733 -3.941180
H 12.061837 6.604062 -2.613638
H 12.546673 5.237417 -1.583298
H 12.875602 6.900074 -1.074332
H 10.159657 8.132313 -1.903490
H 11.058160 8.456396 -0.406348
H 9.369061 7.917003 -0.338095
H 11.343461 4.851985 0.667250
H 10.122492 6.010767 1.217303
H 11.810864 6.517529 1.077674
O 4.212580 5.759668 -2.350738
C 4.916415 6.017955 -3.599609
C 2.813311 6.037326 -2.630267
C 2.842814 7.298663 -3.488703
H 2.294751 6.132972 -1.675788
H 2.414936 5.173734 -3.179542
C 4.215268 7.238143 -4.208554
H 5.969548 6.165265 -3.361226
H 4.801488 5.122221 -4.223916
C 6.736810 7.092166 3.345371
C 6.387621 7.453798 1.911668
C 7.209593 5.639720 3.225933
O 5.935555 6.186511 1.323245
H 5.572179 8.174217 1.816185
H 7.253608 7.797315 1.333507
C 6.249308 5.054671 2.205062
H 2.790722 8.190697 -2.858461
H 2.000766 7.325964 -4.185574
H 4.111038 7.122935 -5.290774
H 4.788550 8.149012 -4.018852
H 5.845901 7.155563 3.980343
H 7.499655 7.758856 3.755590
H 8.236633 5.599256 2.845495
H 7.172183 5.097340 4.174136
H 6.675664 4.261436 1.587820
H 5.308314 4.710575 2.651239

1-pyridine

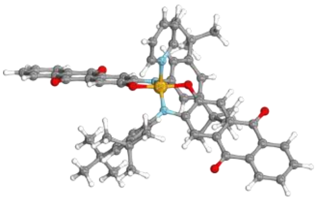

130
xyz, charge: 0, multiplicity: 1
C -1.388220 3.357758 1.294814
C -1.671291 2.291153 2.149168
C -0.105690 3.525201 0.790384
C 0.905269 2.627382 1.135617
C 0.620613 1.554513 1.995324
C -0.671408 1.393298 2.497504
H -2.172511 4.058790 1.023630
H -2.675505 2.162872 2.543061
H 0.140313 4.346683 0.124812
H -0.865892 0.555606 3.159904
C 2.273841 2.828802 0.584176
C 3.312011 1.852859 0.962148
C 3.036379 0.792301 1.848415
C 1.675133 0.575949 2.380671
C 4.607048 2.027297 0.455058
C 5.592929 1.145836 0.838292
C 5.321929 0.072061 1.721461
C 4.045644 -0.100463 2.230649
H 3.806791 -0.901948 2.921488
H 4.820632 2.858316 -0.208357
O 2.519436 3.779837 -0.153032
O 1.411641 -0.373519 3.111896
O 6.878862 1.226237 0.459939
N 6.473059 -0.663202 1.977774
C 6.434821 -1.762043 2.889349
C 5.665778 -2.885241 2.580274
C 5.623236 -3.965187 3.456177
C 6.388542 -3.896655 4.629953
C 7.166310 -2.787073 4.952668
C 7.165489 -1.705846 4.064577
H 7.748713 -0.812740 4.272105
H 6.366915 -4.740059 5.310027
H 5.100562 -2.884747 1.654107
C 4.772272 -5.207718 3.184155
C 7.999857 -2.701417 6.233548
C 9.479181 -2.472791 5.868160
H 9.863297 -3.292301 5.251508
H 10.087379 -2.415968 6.778202
H 9.624210 -1.542283 5.311137
C 7.494412 -1.519618 7.083402
H 7.593563 -0.568214 6.551958
H 8.074199 -1.447586 8.010790
H 6.438908 -1.652235 7.344481
C 7.904933 -3.977231 7.078102
H 8.271397 -4.852952 6.531229
H 6.878173 -4.173234 7.406525
H 8.523093 -3.864966 7.974839
C 5.678206 -6.452622 3.138613
H 6.431364 -6.355529 2.349026
H 5.079337 -7.348216 2.936479
H 6.203590 -6.608038 4.085617
C 4.014777 -5.114264 1.854411
H 4.699430 -5.026257 1.003127
H 3.326319 -4.262289 1.838603
H 3.421078 -6.022740 1.708800
C 3.737402 -5.365389 4.314966
H 4.219708 -5.493475 5.288774
H 3.108835 -6.244627 4.131882
H 3.090075 -4.483798 4.371715
H 11.492109 3.899222 -1.491927
H 6.790529 7.113135 1.842887
H 5.378766 4.986010 1.555687
H 9.456346 6.968454 -0.661134
H 11.016343 7.114423 1.381013
H 7.041293 3.927632 4.777005
C 13.625454 0.820173 3.793606
C 11.072587 -1.760965 2.684170
C 10.104241 -0.923612 2.177287
C 9.085324 2.541029 1.602522
C 10.265055 0.483641 2.186697
C 11.409671 1.046087 2.726590
C 12.399613 0.202715 3.246689
C 14.652481 -0.086499 4.377685
C 15.812767 0.464199 4.923552
C 14.472928 -1.478926 4.382498
C 15.455650 -2.301947 4.934031
C 9.998469 3.246674 0.817052
C 12.235835 -1.196927 3.225725
C 13.245572 -2.098115 3.809924
C 16.785388 -0.361738 5.470409
C 16.606743 -1.745843 5.475441
C 8.015789 4.602430 2.273161
C 7.113562 5.000102 4.572160
C 8.114711 3.207919 2.331500
C 10.936367 5.461736 -0.061398
C 5.540624 4.791462 2.621219
C 6.948297 6.829590 2.889276
C 11.695030 6.419140 0.877867
C 11.967575 4.584727 -0.781173
C 10.171254 6.278636 -1.119686
C 6.919519 5.308202 3.074937
C 8.938567 5.289406 1.487845
C 9.943896 4.635522 0.759910
N 9.150931 1.114998 1.646416
O 13.087544 -3.315799 3.834049
O 8.929615 -1.332247 1.671141
O 13.802171 2.034194 3.770023
H 6.342230 5.506696 5.163561
H 17.369476 -2.389945 5.903776
H 7.894198 7.263863 3.231654
H 9.614656 5.616802 -1.792239
H 12.235645 5.857901 1.647414
H 5.434522 3.714327 2.781633
H 15.292443 -3.375099 4.926202
H 17.686685 0.070839 5.895374
H 8.885065 6.370546 1.437350
H 10.939603 -2.837356 2.690868
H 7.424368 2.624143 2.934371
H 10.749384 2.686364 0.269826
H 15.927831 1.543470 4.907164
H 11.558681 2.119884 2.764500
H 12.656115 5.221364 -1.346523
H 12.563494 3.996932 -0.074375
H 6.144007 7.281642 3.478854
H 12.420728 7.010646 0.307695
H 4.745856 5.293637 3.184671
H 10.872070 6.870714 -1.719643
H 8.094969 5.344386 4.915869
Si 7.893942 -0.020609 1.151144
N 8.116657 -0.694037 -0.648056
C 8.420001 0.152106 -1.651700
C 7.968016 -2.009636 -0.897843
C 8.599429 -0.302302 -2.945047
C 8.106215 -2.518404 -2.175926
C 8.433863 -1.656024 -3.216378
H 8.558600 -2.034293 -4.226319
H 7.965847 -3.580271 -2.344131
H 8.509551 1.200351 -1.383295
H 8.856998 0.403922 -3.726372
H 7.739382 -2.636382 -0.041235

1-(pyridine)$_2$ trans

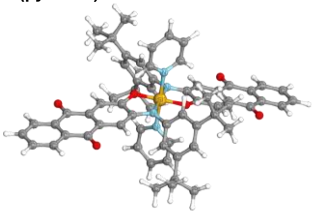

141
xyz, charge: 0, multiplicity: 1
C -1.895751 3.209101 0.902967
C -2.244970 1.917618 1.299871
C -0.584820 3.648156 1.032487
C 0.389270 2.800007 1.560297
C 0.037798 1.500598 1.959649
C -1.281906 1.066809 1.825201
H -2.650796 3.873126 0.491645
H -3.271344 1.576804 1.197621
H -0.287872 4.647937 0.731440
H -1.526984 0.057279 2.140111
C 1.788518 3.295827 1.693840
C 2.784726 2.372903 2.254878
C 2.437957 1.069330 2.664587
C 1.053571 0.567741 2.520738
C 4.108677 2.816738 2.401030
C 5.046500 1.960946 2.931161
C 4.715818 0.637081 3.330784
C 3.396127 0.207735 3.205841
H 3.085169 -0.781518 3.522849
H 4.376566 3.825122 2.102625
O 2.082959 4.438178 1.346926
O 0.744255 -0.574670 2.844558
O 6.333602 2.300626 3.144084
N 5.801828 -0.017924 3.860190
C 5.725521 -1.394490 4.191695
C 5.216281 -2.316758 3.268920
C 5.180205 -3.673008 3.570364
C 5.688803 -4.093358 4.808786
C 6.210354 -3.195588 5.737478
C 6.203735 -1.835455 5.417461
H 6.604706 -1.102674 6.109740
H 5.674776 -5.151158 5.044254
H 4.861130 -1.946812 2.313743
C 4.618015 -4.708858 2.593533
C 6.810276 -3.636424 7.074759
C 8.303428 -3.254116 7.096074
H 8.844638 -3.761687 6.290354
H 8.757881 -3.544948 8.050391

H 8.446019 -2.176367 6.964581
C 6.081350 -2.922555 8.228800
H 6.185308 -1.834873 8.164418
H 6.496531 -3.241961 9.191753
H 5.012108 -3.159945 8.217703
C 6.696071 -5.148426 7.299303
H 7.236138 -5.714070 6.532459
H 5.651474 -5.479003 7.306399
H 7.134210 -5.407038 8.269045
C 5.720670 -5.721244 2.230632
H 6.573031 -5.214184 1.764429
H 5.334568 -6.465932 1.524865
H 6.089804 -6.253708 3.112367
C 4.106060 -4.068173 1.297979
H 4.907037 -3.551049 0.756650
H 3.298633 -3.352906 1.490001
H 3.710067 -4.847180 0.638002
C 3.441568 -5.446861 3.260549
H 3.755757 -5.972188 4.167600
H 3.020131 -6.187701 2.571122
H 2.649900 -4.741510 3.534636
H 10.230598 2.881469 -1.130343
H 7.400216 8.040668 1.840390
H 5.752272 6.292918 2.670329
H 8.915159 6.419898 -1.054214
H 11.114475 6.917820 0.192093
H 8.124156 6.001586 5.599813
C 13.733879 0.844742 3.183202
C 10.629658 -1.121582 4.133709
C 9.672041 -0.155306 3.928340
C 8.984240 3.244449 2.798597
C 10.005760 1.148738 3.470403
C 11.345887 1.461109 3.251193
C 12.324733 0.484267 3.453792
C 14.769462 -0.198430 3.420480
C 16.110925 0.111673 3.192073
C 14.415809 -1.483667 3.861608
C 15.409376 -2.441068 4.068936
C 9.620229 3.428074 1.564440
C 11.976815 -0.810426 3.889822
C 12.993233 -1.846461 4.118754
C 17.093187 -0.846723 3.401841
C 16.741777 -2.124104 3.840283
C 8.406513 5.596146 2.860933
C 8.263473 6.904875 4.997646
C 8.396672 4.324989 3.441089
C 10.372070 4.928643 -0.364043
C 6.186582 6.401278 3.670005
C 7.835723 8.078559 2.844662
C 11.500536 5.958918 -0.166969
C 10.990356 3.648056 -0.937596
C 9.352029 5.471900 -1.382335
C 7.687359 6.740956 3.578486
C 9.054403 5.757173 1.638292
C 9.673598 4.686102 0.976218
N 8.893844 1.950341 3.371250
O 12.696405 -2.973297 4.511409
O 8.358114 -0.341791 4.165545
O 14.047927 1.958050 2.773853
H 7.762813 7.731906 5.514386
H 17.511919 -2.872775 4.003299
H 8.885975 8.378761 2.758838
H 8.533049 4.758388 -1.527825
H 12.232367 5.594212 0.561593
H 6.017166 5.463376 4.209334
H 15.110095 -3.427095 4.410352
H 18.136353 -0.601348 3.223943
H 9.082793 6.738189 1.178583
H 10.359290 -2.111942 4.485421
H 7.898982 4.154064 4.389819
H 10.059466 2.564388 1.078183
H 16.357480 1.111962 2.850004
H 11.656623 2.449109 2.930031
H 11.480495 3.875252 -1.890152
H 11.746816 3.226202 -0.266598
H 7.309858 8.860709 3.402303
H 12.018724 6.138520 -1.116168
H 5.646573 7.198814 4.193616
H 9.836858 5.642198 -2.350665
H 9.336487 7.121214 4.957903
Si 7.345819 0.978347 3.652240
N 7.460307 0.314464 1.708950
C 7.726528 -0.978959 1.443121
C 7.269334 1.166239 0.683067
C 7.339074 0.751401 -0.636418
C 7.613075 -0.582354 -0.914101
C 7.809171 -1.458536 0.146549
H 7.865756 -1.624445 2.303530
H 8.028658 -2.507869 -0.018176
H 7.064403 2.197230 0.950278
H 7.175396 1.473428 -1.429065
C 6.064591 2.080607 6.097524
N 7.230838 1.640619 5.594915
H 5.208264 2.060417 5.431696
C 5.951209 2.537639 7.400256
C 8.320465 1.639402 6.381687
H 4.989747 2.887144 7.760110
H 9.242760 1.281466 5.936394
C 7.075035 2.532853 8.217438
C 8.278348 2.072915 7.696624
H 9.182924 2.049160 8.294313
H 7.672789 -0.933123 -1.940025
H 7.014047 2.882008 9.243866

1-(pyridine)$_2$ cis

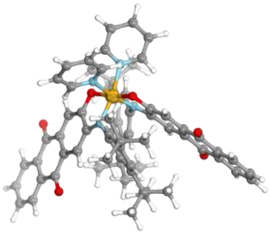

141
xyz, charge: 0, multiplicity: 1
C 1.603239 5.155430 -1.479820
C 1.941869 4.213336 -2.451885
C 1.815556 4.878095 -0.135775
C 2.367030 3.655817 0.248975
C 2.708258 2.708139 -0.729228
C 2.493262 2.995233 -2.077767
H 1.173527 6.108744 -1.774584
H 1.775845 4.433092 -3.502728
H 1.562944 5.594400 0.639778
H 2.769523 2.246697 -2.813846
C 2.591091 3.383134 1.696927
C 3.181649 2.091211 2.058846
C 3.535441 1.135067 1.083865
C 3.316471 1.401028 -0.354755
C 3.422362 1.819883 3.415253
C 4.021324 0.635964 3.762217
C 4.404188 -0.330388 2.790105
C 4.130932 -0.075815 1.447252
H 3.160337 2.557446 4.165634
H 4.388752 -0.787239 0.670069
O 2.293464 4.225651 2.544278
O 3.633545 0.588499 -1.217743
O 4.314055 0.289719 5.040177
N 4.994141 -1.422552 3.372514
C 5.597136 -2.456727 2.609444
C 5.317914 -3.779011 2.928797
C 6.536246 -2.162724 1.614708
C 7.205956 -3.183618 0.950862
C 6.914395 -4.509952 1.303316
C 5.982501 -4.828185 2.287027
H 4.593349 -3.980866 3.711485
H 7.440450 -5.308335 0.793995
H 6.751372 -1.122467 1.400646
C 8.243459 -2.906046 -0.139839
C 8.531214 -1.408771 -0.293556
H 7.639801 -0.850286 -0.598875
H 9.294436 -1.259116 -1.064545
H 8.914038 -0.978469 0.639711
C 7.709334 -3.444561 -1.480892
H 6.765552 -2.955529 -1.745583
H 7.530621 -4.523790 -1.435772
H 8.433968 -3.253488 -2.281200
C 9.569098 -3.608115 0.207265
H 9.955100 -3.239031 1.161954
H 10.317645 -3.403643 -0.566815
H 9.458182 -4.693514 0.287514
C 5.683623 -6.269148 2.709703
C 6.412742 -7.298600 1.838548
H 6.127165 -7.209500 0.784539
H 6.152226 -8.308929 2.171974
H 7.500095 -7.195396 1.917137
C 4.170303 -6.537186 2.607317
H 3.951598 -7.575258 2.884093
H 3.815256 -6.370005 1.584734
H 3.594961 -5.885661 3.273109
C 6.141862 -6.464730 4.168159
H 7.225987 -6.330920 4.249818
H 5.893421 -7.475963 4.512811
H 5.658907 -5.744505 4.838514
H 15.449677 -3.097297 1.468411
C 14.492137 -3.301812 1.938967
C 14.147952 -4.610001 2.281380
H 14.838143 -5.423812 2.077183
C 13.610789 -2.260458 2.197042
H 13.852099 -1.234492 1.937030
H 9.502706 3.780601 1.519402
H 8.458730 5.199067 1.714869
C 12.923937 -4.874883 2.881434
H 8.846195 4.606462 0.088938
C 8.624344 4.296860 1.117052
C 12.379867 -2.517819 2.801807
O 11.747595 -0.241193 2.728473
C 12.033765 -3.834127 3.147092
C 11.446836 -1.384739 3.056141
H 12.630879 -5.883793 3.154558
C 10.722099 -4.143730 3.783463
C 10.151531 -1.696169 3.698831
O 10.419996 -5.305415 4.058582
C 9.819595 -3.020561 4.053639
C 9.256287 -0.646483 3.921437
H 9.546706 0.358995 3.636530
H 8.050327 5.208473 6.404436
C 7.391938 3.372951 1.140392
C 8.576145 -3.287613 4.647709
C 8.006576 -0.915730 4.477283
H 7.495069 0.846546 2.321217
C 7.231454 1.635440 3.015726
C 7.697756 -2.254071 4.849600
C 7.114557 2.956559 2.587313
H 8.559076 1.613773 0.552756
H 5.943106 5.032111 1.130533
H 6.477750 3.664310 7.688440
N 6.972101 -0.062015 4.764198
H 8.313428 -4.305617 4.911600
C 6.966570 1.293532 4.342302
C 7.667423 2.166052 0.237002
C 6.173772 4.121634 0.569408
C 7.164361 5.708429 5.997935
H 6.361034 4.408005 -0.471864
H 7.836861 2.507067 -0.789795
C 6.723019 3.919700 3.520782
O 6.467695 -2.412886 5.397534
C 6.590958 2.277409 5.255705
C 6.447065 3.603317 4.852863
H 6.621108 4.950440 3.195911
H 7.445823 6.165827 5.044362
H 6.871560 6.510962 6.685557
C 5.628159 4.152559 7.197329
H 6.383214 1.977358 6.275670
C 6.007460 4.707352 5.818052
H 5.298379 4.973572 7.843070
H 6.813986 1.477644 0.220918
H 5.287949 3.480120 0.593714
H 4.806236 3.430418 7.124261
C 4.780000 5.440814 5.244772
H 5.003054 5.929306 4.292257
H 4.443507 6.214531 5.944988
H 3.950601 4.749295 5.065961
Si 5.440401 -1.034676 5.124822
C 1.688250 -3.042557 5.521466
C 2.714593 -2.159454 5.230317
C 1.924835 -4.086058 6.408520
N 3.932852 -2.275850 5.786925
C 3.183404 -4.200257 6.985730
C 4.161630 -3.276746 6.654730
H 5.165557 -3.335752 7.059966
H 0.723495 -2.912463 5.043687
H 2.579413 -1.343660 4.528192
H 1.141685 -4.800785 6.643101
H 3.418034 -5.000608 7.678967
C 5.922839 0.355382 9.721159

C 4.694371 0.485146 9.085649
C 6.973448 -0.237293 9.030864
C 4.557715 0.026521 7.785698
C 6.768312 -0.667618 7.730609
N 5.579168 -0.536508 7.117210
H 7.951511 -0.359415 9.483002
H 3.626881 0.124554 7.238484
H 7.565703 -1.117793 7.149055
H 6.060658 0.713634 10.736947
H 3.846788 0.946369 9.580672

1-(pyridine)$_2$ cis 2

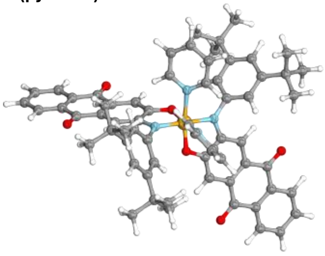

141
xyz, charge: 0, multiplicity: 1
C -2.363731 19.187536 3.838521
C -2.399153 18.139147 2.918693
C -1.157797 19.801439 4.150044
C 0.023390 19.374664 3.541847
C -0.003566 18.334500 2.622650
C -1.210902 17.709855 2.306789
H 0.965679 19.856026 3.787925
H -1.134590 20.615199 4.869422
H -3.296464 19.501038 4.296928
H 0.900584 17.984444 2.134426
C -3.706143 17.495295 2.601652
C -3.696955 16.370049 1.655249
C -2.506263 15.934285 1.040167
C -1.215797 16.598453 1.315424
C -4.907092 15.719257 1.367885
C -4.909071 14.670617 0.475256
C -3.721538 14.243438 -0.174028
C -2.518179 14.869665 0.132806
H -5.821053 16.039968 1.857057
H -1.579865 14.557620 -0.311964
O -4.743662 17.901542 3.120145
O -0.179293 16.258524 0.751529
O -6.006837 13.982048 0.119060
N -3.954813 13.203823 -1.052775
C -2.813467 12.556981 -1.605883
C -2.029216 13.235753 -2.547188
C -2.475687 11.268457 -1.218275
C -1.371967 10.619902 -1.787366
C -0.617398 11.308686 -2.733227
C -0.919447 12.625116 -3.118078
H -2.313793 14.249850 -2.810348
H -3.089667 10.784378 -0.466448
C -0.016589 13.341107 -4.125205
C -0.502092 14.761959 -4.434877
H -1.506168 14.761555 -4.874814
H 0.175537 15.229461 -5.156894
H -0.514557 15.389763 -3.537411
H 0.245698 10.825397 -3.176158
C 0.021293 12.545090 -5.443132
H -0.982269 12.468473 -5.877564
H 0.403889 11.530607 -5.295767
H 0.670657 13.046371 -6.170111
C 1.406295 13.437589 -3.541089
H 1.397021 13.987553 -2.594163
H 2.067202 13.963344 -4.239940
H 1.833880 12.448131 -3.352591
C -1.022920 9.204770 -1.319912
C -0.718205 9.238857 0.190347
H 0.117867 9.914105 0.400582
H -0.451560 8.236476 0.544509
H -1.580937 9.581810 0.768991
C -2.219796 8.270167 -1.575679
H -3.116941 8.606618 -1.046235
H -1.988060 7.255671 -1.231600
H -2.453062 8.227465 -2.645752
C 0.198460 8.630828 -2.046764
H 1.097508 9.229136 -1.863497
H 0.035643 8.568393 -3.128794
H 0.396459 7.617756 -1.681379
H -10.307194 10.223737 -4.251662
H -11.278027 16.513286 -3.262478
H -10.794488 12.419215 -5.452427
H -9.548815 16.882466 -3.142213
H -12.021812 9.808662 -4.361672
C -11.274431 10.318485 -3.744794
C -10.502063 16.913396 -2.602563
H -12.488584 11.904417 -5.579691
C -11.769190 12.442006 -4.951179
H -10.745485 17.961824 -2.395245
H -11.219175 9.791458 -2.786109
C -11.679781 11.785626 -3.560797
C -10.914345 13.909490 -2.409534
C -8.613153 6.387307 4.490185
C -9.784732 5.798996 4.947923
C -8.944271 14.029750 -1.049388
C -10.066148 14.658862 -1.590326
C -11.017788 6.198457 4.430974
C -10.683297 12.559513 -2.693718
C -7.392409 8.000026 3.039519
C -8.664045 7.382718 3.513962
C -11.077049 7.185772 3.456647
C -9.904496 7.784575 2.993892
C -6.312180 9.696574 1.599533
C -7.490182 9.070781 2.036694
C -9.992506 8.839077 1.945931
C -6.393278 10.693854 0.653315
C -8.733333 9.479464 1.513821
C -8.806325 10.491791 0.551275
C -7.637136 11.092986 0.097280
C -8.667711 12.698536 -1.353362
O -6.310789 7.617101 3.479792
O -5.332332 11.352924 0.156747
O -11.077335 9.153004 1.463737
N -7.486771 12.080439 -0.855904
H -12.092254 13.485708 -4.887892
C -9.539995 11.966351 -2.161786
C -10.400264 16.121759 -1.285304
H -11.797368 14.386547 -2.824760
H -8.257282 14.552293 -0.395938
H -7.641472 6.094180 4.875326
H -9.740464 5.026618 5.710629
H -11.932957 5.737197 4.791341
H -5.356514 9.397492 2.017516
H -12.023058 7.513592 3.037142
H -9.782382 10.784333 0.180702
H -9.299585 10.926975 -2.362353
H -8.348918 16.776867 -0.888301
H -12.563210 15.774860 -1.151054
C -9.334399 16.789487 -0.408305
C -11.749727 16.185480 -0.544629
H -13.789260 11.252063 -3.483342
C -13.064798 11.817901 -2.886294
H -9.607132 17.835319 -0.233031
H -11.999906 17.224665 -0.301668
H -13.017124 11.371721 -1.887430
H -13.440963 12.840073 -2.781603
H -9.245637 16.302347 0.568045
H -11.704253 15.614343 0.388433
Si -5.719259 12.638920 -0.967281
C -6.362708 16.099605 -3.456650
C -5.917411 15.181330 -2.519725
C -7.207945 15.674513 -4.474044
C -7.562804 14.333386 -4.528011
C -7.069278 13.471541 -3.563793
N -6.266397 13.883830 -2.566175
H -5.268800 15.488097 -1.706611
H -6.052136 17.135246 -3.371838
H -7.583973 16.377117 -5.211847
H -8.226031 13.949653 -5.295080
H -7.330414 12.420832 -3.573303
C -4.618466 11.662545 -3.624110
C -4.255418 10.752549 -4.602076
H -4.329923 12.703393 -3.698778
N -5.327945 11.311346 -2.536754
H -3.664131 11.087978 -5.446749
C -4.648990 9.428104 -4.466110
C -5.711971 10.029095 -2.408248
C -5.398271 9.066793 -3.353668
H -4.375555 8.689126 -5.213492
H -6.281973 9.772323 -1.522240
H -5.730044 8.045877 -3.199553

1-DMAP

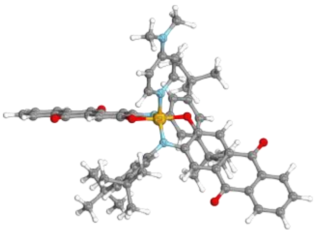

138
xyz, charge: 0, multiplicity: 1
C -1.233216 3.683031 1.440880
C -1.515119 2.641730 2.326186
C 0.025032 3.784815 0.862876
C 1.012544 2.846306 1.164922
C 0.729171 1.799284 2.055833
C -0.538249 1.703746 2.631861
H -1.999237 4.415812 1.203266
H -2.500146 2.564679 2.777794
H 0.270086 4.585366 0.171897
H -0.731867 0.884423 3.317140
C 2.355998 2.977299 0.534546
C 3.368795 1.962211 0.870370
C 3.096469 0.927378 1.788062
C 1.759831 0.778934 2.397138
C 4.643395 2.076502 0.297395
C 5.612448 1.161516 0.643451
C 5.340813 0.107624 1.552135
C 4.087152 -0.002270 2.130594
H 3.853403 -0.783017 2.846585
H 4.858405 2.894262 -0.382037
O 2.599286 3.906831 -0.231328
O 1.494297 -0.146921 3.157942
O 6.880502 1.190719 0.208834
N 6.469708 -0.671848 1.761309
C 6.459358 -1.684210 2.768540
C 5.686784 -2.831998 2.590799
C 5.668077 -3.818921 3.571527
C 6.457195 -3.631758 4.716324
C 7.237549 -2.494203 4.910060
C 7.215663 -1.509954 3.916221
H 7.799991 -0.599035 4.019865
H 6.452368 -4.402465 5.478194
H 5.104024 -2.923917 1.680387
C 4.819161 -5.086032 3.443598
C 8.091753 -2.274649 6.161107
C 9.563832 -2.078458 5.750919
H 9.941704 -2.953137 5.210891
H 10.185715 -1.929614 6.641303
H 9.694974 -1.206644 5.102752
C 7.593955 -1.013237 6.893160
H 7.682125 -0.121305 6.265175
H 8.187381 -0.844954 7.799254
H 6.542836 -1.120577 7.182171
C 8.016654 -3.457265 7.133590
H 8.379351 -4.382991 6.673390
H 6.996000 -3.622360 7.496364
H 8.647971 -3.251063 8.004138
C 5.733229 -6.324721 3.496521
H 6.466399 -6.299863 2.682951
H 5.136766 -7.239156 3.397039
H 6.282640 -6.384068 4.440810
C 4.029272 -5.126736 2.130159
H 4.692959 -5.118998 1.258160
H 3.335491 -4.282947 2.048157
H 3.438109 -6.047663 2.089069
C 3.813031 -5.138684 4.609481
H 4.319393 -5.168880 5.579013
H 3.186805 -6.034880 4.528383
H 3.160664 -4.259059 4.596563
H 11.319106 4.232937 -1.412724
H 6.589275 6.882354 2.312443
H 5.239905 4.742595 1.829307
H 9.279246 7.148572 -0.143738
H 10.871205 7.026204 1.874570
H 7.008637 3.416956 4.887083
C 13.496795 0.654540 3.792377
C 11.002862 -1.885500 2.474930
C 10.056199 -1.032138 1.953839
C 9.022813 2.446011 1.545704

C	10.218815	0.375030	2.013317
C	11.335844	0.917524	2.626850
C	12.301934	0.056749	3.164211
C	14.494605	-0.271988	4.397140
C	15.624331	0.259100	5.020836
C	14.317072	-1.663382	4.344469
C	15.271004	-2.505299	4.917501
C	9.908695	3.260937	0.840562
C	12.141669	-1.341572	3.085770
C	13.121183	-2.261837	3.687865
C	16.568651	-0.585500	5.588686
C	16.391843	-1.968834	5.536832
C	7.903057	4.386926	2.454854
C	7.038955	4.507300	4.797981
C	8.039986	2.998577	2.350870
C	10.780663	5.587736	0.220157
C	5.429607	4.449802	2.867451
C	6.774162	6.497310	3.321343
C	11.548303	6.417889	1.267185
C	11.804912	4.829345	-0.632163
C	9.988081	6.531481	-0.703805
C	6.800602	4.964764	3.345883
C	8.800378	5.184159	1.747899
C	9.814938	4.645424	0.942519
N	9.132446	1.025753	1.445257
O	12.966495	-3.480521	3.661943
O	8.902713	-1.422213	1.393068
O	13.673844	1.868782	3.821536
H	6.263903	4.919042	5.454701
H	17.132127	-2.627697	5.981910
H	7.710315	6.927210	3.694674
H	9.421301	5.960426	-1.447258
H	12.110874	5.763556	1.941636
H	5.360348	3.359151	2.922169
H	15.109399	-3.577418	4.864209
H	17.446062	-0.168098	6.074574
H	8.719073	6.262168	1.823305
H	10.866825	-2.961284	2.447028
H	7.371733	2.330225	2.888083
H	10.667159	2.786221	0.227164
H	15.738195	1.338316	5.047551
H	11.479986	1.989525	2.709646
H	12.469216	5.545920	-1.126618
H	12.427940	4.165679	-0.022768
H	5.965183	6.856209	3.966091
H	12.255239	7.093100	0.770887
H	4.631898	4.866930	3.492989
H	10.672926	7.204342	-1.232967
H	8.015582	4.848769	5.157840
Si	7.883713	-0.088413	0.874673
N	8.086932	-0.739863	-0.909423
C	8.251723	0.111663	-1.948891
C	8.071204	-2.067542	-1.173585
C	8.416627	-0.316007	-3.240079
C	8.199091	-2.574180	-2.440136
C	8.387987	-1.700819	-3.540233
N	8.532390	-2.161899	-4.802365
H	8.159939	-3.648141	-2.565312
H	8.246142	1.168161	-1.704018
H	8.558258	0.431065	-4.009762
H	7.950580	-2.721644	-0.316964
C	8.720123	-1.228523	-5.908888
C	8.502336	-3.596789	-5.068675
H	9.638087	-0.639127	-5.785231
H	7.869907	-0.540282	-6.000004
H	8.801406	-1.789612	-6.839879
H	9.302936	-4.120439	-4.530394
H	8.646949	-3.765171	-6.135872
H	7.538785	-4.037999	-4.781972

1-(DMAP)$_2$

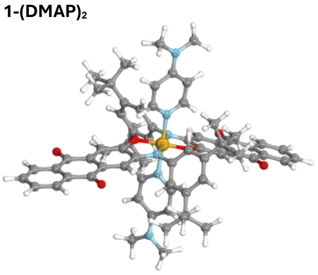

157

xyz, charge: 0, multiplicity: 1

C	-1.831065	2.900208	0.683308
C	-2.181327	1.614846	1.098494
C	-0.524081	3.347368	0.826213
C	0.444889	2.513960	1.385879
C	0.092346	1.221004	1.803507
C	-1.222965	0.778642	1.655404
H	-2.582071	3.553209	0.247419
H	-3.204479	1.267377	0.985976
H	-0.225784	4.342645	0.511706
H	-1.468180	-0.226083	1.985385
C	1.840732	3.018843	1.533536
C	2.829025	2.113657	2.129921
C	2.481099	0.815147	2.557776
C	1.103970	0.303585	2.399068
C	4.148227	2.566575	2.295519
C	5.083020	1.727778	2.858159
C	4.751664	0.404750	3.270930
C	3.434509	-0.030768	3.130973
H	3.121078	-1.014331	3.462452
H	4.416002	3.571856	1.986857
O	2.132523	4.156720	1.166557
O	0.791408	-0.834629	2.737281
O	6.357999	2.085021	3.095629
N	5.828397	-0.234796	3.827890
C	5.749455	-1.587174	4.235935
C	5.234491	-2.564114	3.374307
C	5.200958	-3.899648	3.756792
C	5.713766	-4.246097	5.016442
C	6.242253	-3.293588	5.884730
C	6.236834	-1.956115	5.482567
H	6.644605	-1.183959	6.125903
H	5.699720	-5.287774	5.315567
H	4.880579	-2.252302	2.398323
C	4.638453	-4.992418	2.843962
C	6.850360	-3.650793	7.243311
C	8.344331	-3.271550	7.230955
H	8.878097	-3.824373	6.450581
H	8.805640	-3.508471	8.197121
H	8.486596	-2.203431	7.036108
C	6.132252	-2.862037	8.354874
H	6.241154	-1.781091	8.219684
H	6.552512	-3.122264	9.333632
H	5.061760	-3.094171	8.365274
C	6.733099	-5.145034	7.564103
H	7.267190	-5.759207	6.831167
H	5.687463	-5.470947	7.596502
H	7.175590	-5.343962	8.546039
C	5.739139	-6.027865	2.545803
H	6.594986	-5.551468	2.054419
H	5.353118	-6.812464	1.884321
H	6.103473	-6.507816	3.459086
C	4.131541	-4.431200	1.509947
H	4.935336	-3.946746	0.943018
H	3.324183	-3.704980	1.655102
H	3.737704	-5.248918	0.896825
C	3.458456	-5.685985	3.551047
H	3.769135	-6.154358	4.490005
H	3.037474	-6.467486	2.907508
H	2.667534	-4.963461	3.778652
H	10.208351	3.018651	-1.102781
H	7.375296	7.907330	2.276357
H	5.761116	6.074765	3.004534
H	8.900762	6.542503	-0.739886
H	11.113755	6.939084	0.516810
H	8.187545	5.604621	5.860264
C	13.754187	0.605563	2.960304
C	10.664136	-1.363948	3.946350
C	9.707172	-0.385164	3.804328
C	9.017796	3.072124	2.853924
C	10.039215	0.931404	3.371985
C	11.376679	1.237883	3.124093
C	12.352976	0.247646	3.263528
C	14.788141	-0.453793	3.129352
C	16.124620	-0.147291	2.869468
C	14.436215	-1.749923	3.537603
C	15.426852	-2.721924	3.681002
C	9.641760	3.355074	1.632135
C	12.006489	-1.058957	3.667272
C	13.017743	-2.109480	3.826939
C	17.104359	-1.120240	3.015696
C	16.754698	-2.408726	3.421602
C	8.425520	5.409495	3.098382
C	8.303816	6.554197	5.327840
C	8.427432	4.098248	3.579335
C	10.362851	5.000971	-0.185541
C	6.210315	6.124631	4.002132
C	7.825318	7.878895	3.274540
C	11.494905	6.012366	0.076954
C	10.972036	3.768676	-0.865144
C	9.331336	5.622260	-1.146006
C	7.705113	6.489158	3.909868
C	9.062815	5.669278	1.887279
C	9.681021	4.654290	1.140677
N	8.935235	1.742692	3.330945
O	12.726506	-3.249154	4.188745
O	8.405029	-0.569286	4.087595
O	14.068879	1.729224	2.578249
H	7.801372	7.332601	5.914339
H	17.522447	-3.169036	3.535183
H	8.870308	8.198744	3.195281
H	8.508819	4.923159	-1.334663
H	12.235718	5.590871	0.764662
H	6.061949	5.151132	4.480989
H	15.128374	-3.716265	3.998299
H	18.143812	-0.877454	2.813645
H	9.081486	6.682645	1.503208
H	10.395491	-2.361893	4.277253
H	7.937697	3.852505	4.515363
H	10.076118	2.532941	1.074972
H	16.369121	0.862206	2.553841
H	11.688041	2.233672	2.829149
H	11.448036	4.068721	-1.804865
H	11.737833	3.296677	-0.239797
H	7.298308	8.611414	3.895221
H	12.000839	6.267448	-0.861711
H	5.669630	6.879379	4.585571
H	9.804452	5.866628	-2.104433
H	9.373142	6.788275	5.286460
Si	7.380352	0.764064	3.610802
N	7.480944	0.120946	1.683363
C	7.661298	-1.183147	1.387876
C	7.367485	0.981378	0.650293
C	7.429098	0.591455	-0.666232
C	7.615610	-0.772345	-0.991682
C	7.730312	-1.662452	0.101583
H	7.748448	-1.855047	2.235322
H	7.881559	-2.724781	-0.040040
H	7.223887	2.024121	0.913500
H	7.321896	1.351403	-1.429411
N	7.680854	-1.201619	-2.277307
C	7.849652	-2.619539	-2.567148
H	8.794187	-3.004849	-2.159867
H	7.863864	-2.765236	-3.647766
H	7.024203	-3.215705	-2.154756
C	7.563704	-0.248726	-3.373322
H	8.353980	0.512715	-3.328933
H	6.589880	0.259790	-3.365227
H	7.660475	-0.779107	-4.321215
C	6.128496	1.861048	6.040787
N	7.280877	1.398742	5.515169
H	5.264329	1.866626	5.384769
H	8.641593	1.660233	10.436347
H	5.030271	2.460223	9.983478
C	6.014575	2.310197	7.334051
C	8.364000	1.379855	6.317935
H	5.049172	2.667623	7.667692
H	9.285309	1.009925	5.880302
C	7.141279	2.291139	8.190671
C	5.817385	3.225322	10.012939
C	8.260582	2.683646	10.321272
N	7.074126	2.718606	9.474317
C	8.342278	1.798694	7.626108
H	5.468358	4.106595	9.458310
H	8.004877	3.061805	11.311413
H	9.260824	1.743869	8.195626
H	9.064361	3.311565	9.914151
H	5.965453	3.517351	11.052926

1-OPEt$_3$

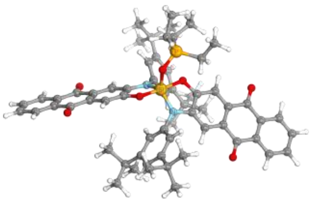

142
xyz, charge: 0, multiplicity: 1

C	-1.108969	3.778148	1.457735
C	-1.348958	2.707131	2.319634
C	0.132266	3.920504	0.852043
C	1.144374	2.993137	1.102442
C	0.903126	1.916225	1.969572
C	-0.347266	1.780041	2.574001
H	-1.894265	4.502354	1.260046
H	-2.320667	2.598385	2.793103
H	0.345016	4.744882	0.178579
H	-0.508072	0.938175	3.240186
C	2.469685	3.169104	0.444119
C	3.509180	2.163156	0.722271
C	3.277798	1.099262	1.618112
C	1.961377	0.907197	2.255097
C	4.766718	2.319785	0.122403
C	5.765623	1.418398	0.420613
C	5.533128	0.336729	1.310818
C	4.296296	0.182637	1.912791
H	4.095969	-0.621479	2.613242
H	4.948603	3.158375	-0.541061
O	2.676160	4.127830	-0.295626
O	1.732427	-0.045049	2.996046
O	7.018419	1.486274	-0.045127
N	6.683064	-0.420989	1.480127
C	6.713707	-1.474237	2.439844
C	6.011211	-2.655502	2.194207
C	6.053651	-3.699626	3.113097
C	6.814353	-3.525955	4.279116
C	7.513186	-2.351151	4.548681
C	7.447003	-1.319319	3.605486
H	7.974404	-0.382248	3.765331
H	6.850167	-4.336109	4.998033
H	5.431913	-2.725808	1.279346
C	5.300379	-5.013626	2.894457
C	8.319183	-2.140039	5.832924
C	9.784717	-1.825836	5.478124
H	10.237876	-2.648594	4.914830
H	10.368553	-1.674280	6.393489
H	9.873469	-0.919062	4.871983
C	7.716660	-0.952409	6.608744
H	7.763781	-0.025978	6.028045
H	8.270395	-0.792097	7.540947
H	6.666918	-1.142262	6.857281
C	8.298191	-3.372113	6.744776
H	8.734767	-4.248001	6.252153
H	7.281841	-3.621892	7.069087
H	8.890892	-3.168237	7.642524
C	6.298846	-6.186438	2.909829
H	7.051723	-6.063951	2.122689
H	5.773442	-7.133575	2.740903
H	6.825423	-6.261093	3.865763
C	4.552779	-5.039264	1.556137
H	5.239539	-4.929896	0.708006
H	3.798041	-4.247827	1.496417
H	4.037319	-5.998826	1.443780
C	4.270139	-5.200274	4.024770
H	4.752808	-5.245579	5.005700
H	3.712411	-6.132824	3.879530
H	3.557073	-4.369270	4.037929
H	11.163300	4.995416	-1.236527
H	6.297611	6.866083	2.793218
H	5.097857	4.714242	2.050992
H	8.894726	7.578988	0.323425
H	10.511260	7.395690	2.316409
H	6.969539	3.150334	4.926663
C	13.458306	1.030135	3.872995
C	11.191308	-1.537794	2.239595
C	10.232229	-0.701375	1.716183
C	9.029258	2.732167	1.501773
C	10.316012	0.707023	1.861266
C	11.364821	1.261744	2.580399
C	12.341514	0.416982	3.123023
C	14.464511	0.115586	4.481982
C	15.518411	0.659237	5.217367
C	14.370092	-1.276248	4.323842
C	15.329997	-2.105770	4.904948
C	9.845228	3.682706	0.891426
C	12.264923	-0.979299	2.950170
C	13.258018	-1.886706	3.544531
C	16.468947	-0.173239	5.792847
C	16.374520	-1.556717	5.636580
C	7.780803	4.467807	2.630543
C	6.928886	4.243252	4.970874
C	8.017256	3.114555	2.367220
C	10.527091	6.124892	0.527022
C	5.312945	4.311343	3.046334
C	6.519543	6.375472	3.747296
C	11.231107	6.908935	1.651365
C	11.604040	5.545870	-0.397925
C	9.648001	7.084494	-0.297168
C	6.649225	4.856686	3.585153
C	8.608592	5.403584	2.014761
C	9.649564	5.036239	1.149086
N	9.246162	1.338971	1.255768
O	13.180505	-3.107039	3.412049
O	9.146633	-1.109747	1.043033
O	13.562134	2.245891	3.996842
H	6.133994	4.520283	5.672958
H	17.119307	-2.205954	6.088158
H	7.429058	6.819738	4.166938
H	9.124850	6.543343	-1.092857
H	11.852434	6.241183	2.257806
H	5.318698	3.219753	2.967676
H	15.234033	-3.178439	4.768929
H	17.286858	0.253878	6.366108
H	8.448200	6.457328	2.210552
H	11.108641	-2.617126	2.155897
H	7.403688	2.342673	2.825206
H	10.625135	3.337885	0.221339
H	15.569363	1.738306	5.324058
H	11.446068	2.332463	2.735251
H	12.203271	6.362346	-0.814548
H	12.283793	4.877041	0.141440
H	5.695423	6.597782	4.433152
H	11.874522	7.687071	1.224195
H	4.493237	4.593840	3.717290
H	10.267737	7.863124	-0.757054
H	7.884284	4.601552	5.369444
Si	8.053286	0.222211	0.563554
O	8.198124	-0.352366	-1.098799
P	8.821607	-1.672920	-1.668004
C	8.018417	-3.141092	-0.941298
C	10.637574	-1.698786	-1.464226
C	8.399182	-1.581211	-3.441929
C	8.832506	-4.436394	-1.006886
H	7.049812	-3.248203	-1.447926
H	7.810512	-2.882428	0.103373
H	9.056653	-4.744915	-2.031768
H	8.261848	-5.241497	-0.535452
H	9.773748	-4.340164	-0.458129
C	8.408510	-2.896147	-4.226136
H	9.072472	-0.840555	-3.892624
H	7.397433	-1.132924	-3.456046
H	9.394063	-3.366873	-4.246482
H	8.113960	-2.701256	-5.261157
H	7.694345	-3.614606	-3.813857
C	11.438446	-2.430498	-2.544309
H	10.853202	-2.103245	-0.470630
H	10.919644	-0.637418	-1.424537
H	11.202457	-3.497314	-2.581394
H	12.503787	-2.339758	-2.314430
H	11.278700	-2.003896	-3.538526

1-(OPEt$_3$)$_2$

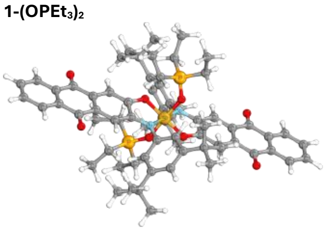

165
xyz, charge: 0, multiplicity: 1

C	-1.493098	2.670264	1.880151
C	-1.741508	1.404967	2.413502
C	-0.187082	3.101949	1.689198
C	0.882502	2.272576	2.028358
C	0.632529	0.999713	2.564728
C	-0.682770	0.573489	2.753903
H	-2.322533	3.319377	1.613420
H	-2.764000	1.069221	2.562295
H	0.033827	4.081641	1.276984
H	-0.847974	-0.415859	3.169139
C	2.276323	2.761930	1.819836
C	3.374261	1.854510	2.176921
C	3.126687	0.580164	2.733840
C	1.752221	0.086190	2.930908
C	4.694020	2.280068	1.971772
C	5.737335	1.446679	2.320111
C	5.499962	0.165061	2.888923
C	4.189346	-0.256188	3.088894
H	3.965134	-1.229749	3.513120
H	4.883922	3.250399	1.525872
O	2.477775	3.889112	1.369783
O	1.519615	-1.036650	3.373759
O	7.025001	1.755282	2.144732
N	6.668282	-0.498527	3.201891
C	6.670781	-1.909602	3.202988
C	5.972617	-2.614814	2.206172
C	5.954326	-4.003676	2.185062
C	6.681097	-4.697969	3.166915
C	7.393396	-4.027667	4.157750
C	7.366962	-2.629827	4.168703
H	7.912912	-2.081743	4.923622
H	6.669958	-5.781404	3.157601
H	5.432501	-2.039909	1.462715
C	5.138614	-4.792647	1.156724
C	8.179761	-4.756558	5.251501
C	9.646121	-4.286421	5.231598
H	10.112172	-4.496407	4.262463
H	10.225963	-4.803012	6.004862
H	9.726478	-3.210571	5.416388
C	7.561022	-4.421084	6.622402
H	7.600812	-3.345331	6.824169
H	8.104698	-4.937006	7.422723
H	6.511093	-4.730354	6.659977
C	8.160620	-6.278710	5.073500
H	8.598360	-6.577663	4.114567
H	7.143858	-6.681723	5.132392
H	8.749316	-6.747724	5.868953
C	6.036873	-5.810810	0.431307
H	6.849546	-5.302051	-0.099570
H	5.451429	-6.374264	-0.304286
H	6.485490	-6.530876	1.121886
C	4.494714	-3.885499	0.101943
H	5.248653	-3.324773	-0.463701
H	3.795567	-3.171476	0.549659
H	3.932003	-4.496793	-0.611429
C	4.013731	-5.542163	1.897980
H	4.418526	-6.244795	2.633164
H	3.401575	-6.108801	1.186431
H	3.364634	-4.835743	2.426110
H	11.187089	3.128042	-1.697251
H	7.450250	7.711468	1.199858
H	5.957662	5.736571	1.815930
H	9.514216	6.509112	-1.567637
H	11.495629	7.124840	-0.044105
H	8.116459	5.573096	4.909837
C	14.500106	1.222926	2.688013
C	11.594736	-0.964544	3.771952
C	10.541461	-0.127267	3.482737
C	9.526701	3.096100	2.099459
C	10.745179	1.153904	2.878191
C	12.059856	1.591038	2.680553
C	13.129191	0.739788	2.966645
C	15.629468	0.300264	2.991045
C	16.937196	0.728003	2.757685
C	15.396593	-0.988894	3.497649
C	16.476172	-1.831803	3.763230
C	10.258161	3.429362	0.954343
C	12.909957	-0.549759	3.495611
C	14.012159	-1.476495	3.758253
C	18.005896	-0.116415	3.027974
C	17.774687	-1.397231	3.530816

C 8.659502 5.357999 2.201775
C 8.183701 6.523461 4.369101
C 8.735990 4.060439 2.711513
C 11.030129 5.119299 -0.801344
C 6.294735 5.850212 2.851441
C 7.787201 7.751356 2.241194
C 12.017301 6.238074 -0.416957
C 11.838669 3.948656 -1.374684
C 10.075691 5.629771 -1.897199
C 7.745029 6.368018 2.899978
C 9.414701 5.671131 1.073845
C 10.221233 4.718010 0.434998
N 9.567178 1.786566 2.638213
O 13.820103 -2.619482 4.177585
O 9.258040 -0.427037 3.738505
O 14.710005 2.337525 2.219640
H 7.541383 7.248231 4.883140
H 18.611950 -2.056684 3.741474
H 8.794246 8.182609 2.265829
H 9.352707 4.854304 -2.172363
H 12.703476 5.895011 0.364862
H 6.194430 4.874604 3.337962
H 16.269896 -2.823700 4.153112
H 19.022441 0.221043 2.846941
H 9.377471 6.676448 0.670458
H 11.429568 -1.943680 4.211277
H 8.148147 3.766149 3.573089
H 10.852992 2.660808 0.477201
H 17.089661 1.728132 2.364039
H 12.277461 2.578835 2.290843
H 12.400108 4.288891 -2.251290
H 12.561524 3.559232 -0.648645
H 7.118931 8.432700 2.778425
H 12.609890 6.537194 -1.289589
H 5.620530 6.553061 3.354660
H 10.642224 5.909525 -2.793193
H 9.220199 6.873092 4.430673
Si 8.110902 0.644218 2.919785
O 8.445042 -0.192147 1.264478
P 9.252964 -1.262473 0.509324
C 9.510790 -2.787166 1.477880
C 10.862365 -0.558773 -0.010329
C 8.256102 -1.666394 -0.968609
C 10.744354 -3.617639 1.116396
H 8.586018 -3.374869 1.398764
H 9.559780 -2.457757 2.520770
H 10.725051 -3.975739 0.083038
H 10.786135 -4.496570 1.766550
H 11.669272 -3.054912 1.275972
C 8.561551 -2.987798 -1.676145
H 8.333198 -0.812051 -1.653492
H 7.226093 -1.665599 -0.587837
H 9.565141 -3.010386 -2.107746
H 7.846374 -3.142474 -2.489680
H 8.466804 -3.835728 -0.992208
C 11.512751 -1.127846 -1.273300
H 11.541046 -0.629531 0.848864
H 10.646503 0.509268 -0.143115
H 11.758646 -2.187938 -1.173991
H 12.445909 -0.590666 -1.467619
H 10.872446 -1.001513 -2.151248
H 7.212762 -0.496521 6.333492
H 7.886787 -1.092177 8.668025
H 7.053000 0.460581 8.658019
C 7.958772 -0.055203 8.326401
C 8.091781 -0.032843 6.801559
H 6.142909 2.226373 7.035603
H 8.963673 -0.606914 6.462291
O 7.684755 1.533424 4.546395
C 7.073568 2.773368 6.835181
H 8.816563 0.398536 8.828905
P 8.197229 1.603000 5.992587
C 7.593345 3.477086 8.090376
H 7.834751 2.776614 8.893982
H 6.827801 4.161577 8.467180
H 10.821383 0.872081 7.504205
C 9.909579 2.233481 6.066945
H 8.485780 4.071890 7.874711
C 10.702766 1.947855 7.344866
H 10.419866 1.780573 5.208834
H 10.243719 2.381787 8.236942
H 9.857604 3.309642 5.851179
H 11.705775 2.372801 7.246528
H 6.828915 3.508968 6.057254

1-HMPA

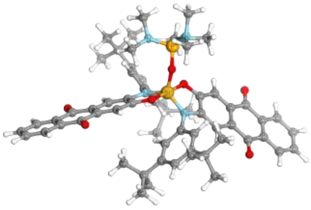

148
xyz, charge: 0, multiplicity: 1
C -1.446351 3.098517 1.717122
C -1.662012 2.072221 2.637704
C -0.213650 3.224989 1.090767
C 0.814350 2.326762 1.379826
C 0.597627 1.294681 2.306516
C -0.644482 1.174053 2.930606
H -2.243976 3.800133 1.489664
H -2.627133 1.975491 3.126894
H -0.020254 4.014418 0.371189
H -0.786585 0.367229 3.642808
C 2.128555 2.481695 0.696687
C 3.184401 1.511532 1.024561
C 2.981322 0.490084 1.974374
C 1.673112 0.317159 2.637284
C 4.433533 1.651152 0.401640
C 5.444808 0.775206 0.727499
C 5.243833 -0.261331 1.674633
C 4.015080 -0.396741 2.302174
H 3.835191 -1.167819 3.043834
H 4.591248 2.466911 -0.296239
O 2.313125 3.393571 -0.107811
O 1.465938 -0.593295 3.433295
O 6.687853 0.820166 0.230770
N 6.401665 -0.995049 1.858097
C 6.453838 -1.974013 2.899039
C 5.759397 -3.174204 2.755071
C 5.792632 -4.125054 3.770213
C 6.551623 -3.845543 4.916395
C 7.253116 -2.653055 5.077638
C 7.181732 -1.708781 4.047547
H 7.703998 -0.758433 4.124422
H 6.587064 -4.587742 5.705275
H 5.199741 -3.337227 1.840432
C 5.033439 -5.450727 3.677812
C 8.068532 -2.332269 6.333039
C 9.529248 -2.040943 5.939978
H 9.980265 -2.902746 5.436693
H 10.121777 -1.816573 6.834669
H 9.607527 -1.183917 5.264058
C 7.465810 -1.089241 7.015856
H 7.496745 -0.214434 6.359061
H 8.029303 -0.847272 7.924515
H 6.420916 -1.266476 7.292845
C 8.064102 -3.486200 7.342221
H 8.498611 -4.397208 6.916166
H 7.052759 -3.712916 7.697626
H 8.666284 -3.208062 8.213486
C 6.029323 -6.621106 3.784093
H 6.770130 -6.572839 2.978705
H 5.498127 -7.577236 3.709459
H 6.568127 -6.610428 4.736328
C 4.266438 -5.590596 2.357633
H 4.940378 -5.567022 1.494017
H 3.518132 -4.799433 2.238511
H 3.739915 -6.550746 2.342268
C 4.017677 -5.532428 4.833579
H 4.511801 -5.494801 5.809180
H 3.455086 -6.471710 4.776899
H 3.307266 -4.700345 4.782917
H 11.380587 3.467588 -1.710344
H 6.624352 6.801995 1.421041
H 5.183394 4.677831 1.199709
H 9.361576 6.571749 -1.014762
H 10.879532 6.762800 1.060165
H 6.837116 3.701807 4.447757
C 13.299260 0.662639 3.892726
C 10.907378 -2.013336 2.658403
C 9.947418 -1.216961 2.071864
C 8.904271 2.207731 1.354735
C 10.066412 0.197552 2.069657
C 11.157045 0.801944 2.673676
C 12.134434 -0.001190 3.276560
C 14.300959 -0.200020 4.579499
C 15.397255 0.396037 5.204149
C 14.160412 -1.596479 4.601867
C 15.117448 -2.378327 5.249796
C 9.851394 2.893030 0.590866
C 12.013271 -1.405764 3.269684
C 13.000411 -2.263617 3.946519
C 16.344539 -0.389053 5.847568
C 16.204537 -1.777335 5.870215
C 7.824709 4.294308 1.933402
C 6.919968 4.767359 4.213089
C 7.909213 2.899621 2.026658
C 10.817084 5.073948 -0.338054
C 5.345953 4.516154 2.270678
C 6.772025 6.548064 2.476634
C 11.564255 6.050804 0.589749
C 11.854353 4.168546 -1.012349
C 10.083053 5.865822 -1.436499
C 6.729217 5.032989 2.706916
C 8.772911 4.956073 1.155981
C 9.799575 4.277757 0.482152
N 8.978566 0.787018 1.443202
O 12.879798 -3.485746 3.983903
O 8.840918 -1.671474 1.475426
O 13.450496 1.880442 3.846287
H 6.153116 5.298422 4.788662
H 16.946924 -2.389606 6.374378
H 7.718351 6.985498 2.813782
H 9.537727 5.187737 -2.103385
H 12.083502 5.506149 1.385350
H 5.228702 3.446540 2.466546
H 14.984867 -3.455737 5.253552
H 17.195374 0.078864 6.334706
H 8.726955 6.035773 1.075340
H 10.805143 -3.093171 2.674652
H 7.195503 2.335802 2.621201
H 10.622812 2.313264 0.094581
H 15.482932 1.477711 5.171963
H 11.273012 1.880219 2.700893
H 12.558329 4.780746 -1.585728
H 12.431456 3.594741 -0.279071
H 5.966896 7.023365 3.046389
H 12.306128 6.622548 0.020059
H 4.556443 5.044282 2.817478
H 10.800638 6.436430 -2.037555
H 7.904640 5.111626 4.547384
Si 7.770992 -0.401069 0.903377
O 7.997459 -1.044942 -0.723862
P 8.282194 -0.478125 -2.134085
N 7.785641 -1.708946 -3.142971
N 7.491102 0.853705 -2.689539
N 9.888052 -0.055672 -2.160709
C 7.777859 -3.104151 -2.681438
H 7.023103 -3.655199 -3.254469
H 7.518712 -3.156091 -1.624098
H 8.752469 -3.591611 -2.834563
C 8.033395 -1.579335 -4.582162
H 7.260311 -2.131125 -5.130423
H 9.014235 -1.987825 -4.870434
H 7.983394 -0.531646 -4.890403
C 10.497988 0.579818 -3.331548
H 11.226505 1.330472 -2.998744
H 9.746098 1.082132 -3.943628
H 11.028249 -0.155184 -3.954697
C 10.866812 -0.735627 -1.299503
H 10.371267 -1.286386 -0.500056
H 11.532290 0.007353 -0.842024
H 11.476931 -1.438066 -1.884778
C 7.881512 2.215506 -2.280791
H 7.225649 2.576522 -1.480597
H 7.805108 2.882091 -3.148925
H 8.902768 2.235642 -1.899900
C 6.065224 0.770442 -3.050278
H 5.803182 -0.240425 -3.366101
H 5.869814 1.463547 -3.876960
H 5.435606 1.048740 -2.196339

1-(HMPA)$_2$

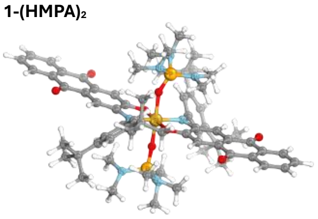

177

xyz, charge: 0, multiplicity: 1

C	-2.380039	3.616201	2.709710
C	-2.698909	2.324124	3.128598
C	-1.052563	3.984453	2.532656
C	-0.031487	3.064741	2.773343
C	-0.352577	1.764266	3.195193
C	-1.688830	1.402120	3.369810
H	-3.171352	4.336373	2.521217
H	-3.737901	2.038138	3.265843
H	-0.778072	4.983021	2.207096
H	-1.909643	0.390252	3.695193
C	1.386151	3.484165	2.577709
C	2.427550	2.493646	2.857510
C	2.114814	1.180280	3.267289
C	0.712756	0.756051	3.457860
C	3.773831	2.863651	2.701276
C	4.768647	1.942784	2.945407
C	4.467186	0.606721	3.338834
C	3.126545	0.248741	3.511867
H	2.842045	-0.747425	3.832835
H	4.009665	3.877872	2.397281
O	1.652540	4.625503	2.196998
O	0.421936	-0.381588	3.817468
O	6.073801	2.214214	2.854491
N	5.595160	-0.128706	3.556394
C	5.488780	-1.523741	3.777127
C	4.689154	-2.305439	2.932419
C	4.539117	-3.668621	3.152019
C	5.239510	-4.250686	4.219041
C	6.053242	-3.498992	5.062784
C	6.159560	-2.123383	4.834795
H	6.774844	-1.498915	5.473114
H	5.136869	-5.315796	4.390559
H	4.182285	-1.813747	2.109868
C	3.646532	-4.543103	2.267148
C	6.831229	-4.117454	6.226942
C	8.338652	-3.926936	5.974258
H	8.643754	-4.438448	5.055245
H	8.924787	-4.336765	6.805258
H	8.596502	-2.870250	5.863041
C	6.431803	-3.411363	7.537359
H	6.630690	-2.335823	7.491934
H	6.996220	-3.827317	8.380685
H	5.362725	-3.545951	7.737247
C	6.555759	-5.616218	6.392336
H	6.861336	-6.183538	5.506670
H	5.495737	-5.815298	6.585631
H	7.128663	-6.000312	7.243166
C	4.522307	-5.567431	1.521754
H	5.262184	-5.058083	0.893614
H	3.903104	-6.204979	0.879448
H	5.065098	-6.213399	2.218951
C	2.867198	-3.720854	1.233506
H	3.535049	-3.202753	0.536884
H	2.222819	-2.975454	1.713176
H	2.228279	-4.387049	0.643953
C	2.620719	-5.285785	3.144659
H	3.104813	-5.948551	3.867954
H	1.963178	-5.899261	2.517685
H	2.001098	-4.572891	3.699072
H	11.182786	3.338798	-0.921483
H	7.173608	8.060300	1.612026
H	5.468873	6.169078	1.941263
H	9.415335	6.665606	-1.078105
H	11.119505	7.352061	0.723719
H	7.182629	5.695149	5.239395
C	13.503916	1.189217	3.645656
C	10.469956	-1.076255	3.843643
C	9.480193	-0.182798	3.493599
C	8.764261	3.241682	2.450470
C	9.780664	1.174826	3.172355
C	11.105005	1.603549	3.261220
C	12.112131	0.700701	3.614129
C	14.564942	0.220467	4.041274
C	15.891145	0.650559	4.100906
C	14.249924	-1.113590	4.344741
C	15.266536	-1.998745	4.704377
C	9.668470	3.592513	1.435810
C	11.805087	-0.646435	3.899241
C	12.841812	-1.604497	4.295447
C	16.896966	-0.236977	4.460106
C	16.584044	-1.562885	4.761469
C	8.072035	5.566144	2.605401
C	7.418411	6.656594	4.771010
C	7.997259	4.240422	3.044848
C	10.814279	5.320389	-0.059560
C	5.691748	6.216044	3.013117
C	7.382551	8.019634	2.686554
C	11.702409	6.460362	0.475429
C	11.737838	4.165004	-0.463969
C	10.053385	5.806092	-1.307446
C	7.159403	6.613293	3.253138
C	8.975885	5.882184	1.594355
C	9.805117	4.909150	1.016207
N	8.654028	1.889780	2.855768
O	12.579780	-2.771374	4.587421
O	8.182986	-0.501783	3.443535
O	13.794490	2.346935	3.352844
H	6.789106	7.422388	5.240034
H	17.372157	-2.256497	5.041122
H	8.407727	8.367609	2.855254
H	9.411728	5.009589	-1.702551
H	12.235683	6.141034	1.377029
H	5.475032	5.234991	3.446252
H	14.996313	-3.024703	4.934368
H	17.928166	0.101876	4.505607
H	9.056443	6.907151	1.252196
H	10.227723	-2.108945	4.073151
H	7.318395	3.966208	3.842652
H	10.265209	2.806749	0.991085
H	16.107366	1.686754	3.859956
H	11.385533	2.631962	3.063309
H	12.464303	4.518528	-1.203344
H	12.294728	3.772491	0.394378
H	6.705336	8.723922	3.181270
H	12.443461	6.745868	-0.280141
H	5.012885	6.945773	3.469510
H	10.756427	6.104602	-2.094222
H	8.467275	6.895195	4.980290
Si	7.130727	0.857008	3.164347
O	7.003919	0.290206	1.373750
P	6.848050	0.724656	-0.077585
N	8.365925	1.119335	-0.657811
N	5.910977	2.021932	-0.474223
N	6.109785	-0.578329	-0.834572
C	9.506752	0.301010	-0.226572
H	9.348080	-0.084241	0.781392
H	10.414544	0.916553	-0.215767
H	9.678084	-0.544223	-0.911512
C	8.552121	1.690619	-1.992189
H	7.666788	2.247555	-2.305490
H	8.765337	0.915459	-2.744417
H	9.402082	2.384604	-1.972256
C	6.352033	-1.931916	-0.315605
H	6.378332	-1.926903	0.774702
H	7.294753	-2.356788	-0.695425
H	5.531383	-2.581947	-0.642048
C	5.983983	-0.550047	-2.294207
H	5.125690	-1.164450	-2.593630
H	6.879605	-0.949739	-2.796057
H	5.809984	0.468736	-2.650978
C	6.370162	3.396080	-0.215734
H	6.110931	4.032137	-1.072445
H	7.450436	3.429076	-0.059275
H	5.892006	3.796052	0.686201
C	4.455990	1.923765	-0.658327
H	4.138971	0.880252	-0.691980
H	4.172431	2.415829	-1.598637
H	3.931072	2.417077	0.168063
H	3.830366	3.327174	7.102766
H	7.131118	4.983351	7.822455
H	6.147148	1.092004	9.046810
H	5.017962	-0.275220	7.137395
H	8.977901	4.548914	6.206971
H	8.694575	-1.143859	7.632368
P	6.836995	1.681266	6.396913
C	4.429754	2.955478	6.260424
C	7.077571	3.898179	7.978242
C	4.392351	0.594014	6.928203
C	8.671738	-0.157391	7.154566
C	7.097057	0.581448	8.869220
C	8.833226	3.471707	6.342431
N	7.371704	0.473621	7.433213
N	7.431705	3.219852	6.727091
N	5.215988	1.787146	6.683440
O	7.348269	1.322149	5.016874
H	8.814489	-0.287756	6.080877
H	9.533729	3.123659	7.117628
H	3.731259	0.775143	7.786246
H	7.028353	-0.426391	9.298633
H	5.082867	3.757812	5.909488
H	6.060024	3.647363	8.285383
H	3.752663	2.682417	5.442363
H	7.893392	1.123733	9.402292
H	7.765380	3.639966	8.798323
H	3.773890	0.371301	6.049745
H	9.069259	2.976633	5.399255
H	9.511000	0.433269	7.552907

1-DMSO

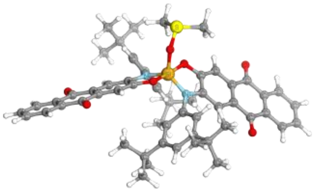

129

xyz, charge: 0, multiplicity: 1

C	-1.157618	3.305286	0.978723
C	-1.430581	2.337795	1.946685
C	0.096357	3.363702	0.385704
C	1.088876	2.455329	0.755895
C	0.814539	1.482446	1.730485
C	-0.448957	1.429986	2.320513
H	-1.927508	4.014429	0.688126
H	-2.412480	2.294508	2.409251
H	0.333529	4.106643	-0.369328
H	-0.636231	0.667992	3.070599
C	2.426234	2.537360	0.107746
C	3.445355	1.560518	0.523194
C	3.184449	0.596154	1.516149
C	1.849693	0.496100	2.147022
C	4.721227	1.641346	-0.055604
C	5.693996	0.762376	0.359842
C	5.434187	-0.227764	1.339615
C	4.179698	-0.298649	1.927746
H	3.952147	-1.024716	2.700936
H	4.929413	2.424078	-0.778694
O	2.662944	3.396270	-0.739524
O	1.596255	-0.363394	2.983786
O	6.962995	0.757898	-0.086945
N	6.563862	-0.981687	1.595592
C	6.575670	-1.890578	2.700535
C	5.821203	-3.061160	2.649169
C	5.822013	-3.936548	3.730892
C	6.611696	-3.614862	4.844994
C	7.375836	-2.452233	4.911232
C	7.334366	-1.581195	3.817706
H	7.907365	-0.657202	3.820670
H	6.623007	-4.300371	5.684236
H	5.245080	-3.265192	1.753350
C	4.999176	-5.226851	3.741544
C	8.237559	-2.088574	6.122913
C	9.705061	-1.933906	5.679192
H	10.079809	-2.861278	5.233094
H	10.335074	-1.686972	6.541704
H	9.826211	-1.137163	4.938596
C	7.739801	-0.754594	6.712210
H	7.821463	0.061741	5.987639
H	8.338290	-0.483527	7.589592
H	6.690911	-0.831813	7.018569
C	8.176025	-3.154578	7.222691
H	8.543142	-4.123657	6.867359
H	7.158235	-3.284746	7.607126
H	8.810150	-2.847825	8.060975
C	5.941735	-6.433685	3.909575
H	6.667814	-6.474887	3.090609

H 5.365011 -7.365953 3.908236
H 6.498913 -6.385580 4.850041
C 4.199692 -5.417100 2.447238
H 4.855879 -5.486731 1.572627
H 3.488077 -4.599540 2.287159
H 3.626819 -6.348441 2.506942
C 4.004781 -5.182417 4.917710
H 4.519489 -5.104052 5.880149
H 3.398441 -6.095573 4.933938
H 3.332209 -4.323051 4.823486
H 11.431155 3.945098 -1.474244
H 6.379269 6.490253 1.932885
H 5.119474 4.293066 1.445127
H 9.184431 6.791308 -0.425247
H 10.654271 6.806767 1.691068
H 6.822125 3.085891 4.584867
C 13.506065 0.465092 3.703891
C 11.094083 -2.143719 2.366080
C 10.134997 -1.317165 1.821993
C 9.013638 2.131646 1.363479
C 10.267337 0.094921 1.873620
C 11.360645 0.667581 2.500422
C 12.337115 -0.166332 3.060652
C 14.514068 -0.431707 4.334120
C 15.621019 0.131097 4.970923
C 14.369238 -1.827315 4.291844
C 15.332773 -2.641728 4.888042
C 9.913792 2.971309 0.703644
C 12.208541 -1.568128 2.991010
C 13.197662 -2.459949 3.623375
C 16.575053 -0.686328 5.561628
C 16.430876 -2.073826 5.519886
C 7.774600 4.047144 2.172635
C 6.820403 4.175083 4.477211
C 7.962906 2.662685 2.096077
C 10.731016 5.316742 0.082117
C 5.284032 4.025863 2.494500
C 6.536852 6.129191 2.955270
C 11.393741 6.210581 1.147987
C 11.839600 4.581499 -0.680123
C 9.958246 6.196046 -0.918973
C 6.617524 4.599305 3.009392
C 8.680594 4.866261 1.502111
C 9.761134 4.352754 0.769306
N 9.171978 0.718026 1.286425
O 13.067572 -3.680744 3.613228
O 8.996429 -1.744074 1.257608
O 13.653256 1.683909 3.723313
H 6.010010 4.572175 5.099207
H 17.178960 -2.711401 5.982502
H 7.440517 6.599805 3.358121
H 9.470566 5.578144 -1.681401
H 11.938964 5.602232 1.877315
H 5.251474 2.934701 2.569866
H 15.196865 -3.717745 4.842335
H 17.434827 -0.244532 6.057345
H 8.556517 5.941539 1.554024
H 10.982323 -3.222350 2.343677
H 7.289952 1.980365 2.609477
H 10.736159 2.518765 0.159845
H 15.709869 1.212821 4.989254
H 11.478845 1.742915 2.581610
H 12.504190 5.312182 -1.152670
H 12.449011 3.961219 -0.013971
H 5.690121 6.469015 3.560495
H 12.102010 6.900922 0.675362
H 4.450984 4.425203 3.084501
H 10.643666 6.887542 -1.422477
H 7.772206 4.554771 4.864056
Si 7.988523 -0.448815 0.688461
O 8.391803 -1.113893 -0.964555
S 8.294110 -0.518786 -2.426656
C 6.541389 -0.209936 -2.826354
H 6.502513 -0.043305 -3.907981
H 6.146134 0.635887 -2.265629
H 6.001019 -1.127393 -2.577339
C 8.921657 1.186718 -2.340288
H 9.971166 1.118548 -2.042712
H 8.341701 1.764061 -1.616457
H 8.846097 1.601786 -3.350777

1-(DMSO)$_2$

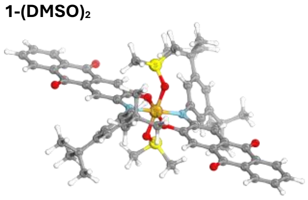

139
xyz, charge: 0, multiplicity: 1
C -2.201234 3.499002 1.356363
C -2.526258 2.196341 1.736480
C -0.878712 3.922044 1.377511
C 0.130768 3.047047 1.779797
C -0.196281 1.736214 2.161893
C -1.527531 1.318330 2.135947
H -2.983901 4.184305 1.043196
H -3.561661 1.867939 1.719833
H -0.599458 4.929981 1.086771
H -1.753237 0.299308 2.434390
C 1.542679 3.526999 1.799657
C 2.575970 2.576883 2.236173
C 2.249252 1.265592 2.637423
C 0.858734 0.773937 2.586029
C 3.910865 3.003151 2.282609
C 4.887791 2.124698 2.700764
C 4.577359 0.785708 3.072442
C 3.245019 0.379508 3.061915
H 2.944235 -0.610975 3.382701
H 4.164391 4.018117 1.995725
O 1.814527 4.678574 1.466439
O 0.568847 -0.385312 2.872171
O 6.178856 2.453361 2.806351
N 5.707760 0.082093 3.431375
C 5.594177 -1.259020 3.885255
C 4.890674 -2.201610 3.121482
C 4.767679 -3.519995 3.538930
C 5.401813 -3.902671 4.730115
C 6.123117 -2.994812 5.499705
C 6.187043 -1.664733 5.073425
H 6.739749 -0.935644 5.651730
H 5.325128 -4.933993 5.053824
H 4.432002 -1.878806 2.194660
C 3.967322 -4.553979 2.743115
C 6.864062 -3.396512 6.778163
C 8.370266 -3.135943 6.582145
H 8.760375 -3.728110 5.747592
H 8.927019 -3.409804 7.485984
H 8.572790 -2.082335 6.362785
C 6.351562 -2.554248 7.961181
H 6.542336 -1.487832 7.803101
H 6.860132 -2.849920 8.886218
H 5.273229 -2.687944 8.098743
C 6.669908 -4.876145 7.128305
H 7.053937 -5.531317 6.339201
H 5.614939 -5.117437 7.299461
H 7.218868 -5.109357 8.046760
C 4.888378 -5.720269 2.338696
H 5.721389 -5.360890 1.724273
H 4.327424 -6.463411 1.760005
H 5.311196 -6.225632 3.212166
C 3.352006 -3.961596 1.469397
H 4.123725 -3.594568 0.782025
H 2.661517 -3.140595 1.693250
H 2.785476 -4.737044 0.943232
C 2.819301 -5.085662 3.622711
H 3.196710 -5.567741 4.529669
H 2.228512 -5.824409 3.068579
H 2.155006 -4.268751 3.924039
H 10.683110 3.626482 -0.988217
H 7.185137 8.370381 1.916404
H 5.504431 6.491151 2.284522
H 9.259477 7.117945 -0.782179
H 11.192100 7.509466 0.874632
H 7.318078 6.010128 5.545879
C 13.618762 1.432713 3.607503
C 10.562465 -0.780455 4.009584
C 9.571549 0.133955 3.733679
C 8.812612 3.557909 2.713511
C 9.870535 1.482515 3.382559
C 11.206164 1.889553 3.373529
C 12.216743 0.966132 3.655696
C 14.685867 0.439983 3.914083
C 16.020626 0.846609 3.886547
C 14.368436 -0.892192 4.224030
C 15.391287 -1.799173 4.502865
C 9.623748 3.877256 1.618372
C 11.906273 -0.374134 3.965757
C 12.952667 -1.358597 4.264569
C 17.032287 -0.062578 4.165470
C 16.716968 -1.386458 4.473708
C 8.114399 5.873230 2.877851
C 7.537743 6.967696 5.062714
C 8.069827 4.553459 3.335808
C 10.622482 5.581502 -0.005872
C 5.751285 6.526321 3.350905
C 7.436965 8.326968 2.981333
C 11.666041 6.601976 0.488015
C 11.373195 4.380001 -0.592336
C 9.773549 6.214627 -1.124310
C 7.226897 6.921022 3.554676
C 8.952539 6.171980 1.806953
C 9.717683 5.186832 1.164297
N 8.735011 2.220916 3.179939
O 12.688791 -2.527562 4.543949
O 8.258852 -0.147325 3.803007
O 13.905514 2.592476 3.325067
H 6.925427 7.733570 5.552804
H 17.509867 -2.096579 4.691257
H 8.470678 8.667343 3.109345
H 9.013475 5.509935 -1.478687
H 12.277121 6.171250 1.288521
H 5.536025 5.540201 3.774576
H 15.119711 -2.823124 4.739934
H 18.070076 0.257724 4.143046
H 9.014660 7.193264 1.449726
H 10.318045 -1.805325 4.269395
H 7.415843 4.271823 4.153578
H 10.167767 3.076149 1.132115
H 16.238786 1.881717 3.642811
H 11.488659 2.911374 3.144697
H 12.008104 4.715799 -1.419140
H 12.021294 3.903669 0.151836
H 6.784518 9.035128 3.503057
H 12.330174 6.892537 -0.334523
H 5.089585 7.256164 3.831903
H 10.410839 6.491878 -1.972227
H 8.593389 7.204412 5.235927
Si 7.219083 1.168383 3.310713
O 7.507356 0.718984 1.452482
S 7.114590 -0.723711 0.959600
C 8.366300 -0.979397 -0.332232
H 8.121592 -1.890121 -0.886672
H 9.323843 -1.097015 0.181520
H 8.386722 -0.104734 -0.987479
C 5.665642 -0.420033 -0.100919
H 4.858992 -0.086144 0.559375
H 5.385978 -1.354485 -0.597176
H 5.913589 0.362527 -0.822877
H 9.606844 2.863778 7.352332
H 6.159434 0.945237 7.750571
S 7.966766 1.472624 6.300469
C 8.997342 2.971133 6.449369
C 6.919580 1.729813 7.763159
O 6.933819 1.772176 5.155709
H 7.539394 1.643873 8.660366
H 9.638502 3.014707 5.566199
H 8.351485 3.851102 6.503971
H 6.451299 2.714983 7.691527

1-DIBA

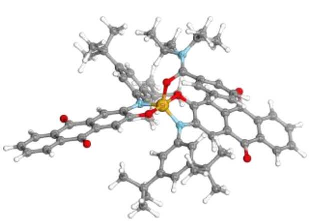

153
xyz, charge: 0, multiplicity: 1
C -1.517734 2.156618 0.940908
C -1.713115 1.080879 1.807980

C -0.250761 2.428454 0.442069
C 0.832658 1.629173 0.808781
C 0.636149 0.546687 1.681324
C -0.641130 0.278288 2.174903
H -2.357928 2.783413 0.655779
H -2.705107 0.870800 2.197646
H -0.072642 3.258610 -0.234312
H -0.767032 -0.564851 2.846956
C 2.182508 1.939437 0.261990
C 3.303649 1.092557 0.697586
C 3.115173 0.010905 1.582478
C 1.769689 -0.330933 2.088298
C 4.587826 1.383050 0.212236
C 5.643668 0.588893 0.601919
C 5.460407 -0.506059 1.483909
C 4.196115 -0.784793 1.981720
H 4.020010 -1.610999 2.662032
H 4.728663 2.221676 -0.461804
O 2.342086 2.870444 -0.525722
O 1.578884 -1.302482 2.812901
O 6.905082 0.736072 0.179071
N 6.652893 -1.164889 1.706045
C 6.703225 -2.236167 2.651501
C 6.030027 -3.427804 2.383474
C 6.053551 -4.463054 3.312800
C 6.789314 -4.280108 4.493026
C 7.476129 -3.101050 4.772434
C 7.409084 -2.067753 3.831335
H 7.916518 -1.121907 4.002660
H 6.818237 -5.088994 5.213635
H 5.495724 -3.516197 1.442929
H 5.305237 -5.780023 3.094024
C 8.267468 -2.887828 6.065490
C 9.729781 -2.542533 5.726085
H 10.204026 -3.349038 5.156984
H 10.303508 -2.390893 6.647836
H 9.806480 -1.627702 5.130736
C 7.636214 -1.721450 6.850262
H 7.664439 -0.789233 6.277812
H 8.182503 -1.557725 7.786422
H 6.590002 -1.937444 7.092844
C 8.263875 -4.130365 6.963412
H 8.716312 -4.992785 6.461636
H 7.250894 -4.400887 7.281751
H 8.850485 -3.926886 7.865326
C 6.307084 -6.949833 3.107149
H 7.057431 -6.825224 2.318837
H 5.783624 -7.898813 2.941352
H 6.834793 -7.020761 4.062922
C 4.556497 -5.804208 1.756765
H 5.245513 -5.698764 0.911931
H 3.809465 -5.004586 1.696268
H 4.033662 -6.760583 1.647570
C 4.274956 -5.970398 4.223698
H 4.755752 -6.017374 5.205428
H 3.719621 -6.904119 4.075928
H 3.559603 -5.141080 4.237569
H 12.345925 2.698525 -1.685410
H 7.414011 6.774222 0.091663
H 5.870937 4.716825 -0.020234
H 10.454079 5.962606 -1.864254
H 11.625686 6.439672 0.393872
H 6.945275 4.211381 3.560545
C 13.540104 0.517899 3.824238
C 11.159474 -2.127110 2.507423
C 10.208553 -1.315400 1.927406
C 9.281164 2.100985 1.063536
C 10.323534 0.096842 1.963335
C 11.408082 0.685604 2.593074
C 12.378657 -0.130873 3.186654
C 14.536900 -0.361261 4.497171
C 15.633455 0.219193 5.135956
C 14.391523 -1.757474 4.493089
C 15.344217 -2.554525 5.128891
C 10.367749 2.594518 0.330152
C 12.255825 -1.535282 3.147751
C 13.230266 -2.408413 3.823943
C 16.576792 -0.581030 5.766232
C 16.432112 -1.969062 5.762417
C 8.322857 4.320922 1.161359
C 7.142442 5.216994 3.177301
C 8.277985 2.959077 1.486253
C 11.664824 4.540627 -0.709779
C 5.853006 4.732341 1.075515
C 7.388567 6.690453 1.183952
C 12.314760 5.616494 0.182610
C 12.731156 3.486069 -1.027746
C 11.204623 5.183680 -2.031063
C 7.194051 5.239060 1.637434
C 9.415470 4.790000 0.436123
C 10.460173 3.947042 0.025983
N 9.229788 0.707469 1.357381
O 13.096766 -3.629380 3.851785
O 9.103305 -1.756816 1.316699
O 13.693831 1.736169 3.804518
H 6.343765 5.875853 3.537125
H 17.171745 -2.593242 6.255934
H 8.309118 7.124452 1.589572
H 10.767496 4.431958 -2.698792
H 12.629415 5.184424 1.138339
H 5.623197 3.720899 1.422274
H 15.207815 -3.631336 5.112287
H 17.428415 -0.125275 6.263436
H 9.474873 5.844484 0.193447
H 11.058876 -3.206867 2.490667
H 7.456031 2.552253 2.066626
H 11.140839 1.895830 0.028423
H 15.722753 1.300990 5.124269
H 11.526808 1.762582 2.645810
H 13.572806 3.961742 -1.541994
H 13.118900 3.016166 -0.117363
H 6.550921 7.297300 1.543049
H 13.197443 6.035671 -0.314102
H 5.034085 5.387671 1.393036
H 12.056433 5.640884 -2.547787
H 8.091920 5.560184 3.601998
Si 8.029851 -0.487489 0.810407
O 8.334116 -0.955821 -0.885795
C 7.453825 -0.868583 -1.839595
C 6.398655 -1.903220 -1.818919
N 7.567320 0.074606 -2.751186
C 6.908417 0.061789 -4.096808
C 5.462612 0.563382 -4.108915
C 7.064211 -1.279081 -4.809522
H 7.502985 0.790437 -4.655440
C 8.533097 1.195675 -2.506663
H 8.841654 1.102729 -1.466886
C 7.843115 2.547710 -2.646765
C 9.775958 1.048980 -3.378090
H 4.760135 -0.236660 -3.868004
H 5.226354 0.920224 -5.116538
H 5.309192 1.391342 -3.412350
H 6.429880 -2.053536 -4.372438
H 8.103980 -1.620395 -4.790079
H 6.765066 -1.151427 -5.854589
H 6.967247 2.600595 -1.994464
H 7.551431 2.780696 -3.676558
H 8.545030 3.317314 -2.309914
H 10.256824 0.079502 -3.215399
H 10.487397 1.831722 -3.097985
H 9.563687 1.165963 -4.446329
C 6.798607 -3.242166 -1.721227
C 5.842444 -4.238599 -1.602000
C 4.490927 -3.904677 -1.516915
C 4.098136 -2.571555 -1.556860
C 5.046819 -1.567510 -1.721177
H 4.742733 -0.527939 -1.699529
H 7.856026 -3.488198 -1.740868
H 6.152136 -5.277919 -1.551255
H 3.747000 -4.686092 -1.392073
H 3.052334 -2.303760 -1.441475

1-^{dipp}NHC

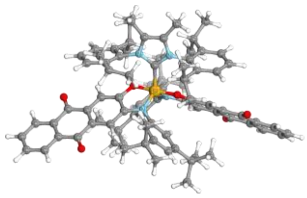

190
xyz, charge: 0, multiplicity: 1
C -1.656206 2.807171 -2.180373
C -2.069217 1.483221 -2.334767
C -0.638828 3.120121 -1.288770
C -0.023542 2.112009 -0.545662
C -0.438649 0.779663 -0.701488
C -1.463930 0.474379 -1.597357
H -2.130859 3.594795 -2.758793
H -2.865167 1.240583 -3.033139
H -0.299459 4.141852 -1.149716
H -1.767276 -0.563066 -1.698074
C 1.065764 2.470576 0.404737
C 1.706665 1.375887 1.146421
C 1.300256 0.035364 0.988475
C 0.193548 -0.321847 0.079204
C 2.765746 1.689657 2.008638
C 3.369402 0.679261 2.721416
C 2.959218 -0.669326 2.579852
C 1.934200 -0.989787 1.700392
H 3.086221 2.718943 2.103344
H 1.611885 -2.013968 1.546008
O 1.407699 3.643472 0.552716
O -0.201662 -1.477724 -0.039524
O 4.392214 0.844558 3.571893
N 3.726435 -1.503343 3.371547
C 3.597212 -2.917854 3.214285
C 4.654672 -3.635266 2.652881
C 4.565890 -5.012065 2.488167
C 3.368066 -5.644759 2.856266
C 2.281450 -4.941928 3.370279
C 2.422129 -3.562661 3.568635
H 1.607543 -2.976331 3.983458
H 3.286051 -6.716182 2.716552
H 5.544390 -3.091765 2.354916
C 5.721616 -5.841686 1.922875
C 6.895606 -4.966998 1.465685
H 6.597420 -4.269943 0.674410
H 7.690020 -5.605707 1.065160
H 7.322716 -4.392608 2.294106
C 6.228728 -6.800177 3.017848
H 6.598473 -6.242569 3.884893
H 7.052651 -7.412290 2.632362
H 5.436733 -7.474646 3.359592
C 5.232062 -6.657868 0.711486
H 4.837138 -5.996832 -0.067584
H 4.444435 -7.366751 0.984444
H 6.063554 -7.232349 0.287471
C 0.938652 -5.606751 3.685936
C -0.149859 -4.959720 2.806757
H -0.253699 -3.889143 3.009525
H -1.120312 -5.433982 2.994570
H 0.090242 -5.072781 1.744421
C 0.585522 -5.399522 5.169859
H 0.522324 -4.336674 5.425263
H 1.340693 -5.861020 5.815402
H -0.385697 -5.855860 5.394390
C 0.951398 -7.113706 3.404795
H 1.155768 -7.326068 2.349901
H -0.030003 -7.536981 3.643822
H 1.696449 -7.634826 4.016181
H 8.891073 4.657538 3.181867
H 4.495969 3.140566 -0.050833
H 5.928470 3.879898 -0.791178
C 5.201148 3.066632 -0.885808
H 9.448593 3.305988 4.184212
C 9.687672 3.916088 3.307203
H 4.639583 3.217567 -1.815227
H 10.621526 4.451780 3.514252
N 6.652904 -0.447448 3.544278
C 7.052050 0.509789 2.564492
C 7.492789 -1.503984 3.855814
C 8.156426 1.321773 2.778283
C 6.834873 -2.451756 4.681141
O 5.584190 -2.110394 5.012101
C 6.335888 0.596110 1.368645
C 8.793642 -1.746911 3.436639
C 7.447577 -3.623632 5.059139
C 9.440960 -2.915843 3.856470
C 8.771501 -3.856983 4.665374
C 10.833700 -3.138678 3.422643
C 8.564908 2.239566 1.802917
H 9.361522 4.720620 0.697444
C 6.687778 1.528050 0.399695
C 7.805439 2.342881 0.640619
C 11.487956 -4.412817 3.835670
C 12.790739 -4.675778 3.410831

C 9.429035 -5.089922 5.119210
C 10.817860 -5.344129 4.645175
O 11.443422 -2.313862 2.747979
C 13.420916 -5.855756 3.783195
C 11.459593 -6.525890 5.017619
H 8.710381 1.219124 3.706348
C 12.754710 -6.781578 4.587040
O 8.864109 -5.891219 5.863062
C 5.893758 1.689900 -0.899277
H 5.501477 -0.079402 1.217310
C 9.838825 3.056441 2.038463
H 9.324499 -1.060071 2.786278
H 6.928251 -4.354907 5.666575
H 13.287456 -3.938876 2.787379
H 14.434078 -6.057925 3.447413
H 8.092367 3.063593 -0.115852
H 10.919921 -7.229135 5.644224
H 13.249449 -7.704448 4.876425
H 4.071841 0.650621 -0.262241
C 10.160512 3.990176 0.866117
H 7.587382 2.407093 -2.107730
C 4.817963 0.608585 -1.062175
C 6.843812 1.604414 -2.108885
H 11.078253 4.546509 1.084610
H 4.290240 0.761358 -2.009568
C 11.020895 2.083342 2.219794
H 10.886683 1.434825 3.091169
H 6.270516 1.686127 -3.039243
H 10.323763 3.431567 -0.061761
H 11.953943 2.642233 2.357069
H 5.254319 -0.396469 -1.080859
H 7.377694 0.647977 -2.116173
H 11.130750 1.437722 1.342306
Si 5.019753 -0.631999 4.266217
C 5.006855 0.631805 8.290440
N 5.387685 -0.196366 7.245318
C 4.160774 1.559450 7.764212
N 4.038286 1.269147 6.414669
C 4.784996 0.175396 6.072706
C 6.261834 -1.318426 7.522092
C 7.648489 -1.135899 7.588030
C 8.407362 -2.240326 8.004473
C 7.820949 -3.435955 8.371391
C 6.439994 -3.578306 8.300011
C 5.634896 -2.532691 7.861888
H 8.438518 -4.275297 8.675647
H 5.986603 -4.529329 8.562208
H 9.490365 -2.147150 8.024561
C 8.457558 0.127554 7.289437
C 7.798170 1.263282 6.507350
H 7.044458 1.795322 7.098004
H 8.567723 1.997010 6.242846
H 7.340491 0.914694 5.579741
C 9.065764 0.688450 8.587889
H 9.293509 -0.230409 6.669747
H 8.280836 1.078144 9.244361
H 9.620297 -0.071534 9.144684
H 9.749643 1.512461 8.357433
C 4.132240 -2.725753 7.757741
C 3.473258 -2.663717 9.144052
H 3.850786 -3.466550 9.787495
H 2.387667 -2.783371 9.054709
H 3.671506 -1.711211 9.645913
C 3.761015 -4.030046 7.042644
H 3.720493 -1.910604 7.152311
H 2.678864 -4.060762 6.881506
H 4.029733 -4.911470 7.634812
H 4.246636 -4.097232 6.065543
C 5.467543 0.492825 9.699488
H 6.114264 1.329974 9.984775
H 6.026267 -0.433255 9.846491
H 4.611465 0.492174 10.381695
C 3.127045 2.076014 5.628958
C 1.761400 1.779088 5.717459
C 0.877359 2.643132 5.064881
C 1.338550 3.773674 4.409068
C 2.697975 4.058636 4.376053
C 3.628327 3.210471 4.973545
C 5.113904 3.516532 4.900357
H -0.187212 2.433337 5.082778
H 0.638359 4.432738 3.904843
H 3.042414 4.942898 3.848646
C 5.561756 3.928725 3.493857
H 5.311093 3.161460 2.757426
H 6.649152 4.047449 3.480577
H 5.120281 4.881385 3.181976
C 5.511482 4.589396 5.925649
H 5.658517 2.599479 5.144839
H 5.305108 4.270196 6.952719
H 4.966405 5.522973 5.744479
H 6.583861 4.801832 5.849908
C 1.237446 0.577539 6.487807
C -0.054086 0.886072 7.255200
H 0.033444 1.801275 7.850226
H -0.290679 0.056522 7.929526
H -0.906663 1.005329 6.578985
C 1.035834 -0.626437 5.558542
H 1.993527 0.288339 7.229714
H 0.332894 -0.383394 4.753384
H 0.637668 -1.479166 6.121559
H 1.979139 -0.930263 5.097873
C 3.410877 2.642925 8.451562
H 2.395503 2.317835 8.710343
H 3.315582 3.531331 7.822903
H 3.924310 2.917153 9.376653

1-SIMes

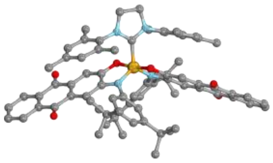

168
xyz, charge: 0, multiplicity:1
C -5.20089 -0.77993 -4.55793
N -5.71732 -2.14189 -4.28792
C -5.33064 -0.09571 -3.18941
N -6.03322 -1.11985 -2.38199
C -6.19700 -2.26203 -3.04984
C -13.82722 -0.92487 4.23639
C -13.02347 -1.51941 5.20985
C -13.43546 -0.94043 2.90422
C -12.23867 -1.55431 2.53338
C -11.43047 -2.15344 3.51268
C -11.82875 -2.12863 4.84980
H -14.76137 -0.44866 4.52054
H -13.33234 -1.50616 6.25125
H -14.04165 -0.48339 2.12828
H -11.18464 -2.59612 5.58803
C -11.83775 -1.55854 1.09868
C -10.58274 -2.23983 0.74479
C -9.78533 -2.86984 1.72205
C -10.14282 -2.80813 3.15046
C -10.19407 -2.27081 -0.60131
C -9.01290 -2.89254 -0.94147
C -8.21470 -3.54872 0.03294
C -8.61112 -3.54151 1.36062
H -10.81852 -1.80426 -1.35572
H -8.03534 -4.04013 2.13241
O -12.53906 -1.00961 0.25111
O -9.40741 -3.26444 4.02343
O -8.52284 -2.96007 -2.18595
N -7.11137 -4.15388 -0.54592
C -5.80208 -7.92734 -4.98477
C -5.48060 -5.75497 -3.29121
C -6.91410 -7.13305 -4.67959
C -5.97633 -9.03369 -5.94264
C -4.39255 -6.56011 -3.54685
C -4.54244 -7.64944 -4.41566
C -2.47160 -10.48447 -5.88662
C -4.80183 -9.91053 -6.20661
C -3.55622 -9.65041 -5.61337
C -3.86190 -11.82709 -7.33066
C -2.62425 -11.56928 -6.73998
C -3.36904 -8.49122 -4.69669
C -6.75842 -6.04080 -3.84134
C -4.94544 -10.99967 -7.06702
N -7.71015 -5.13728 -3.39771
O -5.45270 -4.66988 -2.50697
O -7.04319 -9.22853 -6.52142
O -2.26898 -8.25710 -4.20015
H -1.51913 -10.26108 -5.41615
H -3.97877 -12.67649 -7.99771
H -5.91795 -11.17740 -7.51537
H -1.77796 -12.21790 -6.94791
H -3.42819 -6.35868 -3.09278
H -7.87491 -7.39107 -5.11088
Si -7.03216 -3.90214 -2.30764
C -9.10168 -5.41540 -3.56463
C -9.87972 -5.63461 -2.43061
C -11.24736 -5.87542 -2.54410
C -11.79576 -5.93366 -3.82786
C -11.02901 -5.75509 -4.98184
C -9.66909 -5.47773 -4.83408
H -9.03033 -5.30736 -5.69260
H -9.39995 -5.60473 -1.45867
H -12.85852 -6.13126 -3.93217
C -12.14617 -6.06177 -1.31897
C -11.35217 -6.04446 -0.00730
H -10.60520 -6.84575 0.02835
H -12.03735 -6.19107 0.83441
H -10.84324 -5.08749 0.14694
C -12.88617 -7.40880 -1.41850
H -13.53016 -7.45883 -2.30191
H -13.51955 -7.55449 -0.53597
H -12.17388 -8.23927 -1.47246
C -13.17042 -4.91104 -1.27566
H -13.82658 -5.02183 -0.40433
H -13.79836 -4.89556 -2.17241
H -12.66330 -3.94306 -1.19948
C -11.69426 -5.87716 -6.35532
C -10.71123 -5.61652 -7.50236
H -9.87428 -6.32404 -7.49104
H -10.31063 -4.59727 -7.45887
H -11.22884 -5.72854 -8.46092
C -12.84286 -4.85733 -6.46753
H -13.61594 -5.03026 -5.71299
H -13.31620 -4.92750 -7.45387
H -12.46812 -3.83635 -6.33546
C -12.25302 -7.30390 -6.51657
H -12.73042 -7.41598 -7.49717
H -12.99928 -7.53244 -5.74950
H -11.44968 -8.04412 -6.43661
C -6.35543 -5.12313 0.18279
C -5.63082 -4.75910 1.31408
C -4.89947 -5.71769 2.01716
C -4.89482 -7.02904 1.53564
C -5.61565 -7.41690 0.40327
C -6.36603 -6.44498 -0.25521
H -5.65247 -3.72234 1.62830
H -4.31341 -7.77786 2.06541
H -6.96332 -6.69880 -1.12381
C -4.12047 -5.37939 3.29096
C -2.63531 -5.74098 3.10246
H -2.06701 -5.48855 4.00531
H -2.20665 -5.18954 2.25843
H -2.49789 -6.80893 2.90896
C -4.70377 -6.18943 4.46468
H -5.76175 -5.94747 4.61233
H -4.16360 -5.95904 5.39053
H -4.62614 -7.26649 4.28733
C -4.20746 -3.89093 3.64746
H -3.64814 -3.70201 4.56984
H -5.24242 -3.57106 3.81409
H -3.76906 -3.26719 2.86001
C -5.55587 -8.86748 -0.08252
C -4.11016 -9.18990 -0.50814
H -4.04243 -10.22619 -0.85963
H -3.40783 -9.06771 0.32283
H -3.78950 -8.53212 -1.32329
C -5.98214 -9.81334 1.05576
H -7.00044 -9.58500 1.38869
H -5.31862 -9.73699 1.92267
H -5.95773 -10.85255 0.70836
C -6.47488 -9.11629 -1.28452
H -6.18858 -8.50737 -2.14816
H -7.52435 -8.90953 -1.04544
H -6.40331 -10.16684 -1.58559
H -4.16769 -0.83371 -4.91505
H -4.36111 0.13236 -2.73072
H -5.92051 0.82558 -3.22392
H -5.81006 -0.30290 -5.33491
C -6.34449 -0.87857 -1.00373
C -7.50931 -0.17213 -0.68495
C -5.44111 -1.30813 -0.02207

C -7.79345 0.04335 0.66374
C -5.76189 -1.05484 1.30834
C -6.94502 -0.40675 1.67107
H -8.71733 0.55218 0.92888
H -5.07682 -1.38131 2.08788
C -5.77935 -3.12197 -5.33203
C -4.63571 -3.87184 -5.62746
C -6.96806 -3.25663 -6.06215
C -4.72438 -4.82463 -6.64262
C -7.00109 -4.21004 -7.07554
C -5.90022 -5.02125 -7.36083
H -3.85710 -5.44578 -6.85416
H -7.91406 -4.33399 -7.65405
C -3.34796 -3.67316 -4.87933
H -3.52393 -3.53893 -3.80784
H -2.69287 -4.53732 -5.01193
H -2.80785 -2.79232 -5.25014
C -6.00905 -6.11037 -8.38921
H -5.02494 -6.48337 -8.68517
H -6.57712 -6.95695 -7.98298
H -6.53430 -5.76272 -9.28465
C -8.17906 -2.42421 -5.74441
H -7.93342 -1.36047 -5.65169
H -8.93094 -2.52812 -6.53045
H -8.64324 -2.73415 -4.80047
C -8.43803 0.33807 -1.75015
H -8.05328 1.26263 -2.19985
H -8.57477 -0.39772 -2.54825
H -9.41807 0.56556 -1.32443
C -7.30706 -0.24734 3.11987
H -8.10344 0.48909 3.25638
H -7.66378 -1.20236 3.52633
H -6.44175 0.05985 3.71605
C -4.18343 -2.04681 -0.38681
H -3.52346 -2.12886 0.48018
H -4.40202 -3.06405 -0.73296
H -3.62854 -1.54369 -1.18650

1-^{iPr}NHC

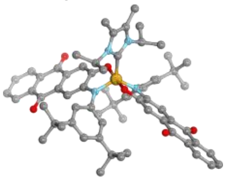

152
xyz, charge: 0, multiplicity:1
C -4.93269 -0.88755 -4.24468
N -5.30546 -2.21299 -4.03325
C -5.60639 -0.13842 -3.32068
N -6.36117 -1.02693 -2.55918
C -6.17816 -2.28874 -3.00509
C -7.35381 -0.69170 -1.50698
C -4.86080 -3.40860 -4.79437
C -1.38560 -8.09120 4.67889
C -1.29035 -9.18721 3.82032
C -2.08668 -6.95846 4.28759
C -2.69836 -6.91167 3.03426
C -2.60319 -8.01443 2.17085
C -1.89611 -9.14868 2.57159
H -0.91065 -8.12373 5.65538
H -0.74186 -10.07265 4.12904
H -2.17621 -6.09290 4.93657
H -1.83683 -9.98771 1.88536
C -3.44736 -5.68718 2.63734
C -4.07461 -5.67284 1.30503
C -4.00823 -6.79130 0.44959
C -3.24267 -7.99582 0.82528
C -4.80454 -4.53816 0.92236
C -5.44712 -4.53603 -0.29699
C -5.37556 -5.65519 -1.16760
C -4.66311 -6.78162 -0.78902
H -4.87756 -3.69042 1.59538
H -4.60970 -7.66316 -1.41912
O -3.54175 -4.73408 3.40745
O -3.13034 -8.95422 0.06652
O -6.20596 -3.53542 -0.75786
N -6.12896 -5.44854 -2.31383
C -11.82345 -4.46184 -3.51214
C -9.07991 -4.22162 -3.87247
C -11.01347 -4.18614 -2.40254
C -13.27985 -4.57810 -3.30211
C -9.87084 -4.49225 -4.96823
C -11.25734 -4.61316 -4.79407
C -14.36287 -5.44752 -6.83341
C -14.12004 -4.93370 -4.47987
C -13.54906 -5.11707 -5.74913
C -16.30013 -5.41054 -5.39612
C -15.73241 -5.59350 -6.65773
C -12.08244 -4.96858 -5.96079
C -9.64502 -4.05920 -2.57999
C -15.49739 -5.08152 -4.31210
N -8.66261 -3.83715 -1.62752
O -7.74600 -4.12200 -3.89974
O -13.79185 -4.39185 -2.20218
O -11.58860 -5.14996 -7.07149
H -13.89655 -5.58487 -7.80401
H -17.37183 -5.52605 -5.26108
H -15.91412 -4.93386 -3.32067
H -16.36260 -5.85073 -7.50440
H -9.43577 -4.63919 -5.95121
H -11.47420 -4.09277 -1.42477
Si -7.01206 -3.89998 -2.29765
C -8.99630 -3.84141 -0.23905
C -8.55970 -4.89416 0.55862
C -8.82255 -4.90052 1.92730
C -9.56033 -3.83740 2.45521
C -10.03946 -2.78668 1.66876
C -9.73944 -2.79757 0.30541
H -10.08557 -2.01016 -0.35542
H -8.00009 -5.69794 0.09270
H -9.77528 -3.83188 3.51966
C -8.32227 -6.01575 2.84875
C -7.59393 -7.12288 2.07720
H -8.24725 -7.60203 1.33924
H -7.26178 -7.89573 2.77838
H -6.70508 -6.74321 1.56261
C -9.51756 -6.64750 3.58706
H -10.04132 -5.92047 4.21546
H -9.16993 -7.45947 4.23590
H -10.23981 -7.06058 2.87458
C -7.34040 -5.41566 3.87355
H -6.96760 -6.20022 4.54239
H -7.81868 -4.64690 4.48923
H -6.47952 -4.96161 3.37186
C -10.87104 -1.67648 2.31638
C -11.27049 -0.58769 1.31374
H -11.87684 -0.98895 0.49438
H -10.38990 -0.09172 0.88843
H -11.86572 0.17754 1.82284
C -10.05843 -1.01374 3.44453
H -9.79411 -1.72747 4.23025
H -10.64011 -0.20745 3.90626
H -9.12776 -0.58783 3.05317
C -12.15854 -2.28935 2.89997
H -12.77349 -1.50967 3.36455
H -11.93492 -3.04211 3.66205
H -12.74788 -2.77177 2.11296
C -6.34674 -6.52251 -3.22921
C -5.29715 -7.00179 -4.00903
C -5.51672 -8.03804 -4.91868
C -6.81199 -8.54981 -5.03166
C -7.87701 -8.08977 -4.25249
C -7.61909 -7.07517 -3.33259
H -4.31321 -6.56221 -3.87899
H -6.99760 -9.34403 -5.74860
H -8.40389 -6.68832 -2.69169
C -4.39567 -8.62793 -5.77843
C -4.75302 -8.47435 -7.26871
H -3.95271 -8.88658 -7.89424
H -4.88466 -7.41809 -7.52883
H -5.67948 -8.99828 -7.52164
C -4.23500 -10.12221 -5.43830
H -3.99013 -10.25291 -4.37887
H -3.42892 -10.56244 -6.03683
H -5.15195 -10.68291 -5.64343
C -3.04943 -7.93653 -5.53560
H -2.28167 -8.40032 -6.16366
H -2.72574 -8.02743 -4.49308
H -3.09124 -6.87316 -5.79811
C -9.27296 -8.69156 -4.43273
C -9.76929 -8.38402 -5.85877
H -10.76915 -8.80754 -6.00967
H -9.10460 -8.80905 -6.61796
H -9.83114 -7.30383 -6.02742
C -9.20759 -10.21664 -4.22612
H -8.83371 -10.45721 -3.22502
H -8.55278 -10.70170 -4.95663
H -10.20755 -10.65180 -4.33482
C -10.28633 -8.11487 -3.43664
H -10.41116 -7.03443 -3.56291
H -9.99572 -8.31449 -2.39899
H -11.26391 -8.57949 -3.60326
C -5.44713 -3.43439 -6.20165
H -5.00383 -2.68135 -6.85936
H -6.52973 -3.29321 -6.16402
H -5.25073 -4.41827 -6.63958
C -3.34514 -3.57645 -4.75316
H -5.29377 -4.25156 -4.25140
H -2.96806 -3.49554 -3.72883
H -2.82218 -2.85440 -5.38485
H -3.09816 -4.57480 -5.12578
C -6.77050 0.16978 -0.39255
H -5.80125 -0.21883 -0.06685
H -6.66553 1.22056 -0.67282
H -7.45266 0.12135 0.46195
C -8.61816 -0.11620 -2.13958
H -7.60954 -1.65129 -1.05410
H -8.42804 0.82393 -2.66551
H -9.35543 0.08497 -1.35665
H -9.05499 -0.82916 -2.84646
C -5.53277 1.33144 -3.09636
H -6.51979 1.78661 -2.98502
H -4.94679 1.57722 -2.20419
H -5.04722 1.80753 -3.95037
C -3.93835 -0.42695 -5.25283
H -2.90934 -0.60577 -4.92171
H -4.07123 -0.91529 -6.22000
H -4.04897 0.64758 -5.41200

1-PBu_3

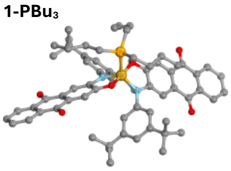

159
xyz, charge: 0, multiplicity:1
P 6.62700 -0.83902 4.08463
C 7.99275 -1.78090 4.89427
H 8.76536 -1.91172 4.12740
H 8.41979 -1.14191 5.68048
C 7.58329 -3.14616 5.45563
H 6.95276 -3.66858 4.72764
H 6.96993 -3.01994 6.35792
C 8.79716 -4.02384 5.76649
H 9.41834 -3.53710 6.53107
H 9.41522 -4.10185 4.86160
C 5.49691 -0.31753 5.47816
H 6.01544 -0.61451 6.40060
H 5.48149 0.77829 5.46076
C 4.06555 -0.85942 5.48275
H 3.55062 -0.54199 4.57046
H 3.53676 -0.38502 6.32110
C 3.96767 -2.37650 5.61863
H 4.34190 -2.68190 6.60667
H 4.61753 -2.86033 4.87799
C 7.48138 0.71139 3.53289
H 7.61043 1.34781 4.41942
H 8.47905 0.40286 3.19901
C 6.76883 1.46555 2.40583
H 6.79255 0.84810 1.49906
H 7.35523 2.36603 2.17672
C 5.32630 1.86590 2.71220
H 4.73342 0.97048 2.95066
H 5.30348 2.50131 3.60939
C 2.54232 -2.87797 5.41317
H 2.19706 -2.63391 4.40324
H 2.48297 -3.96459 5.51846
H 1.85020 -2.42255 6.13101
C 8.39565 -5.42276 6.22692
H 9.27386 -6.02841 6.47050
H 7.75648 -5.38350 7.11679
H 7.84643 -5.94822 5.43650
C 4.66547 2.59412 1.54461
H 4.61478 1.94786 0.66129

H 3.64116 2.89032 1.78976
H 5.22449 3.49735 1.27459
C -2.70639 1.19787 -1.53147
C -2.02215 1.41827 -2.72768
C -2.03050 0.70913 -0.42163
H -3.77007 1.40905 -1.46791
C -0.66272 1.14992 -2.81229
C 0.02427 0.65773 -1.70175
C -0.66413 0.43642 -0.49811
H -2.53975 0.52877 0.51990
H -2.55366 1.80014 -3.59467
H -0.10779 1.31307 -3.73085
C 0.03866 -0.09024 0.70404
C 1.48338 -0.35360 0.58773
C 2.17144 -0.16394 -0.62677
C 1.48275 0.38083 -1.81653
C 2.16002 -0.86339 1.70583
C 3.49715 -1.16751 1.58885
C 4.20392 -0.95336 0.37976
C 3.53468 -0.46444 -0.73205
H 1.61745 -1.02953 2.63041
H 4.03607 -0.31266 -1.68214
O 2.08315 0.60229 -2.86294
O -0.57362 -0.30139 1.74791
O 4.25420 -1.68810 2.57169
N 5.53065 -1.32619 0.50833
C 7.18960 -4.31026 2.20302
C 9.49024 -3.60218 1.79607
C 9.90449 -4.93533 1.92771
C 8.15268 -3.30275 1.92843
C 8.95520 -5.94338 2.18064
C 7.59624 -5.63152 2.30870
C 11.33244 -5.25181 1.74943
C 13.49436 -8.33603 1.84588
C 12.56069 -9.33214 2.13495
C 13.08834 -7.01392 1.72363
C 11.22188 -9.00475 2.30166
C 11.74443 -6.67623 1.88758
C 9.36541 -7.35114 2.36529
C 10.80491 -7.67879 2.17760
O 8.55665 -8.22236 2.67025
O 12.15870 -4.38085 1.49067
O 7.64144 -2.06396 1.81315
N 5.91329 -3.77715 2.31543
H 12.88125 -10.36572 2.22944
H 13.79521 -6.22108 1.49987
H 14.54137 -8.59486 1.71626
H 10.47642 -9.76056 2.52836
H 10.21885 -2.82971 1.57262
H 6.89245 -6.43631 2.49031
Si 5.90321 -2.01505 2.10285
C 6.39848 -1.28629 -0.62410
C 4.77772 -4.59634 2.57661
C 3.66859 -4.50349 1.73432
C 2.52044 -5.24200 1.99748
C 2.52891 -6.10976 3.10058
C 3.63977 -6.24853 3.93030
C 4.76255 -5.45912 3.66382
H 3.72271 -3.84106 0.87747
H 1.63976 -6.69376 3.30668
H 5.63607 -5.51569 4.30570
C 6.70148 -0.07106 -1.22562
C 7.54249 -0.02236 -2.33745
C 8.07827 -1.22252 -2.81352
C 7.78281 -2.45654 -2.22450
C 6.92412 -2.47156 -1.12504
H 6.26405 0.83689 -0.82060
H 6.65160 -3.40659 -0.64239
H 8.73805 -1.19691 -3.67002
C 7.83328 1.33260 -2.98687
C 8.33939 -3.78016 -2.75709
C 9.29381 -3.57921 -3.93996
H 8.79293 -3.10814 -4.79291
H 9.66749 -4.55305 -4.27305
H 10.16013 -2.96882 -3.66212
C 9.10627 -4.50756 -1.63633
H 8.45885 -4.74707 -0.78719
H 9.93220 -3.89270 -1.26291
H 9.52154 -5.44891 -2.01469
C 7.16567 -4.66230 -3.22557
H 6.59311 -4.16075 -4.01329
H 6.48058 -4.89038 -2.40311
H 7.54257 -5.61211 -3.62232
C 8.47186 2.27115 -1.94483
H 7.80496 2.44405 -1.09388
H 8.69451 3.24396 -2.39836
H 9.40600 1.84630 -1.56146
C 6.50851 1.94388 -3.48338
H 5.80210 2.10543 -2.66292
H 6.02499 1.28483 -4.21208
H 6.69433 2.91208 -3.96273
C 8.78967 1.21803 -4.17937
H 9.76080 0.80617 -3.88342
H 8.96515 2.21310 -4.60156
H 8.37254 0.59038 -4.97425
C 1.26125 -5.11636 1.13597
C 0.15373 -4.45655 1.98047
H -0.06755 -5.04128 2.87950
H -0.76848 -4.36775 1.39455
H 0.45224 -3.45007 2.29333
C 1.49234 -4.25535 -0.11271
H 0.56908 -4.21341 -0.70008
H 2.27726 -4.67281 -0.75348
H 1.76168 -3.22546 0.14429
C 0.79688 -6.51093 0.67564
H 0.52699 -7.15512 1.51776
H 1.58442 -7.01324 0.10350
H -0.08727 -6.41658 0.03523
C 3.69918 -7.25101 5.08632
C 4.07580 -6.53629 6.39784
H 5.03932 -6.02237 6.32324
H 4.15236 -7.26614 7.21194
H 3.31714 -5.79633 6.67411
C 4.77665 -8.30393 4.75730
H 4.83167 -9.05330 5.55559
H 5.76838 -7.85199 4.65097
H 4.54397 -8.81649 3.81795
C 2.36370 -7.97163 5.30201
H 1.55616 -7.26734 5.53197
H 2.45456 -8.66282 6.14651
H 2.07388 -8.55961 4.42462

1-PCy$_3$

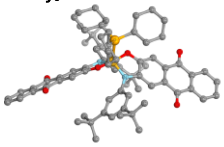

171
xyz, charge: 0, multiplicity:1
P 4.88275 -1.76338 12.51748
Si 6.94168 -0.71266 13.05649
O 7.66620 -2.29742 12.77597
N 8.04458 -0.06073 11.82069
C 12.86079 -2.87946 8.99371
O 12.16332 -0.63823 8.71103
N 7.35443 -0.59349 14.77899
C 12.62084 -4.13012 9.58480
O 11.26822 -5.43317 11.02265
C 13.47896 -5.19790 9.31877
H 13.27209 -6.15421 9.78892
O 6.02055 0.78257 13.21926
C 14.56556 -5.02408 8.47226
H 15.23146 -5.85769 8.26785
O 4.37952 4.90396 15.70178
C 14.80359 -3.78116 7.88395
H 15.65484 -3.64774 7.22243
O 7.20471 2.00449 19.27176
C 13.95451 -2.71374 8.14304
H 14.11779 -1.73721 7.69799
C 11.96360 -1.71966 9.25488
C 10.83559 -1.92405 10.18730
C 10.57991 -3.18918 10.75350
C 11.46359 -4.33938 10.49783
C 10.01817 -0.82721 10.48748
H 10.24816 0.13771 10.04826
C 8.94963 -0.99949 11.35427
C 8.70601 -2.27386 11.92709
C 9.49937 -3.35945 11.63148
H 9.31960 -4.32772 12.08677
C 8.20447 1.32059 11.49609
C 8.53222 2.21676 12.50815
H 8.68995 1.83087 13.50947
C 8.63072 3.58050 12.23958
C 8.43273 4.00171 10.92166
H 8.50950 5.06137 10.69666
C 8.14393 3.11481 9.88102
C 8.01921 1.75939 10.18900
H 7.78024 1.02696 9.42564
C 6.07265 1.30711 14.45274
C 6.83544 0.54383 15.37635
C 8.26717 -1.44100 15.47311
C 9.57629 -1.53809 15.01327
H 9.87261 -0.93034 14.16495
C 10.47478 -2.41326 15.62137
C 10.02358 -3.15347 16.71754
H 10.71378 -3.83751 17.20236
C 8.72129 -3.04989 17.21506
C 7.83896 -2.18253 16.57019
H 6.80966 -2.07608 16.89600
C 4.63441 -1.65939 10.67743
H 3.64737 -2.10084 10.46681
C 5.69337 -2.46074 9.90263
H 5.68237 -3.51508 10.20126
H 6.69446 -2.07347 10.13589
C 5.45289 -2.35240 8.39370
H 4.48797 -2.81834 8.14454
H 6.22643 -2.91856 7.86189
C 5.44707 -0.89401 7.93814
H 5.25820 -0.82953 6.85991
H 6.44390 -0.46305 8.11005
C 4.40319 -0.08734 8.71026
H 3.39868 -0.46054 8.46122
H 4.43075 0.96755 8.41096
C 4.62202 -0.18834 10.22277
H 5.57979 0.28198 10.48379
H 3.84500 0.37780 10.74923
C 8.92511 4.60711 13.33649
C 7.71028 5.54427 13.48109
H 7.49675 6.07610 12.54825
H 6.81455 4.98075 13.76346
H 7.90057 6.29131 14.26055
C 7.96691 3.64964 8.45770
C 9.26856 4.34359 8.01302
H 9.16338 4.72625 6.99112
H 9.51948 5.18666 8.66387
H 10.10783 3.64025 8.03388
C 5.47382 2.49072 14.82225
H 4.90773 3.08017 14.10839
C 5.63373 2.95034 16.13811
C 6.39967 2.20586 17.05679
C 7.00323 1.00058 16.67390
H 7.59851 0.45555 17.39894
C 11.90787 -2.59150 15.11358
C 12.89933 -2.31040 16.25828
H 13.92850 -2.41960 15.89774
H 12.77362 -1.29109 16.63917
H 12.76475 -3.00241 17.09534
C 8.30545 -3.88216 18.43051
C 9.15947 -3.46228 19.64210
H 10.22561 -3.62726 19.45817
H 9.01659 -2.39974 19.86586
H 8.87460 -4.04355 20.52691
C 3.45919 -0.84425 13.27796
H 3.72745 0.19854 13.05140
C 2.06580 -1.10771 12.68793
H 1.74253 -2.13026 12.92068
H 2.07319 -1.01339 11.59599
C 1.06260 -0.10925 13.28157
H 0.06350 -0.30439 12.87331
H 1.34240 0.90607 12.96524
C 1.03774 -0.17404 14.81013
H 0.65464 -1.15672 15.12317
H 0.34392 0.57604 15.20754
C 2.43479 0.03116 15.39880
H 2.41051 -0.06972 16.49026
H 2.78739 1.04995 15.18411
C 3.42851 -0.97372 14.80989
H 4.42619 -0.81890 15.23938
H 3.11083 -1.98945 15.08152
C 4.92304 -3.56710 12.94989
H 5.73436 -3.93483 12.30342
C 5.36632 -3.84947 14.39554
H 4.59483 -3.51414 15.09946
H 6.28552 -3.30537 14.63041
C 5.59286 -5.35282 14.58203
H 5.89071 -5.55081 15.61882
H 6.43310 -5.66944 13.94749

C 4.34217 -6.15688 14.22313
H 3.53747 -5.90820 14.93083
H 4.53518 -7.23096 14.32972
C 3.87190 -5.84442 12.80110
H 2.94944 -6.39190 12.57245
H 4.63045 -6.18487 12.08150
C 3.64128 -4.34037 12.61020
H 3.31406 -4.13714 11.58273
H 2.83201 -4.01580 13.27810
C 10.16977 5.42959 12.95380
H 11.04052 4.77791 12.82360
H 10.02251 5.98588 12.02295
H 10.39684 6.15526 13.74312
C 9.18398 3.94696 14.69648
H 8.31311 3.38308 15.04761
H 10.04722 3.27267 14.66430
H 9.39482 4.72145 15.44144
C 7.65151 2.53570 7.45264
H 7.54380 2.96694 6.45183
H 8.45187 1.78869 7.40796
H 6.71202 2.02655 7.69612
C 6.80716 4.66305 8.43230
H 6.66226 5.04709 7.41579
H 5.87427 4.19107 8.75998
H 6.99914 5.51675 9.08916
C 5.02764 4.24058 16.50797
C 5.23435 4.72548 17.90028
C 5.97785 3.97571 18.82559
C 6.58125 2.66709 18.44856
C 12.23394 -1.64556 13.95116
H 11.58810 -1.82739 13.08547
H 12.13932 -0.59363 14.24331
H 13.26765 -1.80936 13.62889
C 12.08592 -4.04011 14.61914
H 11.39580 -4.26006 13.79774
H 13.10775 -4.19061 14.25170
H 11.90427 -4.76536 15.41894
C 8.53210 -5.37673 18.13500
H 7.95440 -5.69044 17.25841
H 9.58497 -5.59896 17.93791
H 8.21691 -5.98310 18.99206
C 6.82837 -3.68448 18.79142
H 6.16812 -3.98273 17.96831
H 6.57873 -4.30439 19.65889
H 6.60960 -2.64376 19.05411
C 4.67880 5.94637 18.28484
H 4.10891 6.50694 17.55044
C 4.86084 6.41797 19.57795
H 4.42742 7.36926 19.87340
C 5.59931 5.67253 20.49795
H 5.74075 6.04404 21.50890
C 6.15468 4.45634 20.12363
H 6.73281 3.85762 20.82052

1-DABCO

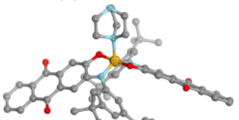

139
xyz, charge: 0, multiplicity: 1
C -9.54397 2.15877 -5.43242
C -9.90913 0.89638 -4.96264
C -8.34869 2.73537 -5.02468
C -7.50802 2.05463 -4.14312
C -7.87499 0.78430 -3.67101
C -9.07852 0.21231 -4.08547
C -6.23048 2.69181 -3.72148
C -5.37277 1.94619 -2.78334
C -5.72244 0.65783 -2.33243
C -7.00229 0.03211 -2.72764
C -4.15680 2.52396 -2.38837
C -3.32251 1.80806 -1.55987
C -3.67075 0.51483 -1.09540
C -4.86861 -0.06094 -1.48579
H -3.87591 3.50347 -2.76080
H -5.15649 -1.05717 -1.16625
H -8.04181 3.71556 -5.37594
H -9.33978 -0.77048 -3.70576
H -10.19586 2.69192 -6.11860
H -10.84469 0.44739 -5.28381
O -5.90686 3.79561 -4.15276
O -7.34181 -1.06590 -2.29899
O -2.11050 2.22285 -1.15496
N -2.65777 -0.01987 -0.31143
C -2.70979 -1.38699 0.10175
C -1.81810 -2.29684 -0.46632
C -1.80241 -3.62111 -0.04721
C -2.73616 -4.01510 0.92421
C -3.66010 -3.13127 1.47611
C -3.62452 -1.79446 1.05809
H -4.31075 -1.05977 1.47103
H -1.12832 -1.93885 -1.22343
H -2.73449 -5.04819 1.25137
C -4.70215 -3.55975 2.51171
C -6.10996 -3.30045 1.94115
H -6.26876 -2.24168 1.71415
H -6.87434 -3.60836 2.66405
H -6.26296 -3.86486 1.01536
C -4.51080 -2.73857 3.80125
H -4.64058 -1.66664 3.62155
H -3.50824 -2.89494 4.21426
H -5.24617 -3.04186 4.55542
C -4.59404 -5.04608 2.87093
H -4.75482 -5.68618 1.99678
H -5.36085 -5.29805 3.61100
H -3.61880 -5.28928 3.30688
C -0.80234 -4.63937 -0.59966
C 0.09860 -4.03885 -1.68619
H -0.48122 -3.68420 -2.54578
H 0.79176 -4.80595 -2.04704
H 0.70014 -3.20732 -1.30254
C -1.56537 -5.83101 -1.20876
H -2.22696 -5.49689 -2.01530
H -2.17665 -6.34897 -0.46332
H -0.85676 -6.55765 -1.62227
C 0.09659 -5.13487 0.54970
H 0.66206 -4.30671 0.98991
H 0.81591 -5.87179 0.17400
H -0.48750 -5.60918 1.34484
H 2.20445 5.03977 -2.69460
H 2.78946 6.07876 -4.00183
C 2.03535 5.32872 -3.73658
H 1.04628 5.79489 -3.80298
H 3.71900 3.13523 -3.52854
H 4.30462 4.18894 -4.82593
C 3.52338 3.47043 -4.55183
C 2.13216 4.11953 -4.68632
O -0.32295 -0.05382 0.80416
H 1.80763 2.85025 -2.28364
C 1.07231 2.53936 -3.02104
C 1.06540 3.08870 -4.31014
N 0.13822 1.02368 -1.34926
H -3.32078 2.89168 -5.30503
H 2.76256 5.35630 -6.34054
C 0.83990 -0.46419 0.27004
C 1.97475 4.62707 -6.12426
C 1.13794 0.13756 -0.97617
H 1.01066 5.12466 -6.27664
C 1.66702 -1.41023 0.83118
H 1.42274 -1.88568 1.77530
C 0.13870 1.58055 -2.66723
C 0.07913 2.66540 -5.19739
H 3.61006 2.59874 -5.20894
C 2.28052 -0.23368 -1.66702
C 2.82616 -1.78768 0.13700
H 0.04356 3.09641 -6.19100
C -2.81641 2.51656 -6.20188
O 3.38663 -3.39631 1.77362
C 3.12649 -1.19978 -1.10799
C 3.67153 -2.84926 0.71110
C -0.81498 1.13996 -3.58448
H -2.22151 3.33291 -6.62379
C -0.87549 1.69416 -4.85664
H 2.52898 0.19168 -2.63382
H -3.40659 0.50081 -4.44194
C 4.34669 -1.57544 -1.85415
C 4.88623 -3.25028 -0.04996
H -3.58611 2.24656 -6.93425
H 2.06703 3.81424 -6.85265
C 5.20721 -2.64384 -1.27495
C 5.71388 -4.24784 0.46699
O 4.64709 -1.03281 -2.91221
C -1.94575 1.28835 -5.87249
H 5.44329 -4.70155 1.41528
H -1.51744 0.37473 -3.27114
C -2.86358 0.18152 -5.33849
C 6.35247 -3.04196 -1.96584
C 6.85091 -4.63841 -0.22726
C 7.17046 -4.03510 -1.44447
H -3.60826 -0.07151 -6.10043
H 7.49283 -5.41519 0.17845
H 6.57900 -2.55743 -2.91043
H -2.30550 -0.73178 -5.10327
H 8.06050 -4.34300 -1.98579
C -1.26798 0.77695 -7.15783
H -0.65124 1.54860 -7.62877
H -2.02771 0.46750 -7.88463
H -0.62587 -0.08392 -6.94225
Si -1.23665 1.02936 -0.23117
N -1.22934 2.28417 1.32897
C -1.97080 3.58412 1.12317
C -1.86417 1.61024 2.52807
C 0.20317 2.62788 1.67450
C 0.22084 3.69430 2.79743
C -1.54538 2.43974 3.79707
H -1.48463 0.58996 2.59586
H -2.93901 1.56536 2.32217
H 0.68981 2.97792 0.75721
H 0.68909 1.70115 1.98503
H 0.48049 4.68255 2.40122
H 0.97407 3.43098 3.54855
H -0.75623 1.95905 4.38649
H -2.43572 2.50961 4.43238
C -2.03940 4.35089 2.46875
H -2.96491 3.35315 0.73656
H -1.43817 4.14263 0.34930
H -3.04646 4.29498 2.89797
H -1.80943 5.41035 2.30857
N -1.09437 3.79381 3.44512

[1-F][NBu$_4$]

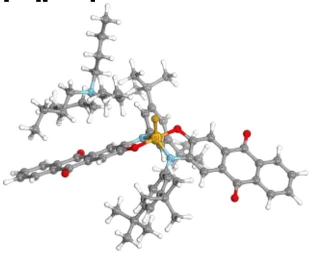

173
xyz, charge: 0, multiplicity: 1
C -0.821055 3.008050 0.806418
C -1.096529 2.043724 1.775839
C 0.472596 3.157782 0.320377
C 1.501460 2.343346 0.795862
C 1.224021 1.374416 1.775041
C -0.077262 1.231273 2.258568
H -1.617610 3.643575 0.429733
H -2.107775 1.927032 2.155307
H 0.701234 3.903466 -0.434853
H -0.275841 0.477167 3.013994
C 2.878081 2.511403 0.253608
C 3.918845 1.592673 0.729330
C 3.649197 0.636406 1.733803
C 2.297556 0.483824 2.294396
C 5.207955 1.687757 0.177575
C 6.192598 0.819773 0.606024
C 5.915570 -0.162476 1.599690
C 4.654423 -0.236979 2.172012
H 4.425036 -0.973022 2.935603
H 5.421461 2.432349 -0.582860
O 3.116928 3.390547 -0.577865
O 2.044539 -0.359602 3.156091
O 7.439887 0.795180 0.154647
N 7.020104 -0.938724 1.848912
C 6.938834 -1.968930 2.837475
C 6.434633 -3.218373 2.476352
C 6.346490 -4.236981 3.421451
C 6.782172 -3.967183 4.728934
C 7.285276 -2.722607 5.107548
C 7.349667 -1.713947 4.135844
H 7.734104 -0.726032 4.376343
H 6.725125 -4.760614 5.465329

H	6.133035	-3.372490	1.445780
C	5.804618	-5.627243	3.077152
C	7.765873	-2.422571	6.530285
C	9.245638	-1.994876	6.486963
H	9.868588	-2.787700	6.056927
H	9.607369	-1.788871	7.501487
H	9.386967	-1.087959	5.890140
C	6.924263	-1.273293	7.117161
H	7.026573	-0.355943	6.528172
H	7.251286	-1.054048	8.140587
H	5.861935	-1.542307	7.147369
C	7.642307	-3.635713	7.458509
H	8.243663	-4.481242	7.105653
H	6.603041	-3.968454	7.560299
H	8.003897	-3.364654	8.456491
C	6.901257	-6.678782	3.329897
H	7.783697	-6.478550	2.711166
H	6.527812	-7.679322	3.080629
H	7.217373	-6.690123	4.378066
C	5.365040	-5.730897	1.611801
H	6.200174	-5.555299	0.924204
H	4.565840	-5.017244	1.380050
H	4.979599	-6.737950	1.417782
C	4.587881	-5.935926	3.969393
H	4.854474	-5.936316	5.031234
H	4.184004	-6.925415	3.724003
H	3.794310	-5.194560	3.818322
H	11.439229	3.511378	-1.978930
H	6.966073	6.666470	1.722418
H	5.661167	4.463514	1.718305
H	9.324560	6.508399	-1.070811
H	11.096688	6.877295	0.739621
H	7.752359	3.669020	4.760467
C	13.539544	0.934225	4.560215
C	11.368065	-1.871488	3.214366
C	10.494322	-1.144987	2.431223
C	9.473190	2.210941	1.427307
C	10.603864	0.270965	2.334928
C	11.597975	0.939546	3.033967
C	12.486588	0.206371	3.833578
C	14.447778	0.148398	5.440167
C	15.440076	0.809974	6.165109
C	14.319037	-1.246462	5.549163
C	15.184169	-1.956956	6.382499
C	10.261388	2.916928	0.518463
C	12.376734	-1.198999	3.923738
C	13.270142	-1.980179	4.788265
C	16.297055	0.094641	6.992977
C	16.168918	-1.290049	7.101666
C	8.382466	4.252034	2.129320
C	7.733328	4.731843	4.497061
C	8.555011	2.865632	2.233680
C	10.958151	5.127219	-0.579171
C	5.958891	4.329929	2.764890
C	7.260736	6.452335	2.756125
C	11.756271	6.188941	0.201271
C	11.949148	4.267696	-1.371583
C	10.019024	5.830384	-1.577587
C	7.347873	4.945325	3.020978
C	9.173831	4.942735	1.212061
C	10.123319	4.298601	0.402047
N	9.613768	0.791252	1.532675
O	13.165558	-3.204130	4.895767
O	9.484780	-1.658606	1.735548
O	13.674366	2.154247	4.457462
H	7.006575	5.227658	5.151788
H	16.839180	-1.849097	7.748602
H	8.211095	6.959440	2.957786
H	9.429297	5.097307	-2.140270
H	12.424499	5.716433	0.930532
H	5.941996	3.258318	2.989415
H	15.072813	-3.034302	6.456543
H	17.066904	0.615575	7.555278
H	9.056738	6.016037	1.115640
H	11.270832	-2.949255	3.296836
H	7.963140	2.278807	2.931679
H	10.967862	2.362394	-0.090417
H	15.527448	1.887910	6.070766
H	11.695926	2.019012	2.980783
H	12.520801	4.909280	-2.051148
H	12.663197	3.759408	-0.713654
H	6.503837	6.891666	3.415334
H	12.367875	6.780966	-0.490068
H	5.209219	4.818633	3.398799
H	10.603868	6.421260	-2.292839
H	8.724339	5.151584	4.705498
Si	8.470394	-0.438543	0.933588
F	8.746192	-1.137840	-0.520998
N	4.268805	-2.208347	-2.120296
C	5.641696	-1.576063	-2.388113
C	4.328420	-3.723332	-2.267025
C	3.207753	-1.643731	-3.042634
C	3.978864	-1.904621	-0.643383
C	2.784833	-2.569333	0.033376
H	3.907769	-0.817193	-0.565556
H	4.895357	-2.213824	-0.131781
C	2.915119	-0.160801	-2.861118
H	2.306446	-2.240532	-2.881906
H	3.549949	-1.837240	-4.062369
C	5.989705	-1.230278	-3.829763
H	6.369609	-2.276519	-1.964410
H	5.676213	-0.661579	-1.778172
C	4.889958	-4.286644	-3.561681
H	3.302856	-4.076061	-2.129206
H	4.929965	-4.076435	-1.421533
C	4.748409	-5.812862	-3.545834
H	5.950775	-4.033986	-3.666695
H	4.360058	-3.887685	-4.434734
C	5.334791	-6.453107	-4.800636
H	3.687042	-6.079668	-3.454020
H	5.252108	-6.214503	-2.655749
H	4.828970	-6.088833	-5.702481
H	5.228268	-7.542727	-4.774286
H	6.402080	-6.223206	-4.898752
C	7.455444	-0.787616	-3.900887
H	5.357717	-0.410820	-4.190854
H	5.839317	-2.076013	-4.505890
C	7.854191	-0.367430	-5.312418
H	8.099660	-1.609506	-3.561243
H	7.619098	0.046808	-3.205778
H	7.724026	-1.193150	-6.021931
H	8.903715	-0.056870	-5.348826
H	7.244060	0.473978	-5.661264
C	2.065173	0.348036	-4.028269
H	3.843434	0.420879	-2.805449
H	2.373071	0.014848	-1.925715
C	1.670581	1.811060	-3.852635
H	1.161902	-0.271629	-4.115255
H	2.623942	0.221770	-4.965548
H	1.074681	1.950778	-2.942886
H	1.074858	2.165257	-4.700705
H	2.557006	2.451032	-3.773357
C	1.399958	-1.961630	-0.200353
H	3.011628	-2.461823	1.104327
H	2.766113	-3.649587	-0.147828
C	0.367215	-2.545918	0.759927
H	1.062850	-2.124326	-1.230636
H	1.457819	-0.874999	-0.055821
H	0.264923	-3.628937	0.620302
H	-0.617970	-2.093529	0.602468
H	0.657313	-2.366934	1.801892

[1-Cl][PPh$_4$]

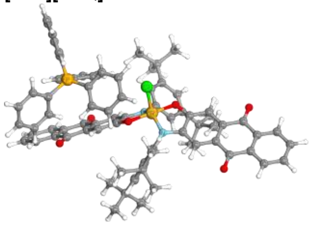

165

xyz, charge: 0, multiplicity: 1

C	-1.382260	2.757230	1.352451
C	-1.651895	1.743674	2.272074
C	-0.087014	2.947450	0.885749
C	0.948263	2.123690	1.330306
C	0.676228	1.100758	2.254416
C	-0.626483	0.919544	2.720832
H	-2.184461	3.399893	1.000598
H	-2.663929	1.597233	2.638886
H	0.138509	3.733387	0.171481
H	-0.821953	0.127158	3.436650
C	2.328120	2.345207	0.821534
C	3.373766	1.408897	1.242755
C	3.114830	0.407610	2.205125
C	1.758108	0.199039	2.736599
C	4.661718	1.542313	0.699655
C	5.655325	0.673150	1.101266
C	5.403527	-0.326800	2.080065
C	4.136111	-0.450902	2.631119
H	3.915414	-1.208989	3.374837
H	4.871237	2.320504	-0.026983
O	2.567067	3.290335	0.063331
O	1.507064	-0.699284	3.540884
O	6.905350	0.689129	0.651666
N	6.540280	-1.052917	2.350240
C	6.484355	-2.108907	3.310584
C	5.740640	-3.254325	3.021494
C	5.663106	-4.290637	3.947322
C	6.367822	-4.155430	5.154818
C	7.118446	-3.019726	5.457955
C	7.156466	-1.982639	4.516216
H	7.723491	-1.074756	4.704015
H	6.321878	-4.966673	5.872364
H	5.232043	-3.308923	2.064770
C	4.845597	-5.559147	3.687624
C	7.889752	-2.863157	6.772346
C	9.384051	-2.651142	6.461912
H	9.793434	-3.504572	5.908997
H	9.951187	-2.543997	7.394429
H	9.548708	-1.749006	5.864080
C	7.351786	-1.634986	7.530696
H	7.479283	-0.714872	6.951142
H	7.890042	-1.512248	8.478210
H	6.285032	-1.750776	7.755401
C	7.755676	-4.089583	7.681433
H	8.143971	-4.996018	7.203241
H	6.714885	-4.270801	7.973236
H	8.333113	-3.923125	8.597419
C	5.780944	-6.782458	3.702726
H	6.550938	-6.694540	2.927445
H	5.207115	-7.697437	3.512309
H	6.283253	-6.896241	4.668985
C	4.124123	-5.518224	2.335409
H	4.828453	-5.444496	1.498939
H	3.425072	-4.675769	2.274566
H	3.547282	-6.440319	2.204153
C	3.784921	-5.715738	4.793754
H	4.241736	-5.811186	5.784117
H	3.184964	-6.615616	4.612532
H	3.109624	-4.852214	4.811300
H	10.847960	3.235071	-1.964075
H	6.769780	6.624183	1.834681
H	5.390671	4.460047	1.959861
H	9.172636	6.454240	-0.869755
H	11.054669	6.519924	0.863677
H	7.549621	3.720717	4.963032
C	13.668000	0.670939	3.919278
C	11.134988	-2.049662	3.159027
C	10.148428	-1.295047	2.558586
C	9.092795	2.070494	1.627245
C	10.296786	0.109045	2.392521
C	11.444030	0.742232	2.848202
C	12.449521	-0.017739	3.462211
C	14.721460	-0.146153	4.581775
C	15.886746	0.475281	5.033527
C	14.558969	-1.530957	4.755568
C	15.564450	-2.271805	5.378335
C	9.868862	2.711244	0.659854
C	12.300068	-1.413733	3.616213
C	13.325922	-2.221846	4.287664
C	16.883170	-0.270207	5.652094
C	16.722083	-1.645100	5.824054
C	8.110190	4.178074	2.292658
C	7.557558	4.773041	4.660509
C	8.231681	2.790719	2.440884
C	10.642590	4.858609	-0.509445
C	5.714766	4.368984	3.003200
C	7.089629	6.440636	2.866866
C	11.601604	5.793776	0.252810
C	11.478897	3.922562	-1.388509
C	9.739218	5.702191	-1.427977
C	7.132504	4.943220	3.189792
C	8.899673	4.804882	1.329144
C	9.787830	4.094007	0.505625
N	9.184895	0.653812	1.790537
O	13.184264	-3.433097	4.467923

O 8.989008 -1.774226 2.114735
O 13.825253 1.882587 3.764282
H 6.868154 5.317366 5.317062
H 17.501441 -2.227808 6.307078
H 8.062658 6.921397 3.018679
H 9.022943 5.067758 -1.962843
H 12.260517 5.220594 0.915516
H 5.665237 3.311736 3.283328
H 15.424594 -3.340807 5.506189
H 17.787818 0.219552 6.001423
H 8.833087 5.879933 1.207975
H 11.010917 -3.119602 3.290284
H 7.645999 2.253563 3.182460
H 10.524111 2.107596 0.040127
H 15.997709 1.546042 4.893136
H 11.578066 1.813604 2.741758
H 12.060396 4.517404 -2.101403
H 12.185647 3.328457 -0.798434
H 6.369838 6.932289 3.530686
H 12.228191 6.350177 -0.454865
H 5.002028 4.916579 3.631535
H 10.349922 6.228467 -2.171146
H 8.567859 5.166299 4.823116
Si 7.946960 -0.558396 1.363456
Cl 8.152126 -1.535456 -0.557932
C 4.517991 -0.970180 -7.885887
C 4.736011 -0.245020 -6.721225
C 3.592349 -2.012029 -7.898881
C 2.882172 -2.332476 -6.745606
C 3.089712 -1.612340 -5.573433
C 4.015922 -0.566384 -5.562850
H 5.076056 -0.722625 -8.783988
H 5.465294 0.560911 -6.713502
H 3.428836 -2.579024 -8.810994
H 2.166139 -3.148871 -6.752775
H 2.539092 -1.873129 -4.674674
C 6.049480 0.660863 -3.855304
C 6.537668 1.842909 -3.291283
C 6.929644 -0.362825 -4.228499
C 7.906848 1.995684 -3.101195
C 8.295462 -0.194728 -4.037954
C 8.783974 0.982030 -3.475819
H 5.862124 2.644084 -3.007378
H 6.553098 -1.284201 -4.663577
H 8.287560 2.914268 -2.665204
H 8.978234 -0.988936 -4.324566
H 9.852694 1.107141 -3.325807
C 1.695759 -1.080462 -1.285030
C 2.534986 -1.833257 -0.465062
C 2.218677 -0.385789 -2.368970
C 3.900331 -1.895546 -0.728117
C 3.593748 -0.451258 -2.630001
C 4.437102 -1.204460 -1.809183
H 0.631173 -1.027045 -1.076432
H 2.123032 -2.368039 0.386487
H 1.562914 0.205509 -3.002129
H 4.557237 -2.471323 -0.082725
H 5.506527 -1.242816 -1.994394
C 2.185181 4.484833 -4.422846
C 2.546931 3.989965 -3.171862
C 2.434693 3.737379 -5.570819
C 3.163228 2.749149 -3.064395
C 3.052727 2.494908 -5.476759
C 3.421682 2.003417 -4.221024
H 1.699692 5.453525 -4.502609
H 2.346064 4.560545 -2.270656
H 2.143705 4.117540 -6.545532
H 3.428551 2.368954 -2.082039
H 3.233995 1.915357 -6.376488
P 4.269689 0.413688 -4.064306

[1-N_3][NBu_4]

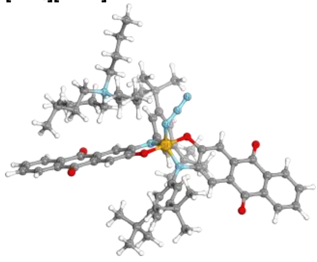

175
xyz, charge: 0, multiplicity: 1
C -0.913239 2.926806 0.700892
C -1.184990 1.969241 1.678030
C 0.384431 3.094982 0.231822
C 1.421047 2.305590 0.731926
C 1.147236 1.343366 1.718810
C -0.158056 1.181776 2.185380
H -1.715825 3.542853 0.305144
H -2.199312 1.838416 2.044442
H 0.610665 3.835966 -0.528752
H -0.353682 0.432674 2.946530
C 2.802156 2.494174 0.208814
C 3.852781 1.601816 0.711703
C 3.585146 0.646920 1.717537
C 2.228572 0.477150 2.262080
C 5.149546 1.719548 0.182707
C 6.141896 0.873726 0.634127
C 5.868750 -0.107932 1.629027
C 4.599104 -0.203923 2.179110
H 4.371428 -0.938016 2.945102
H 5.361966 2.462028 -0.579977
O 3.037164 3.367861 -0.629537
O 1.979051 -0.360793 3.129903
O 7.397678 0.872320 0.201542
N 6.985284 -0.857304 1.904002
C 6.914127 -1.899093 2.880138
C 6.387407 -3.138755 2.515623
C 6.325122 -4.172971 3.445492
C 6.804528 -3.927667 4.742635
C 7.328427 -2.693096 5.125157
C 7.370564 -1.669362 4.167994
H 7.772205 -0.689039 4.411226
H 6.766003 -4.733131 5.467079
H 6.047807 -3.273276 1.494091
C 5.766322 -5.555119 3.095667
C 7.854518 -2.419198 6.537171
C 9.335804 -2.002436 6.456624
H 9.940371 -2.794554 5.999829
H 9.727275 -1.811697 7.463059
H 9.467753 -1.089224 5.867329
C 7.040353 -1.272545 7.166325
H 7.132200 -0.347323 6.588115
H 7.399981 -1.071148 8.182448
H 5.977315 -1.533937 7.224923
C 7.748582 -3.645460 7.450150
H 8.332025 -4.490206 7.066750
H 6.710054 -3.971524 7.577958
H 8.142061 -3.392419 8.440780
C 6.859780 -6.617677 3.313033
H 7.730835 -6.417008 2.678459
H 6.473136 -7.612104 3.059628
H 7.197361 -6.644577 4.354155
C 5.295981 -5.637287 1.638631
H 6.117085 -5.452625 0.936625
H 4.493094 -4.919391 1.434933
H 4.904762 -6.640823 1.438155
C 4.566078 -5.865184 4.009651
H 4.854920 -5.879438 5.065637
H 4.149503 -6.848781 3.761781
H 3.775354 -5.116175 3.883679
H 11.504733 3.582271 -1.878929
H 6.923589 6.729123 1.692827
H 5.625779 4.516109 1.677776
H 9.368010 6.579615 -1.029602
H 11.088694 6.948369 0.828524
H 7.661339 3.754667 4.765385
C 13.596505 0.952572 4.483394
C 11.378634 -1.828421 3.161493
C 10.483238 -1.087268 2.419777
C 9.446265 2.282337 1.472460
C 10.590233 0.328868 2.340836
C 11.606314 0.984990 3.020689
C 12.518342 0.237666 3.779214
C 14.533464 0.150870 5.317334
C 15.552710 0.798597 6.017036
C 14.407163 -1.245728 5.405824
C 15.302113 -1.971885 6.192906
C 10.258942 2.987872 0.584605
C 12.410224 -1.168601 3.849709
C 13.328228 -1.964675 4.673926
C 16.439195 0.067742 6.798912
C 16.313884 -1.318755 6.886588
C 8.339525 4.324236 2.146126
C 7.639778 4.816007 4.496201
C 8.508433 2.937811 2.255231
C 10.986948 5.197861 -0.492767
C 5.902229 4.395156 2.731746
C 7.195076 6.523223 2.734547
C 11.763087 6.260172 0.308579
C 11.998843 4.337575 -1.257444
C 10.075015 5.900208 -1.516788
C 7.283204 5.018383 3.011271
C 9.155398 5.014379 1.250283
C 10.125859 4.369672 0.465837
N 9.577787 0.862460 1.576478
O 13.219418 -3.188951 4.771065
O 9.450118 -1.590657 1.745677
O 13.728716 2.173746 4.396420
H 6.896295 5.309999 5.133317
H 17.007727 -1.889910 7.497034
H 8.138053 7.036253 2.954854
H 9.499757 5.166578 -2.093532
H 12.411410 5.788424 1.056087
H 5.883554 3.326082 2.967728
H 15.192238 -3.050386 6.251159
H 17.229894 0.577937 7.341739
H 9.041458 6.087727 1.150673
H 11.282816 -2.907416 3.227479
H 7.898617 2.351869 2.938203
H 10.979594 2.432831 -0.006985
H 15.637984 1.878019 5.939302
H 11.704300 2.065021 2.981925
H 12.590345 4.978440 -1.920449
H 12.693284 3.827785 -0.579917
H 6.421224 6.963179 3.373321
H 12.393023 6.852190 -0.366038
H 5.137778 4.888049 3.344345
H 10.679062 6.489190 -2.217474
H 8.623209 5.244349 4.722203
Si 8.426288 -0.358360 0.970780
N 8.664514 -1.082135 -0.661674
N 4.319914 -2.191410 -2.193309
C 5.690789 -1.559594 -2.472985
C 4.388124 -3.708341 -2.319705
C 3.254668 -1.644526 -3.121564
C 4.029511 -1.871239 -0.719840
C 2.837859 -2.536542 -0.038645
H 3.953184 -0.783159 -0.653002
H 4.948515 -2.170685 -0.206171
C 2.950583 -0.161887 -2.955976
H 2.357691 -2.246076 -2.954970
H 3.597932 -1.846313 -4.139308
C 6.029385 -1.230069 -3.920869
H 6.421214 -2.255724 -2.046988
H 5.730598 -0.640526 -1.880784
C 4.960533 -4.285214 -3.603553
H 3.363900 -4.065204 -2.183019
H 4.986960 -4.046462 -1.465954
C 4.834433 -5.812366 -3.565299
H 6.019246 -4.023245 -3.708518
H 4.429557 -3.904774 -4.484203
C 5.431398 -6.464983 -4.808689
H 3.775535 -6.088583 -3.472798
H 5.339476 -6.195440 -2.667862
H 4.924831 -6.119193 -5.717375
H 5.335930 -7.555134 -4.766619
H 6.496600 -6.225572 -4.906751
C 7.499558 -0.807022 -4.015091
H 5.403148 -0.406331 -4.282426
H 5.860661 -2.079933 -4.587371
C 7.885958 -0.411617 -5.437080
H 8.137223 -1.633452 -3.672131
H 7.683059 0.032661 -3.331924
H 7.735055 -1.244976 -6.133410
H 8.939102 -0.116189 -5.491137
H 7.282913 0.433405 -5.789572
C 2.097083 0.327234 -4.128973
H 3.874281 0.427633 -2.907335
H 2.406801 0.020008 -2.022716
C 1.682215 1.786063 -3.965424
H 1.202262 -0.304884 -4.213998
H 2.660102 0.201319 -5.063738
H 1.074694 1.923150 -3.062965
H 1.090836 2.127751 -4.821632
H 2.559397 2.438016 -3.880591

C 1.448553 -1.943061 -0.282783
H 3.059801 -2.416322 1.031687
H 2.827536 -3.618644 -0.208479
C 0.417955 -2.527306 0.679950
H 1.115451 -2.119613 -1.312031
H 1.495651 -0.854447 -0.149618
H 0.331239 -3.613511 0.555181
H -0.572317 -2.090955 0.509576
H 0.698811 -2.329088 1.720874
N 9.285350 -2.090986 -0.904632
N 9.835591 -3.031355 -1.236775

[1-NCS][NBu$_4$]

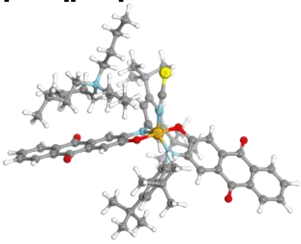

175
xyz, charge: 0, multiplicity: 1
C -0.820224 2.920989 0.842639
C -1.123138 1.947150 1.794327
C 0.484390 3.066944 0.385849
C 1.497417 2.240586 0.874743
C 1.192473 1.262956 1.837003
C -0.120245 1.122002 2.289342
H -1.604208 3.566299 0.456406
H -2.143105 1.833214 2.150519
H 0.733923 3.818981 -0.356309
H -0.340481 0.359900 3.030562
C 2.884642 2.400229 0.358477
C 3.915649 1.488370 0.868930
C 3.619216 0.526401 1.860306
C 2.247624 0.357995 2.368560
C 5.218009 1.582153 0.348473
C 6.190735 0.717726 0.807966
C 5.898697 -0.243951 1.815316
C 4.616365 -0.336008 2.336382
H 4.369118 -1.071485 3.095118
H 5.448703 2.317422 -0.416058
O 3.141789 3.267287 -0.479999
O 1.965956 -0.509562 3.195925
O 7.447118 0.682281 0.375805
N 7.011915 -0.983573 2.130575
C 6.899260 -2.085286 3.034017
C 6.375893 -3.288627 2.558933
C 6.282600 -4.391956 3.402456
C 6.726652 -4.250470 4.727619
C 7.247735 -3.053471 5.218747
C 7.322404 -1.958468 4.346340
H 7.732934 -1.006376 4.671479
H 6.664362 -5.109204 5.386347
H 6.066839 -3.337944 1.519715
C 5.730845 -5.739322 2.928548
C 7.746597 -2.896550 6.658201
C 9.240838 -2.520699 6.637766
H 9.832901 -3.299188 6.142777
H 9.614009 -2.409295 7.662972
H 9.415342 -1.575243 6.113549
C 6.950902 -1.774459 7.351571
H 7.085041 -0.811707 6.847877
H 7.289157 -1.659950 8.388329
H 5.879474 -2.006146 7.364991
C 7.583891 -4.180770 7.478480
H 8.157573 -5.012130 7.053334
H 6.534170 -4.487082 7.553074
H 7.953412 -4.008678 8.495366
C 6.814724 -6.820618 3.093960
H 7.703297 -6.577055 2.499908
H 6.432238 -7.791275 2.756280
H 7.123812 -6.926961 4.138903
C 5.302548 -5.703301 1.456684
H 6.141407 -5.460644 0.794171
H 4.502280 -4.972440 1.291983
H 4.919709 -6.687501 1.165140
C 4.502930 -6.114155 3.779512
H 4.760746 -6.217926 4.838517
H 4.087424 -7.070247 3.439276
H 3.720853 -5.350621 3.693100
H 11.115054 2.965974 -2.304528
H 7.094758 6.718383 1.350064
H 5.755261 4.547493 1.675787
H 9.082985 6.073030 -1.629909
H 11.029825 6.695816 -0.089511
H 8.017858 4.058821 4.653614
C 13.635206 1.219001 4.573059
C 11.432493 -1.702526 3.564697
C 10.534024 -1.051184 2.745498
C 9.492013 2.183532 1.415262
C 10.637424 0.346744 2.510432
C 11.650578 1.079593 3.109340
C 12.563232 0.425304 3.949215
C 14.579638 0.517332 5.485008
C 15.598551 1.242467 6.104580
C 14.458822 -0.861269 5.728586
C 15.358698 -1.491910 6.589036
C 10.194217 2.745778 0.349680
C 12.458307 -0.964420 4.177505
C 13.382288 -1.661237 5.082193
C 16.490391 0.606276 6.959660
C 16.370395 -0.762222 7.201797
C 8.482306 4.317077 1.934514
C 8.004978 5.084544 4.271121
C 8.655819 2.953633 2.207649
C 10.772488 4.770853 -1.112941
C 6.113105 4.541138 2.712032
C 7.462573 6.609276 2.376710
C 11.637152 5.929947 -0.582831
C 11.682315 3.796027 -1.868353
C 9.728863 5.325218 -2.101534
C 7.529974 5.140175 2.807085
C 9.184598 4.863397 0.860678
C 10.047240 4.100037 0.057108
N 9.625508 0.784379 1.686070
O 13.279644 -2.868019 5.311685
O 9.502274 -1.626523 2.130359
O 13.756827 2.425393 4.358375
H 7.331902 5.671898 4.907027
H 17.067586 -1.259389 7.870407
H 8.439825 7.100290 2.448098
H 9.092374 4.519950 -2.486961
H 12.378444 5.566392 0.138267
H 6.086251 3.508669 3.075807
H 15.253258 -2.557393 6.768366
H 17.281588 1.176241 7.438554
H 9.062381 5.915504 0.630006
H 11.343993 -2.768354 3.748248
H 8.123495 2.472399 3.024234
H 10.834201 2.099862 -0.242296
H 15.679130 2.306915 5.906894
H 11.745674 2.148469 2.947928
H 12.180982 4.325568 -2.687602
H 12.460267 3.378889 -1.218715
H 6.771592 7.146717 3.035529
H 12.172504 6.408357 -1.411619
H 5.414349 5.129235 3.319102
H 10.231100 5.801856 -2.951960
H 9.015979 5.496547 4.370689
Si 8.473743 -0.492185 1.231738
N 8.761504 -1.397718 -0.278754
N 4.302367 -2.012845 -2.137966
C 5.604319 -1.221057 -2.305197
C 4.538671 -3.502535 -2.358701
C 3.220258 -1.516470 -3.073394
C 3.918833 -1.825743 -0.664475
C 2.726844 -2.596664 -0.102905
H 3.771162 -0.752904 -0.523784
H 4.825716 -2.107707 -0.120716
C 2.789159 -0.073787 -2.848887
H 2.372074 -2.195694 -2.949774
H 3.602894 -1.645569 -4.089533
C 5.990352 -0.760283 -3.704137
H 6.383366 -1.855847 -1.871081
H 5.503374 -0.340068 -1.665334
C 5.265174 -3.920036 -3.625386
H 3.552417 -3.973102 -2.327552
H 5.110403 -3.841149 -1.486887
C 5.339690 -5.449797 -3.677675
H 6.286107 -3.523886 -3.628582
H 4.753665 -3.552367 -4.522829
C 6.127425 -5.938668 -4.889538
H 4.323857 -5.866605 -3.699910
H 5.812999 -5.819176 -2.757063
H 5.664877 -5.600136 -5.824075
H 6.173845 -7.032522 -4.915700
H 7.156074 -5.559820 -4.867383
C 7.372648 -0.098680 -3.639273
H 5.268116 -0.027299 -4.081203
H 6.020394 -1.583257 -4.422274
C 7.793877 0.477099 -4.987697
H 8.112660 -0.837829 -3.304600
H 7.361301 0.697142 -2.881801
H 7.830303 -0.304646 -5.755546
H 8.787201 0.934265 -4.927368
H 7.090489 1.246770 -5.326375
C 1.879510 0.386369 -3.990494
H 3.655736 0.595054 -2.782960
H 2.242975 0.021996 -1.904799
C 1.361447 1.803693 -3.766390
H 1.032464 -0.307788 -4.081329
H 2.430964 0.335431 -4.939106
H 0.782295 1.867102 -2.837320
H 0.712385 2.123925 -4.588404
H 2.189359 2.518608 -3.694180
C 1.340370 -1.971878 -0.282947
H 2.928461 -2.632202 0.977420
H 2.723141 -3.642528 -0.429067
C 0.300700 -2.660372 0.596861
H 1.015840 -2.017307 -1.329195
H 1.393967 -0.908597 -0.015220
H 0.208009 -3.723550 0.344072
H -0.685843 -2.199883 0.476087
H 0.578201 -2.589296 1.655214
C 8.825887 -2.426481 -0.869041
S 8.907515 -3.782400 -1.707967

[1-CN][NBu$_4$]

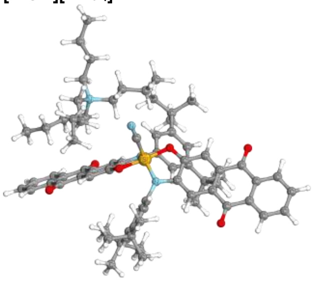

174
xyz, charge: 0, multiplicity: 1
C -1.363773 2.383237 1.332008
C -1.485244 1.253016 2.140608
C -0.122460 2.744509 0.822608
C 1.007335 1.980274 1.116989
C 0.886428 0.845008 1.936097
C -0.364570 0.488571 2.442229
H -2.240273 2.980544 1.097215
H -2.456314 0.969878 2.536868
H -0.013189 3.617294 0.186323
H -0.444014 -0.392808 3.071130
C 2.321145 2.367746 0.536837
C 3.478762 1.511125 0.829455
C 3.369318 0.394303 1.685689
C 2.072252 0.006919 2.264420
C 4.715967 1.836118 0.253940
C 5.809042 1.036418 0.514917
C 5.701961 -0.104559 1.357160
C 4.488601 -0.405702 1.957873
H 4.381595 -1.259393 2.618805
H 4.809733 2.710991 -0.381075
O 2.419598 3.369321 -0.174163
O 1.954830 -0.983938 2.986494
O 7.032953 1.243515 0.033289
N 6.912697 -0.758442 1.467142
C 7.071228 -1.776646 2.460115
C 6.485108 -3.030857 2.282307
C 6.621138 -4.010437 3.263398
C 7.365776 -3.702633 4.413640
C 7.955006 -2.454277 4.609042
C 7.790133 -1.486175 3.610215
H 8.223328 -0.495105 3.718849
H 7.477861 -4.465158 5.176070
H 5.922383 -3.216435 1.374740

C 5.989229 -5.398598 3.128018
C 8.748400 -2.098277 5.869240
C 10.181665 -1.696255 5.473600
H 10.690574 -2.520115 4.960372
H 10.760842 -1.441249 6.369263
H 10.190206 -0.825835 4.808844
C 8.066946 -0.909691 6.573524
H 8.036772 -0.023152 5.931447
H 8.618443 -0.646319 7.483946
H 7.037920 -1.160292 6.856694
C 8.829035 -3.265090 6.859308
H 9.332620 -4.135551 6.423648
H 7.836754 -3.575930 7.205746
H 9.404441 -2.953548 7.738012
C 7.097543 -6.467593 3.156925
H 7.803626 -6.320648 2.331089
H 6.658630 -7.467682 3.058012
H 7.661517 -6.439132 4.094797
C 5.202023 -5.555676 1.822356
H 5.843134 -5.423753 0.943452
H 4.373291 -4.840545 1.758697
H 4.775702 -6.563780 1.774838
C 5.022635 -5.634794 4.304167
H 5.540402 -5.602533 5.268225
H 4.549676 -6.619602 4.209281
H 4.231987 -4.875459 4.317142
H 10.892472 4.968559 -1.227325
H 6.101050 6.593260 3.006398
H 4.992356 4.410779 2.241063
H 8.509407 7.411544 0.397456
H 10.204657 7.367944 2.323480
H 6.972116 2.866760 5.052617
C 13.589583 1.127243 3.836903
C 11.527472 -1.521820 2.069049
C 10.478475 -0.747283 1.622079
C 9.006774 2.597581 1.601329
C 10.440088 0.652414 1.869785
C 11.453374 1.251522 2.603046
C 12.519497 0.468505 3.067578
C 14.701617 0.279696 4.346706
C 15.728515 0.869764 5.085234
C 14.731294 -1.102230 4.093814
C 15.786898 -1.871959 4.584681
C 9.742995 3.594957 0.959948
C 12.563929 -0.915677 2.796076
C 13.650920 -1.760100 3.309191
C 16.776288 0.095869 5.569355
C 16.805239 -1.276200 5.319077
C 7.690049 4.259560 2.764735
C 6.888241 3.956607 5.118293
C 7.996914 2.919002 2.495709
C 10.245786 6.074602 0.549523
C 5.244046 3.990693 3.221893
C 6.371455 6.093088 3.943340
C 10.931982 6.922998 1.636446
C 11.323154 5.558716 -0.410375
C 9.270770 6.961536 -0.248158
C 6.563500 4.585374 3.749789
C 8.436782 5.243679 2.118772
C 9.468341 4.936057 1.216055
N 9.299393 1.223320 1.342139
O 13.677678 -2.975839 3.108970
O 9.420245 -1.210402 0.953067
O 13.574099 2.337592 4.061610
H 6.092861 4.188938 5.836486
H 17.624599 -1.880763 5.697822
H 7.272271 6.571977 4.343593
H 8.758565 6.378770 -1.022559
H 11.628107 6.314288 2.225336
H 5.306673 2.902423 3.120210
H 15.796253 -2.938397 4.381793
H 17.572688 0.560952 6.143435
H 8.219324 6.287040 2.316634
H 11.544715 -2.591050 1.884240
H 7.447164 2.112289 2.974340
H 10.520277 3.295817 0.264565
H 15.692273 1.938542 5.272109
H 11.437838 2.313339 2.825209
H 11.848966 6.410241 -0.855973
H 12.067380 4.941645 0.105876
H 5.558367 6.264227 4.657500
H 11.499711 7.738878 1.173031
H 4.423853 4.218997 3.913206
H 9.818981 7.774690 -0.738759
H 7.832503 4.347216 5.515188
Si 8.226795 0.040548 0.564586
C 8.532527 -0.324107 -1.280875
N 3.980856 -2.563152 -2.141096
C 5.287150 -3.037288 -1.491877
C 3.145782 -3.741507 -2.622410
C 4.252337 -1.618428 -3.295339
C 3.203790 -1.887301 -0.999751
C 1.737485 -1.522410 -1.207122
H 3.781541 -1.004535 -0.717364
H 3.272731 -2.606766 -0.177574
C 4.905167 -0.301947 -2.901623
H 3.293319 -1.445387 -3.789918
H 4.890622 -2.162371 -3.995713
C 6.480376 -3.312690 -2.395809
H 5.016112 -3.933558 -0.922988
H 5.564267 -2.260033 -0.772433
C 3.811387 -4.752329 -3.541441
H 2.282425 -3.303410 -3.130394
H 2.793674 -4.245162 -1.715244
C 2.755265 -5.738313 -4.053965
H 4.584687 -5.312511 -3.004384
H 4.285692 -4.263019 -4.399918
C 3.367670 -6.820245 -4.938526
H 1.988878 -5.188432 -4.616639
H 2.245178 -6.203717 -3.199498
H 3.865671 -6.381092 -5.810860
H 2.601550 -7.512232 -5.303848
H 4.112960 -7.405755 -4.387702
C 7.624813 -3.871985 -1.541949
H 6.823794 -2.391667 -2.879630
H 6.242975 -4.028959 -3.185838
C 8.850157 -4.214973 -2.382726
H 7.278294 -4.767677 -1.008446
H 7.899426 -3.134885 -0.775422
H 8.610077 -4.977152 -3.133423
H 9.660919 -4.602607 -1.756692
H 9.226622 -3.330725 -2.909306
C 5.453803 0.410233 -4.140596
H 5.728997 -0.462553 -2.194591
H 4.179596 0.351082 -2.404936
C 5.987254 1.799507 -3.804452
H 4.662295 0.490038 -4.898916
H 6.253255 -0.201023 -4.581030
H 5.188259 2.443876 -3.418618
H 6.412384 2.285542 -4.689037
H 6.770916 1.744024 -3.039799
C 1.411033 -0.252688 -1.994064
H 1.369645 -1.372083 -0.181427
H 1.161704 -2.370940 -1.592790
C -0.078116 0.074373 -1.908400
H 1.696099 -0.351017 -3.047903
H 1.997296 0.581016 -1.586019
H -0.682058 -0.713933 -2.373744
H -0.308091 1.017357 -2.415921
H -0.399873 0.168424 -0.864081
N 8.743958 -0.491069 -2.408774

NBu$_4$F

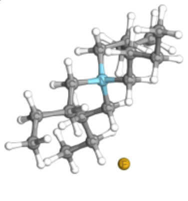

54
xyz, charge: 0, multiplicity: 1
N 9.118066 -1.619227 -6.246824
C 10.222969 -1.413664 -7.272349
C 9.072218 -3.061248 -5.754442
C 9.421424 -0.642909 -5.114320
C 7.755244 -1.340329 -6.862259
C 10.320566 -3.575227 -5.033911
H 8.858816 -3.660432 -6.641842
H 8.197697 -3.128951 -5.100205
C 10.219203 -3.583342 -3.505889
H 11.215489 -3.022221 -5.342671
H 10.483593 -4.605422 -5.374842
C 11.474280 -4.158754 -2.856920
H 10.045901 -2.568161 -3.129872
H 9.342575 -4.176461 -3.212245
H 11.657062 -5.186341 -3.193453
H 11.385980 -4.174020 -1.765257
H 12.358117 -3.561971 -3.112483
C 8.333348 -0.490636 -4.065199
H 10.348523 -1.008357 -4.661255
H 9.636228 0.336152 -5.582087
C 8.862346 0.363463 -2.908495
H 8.000080 -1.460387 -3.672568
H 7.455749 0.010146 -4.492928
C 7.802301 0.598646 -1.837097
H 9.213240 1.325511 -3.304644
H 9.736695 -0.130251 -2.462257
H 6.933362 1.123744 -2.250613
H 8.197515 1.204726 -1.014697
H 7.450856 -0.350367 -1.414951
C 7.600445 0.021694 -7.516933
H 7.031911 -1.461361 -6.050952
H 7.570655 -2.140753 -7.584737
C 6.125935 0.277981 -7.841548
H 8.182121 0.066921 -8.446252
H 7.983542 0.817446 -6.866449
C 5.923105 1.616096 -8.546169
H 5.542140 0.257888 -6.911117
H 5.739449 -0.535211 -8.471846
H 6.283271 2.444530 -7.925201
H 4.864312 1.794000 -8.763961
H 6.471249 1.648508 -9.495028
C 10.190131 -2.330841 -8.482525
H 11.168872 -1.521056 -6.734377
H 10.148135 -0.366648 -7.576833
C 11.279972 -1.910783 -9.474710
H 9.215622 -2.282127 -8.983922
H 10.360431 -3.374299 -8.189863
C 11.307048 -2.807227 -10.709038
H 12.257457 -1.939060 -8.974245
H 11.111816 -0.868404 -9.776797
H 11.500660 -3.851169 -10.435252
H 12.089826 -2.493535 -11.408006
H 10.349154 -2.772156 -11.241399
F 9.975170 2.084757 -6.255524

PPh$_4$Cl

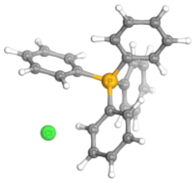

46
xyz, charge: 0, multiplicity: 1
C 3.854825 -0.873037 -8.583807
C 4.270372 -0.517758 -7.306538
C 2.776350 -1.739468 -8.753003
C 2.110032 -2.253638 -7.644315
C 2.515509 -1.903996 -6.360188
C 3.595400 -1.033424 -6.192665
H 4.377753 -0.474814 -9.448274
H 5.115078 0.154018 -7.178066
H 2.457772 -2.017741 -9.753654
H 1.273976 -2.934146 -7.775375
H 1.996788 -2.313783 -5.498680
C 5.919341 -0.564862 -4.475756
C 6.622609 0.379842 -3.723157
C 6.602227 -1.588114 -5.145577
C 8.008149 0.294690 -3.639414
C 7.986797 -1.662490 -5.053174
C 8.689047 -0.723536 -4.300606
H 6.101118 1.179849 -3.207738
H 6.059689 -2.321201 -5.736046
H 8.553943 1.029552 -3.055358
H 8.516796 -2.455577 -5.572234
H 9.771588 -0.784662 -4.232391
C 1.600216 -2.334373 -1.892054
C 2.363039 -3.401003 -1.422133
C 2.129630 -1.458688 -2.831963
C 3.657641 -3.598800 -1.894206
C 3.433155 -1.658565 -3.301617
C 4.199471 -2.729376 -2.834096
H 0.592586 -2.175911 -1.519522
H 1.948970 -4.075617 -0.678091
H 1.535453 -0.622369 -3.189591

H	4.256417	-4.423495	-1.519461
H	5.217339	-2.877926	-3.181264
C	2.612014	3.704592	-3.599960
C	3.064937	2.877222	-2.574519
C	2.598434	3.246834	-4.915064
C	3.505374	1.588719	-2.854257
C	3.040438	1.962034	-5.212646
C	3.496824	1.136903	-4.180516
H	2.264223	4.708693	-3.373019
H	3.074071	3.230100	-1.547152
H	2.238292	3.887927	-5.714301
H	3.857708	0.954387	-2.038302
H	3.018886	1.608654	-6.238904
P	4.111034	-0.525798	-4.535484
Cl	4.885558	0.102019	0.174257

NBu_4N_3

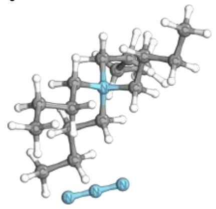

56

xyz, charge: 0, multiplicity: 1

N	9.179141	-1.616235	-6.211664
C	10.288533	-1.456374	-7.242480
C	9.068648	-3.060978	-5.731299
C	9.533549	-0.678014	-5.063838
C	7.824313	-1.268267	-6.814621
C	10.299736	-3.642279	-5.032339
H	8.813135	-3.636857	-6.622634
H	8.200799	-3.091772	-5.065922
C	10.196416	-3.690406	-3.505079
H	11.213543	-3.111989	-5.324590
H	10.427057	-4.666588	-5.404081
C	11.434368	-4.316196	-2.870959
H	10.048398	-2.681151	-3.102274
H	9.303585	-4.267477	-3.228704
H	11.592357	-5.337652	-3.237474
H	11.341954	-4.362001	-1.780675
H	12.333575	-3.734499	-3.106425
C	8.457957	-0.473476	-4.010831
H	10.443489	-1.091229	-4.617832
H	9.803596	0.280784	-5.516261
C	9.051078	0.300518	-2.828971
H	8.057621	-1.429701	-3.651814
H	7.622203	0.102477	-4.425563
C	7.992526	0.660326	-1.791941
H	9.527432	1.217005	-3.202684
H	9.842977	-0.301830	-2.363157
H	7.216592	1.298459	-2.230771
H	8.433359	1.202538	-0.948674
H	7.504422	-0.238290	-1.395897
C	7.721692	0.111349	-7.438620
H	7.100358	-1.376594	-6.002058
H	7.607683	-2.045562	-7.552774
C	6.268559	0.409410	-7.820345
H	8.346432	0.179058	-8.337050
H	8.061835	0.882410	-6.738766
C	6.130702	1.782988	-8.470718
H	5.641199	0.362035	-6.919911
H	5.899069	-0.367000	-8.504488
H	6.484316	2.570859	-7.794683
H	5.087598	1.999264	-8.725239
H	6.721530	1.843589	-9.392232
C	10.207151	-2.365361	-8.456064
H	11.230641	-1.610031	-6.708991
H	10.260988	-0.406623	-7.546829
C	11.321463	-2.003695	-9.444063
H	9.238256	-2.260852	-8.959649
H	10.318789	-3.417165	-8.166281
C	11.299042	-2.896042	-10.681352
H	12.294356	-2.090560	-8.941674
H	11.214129	-0.952369	-9.742443
H	11.429954	-3.950290	-10.410507
H	12.101148	-2.626784	-11.376649
H	10.346713	-2.802798	-11.216430
N	10.400890	2.583858	-6.588049
N	9.448952	2.840079	-5.939775
N	8.491906	3.082294	-5.294708

NBu₄NCS

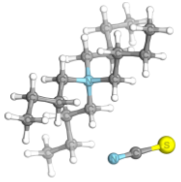

56

xyz, charge: 0, multiplicity: 1

N	-0.971152	1.191918	0.271562
C	-0.953802	2.308670	1.308327
C	-2.180203	1.305389	-0.645503
C	-0.997818	-0.107496	1.066287
C	0.252633	1.258818	-0.631408
C	-0.911706	3.725800	0.766298
H	-0.086072	2.111918	1.944232
H	-1.844463	2.160854	1.925016
C	-0.866340	4.712692	1.938351
H	-1.794713	3.941575	0.152279
H	-0.025376	3.880852	0.139250
C	1.598476	1.197885	0.068705
H	0.159472	2.188051	-1.200569
H	0.151117	0.431169	-1.339037
C	2.722764	1.236002	-0.971502
H	1.692416	0.280518	0.661674
H	1.720480	2.043391	0.755520
C	4.101549	1.191691	-0.319231
H	2.628197	2.148002	-1.576645
H	2.610776	0.387655	-1.660340
H	4.246476	2.045405	0.352898
H	4.896471	1.218041	-1.072297
H	4.228458	0.276935	0.271358
C	-0.999510	-1.391274	0.256562
H	-1.886542	-0.054306	1.699974
H	-0.130494	-0.074275	1.731389
C	-1.058558	-2.588064	1.212788
H	-0.093360	-1.471345	-0.355913
H	-1.862364	-1.432684	-0.419402
C	-1.049742	-3.919255	0.467489
H	-1.963682	-2.513702	1.830401
H	-0.205184	-2.542187	1.902714
H	-1.909088	-3.998896	-0.208775
H	-1.093646	-4.761949	1.165618
H	-0.139215	-4.029348	-0.133175
C	-3.539131	1.241224	0.029720
H	-2.081471	0.500838	-1.379759
H	-2.065195	2.251791	-1.181450
C	-4.642824	1.379179	-1.024507
H	-3.649396	2.045725	0.766414
H	-3.669837	0.289403	0.558047
C	-6.035537	1.326532	-0.403378
H	-4.537956	0.577094	-1.767591
H	-4.513379	2.327203	-1.563996
H	-6.198191	0.377259	0.120283
H	-6.813236	1.424253	-1.168303
H	-6.174195	2.136801	0.321860
C	-0.818754	6.162260	1.464975
H	0.012875	4.494668	2.559500
H	-1.747084	4.558994	2.576284
H	0.069446	6.347082	0.849383
H	-0.787075	6.853885	2.313565
H	-1.701286	6.410491	0.863568
N	1.440396	1.217415	3.831519
C	0.462556	0.805463	4.349492
S	-0.907418	0.231745	5.065139

NBu₄CN

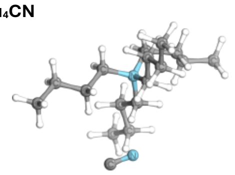

55

xyz, charge: 0, multiplicity: 1

N	8.950802	-1.629629	-6.001321
C	8.804870	-1.895411	-7.494552
C	8.977017	-2.928049	-5.200520
C	10.249961	-0.849476	-5.843553
C	7.764151	-0.834303	-5.474257
C	10.115502	-3.903401	-5.506201
H	8.007405	-3.394991	-5.384643
H	8.997198	-2.634059	-4.147133
C	11.293127	-3.853307	-4.528033
H	10.480248	-3.785442	-6.533168
H	9.681760	-4.910664	-5.467701
C	12.335253	-4.921822	-4.844847
H	11.768984	-2.865608	-4.547333
H	10.913016	-3.996017	-3.507456
H	11.896452	-5.925897	-4.803034
H	13.165734	-4.889169	-4.131733
H	12.750898	-4.780752	-5.849655
C	10.514391	-0.269686	-4.464577
H	11.045550	-1.541187	-6.136713
H	10.222850	-0.052375	-6.592958
C	11.921519	0.334761	-4.425552
H	10.431970	-1.036588	-3.684519
H	9.786292	0.516541	-4.232451
C	12.237202	0.958163	-3.069292
H	12.010311	1.093918	-5.214384
H	12.658734	-0.446180	-4.656382
H	11.526774	1.757492	-2.828493
H	13.243642	1.389743	-3.056962
H	12.183606	0.209465	-2.269915
C	7.500939	0.489466	-6.168421
H	7.957181	-0.679041	-4.409165
H	6.892796	-1.489913	-5.556233
C	6.397269	1.251579	-5.428393
H	7.188505	0.327430	-7.206991
H	8.405228	1.107999	-6.198247
C	6.067846	2.577002	-6.108672
H	6.716117	1.435325	-4.393321
H	5.494639	0.627374	-5.373605
H	6.951015	3.224608	-6.155583
H	5.283371	3.115996	-5.566573
H	5.718116	2.416953	-7.135151
C	7.592398	-2.704558	-7.918320
H	9.726128	-2.393023	-7.809986
H	8.799355	-0.912085	-7.972841
C	7.556925	-2.798220	-9.447836
H	6.664591	-2.234111	-7.570986
H	7.628167	-3.717849	-7.500139
C	6.361108	-3.604312	-9.945604
H	8.488729	-3.257261	-9.804717
H	7.523859	-1.785407	-9.871078
H	6.386212	-4.629746	-9.558566
H	6.351088	-3.659633	-11.039380
H	5.417351	-3.147516	-9.625256
C	9.917535	2.229885	-8.529223
N	10.268594	1.169849	-8.883873

[CN]

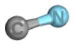

2

xyz, charge: -1, multiplicity: 1

N	-2.793857	1.071710	-0.090971
C	-1.709193	1.449540	0.153971

[N_3]

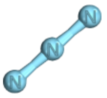

3

xyz, charge: -1, multiplicity: 1

N	4.592661	-0.560410	-5.980582
N	3.572312	-0.910174	-5.496611
N	5.613007	-0.210646	-6.464557

[NCS]

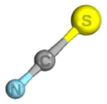

3

xyz, charge: -1, multiplicity: 1

C	-4.147592	-0.235957	0.019214
N	-3.693129	0.851265	0.004908
S	-4.788149	-1.768409	0.039298

MeCN

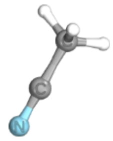

6

xyz, charge: 0, multiplicity: 1

C	2.946713	8.186556	14.545857
C	2.620733	7.731046	13.207968
N	2.361164	7.368368	12.142796
H	2.586516	9.208484	14.692760
H	4.029800	8.168804	14.695082
H	2.476983	7.536151	15.288816

Et_2O

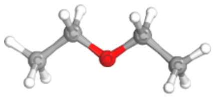

15

xyz, charge: 0, multiplicity: 1

C	0.558388	7.522754	6.128818
C	0.981066	7.115168	7.524461
H	1.937659	6.585125	7.495444
H	0.234286	6.453358	7.973006
H	1.091307	7.998515	8.161426
O	0.428787	6.348472	5.338832
H	1.305891	8.200349	5.678331
H	-0.402780	8.066987	6.156741
C	0.032903	6.632257	4.003709
C	-0.077543	5.323708	3.249973
H	0.773678	7.296592	3.523365
H	-0.935060	7.165050	4.002032
H	0.884604	4.803049	3.243236
H	-0.382341	5.505997	2.214578
H	-0.818585	4.671509	3.721518

THF

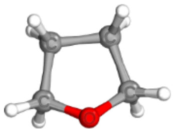

13

xyz, charge: 0, multiplicity: 1

C	0.411222	7.811441	5.984014
C	0.719111	6.312961	6.148021
O	-0.548355	7.889463	4.930663
H	1.322375	8.368661	5.703944
H	-0.026272	8.276545	6.871939
C	-0.098110	6.948740	3.956542
C	0.372396	5.721856	4.757324
H	0.736469	7.379736	3.376241
H	-0.930385	6.744919	3.276905
H	1.228294	5.233410	4.282194
H	-0.430430	4.984110	4.840198
H	0.086909	5.872086	6.923680
H	1.762536	6.143181	6.429686

Pyridine

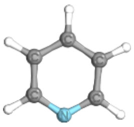

11

xyz, charge: 0, multiplicity: 1

C	4.353718	-4.321812	-0.725173
C	4.196662	-3.020725	-1.194232
C	3.365849	-4.855346	0.094174
C	2.265566	-4.066405	0.408564
C	2.202432	-2.776796	-0.111485
N	3.144417	-2.250052	-0.900548
H	5.231396	-4.900180	-0.997998
H	4.953840	-2.574189	-1.838488
H	3.451839	-5.867149	0.480505
H	1.468828	-4.439993	1.044885
H	1.352662	-2.133713	0.116775

DMAP

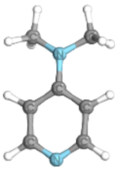

19

xyz, charge: 0, multiplicity: 1

C	2.053708	-0.995674	-4.151777
C	1.826924	-1.802107	-5.255463
C	1.128694	-1.004608	-3.086890
C	0.031159	-1.878912	-3.231036
C	-0.086152	-2.637559	-4.384533
N	0.780136	-2.622699	-5.404662
H	2.941520	-0.375223	-4.130110
H	2.545111	-1.787477	-6.075798
H	-0.724724	-1.976302	-2.461106
H	-0.938867	-3.308956	-4.489708
N	1.282499	-0.204650	-1.981598
C	2.537480	0.504332	-1.784748
H	3.396833	-0.177850	-1.681120
H	2.467775	1.110122	-0.879232
H	2.738495	1.184497	-2.620649
C	0.427576	-0.417496	-0.823946
H	-0.628435	-0.285888	-1.087182
H	0.669309	0.323745	-0.059797
H	0.551134	-1.421178	-0.385839

$OPEt_3$

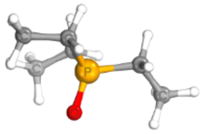

23

xyz, charge: 0, multiplicity: 1

O	1.818422	1.451259	-4.131825
P	2.334900	0.096244	-3.741574
C	1.091377	-0.953858	-2.886562
C	3.806892	0.170618	-2.626682
C	2.869926	-0.937193	-5.166145
C	3.736758	-0.146618	-6.149099
H	1.958352	-1.292570	-5.664352
H	3.396832	-1.823710	-4.786635
H	3.208231	0.748825	-6.487226
H	3.985177	-0.757210	-7.022695
H	4.677456	0.175832	-5.690836
C	3.810496	1.429528	-1.755123
H	4.699936	0.157742	-3.264721
H	3.841191	-0.743446	-2.018484
H	3.732426	2.322798	-2.380137
H	4.733575	1.487515	-1.169520
H	2.966187	1.445748	-1.060473
C	0.595931	-0.349798	-1.570486
H	1.513707	-1.956161	-2.731273
H	0.257497	-1.060106	-3.593297
H	1.384846	-0.340896	-0.811780
H	-0.240646	-0.930494	-1.169523
H	0.256183	0.679333	-1.721739

HMPA

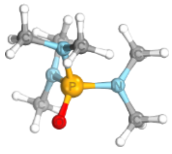

29

xyz, charge: 0, multiplicity: 1

O	1.510391	1.696556	-3.866573
P	2.200788	0.380099	-3.751109
N	1.429855	-0.913421	-2.976741
N	3.601578	0.535156	-2.820187
N	2.472829	-0.272901	-5.287135
C	2.633237	0.657254	-6.403974
H	2.259358	0.188380	-7.324262
H	3.688345	0.933719	-6.568816
H	2.056493	1.563993	-6.210979
C	4.262489	1.837477	-2.771711
H	5.069887	1.921605	-3.518251
H	4.705169	1.986546	-1.777175
H	3.529756	2.626506	-2.952530
C	1.203321	-0.773251	-1.531417
H	2.088164	-0.358187	-1.044726
H	0.998392	-1.763472	-1.105578
H	0.346127	-0.117300	-1.311739
C	0.243732	-1.469150	-3.643511
H	0.420594	-1.568595	-4.716231
H	-0.645254	-0.835816	-3.495416
H	0.033819	-2.462114	-3.226570
C	3.174672	-1.537108	-5.476590
H	2.973759	-2.214602	-4.642423
H	4.265969	-1.407337	-5.569507
H	2.818578	-2.018933	-6.398082
C	4.490145	-0.586257	-2.541696
H	3.930806	-1.525388	-2.537738
H	4.944752	-0.458257	-1.548734
H	5.310009	-0.667360	-3.274578

DMSO

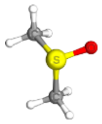

10

xyz, charge: 0, multiplicity: 1

O	1.534856	-1.960244	-4.631953
S	1.490977	-0.545107	-4.089562
C	0.802866	-0.665515	-2.381992
H	0.866892	0.305815	-1.881745
H	1.360038	-1.431699	-1.834971
H	-0.242552	-0.965772	-2.484735
C	3.211291	-0.138995	-3.561047
H	3.813710	-0.079234	-4.470515
H	3.579101	-0.946478	-2.921208
H	3.226461	0.821740	-3.037041

DIBA

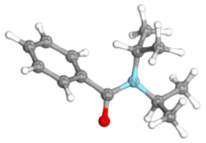

34

xyz, charge: 0, multiplicity: 1

O	-0.584038	-2.224236	-1.621757
C	-0.820852	-1.419445	-0.722410
N	-2.076002	-1.249778	-0.188508
C	0.324416	-0.618472	-0.164401
C	0.719422	-0.729567	1.170286
C	1.089178	0.148640	-1.044561
C	2.211911	0.827994	-0.586426
C	2.599425	0.720259	0.747014
C	1.857489	-0.069054	1.621201
H	2.166881	-0.173173	2.657296
H	0.143290	-1.349020	1.852048
H	2.794196	1.434011	-1.274621
H	3.483758	1.241896	1.101408
H	0.799475	0.197760	-2.090283
C	-2.395454	-0.057340	0.626636
C	-3.421196	0.869846	-0.031108
H	-3.122924	1.127525	-1.051519
H	-3.485981	1.795191	0.551240
H	-4.422427	0.431402	-0.060348
C	-2.776409	-0.369060	2.079826
H	-1.457915	0.504516	0.665057
H	-2.672257	0.542049	2.679104
H	-2.118811	-1.131443	2.506367
H	-3.808549	-0.711979	2.179692
C	-3.107798	-2.165395	-0.740062
C	-4.219723	-2.509945	0.245715
H	-3.820593	-2.886397	1.191689
H	-4.833544	-3.300410	-0.198287
H	-4.883605	-1.665881	0.453316
C	-3.666609	-1.684359	-2.084018
H	-2.553333	-3.087568	-0.945441
H	-4.234464	-2.496166	-2.551729
H	-2.850674	-1.413965	-2.759039
H	-4.338311	-0.829016	-1.970389

^{dipp}NHC

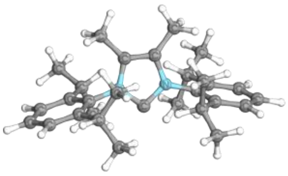

71
xyz, charge: 0, multiplicity: 1
C 4.888097 0.666809 8.677576
N 5.339119 -0.084512 7.582004
C 4.033473 1.590623 8.165085
N 3.998149 1.347423 6.783168
C 4.790819 0.303771 6.389972
C 6.236967 -1.192738 7.733521
C 7.612548 -1.031691 7.497397
C 8.434870 -2.140854 7.718020
C 7.920329 -3.353940 8.151655
C 6.556227 -3.491126 8.364122
C 5.691269 -2.415643 8.159468
H 8.584863 -4.196913 8.319195
H 6.158240 -4.447897 8.687889
H 9.503132 -2.042072 7.540653
C 8.270383 0.225209 6.945272
C 8.038617 0.330670 5.428864
H 6.968985 0.413921 5.208207
H 8.559148 1.208340 5.026897
H 8.418703 -0.559753 4.917161
C 7.926806 1.538906 7.658054
H 9.347913 0.069904 7.096707
H 6.904343 1.868634 7.463883
H 8.059879 1.452330 8.741764
H 8.601287 2.325938 7.302251
C 4.191244 -2.600303 8.298183
C 3.792980 -3.494758 9.476248
H 4.074388 -4.539936 9.309018
H 2.706154 -3.471665 9.608768
H 4.259406 -3.164932 10.410892
C 3.630893 -3.150347 6.976585
H 3.736236 -1.616354 8.461163
H 2.541932 -3.258958 7.035073
H 4.063750 -4.134294 6.761146
H 3.872272 -2.477980 6.147494
C 5.324384 0.402828 10.072664
H 4.964265 1.190501 10.739106
H 6.416325 0.359173 10.147807
H 4.939589 -0.555920 10.442439
C 3.202048 2.125244 5.880453
C 1.824830 1.868084 5.812267
C 1.049551 2.676859 4.979813
C 1.629787 3.698384 4.240526
C 2.999859 3.915682 4.302395
C 3.816800 3.131870 5.119069
C 5.313054 3.375064 5.191218
H -0.019036 2.501228 4.901738
H 1.011765 4.321400 3.599925
H 3.441263 4.704410 3.701198
C 5.915336 3.810796 3.852590
H 5.622783 3.131721 3.045565
H 7.008109 3.805695 3.923801
H 5.613838 4.827050 3.574670
C 5.650246 4.393532 6.290511
H 5.780014 2.421225 5.457317
H 5.333866 4.038752 7.276399
H 5.153683 5.351460 6.094938
H 6.731299 4.568925 6.329700
C 1.210708 0.683238 6.533599
C -0.188964 0.964686 7.087906
H -0.207667 1.875676 7.695854
H -0.516943 0.127682 7.713299
H -0.928474 1.077896 6.287970
C 1.193923 -0.525506 5.583933
H 1.857875 0.422536 7.379326
H 0.564673 -0.314435 4.711421
H 0.792106 -1.409599 6.091947
H 2.204954 -0.752690 5.232044
C 3.220615 2.646634 8.824444
H 2.157674 2.376324 8.861985
H 3.287810 3.598703 8.287265
H 3.562980 2.805550 9.850163

SIMes

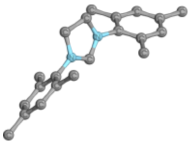

49
xyz, charge: 0, multiplicity: 1
C -5.09921 -0.89364 -4.42544
N -5.56627 -2.27216 -4.09847
C -5.86538 -0.06126 -3.39859
N -6.35643 -1.11765 -2.46674
C -6.22887 -2.37511 -2.93083
H -4.00929 -0.82791 -4.29663
H -5.23432 0.66698 -2.87944
H -6.71842 0.47483 -3.83879
H -5.33850 -0.62589 -5.45941
C -7.13570 -0.76300 -1.32783
C -8.53050 -0.91461 -1.36855
C -6.49741 -0.25045 -0.19252
C -9.27140 -0.54011 -0.25051
C -7.27936 0.11755 0.90420
C -8.66481 -0.01579 0.89206
H -10.35313 -0.66002 -0.27196
H -6.78744 0.50452 1.79467
C -5.08829 -3.38765 -4.84485
C -4.07090 -4.19362 -4.31053
C -5.62330 -3.64512 -6.11246
C -3.60157 -5.26121 -5.07173
C -5.11982 -4.72286 -6.84347
C -4.11200 -5.54058 -6.34039
H -2.81294 -5.89133 -4.66434
H -5.53775 -4.93267 -7.82638
C -3.50664 -3.91842 -2.94538
H -4.26773 -4.07936 -2.17454
H -2.65027 -4.56691 -2.74300
H -3.18028 -2.87645 -2.84971
C -3.60470 -6.71969 -7.12592
H -4.05544 -7.65208 -6.76454
H -3.84598 -6.62523 -8.18831
H -2.51944 -6.82437 -7.02854
C -6.73011 -2.79869 -6.67810
H -7.30158 -3.36003 -7.42217
H -7.41294 -2.46576 -5.89063
H -6.33896 -1.90314 -7.17770
C -9.20849 -1.48408 -2.58256
H -8.91928 -0.94486 -3.49199
H -8.91654 -2.52891 -2.73243
H -10.29536 -1.42745 -2.48081
C -9.49620 0.40552 2.07358
H -10.28148 -0.32659 2.28752
H -8.88289 0.52142 2.97142
H -9.99004 1.36598 1.88192
C -5.00051 -0.12022 -0.13278
H -4.65585 -0.11633 0.90473
H -4.51230 -0.94480 -0.66098
H -4.65761 0.81571 -0.59212

^{iPr}NHC

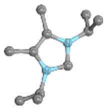

33
xyz, charge: 0, multiplicity: 1
C -4.90695 -0.86512 -4.24948
N -5.27402 -2.19202 -4.00577
C -5.61027 -0.11138 -3.35348
N -6.37192 -1.01519 -2.60662
C -6.18801 -2.30954 -2.99889
C -7.35495 -0.71629 -1.55455
C -4.85483 -3.39554 -4.73946
C -5.48047 -3.45319 -6.13282
H -5.08999 -2.67190 -6.79252
H -6.56615 -3.33856 -6.06470
H -5.26135 -4.42022 -6.59790
C -3.33859 -3.58224 -4.76596
H -5.29092 -4.20171 -4.13860
H -2.91345 -3.44355 -3.76717
H -2.84529 -2.89221 -5.45628
H -3.10655 -4.59920 -5.09839
C -6.76824 0.09462 -0.40011
H -5.81377 -0.32979 -0.07383
H -6.61352 1.14455 -0.66413
H -7.46140 0.06899 0.44690
C -8.62403 -0.08886 -2.13071
H -7.61028 -1.71415 -1.18009
H -8.44405 0.91568 -2.52655
H -9.38502 -0.00540 -1.34750
H -9.02177 -0.71271 -2.93636
C -5.55347 1.36323 -3.14096
H -6.54783 1.81221 -3.06010
H -4.99537 1.62939 -2.23534
H -5.05020 1.84134 -3.98507
C -3.89793 -0.41011 -5.24835
H -2.87297 -0.64433 -4.93682
H -4.05412 -0.85817 -6.23410
H -3.96014 0.67395 -5.37218

PBu_3

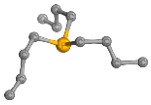

40
xyz, charge: 0, multiplicity: 1
P 5.68471 -0.37930 4.78930
C 5.85787 -2.20725 5.17415
H 5.21472 -2.78062 4.48998
H 6.89616 -2.48767 4.94974
C 5.54337 -2.58146 6.62696
H 5.65858 -3.66934 6.73866
H 4.48812 -2.36939 6.84826
C 6.42755 -1.88250 7.66005
H 6.29232 -0.79581 7.58578
H 7.48232 -2.07080 7.41588
C 5.71584 -0.49183 2.91706
H 4.90789 -1.16030 2.58409
H 5.48468 0.50968 2.52903
C 7.05505 -0.95512 2.33241
H 6.95847 -1.00381 1.23809
H 7.27131 -1.98204 2.65825
C 8.23808 -0.05265 2.68411
H 8.37002 -0.02600 3.77340
H 8.00404 0.97859 2.38507
C 3.82931 -0.22689 5.02219
H 3.33655 -1.09616 4.56170
H 3.63372 -0.28166 6.10193
C 3.22579 1.07038 4.47211
H 2.13993 1.04914 4.64441
H 3.35351 1.11009 3.38143
C 3.80005 2.34276 5.09564
H 3.69788 2.28465 6.18826
H 4.87842 2.39557 4.89804
C 9.53322 -0.50635 2.01471
H 9.43537 -0.50411 0.92290
H 10.37085 0.14777 2.27687
H 9.79709 -1.52530 2.32073
C 6.13308 -2.34657 9.08456
H 6.29851 -3.42498 9.19079
H 6.77184 -1.83676 9.81279
H 5.09048 -2.14394 9.35560
C 3.11411 3.60404 4.57604
H 3.22739 3.69297 3.48940
H 2.04037 3.58891 4.79621
H 3.53608 4.50631 5.03001

PCy_3

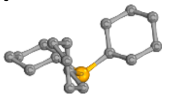

52
xyz, charge: 0, multiplicity: 1
P 5.13584 -1.77786 12.57622
C 4.75592 -1.68055 10.73485
H 3.78307 -2.15114 10.52297
C 5.82352 -2.40521 9.90008
H 5.85060 -3.47236 10.14919
H 6.81406 -1.99527 10.14862
C 5.56611 -2.25005 8.39698
H 4.61964 -2.74912 8.14150
H 6.35515 -2.76101 7.83084
C 5.48318 -0.77897 7.98975
H 5.26731 -0.69010 6.91776

H 6.45946 -0.30126 8.15838
C 4.42017 -0.05109 8.81256
H 3.42957 -0.46720 8.57601
H 4.38856 1.01259 8.54480
C 4.68466 -0.20252 10.31351
H 5.63985 0.28146 10.56689
H 3.90797 0.32576 10.87928
C 3.57757 -0.99558 13.31035
H 3.78958 0.08047 13.18433
C 2.21056 -1.25780 12.66036
H 1.93265 -2.31146 12.78186
H 2.24353 -1.06421 11.58246
C 1.13003 -0.37739 13.30200
H 0.15679 -0.58088 12.83731
H 1.36169 0.67923 13.10078
C 1.05506 -0.59728 14.81392
H 0.73291 -1.63056 15.01159
H 0.29785 0.06024 15.25900
C 2.41704 -0.36375 15.47003
H 2.36245 -0.56625 16.54720
H 2.69845 0.69364 15.35951
C 3.49581 -1.24014 14.82551
H 4.47124 -1.05658 15.29453
H 3.24657 -2.29445 15.00945
C 5.05852 -3.63470 12.88952
H 5.78463 -4.01280 12.15020
C 5.64234 -4.00284 14.26531
H 4.96752 -3.66337 15.06209
H 6.59517 -3.48134 14.41706
C 5.83490 -5.51827 14.38863
H 6.22835 -5.76770 15.38221
H 6.58974 -5.84472 13.65798
C 4.52716 -6.26947 14.12988
H 3.80233 -6.01271 14.91680
H 4.68979 -7.35276 14.19169
C 3.93830 -5.89583 12.76801
H 2.98408 -6.41420 12.60867
H 4.61919 -6.23280 11.97234
C 3.74008 -4.37944 12.65120
H 3.32088 -4.12691 11.66936
H 3.00503 -4.06431 13.40409

DABCO

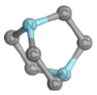

20
xyz, charge: 0, multiplicity: 1
C 2.61634 3.22035 3.52392
C 1.97133 1.95160 2.88361
N 2.57689 3.12792 4.99420
H 2.08657 4.13387 3.22518
H 3.66483 3.33348 3.22021
C 1.17419 2.96996 5.41758
C 0.56435 1.67302 4.79965
H 0.62227 3.86344 5.09910
H 1.14996 2.94147 6.51441
N 1.55194 1.00569 3.93298
H -0.32420 1.89604 4.19539
H 0.26267 0.96151 5.57881
H 2.67631 1.43460 2.22018
H 1.08672 2.20928 2.28743
C 3.33091 1.93203 5.41021
C 2.72944 0.65404 4.74643
H 3.29133 1.87125 6.50525
H 4.38128 2.07512 5.12619
H 2.41669 -0.08000 5.49995
H 3.45886 0.16211 4.09044

p-F-PhOH

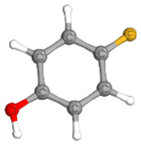

13
xyz, charge: 0, multiplicity: 1
C -5.012230 3.715799 -0.000000
C -5.023132 2.332543 0.000000
C -3.787010 4.377197 -0.000000
C -2.598702 3.647488 -0.000000
C -2.633212 2.253232 -0.000000
C -3.853362 1.589484 -0.000000
H -5.947796 4.265027 -0.000000
F -6.212876 1.684767 0.000000
H -3.762942 5.464734 -0.000000
H -1.699083 1.701113 -0.000000
H -3.902867 0.505637 0.000000
O -1.365280 4.242178 0.000000
H -1.474446 5.199542 0.000000

p-F-PhOH-F

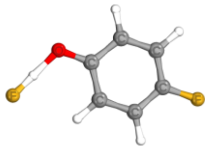

14
xyz, charge: -1, multiplicity: 1
C -4.806586 3.807589 0.000000
C -4.968666 2.431750 -0.000000
C -3.525121 4.346818 0.000000
C -2.362466 3.519240 0.000000
C -2.595655 2.113436 0.000000
C -3.879160 1.577454 -0.000000
H -5.683739 4.450598 -0.000000
F -6.243066 1.900147 -0.000000
H -3.380723 5.423891 0.000000
H -1.728141 1.458300 0.000000
H -4.039799 0.501812 -0.000000
O -1.154689 3.994613 -0.000000
H -1.075509 5.325628 0.000000
F -1.002571 6.376134 -0.000000

p-F-PhOH-Cl

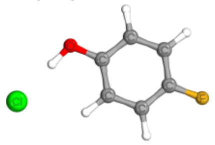

14
xyz, charge: -1, multiplicity: 1
C -4.784228 3.826373 -0.000535
C -4.973609 2.454891 0.000320
C -3.491316 4.339098 -0.001207
C -2.384825 3.469092 -0.000963
C -2.613611 2.083056 -0.000004
C -3.908305 1.572498 0.000604
H -5.644923 4.489157 -0.000675
F -6.251555 1.954844 0.000931
H -3.305399 5.410829 -0.001698
H -1.756225 1.416302 0.000230
H -4.091934 0.501977 0.001328
O -1.117468 3.899019 -0.001855
H -1.077391 4.916418 -0.000462
Cl -1.045101 6.893857 0.003985

p-F-PhOH-Br

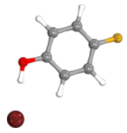

14
xyz, charge: -1, multiplicity: 1
Br 2.99902 0.49887 0.68326
O 0.07508 -0.30769 -0.28933
C -0.10541 -1.63438 -0.19601
C 0.88684 -2.51385 0.27059
C 0.63236 -3.87954 0.34158
C -0.60423 -4.36230 -0.05080
C -1.60124 -3.52261 -0.51441
C -1.34843 -2.15617 -0.58604
H 1.84783 -2.10210 0.57127
H -2.56025 -3.93472 -0.81424
H -2.10990 -1.47030 -0.94539
H 1.39164 -4.56824 0.70050
F -0.85042 -5.70905 0.02066
H 0.99810 -0.04617 0.01669

p-F-PhOH-N_3

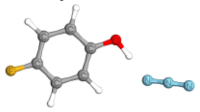

16
xyz, charge: -1, multiplicity: 1
C 2.808972 3.489769 0.745826
C 1.465803 3.714114 0.498385
C 3.749909 3.843095 -0.216085
C 3.343417 4.423603 -1.434480
C 1.969186 4.637272 -1.647701
C 1.029539 4.283456 -0.684912
H 3.115706 3.041413 1.686552
F 0.541460 3.363120 1.449517
H 4.810110 3.680172 -0.046190
H 1.656713 5.086667 -2.585796
H -0.032012 4.446936 -0.846991
O 4.198372 4.778448 -2.393839
N 6.634195 4.210396 -1.679465
N 7.559267 4.932715 -1.889379
N 8.484958 5.614128 -2.081049
H 5.188545 4.576695 -2.118114

p-F-PhOH-NCS

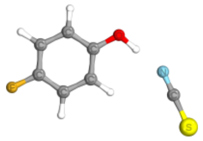

16
xyz, charge: -1, multiplicity: 1
C 3.066512 3.552645 0.621295
C 1.684818 3.574750 0.548433
C 3.813558 4.072177 -0.431167
C 3.171816 4.615163 -1.559230
C 1.767711 4.620353 -1.598347
C 1.021333 4.100898 -0.546001
H 3.555641 3.132038 1.494802
F 0.951652 3.062689 1.586212
H 4.898744 4.063555 -0.392813
H 1.276303 5.040232 -2.471014
H -0.064383 4.102384 -0.570921
O 3.833417 5.129897 -2.602910
N 6.454739 4.983699 -2.275125
C 7.438832 4.696274 -1.690568
S 8.811830 4.300605 -0.885907
H 4.841613 5.074643 -2.470460

p-F-PhOH-CN

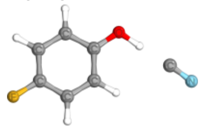

15
xyz, charge: -1, multiplicity: 1
C -4.531939 3.997149 0.000000
C -4.864962 2.653696 -0.000000
C -3.191768 4.369677 0.000000
C -2.171031 3.393346 0.000000
C -2.555572 2.037162 -0.000000
C -3.896035 1.665926 -0.000000
H -5.317895 4.747161 0.000001
F -6.188951 2.290118 -0.000000
H -2.913316 5.418844 0.000001
H -1.774081 1.282744 -0.000000
H -4.190596 0.620198 -0.000001
O -0.877496 3.690678 0.000000
C -0.378950 6.316052 0.000000
N -0.230629 7.473834 -0.000001
H -0.691980 4.753494 0.000000

p-F-PhOH-pyridine

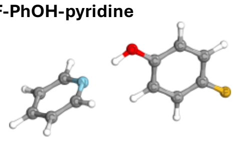

24
xyz, charge: 0, multiplicity: 1

C	-1.375291	5.486940	0.012714
C	-1.424102	4.103038	0.053686
C	-0.138077	6.118337	0.026092
C	1.041099	5.367426	0.079976
C	0.963062	3.970063	0.120093
C	-0.275833	3.334170	0.107135
H	-2.297295	6.057958	-0.028900
F	-2.633803	3.484521	0.040834
H	-0.066994	7.200926	-0.004847
H	1.873050	3.378242	0.160953
H	-0.351465	2.251930	0.137753
O	2.216996	6.041475	0.090345
H	2.979781	5.412351	0.137294
C	5.100022	3.974432	-0.883655
C	6.253311	3.199506	-0.859027
N	4.468590	4.371608	0.226891
C	4.973874	4.003313	1.409648
C	6.121861	3.229726	1.531214
C	6.773439	2.820583	0.373283
H	4.659241	4.293387	-1.826913
H	6.730330	2.902066	-1.787384
H	4.432590	4.345204	2.290541
H	6.493830	2.956514	2.513486
H	7.673323	2.214983	0.430418

p-F-PhOH-DMAP

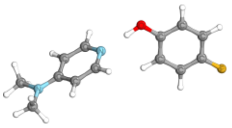

32
xyz, charge: 0, multiplicity: 1

C	-1.204299	5.229344	-0.144140
C	-1.189311	3.933416	0.344890
C	0.002846	5.861977	-0.413127
C	1.217757	5.201523	-0.193415
C	1.202369	3.891612	0.304529
C	-0.006331	3.254629	0.573749
H	-2.151939	5.731540	-0.310954
F	-2.370431	3.312377	0.607785
H	0.023867	6.876729	-0.797927
H	2.139187	3.372640	0.485174
H	-0.032198	2.240647	0.960199
O	2.358705	5.869920	-0.475820
H	3.158130	5.296693	-0.307960
C	5.009327	3.441641	-0.966115
C	6.167667	2.695746	-0.871804
N	4.632186	4.366361	-0.071704
C	5.452510	4.559752	0.970615
C	6.634449	3.872731	1.166856
C	7.032539	2.892880	0.228905
H	4.338819	3.293116	-1.811878
H	6.390644	1.974872	-1.648552
H	5.141328	5.315257	1.690752
H	7.234587	4.102530	2.038467
N	8.182912	2.177289	0.378605
C	9.085789	2.472979	1.481143
H	8.590479	2.333823	2.450889
H	9.470308	3.502514	1.433871
H	9.936129	1.790783	1.441474
C	8.596576	1.239699	-0.654850
H	7.843371	0.455642	-0.806841
H	9.524174	0.754347	-0.348389
H	8.773863	1.740091	-1.618298

p-F-PhOH-DMSO

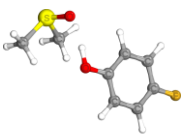

23
xyz, charge: 0, multiplicity: 1

C	1.583846	3.584272	1.070112
C	0.876483	2.810535	0.166977
C	2.461533	4.551867	0.588948
C	2.622243	4.731127	-0.789785
C	1.895244	3.935795	-1.680007
C	1.015252	2.972137	-1.201712
H	1.442796	3.434884	2.135803
F	0.019837	1.867140	0.635160
H	3.021060	5.176279	1.280067
H	2.024096	4.088556	-2.746966
H	0.440191	2.349282	-1.879284
O	3.470149	5.657131	-1.311348
H	4.014194	6.055939	-0.587182
O	5.275888	6.435701	0.546086
S	6.561657	5.646404	0.263282
C	6.903138	5.864035	-1.527012
H	6.000737	5.605900	-2.089669
H	7.748100	5.229907	-1.812293
H	7.158346	6.916032	-1.674902
C	6.077125	3.877426	0.169560
H	5.726625	3.590839	1.163956
H	6.954313	3.283258	-0.104531
H	5.272157	3.760943	-0.561806

p-F-PhOH-OPEt$_3$

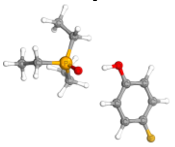

36
xyz, charge: 0, multiplicity: 1

C	2.719569	2.350120	-0.537554
C	1.773851	2.613208	0.437370
C	3.108524	3.374446	-1.396898
C	2.550668	4.652900	-1.266577
C	1.595383	4.889230	-0.272335
C	1.201478	3.866377	0.581941
H	3.139026	1.353056	-0.627366
F	1.392646	1.613714	1.275410
H	3.843509	3.189987	-2.175490
H	1.164762	5.881841	-0.184919
H	0.458954	4.033911	1.355596
O	2.897986	5.685962	-2.072790
H	3.686593	5.433206	-2.617623
O	5.277854	5.152963	-3.209998
P	6.305158	5.842777	-2.330987
C	7.963545	5.741388	-3.117072
C	6.389592	5.048685	-0.676079
C	5.872999	7.613953	-2.098361
C	6.989710	8.593892	-1.731054
H	5.057725	7.638415	-1.363033
H	5.417715	7.897170	-3.056841
H	7.462012	8.350316	-0.775962
H	6.582022	9.606188	-1.647641
H	7.769399	8.619058	-2.498452
C	9.198420	5.872360	-2.222382
H	7.975433	6.489215	-3.921183
H	7.954695	4.762637	-3.614494
H	9.249241	6.842032	-1.720574
H	10.106519	5.764584	-2.823825
H	9.220357	5.092510	-1.455411
C	6.935972	5.864390	0.497980
H	6.949245	4.112118	-0.804187
H	5.350626	4.751464	-0.475477
H	7.974385	6.172644	0.350168
H	6.895767	5.265359	1.412968
H	6.334759	6.761683	0.671366

p-F-PhOH-HMPA

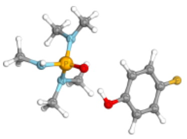

39
xyz, charge: 0, multiplicity: 1

C	1.500321	5.479961	-0.133151
C	0.969779	4.487704	0.672221
C	2.456429	5.139377	-1.085728
C	2.876983	3.810024	-1.218372
C	2.321220	2.827575	-0.392041
C	1.362027	3.164524	0.555779
H	1.162026	6.505095	-0.019152
F	0.035392	4.819857	1.601879
H	2.885259	5.900271	-1.731053
H	2.650689	1.799544	-0.506716
H	0.920558	2.412181	1.201554
O	3.816349	3.424205	-2.115377
H	4.225802	4.219741	-2.536254
O	5.121139	5.615339	-3.009609
P	6.286543	5.968627	-2.124387
N	7.672080	5.901683	-3.071269
N	6.699541	5.001494	-0.821738
N	6.014856	7.451100	-1.389304
C	6.925519	8.042099	-0.414724
H	7.584299	7.279625	0.007747
H	6.351811	8.491388	0.408791
H	7.546278	8.834378	-0.861964
C	8.967664	6.428920	-2.647004
H	9.019771	7.527806	-2.650665
H	9.752645	6.045900	-3.307730
H	9.190392	6.078778	-1.633739
C	5.846603	4.994159	0.375999
H	6.472724	4.839570	1.264634
H	5.100898	4.188474	0.328759
H	5.314622	5.941737	0.479999
H	7.467840	5.995261	-4.056847
C	7.284143	3.676496	-1.076315
H	7.929002	3.706022	-1.956412
H	6.500989	2.920590	-1.234637
H	7.885306	3.381494	-0.207022
C	5.106629	8.411923	-2.010930
H	4.443590	7.898600	-2.709975
H	5.654355	9.198591	-2.553943
H	4.495294	8.895964	-1.236865

p-F-PhOH-DIBA

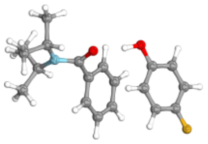

47
xyz, charge: 0, multiplicity: 1

C	-0.209140	3.197678	0.836782
C	0.222286	2.863423	-0.435934
C	0.117604	4.447228	1.351018
C	0.864417	5.351206	0.590401
C	1.267543	4.996480	-0.701048
C	0.952970	3.742923	-1.214900
H	-0.787008	2.484114	1.415460
F	-0.083045	1.635813	-0.935432
H	-0.194635	4.735597	2.349887
H	1.842019	5.701424	-1.294965
H	1.269042	3.448720	-2.210676
O	1.172113	6.548553	1.148767
H	1.905466	6.971117	0.649052
C	4.839719	4.772668	-0.847953
C	4.678011	3.401777	-0.686262
C	4.520551	5.643155	0.197016
C	4.043283	5.129897	1.403219
C	3.897566	3.757022	1.565281
C	4.208156	2.891827	0.520720
H	5.186826	5.173286	-1.796697
H	4.904998	2.730660	-1.509523
H	3.758475	5.810123	2.200605
H	3.515431	3.364037	2.502502
H	4.068860	1.821672	0.641340
C	4.509654	7.128057	-0.016086
N	5.673664	7.783396	-0.275913
C	5.530552	9.237508	-0.562905
C	5.433263	10.073250	0.717333
H	5.105568	11.086848	0.462793
H	6.394208	10.151620	1.232893
H	4.694682	9.643850	1.399171
C	6.578904	9.785230	-1.525299
H	4.558730	9.310894	-1.062106
H	6.618606	9.208773	-2.453505
H	7.581037	9.825505	-1.089916
H	6.296895	10.811741	-1.780295
C	6.977016	7.118784	-0.035093
C	7.806386	7.804676	1.052198
H	8.183436	8.781620	0.738395
H	8.673946	7.176628	1.279807
H	7.226269	7.931624	1.970806

C 7.800238 6.872636 -1.304624
H 6.707860 6.134276 0.357769
H 8.318857 7.768627 -1.651923
H 7.171780 6.500008 -2.118101
H 8.560063 6.113444 -1.090691
O 3.416346 7.718433 0.016640

p-F-PhOH-^{dipp}NHC

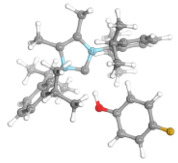

84
xyz, charge: 0, multiplicity: 1
C 7.045455 3.075197 -1.557678
C 5.855023 2.568030 -1.069708
C 8.199923 2.945071 -0.790552
C 8.161068 2.297074 0.453203
C 6.937623 1.795926 0.917711
C 5.780841 1.934578 0.160162
H 7.069221 3.576582 -2.520441
F 4.723336 2.698761 -1.814783
H 9.134171 3.360739 -1.153791
H 6.912622 1.296812 1.881471
H 4.828186 1.551155 0.512040
O 9.251590 2.149495 1.233615
H 10.114155 2.246752 0.688155
C 10.517397 4.709656 -3.752082
C 11.249254 3.752318 -3.049397
C 10.161726 5.913861 -3.157809
C 10.536184 6.185330 -1.850279
C 11.269717 5.260105 -1.104018
C 11.606168 4.048475 -1.724138
C 11.566317 2.411115 -3.683630
H 10.210786 4.506604 -4.773784
H 9.584357 6.643696 -3.718236
H 10.246754 7.128208 -1.395365
C 11.643623 5.578393 0.331310
C 12.453612 -1.240816 3.590329
C 12.121792 -1.637829 2.303666
C 12.612732 0.555876 1.494336
C 12.189334 -0.754421 1.221873
C 12.856620 0.064457 3.831760
C 12.944168 0.987427 2.789773
H 13.098352 0.372246 4.844313
C 11.718608 -1.276511 -0.129042
C 13.299069 2.435056 3.078290
H 12.391879 -1.951147 4.409868
H 11.793796 -2.658586 2.123276
C 10.331441 1.499396 -3.627958
H 12.354444 1.931294 -3.092264
C 12.084495 2.542278 -5.120486
C 12.493928 6.853318 0.417852
H 12.245716 4.750530 0.722309
C 10.388020 5.689987 1.207548
H 13.386872 6.782161 -0.212385
H 12.815595 7.021405 1.451425
H 11.928095 7.735486 0.099370
H 9.741926 6.506452 0.866096
H 10.668448 5.894595 2.246458
H 9.806166 4.763439 1.187270
H 9.977453 1.372679 -2.600958
H 10.566281 0.510814 -4.038545
H 9.510021 1.928547 -4.213256
H 11.301041 2.885096 -5.804389
H 12.427330 1.567544 -5.483143
H 12.919885 3.247564 -5.186266
C 12.041014 3.189579 3.538028
H 13.638672 2.901358 2.145991
C 14.429137 2.586568 4.102541
C 10.260927 -0.865556 -0.390609
H 11.729013 -2.370393 -0.023655
C 12.606742 -0.956083 -1.337082
H 9.613692 -1.151310 0.444142
H 9.890532 -1.348694 -1.302093
H 10.179340 0.217696 -0.513026
H 12.536766 0.091663 -1.638017
H 12.282857 -1.564014 -2.189009
H 13.657964 -1.192158 -1.140811
H 14.111145 2.287302 5.106732
H 14.738156 3.635453 4.161901
H 15.304339 1.985502 3.833213
H 11.238086 3.117345 2.798312
H 12.271505 4.248281 3.705210
H 11.667126 2.768168 4.478230
N 12.314810 3.059460 -0.964303
C 13.695429 2.845550 -1.042546
C 13.967933 1.856011 -0.148764
N 12.740055 1.513284 0.430136
C 11.707926 2.257899 -0.048342
C 14.573317 3.622744 -1.954443
H 14.339238 3.428157 -3.007832
H 15.620639 3.362954 -1.785582
H 14.456608 4.700238 -1.791486
C 15.245787 1.205983 0.238186
H 15.521167 1.444113 1.272678
H 16.054774 1.539450 -0.415634
H 15.174010 0.115416 0.168337

p-F-PhOH-SIMes

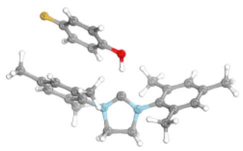

62
xyz, charge: 0, multiplicity: 1
O 0.05389 2.67990 -1.33721
C 0.74593 3.21333 -0.30734
C 2.14529 3.16703 -0.33819
C 0.11165 3.81219 0.79086
C 0.85673 4.32454 1.84711
C 2.23663 4.25075 1.78883
C 2.89642 3.69005 0.70895
H 2.63160 2.70727 -1.19321
H -0.97309 3.86187 0.81693
H 0.37510 4.77538 2.70905
H 3.98145 3.65847 0.69630
F 2.96886 4.74671 2.82711
C -2.14632 1.30846 -0.37056
N -2.23006 0.79180 0.86389
C -3.31987 -0.20918 1.01111
C -4.13443 0.03349 -0.26094
H -3.89404 -0.04306 1.92705
H -2.88817 -1.21933 1.05086
N -3.17968 0.82953 -1.07972
H -5.04169 0.62634 -0.07590
H -4.42390 -0.89103 -0.76819
H -0.82118 2.26506 -0.99028
C -3.52764 1.27779 -2.38870
C -3.53455 0.35572 -3.44353
C -3.90416 0.80018 -4.71279
C -4.25305 2.12710 -4.95143
C -4.22909 3.02142 -3.88199
C -3.87185 2.62245 -2.59565
H -4.49755 4.06223 -4.05209
H -3.90352 0.09097 -5.53831
C -4.60935 2.59622 -6.33565
H -4.98152 1.77368 -6.95279
H -3.73088 3.01721 -6.83975
H -5.37430 3.37798 -6.30591
C -3.13285 -1.07873 -3.23601
H -2.35090 -1.16654 -2.47574
H -2.75767 -1.50885 -4.16826
H -3.98224 -1.69524 -2.91504
C -3.84509 3.62547 -1.47631
H -4.41542 4.51745 -1.74831
H -2.81611 3.93178 -1.25504
H -4.26245 3.21580 -0.55069
C -1.22276 0.95634 1.86302
C 0.06325 0.44223 1.64236
C 1.03965 0.67756 2.60780
C 0.76715 1.39046 3.77346
C -0.52968 1.85732 3.97701
C -1.53602 1.65871 3.03362
H 2.04739 0.30583 2.43423
H -0.75814 2.42089 4.87976
C -2.90694 2.23478 3.26089
H -3.59658 1.49159 3.68114
H -3.34580 2.59993 2.32654
H -2.86051 3.06676 3.96847
C 0.40798 -0.31827 0.39273
H -0.39856 -0.99500 0.09186
H 1.31464 -0.90916 0.54707
H 0.58501 0.36572 -0.44558
C 1.86155 1.69573 4.75754
H 2.58423 0.87636 4.81733
H 1.46131 1.87838 5.75893
H 2.40880 2.59488 4.44746

p-F-PhOH-^{iPr}NHC

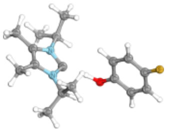

46
xyz, charge: 0, multiplicity: 1
N -0.09352 1.45295 0.35349
C 0.48719 0.26996 0.67487
C 0.75383 2.25272 -0.41660
C 1.90952 1.53850 -0.57328
N 1.71711 0.33457 0.10695
C 0.44415 3.62940 -0.89534
H -0.54294 3.69668 -1.36117
H 1.17802 3.93469 -1.64475
H 0.48048 4.36509 -0.08351
C 3.16371 1.94968 -1.26501
H 2.97078 2.81957 -1.89704
H 3.56043 1.16056 -1.90921
H 3.95141 2.22843 -0.55556
C -1.48656 1.71706 0.76037
C -1.63698 3.01988 1.54247
H -0.87609 3.09477 2.32514
H -2.62118 3.03812 2.02060
H -1.56898 3.90113 0.89885
C -2.44425 1.62334 -0.42625
H -1.70539 0.88403 1.43879
H -2.28947 2.43307 -1.14586
H -3.47727 1.68787 -0.06992
H -2.31945 0.66898 -0.94603
C 2.63334 -0.82091 0.20624
C 2.74993 -1.56306 -1.12338
H 3.31530 -2.48831 -0.97357
H 3.27128 -0.97228 -1.88303
H 1.75896 -1.83208 -1.49898
C 3.98908 -0.44329 0.79869
H 2.12423 -1.48204 0.91688
H 4.52419 -1.35779 1.07220
H 3.86615 0.16254 1.70163
H 4.61607 0.10344 0.08894
O -0.31588 -2.01425 1.95785
C -0.41938 -3.01516 1.06489
H -0.09974 -1.11858 1.47784
C -0.21910 -4.33211 1.50537
C -0.32160 -5.40198 0.62470
C -0.61313 -5.15133 -0.70624
C -0.81912 -3.86731 -1.17721
C -0.73011 -2.79879 -0.28851
H 0.01587 -4.49768 2.55230
H -1.06012 -3.70972 -2.22414
H -0.90707 -1.78624 -0.63810
H -0.17181 -6.42362 0.95987
F -0.70683 -6.19752 -1.57162

p-F-PhOH-PBu_3

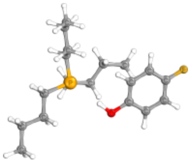

53
xyz, charge: 0, multiplicity: 1
P -2.16841 3.56209 2.22918
C -1.11364 2.03406 1.99758
C -3.61187 3.20724 1.10225

C -2.94001 3.19466 3.88566
C -4.60560 4.36725 1.01508
H -4.11685 2.29091 1.44231
H -3.20529 2.99319 0.10556
C -2.01753 3.53720 5.05743
H -3.23786 2.13601 3.92219
H -3.86115 3.78800 3.95950
C 0.06827 2.23970 1.04335
H -1.74922 1.20208 1.66031
H -0.73643 1.76186 2.99156
C -0.33756 2.54622 -0.39717
H 0.69764 3.05822 1.41946
H 0.69323 1.33536 1.05444
C -2.64604 3.22001 6.41545
H -1.75466 4.60307 5.01412
H -1.06937 2.98848 4.96570
C -5.77773 4.06887 0.07853
H -4.08385 5.27085 0.66963
H -4.99770 4.60656 2.01355
C -6.76477 5.23028 -0.01426
H -6.29766 3.16613 0.42737
H -5.38742 3.83161 -0.92068
H -7.18859 5.46548 0.96854
H -7.59453 4.99768 -0.68902
H -6.27276 6.13517 -0.38809
C -1.72568 3.56982 7.58272
H -2.90523 2.15301 6.45363
H -3.59254 3.76969 6.51090
H -1.47470 4.63636 7.57883
H -2.19373 3.33915 8.54493
H -0.78634 3.00862 7.52396
C 0.86818 2.76481 -1.30685
H -0.96184 1.72688 -0.78297
H -0.95837 3.45233 -0.41492
H 1.51147 1.87744 -1.32952
H 0.56086 2.98578 -2.33383
H 1.46910 3.60953 -0.95171
H -0.58682 5.33406 2.12844
O 0.28988 5.77761 2.07769
C 0.57466 6.06979 0.78009
C 1.91136 6.29299 0.43971
C -0.41401 6.16897 -0.20435
C -0.06786 6.46557 -1.51943
C 1.26378 6.67854 -1.82905
C 2.25974 6.60513 -0.86911
H 2.66907 6.21638 1.21305
H -1.45580 6.01681 0.06407
H -0.82188 6.54211 -2.29625
H 3.29315 6.78134 -1.14970
F 1.60356 6.97156 -3.10978

p-F-PhOH-PCy$_3$

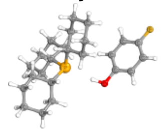

65
xyz, charge: 0, multiplicity: 1
P -1.43157 3.32317 1.94789
C -0.95683 1.65004 1.23301
C -3.10344 3.75784 1.18271
C -2.02850 3.00786 3.70625
C -3.38721 5.24572 1.46261
C -0.89786 3.11501 4.74262
C -0.28531 1.85212 -0.14066
C 0.11299 0.51646 -0.77558
H -0.93088 2.40919 -0.82500
H 0.61755 2.46365 0.00780
C -1.45724 3.07124 6.16848
H -0.33599 4.04323 4.58092
H -0.18462 2.29068 4.61239
C -4.77366 5.65726 0.96015
H -2.62650 5.85155 0.95098
H -3.29502 5.46945 2.53286
C -4.91674 5.36726 -0.53399
H -5.54561 5.10456 1.51608
H -4.93779 6.72298 1.16120
C -2.30401 1.81774 6.39603
H -0.63559 3.11618 6.89391
H -2.07856 3.96286 6.33861
C 1.06125 -0.26290 0.13502
H -0.79216 -0.08054 -0.96051
H 0.57920 0.69478 -1.75249
H -0.08537 5.25274 1.83818
O 0.18748 6.20118 1.83792
C -0.06553 6.74062 0.61638
C -0.23753 8.12503 0.53331
C -0.15080 5.96519 -0.54527
C -0.43105 6.56271 -1.77112
C -0.60128 7.93477 -1.82240
C -0.50049 8.72798 -0.69087
H -0.16757 8.71678 1.44049
H 0.00615 4.89235 -0.48584
H -0.50275 5.97460 -2.68063
H -0.63620 9.80166 -0.77194
F -0.86916 8.52191 -3.01673
C -4.63149 3.89429 -0.82671
H -4.20231 5.99179 -1.09046
H -5.91972 5.63916 -0.88523
C -3.24535 3.47898 -0.31843
H -4.70321 3.69550 -1.90334
H -5.39544 3.27187 -0.33746
H -2.48186 4.04686 -0.86989
H -3.08432 2.41550 -0.53068
H -3.86711 3.15600 1.70098
C 0.44912 -0.44268 1.52374
H 2.00752 0.28994 0.22593
H 1.30469 -1.23764 -0.30517
C 0.03376 0.89835 2.13975
H 1.15596 -0.95127 2.19122
H -0.43730 -1.08976 1.44939
H 0.92552 1.52608 2.28886
H -0.39211 0.71921 3.13000
H -1.86155 1.03271 1.10870
C -3.43427 1.72652 5.36917
H -2.71321 1.81298 7.41380
H -1.66381 0.92720 6.30798
C -2.88493 1.75360 3.93748
H -4.12065 2.57451 5.50920
H -4.02247 0.81354 5.52584
H -2.68547 3.87707 3.87932
H -3.71359 1.71051 3.22051
H -2.28521 0.84913 3.77534

p-F-PhOH-DABCO

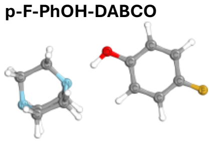

33
xyz, charge: 0, multiplicity: 1
N 4.03078 2.34807 0.10203
O 1.77643 1.26472 -1.00382
C 1.36216 0.05984 -0.55052
C 2.08344 -0.68908 0.38771
C 1.61053 -1.92503 0.82085
C 0.41789 -2.40219 0.30855
C -0.31590 -1.68465 -0.62217
C 0.15896 -0.45159 -1.05040
H 3.02186 -0.30521 0.77785
H -1.24745 -2.09116 -1.00285
H -0.39458 0.13345 -1.77796
H 2.16071 -2.51490 1.54698
F -0.04647 -3.60781 0.72859
C 3.82483 2.72709 1.51745
C 4.09755 3.58094 -0.71708
C 5.31555 1.62571 -0.01921
C 5.03621 3.57076 2.01704
H 3.70062 1.80858 2.10441
H 2.88375 3.28665 1.57912
C 5.22264 4.51187 -0.17552
H 3.11356 4.06379 -0.68305
H 4.28102 3.28025 -1.75557
C 6.48733 2.56397 0.40142
H 5.41801 1.28198 -1.05559
H 5.26466 0.73623 0.62092
N 5.96476 3.84405 0.90729
H 5.59151 3.04218 2.80129
H 4.70565 4.52883 2.43586
H 4.80795 5.44533 0.22369
H 5.93226 4.77862 -0.96796
H 7.14715 2.77887 -0.44776
H 7.09964 2.10502 1.18699
H 2.62547 1.54522 -0.55077

7 References

1 D. O. Soloviev and C. A. Hunter, *Chem. Sci.*, 2024, **15**, 15299-15310.
2 N. Ansmann, D. Hartmann, S. Sailer, P. Erdmann, R. Maskey, M. Schorpp and L. Greb, *Angew. Chem. Int. Ed.*, 2022, **61**, e202203947.
3 F. Neese, F. Wennmohs, U. Becker and C. Riplinger, *J. Chem. Phys.*, 2020, **152**, 224108.
4 F. Weigend, *Phys. Chem. Chem. Phys.*, 2006, **8**, 1057-1065.
5 S. Grimme, A. Hansen, S. Ehlert and J. M. Mewes, *J. Chem. Phys.*, 2021, **154**, 064103.
6 S. Grimme, S. Ehrlich and L. Goerigk, *J. Comput. Chem.*, 2011, **32**, 1456-1465.
7 S. Kozuch, D. Gruzman and J. M. L. Martin, *J. Phys. Chem. C*, 2010, **114**, 20801-20808.
8 C. C. Pye, T. Ziegler, E. van Lenthe and J. N. Louwen, *Can. J. Chem.*, 2009, **87**, 790-797.
9 F. Weigend and R. Ahlrichs, *Phys. Chem. Chem. Phys.*, 2005, **7**, 3297-3305.
10 A. V. Marenich, C. J. Cramer and D. G. Truhlar, *J. Phys. Chem. B*, 2009, **113**, 6378-6396.
11 S. Grimme, *Chem. Eur. J.*, 2012, **18**, 9955-9964.
12 S. Hirata and M. Head-Gordon, *Chem. Phys. Lett.*, 1999, **314**, 291-299.
13 Y. S. Lin, G. D. Li, S. P. Mao and J. D. Chai, *J. Chem. Theory Comput.*, 2013, **9**, 263-272.
14 C. Adamo, M. Cossi and V. Barone, *J. Mol. Struct. Theochem.* , 1999, **493**, 145-157.
15 E. D. Glendening, C. R. Landis and F. Weinhold, *J. Comput. Chem.*, 2013, **34**, 1429-1437.
16 H. Bohrer, N. Trapp, D. Himmel, M. Schleep and I. Krossing, *Dalton Trans.*, 2015, **44**, 7489-7499.
17 P. Erdmann and L. Greb, *Chemphyschem*, 2021, **22**, 935-943.
18 P. Erdmann, J. Leitner, J. Schwarz and L. Greb, *Chemphyschem*, 2020, **21**, 987-994.
19 T. Thorwart, D. Hartmann and L. Greb, *Chem. Eur. J.*, 2022, **28**, e202202273.
20 R. Maskey, M. Schädler, C. Legler and L. Greb, *Angew. Chem. Int. Ed.*, 2018, **57**, 1717-1720.
21 R. Lo, D. Manna, M. Lamanec, M. Dracínsky, P. Bour, T. Wu, G. Bastien, J. Kaleta, V. M. Miriyala, V. Spirko, A. Masínová, D. Nachtigallová and P. Hobza, *Nat. Commun.*, 2022, **13**, 2107.
22 D. Manna, R. Lo, D. Nachtigallová, Z. Trávnícek and P. Hobza, *Chem. Eur. J.*, 2023, **29**, e202300635.
23 A. V. Levanov, U. D. Gurbanova, O. Y. Isaikina and V. V. Lunin, *Russ. J. Phys. Chem. A*, 2019, **93**, 93-101.
24 E. A. Betterton and J. L. Robinson, *J. Air Waste Manag. Assoc.*, 1997, **47**, 1216-1219.
25 Y. Chiang and A. J. Kresge, *Can. J. Chem.*, 2000, **78**, 1627-1628.
26 J. H. Boughton and R. N. Keller, *J. Inorg. Nucl. Chem.*, 1966, **28**, 2851-2859.
27 P. Mech, M. Bogunia, A. Nowacki and M. Makowski, *J. Phys. Chem. A*, 2020, **124**, 538-551.
28 E. A. Castro, M. Aliaga and J. G. Santos, *J. Org. Chem.*, 2004, **69**, 6711-6714.
29 A. Bagno and G. Scorrano, *J. Am. Chem. Soc.*, 1988, **110**, 4577-4582.
30 W. A. Henderson and C. A. Streuli, *J. Am. Chem. Soc.*, 1960, **82**, 5791-5794.
31 V. K. Aggarwal, I. Emme and S. Y. Fulford, *J. Org. Chem.*, 2003, **68**, 692-700.
32 *S.* Bruker, *V8.40B*, Bruker AXS Inc., Madison, Wisconsin, USA.
33 L. Krause, R. Herbst-Irmer, G. M. Sheldrick and D. Stalke, *J. Appl. Crystallogr.*, 2015, **48**, 3-10.
34 G. M. Sheldrick, *Acta Crystallogr. C.*, 2015, **71**, 3-8.
35 G. M. Sheldrick, *Acta Crystallogr. A.*, 2015, **71**, 3-8.
36 C. R. Groom, I. J. Bruno, M. P. Lightfoot and S. C. Ward, *Acta Crystallogr. B.*, 2016, **72**, 171-179.
37 F. D. Kratzert, V146, https://dkratzert.de/finalcif.html.
